# Which type of social activities may reduce cognitive decline in the elderly?: a longitudinal population-based study

**DOI:** 10.1186/s12877-016-0343-x

**Published:** 2016-09-27

**Authors:** Seung Hee Lee, Young Bum Kim

**Affiliations:** 1 Department of Nursing, University of Ulsan, Ulsan, Republic of Korea; 2 Hallym University Institute of Aging, Chuncheon, Republic of Korea

**Keywords:** Cognitive impairment, Social activity, Elderly, Korea

## Abstract

**Background:**

Previous studies have found that social activities are beneficial for the reduction of cognitive decline (CD) in the elderly. However, knowledge regarding the types of social activities that reduce CD in later life is limited. The aim of this study is to examine which type of social activities reduce CD 4 years later among young-old (Y-O) and old-old (O-O) adults.

**Methods:**

We conducted a secondary analysis using data from cognitively intact adults 65 years of age or older who participated in the Korean Longitudinal Study of Aging (KLoSA). Cognitive function was assessed using the Korean version of the Mini-Mental State Examination (MMSE). We computed CD between 2008 and 2012 by subtracting the Wave 4 MMSE score from the Wave 2 MMSE score. Multivariate linear regression analysis was conducted regarding the effects of social activities on CD after adjusting for age, sex, education, income, marital status, activities of daily living (ADL), instrumental activities of daily living (IADL), chronic diseases, quality of life, depressive symptom, change in depressive symptom, and cognitive functioning at baseline.

**Results:**

Subjects who participated in senior citizen clubs or senior centers at baseline had a lower risk of CD 4 years later than those who did not in Y-O adults. Frequent contact with offspring by phone or letters was associated with reduced CD in O-O adults. Frequent face-to-face contact with offspring was positively associated with CD in O-O adults. Participating in two or more formal social activities was associated with reduced CD compared with nonparticipation in O-O adults.

**Conclusion:**

Encouraging older adults to participate in senior citizen clubs or to have frequent contacts with adult children by phone or letters may help reduce CD in later life among older adults. Participation in a variety of formal social activities may also have a beneficial effect on preventing CD in older adults.

**Electronic supplementary material:**

The online version of this article (doi:10.1186/s12877-016-0343-x) contains supplementary material, which is available to authorized users.

## Background

The number of people living with dementia in 2015 was estimated at 47.5 million globally, and that number is expected to double by 2030, reaching 135.5 million by 2050 [1]. The prevalence of dementia increases with age, with a risk of the disease doubling every five years after 65 [2].

Both cognitive impairment and social disengagement are prevalent concomitants of old age [3]. Cognitive impairment has been associated with adverse physical and psychological changes including self-rated health, disability, quality of life, and mortality [3, 4]. Most epidemiological studies on cognitive function have shown that health factors, including chronic diseases (e.g., hypertension, diabetes, and cardiovascular disease), lower physical function, and depressive symptoms predict risk of CD [5, 6]. Social factors including old age, sex, marital status, education, socioeconomic position, and social isolation are also associated with CD [3, 7]. Cognitive impairment can cause severe physical, psychological, and socioeconomic burdens not only for the individuals affected, but also their families and society [4, 8]. Finding a good population-based strategy for preventing or delaying CD is a challenging public health priority for the aging society [9]. Numerous studies have shown that social activities are beneficial to various health outcomes of elderly people including self-rated health, physical functioning, depressive symptoms, and quality of life [10–12]. Previous research has also shown that greater participation in social activities can reduce CD in the elderly by providing intellectual and emotional stimulation [13–17]. Reportedly, intellectual stimulation through social activities can maintain cognitive function by increasing cognitive reserve capacity [13, 18]. Participation in social activities can also provide meaningful social roles, self-esteem, and social competence, which might protect against neuropathology including reduction of the stress response [19, 20]. Although social activity is widely believed to guard against CD in later life, knowledge regarding the types of protective social activity is limited.

Social activities can be classified into two categories (formal and informal) based on intimacy and intensity [21]. Formal activity describes an activity with formal organizations such as an alumni society, based on specific objectives and focusing on the achievement of a goal. Conversely, informal activity includes interactions with family, friends, and neighbors. It has universal character in objectives and focuses on emotional function [19, 21]. There may be some differences among activities classified as informal. In later life, there may be some differences in relationships between close friends and children [22]. Despite the intimacy on which both types of relationships are based, the children-parent relationship is dominated by the kinship model and family norms, while the relationship with friends is based on the egalitarian relationship model and voluntarism [22]. Furthermore, due to the differences in the norms on which these relationships are based, the mental health of the elderly may be affected differently [12, 22–24].

A growing body of literature has shown that type of social activity can differentially affect morale, mental health, and survival in old age [10, 12, 19, 24]. For example, a previous study reported that informal social activities such as ‘face-to-face talks’ and ‘phone conversations’ with friends were significantly related to decreased mortality risk in later life after controlling for relevant confounders [25]. Several researchers have reported that risk of late-life depression showed an inverse association with frequent participation in informal activities such as contact with offspring by phone or letters; however, no significant association with participation in formal activities such as volunteering and attending an alumni society was noted [12]. Another study among older Chinese people indicated that playing Mahjong, chess or card games; interacting with friends; and having an activity center in the community are more beneficial for maintaining episodic memory than are other social activities such as volunteering [26]. A 20-year follow-up study of Swedish samples showed that prior political activities or mental activities were associated with late-life cognition after adjusting for covariates, but activities in organization were not [27]. However, knowledge regarding the effects of different types of social activities on CD in older people is limited. Due to the different natures of various social activities, all types of social activities might not have a beneficial effect on cognitive function among older people. For example, in Asian countries, because of traditional filial piety, familism, and lack of a welfare system, the types of social activity beneficial to cognitive function in older age might be different than those in the Western world.

In the industrialized nations, most people 65–74 years of age are still living independently and are in relatively good health [28]. However, the status of adults 75 years of age and older becomes increasingly worse in terms of mental and physical health [28]. Many gerontologists have also suggested that older adults are not a homogeneous group [28, 29]. Based on the physical and mental differences in later life depending on age group, numerous studies on CD have divided older adults into two or more age groups [29–31].

The aim of this study is to investigate which types of social activity reduce cognitive decline 4 years later among young-old (Y-O) (age 65–74 years) and old-old (O-O) (age ≥ 75 years) adults using data from a longitudinal study of a community-dwelling Korean population.

## Methods

### Study design and participants

The present study employed a secondary analysis approach using data from the Korean Longitudinal Study of Aging (KLoSA), an ongoing panel survey examining the socioeconomic, physical, and psychological aspects of aging among community-dwelling Koreans 45 years of age or older. The sample was stratified based on age and sex; to ensure national representation, participants were selected randomly using a multistage, stratified, probability sampling design from household units selected according to geographical area, including both urban and rural areas. The survey began in 2006, and follow-up interviews were conducted every 2 years (in 2008, 2010, 2012). Before the data collection, all participants provided written informed consent. More sampling design details and KLoSA methods have been presented elsewhere [12, 24].

We did not consider Mini-Mental State Examination (MMSE) scores from Wave 1 (2006) in the analysis because of the possibility of inaccurate measurements caused by stress or a learning effect from the first survey [32]. In the present study, we used data from Wave 2 (2008) and Wave 4 (2012) of the KLoSA. Of 10,254 Wave 1 subjects, 8,688 individuals completed Wave 2 interviews conducted by a skilled interviewer in 2008. To focus on the influence of social activities on CD in elderly people, we excluded subjects younger than 65 years (*n* = 3,065) from the Wave 2 data (*n* = 8,688). Because impaired cognition can limit social activity of individuals [13], subjects whose MMSE scores were lower than 24 (*n* = 1,880) were excluded. Subjects who had missing social activity and income (*n* = 174) and depression data (*n* = 51), were without children, or cohabited with all children (*n* = 1,690) were also excluded. Of the remaining 1,828 Wave 2 subjects who met the criteria for inclusion, 1,568 completed the Wave 4 interviews in 2012. Attrition between Wave 2 and Wave 4 was caused mostly by refusal, failure to contact, and mortality (260 were lost to follow-up). Finally, we analyzed data from 1,568 subjects who participated in both Wave 2 (2008) and Wave 4 interviews. Differences in education and MMSE scores between subjects lost to follow-up and those completing the interview at Wave 4 were not significant. However, individuals lost to follow-up were significantly older, included a greater proportion of males, and met with close friends less frequently than those completing the interview.

### Measures

Cognitive function was assessed using the Korean version of the MMSE [33], which is widely used for assessing global cognitive function. The MMSE tests cognitive functions including orientation, recall, language, registration, attention, and calculation, as well as the ability to follow simple commands [33, 34]. Total scores range from 0 to 30, with higher scores indicating better cognition. Psychometric properties of the MMSE have been validated in previous studies conducted in Korean people [33, 35]. The dependent variable in the study was CD over time. We computed CD between 2008 and 2012 by subtracting Wave 4 MMSE scores from Wave 2 MMSE scores.

Social activities were divided into formal and informal based on intimacy and intensity of participation required [21]. Three variables representing formal social activities were employed at baseline: (a) level of participation in church or other religious groups, (b) level of participation in senior citizen clubs or senior centers, and (c) level of participation in alumni societies or family councils. Participants rated how often they participated in each activity based on a 10-point scale (0, never; 1, almost never; 2, less than once a year; 3, 1–2 times a year; 4, 3–4 times a year; 5, 5–6 times per year; 6, once a month; 7, twice a month; 8, once a week; 9, 2–3 times a week; and 10, every day or almost every day). Three variables representing informal social activities were employed at baseline: (a) level of face-to-face contact with close friends, (b) level of face-to-face contact with children, and (c) level of contact with children by phone or letters [19]. Participants answered the following three questions: 1) how often do you meet with your close friends?; 2) how often do you meet with your adult children?; and 3) how often do you contact your children by phone or letter?) using the same 0–10-point scale.

Because, the frequency of subjects who were involved in formal social activities was not normally distributed, a dichotomous variable was considered for formal social activity variables; participation or no participation. The total number of formal social activities that each subject participated in was summed and categorized as 0 (no participation), 1, or ≥2.

Sociodemographic variables, health-related variables, and mental well-being variables were considered as potentially confounding variables in the present study. Sociodemographic variables included age, sex, marital status, education, monthly household income, living arrangement, and residential area. Health-related variables included comorbidities (hypertension, diabetes mellitus, heart disease, stroke, cancer, and hearing problems), activities of daily living (ADL), and instrumental activities of daily living (IADL). Mental well-being variables included quality of life and depressive symptom. Age was classified into the following two groups: Y-O (65–74 years) and O-O (≥75 years) [30, 36]. Monthly household income was calculated as the total household income divided by the square root of the number of household members. These scores were then divided into quartiles (e.g., <250,000 South Korean won [KRW], 250,000 KRW–1,000,000 KRW, 1,000,000 KRW–2,500,000 KRW, and >2,500,000 KRW). Subjects were asked if they were ever diagnosed with hypertension, diabetes mellitus, heart disease (congestive heart failure or myocardial infarction), stroke, and/or cancer by a physician. Subjects were also asked if they had hearing difficulties. Responses were coded ‘yes’ or ‘no’. ADL was measured using the 7-item Korean Activities of Daily Living Scale. The scale quantifies the ability to independently perform (no help, some help, unable to do) the following seven activities: dressing, washing, bathing or showering, eating, getting out of bed and walking across a room, controlling urination and defecation, and using the toilet. IADL were assessed using the 10-item Korean Instrumental Activities of Daily Living Scale, which quantifies the ability to independently perform the following 10 activities: personal grooming, household chores, preparing meals, doing laundry, going out a short distance, using transportation, shopping, managing finances, making phone calls, and taking medications. These two measures (ADL/IALD) have been widely used and are known to have acceptable reliability [12, 24]. Respondents needing help with or unable to perform one or more activities were considered to be functionally dependent in that activity (ADL or IADL). Quality of life was assessed with a single question using the visual analog scale. Participants answered the following question phrased as, “how is your overall quality of life?” They rated their quality of life on a scale from 0 (worst state) to 100 (best state), with a higher score indicating a higher level of quality of life. Depressive symptom was assessed with the 10-item short-form Center for Epidemiological Studies Depression (CES-D10) Scale, which is widely used to detect depressive symptoms in nonpsychiatric community settings [24, 37]. The CES-D10 assesses self-reported depressive symptoms experienced during the two weeks prior to testing; the scale includes two positively phrased items (“feel pretty good” and “generally satisfied”) and eight negatively phrased items (e.g., “feel depressed” and “feel afraid”). Each item is scored from 0 to 3; (0) very rarely or less than once per day; (1) sometimes or 1–2 days during the past week; (2) often or 3–4 days during the past week; and (3) almost always or 5–7 days during the past week. The positively phrased items were reverse-coded. Total scores ranged from 0–30, with higher scores indicating more severe depression symptom. Because late life depressive symptom tends to increase slightly over time [38] and elevated depressive symptom would lead to larger CD [39], a change in depressive symptom was also included in the analyses. A change in depressive symptom was calculated by dividing the differences of depressive symptom score between Wave 2 and Wave 4 by Wave 2 depressive symptom score. This means the ratio of changes in depressive symptom between wave 2 and wave 4. A higher score indicates more elevated depressive symptom.

### Statistical analysis

Baseline characteristics of the samples were summarized as frequency, percentage, and mean (with standard deviation; SD) by age group. Chi-square (χ2) and t-tests were conducted to compare the sociodemographic distributions, health-related characteristics, social activities, and cognitive function by age group. First, we examined associations among study variables using Pearson’s correlation. Next, multivariate linear regression analysis regarding the effects of social activity on the continuous CD between 2008 and 2012 was performed after controlling for potentially confounding variables and MMSE score at baseline. Social activity at baseline was used as the independent variable. We also conducted multivariate linear regression analysis separately by age group to investigate any differences in relationships between CD over time and social activities by age group. Model validation was performed by checking outliers and test of the linearity assumption. There was no evidence of nonlinearity. Multicollinearity was checked for all study variables by using correlations, tolerances and variance inflation factors (VIF) (data shown in Additional file 1: Table S5 and Additional file 1: Table S6). The correlations were sufficiently low (*r* < 0.71) and all VIF scores were below 2.5, allowing for entry of all variables into multivariate regression models [40]. Because violation of homoscedasticity was noted in regression, the Huber-White sandwich robust standard error (SE) were used in the multivariate linear regression analyses. Statistical analyses were performed using Stata (version 14, Stata Corp., College Station, TX, USA) software. All analyses reported were two-tailed with α set at 0.05.

## Results

The study population consisted of 1568 non-demented community-dwelling subjects who had good cognition at baseline (MMSE score ≥ 24). Additional file 1: Table S1 shows the characteristics, social activities, and cognitive function levels of the subjects at baseline and CD 4 years later. O-O adults were more likely to be bereaved, less educated, more dependent in IADL, have lower household incomes, and have more comorbidities than Y-O adults. O-O adults had lower MMSE scores in 2008 and 2012 than Y-O adults. The average CD 4 years later was more pronounced in O-O adults than Y-O adults (Y-O adults, mean = 2.18; O-O adults, mean = 4.59; *P* < 0.001). CD in MMSE sub-scores was also more pronounced in O-O adults than Y-O adults (Additional file 1: Table S2 and Additional file 1: Table S3).

Overall, the subjects were more involved in informal social activities (e.g., face-to-face contact with close friends) than formal social activities (e.g., participating in alumni societies or family councils). The percentages of subjects who were involved in informal social activities ranged from 99.6 to 100.0 % (contact with close friends, 100.0 %; contact with one’s children, 99.6 %; contact with one’s children by phone or letter, 99.7 %). The levels of participation in informal social activities were different between Y-O adults and O-O adults. O-O adults had significantly less frequent phone and face-to-face contacts with their children compared with Y-O adults. The percentages of subjects who were involved in formal social activities ranged from 13.7 to 59.8 % (church or other religious groups, 24.9 %; senior citizen clubs or senior centers, 59.8 %; alumni societies or family councils, 13.7 %). Subjects who participated in senior citizen clubs were more likely to be men, younger and married, they had better ADL compared with those who did not. The majority of subjects who were involved in religious activity were women (Additional file 1: Table S4).

Additional file 1: Table S7 shows the results of the multivariate linear regression analysis with social activities at baseline as the independent variable and CD 4 years later as the dependent variable, controlling for sociodemographics, health-related characteristics, mental well-being (quality of life and depressive symptom), and cognitive function at baseline. We investigated the types of social activity that reduce the risk of CD over time. In formal social activities, subjects who participated in senior citizen clubs or senior centers at baseline had a lower risk of CD 4 years later than those who did not in Y-O adults and total sample (Y-O adults, B = − 0.80, SE = 0.40, *p* = 0.045; total sample, B = − 0.95, SE = 0.37, *p* = 0.012). In informal social activities, more frequent contact with children by phone or letters was associated with reduced CD in O-O adults (B = − 0.95, SE = 0.45, *p* = 0.038) but not in Y-O adults (B = − 0.14, SE = 0.15, *p* = 0.343). More frequent face-to-face contact with children was positively associated with CD in O-O adults (B = 1.54, SE = 0.45, *p* = 0.001) but not in Y-O adults (B = 0.08, SE = 0.15, *p* = 0.589). After adjustment for covariates, participating in two or more formal social activities was significantly associated with lower CD compared with nonparticipation in O-O adults (B = −3.17, SE = 1.26, *p* = 0.012) (Table 1).

**Table 1:** Multivariate linear regression analysis of the associations between the number of formal social activities and cognitive decline 4 years later

Variable	Total	Young-old	Old-old
	Coefficients (SE)	Coefficients (SE)	Coefficients (SE)
The number of formal social activities			
Zero	Ref.	Ref.	Ref.
One	−0.64(0.42)	−0.18(0.43)	−1.96(1.05)
Two or more	−0.94(0.49)	−0.25(0.50)	−3.17(1.26)*
Adjusted R-square	0.07	0.06	0.13
*F*-value	4.33***	3.13***	2.26**

*Note*: Adjusted for age, sex, marital status, education, household income, living arrangement, residential area, comorbidities, activities of daily living (ADL), instrumental activities of daily living (IADL), depressive symptom, and baseline Mini-Mental State Examination (MMSE) score. *SE* standard error

*p < 0.05, **p < 0.01, ***p < 0.001

As expected, in the study sample, older age and being dependent in ADL was positively associated with CD. These results were similar when the analyses were repeated separately for Y-O adults and O-O adults. Subjects with elevated depressive symptom over time or high levels of depressive symptom at baseline had higher risk of CD after adjusting for related covariates. Subjects with higher level of quality of life had a lower risk of CD. Subjects with low cognitive level at baseline had higher risk of CD.

Additionally, we analyzed which type of social activity can mediate associations between CD and depressive symptom using sobel tests. The depressive symptom at baseline did not significantly predict the level of social activities (B = −0.01, SE = 0.01, *p* = 0.490, for level of face to face contact with one’s children; B = −0.01, SE = 0.02, *p* = 0.136, for level of contact with one’s children by phone or letter; B = 0.01, SE = 0.02, *p* = 0.706, for participation in senior citizen clubs or senior centers) and so the conditions for mediation were not met.

## Discussion

Using community-based longitudinal data of people whose initial cognition level was good, this study examined the types of social activities that can reduce cognitive decline among Y-O and O-O adults 4 years later. Several important findings emerged from our study. We found that older adults who participated more frequently in senior citizen clubs or senior centers at baseline had lower risk of CD 4 years later than those who did not. This association was independent of the influence of age, sex, education, income, marital status, ADL, IADL, chronic diseases, depressive symptoms, elevated depressive symptoms, quality of life, and cognitive functioning at baseline. A possible explanation for this is that senior center participants are more involved in cognitively and emotionally stimulating activities than non-participants. In many countries, the senior citizen center is an organization that provides various services, including physical/mental health, social and educational services, and recreational activities for older people [26, 41]. Studies has indicated that intellectually challenging activities and active interpersonal exchanges can increase or maintain cognitive reserve, allowing individuals to cope longer before cognitive impairment is manifested [42], and produce beneficial effects on cognition even in old age [13]. Studies of cognitive reserve suggest that there are individual differences in the ability to cope with the brain pathologic changes [43, 44] and cognitive reserve may be improved by exposure to an enriched environment such as social interaction or mentally challenging activity, physical activity, education [45], and may buffer against age-related CD [46]. Biological mechanisms related to the vascular hypothesis could also partially explain our findings. Reportedly, an active social activity might enhance cardiopulmonary fitness and cerebral oxygenation, which could protect against neuropathology in older people [47]. Other research on senior center participation and health has shown that senior center participants had higher levels of social interaction and better mental health than non-participants [48]. In South Korea, older people participating in senior citizen clubs can spend their leisure time with other people playing hwatu (Korean card game) or chess, singing songs, or dancing [49]. The senior centers provide diverse group activities for the elderly including painting, calligraphy, origami, gardening, playing musical instruments, acting, and exercise programs [41, 49]. Our result suggests that participation in senior citizen clubs may protect against CD in elderly people.

The interesting finding in our study was that frequent face-to-face contact with one’s children was positively associated with CD 4 years later in O-O adults but not in Y-O adults after controlling for potential confounders. According to the social exchange theory, excessive giving or receiving might be harmful for mental and physical health [50]. Excessive giving may result in exhaustion of resources of giver. On the contrary, excessive receiving can be distressing to the recipient because it may results in loss of independence [51]. Both situations might be harmful for mental and physical health among older adults [52]. In Korea, participation of women in the labor market has grown speedily as a result of industrialization. High female employment rate has resulted in increased need of assistance for household affairs such as taking care of young children in the family [19]. In Asian cultures where family interdependence is culturally valued, when adult children request, most of older adults meet their adult children and provide instrumental support [19, 53]. The burdens of helping their adult children might generate chronic unremitting stress, which may result in neuronal degeneration and have a negative impact on cognitive function in older adults [44, 54]. Several existing studies in Asian countries have reported that the care-giving burdens for their adult children might lead to stress and depressive symptoms among older adults [19, 53]. A study among older Nepalese adults found that close family relationship was related with greater stress and likely to have a negative impact on mental well-being in older women [23].

Receiving support that cannot be returned, meanwhile, can be distressing to the recipient [50]. With advancing years, older adults exchange support unequally with their children. The inability to reciprocate support can cause negative effects and stress in older people. Evidence has indicated that this stress can negatively impact cognitive functions [55]. An elderly person’s dependency generally increases with age. Age and poor health can restrict the physical functioning of older people and therefore increase the possibility of receiving aid from their adult children [51]. Empirical studies have found that O-O adults tend to have worse physical health and everyday functioning than Y-O adults [56]. While Y-O adults generally reciprocate their children’s support fairly equally, O-O adults are less likely to maintain reciprocal exchanges and become dependent on their adult children [22, 56]. O-O adults might experience this situation as a hassle or burden, which might impair cognitive functions. Studies have indicated that greater dependence can cause a devaluing of self and a lowering of morale [51, 57]. Our findings showing that face-to-face contact with one’s children had a positive association with CD 4 years later only in O-O adults can be interpreted as mentioned above. Our results were in agreement with a study on social support and cognitive function among American elderly that showed that lack of reciprocity in the parent-children relationship can have deleterious effects on cognitive function in older adults [58]. In Korea, there are strong instrumental interdependences between children and older parents because of insufficient social welfare programs (e.g., elder care support and child-caring) [59]. Thus, plenty of social welfare programs for older parents and adult children should be developed to reduce excessive support exchange of parent-children.

Conversely, another study reported that co-residence with children or close interaction with children was positively associated with emotional well-being and self-rated health in the Japanese elderly [60]. A study in Europe also showed that few contacts with children were associated with an increased number of depressive symptoms in older people [61]. Because impacts of contacts with children on older adults’ mental health are inconsistent in the studies, more research is needed to identify plausible relationships between them.

Frequent contact with children by phone or letters was associated with reduced risk of CD 4 years later in O-O adults. As a result of Westernized lifestyle and urbanization in recent decades, many Koreans no longer live with their parents. The proportion of empty nest families and elderly living alone is steadily increasing in Korea [62]. Talking with their adult children on the phone is likely to provide older adults emotional support and intimacy. This finding showing the beneficial effect of contact with children by phone or letters on CD is in agreement with previous study results reporting that frequent contact with children by phone or letters was significantly protective against depression among older people [12]. Studies have indicated that perceived emotional support protects against CD and operates as an antidote to stress, thus delaying neurodegenerative processes, whereas loneliness or isolation can worsen CD in older adults [20, 55]. Other studies have also shown that talking on the phone with children can provide older people with emotional support and be an important social activity in late life, confirming the concept that intimacy is needed for psychological well-being [12, 25].

In this study, older adults tended to more frequently participate in informal rather than formal social activities. Empirical evidence showed that Asians are likely to have a family-oriented culture, and older Asians tend to participate in fewer formal social activities compared to older Western people [12]. Our result also showed that there was a significant association between participating in two or more formal social activities and lower CD for O-O adults. This finding is line with a prior longitudinal study that greater social group participation can prevent CD [54]. Other studies among older adults also have reported that participation in a number of different organization reduce the onset of long-term care [63] and participation in a variety of social groups is effective for prevention of CD [64]. Our result indicates that encouraging older adults to participate in various formal social activities may help preserve the cognitive function in community-dwelling elderly population.

In our study, a higher level of quality of life showed a lower risk of CD. Quality of life has been associated with decreased risk of depressive symptoms and cognitive impairment in later life [65]. Our finding is consistent with a prior study reporting that quality of life was inversely associated with CD [66].

Both elevated depressive symptom over time and high levels of depressive symptom at baseline were positively associated with CD. Depressive symptoms have been associated with increased risk of cognitive impairment in later life [17, 38]. Consistent with our results, prior longitudinal studies have reported that cognitive function significantly declined over time in elderly women with elevated depressive symptom [6] and depressive symptoms at baseline predicted CD independently of age, gender, duration of follow up and baseline cognitive status [39].

Generally, formal and informal social activities have been considered to be more relevant for psychosocial well-being such as depressive symptom than for cognition. Ample evidence has shown the associations between social activities and depressive symptoms [12, 23]. Recently, however, many works have reported the significant relationship between social activities and CD [27, 54, 64]. Although age has been believed to be the strongest predictor of cognitive decline, our study showed that social activities may reduce CD in later life independently of the influence of age.

The possibility that social activities can mediate the relationship between depressive symptoms and CD might be raised because of associations between depressive symptoms and social activities. However, in our analyses, the social activities did not show mediating effect between depressive symptom and CD. Thus, it is reasonable to assume that social activities have direct relationship with CD in this sample.

This study had several strengths. First, using national longitudinal data, we examined a causal effect of social activity on delaying or preventing CD in late life. Second, we adjusted for various potential confounders of sociodemographics, health-related variables, mental well-being variables, and cognitive functioning at baseline. Third, subjects with cognitive impairment at baseline were excluded because impaired cognition can reduce social activity.

The present study had several limitations. First, although our analyses were restricted to study participants who had an MMSE score ≥ 24 at baseline, and adjusted for a wide variety of potential confounders, there still may be a possible reverse causation between cognitive function and social activity. Second, our findings are based a global measure of cognitive function. Thus, we cannot know which domains of cognitive function are specifically affected by social activities. Other research has measured specific cognitive domains such as episodic memory, semantic memory, perceptual speed, and visuospatial ability [67]. Further research using fuller batteries of cognitive function will be able to address the relationships between specific domains of cognitive function and social activity. Third, social activity measures relied on self-reports, which are subject to errors of recall [15]. Fourth, empirical evidence has indicated that quality rather than quantity of social interactions is more important to predict health outcomes [68]. However, in this study, social activity was measured only based on frequency. Our scale did not involve a potentially important dimension of social activity such as specific content, period of time, satisfaction, troublesome aspects, or burden. Future research should use detailed instruments including quantity and quality of social activity in order to obtain more accurate information on how social activities affect CD in late life. Finally, social activities and covariates were only measured at baseline. Previous studies have indicated that changes in social activities may affect CD in old age [64] and changes in other covariates such as health condition can influence the risk of CD [69]. Thus, future research needs to take into account the change over time in social activities and covariates.

Despite these limitations, this study supports the notion that more participation in social activity may help to prevent or postpone CD in old age and provides important information on cognitive function, offering convincing evidence for the types of social activity that reduce CD over time using a large, nationally representative sample of the Korean elderly.

## Conclusion

This community-based prospective study showed that participation in senior citizen clubs or senior centers and frequent contact with adult children by phone or letters may produce beneficial effects in reducing CD in later life among older adults. Participation in a variety of formal social activities may also have a positive effect on preventing CD in O-O adults. Our findings suggest that encouraging older adults to participate in intellectually challenging activities and active interpersonal exchanges may help preserve the cognitive function in community-dwelling elderly population.

## Additional files

Additional file 1: Table S1. Baseline characteristics of the sample based on age group. **Table S2.** Cognitive decline 4 years later among study population. **Table S3.** Percentage of cognitive decline 4 years later among study population. **Table S4.** Participation in formal social activities according to subject’s characteristics at baseline. **Table S5.** Correlations of the study variables for total sample. **Table S5**–**1.** Correlations of the study variables for Y-O adults. **Table S5**–**2.** Correlations of the study variables for O-O adults. **Table S6.** Variance inflation factors (VIF) of the study variables for multicollinearity. **Table S7.** Multivariate linear regression analysis of the associations between social activities and cognitive decline 4 years later. (DOCX 56 kb)
Additional file 2: KLoSA_2006_Wave1_English Questionnaire. (PDF 1994 kb)
Additional file 3: 2wave KLoSA Questionnaire English version_2008. (PDF 1412 kb)
Additional file 4: 2 wave KLoSA Codebook English version_2008. (XLSX 968 kb)
Additional file 5: 4wave KLoSA Questionnaire English versioin_2012. (PDF 1433 kb)
Additional file 6: 4wave KLoSA CodeBook English version_2012. (XLSX 944 kb)

## Abbreviations

ADL: Activity of daily living
CES-D: Center for epidemiological studies depression
IADL: Instrumental activity of daily living
KLoSA: Korean longitudinal study of aging
KRW: Korean won
MMSE: Mini-mental state examination
O-O: Old-old
SE: standard errors
Y-O: Young-old
