# Supplementary material for: Which type of social activities may reduce cognitive decline in the elderly?: a longitudinal population-based study
Source: BMC Geriatr. 2016 Sep 27;16:165. doi: 10.1186/s12877-016-0343-x (PMC5039914; doi:10.1186/s12877-016-0343-x)
Supplement: Additional file 1: Table S1. — Baseline characteristics of the sample based on age group. Table S2. Cognitive decline 4 years later among study population. Table S3. Percentage of cognitive decline 4 years later among study population. Table S4. Participation in formal social activities according to subject’s characteristics at baseline. Table S5. Correlations of the study variables for total sample. Table S5–1. Correlations of the study variables for Y-O adults. Table S5–2. Correlations of the study variables for O-O adults. Table S6. Variance inflation factors (VIF) of the study variables for multicollinearity. Table S7. Multivariate linear regression analysis of the associations between social activities and cognitive decline 4 years later. (DOCX 56 kb) [file 12877_2016_343_MOESM1_ESM.docx]

**Supplementary Table 1** Baseline characteristics of the sample based on age group

| Variable | | Total  (n = 1568) | Young-old  (65–74 years)  (n=1220) | Old-old  (≥75 years)  (n=348) | *t or X^2^* |
| --- | --- | --- | --- | --- | --- |
|  | | Mean ± SD or n (%) | Mean ± SD or n (%) | Mean ± SD or n (%) |  |
| **Socio-demographics** | |  |  |  |  |
| Age (years) | | 71.06 ± 0.12 | 68.97 ± 0.08 | 78.41 ± 0.16 | -54.892^***^ |
| Sex | |  |  |  | 1.461 |
| Male | | 852 (54.3) | 653(53.5) | 199(57.2) |  |
| Female | | 716 (45.7) | 567(46.5) | 149(42.8) |  |
| Marital status | |  |  |  | 13.381^***^ |
| Widowed | | 347(22.1) | 245(20.1) | 102(29.3) |  |
| Married | | 1221(77.9) | 975(79.9) | 246(70.1) |  |
| Education | |  |  |  | 8.240^*^ |
| ≤Elementary school | | 882(56.3) | 665(54.5) | 217(62.4) |  |
| Middle school | | 238(15.2) | 193(15.8) | 45(12.9) |  |
| High school | | 302(19.3) | 249(20.4) | 53(15.2) |  |
| College or above | | 146(9.3) | 113(9.3) | 33(9.5) |  |
| Household income | |  |  |  | 10.887^*^ |
| 1 quartile (lowest) | | 390(24.9) | 283(23.2) | 107(30.7) |  |
| 2 quartile | | 395(25.2) | 307(25.2) | 88(25.3) |  |
| 3 quartile | | 420(26.8) | 345(28.3) | 75(21.6) |  |
| 4 quartile | | 363(23.1) | 285(23.3) | 78(22.4) |  |
| Living arrangement | |  |  |  | 1.427 |
| With someone | | 1372(87.5) | 1074(88.0) | 298(85.6) |  |
| Alone | | 196(12.5) | 146(12.0) | 50(14.4) |  |
| Residential area | |  |  |  |  |
| Rural | | 462(29.5) | 350(28.7) | 112(32.2) | 1.592 |
| Urban | | 1106(70.5) | 870(71.3) | 236(67.8) |  |
| **Health-related variables** | |  |  |  |  |
| Comorbidities | |  |  |  |  |
| Hypertension | |  |  |  | 10.005^**^ |
| Yes | | 659(42.0) | 487(39.9) | 172(49.4) |  |
| No | | 909(58.0) | 733(60.1) | 176(50.6) |  |
| Diabetes mellitus | |  |  |  | 2.454 |
| Yes | | 263(16.8) | 195(16.0) | 68(19.5) |  |
| No | | 1305(83.2) | 1025(84.0) | 280(80.5) |  |
| Cancer | |  |  |  | 1.449 |
| Yes | | 40(2.6) | 28(2.3) | 12(3.5) |  |
| No | 1528(97.4) | | 1192(97.7) | 336(96.5) |  |
| Heart disease | |  |  |  | 2.325 |
| Yes | | 135(8.6) | 98(8.0) | 37(10.6) |  |
| No | | 1433(91.4) | 1122(92.0) | 311(89.4) |  |
| Stroke | |  |  |  | 0.301 |
| Yes | | 62(4.0) | 50(4.1) | 12(3.5) |  |
| No | | 1506(96.0) | 1170(95.9) | 336(96.5) |  |
| Hearing problems | |  |  |  | 11.804^***^ |
| Yes | | 66(4.2) | 40(3.3) | 26(7.5) |  |
| No | | 1502(95.8) | 1180(96.7) | 322(92.5) |  |
| ADL | |  |  |  | 1.197 |
| Independent | | 1546(98.6) | 1205(98.8) | 341(98.0) |  |
| Dependent | | 22(1.40) | 15(1.2) | 7(2.0) |  |
| IADL | |  |  |  | 10.796^***^ |
| Independent | | 1415(90.2) | 1117(91.6) | 298(85.6) |  |
| Dependent | | 153(9.8) | 103(8.4) | 50(14.4) |  |
| **Mental well-being** | |  |  |  |  |
| Depressive symptom at baseline | | 6.90 ± 5.07 | 6.81 ± 4.99 | 7.22 ± 5.37 | -1.321 |
| Ratio of change in depressive symptom 4 years later | | .37±1.63 | 0.34±1.62 | 0.47±1.68 | -1.21 |
| Quality of life | | 62.01±17.57 | 61.89±17.44 | 62.44±18.05 | -0.522 |
| **Social activities** | |  |  |  |  |
| Formal social activities | |  |  |  |  |
| Level of participation in church or other religious groups | | 1.88±3.36 | 1.84±3.33 | 2.00±3.45 | -0.770 |
| Level of participation in senior citizen clubs or senior centers | | 4.03±3.58 | 4.06±3.47 | 3.92±3.93 | 0.638 |
| Level of participation in alumni societies or family councils | | 0.67±1.79 | 0.70±1.81 | 0.57±1.72 | 1.163 |
| Informal social activities | |  |  |  |  |
| Level of face-to-face contact with close friends | | 8.03 ± 2.26 | 8.05±2.19 | 7.97±2.47 | 0.628 |
| Level of face-to-face contact with one’s children | | 5.09±1.52 | 5.17±1.56 | 4.80±1.33 | 4.083^***^ |
| Level of contact with one’s children by phone or letter | | 7.17±1.49 | 7.24±1.51 | 6.92±1.41 | 3.447^***^ |
| **Cognitive function** | |  |  |  |  |
| MMSE score (mean, SD) in 2008 | | 26.64 ± 1.69 | 26.73 ± 1.69 | 26.32 ± 1.68 | 4.085^***^ |
| MMSE score (mean, SD) in 2012 | | 23.92 ± 6.63 | 24.55 ± 6.06 | 21.73 ± 7.97 | 7.104^***^ |
| Decline in cognitive function 4 years later | | 2.72 ± 6.52 | 2.18 ± 5.96 | 4.59 ± 7.90 | -6.136^***^ |

Note: SD = standard deviation; ADL = activities of daily living; IADL = instrumental activities of daily living; MMSE = Mini-Mental State Examination, * p < 0.05, ** p < 0.01, *** p < 0.001

**Supplementary Table 2** Cognitive decline 4 years later among study population

|  | Total  (n = 1568) | Young-old  (n=1220) | Old-old  (n=348) | *t* |
| --- | --- | --- | --- | --- |
|  | Mean ± SD | Mean ± SD | Mean ± SD |  |
| Global cognition (MMSE score) | 2.72 ± 6.52 | 2.18 ± 5.96 | 4.59 ± 7.90 | -6.136^***^ |
| Orientation for time (5 points) | 0.39 ± 1.27 | 0.31 ± 1.17 | 0.67 ± 1.56 | -4.623^***^ |
| Orientation for place (5 points) | 0.39 ± 1.23 | 0.32 ± 1.13 | 0.61 ± 1.50 | -3.900^***^ |
| Registration (3 points) | 0.34 ± 0.93 | 0.27 ± 0.87 | 0.59 ± 1.08 | -5.776^***^ |
| Attention & calculation (5 points) | 0.71 ± 1.79 | 0.60 ± 1.70 | 1.13 ± 2.00 | -4.913^***^ |
| Recall (3 points) | 0.21 ± 1.15 | 0.16 ± 1.12 | 0.37 ± 1.24 | -3.076^**^ |
| Language (8 points) | 0.60 ± 2.02 | 0.46 ± 1.87 | 1.11 ± 2.40 | -5.373^***^ |
| Visual construction (1 points) | 0.13 ± 0.51 | 0.10 ± 0.50 | 0.23 ±0.51 | -4.376^***^ |

All positive values indicate a decline in the MMSE score. * p < 0.05, ** p < 0.01, *** p < 0.001

**Supplementary Table 3** Percentage of cognitive decline 4 years later among study population

|  | Total  (n = 1568) | Young-old  (n=1220) | Old-old  (n=348) | *X^2^* |
| --- | --- | --- | --- | --- |
|  | n (%) | n (%) | n (%) |  |
| Global cognition (MMSE score) | 867 (55.3) | 634 (52.0) | 233 (67.0) | 24.602^***^ |
| Orientation for time | 305 (19.5) | 201 (16.5) | 104 (29.9) | 31.075^***^ |
| Orientation for place | 287 (18.3) | 197 (16.2) | 90 (25.9) | 17.088^***^ |
| Registration | 398 (25.4) | 266 (21.8) | 132 (37.9) | 37.185^***^ |
| Attention & calculation | 621 (39.6) | 451 (37.0) | 170 (48.9) | 15.985^***^ |
| Recall | 566 (36.1) | 412 (33.8) | 154 (44.25) | 12.898^***^ |
| Language | 596 (38.0) | 430 (35.3) | 166 (47.7) | 17.827^***^ |
| Visual construction | 314 (20.0) | 219 (18.0) | 95 (27.3) | 14.774^***^ |

* p < 0.05, ** p < 0.01, *** p < 0.001

**Supplementary Table 4** Participation in formal social activities according to subject’s characteristics at baseline

| Variable | Participation in church or other religious groups | | *t or X^2^* | Participation in senior citizen clubs or senior centers | | *t or X^2^* | Participation in alumni societies or family councils | | *t or X^2^* |
| --- | --- | --- | --- | --- | --- | --- | --- | --- | --- |
|  | Mean ± SD or n (%) | |  | Mean ± SD or n (%) | |  | Mean ± SD or n (%) | |  |
|  | YES  (n=391) | No  (n=1177) |  | YES  (n=937) | No  (n=631) |  | YES  (n=215) | No  (n=1353) |  |
| Age (yr), mean±SD * | 71.13 ± 4.89 | 71.04 ± 4.82 | 0.090 | 70.75 ± 4.76 | 71.53 ± 4.91 | 10.020^**^ | 70.60 ± 4.49 | 71.14 ± 4.89 | 2.230 |
| Gender |  |  | 48.544^***^ |  |  | 10.853^**^ |  |  | 119.530^***^ |
| Female | 238 (60.9) | 478 (40.6) |  | 396 (42.3) | 320 (50.7) |  | 24 (11.2) | 692 (51.2) |  |
| Male | 153 (39.1) | 699 (59.4) |  | 541 (57.7) | 311 (49.3) |  | 191 (88.8) | 661 (48.8) |  |
| Marital status |  |  | 7.496^**^ |  |  | 7.021^**^ |  |  | 23.8^***^ |
| Bereaved | 106 (27.1) | 241 (20.5) |  | 186 (19.9) | 161 (25.5) |  | 20 (9.3) | 327 (24.2) |  |
| Married | 285 (72.9) | 936 (79.5) |  | 751 (80.1) | 470 (74.5) |  | 195 (90.7) | 1026(75.8) |  |
| Education |  |  | 1.105 |  |  | 5.752 |  |  | 133.701^***^ |
| ≤Elementary school | 212 (54.2) | 670 (56.9) |  | 507 (54.1) | 375 (59.4) |  | 52 (24.2) | 830 (61.4) |  |
| Middle school | 60 (15.4) | 178 (15.1) |  | 150 (16.0) | 88 (13.9) |  | 38 (17.7) | 200 (14.8) |  |
| High school | 79 (20.2) | 223 (19.0) |  | 183 (19.5) | 119 (18.9) |  | 73 (33.9) | 229 (16.9) |  |
| College or above | 40 (10.2) | 106 (9.0) |  | 97 (10.4) | 49 (7.8) |  | 52 (24.1) | 94 (6.9) |  |
| Household income |  |  | 2.859 |  |  | 14.465^**^ |  |  | 34.227^***^ |
| 1 quartile (lowest) | 103 (26.3) | 287 (24.4) |  | 213 (22.7) | 177 (28.1) |  | 42 (19.5) | 348 (25.7) |  |
| 2 quartile | 102 (26.1) | 293 (24.9) |  | 224 (23.9) | 171 (27.1) |  | 36 (16.7) | 359 (26.5) |  |
| 3 quartile | 92 (23.5) | 328 (27.8) |  | 256 (27.3) | 164 (26.0) |  | 55 (25.6) | 365 (27.0) |  |
| 4 quartile | 94 (24.1) | 269 (22.9) |  | 244 (26.1) | 119 (18.8) |  | 82 (38.1) | 281 (20.8) |  |
| ADL |  |  | 1.557 |  |  | 7.243^**^ |  |  | 4.536 |
| Independent | 383 (98.0) | 1163 (98.8) |  | 930 (99.3) | 616 (97.6) |  | 215 (100.0) | 1331 (98.4) |  |
| Dependent | 8 (2.0) | 14 (1.2) |  | 7 (0.7) | 15 (2.4) |  | 0 (0.0) | 22 (1.6) |  |
| IADL |  |  | 3.241 |  |  | 0.062 |  |  | 6.096^*^ |
| Independent | 1053 (89.5) | 362 (92.6) |  | 837 (90.4) | 568 (90.0) |  | 204 (94.9) | 1211 (89.5) |  |
| Dependent | 124 (10.5) | 29 (7.4) |  | 90 (9.6) | 63 (10.0) |  | 11(5.1) | 142 (10.5) |  |

* p < 0.05, ** p < 0.01, *** p < 0.001

**Supplementary Table 5** Correlations of the study variables for total sample

| Variables | 1 | 2 | 3 | 4 | 5 | 6 | 7 | 8 | 9 | 10 | 11 | 12 | 13 | 14 | 15 | 16 | 17 | 18 | 19 | 20 | 21 | 22 | 23 | 24 | 25 | 26 |
| --- | --- | --- | --- | --- | --- | --- | --- | --- | --- | --- | --- | --- | --- | --- | --- | --- | --- | --- | --- | --- | --- | --- | --- | --- | --- | --- |
| Cognitive decline | 1. |  |  |  |  |  |  |  |  |  |  |  |  |  |  |  |  |  |  |  |  |  |  |  |  |  |
| Age | 0.175^***^ | 1 |  |  |  |  |  |  |  |  |  |  |  |  |  |  |  |  |  |  |  |  |  |  |  |  |
| Sex | -0.020 | 0.075^**^ | 1 |  |  |  |  |  |  |  |  |  |  |  |  |  |  |  |  |  |  |  |  |  |  |  |
| Marital status | 0.043 | 0.111^***^ | -0.401^***^ | 1 |  |  |  |  |  |  |  |  |  |  |  |  |  |  |  |  |  |  |  |  |  |  |
| Education | -0.027 | -0.074^**^ | 0.359^***^ | -0.200^***^ | 1 |  |  |  |  |  |  |  |  |  |  |  |  |  |  |  |  |  |  |  |  |  |
| Household income | 0.010 | -0.102^***^ | 0.068^**^ | -0.075^**^ | 0.112^***^ | 1 |  |  |  |  |  |  |  |  |  |  |  |  |  |  |  |  |  |  |  |  |
| Living arrangement | 0.018 | 0.040 | -0.269^***^ | 0.687^***^ | -0.120^***^ | -0.184^***^ | 1 |  |  |  |  |  |  |  |  |  |  |  |  |  |  |  |  |  |  |  |
| Residential area | -0.013 | -0.008 | -0.006 | 0.061^*^ | 0.187^***^ | -0.003 | 0.012 | 1 |  |  |  |  |  |  |  |  |  |  |  |  |  |  |  |  |  |  |
| Hypertension | 0.046 | 0.093^***^ | -0.088^***^ | 0.054^*^ | 0.025 | -0.084^***^ | 0.073^**^ | 0.074^**^ | 1 |  |  |  |  |  |  |  |  |  |  |  |  |  |  |  |  |  |
| Diabetes mellitus | -0.019 | 0.013 | -0.055^*^ | 0.068^**^ | -0.059^*^ | -0.026 | 0.068^**^ | 0.032 | 0.206^***^ | 1 |  |  |  |  |  |  |  |  |  |  |  |  |  |  |  |  |
| Cancer | 0.028 | 0.024 | 0.018 | -0.017 | 0.004 | -0.066^**^ | -0.012 | 0.007 | -0.056^*^ | -0.019 | 1 |  |  |  |  |  |  |  |  |  |  |  |  |  |  |  |
| Heart disease | 0.004 | 0.037 | -0.043 | 0.037 | -0.000 | -0.029 | 0.008 | 0.034 | 0.107^***^ | 0.075^**^ | -0.035 | 1 |  |  |  |  |  |  |  |  |  |  |  |  |  |  |
| Stroke | 0.052^*^ | 0.003 | 0.055^*^ | -0.015 | -0.021 | -0.020 | -0.027 | -0.005 | 0.119^***^ | 0.040 | -0.012 | 0.019 | 1 |  |  |  |  |  |  |  |  |  |  |  |  |  |
| Hearing problems | 0.040 | 0.128^***^ | 0.084^***^ | 0.013 | -0.044 | -0.041 | 0.007 | -0.011 | -0.018 | 0.042 | 0.006 | 0.038 | 0.039 | 1 |  |  |  |  |  |  |  |  |  |  |  |  |
| ADL | 0.088^***^ | 0.033 | 0.011 | 0.038 | 0.026 | -0.007 | -0.012 | 0.018 | 0.030 | 0.005 | 0.015 | 0.060^*^ | 0.115^***^ | 0.002 | 1 |  |  |  |  |  |  |  |  |  |  |  |
| IADL | 0.029 | 0.109^***^ | 0.103^***^ | -0.052^*^ | 0.026 | -0.054^*^ | -0.066^**^ | 0.038 | 0.016 | 0.031 | -0.012 | 0.045 | 0.088^***^ | 0.027 | 0.290^***^ | 1 |  |  |  |  |  |  |  |  |  |  |
| Depressive symptom at baseline | 0.093^***^ | 0.058^*^ | -0.184^***^ | 0.137^***^ | -0.113^***^ | -0.112^***^ | 0.095^***^ | -0.025 | 0.042 | 0.078^**^ | 0.086^***^ | 0.030 | 0.034 | 0.031 | 0.154^***^ | 0.127^***^ | 1 |  |  |  |  |  |  |  |  |  |
| Ratio of change in depressive symptom 4 years later | 0.100^***^ | 0.055^*^ | 0.050 | -0.036 | 0.053 | 0.024 | -0.021 | -0.011 | 0.041 | -0.006 | -0.042 | -0.008 | 0.072 | -0.041 | -0.030 | -0.052^*^ | -0.388^***^ | 1.00 |  |  |  |  |  |  |  |  |
| Quality of life | -0.080^**^ | -0.017 | 0.074^**^ | -0.116^***^ | 0.190^***^ | 0.125^***^ | -0.054^*^ | -0.034 | -0.033 | -0.071^**^ | -0.042 | -0.021 | -0.021 | -0.055^*^ | -0.079^**^ | -0.052^*^ | -0.313^***^ | 0.059^*^ | 1 |  |  |  |  |  |  |  |
| Baseline MMSE | 0.059^*^ | -0.142^***^ | 0.104^***^ | -0.025 | 0.190^***^ | 0.093^***^ | -0.023 | 0.119^***^ | -0.021 | -0.044 | -0.047 | -0.029 | -0.027 | -0.070^**^ | -0.064^*^ | -0.043 | -0.161^***^ | 0.009 | 0.093^***^ | 1 |  |  |  |  |  |  |
| Participation in church or other religious groups | 0.009 | 0.008 | -0.176^***^ | 0.172^**^ | 0.024 | -0.020 | 0.063^*^ | 0.124^***^ | 0.074^**^ | 0.045 | -0.037 | 0.049 | 0.050 | -0.011 | 0.032 | -0.046 | 0.023 | 0.012 | 0.047 | -0.005 | 1 |  |  |  |  |  |
| Participation in senior citizen clubs or senior centers | -0.097^***^ | -0.080^**^ | 0.083^**^ | -0.066^**^ | 0.053^*^ | 0.060^*^ | -0.008 | -0.117^***^ | -0.034 | -0.032 | -0.032 | -0.026 | -0.020 | -0.087^***^ | -0.068^**^ | -0.006 | -0.036 | 0.071^**^ | 0.112^***^ | 0.049 | -0.200^***^ | 1 |  |  |  |  |
| Participation in alumni societies or family councils | -0.015 | -0.038 | 0.276^***^ | -0.123^***^ | 0.258^***^ | 0.049 | -0.089^***^ | 0.038 | -0.039 | -0.065^*^ | -0.006 | 0.043 | -0.024 | -0.047 | -0.048 | -0.062^*^ | -0.159^***^ | 0.037 | 0.171^***^ | 0.095^***^ | -0.003 | 0.085^***^ | 1 |  |  |  |
| Level of face-to-face contact with close friends | -0.052^*^ | -0.028 | -0.040 | 0.040 | -0.040 | -0.051^*^ | 0.095^***^ | -0.087^***^ | -0.032 | 0.020 | -0.017 | 0.042 | -0.064^*^ | 0.014 | -0.122^***^ | -0.042 | -0.146^***^ | -0.006 | 0.118^***^ | 0.023 | 0.095^***^ | 0.105^***^ | 0.056^*^ | 1 |  |  |
| Level of face-to-face contact with one’s children | -0.020 | -0.121^***^ | 0.067^**^ | -0.030 | 0.127^***^ | 0.036 | -0.008 | 0.127^***^ | -0.037 | -0.031 | -0.014 | 0.033 | -0.014 | -0.067^**^ | -0.042 | -0.060^*^ | -0.111^***^ | -0.034 | 0.123^***^ | 0.072^**^ | 0.025 | 0.002 | 0.067^**^ | 0.040 | 1 |  |
| Level of contact with one’s children by phone or letter | -0.073^**^ | -0.108^***^ | 0.002 | -0.018 | 0.097^***^ | 0.089^***^ | -0.016 | 0.028 | -0.057^*^ | -0.036 | -0.035 | -0.002 | -0.026 | -0.105^***^ | -0.063^*^ | -0.067^**^ | -0.133^***^ | -0.012 | 0.196^***^ | 0.071^**^ | 0.046 | 0.048 | 0.081^**^ | 0.067^**^ | 0.615^***^ | 1 |

* p < 0.05, ** p < 0.01, *** p < 0.001

**Supplementary Table 5-1** Correlations of the study variables for Y-O adults

| Variables | 1 | 2 | 3 | 4 | 5 | 6 | 7 | 8 | 9 | 10 | 11 | 12 | 13 | 14 | 15 | 16 | 17 | 18 | 19 | 20 | 21 | 22 | 23 | 24 | 25 | 26 |
| --- | --- | --- | --- | --- | --- | --- | --- | --- | --- | --- | --- | --- | --- | --- | --- | --- | --- | --- | --- | --- | --- | --- | --- | --- | --- | --- |
| Cognitive decline | 1. |  |  |  |  |  |  |  |  |  |  |  |  |  |  |  |  |  |  |  |  |  |  |  |  |  |
| Age | 0.10^8***^ | 1 |  |  |  |  |  |  |  |  |  |  |  |  |  |  |  |  |  |  |  |  |  |  |  |  |
| Sex | -0.040 | 0.083^**^ | 1 |  |  |  |  |  |  |  |  |  |  |  |  |  |  |  |  |  |  |  |  |  |  |  |
| Marital status | 0.040 | 0.056 | -0.356^***^ | 1 |  |  |  |  |  |  |  |  |  |  |  |  |  |  |  |  |  |  |  |  |  |  |
| Education | -.018 | -0.026 | 0.369^***^ | -0.203^***^ | 1 |  |  |  |  |  |  |  |  |  |  |  |  |  |  |  |  |  |  |  |  |  |
| Household income | -0.010 | -0.083^**^ | 0.084^**^ | -0.081^**^ | 0.104^***^ | 1 |  |  |  |  |  |  |  |  |  |  |  |  |  |  |  |  |  |  |  |  |
| Living arrangement | 0.020 | 0.050 | -0.254^***^ | 0.709^***^ | -0.129^***^ | -0.156^***^ | 1 |  |  |  |  |  |  |  |  |  |  |  |  |  |  |  |  |  |  |  |
| Residential area | -0.007 | 0.031 | -0.010 | 0.068^*^ | 0.194^***^ | -0.027 | 0.005 | 1 |  |  |  |  |  |  |  |  |  |  |  |  |  |  |  |  |  |  |
| Hypertension | 0.039 | 0.067^**^ | -0.086^**^ | 0.019 | 0.018 | -0.076^**^ | -0.050 | 0.070^*^ | 1 |  |  |  |  |  |  |  |  |  |  |  |  |  |  |  |  |  |
| Diabetes mellitus | -0.001 | -0.005 | -0.060^*^ | 0.072^*^ | -0.066^*^ | -0.036 | 0.053 | 0.044 | 0.197^***^ | 1 |  |  |  |  |  |  |  |  |  |  |  |  |  |  |  |  |
| Cancer | -0.021 | -0.010 | 0.000 | -0.007 | 0.014 | -0.071^*^ | -0.023 | 0.013 | -0.047 | -0.022 | 1 |  |  |  |  |  |  |  |  |  |  |  |  |  |  |  |
| Heart disease | 0.002 | 0.012 | -0.051 | 0.021 | -0.004 | -0.031 | -0.007 | 0.067* | 0.110^***^ | 0.085^**^ | -0.025 | 1 |  |  |  |  |  |  |  |  |  |  |  |  |  |  |
| Stroke | 0.049 | 0.047 | 0.068^*^ | -0.002 | -0.023 | -0.014 | -0.025 | 0.003 | 0.144^***^ | 0.034 | -0.032 | 0.015 | 1 |  |  |  |  |  |  |  |  |  |  |  |  |  |
| Hearing problems | 0.011 | 0.110^***^ | 0.061^**^ | 0.048 | -0.039 | -0.041 | 0.017 | 0.015 | 0.000 | 0.007 | 0.033 | 0.013 | 0.032 | 1 |  |  |  |  |  |  |  |  |  |  |  |  |
| ADL | 0.122^***^ | 0.031 | -0.000 | 0.051 | 0.032 | -0.009 | 0.005 | 0.024 | 0.015 | 0.033 | -0.017 | 0.076^**^ | 0.165^***^ | 0.021 | 1 |  |  |  |  |  |  |  |  |  |  |  |
| IADL | 0.021 | 0.097^***^ | 0.123^***^ | -0.074^**^ | 0.072^*^ | -0.043 | -0.058^*^ | 0.043 | -0.000 | 0.029 | -0.027 | 0.062^*^ | 0.086^**^ | -0.006 | 0.260^***^ | 1 |  |  |  |  |  |  |  |  |  |  |
| Depressive symptom at baseline | 0.073^*^ | 0.062^*^ | -0.205^***^ | 0.140^***^ | -0.128^***^ | -0.138^***^ | 0.117^**^* | -0.032 | 0.036 | 0.085** | 0.080** | 0.041 | 0.032 | 0.036 | 0.131^***^ | 0.084^**^ | 1 |  |  |  |  |  |  |  |  |  |
| Ratio of change in depressive symptom 4 years later | 0.088^**^ | 0.049 | 0.030 | -0.032 | 0.056 | 0.044 | -0.035 | 0.022 | 0.048 | -0.020 | -0.047 | 0.017 | 0.068 | -0.041 | -0.029 | -0.053 | -0.387^***^ | 1.00 |  |  |  |  |  |  |  |  |
| Quality of life | -0.106^***^ | -0.074 | 0.066^*^ | -0.114^***^ | 0.186^***^ | 0.124^***^ | -0.053 | -0.034 | -0.004 | -0.086^**^ | -0.057^*^ | -0.046 | -0.046 | -0.086^**^ | -0.046 | -0.043 | -0.320^***^ | 0.042 | 1 |  |  |  |  |  |  |  |
| Baseline MMSE | 0.082^**^ | -0.102^***^ | 0.100^***^ | -0.027 | 0.191^***^ | 0.083^**^ | -0.041 | 0.103^***^ | -0.002 | -0.060^*^ | -0.060^*^ | -0.025 | -0.039 | -0.031 | -0.102^***^ | -0.040 | -0.150^***^ | -0.011 | 0.118^***^ | 1 |  |  |  |  |  |  |
| Participation in church or other religious groups | 0.034 | -0.004 | -0.180^***^ | 0.062^*^ | 0.012 | -0.023 | 0.047 | 0.123^***^ | -0.073^*^ | 0.062^*^ | -0.037^*^ | 0.069^*^ | 0.083^**^ | -0.009 | 0.040 | -0.030 | 0.027 | 0.014 | 0.021 | 0.009 | 1 |  |  |  |  |  |
| Participation in senior citizen clubs or senior centers | -0.076^*^ | ^*^-0.064^*^ | 0.083^**^ | -0.082^**^ | 0.054 | 0.076^**^ | -0.014 | -0.107^***^ | -0.025 | -0.050 | -0.070^*^ | -0.057^*^ | -0.006 | -0.062^*^ | -0.065^*^ | -0.014 | -0.048 | 0.023 | 0.127^***^ | 0.061^*^ | -0.199^***^ | 1 |  |  |  |  |
| Participation in alumni societies or family councils | -0.003 | -0.004 | 0.292^***^ | -0.111^***^ | 0.260^***^ | 0.053 | -0.086^**^ | 0.037 | -0.018 | -0.057^*^ | 0.015 | 0.034 | -0.014 | -0.049 | -0.046 | -0.040 | -0.169^***^ | 0.061^*^ | 0.172^***^ | 0.089^**^ | 0.010 | 0.079^**^ | 1 |  |  |  |
| Level of face-to-face contact with close friends | -0.017 | -0.025 | -0.038 | 0.043 | -0.040 | -0.031 | 0.077^**^ | -0.113^***^ | -0.009 | 0.011 | -0.021 | 0.034 | -0.045 | 0.008 | -0.064^*^ | -0.001 | -0.116^***^ | 0.022 | 0.103^***^ | 0.037 | 0.087^**^ | 0.073^*^ | 0.050 | 1 |  |  |
| Level of face-to-face contact with one’s children | -0.038 | -0.085^**^ | 0.058^*^ | -0.017 | 0.111^***^ | 0.044 | -0.021 | 0.127^***^ | -0.051 | -0.038 | 0.002 | 0.054^*^ | -0.003 | -0.054 | -0.011 | -0.031 | -0.112^***^ | 0.000 | 0.118^***^ | 0.075^**^ | 0.045 | -0.005 | 0.059^*^ | 0.034 | 1 |  |
| Level of contact with one’s children by phone or letter | -0.063^*^ | -0.063^*^ | -0.009 | -0.012 | 0.084^**^ | 0.100^***^ | -0.030 | 0.021 | -0.062* | -0.040 | -0.038 | 0.017 | -0.013 | -0.103^***^ | -0.040 | -0.054 | -0.127^***^ | -0.012 | 0.192^***^ | 0.080^**^ | 0.068^*^ | 0.042 | 0.071^*^ | 0.072^*^ | 0.611^***^ | 1 |

* p < 0.05, ** p < 0.01, *** p < 0.001

**Supplementary Table 5-2** Correlations of the study variables for O-O adults

| Variables | 1 | 2 | 3 | 4 | 5 | 6 | 7 | 8 | 9 | 10 | 11 | 12 | 13 | 14 | 15 | 16 | 17 | 18 | 19 | 20 | 21 | 22 | 23 | 24 | 25 | 26 |
| --- | --- | --- | --- | --- | --- | --- | --- | --- | --- | --- | --- | --- | --- | --- | --- | --- | --- | --- | --- | --- | --- | --- | --- | --- | --- | --- |
| Cognitive decline | 1. |  |  |  |  |  |  |  |  |  |  |  |  |  |  |  |  |  |  |  |  |  |  |  |  |  |
| Age | 0.040 | 1 |  |  |  |  |  |  |  |  |  |  |  |  |  |  |  |  |  |  |  |  |  |  |  |  |
| Sex | 0.014 | 0.093 | 1 |  |  |  |  |  |  |  |  |  |  |  |  |  |  |  |  |  |  |  |  |  |  |  |
| Marital status | 0.002 | 0.061 | -0.564^***^ | 1 |  |  |  |  |  |  |  |  |  |  |  |  |  |  |  |  |  |  |  |  |  |  |
| Education | -0.014 | -0.069 | 0.337^***^ | -0.172^**^ | 1 |  |  |  |  |  |  |  |  |  |  |  |  |  |  |  |  |  |  |  |  |  |
| Household income | 0.097 | -0.048 | 0.028 | -0.035 | 0.119^*^ | 1 |  |  |  |  |  |  |  |  |  |  |  |  |  |  |  |  |  |  |  |  |
| Living arrangement | -0.002 | -0.042 | -0.324^***^ | 0.629^***^ | -0.082 | -0.260^***^ | 1 |  |  |  |  |  |  |  |  |  |  |  |  |  |  |  |  |  |  |  |
| Residential area | -0.010 | 0.026 | 0.013 | 0.054 | 0.154^**^ | 0.061 | 0.037 | 1 |  |  |  |  |  |  |  |  |  |  |  |  |  |  |  |  |  |  |
| Hypertension | 0.023 | -0.012 | -0.109^*^ | 0.130^*^ | 0.074 | -0.089 | 0.136^**^ | 0.103 | 1 |  |  |  |  |  |  |  |  |  |  |  |  |  |  |  |  |  |
| Diabetes mellitus | -0.065 | -0.011 | -0.042 | 0.046 | -0.024 | 0.014 | 0.108^*^ | -0.002 | 0.223^***^ |  |  |  |  |  |  |  |  |  |  |  |  |  |  |  |  |  |
| Cancer | 0.128^*^ | 0.021 | 0.068 | -0.052 | -0.017 | -0.045 | 0.012 | -0.005 | -0.092 | -0.014 | 1 |  |  |  |  |  |  |  |  |  |  |  |  |  |  |  |
| Heart disease | -0.017 | -0.013 | -0.022 | 0.066 | 0.021 | -0.013 | 0.045 | -0.062 | 0.088 | 0.042 | -0.065 | 1 |  |  |  |  |  |  |  |  |  |  |  |  |  |  |
| Stroke | 0.074 | -0.057 | 0.004 | -0.052 | -0.017 | -0.045 | -0.033 | -0.038 | 0.034 | 0.066 | 0.051 | 0.037 | 1 |  |  |  |  |  |  |  |  |  |  |  |  |  |
| Hearing problems | 0.058 | 0.077 | 0.135^*^ | -0.086 | -0.040 | -0.024 | -0.023 | -0.062 | -0.084 | -0.030 | -0.054 | 0.079 | 0.066 | 1 |  |  |  |  |  |  |  |  |  |  |  |  |
| ADL | 0.008 | -0.013 | 0.041 | -0.002 | 0.015 | 0.007 | -0.059 | 0.011 | 0.063 | -0.071 | 0.085 | 0.017 | -0.027 | -0.041 | 1 |  |  |  |  |  |  |  |  |  |  |  |
| IADL | 0.007 | 0.004 | 0.040 | -0.028 | -0.082 | -0.064 | -0.098 | 0.037 | 0.038 | 0.025 | 0.012 | -0.008 | 0.102 | 0.071 | 0.350^***^ | 1 |  |  |  |  |  |  |  |  |  |  |
| Depressive symptom at baseline | 0127^*^ | 0.021 | -0.120^*^ | 0.118^*^ | -0.057 | -0.028 | 0.026 | 0.002 | 0.050 | 0.053 | 0.101 | -0.007 | 0.045 | 0.013 | 0.212^***^ | 0.230^***^ | 1 |  |  |  |  |  |  |  |  |  |
| Ratio of change in depressive symptom 4 years later | 0.122^*^ | 0.048 | 0.115^*^ | -0.061 | 0.052 | -0.029 | 0.019 | -0.115^*^ | 0.008 | 0.032 | -0.033 | -0.022 | 0.087 | -0.052 | -0.038 | -0.063 | -0.399^***^ | 1.00 |  |  |  |  |  |  |  |  |
| Quality of life | -0.029 | 0.033 | 0.098 | -0.131^*^ | 0.211^***^ | 0.135^***^ | -0.060 | -0.023 | -0.003 | -0.027 | 0.000 | 0.052 | 0.071 | 0.010 | -0.167^**^ | -0.083 | -292^***^ | 0.113 | 1 |  |  |  |  |  |  |  |
| Baseline MMSE | 0.065 | -0.100 | 0.135^*^ | 0.023 | 0.161^**^ | 0.100 | 0.050 | 0.163^**^ | -0.049 | -0.067 | 0.002 | -0.027 | 0.011 | -0.138^**^ | 0.046 | -0.024 | -0.185^***^ | 0.092 | 0.017 | 1 |  |  |  |  |  |  |
| Participation in church or other religious groups | -0.061 | 0.001 | -0.165^**^ | 0.098 | 0.069 | -0.005 | 0.114^*^ | 0.126^*^ | 0.072 | -0.010 | -0.040 | -0.012 | -0.076 | -0.018 | 0.009 | -0.092 | 0.006 | -0.053 | 0.131 | -0.049 | 1 |  |  |  |  |  |
| Participation in senior citizen clubs or senior centers | -0.122^*^ | 0.011 | 0.094 | 0.007 | 0.031 | -0.006 | 0.019 | -0.158^**^ | -0.040 | 0.036 | 0.081 | 0.077 | -0.077 | -0.131^*^ | -0.072 | 0.035 | 0.013 | -0.015 | 0.065 | -0.021 | -0.202^***^ | 1 |  |  |  |  |
| Participation in alumni societies or family councils | -0.029 | -0.060 | 0.221^***^ | -0.152^**^ | 0.241^***^ | 0.025 | -0.096 | 0.036 | -0.104 | -0.087 | -0.068 | 0.080 | -0.068 | -0.034 | -0.052 | -0.122^*^ | -0.122^*^ | 0.114^*^ | 0.171^**^ | 0.104 | -0.048 | 0.100^***^ | 1 |  |  |  |
| Level of face-to-face contact with close friends | -0.127^*^ | -0.026 | -0.043 | 0.038 | -0.045 | -0.113^*^ | 0.152^**^ | -0.012 | -0.098 | 0.048 | -0.004 | 0.065 | -0.132^*^ | 0.031 | -0.264^***^ | -0.117^*^ | -0.234^***^ | 0.086 | 0.164^**^ | -0.028 | 0.120^*^ | 0.200^***^ | 0.075 | 1 |  |  |
| Level of face-to-face contact with one’s children | 0.102 | 0.007 | 0.123^*^ | -0.034 | 0.165^**^ | -0.031 | 0.058 | 0.116 | 0.056 | 0.013 | -0.056 | -0.032 | -0.070 | -0.076 | -0.135^*^ | -0.122^*^ | -0.098 | -0.015 | 0.157^**^ | 0.012 | -0.048 | -0.003 | 0.089 | 0.059 | 1 |  |
| Level of contact with one’s children by phone or letter | -0.053 | -0.072 | 0.056 | -0.004 | 0.118^*^ | 0.026 | 0.043 | 0.041 | -0.006 | -0.011 | -0.018 | -0.050 | -0.083 | -0.092 | -0.123^*^ | -0.081 | -0.154^**^ | -0.103 | 0.222^***^ | -0.002 | -0.032 | 0.042 | 0.109^*^ | 0.047 | 0.062 | 1 |

* p < 0.05, ** p < 0.01, *** p < 0.001

**Supplementary Table 6** Variance inflation factors (VIF) of the study variables for multicollinearity

| Variables | Total | Young-old | Old-old |
| --- | --- | --- | --- |
| Age | 1.14 | 1.08 | 1.13 |
| Sex | 1.54 | 1.51 | 1.87 |
| Marital status | 2.28 | 2.32 | 2.48 |
| Education | 1.33 | 1.34 | 1.35 |
| Household income | 1.11 | 1.10 | 1.23 |
| Living arrangement | 2.04 | 2.14 | 2.01 |
| Residential area | 1.13 | 1.14 | 1.18 |
| Hypertension | 1.11 | 1.11 | 1.18 |
| Diabetes mellitus | 1.06 | 1.07 | 1.12 |
| Cancer | 1.03 | 1.04 | 1.08 |
| Heart disease | 1.03 | 1.04 | 1.07 |
| Stroke | 1.06 | 1.10 | 1.12 |
| Hearing problems | 1.06 | 1.06 | 1.18 |
| ADL | 1.17 | 1.17 | 1.33 |
| IADL | 1.18 | 1.16 | 1.30 |
| Depressive symptom at baseline | 1.40 | 1.40 | 1.49 |
| Ratio of change in depressive symptom 4 years later | 1.22 | 1.22 | 1.31 |
| Quality of life | 1.21 | 1.21 | 1.35 |
| MMSE score at baseline | 1.09 | 1.10 | 1.19 |
| Participation in church or other religious groups | 1.14 | 1.15 | 1.20 |
| Participation in senior citizen clubs or senior centers | 1.12 | 1.12 | 1.23 |
| Participation in alumni societies or family councils | 1.14 | 1.15 | 1.15 |
| Level of face-to-face contact with close friends | 1.12 | 1.09 | 1.31 |
| Level of face-to-face contact with one’s children | 1.66 | 1.66 | 1.71 |
| Level of contact with one’s children by phone or letter | 1.68 | 1.68 | 1.75 |
| Mean VIF | 1.28 | 1.29 | 1.37 |

**Supplementary Table 7** Multivariate linear regression analysis of the associations between social activities and cognitive decline 4 years later

| Variable | Total | Young-old | Old-old |
| --- | --- | --- | --- |
|  | Coefficients (SE) | Coefficients (SE) | Coefficients (SE) |
| **Socio-demographics** |  |  |  |
| Age (years) | 0.24 (0.04) ^***^ | 0.24 (0.07) ^***^ | 0.06 (0.17) |
| Sex |  |  |  |
| Male (ref. female) | -0.50 (0.43) | -0.57 (0.46) | -0.30 (1.16) |
| Marital status |  |  |  |
| Married (ref. widowed) | -0.08 (0.21) | 0.04 (0.21) | -0.08 (0.54) |
| Education |  |  |  |
| ≥ Middle school (ref. ≤Elementary school) | -0.15(0.40) | -0.10 (0.42) | -0.49 (0.98) |
| Household income |  |  |  |
| > 1 quartile (ref. ≤1 quartile) | 0.64 (0.38) | 0.01 (0.42) | 2.64 (0.93) ^**^ |
| Living arrangement |  |  |  |
| Alone (ref. with someone) | 0.23 (0.69) | 0.02 (0.72) | .43 (1.81) |
| Residential area |  |  |  |
| Urban (ref. rural) | -0.65 (0.39) | -0.61 (0.41) | -0.89 (1.00) |
| **Health-related variables** |  |  |  |
| Hypertension (yes *vs*. no) | 0.43 (0.38) | 0.12 (0.40) | 1.59 (0.94) |
| Diabetes mellitus (yes *vs*. no) | -0.63 (0.40) | -0.52 (0.44) | -1.72 (1.01) |
| Cancer (yes *vs*. no) | 1.45 (1.21) | -1.09 (0.76) | 6.66 (3.04) ^*^ |
| Heart disease (yes *vs*. no) | -0.30 (0.60) | -0.35 (0.62) | -0.47 (1.69) |
| Stroke (yes *vs*. no) | 1.30 (0.97) | 0.64 (0.91) | 1.70 (2.38) |
| Hearing problems (yes *vs*. no) | 0.68 (0.97) | 0.02 (1.13) | 2.15 (2.06) |
| ADL |  |  |  |
| Dependent (ref. independent) | 4.28 (2.44) | 7.64 (2.97) ^*^ | -2.88 (3.70) |
| IADL |  |  |  |
| Dependent (ref. independent) | -.63 (0.62) | -0.82 (0.66) | -0.16 (1.43) |
| **Mental well-being** |  |  |  |
| Depressive symptom score at baseline | 0.13(.05)^**^ | 0.08(.05) | 0.29(0.11)^**^ |
| Ratio of change in depressive symptom 4 years later | 0.52(.12)^***^ | 0.41(.12)^**^ | 0.84(.32)^**^ |
| Quality of life | -0.02 (0.01) | -0.03 (0.01) ^**^ | 0.01 (0.02) |
| MMSE score at baseline | 0.46 (0.11) ^***^ | 0.44 (0.11) ^***^ | 0.58 (0.27)^*^ |
| **Formal social activities** |  |  |  |
| Participation in church or other religious groups (yes *vs*. no) | -0.09 (0.45) | 0.12 (0.50) | -0.39 (1.06) |
| Participation in senior citizen clubs or senior centers (yes *vs*. no) | -0.95 (0.37) ^*^ | -0.80 (0.40) ^*^ | -1.02 (0.97) |
| Participation in alumni societies or family councils (yes *vs*. no) | 0.46 (0.60) | 0.64 (0.64) | -0.04 (1.46) |
| **Informal social activity** |  |  |  |
| Level of face-to-face contact with close friends | 0.05 (0.09) | -0.01 (0.09) | -0.21 (0.23) |
| Level of face-to-face contact with one’s children | 0.34 (0.15)* | 0.08 (0.15) | 1.54 (0.45) ^**^ |
| Level of contact with one’s children by phone or letter | -0.30 (0.15) | -0.14 (0.15) | -0.95 (0.45) ^*^ |
| Adjusted R-square | 0.08 | 0.07 | 0.16 |
| F-value | 4.36^***^ | 3.24^***^ | 2.45^**^ |

Note: SE = standard error ^*^ p < 0.05, ^**^ p < 0.01, ^***^ p < 0.001
