# Supplementary material for: Which type of social activities may reduce cognitive decline in the elderly?: a longitudinal population-based study
Source: BMC Geriatr. 2016 Sep 27;16:165. doi: 10.1186/s12877-016-0343-x (PMC5039914; doi:10.1186/s12877-016-0343-x)
Supplement: Additional file 2: — KLoSA_2006_Wave1_English Questionnaire. (PDF 1994 kb) [file 12877_2016_343_MOESM2_ESM.pdf]

http://klosa.kli.re.kr

KLoSA

Korean Longitudinal Study of Ageing

2006 KLoSA

Wave1 Questionnaire

KLI

KOREA LABOR INSTITUTE

2006 KLoSA Wave 1 Questionnaire

TABLE OF CONTENTS

CV. COVERSCREEN 3

A. DEMOGRAPHICS 9

B. FAMILY AND FAMILY TRANSFER 15

Ba. FAMILY AND FAMILY TRANSFER - CHILDREN & GRANDCHILDREN
Bb. FAMILY AND FAMILY TRANSFER - PARENTS & SIBLINGS

C. HEALTH 67

Ca. HEALTH STATUS
Cb. FUNCTIONAL LIMITATIONS AND HELPERS
Cc. HEALTH INSURANCE AND SERVICES
Cd. COGNITION
Ce. GRIP STRENGTH

D. EMPLOYMENT 115

E. INCOME 169

F. ASSETS AND DEBTS 195

G. EXPECTATIONS AND LIFE SATISFACTION 231

CV. COVERSCREEN

CV. COVERSCREEN SURVEY STRUCTURE

CV002 : CHECK THAT THE HOUSEHOLD(HH) MEMBERS OLDER THAN 45 YEARS OLD

CV004-CV035 : INFORMATION ABOUT HH MEMBERS

CV004-CV009 : CV RESPONDENT INFORMATION (NAME, SEX, THE DATE OF BIRTH, MARITAL STATUS)
CV010-CV014 : CV R'S SPOUSE INFORMATION(NAME, SEX, THE DATE OF BIRTH)
CV015-CV024 : OTHER HH MEMBERS INFORMATION (NAME, SEX, RELATIONSHIP WITH R., DATE OF BIRTH, MARITAL STATUS)
CV025-CV032 : HH MEMBER'S INFORMATION(NAME, SEX, RELATIONSHIP WITH CV R.)
HH MEMBER'S SPOUSE INFORMATION
CV033 : CHECK THAT OTHER HH
CV035 : CHECKING THE TOTAL HH MEMBERS

CV037 : CHECKING LIST OF ELIGIBLE HH MEMBERS
CV050-CV051 : TOTAL HOUSEHOLD INCOME AND ASSET RESPONDENT
CV039 : CHECK THAT COVERSCREEN COMPLETED
CV036 : CHECK THAT THERE IS NO ELIGIBLE PERSON

CV002. We appreciate your participation. Korean Longitudinal Study of Ageing (KLoSA) is interested in learning about important aspects of people's lives such as their health, financial and family situations and applying the results to the social welfare and labor policy. The survey sample is 10,000 Koreans aged over 45 and is conducted every year.

Is there anyone among your household members born before 1962 (older than 45 years old)?

[1] yes [5] no → Go to CV036
Don't know → Go to CV004

The definition of household members: the ones who reside in the same housing unit and related with blood or marriage (co-habitation included).

[The followings are considered as a household member]

① a spouse residing separately due to the workplace, etc.
② child(ren) who is(are) financially dependent but residing separately due to the education, work, etc.
③ relatives who reside together

[The followings are NOT considered as a household member]

① unmarried child(ren) who is(are) financially independent and residing separately
② married child(ren) who resides(reside) separately
③ temporarily visiting or residing relative(s)
④ non-relatives such as a housemaid, chauffeur, or boarder
⑤ family who reside separately due to the army service, working or studying abroad, or long-term institutionalization

CV004. What is your first name?

____

CV005. What is your gender?

IWER: Ask only if distinguishing gender is difficult and if not, make an educated guess.

[1] Male [2] Female

CV007. In what year were you born?

________________________(range: 1900~2006)

CV009. What is your marital status?

IWER: common-law marriage is considered as married

[1] married or living with a spouse
[2] separated / divorced / widowed } → Go to CV015
[3] never married } → Go to CV015

CV010. What is your spouse's first name?

____

CV011. What is the gender of your spouse?

IWER: Ask only if distinguishing gender is difficult and if not, make an educated guess.

[1] Male [2] Female

CV013. In what year was [he / she] born?

CV015. I like to know a little bit about people whom you live with. First, do you live with other household members?

[1] yes [5] no → Go to CV033

LOOP CHECKPOINT : FOR EACH HOUSEHOLD MEMBER, THE FOLLOWING QUESTIONS ARE ASKED: CV016 ~ CV033.

CV016. What is his or her name?

IWER: Please list other household members in the order of age

----

CV017. What is [HH MEMBER's name]'s sex?

[1] male [2] female

CV018. What is [HHMEMBER's name]'s relationship to you?

[3] daughter / son
[4] daughter-in-law / son-in-law
[5] mother / father
[6] mother-in-law / father-in-law
[7] sister / brother
[8] grandchild

} → Go to CV022

[9] other

CV019. Please specify the other.

CV022. When was [HH MEMBER's name] born?

_________________________(range: 1900~2006)
Don't know } → Go to CV024

CV023. Was [HH MEMBER's name] born before or after the year 1962?

[1] before the year 1962
[2] in the year 1962
[3] after the year 1962

CV024. What is [HH MEMBER's name]'s marital status? [IWER: common-law marriage is considered as married]

IWER: common-law marriage is considered as married

[1] Married or living with a spouse
[2] Separated / Divorced / Widowed
[3] Never married } → Go to CV033

CV025. What is the name of [HH MEMBER's name]'s spouse?

____

CV026. What is [HH MEMBER's spouse's name]'s gender?

IWER: Ask if distinguishing gender is difficult and if not, make an educated guess.

[1] male [2] female

CV027. What is [HH MEMBER's spouse's] relationship to you?

[3] daughter / son
[4] daughter-in-law / son-in-law
[5] mother / father
[6] mother-in-law / father-in-law
[7] sister / brother
[8] grandchild } → Go to CV031
[9] other

CV028. Please specify the other.

____

CV031. When was [HH Member's Spouse's name] born?

_________________________(range: 1900~2006) → Go to CV033
Don't know → Go to CV032

CV032. Was [HH Member's Spouse's first name] born before or after the year 1962?

[1] before the year 1962
[2] in the year 1962
[3] after the year 1962

CV033. Are there other household members?

[1] yes [5] no

CV035. In your household there are [HHSize] people. Those are as follows. [LIST OF ALL PERSONS IN HH] Are there other household members?

IWER: Confirm all listed household members.

[1] yes [5] no

ELIGIBILITY CHECKPOINT: AGE ELIGIBLE RESPONDENT EXISTS?
IF THERE IS ANY AGE QUALIFYING R IN THE HOUSEHOLD, GO TO CV037.

CV037. Now I am going to start main interview.

List of eligible household members

[1] CONTINUE

CV050. I will be asking some questions about household total income. Who would be the most knowledgeable about this among the following household members?

01 ~ 20 [CAPI LIST OF ELIGIBLE PERSONS IN HH WITH PERSON ID]

CV051. I will be asking some questions about household total assets. Who would be the most knowledgeable person about this among the following household members?

01 ~ 20 [CAPI LIST OF ELIGIBLE PERSONS IN HH WITH PERSON ID]

CV039. IWER: Coverscreen is completed. Please select 1.

[1] CONTINUE

A. DEMOGRAPHICS

A. DEMOGRAPHICS SURVEY STRUCTURE

A001-A007 : RESPONDENT INFORMATION

A001-A002 : THE DATE OF BIRTH
A003-A005 : EDUCATION BACKGROUND
A006-A007 : MARITAL STATUS

A008-A013 : SPOUSE THAT IS NOT ELIGIBLE THIS SURVEY(NOT LIVING TOGETHER OR SPOUSE UNDER 45 YEARS OLD) INFORMATION

A008 : SPOUSE'S DATE OF BIRTH
A009-A011 : SPOUSE'S EDUCATION BACKGROUND
A012-A013 : SPOUSE'S EMPLOYMENT STATUS

A014-A019 : RELIGIOUS PREFERENCE, SOCIAL RELATIONSHIP, AND TIMES OF ACTIVITY

A014-A015 : RELIGIOUS PREFERENCE
A016 : TIMES OF MEETING WITH NEIGHBOR(FRIENDS AND SO ON)
A017-A019 : SOCIAL ACTIVITY

A020-A021 : CHECKPOINT OF INTERVIEWER

※ **NOTE: THOSE WHO WERE BORN AFTER JANUARY 1, 1962 WILL NOT BE ASKED.**

A001. In what year, month, and day were you born?

IWER: For those who respond by zodiac sign, please refer to the index card provided

____________Year ____________Month ____________Day

A002. Is your date of birth according to the solar calendar or the lunar calendar?

[1] Solar calendar [5] Lunar calendar

A003. What is the highest level of school or colleage you completed?

[1] No formal education (illiterate) → Go to A006
[2] No formal education (capable of reading) → Go to A006
[3] Elementary school → Go to A005
[4] Middle school → Go to A005
[5] High school → Go to A005
[6] Two-year college → Go to A005
[7] College grad → Go to A005
[8] Post college (Master) → Go to A005
[9] Post college (PhD) → Go to A005
[97] Other
[98] Don't know → Go to A006
[99] Refuse to answer → Go to A006

A004. Please specify other.

------------------------- → Go to A014

A005. Did you get a diploma or pass an equivalency test?

[1] Currently enrolled
[2] Yes, diploma
[3] Dropped out
[4] Yes, equivalency
[5] Completed course of study

A006. Are you currently married, living with a partner, separated, divorced, widowed, or have you never been married?

[1] Currently married or living with a partner (i.e., common-law marriage)
[2] Separated → Go to A008
[3] Divorced → Go to A008
[4] Widowed or missing (dispersed family) → Go to A008
[5] Never married → Go to A014
[98] Don't know → Go to A014
[99] Refuse to answer → Go to A014

A007. What is your spouse name?

SKIP PATTERN CHECKPOINT : SPOUSE ASK A008 ~ A013 IF THE SPOUSE IS LIVING WITH R (A006 = [1]) AND THE SPOUSE'S AGE IS UNDER 45
ASK A008 ~ A011 AND SKIP TO A014 IF ANSWERED [2], [3], [4] IN A006 AND THE SPOUSE'S AGE IS UNDER 45

A008. In what year, month, and day was your spouse born?

_____________Year _____________Month _____________Day

A009. What is the highest level of school or college your spouse completed?

[1] No formal education (illiterate) → Go to A012
[2] No formal education (capable of reading) → Go to A012
[3] Elementary school → Go to A011
[4] Middle school → Go to A011
[5] High school → Go to A011
[6] Two-year college → Go to A011
[7] College grad → Go to A011
[8] Post college (Master) → Go to A011
[9] Post college (PhD) → Go to A011
[97] Other
[98] Don't know → Go to A012
[99] Refuse to answer → Go to A012

A010. Please specify other.

____

A011. Did your spouse get a diploma or pass an equivalency test?

[1] Currently enrolled
[2] Yes, diploma
[3] Dropped out
[4] Yes, equivalency
[5] Completed course of study

A012. Now I'm going to ask you some questions about your spouse's current employment situation. Is your spouse currently working, temporarily laid off, unemployed and looking for work, retired, a home maker, or what? If she/he is working, please tell me she/he has a full-time or part-time job.

IWER: Retirement refers to having stopped working for pay or leaving a job or career and currently not working or engaging in only small pastime work, without intention to go back to work beyond small pastime work.

[1] Full – time worker
[2] Part – time worker
[3] Temporarily laid off, on sick or other leave
[4] Unemployed and looking for work
[5] Retired
[6] Homemaker
[7] Other

(Options 1–7) → Go to A014

A013. Please specify other.

A014. What is your religious preference? Is it protestant, catholic, Buddhism, Won Buddhism, some other religion or do you have no preference?

[1] No preference
[2] Protestant
[3] Catholic
[4] Buddhist
[5] Won Buddhist
[6] Other

(Options 1–5) → Go to A016

A015. Please specify other.

A016. Do you have any close friends or relatives who live nearby and have a close relationship with? If so, how often do you meet them in person? Please refer to the person whom you meet most often.

[1] Almost every day (more than 4 times per week)
[2] Once a week
[3] 2-3 times a week
[4] Once a month
[5] Twice a month (every two weeks)
[6] Once or twice a year
[7] Three or for times a year (once every three or four months)
[8] Five or six times a year (every two months)
[9] Almost never
[10] No close friend or relative

A017. Are you a member of any of the following organizations, clubs, or societies? Please select all that apply.

[1] Church or other religious groups → Go to A019
[2] Social clubs (e.g., private savings club, senior citizens' club, etc) → Go to A019
[3] Sports clubs, arts or music groups, or classes for senior → Go to A019
[4] Alumni society, society for people from the same hometown, family councils → Go to A019
[5] Volunteer groups → Go to A019
[6] Political party, NGOs, interest groups → Go to A019
[7] Others
[8] None → Go to A020
[98] Don't know → Go to A020
[99] Refuse to answer → Go to A020

A018. Please specify other.

____

LOOP CHECKPOINT: SOCIAL ACTIVITIES REPEAT QUESTION A019 ACCORDING TO THE NUMBER CHOSEN IN A017.

A019. Thinking about this organization that you are a member of [chosen from A017], how often do you engage in its activities?

[1] Almost every day (more than 4 times per week)
[2] Once a week
[3] 2-3 times a week
[4] Once a month
[5] Twice a month (every two weeks)
[6] Once or twice a year
[7] Three or four times a year (once every three or four months)
[8] Five or six times a year (every two months)
[9] Almost never a year
[10] Almost never

A020. IWER: HOW OFTEN DID R RECEIVE ASSISTANCE IN ANSWERS IN SECTION A – DEMOGRAPHICS?

[1] Never
[2] A few times
[3] Most or all of the time
} → Go to Section Ba.
[4] The Section was done by a proxy reporter.

A021. IWER: WHAT IS THE PROXY'S RELATIONSHIP TO R? IF UNKNOWN, PLEASE ASK THE PROXY What is your relationship to R?

[1] Spouse
[2] Mother
[3] Father
[4] Mother – in – law
[5] Father – in – law
[6] Sibling
[7] Brother-in-law, sister-in-law
[8] Child
[9] Spouse of child
[10] Grandchild
[11] Other relative
[12] Helper or other non-relative
→ Go to Section Ba.

B. FAMILY

Ba. FAMILY AND FAMILY TRANSFER - CHILDREN & GRANDCHILDREN

Ba. FAMILY AND FAMILY TRANSFER (CHILDREN & GRANDCHILDREN) SURVEY STRUCTURE

* This section is organized by questions for children and grandchildren, so one person among married couple have to answer this question
* The questions for "geographical approach and times of contact" and "transfer among family members (monetary /non-monetary transfer)" is for only children that is not living together

Ba01-Ba46 : CHILDREN INFORMATION

Ba01 : NUMBERS OF CHILDREN
Ba02-Ba11 : BASIC INFORMATION FOR CHILDREN (NAME, SEX, AGE, EDUCATION BACKGROUND, EMPLOYMENT STATUS, HOUSE OWNERSHIP, MARITAL STATUS, NUMBERS OF CHILDREN)
Ba12 : GEOGRAPHICAL APPROACH
Ba13-Ba14 : CONTACT INFORMATION (TIMES OF CONTACT IN PERSON, TIMES OF CONTACT BY PHONE, MAIL OR E-MAIL)
Ba15-Ba30 : FINANCIAL TRANSFER FROM CHILDREN
Ba31-Ba46 : FINANCIAL TRANSFER TO CHILDREN

Ba47-Ba50 : GRANDCHILDREN
Ba47 : NUMBERS OF GRANDCHILDREN
Ba48-Ba50 : CARING WORK FOR GRANDCHILDREN - TERMS/HOURS OF CARING WORK

Ba51 : CHECK POINT OF INTERVIEWER

Ba01. Now I am going to ask you about your children. How many living children do you have?

_________________________(Range: 0~20)

Don't know
Refuse to answer } → Go to Ba47

Loop: Ba02 ~ Ba46, but from Ba12 to Ba46 not living together child only

Ba02. What is your child's name? (Please start with the eldest, in birth order)

IWER: Please double-check the spelling of the name, if the same name is used in COVERSCREEN. If R does not provide the child's name, write down the birth-order of child, e.g., 'the first born' or 'the second born'.

____

Ba03. Is your CHILDn's NAME son or daughter?

[1] Son [5] Daughter

Ba04. How old is CHILDn's NAME?

IWER: For those who respond by zodiac sign, refer to the index card providing the according years

_________________________year-old (range: 1~100)

Ba05. What is the highest level of school or college your CHILDn's NAME completed?

[1] No formal education (illiterate) → Go to Ba08
[2] No formal education (capable of reading) → Go to Ba08
[3] Elementary school → Go to Ba07
[4] Middle school → Go to Ba07
[5] High school → Go to Ba07
[6] Two-year college → Go to Ba07
[7] College grad → Go to Ba07
[8] Post college (Master) → Go to Ba07
[9] Post college (PhD) → Go to Ba07
[97] Other

Ba06. Please specify the other.

____

Ba07. Did your CHILDn's NAME get a diploma or pass an equivalency test?

[1] Currently enrolled
[2] Yes, diploma
[3] Dropped out
[4] Yes, equivalency
[5] Completed course of study

Ba08. Does CHILDn's NAME work for pay?

[1] Yes [5] No

Ba60. Is CHILDn's NAME currently co-residing with you?

IWER: If a child lives away from home due to schooling or job, the child is NOT co-residing with R.

[1] Yes [5] No

Ba09. Does CHILDn's NAME own a home?

[1] Yes [5] No

Ba10. Is CHILDn's NAME married?

[1] Currently married or living together → Go to A007
[2] Separated
[3] Divorced
[4] Widowed or missing (Dispersed family)
[5] Never been married
[6] Don't know
[9] Refuse to answer

Ba11. Does CHILDn's NAME have any children? If so, how many?

_________________________(range: 0~20)

Ba12. How close does CHILDn's NAME live from you?

[1] Within a 30-minute distance by public transportation
[2] Within a 1-hour distance by public transportation
[3] Within a 2-hour distance by public transportation
[4] More than a 2-hour distance by public transportation

Ba13. How often have you had contact CHILDn's NAME in person?

[1] Almost every day (more than 4 times per week)
[2] Once a week
[3] 2－3 times a week
[4] Once a month
[5] Twice a month (every two weeks)
[6] Once or twice a year
[7] Three or four times a year (once every three or four months)
[8] Five or six times a year (every two months)
[9] Almost never a year
[10] Never

Ba14. How often have you had contact CHILDn's NAME by phone, mail or e - mail?

[1] Almost every day (more than 4 times per week)
[2] Once a week
[3] 2-3 times a week
[4] Once a month
[5] Twice a month (every two weeks)
[6] Once or twice a year
[7] Three or four times a year (once every three or four months)
[8] Five or six times a year (every two months)
[9] Almost never a year
[10] Never

Ba15. Not counting any shared housing or shared food, during the LAST CALENDAR YEAR(2005) did you [and your spouse] receive any financial help from CHILDn's NAME?

IWER: －By financial help we mean giving money, helping pay bills, or covering specific types of costs such as those for medical care or insurance, schooling, down payment for a home, rent, etc., but not counting any shared housing or shared food. The financial help can be considered support, a gift or a loan.

－Regular monetary transfer refers to the case in which you received monetary transfers regularly in a certain time interval (e.g., each month, every two months), such as monthly allowances.

－Occasional monetary transfer refers to the case in which you received monetary transfers without any regularity, such as paying for medical bills

or schooling and occasional allowances.
- Monetary transfers include receiving cash as well as bills paid for your behalf. For example, your family member pays for your medical bills, insurance, tuition and so on.
- Non-monetary transfer refers to gifts and goods received.

[1] Yes, received regular monetary transfer → Ba16
[2] Yes, received occasional monetary transfer → Ba16 or Ba23
[3] Yes, non-monetary transfer → Ba16 or Ba23 or Ba29
[5] No, I did not receive any financial help → Ba31

SKIP PATTERN CHECKPOINT: REGULAR MONETARY TRANSFER RECEIVED IF R RECEIVED REGULAR MONETARY TRANSFER IN Ba15, GO TO Ba16. IF R DID NOT RECEIVE REGULAR MONETARY, GO TO SKIP PATTERN.

Ba16. What was the monthly average amount of regular monetary transfer you received from CHILDn's FIRST NAME? (Unit: 10,000 Korean won)

__________________________MW (range: 1 ~ 9997) → Go to Ba22
Don't know
Refuse to answer } → Go to Ba17~Ba21 unfolding

NOTES ON UNFOLDING BRACKET QUESTIONS

- WHEN R SAYS "DON'T KNOW" OR "REFUSE" TO FINANCIAL QUESTIONS (I.E., FAMILY TRANSFER, HEALTH CARE EXPENDITURE, INCOME, AND ASSETS, R WILL BE ASKED WITH "UNFOLDING BRACKET QUESTIONS."
- IN ALL UNFOLDING BRACKET QUESTIONS, A THREHOLD IS PRESENTED, AND R IS ASKED TO RESPOND TO WHETHER THE AMOUNT IS "LESS THAN," "ABOUT EQUAL TO," OR "MORE THAN" THE THRESHOLD.
- THERE ARE 5 THRESHOLDS FOR EACH SET OF UNFOLDING QUESTIONS: THRESHOLDS 1, 2, 3, 4, AND 5, IN AN INCREASING ORDER.
- R IS ASKED WITH A MAXIMUM OF 3 QUESTIONS, PRESENTED IN THE FOLLOWING 3 SCENARIOS.
- R IS RANDOMLY ASSGINED TO 1 OF THESE 3 SCENARIOS.

Ba17 ~ Ba21. UNFOLDING BRACKET QUESTIONS

Ba17. Did it amount to less than, about equal to or more than 10 MW (10,000 Korean won)?

IWER: Go back to Ba16 and record if R answers the amount during the interview. All figures are in MW, which is 10,000 Korean won.

[1] Less than 10 MW
[3] About 10 MW
[5] More than 10 MW

Ba18. Did it amount to less than, about equal to or more than 30 MW (10,000 Korean won)?

IWER: Go back to Ba16 and record if R answers the amount during the interview. All figures are in MW, which is 10,000 Korean won.

[1] Less than 30 MW
[3] About 30 MW
[5] More than 30 MW

Ba19. Did it amount to less than, about equal to or more than 50 MW (10,000 Korean won)?

IWER: Go back to Ba16 and record if R answers the amount during the interview. All figures are in MW, which is 10,000 Korean won.

[1] Less than 50 MW
[3] About 50 MW
[5] More than 50 MW

Ba20. Did it amount to less than, about equal to or more than 100 MW (10,000 Korean won)?

IWER: Go back to Ba16 and record if R answers the amount during the interview. All figures are in MW, which is 10,000 Korean won.

[1] Less than 100 MW
[3] About 100 MW
[5] More than 100 MW

Ba21. Did it amount to less than, about equal to or more than 200 MW (10,000 Korean won)?

IWER: Go back to Ba16 and record if R answers the amount during the interview. All figures are in MW, which is 10,000 Korean won.

[1] Less than 200 MW
[3] About 200 MW
[5] More than 200 MW

Ba22. Which month(s) did you receive such regular monetary help from CHILDn's NAME in LAST YEAR(2005)? Please answer all that apply.

[1] January 2005
[2] February 2005
[3] March 2005
[4] April 2005
[5] May 2005
[6] June 2005
[7] July 2005
[8] August 2005
[9] September 2005
[10] October 2005
[11] November 2005
[12] December 2005
[13] All year round

SKIP PATTERN CHECKPOINT: OCCASIONAL MONETARY TRANSFER RECEIVED IF R RECEIVED OCCASIONAL MONETARY TRANSFER IN Ba15, GO TO Ba23. IF R DID NOT RECEIVE OCCASIONAL MONETARY TRANSFER, GO TO SKIP PATTERN CHECKPOINT: NON-MONETARY TRANSFER.

Ba23. During the LAST CALENDAR YEAR(2005), what was the total amount of occasional monetary transfers you received from CHILDn's NAME? (Unit: 10,000 Korean won)

_________________________MW (range: 1~99997) → Go to Ba29

Don't know
Refuse to answer } → Go to Ba17~Ba21 unfolding

Ba24 ~ Ba28. UNFOLDING BRACKET QUESTIONS

Ba24. Did it amount to less than, about equal to or more than 100 MW (10,000 Korean won)?

IWER: Go back to Ba23 and record if R answers the amount during the interview.

[1] Less than 100 MW
[3] About 100 MW
[5] More than 100 MW

Ba25. Did it amount to less than, about equal to or more than 300 MW (10,000 Korean won)?

IWER: Go back to Ba23 and record if R answers the amount during the interview.

[1] Less than 300 MW
[3] About 300 MW
[5] More than 300 MW

Ba26. Did it amount to less than, about equal to or more than 500 MW (10,000 Korean won)?

IWER: Go back to Ba23 and record if R answers the amount during the interview.

[1] Less than 500 MW
[3] About 500 MW
[5] More than 500 MW

Ba27. Did it amount to less than, about equal to or more than 1,000 MW (10,000 Korean won)?

IWER: Go back to Ba23 and record if R answers the amount during the interview.

[1] Less than 1,000 MW
[3] About 1,000 MW
[5] More than 1,000 MW

Ba28. Did it amount to less than, about equal to or more than 2,000 MW (10,000 Korean won)?

IWER: Go back to Ba23 and record if R answers the amount during the interview.

[1] Less than 2,000 MW
[3] About 2,000 MW
[5] More than 2,000 MW

SKIP PATTERN CHECKPOINT: NON-MONETARY TRANSFER RECEIVED IF R RECEIVED NON-MONETARY TRANSFER IN Ba15, GO TO Ba29.
IF R DID NOT RECEIVE NON-MONETARY TRANSFER, GO TO Ba31.

Ba29. What was the type of non-monetary support? Please check all that apply.

[1] Leisure (e.g., travel)
[2] Health-related products (e.g., vitamins, equipments, etc.)
[3] Household items
[4] Electronics
[5] Dining out and foods
[6] Other

→ Go to Ba31

Ba30. Please specify other.

____

Ba31. Not counting any shared housing or shared food, during the LAST CALENDAR YEAR (2005) did you [and your spouse] give any financial help to CHILDn's NAME?

[1] Yes, made regular monetary transfer → Go to Ba32
[2] Yes, made occasional monetary transfer → Go to Ba32 or Ba39
[3] Yes, made non-monetary transfer → Go to Ba32 or Ba39 or Ba45
[5] No, I did not give any financial help → Go to Ba47

SKIP PATTERN CHECKPOINT: REGULAR MONETARY TRANSFER GIVEN IF R MADE REGULAR MONETARY TRANSFER IN Ba31, GO TO Ba32.
IF R DID NOT MAKE REGULAR MONETARY TRANSFER, GO TO SKIP PATTERN CHECKPOINT: OCCASIONAL MONETARY TRANSFER GIVEN.

Ba32. What was the monthly average amount of regular monetary transfer you gave to CHILDn's NAME? (Unit: 10,000 Korean won)

_________________________MW (range: 1~9997) → Go to Ba38

Don't know
Refuse to answer
→ Go to Ba33~Ba37 Unfolding

Ba33 ~ Ba37. UNFOLDING BRACKET QUESTIONS

Ba33. Did it amount to less than, about equal to or more than 10 MW (10,000 Korean won)?

IWER: Go back to Ba32 and record if R answers the amount during the interview.

[1] Less than 10 MW
[3] About 10 MW
[5] More than 10 MW

Ba34. Did it amount to less than, about equal to or more than 30 MW (10,000 Korean won)?

IWER: Go back to Ba32 and record if R answers the amount during the interview.

[1] Less than 30 MW
[3] About 30 MW
[5] More than 30 MW

Ba35. Did it amount to less than, about equal to or more than 50 MW (10,000 Korean won)?

IWER: Go back to Ba32 and record if R answers the amount during the interview.

[1] Less than 50 MW
[3] About 50 MW
[5] More than 50 MW

Ba36. Did it amount to less than, about equal to or more than 100 MW (10,000 Korean won)?

IWER: Go back to Ba32 and record if R answers the amount during the interview.

[1] Less than 100 MW
[3] About 100 MW
[5] More than 100 MW

Ba37. Did it amount to less than, about equal to or more than 200 MW (10,000 Korean won)?

IWER: Go back to Ba32 and record if R answers the amount during the interview.

[1] Less than 200 MW
[3] About 200 MW
[5] More than 200 MW

Ba38. Which months did you make regular monetary transfers during the LAST YEAR (2005) to CHILDn's NAME? Please answer all that apply.

[1] January 2005
[2] February 2005
[3] March 2005
[4] April 2005
[5] May 2005
[6] June 2005

[7] July 2005
[8] August 2005
[9] September 2005
[10] October 2005
[11] November 2005
[12] December 2005
[13] All year round

SKIP PATTERN CHECKPOINT: OCCASIONAL MONETARY TRANSFER GIVEN IF R MADE OCCASIONAL MONETARY TRANSFER IN Ba31, GO TO Ba39.
IF R DID NOT MAKE OCCASIONAL MONETARY TRANSFER, GO TO SKIP PATTERN CHECKPOINT: NON-MONETARY TRANSFER GIVEN, Ba45.

Ba39. During the LAST CALENDAR YEAR(2005) what was the total amount of occasional monetary transfer given to CHILDn's NAME? (Unit: 10,000 Korean won)

________________________MW (range: 1~99997) → Go to Ba45

Don't know
Refuses to answer } → Go to Ba40~Ba44 unfolding

Ba40 ~ Ba44. UNFOLDING BRACKET QUESTIONS

Ba40. Did it amount to less than, about equal to or more than 100 MW (10,000 Korean won)?

IWER: Go back to Ba39 and record if R answers the amount during the interview.

[1] Less than 100 MW
[3] About 100 MW
[5] More than 100 MW

Ba41. Did it amount to less than, about equal to or more than 300 MW (10,000 Korean won)?

IWER: Go back to Ba39 and record if R answers the amount during the interview.

[1] Less than 300 MW
[3] About 300 MW
[5] More than 300 MW

Ba42. Did it amount to less than, about equal to or more than 500 MW (10,000 Korean won)?

IWER: Go back to Ba39 and record if R answers the amount during the interview.

[1] Less than 500 MW
[3] About 500 MW
[5] More than 500 MW

Ba43. Did it amount to less than, about equal to or more than 1,000 MW (10,000 Korean won)?

IWER: Go back to Ba39 and record if R answers the amount during the interview.

[1] Less than 1,000 MW
[3] About 1,000 MW
[5] More than 1,000 MW

Ba44. Did it amount to less than, about equal to or more than 2,000 MW (10,000 Korean won)?

IWER: Go back to Ba39 and record if R answers the amount during the interview.

[1] Less than 2,000 MW
[3] About 2,000 MW
[5] More than 2,000 MW

SKIP PATTERN CHECKPOINT: NON-MONETARY TRANSFER GIVEN IF R MADE NON-MONETARY TRANSFER IN Ba31, GO TO Ba45.
IF R DID NOT MAKE NON-MONETARY TRANSFER, GO TO Ba47.

Ba45. What was the type of non-monetary transfer given? Please choose all that apply.

[1] Leisure (e.g., travel)
[2] Health-related products (e.g., vitamins, equipments, etc.)
[3] Household items
[4] Electronics
[5] Dining out and foods
→ Go to Ba47 (for [1]–[5])
[6] Other

Ba46. Please specify other.

____

Ba47. Now, I like to ask you few questions about grandchildren. Altogether, how many living grandchildren do you have? Please include those whose parent is dead.

_____________(range: 0~100)

Ba48. During the past 12 months (not calendar year), did you take care of any of your grandchildren younger than 10 years old?

[1] Yes
[5] No → Go to Ba51

Ba49. Roughly how many hours did you spent on caring for grandchildren during the past 12 months?

_______________hours per week

Ba50. For how long did you give such assistance?

IWER: Calculate 1 month as 4 weeks, 6 months as 26 weeks, 1 year as 52 weeks. LAST YEAR means a year from now, not 2005.

_________ weeks (range: 1~52)

Ba51. IWER: HOW OFTEN DID R RECEIVE ASSISTANCE WITH ANSWERS IN SECTION Ba－FAMILY (CHILDREN & GRANDCHILDREN) AND FAMILY TRANSFER?

[1] Never
[2] A few times
[3] Most or all of time
} → Go to Section Bb.
[4] The Section was done by a proxy reporter.

Ba52. IWER: WHAT IS THE PROXY'S RELATIONSHIP TO R? IF UNKNOWN, PLEASE ASK THE PROXY What is your relationship to R?

[1] Spouse
[2] Mother
[3] Father
[4] Mother-in-law
[5] Father-in-law
[6] Sibling
[7] Brother-in-law, sister-in-law
[8] Child
[9] Spouse of child
[10] Grandchild
[11] Other relative
[12] Helper or other non-relative
→ Go to Section Bb.

Bb. FAMILY AND FAMILY TRANSFER – PARENTS & SIBLINGS

Bb. FAMILY AND FAMILY TRANSFER(PARENTS AND SIBLINGS) SURVEY STRUCTURE

B001-B005 : SIBLINGS INFORMATION

B001 : NUMBERS OF SIBLINGS

B002-B005 : BASIC INFORMATION OF SIBLINGS – NAME, RELATIONSHIP TO RESPONDENT, AGE, MARITAL STATUS

B006-B140 : PARENTS INFORMATION

B006-B015 : FATHER (NAME, EDUCATION BACKGROUND, AGE, EMPLOYMENT STATUS, HOUSE OWNERSHIP, DATE OF DEATH, AGE AT THE TIME OF DEATH)

B016-B025 : MOTHER (NAME, EDUCATION BACKGROUND, AGE, EMPLOYMENT STATUS, HOUSE OWNERSHIP, DATE OF DEATH, AGE AT THE TIME OF DEATH)

B026 : WHETHER OR NOT PARENTS LIVE TOGETHER

** IF PARENTS LIVE TOGETHER **

B027 : PARENTS LIVE BY THEMSELVES OR LIVE TOGETHER WITH CHILDREN

B028-B029 : CHILDREN THAT LIVE WITH PARENTS

B030 : GEOGRAPHICAL APPROACH TO PARENTS

B031-B032 : TIMES OF CONTACT TO PARENTS - TIMES OF CONTACT TO MOTHER BY A PERSON, OR BY PHONE/LETTER/E-MAIL

B033-B048 : FINANCIAL TRANSFER FROM PARENTS

B049-B064 : FINANCIAL TRANSFER TO PARENTS

** IF PARENTS LIVE SEPARATELY **

B065 : FATHER – WHETHER OR NOT LIVE WITH CHILDREN

B066-B067 : A PERSON THAT LIVE WITH PARENTS

B068 : GEOGRAPHICAL APPROACH TO FATHER

B069-B070 : TIMES OF CONTACT TO FATHER - TIMES OF CONTACT TO MOTHER BY A PERSON, OR BY PHONE/LETTER/E-MAIL

B071-B086 : FINANCIAL TRANSFER FROM FATHER

B087-B102 : FINANCIAL TRANSFER TO FATHER

B103 : MOTHER – WHETHER OR NOT LIVE WITH CHILDREN

B104-B105 : A PERSON THAT LIVE WITH MOTHER

B106 : GEOGRAPHICAL APPROACH TO MOTHER

B107-B108 : TIMES OF CONTACT TO MOTHER - TIMES OF CONTACT TO MOTHER BY A PERSON, OR BY PHONE/LETTER/E-MAIL

Bb. FAMILY AND FAMILY TRANSFER(PARENTS AND SIBLINGS) SURVEY STRUCTURE (Continued)

B109-B124 : FINANCIAL TRANSFER FROM MOTHER
B125-B140 : FINANCIAL TRANSFER TO MOTHER

B141-B178 : OTHER FAMILY

B141-B159 : FINANCIAL TRANSFER FROM OTHER FAMILY MEMBER
B160-B178 : FINANCIAL TRANSFER TO OTHER FAMILY MEMBER

B179-B185 : ADL/IADL CARING WORK QUESTIONS

B179 : A PERSON THAT UNABLE TO CARRY OUT ACTIVITIES OF DAILY LIVING (ADL)
B180 : A PERSON THAT PROVIDE HELP (ADL)
B181-B182 : A TERM/TIMES(WEEKS) THAT PROVIDE HELP (ADL)
B183 : A PERSON THAT UNABLE TO CARRY OUT INSTRUMENTAL ACTIVITIES SUCHA AS HOUSEHOLD CHORES, TRANSPORTATION AND SO ON (IADL)
B184-B185 : A TERM/TIMES(WEEKS) THAT PROVIDE HELP (IADL)

B186 : CHECKPOINT OF INTERVIEWER

* Sibling *

B001. Now I have some questions about your siblings. How many living siblings do you have?

IWER: Mark '0' if R has no sibling.

__________(range: 0~20) (IF R HAS NO SIBLING, SKIP TO B006)

LOOP CHECKPOINT: SIBLING REPEAT SIBLING QUESTIONS, B002 ~ B005 AND B187, FOR EACH SIBLING IN THE DECREASING ORDER OF AGE.

B002. What is your sibling's name?

IWER: If R doesn’t give the name, please specify the birth order and gender (e.g., first-born brother).

____

B003. What is SIBLING’s NAME’s relationship to you?

[1] Older sister
[2] Older brother

[3] Younger brother
[4] Younger sister

B004. How old is SIBLING's NAME?

IWER: For those who respond by zodiac sign, refer to the index card providing the according years

_________________________years old (range: 0~120)

B005. Is SIBLING's NAME married?

[1] Currently married or living together
[2] Separated
[3] Divorced
[4] Widowed or missing (Dispersed family)
[5] Never married
[8] Don't know
[9] Refuse to answer

B187. Does SIBLING's NAME live with you?

[1] Yes [5] No

* Parents *

B006. Now I have some questions about your parents. What is your father's name?

IWER: Mark 'father' if R doesn't give the name.

____

B007. What is the highest level of school or college your father completed?

[1] No formal education (illiterate) → Go to B010
[2] No formal education (capable of reading) → Go to B010
[3] Elementary school → Go to B009
[4] Middle school → Go to B009
[5] High school → Go to B009
[6] Two-year college → Go to B009
[7] College grad → Go to B009
[8] Post college (Master) → Go to B009
[9] Post college (PhD) → Go to B009

[97] Other
[98] Don't know → Go to B010
[99] Refuse to answer → Go to B010

B008. Please specify other.

B009. Did your father get a diploma or pass an equivalency test?
[1] Currently enrolled
[2] Yes, diploma
[3] Dropped out
[4] Yes, equivalency
[5] Completed course of study

B010. Is he alive?
[1] Yes
[3] Missing (Dispersed family) → B016
[5] No → B014

B011. How old is your father?
IWER: For those who respond by zodiac sign, refer to the index card providing the according years

___________years old (range: 1~120)

B012. Does your father work for pay?
[1] Yes [5] No

B013. Does your father own a house?
[1] Yes [5] No

B188. Does your father live with you?
[1] Yes [5] No

B014. In what year did he die?

___________________ year (range: 1900~2006)

B015. How old was your father when he died?

___________________years old (range: 1~120)

B016. Now I have some questions about your mother. What is your mother's name?

IWER: If R doesn't give the name, please write down 'mother'.

____

B017. What is the highest level of school or college your mother completed?

[1] No formal education (illiterate) → B020
[2] No formal education (capable of reading) → B020
[3] Elementary school → B019
[4] Middle school → B019
[5] High school → B019
[6] Two-year college → B019
[7] College grad → B019
[8] Post college (Master) → B019
[9] Post college (PhD) → B019
[97] Other
[98] Don't know → B020
[99] Refuse to answer → B020

B018. Please specify the other.

____

B019. Did your mother get a diploma or pass an equivalency test?

[1] Currently enrolled
[2] Yes, diploma
[3] Dropped out
[4] Yes, equivalency
[5] Completed course of study

B020. Is she alive?

[1] Yes
[3] Missing (Dispersed family) → B065
[5] No → B024

B021. How old is your mother?

IWER: For those who respond by zodiac sign, refer to the index card providing the according years

____________years old (range: 1~120)

B022. Does your mother work for pay?

[1] Yes [5] No

B023. Does your mother own a house?

[1] Yes [5] No

B189. Does your mother live with you?

[1] Yes → B026 [5] No

B024. In what year did she die?

_______________year (range: 1900~2006)

B025. How old was your mother when she died?

_________years old (range: 1~120)

Logic:
- **B010=1 and B020=1, R is not living together with him/her then go to B026,**
- **B010=5 and B020=5 then, go to B141,**
- **B010=1 and B020=5, and R is not living together with him/her then go to B065,**
- **B010=5 and B020=1, and R is not living together with him/her then go to B103**

B026. Do your parents live together?

[1] Yes [5] No → Go to B065

B027. Do they live by themselves, or with other child (ren) (R's sibling)?

[1] By themselves → Go to B030
[3] With other child (ren)
[5] Other → Go to B029

B028. With whom do they live with? If they live with multiple children, please identify all co-residents.

(Select from the list displayed by CAPI)

B029. Please specify the other.

____

B030. Please specify the other.

[1] Within a 30-minute distance by public transportation
[2] Within a 1-hour distance by public transportation
[3] Within a 2-hour distance by public transportation
[4] More than a 2-hour distance by public transportation

B031. How often do you have contact with your parents in person?

[1] Almost every day (more than 4 times per week)
[2] Once a week
[3] 2-3 times a week
[4] Once a month
[5] Twice a month (every two weeks)
[6] Once or twice a year
[7] Three or four times a year (once every three or four months)
[8] Five or six times a year (every two months)
[9] Almost never
[10] never

B032. How often do you have contact with your parents by phone, mail, or e-mail?

[1] Almost every day (more than 4 times per week)
[2] Once a week
[3] 2-3 times a week
[4] Once a month
[5] Twice a month (every two weeks)
[6] Once or twice a year
[7] Three or for times a year (once every three or four months))
[8] Five or six times a year (every two months)
[9] Almost never
[10] never

B033. Not counting any shared housing or shared food, during the LAST CALENDAR YEAR(2005) did you receive any financial help from your parents? If you did, what kind of help did you receive from your parents? Did you receive regular monetary transfer, occasional monetary transfer, or non-monetary transfer? Please choose all that apply.

[1] Yes, received regular monetary transfer
[2] Yes, received occasional monetary transfer → Go to B034 or B041
[3] Yes, received non-monetary transfer → Go to B034 or B041 or B047
[5] No, I did not receive any financial help → Go to B049

SKIP PATTERN CHECKPOINT: REGULAR MONETARY TRANSFER RECEIVED FROM PARENTS IF R RECEIVED REGULAR MONETARY TRANSFER IN B033, GO TO B034.

B034. What was the monthly average amount of regular financial help you received from your parents? (Unit: 10,000 Korean won)

________________________MW (range: 1~9997) → Go to B040

Don't know
Refuse to answer } → Go to B035~B039 unfolding

B035 ~ B039. UNFOLDING BRACKET QUESTIONS

B035. Did it amount to less than, about equal to or more than 10 MW (10,000 Korean won)?

IWER: Go back to B034 and record if R answers the amount during the interview. All figures are in MW, which is 10,000 Korean won.

[1] Less than 10 MW
[3] About 10 MW
[5] More than 10 MW

B036. Did it amount to less than, about equal to or more than 30 MW (10,000 Korean won)?

IWER: Go back to B034 and record if R answers the amount during the interview. All figures are in MW, which is 10,000 Korean won.

[1] Less than 30 MW
[3] About 30 MW
[5] More than 30 MW

B037. Did it amount to less than, about equal to or more than 50 MW (10,000 Korean won)?

IWER: Go back to B034 and record if R answers the amount during the interview. All figures are in MW, which is 10,000 Korean won.

[1] Less than 50 MW
[3] About 50 MW
[5] More than 50 MW

B038. Did it amount to less than, about equal to or more than 100 MW (10,000 Korean won)?

IWER: Go back to B034 and record if R answers the amount during the interview. All figures are in MW, which is 10,000 Korean won.

[1] Less than 100 MW
[3] About 100 MW
[5] More than 100 MW

B039. Did it amount to less than, about equal to or more than 200 MW (10,000 Korean won)?

IWER: Go back to B034 and record if R answers the amount during the interview.

[1] Less than 200 MW
[3] About 200 MW
[5] More than 200 MW

B040. During the PAST CALENDAR YEAR(2005) which months did you receive regular monetary transfers from your parents? Please choose all that apply.

[1] January 2005
[2] February 2005
[3] March 2005
[4] April 2005
[5] May 2005
[6] June 2005
[7] July 2005
[8] August 2005
[9] September 2005
[10] October 2005
[11] November 2005
[12] December 2005
[13] All year round

SKIP PATTERN CHECKPOINT: OCCASIONAL MONETARY TRANSFER RECEIVED FROM PARENTS IF R RECEIVED OCCASIONAL MONETARY TRANSFER IN B033, GO TO B041.

B041. During the LAST CALENDAR YEAR(2005), what was the total amount of occasional monetary transfers you received from your parents? (Unit: 10,000 Korean won)

_________________________MW (range: 1~99997) → Go to B047

Don't know
Refuse to answer } → Go to B042~B046

B042 ~ B046. UNFOLDING BRACKET QUESTIONS

B042. Did it amount to less than, about equal to or more than 100 MW (10,000 Korean won)?

IWER: Go back to B041 and record if R answers the amount during the interview.

[1] Less than 100 MW
[3] About 100 MW
[5] More than 100 MW

B043. Did it amount to less than, about equal to or more than 300 MW (10,000 Korean won)?

IWER: Go back to B041 and record if R answers the amount during the interview.

[1] Less than 300 MW
[3] About 300 MW
[5] More than 300 MW

B044. Did it amount to less than, about equal to or more than 500 MW (10,000 Korean won)?

IWER: Go back to B041 and record if R answers the amount during the interview.

[1] Less than 500 MW
[3] About 500 MW
[5] More than 500 MW

B045. Did it amount to less than, about equal to or more than 1,000 MW (10,000 Korean won)?

IWER: Go back to B041 and record if R answers the amount during the interview.

[1] Less than 1,000 MW
[3] About 1,000 MW
[5] More than 1,000 MW

B046. Did it amount to less than, about equal to or more than 2,000 MW (10,000 Korean won)?

IWER: Go back to B041 and record if R answers the amount during the interview.

[1] Less than 2,000 MW
[3] About 2,000 MW
[5] More than 2,000 MW

SKIP PATTERN CHECKPOINT: NON-MONETARY TRANSFER RECEIVED IF R RECEIVED NON-MONETARY TRANSFER IN B033, GO TO B047.
IF R DID NOT RECEIVE NON-MONETARY TRANSFER, GO TO B049.

B047. What was the type of non-monetary transfers you received from your parents? Please check all that apply

[1] Leisure (e.g., travel)
[2] Health-related products (e.g., vitamins, equipments, etc)
[3] Household items
[4] Electronics
[5] Dining out and food
[6] Other

} → Go to B049

B048. Please specify other.

____

B049. Not counting any shared housing or shared food, during the LAST CALENDAR YEAR(2005) did you give any financial help to your parents?

[1] Yes, made regular monetary transfer
[2] Yes, made occasional monetary transfer → Go to B050 ~ B057
[3] Yes, made non-monetary transfer → Go to B050 ~ B057 or B063
[5] No, I did not give any financial help → Go to B065

SKIP PATTERN CHECKPOINT: REGULAR MONETARY TRANSFER GIVEN TO PARENTS
IF R MADE REGULAR MONETARY TRANSFER IN B049, GO TO B050.
IF R DID NOT MAKE REGULARLY MONETARY TRANSFER, GO TO SKIP PATTERN CHECKPOINT: OCCASIONAL MONETARY TRANSFER GIVEN TO PARENTS.

B050. What was the monthly average amount of regular monetary transfer you gave to your parents? (Unit: 10,000 Korean won)

________________ MW (range: 1~9997) → Go to B056
Don't know
Refuse to answer } → Go to B051~B055 unfolding

B051 ~ B055. UNFOLDING BRACKET QUESTIONS

B051. Did it amount to less than, about equal to or more than 10 MW (10,000 Korean won)?

IWER: Go back to B050 and record if R answers the amount during the interview.

[1] Less than 10 MW
[3] About 10 MW
[5] More than 10 MW

B052. Did it amount to less than, about equal to or more than 30 MW (10,000 Korean won)?

IWER: Go back to B050 and record if R answers the amount during the interview.

[1] Less than 30 MW
[3] About 30 MW
[5] More than 30 MW

B053. Did it amount to less than, about equal to or more than 50 MW (10,000 Korean won)?

IWER: Go back to B050 and record if R answers the amount during the interview.

[1] Less than 50 MW
[3] About 50 MW
[5] More than 50 MW

B054. Did it amount to less than, about equal to or more than 100 MW (10,000 Korean won)?

IWER: Go back to B050 and record if R answers the amount during the interview.

[1] Less than 100 MW
[3] About 100 MW
[5] More than 100 MW

B055. Did it amount to less than, about equal to or more than 200 MW (10,000 Korean won)?

IWER: Go back to B050 and record if R answers the amount during the interview.

[1] Less than 200 MW
[3] About 200 MW
[5] More than 200 MW

B056. Which months did you give regular monetary transfer to your parents during the PAST CALRENDAR YEAR(2005)? Please choose all that apply.

[1] January 2005
[2] February 2005
[3] March 2005
[4] April 2005
[5] May 2005
[6] June 2005
[7] July 2005
[8] August 2005
[9] September 2005
[10] October 2005
[11] November 2005
[12] December 2005
[13] All year round

SKIP PATTERN CHECKPOINT: OCCASIONAL MONETARY TRANSFER GIVEN TO PARENTS

IF R MADE OCCASIONAL MONETARY TRANSFER TO PARENTS IN B049, GO TO B057.

IF R DID NOT MAKE OCCASIONAL MONETARY TRANSFER, GO TO SKIP PATTERN CHECKPOINT: NON-MONETARY TRANSFER TO PARENTS.

B057. During the PAST CALENDAR YEAR(2005), what was the total amount of occasional monetary transfers given to your parents? (Unit: 10,000 Korean won)

__________________MW (range: 1~99997) → Go to B063

Don't know
Refuse to answer } → Go to B058~B062 unfoding

B058 ~ B062. UNFOLDING BRACKET QUESTIONS

B058. Did it amount to less than, about equal to or more than 100 MW (10,000 Korean won)?

IWER: Go back to B057 and record if R answers the amount during the interview.

[1] Less than 100 MW
[3] About 100 MW
[5] More than 100 MW

B059. Did it amount to less than, about equal to or more than 300 MW (10,000 Korean won)?

IWER: Go back to B057 and record if R answers the amount during the interview.

[1] Less than 300 MW
[3] About 300 MW
[5] More than 300 MW

B060. Did it amount to less than, about equal to or more than 500 MW (10,000 Korean won)?

IWER: Go back to B057 and record if R answers the amount during the interview.

[1] Less than 500 MW
[3] About 500 MW
[5] More than 500 MW

B061. Did it amount to less than, about equal to or more than 1,000 MW (10,000 Korean won)?

IWER: Go back to B057 and record if R answers the amount during the interview.

[1] Less than 1,000 MW
[3] About 1,000 MW
[5] More than 1,000 MW

B062. Did it amount to less than, about equal to or more than 2,000 MW (10,000 Korean won)?

IWER: Go back to B057 and record if R answers the amount during the interview.

[1] Less than 2,000 MW
[3] About 2,000 MW
[5] More than 2,000 MW

SKIP PATTERN CHECKPOINT: NON-MONETARY TRANSFER GIVEN TO PARENTS IF R MADE NON-MONETARY TRANSFER TO PARENTS IN B049, GO TO B063. IF R DID NOT MAKE NON-MONETARY TRANSFER, GO TO B065.

B063. What was the type of non-monetary support you gave to your parents? Please choose all that apply.

[1] Leisure (e.g., travel)
[2] Health-related products (e.g., vitamins, equipments, etc)
[3] Household items
[4] Electronics
[5] Dining out and food
[6] Other

} → Go to B065

B064. Please specify other.

B065. Does your father live by himself or with other child (ren) [R’s sibling]?

[1] By himself → Go to B068
[3] With other child (ren)
[5] Other → Go to B067

B066. With whom does he live with? Please list all, if father lives with multiple children.

IWER: Choose all that apply

(Select from the list displayed by CAPI) (Range: 07~26)

B067. Please specify the other.

B068. How close do you live from your father?

[1] Within a 30-minute distance by public transportation
[2] Within a 1-hour distance by public transportation
[3] Within a 2-hour distance by public transportation
[4] More than a 2-hour distance by public transportation

B069. How often do you have contact with your father in person?

[1] Almost every day (more than 4 times per week)
[2] Once a week
[3] 2-3 times a week
[4] Once a month
[5] Twice a month (every two weeks)
[6] Once or twice a year
[7] Three or four times a year (once every three or four months)
[8] Five or six times a year (every two months)
[9] Almost never a year
[10] never

B070. How often do you have contact with your father by phone, mail, or e-mail?

[1] Almost every day (more than 4 times per week)
[2] Once a week
[3] 2-3 times a week
[4] Once a month
[5] Twice a month (every two weeks)
[6] Once or twice a year
[7] Three or four times a year (once every three or four months)
[8] Five or six times a year (every two months)
[9] Almost never a year
[10] Never

B071. Not counting any shared housing or shared food, during the LAST CALENDAR YEAR (2005) did you receive any financial help from your father?

[1] Yes, received regular monetary transfer
[2] Yes, received occasional monetary transfer → Go to B072 or B079
[3] Yes, received non-monetary transfer → Go to B072 or B079 or B085
[5] No, I did not receive any financial help → Go to B087

SKIP PATTERN CHECKPOINT: REGULAR MONETARY TRANSFER RECEIVED FROM FATHER IF R RECEIVED REGULAR MONETARY TRANSFER IN B071, GO TO B072.

B072. What was the monthly average amount of regular financial help you received from your father? (Unit: 10,000 Korean won)

______________________MW (range: 1~9997) → Go to B078

Don't know
Refuse to answer } → Go to B073~B077 unfolding

B073 ~ B077. UNFOLDING BRACKET QUESTIONS

B073. Did it amount to less than, about equal to or more than 10 MW (10,000 Korean won)?

IWER: Go back to B072 and record if R answers the amount during the interview. All figures are in MW, which is 10,000 Korean won.

[1] Less than 10 MW
[3] About 10 MW
[5] More than 10 MW

B074. Did it amount to less than, about equal to or more than 30 MW (10,000 Korean won)?

IWER: Go back to B072 and record if R answers the amount during the interview. All figures are in MW, which is 10,000 Korean won.

[1] Less than 30 MW
[3] About 30 MW
[5] More than 30 MW

B075. Did it amount to less than, about equal to or more than 50 MW (10,000 Korean won)?

IWER: Go back to B072 and record if R answers the amount during the interview. All figures are in MW, which is 10,000 Korean won.

[1] Less than 50 MW
[3] About 50 MW
[5] More than 50 MW

B076. Did it amount to less than, about equal to or more than 100 MW (10,000 Korean won)?

IWER: Go back to B072 and record if R answers the amount during the interview. All figures are in MW, which is 10,000 Korean won.

[1] Less than 100 MW
[3] About 100 MW
[5] More than 100 MW

B077. Did it amount to less than, about equal to or more than 200 MW (10,000 Korean won)?

IWER: Go back to B072 and record if R answers the amount during the interview.

[1] Less than 200 MW
[3] About 200 MW
[5] More than 200 MW

B078. During the PAST CALENDAR YEAR(2005) which months did you receive regular monetary transfers from your father? Please choose all that apply.

[1] January 2005
[2] February 2005
[3] March 2005
[4] April 2005
[5] May 2005
[6] June 2005
[7] July 2005
[8] August 2005
[9] September 2005
[10] October 2005
[11] November 2005
[12] December 2005
[13] All year round

SKIP PATTERN CHECKPOINT: OCCASIONAL MONETARY TRANSFER RECEIVED FROM FATHER IF R RECEIVED OCCASIONAL MONETARY TRANSFER IN B071, GO TO B079.

B079. During the LAST CALENDAR YEAR(2005), what was the total amount of occasional monetary transfers you received from your father? (Unit: 10,000 Korean won)

_____________________MW (range: 1~99997) → Go to B085

Don't know
Reuse to answer } → Go to B080~B084 unfolding

B080 ~ B084. UNFOLDING BRACKET QUESTIONS

B080. Did it amount to less than, about equal to or more than 100 MW (10,000 Korean won)?

IWER: Go back to B079 and record if R answers the amount during the interview.

[1] Less than 100 MW
[3] About 100 MW
[5] More than 100 MW

B081. Did it amount to less than, about equal to or more than 300 MW (10,000 Korean won)?

IWER: Go back to B079 and record if R answers the amount during the interview.

[1] Less than 300 MW
[3] About 300 MW
[5] More than 300 MW

B082. Did it amount to less than, about equal to or more than 500 MW (10,000 Korean won)?

IWER: Go back to B079 and record if R answers the amount during the interview.

[1] Less than 500 MW
[3] About 500 MW
[5] More than 500 MW

B083. Did it amount to less than, about equal to or more than 1,000 MW (10,000 Korean won)?

IWER: Go back to B079 and record if R answers the amount during the interview.

[1] Less than 1,000 MW
[3] About 1,000 MW
[5] More than 1,000 MW

B084. Did it amount to less than, about equal to or more than 2,000 MW (10,000 Korean won)?

IWER: Go back to B079 and record if R answers the amount during the interview.

[1] Less than 2,000 MW
[3] About 2,000 MW
[5] More than 2,000 MW

SKIP PATTERN CHECKPOINT: NON-MONETARY TRANSFER RECEIVED FROM FATHER IF R RECEIVED NON–MONETARY TRANSFER IN B071, GO TO B085.

B085. What was the type of non-monetary transfers you received from your father? Please check all that apply

[1] Leisure (e.g., travel)
[2] Health-related products (e.g., vitamins, equipments, etc)
[3] Household items
[4] Electronics
[5] Dining out and food
→ Go to B087 (for [1]–[5])
[6] Other

B086. Please specify other.

B087. Not counting any shared housing or shared food, during the LAST CALENDAR YEAR(2005) did you give any financial help to your father?

[1] Yes, made regular monetary transfer
[2] Yes, made occasional monetary transfer → Go to B088 or B095
[3] Yes, made non-monetary transfer → Go to B088 or B095 or B101
[5] No, I did not give any financial help → Go to B103

SKIP PATTERN CHECKPOINT: REGULAR MONETARY TRANSFER GIVEN TO FATHER IF R MADE REGULAR MONETARY TRANSFER TO FATHER IN B087, GO TO B088.

B088. What was the monthly average amount of regular monetary transfer you gave to your father? (Unit: 10,000 Korean won)

______________________MW (range: 1~9997) → Go to B094

Don't know
Refuse to answer
→ Go to B089~B093 unfolding

B089 ~ B093. UNFOLDING BRACKET QUESTIONS

B089. Did it amount to less than, about equal to or more than 10 MW (10,000 Korean won)?

IWER: Go back to B088 and record if R answers the amount during the interview.

[1] Less than 10 MW
[3] About 10 MW
[5] More than 10 MW

B090. Did it amount to less than, about equal to or more than 30 MW (10,000 Korean won)?

IWER: Go back to B088 and record if R answers the amount during the interview.

[1] Less than 30 MW
[3] About 30 MW
[5] More than 30 MW

B091. Did it amount to less than, about equal to or more than 50 MW (10,000 Korean won)?

IWER: Go back to B088 and record if R answers the amount during the interview.

[1] Less than 50 MW
[3] About 50 MW
[5] More than 50 MW

B092. Did it amount to less than, about equal to or more than 100 MW (10,000 Korean won)?

IWER: Go back to B088 and record if R answers the amount during the interview.

[1] Less than 100 MW
[3] About 100 MW
[5] More than 100 MW

B093. Did it amount to less than, about equal to or more than 200 MW (10,000 Korean won)?

IWER: Go back to B088 and record if R answers the amount during the interview.

[1] Less than 200 MW
[3] About 200 MW
[5] More than 200 MW

B094. Which months did you give regular monetary transfer to your father during the PAST CALRENDAR YEAR(2005)? Please choose all that apply.

[1] January 2005
[2] February 2005
[3] March 2005
[4] April 2005
[5] May 2005
[6] June 2005
[7] July 2005
[8] August 2005
[9] September 2005
[10] October 2005
[11] November 2005
[12] December 2005
[13] All year round

SKIP PATTERN CHECKPOINT: OCCASIONAL MONETARY TRANSFER GIVEN TO FATHER IF R MADE OCCASIONAL MONETARY TRANSFER TO FATHER IN B087, GO TO B095.

B095. During the PAST [CALENDAR] YEAR, what was the total amount of occasional monetary transfers given to your father? (Unit: 10,000 Korean won)

_____________________MW (range: 1~99997) → Go to B101

Don't know
Refuse to answer } → Go to B096~B100 unfolding

B096 ~ B100. UNFOLDING BRACKET QUESTIONS

B096. Did it amount to less than, about equal to or more than 100 MW (10,000 Korean won)?

IWER: Go back to B095 and record if R answers the amount during the interview. All figures are in MW, which is 10,000 Korean won.

[1] Less than 100 MW
[3] About 100 MW
[5] More than 100 MW

B097. Did it amount to less than, about equal to or more than 300 MW (10,000 Korean won)?

IWER: Go back to B095 and record if R answers the amount during the interview. All figures are in MW, which is 10,000 Korean won.

[1] Less than 300 MW
[3] About 300 MW
[5] More than 300 MW

B098. Did it amount to less than, about equal to or more than 500 MW (10,000 Korean won)?

IWER: Go back to B095 and record if R answers the amount during the interview. All figures are in MW, which is 10,000 Korean won.

[1] Less than 500 MW
[3] About 500 MW
[5] More than 500 MW

B099. Did it amount to less than, about equal to or more than 1,000 MW (10,000 Korean won)?

IWER: Go back to B095 and record if R answers the amount during the interview. All figures are in MW, which is 10,000 Korean won.

[1] Less than 1,000 MW
[3] About 1,000 MW
[5] More than 1,000 MW

B100. Did it amount to less than, about equal to or more than 2,000 MW (10,000 Korean won)?

IWER: Go back to B095 and record if R answers the amount during the interview.
All figures are in MW, which is 10,000 Korean won.

[1] Less than 2,000 MW
[3] About 2,000 MW
[5] More than 2,000 MW

SKIP PATTERN CHECKPOINT: NON-MONETARY TRANSFER GIVEN TO FATHER IF R MADE NON-MONETARY TRANSFER TO FATHER IN B087, GO TO B101.

B101. What was the type of non-monetary support you gave to your father? Please choose all that apply.

[1] Leisure (e.g., travel)
[2] Health-related products (e.g., vitamins, equipments, etc)
[3] Household items
[4] Electronics
[5] Dining out and food
[6] Other

(Options 1–5) → Go to B103

B102. Please specify other.

B103. Does your mother live by herself or with other child(ren) [R's sibling]?

[1] By herself → Go to B106
[3] With other child (ren)
[5] Other → Go to B105

B104. With whom does she live with? Please list all, if mother lives with multiple children.

IWER: Choose all that apply

B105. Please specify the other.

B106. How close do you live from your mother?

[1] Within a 30-minute distance by public transportation
[2] Within a 1-hour distance by public transportation
[3] Within a 2-hour distance by public transportation
[4] More than a 2-hour distance by public transportation

B107. How often do you have contact with your mother in person?

[1] Almost every day (more than 4 times per week)
[2] Once a week
[3] 2-3 times a week
[4] Once a month
[5] Twice a month (every two weeks)
[6] Once or twice a year
[7] Three or four times a year (once every three or four months)
[8] Five or six times a year (every two months)
[9] Almost never a year
[10] never

B108. How often do you have contact with your mother by phone, mail, or e-mail?

[1] Almost every day (more than 4 times per week)
[2] Once a week
[3] 2-3 times a week
[4] Once a month
[5] Twice a month (every two weeks)
[6] Once or twice a year
[7] Three or four times a year (once every three or four months)
[8] Five or six times a year (every two months)
[9] Almost never a year
[10] Never

B109. Not counting any shared housing or shared food, during the LAST CALENDAR YEAR(2005) did you receive any financial help from your mother? If you did, what kind of help did you receive from your mother? Did you receive regular monetary transfer, occasional monetary transfer, or non-monetary transfer? Please choose all that apply.

[1] Yes, received regular monetary transfer
[2] Yes, received occasional monetary transfer → Go to B110 or B117
[3] Yes, received non-monetary transfer → Go to B110 or B117 or B123
[5] No, I did not receive any financial help → Go to B125

SKIP PATTERN CHECKPOINT: REGULAR MONETARY TRANSFER RECEIVED FROM MOTHER IF R RECEIVED REGULAR MONETARY TRANSFER IN B109, GO TO B110.

B110. What was the monthly average amount of regular financial help you received from your mother? (Unit: 10,000 Korean won)

______________________MW (range: 1~9997) → Go to B116

Don't know
Refuse to answer } → Go to B111~B115 unfolding

B111 ~ B115. UNFOLDING BRACKET QUESTIONS

B111. Did it amount to less than, about equal to or more than 10 MW (10,000 Korean won)?

IWER: Go back to B110 and record if R answers the amount during the interview. All figures are in MW, which is 10,000 Korean won.

[1] Less than 10 MW
[3] About 10 MW
[5] More than 10 MW

B112. Did it amount to less than, about equal to or more than 30 MW (10,000 Korean won)?

IWER: Go back to B110 and record if R answers the amount during the interview. All figures are in MW, which is 10,000 Korean won.

[1] Less than 30 MW
[3] About 30 MW
[5] More than 30 MW

B113. Did it amount to less than, about equal to or more than 50 MW (10,000 Korean won)?

IWER: Go back to B110 and record if R answers the amount during the interview. All figures are in MW, which is 10,000 Korean won.

[1] Less than 50 MW
[3] About 50 MW
[5] More than 50 MW

B114. Did it amount to less than, about equal to or more than 100 MW (10,000 Korean won)?

IWER: Go back to B110 and record if R answers the amount during the interview. All figures are in MW, which is 10,000 Korean won.

[1] Less than 100 MW
[3] About 100 MW
[5] More than 100 MW

B115. Did it amount to less than, about equal to or more than 200 MW (10,000 Korean won)?

IWER: Go back to B110 and record if R answers the amount during the interview.
All figures are in MW, which is 10,000 Korean won.

[1] Less than 200 MW
[3] About 200 MW
[5] More than 200 MW

B116. During the PAST CALENDAR YEAR(2005) which months did you receive regular monetary transfers from your mother? Please choose all that apply.

[1] January 2005
[2] February 2005
[3] March 2005
[4] April 2005
[5] May 2005
[6] June 2005
[7] July 2005
[8] August 2005
[9] September 2005
[10] October 2005
[11] November 2005
[12] December 2005
[13] All year round

SKIP PATTERN CHECKPOINT: OCCASIONAL MONETARY TRANSFER RECEIVED FROM MOTHER IF R RECEIVED OCCASIONAL MONETARY TRANSFER IN B109, GO TO B117. IF R DID NOT RECEIVE OCCASIONAL MONETARY TRANSFER, GO TO SKIP PATTERN CHECKPOINT: NON-MONETARY TRANSFER FROM MOTHER.

B117. During the LAST CALENDAR YEAR, what was the total amount of occasional monetary transfers you received from your mother?

______________________MW (10,000 Korean won) → Go to B123

Don't know
Refuse to answer } → Go to B118~B122 unfolding

B118 ~ B122. UNFOLDING BRACKET QUESTIONS

B118. Did it amount to less than, about equal to or more than 100 MW (10,000 Korean won)?

IWER: Go back to B117 and record if R answers the amount during the interview.
All figures are in MW, which is 10,000 Korean won.

[1] Less than 100 MW
[3] About 100 MW
[5] More than 100 MW

B119. Did it amount to less than, about equal to or more than 300 MW (10,000 Korean won)?

IWER: Go back to B117 and record if R answers the amount during the interview. All figures are in MW, which is 10,000 Korean won.

[1] Less than 300 MW
[3] About 300 MW
[5] More than 300 MW

B120. Did it amount to less than, about equal to or more than 500 MW (10,000 Korean won)?

IWER: Go back to B117 and record if R answers the amount during the interview. All figures are in MW, which is 10,000 Korean won.

[1] Less than 500 MW
[3] About 500 MW
[5] More than 500 MW

B121. Did it amount to less than, about equal to or more than 1,000 MW (10,000 Korean won)?

IWER: Go back to B117 and record if R answers the amount during the interview. All figures are in MW, which is 10,000 Korean won.

[1] Less than 1,000 MW
[3] About 1,000 MW
[5] More than 1,000 MW

B122. Did it amount to less than, about equal to or more than 2,000 MW (10,000 Korean won)?

IWER: Go back to B117 and record if R answers the amount during the interview. All figures are in MW, which is 10,000 Korean won.

[1] Less than 2,000 MW
[3] About 2,000 MW
[5] More than 2,000 MW

SKIP PATTERN CHECKPOINT: NON－MONETARY TRANSFER RECEIVED FROM MOTHER
IF R RECEIVED NON-MONETARY TRANSFER IN B109, GO TO B123.
IF R DID NOT RECEIVE NON-MONETARY TRANSFER, GO TO B125.

B123. What was the type of non-monetary transfers you received from your parents? Please check all that apply.

[1] Leisure (e.g., travel)
[2] Health-related products (e.g., vitamins, equipments, etc)
[3] Household items
[4] Electronics
[5] Dining out and food
[6] Other

} → Go to B125

B124. Please specify other.

B125. Not counting any shared housing or shared food, during the LAST CALENDAR YEAR(2005) did you give any financial help to your mother?

[1] Yes, made regular monetary transfer
[2] Yes, made occasional monetary transfer → Go to B126 or B133
[3] Yes, made non-monetary transfer → Go to B126 or B133 or B1
[5] No, I did not give any financial help → Go to B141

SKIP PATTERN CHECKPOINT: REGULAR MONETARY TRANSFER GIVEN TO MOTHER
IF R MADE REGULAR MONETARY TRANSFER TO MOTHER IN B125, GO TO B126.
IF R DID NOT MAKE REGULAR MONETARY TRANSFER, GO TO SKIP PATTERN CHECKPOINT: OCCASIONAL MONETARY TRANSFER GIVEN TO MOTHER.

B126. What was the monthly average amount of regular monetary transfer you gave to your mother? (Unit: 10,000 Korean won)

______________________MW (range: 1~9997) → Go to B132

Don't know
Refuse to answer } → Go to B127~b131 unfolding

B127 ~ B131. UNFOLDING BRACKET QUESTIONS

B127. Did it amount to less than, about equal to or more than 10 MW (10,000 Korean won)?

IWER: Go back to B126 and record if R answers the amount during the interview.

[1] Less than 10 MW
[3] About 10 MW
[5] More than 10 MW

B128. Did it amount to less than, about equal to or more than 30 MW (10,000 Korean won)?

IWER: Go back to B126 and record if R answers the amount during the interview.

[1] Less than 30 MW
[3] About 30 MW
[5] More than 30 MW

B129. Did it amount to less than, about equal to or more than 50 MW (10,000 Korean won)?

IWER: Go back to B126 and record if R answers the amount during the interview.

[1] Less than 50 MW
[3] About 50 MW
[5] More than 50 MW

B130. Did it amount to less than, about equal to or more than 100 MW (10,000 Korean won)?

IWER: Go back to B126 and record if R answers the amount during the interview.

[1] Less than 100 MW
[3] About 100 MW
[5] More than 100 MW

B131. Did it amount to less than, about equal to or more than 200 MW (10,000 Korean won)?

IWER: Go back to B126 and record if R answers the amount during the interview.

[1] Less than 200 MW
[3] About 200 MW
[5] More than 200 MW

B132. Which months did you give regular monetary transfer to your mother during the PAST CALRENDAR YEAR(2005)? Please check all that apply.

[1] January 2005
[2] February 2005
[3] March 2005
[4] April 2005
[5] May 2005
[6] June 2005
[7] July 2005
[8] August 2005
[9] September 2005
[10] October 2005
[11] November 2005
[12] December 2005
[13] All year round

SKIP PATTERN CHECKPOINT: OCCASIONAL MONETARY TRANSFER GIVEN TO MOTHER
IF R MADE OCCASIONAL MONETARY TRANSFER TO MOTHER IN B125, GO TO B133.
IF R DID NOT MAKE OCCASIONAL MONETARY TRANSFER, GO TO SKIP PATTERN.

B133. During the PAST CALENDAR YEAR(2005), what was the total amount of occasional monetary transfers given to your mother? (Unit: 10,000 Korean won)

____________________MW (range: 1~99997) → Go to B139

Don't know
Refuse to answer } → Go to B134~B138 unfolding

B134 ~ B138. UNFOLDING BRACKET QUESTIONS

B134. Did it amount to less than, about equal to or more than 100 MW (10,000 Korean won)?

IWER: Go back to B133 and record if R answers the amount during the interview.

[1] Less than 100 MW
[3] About 100 MW
[5] More than 100 MW

B135. Did it amount to less than, about equal to or more than 300 MW (10,000 Korean won)?

IWER: Go back to B133 and record if R answers the amount during the interview.

[1] Less than 300 MW
[3] About 300 MW
[5] More than 300 MW

B136. Did it amount to less than, about equal to or more than 500 MW (10,000 Korean won)?

IWER: Go back to B133 and record if R answers the amount during the interview.

[1] Less than 500 MW
[3] About 500 MW
[5] More than 500 MW

B137. Did it amount to less than, about equal to or more than 1,000 MW (10,000 Korean won)?

IWER: Go back to B133 and record if R answers the amount during the interview.

[1] Less than 1,000 MW
[3] About 1,000 MW
[5] More than 1,000 MW

B138. Did it amount to less than, about equal to or more than 2,000 MW (10,000 Korean won)?

IWER: Go back to B133 and record if R answers the amount during the interview.

[1] Less than 2,000 MW
[3] About 2,000 MW
[5] More than 2,000 MW

SKIP PATTERN CHECKPOINT: NON−MONETARY TRANSFER GIVEN TO MOTHER IF R MADE NON−MONETARY TRANSFER TO MOTHER IN B125, GO TO B139. IF R DID NOT MAKE NON-MONETARY TRANSFER, GO TO B141.

B139. What was the type of non-monetary support you gave to your mother? Please choose all that apply.

[1] Leisure (e.g., travel)
[2] Health-related products (e.g., vitamins, equipments, etc)
[3] Household items
[4] Electronics
[5] Dining out and food
[6] Other

(items [1]–[6]) → Go to B141

B140. Please specify other.

B141. Not counting any shared housing or shared food, during the LAST CALENDAR YEAR (2005) did you receive any financial help from any family members who are not co-residing with you (e.g., parents-in-law, siblings, brother/sister-in-law, grandchildren, etc)?

IWER: By financial help we mean giving money, helping pay bills, or covering specific types of costs such as those for medical care or insurance, schooling, down payment for a home, rent, etc., but not counting any shared housing or shared food. The financial help can be considered support, a gift or a loan.

[1] Yes, received financial transfer
[5] No, I did not receive any financial help → Go to B160

B142. From whom did you receive financial help? Please identify all transfer givers. (Select from the list displayed by CAPI)

[5] Mother-in-law
[6] Father-in-law
[27 ~ 46] Siblings list
[47] Bother/sister-in-law
[48] Son/daughter-in-law
[49] Grandchildren 50 Other relatives

} → Go to B144

[50] other relateves

B143. Please specify other.

LOOP CHECKPOINT: TRANSFER GIVER REPEAT TRANSFER QUESTIONS, B144 ~ B159, FOR EACH FINANCIAL GIVER.

B144. What kind of financial help did you receive from [GIVER'S NAME CHOSEN FROM B142]? Did you receive regular monetary transfer, occasional monetary transfer, or non-monetary transfer? Please choose all that apply.

[1] regular monetary transfer
[2] occasional monetary transfer → Go to B145 or B152
[3] non-monetary transfer → Go to B145 or B152 or B158

SKIP PATTERN CHECKPOINT: REGULAR MONETARY TRANSFER RECEIVED FROM OTHER FAMILY MEMBER IF R RECEIVED REGULAR MONETARY TRANSFER IN B144, GO TO B145.
IF R DID NOT RECEIVE REGULAR MONETARY TRANSFER, GO TO SKIP PATTERN.

B145. What was the monthly average amount of regular financial help you received from [GIVER'S NAME CHOSEN FROM B142]? (Unit: 10,000 Korean won)

_____________________MW (range: 1~9997) → Go to B151

Don't know
Refuse to answer } → Go to B146~B150 unfolding

B146 ~ B150. UNFOLDING BRACKET QUESTIONS

B146. Did it amount to less than, about equal to or more than 10 MW (10,000 Korean won)?

IWER: Go back to B145 and record if R answers the amount during the interview. All figures are in MW, which is 10,000 Korean won.

[1] Less than 10 MW
[3] About 10 MW
[5] More than 10 MW

B147. Did it amount to less than, about equal to or more than 30 MW (10,000 Korean won)?

IWER: Go back to B145 and record if R answers the amount during the interview. All figures are in MW, which is 10,000 Korean won.

[1] Less than 30 MW
[3] About 30 MW
[5] More than 30 MW

B148. Did it amount to less than, about equal to or more than 50 MW (10,000 Korean won)?

IWER: Go back to B145 and record if R answers the amount during the interview. All figures are in MW, which is 10,000 Korean won.

[1] Less than 50 MW
[3] About 50 MW
[5] More than 50 MW

B149. Did it amount to less than, about equal to or more than 100 MW (10,000 Korean won)?

IWER: Go back to B145 and record if R answers the amount during the interview. All figures are in MW, which is 10,000 Korean won.

[1] Less than 100 MW
[3] About 100 MW
[5] More than 100 MW

B150. Did it amount to less than, about equal to or more than 200 MW (10,000 Korean won)?

IWER: Go back to B145 and record if R answers the amount during the interview. All figures are in MW, which is 10,000 Korean won.

[1] Less than 200 MW
[3] About 200 MW
[5] More than 200 MW

B151. During the PAST CALENDAR YEAR(2005) which months did you receive regular monetary transfers? Please choose all that apply.

[1] January 2005
[2] February 2005
[3] March 2005
[4] April 2005
[5] May 2005
[6] June 2005
[7] July 2005
[8] August 2005
[9] September 2005
[10] October 2005
[11] November 2005
[12] December 2005
[13] All year round

SKIP PATTERN CHECKPOINT: OCCASIONAL MONETARY TRANSFER RECEIVED FROM OTHER FAMILY MEMBER IF R RECEIVED OCCASIONAL MONETARY TRANSFER IN B144, GO TO B152.
IF R DID NOT RECEIVE OCCASIONAL MONETARY TRANSFER, GO TO SKIP PATTERN CHECKPOINT: NON-MONETARY TRANSFER FROM OTHER FAMILY MEMBER.

B152. During the LAST CALENDAR YEAR(2005), what was the total amount of occasional monetary transfers you received from GIVER'S NAME CHOSEN FROM B142? (Unit: 10,000 Korean won)

______________________MW (range: 1~99997) → Go to B158

Don't know
Refuse to answer } → Go to B153~B157 unfolding

B153 ~ B157. UNFOLDING BRACKET QUESTIONS

B153. Did it amount to less than, about equal to or more than 100 MW (10,000 Korean won)?

IWER: Go back to B152 and record if R answers the amount during the interview.
All figures are in MW, which is 10,000 Korean won.

[1] Less than 100 MW
[3] About 100 MW
[5] More than 100 MW

B154. Did it amount to less than, about equal to or more than 300 MW (10,000 Korean won)?

IWER: Go back to B152 and record if R answers the amount during the interview.
All figures are in MW, which is 10,000 Korean won.

[1] Less than 300 MW
[3] About 300 MW
[5] More than 300 MW

B155. Did it amount to less than, about equal to or more than 500 MW (10,000 Korean won)?

IWER: Go back to B152 and record if R answers the amount during the interview.
All figures are in MW, which is 10,000 Korean won.

[1] Less than 500 MW
[3] About 500 MW
[5] More than 500 MW

B156. Did it amount to less than, about equal to or more than 1,000 MW (10,000 Korean won)?

IWER: Go back to B152 and record if R answers the amount during the interview.
All figures are in MW, which is 10,000 Korean won.

[1] Less than 1,000 MW
[3] About 1,000 MW
[5] More than 1,000 MW

B157. Did it amount to less than, about equal to or more than 2,000 MW (10,000 Korean won)?

IWER: Go back to B152 and record if R answers the amount during the interview.
All figures are in MW, which is 10,000 Korean won.

[1] Less than 2,000 MW
[3] About 2,000 MW
[5] More than 2,000 MW

SKIP PATTERN CHECKPOINT: NON–MONETARY TRANSFER RECEIVED FROM OTHER FAMILY MEMBER IF R RECEIVED NON-MONETARY TRANSFER IN B144, GO TO B158.
IF R DID NOT RECEIVE NON-MONETARY TRANSFER, GO TO B160.

B158. What was the type of non-monetary transfers you received from GIVER'S NAME CHOSEN FROM B142? Please check all that apply.

[1] Leisure (e.g., travel)
[2] Health-related products (e.g., vitamins, equipments, etc)
[3] Household items
[4] Electronics
[5] Dining out and food
[6] Other

(items 1–5) → Go to B160

B159. Please specify other.

B160. Not counting any shared housing or shared food, during the LAST CALENDAR YEAR (2005) did you give any financial help to any family members who are not co-residing with you (e.g., parents-in-law, siblings, brother/sister-in-law, grandchildren, etc)

[1] Yes, I gave financial help.
[5] No, I did not give any financial help. → Go to B179

B161. To whom did you give financial help? Please identify all transfer receivers. (Select from the list displayed by CAPI)

[5] Mother-in-law
[6] Father-in-law
[27~46] Siblings list
[47] Bother / sister-in-law
[48] Son / daughter-in-law
[49] Grandchildren
[50] Other relatives

(items 5–49) → Go to B163B163

B162. Please specify other.

LOOP CHECKPOINT : TRANSFER RECEIVER REPEAT TRANSFER QUESTIONS, B163 ~ B178, FOR EACH FINANCIAL RECIEVER.

B163. What kind of financial help did you give to RECEIVER'S NAME CHOSEN FROM B161? Did you give regular monetary transfer, occasional monetary transfer, or non-monetary transfer? Please choose all that apply.

[1] regular monetary transfer
[2] occasional monetary transfer → Go To B164 or B171
[3] non-monetary transfer → Go to B164 or B171 or B177

SKIP PATTERN CHECKPOINT : REGULAR MONETARY TRANSFER GIVEN TO OTHER FAMILY MEMBER IF R MADE REGULAR MONETARY TRANSFER IN B163, GO TO B164.
IF R DID NOT MAKE REGULAR MONETARY TRANSFER, GO TO SKIP PATTERN.

B164. What was the monthly average amount of regular financial help you gave to RECEIVER'S NAME CHOSEN FROM B161? (Unit: 10,000 Korean won)

______________________ MW (range: 1~9997) → Go to B170

Don't know
Refuse to answer } → Go to B165~B169 unfolding

B165 ~ B169. UNFOLDING BRACKET QUESTIONS

B165. Did it amount to less than, about equal to or more than 10 MW (10,000 Korean won)?

IWER: Go back to B164 and record if R answers the amount during the interview. All figures are in MW, which is 10,000 Korean won.

[1] Less than 10 MW
[3] About 10 MW
[5] More than 10 MW

B166. Did it amount to less than, about equal to or more than 30 MW (10,000 Korean won)?

IWER: Go back to B164 and record if R answers the amount during the interview. All figures are in MW, which is 10,000 Korean won.

[1] Less than 30 MW
[3] About 30 MW
[5] More than 30 MW

B167. Did it amount to less than, about equal to or more than 50 MW (10,000 Korean won)?

IWER: Go back to B164 and record if R answers the amount during the interview. All figures are in MW, which is 10,000 Korean won.

[1] Less than 50 MW
[3] About 50 MW
[5] More than 50 MW

B168. Did it amount to less than, about equal to or more than 100 MW (10,000 Korean won)?

IWER: Go back to B164 and record if R answers the amount during the interview. All figures are in MW, which is 10,000 Korean won.

[1] Less than 100 MW
[3] About 100 MW
[5] More than 100 MW

B169. Did it amount to less than, about equal to or more than 200 MW (10,000 Korean won)?

IWER: Go back to B164 and record if R answers the amount during the interview. All figures are in MW, which is 10,000 Korean won.

[1] Less than 200 MW
[3] About 200 MW
[5] More than 200 MW

B170. During the PAST CALENDAR YEAR(2005) which months did you give regular monetary transfers? Please choose all that apply.

[1] January 2005
[2] February 2005
[3] March 2005
[4] April 2005
[5] May 2005
[6] June 2005
[7] July 2005
[8] August 2005
[9] September 2005
[10] October 2005
[11] November 2005
[12] December 2005
[13] All year round

SKIP PATTERN CHECKPOINT : OCCASIONAL MONETARY TRANSFER GIVEN TO OTHER FAMILY MEMBER IF R MADE OCCASIONAL MONETARY TRANSFER IN B163, GO TO B171.
IF R DID NOT MAKE OCCASIONAL MONETARY TRANSFER, GO TO SKIP PATTERN.

B171. During the LAST CALENDAR YEAR, what was the total amount of occasional monetary transfers you give to RECEIVER'S NAME CHOSEN FROM B161? (Unit: 10,000 Korean won)

_____________________ MW

B172 ~ B176. UNFOLDING BRACKET QUESTIONS

B172. Did it amount to less than, about equal to or more than 100 MW (10,000 Korean won)?

IWER: Go back to B171 and record if R answers the amount during the interview.
All figures are in MW, which is 10,000 Korean won.

[1] Less than 100 MW
[3] About 100 MW
[5] More than 100 MW

B173. Did it amount to less than, about equal to or more than 300 MW (10,000 Korean won)?

IWER: Go back to B171 and record if R answers the amount during the interview.
All figures are in MW, which is 10,000 Korean won.

[1] Less than 300 MW
[3] About 300 MW
[5] More than 300 MW

B174. Did it amount to less than, about equal to or more than 500 MW (10,000 Korean won)?

IWER: Go back to B171 and record if R answers the amount during the interview.
All figures are in MW, which is 10,000 Korean won.

[1] Less than 500 MW
[3] About 500 MW
[5] More than 500 MW

B175. Did it amount to less than, about equal to or more than 1,000 MW (10,000 Korean won)?

IWER: Go back to B171 and record if R answers the amount during the interview.
All figures are in MW, which is 10,000 Korean won.

[1] Less than 1,000 MW
[3] About 1,000 MW
[5] More than 1,000 MW

B176. Did it amount to less than, about equal to or more than 2,000 MW (10,000 Korean won)?

IWER: Go back to B171 and record if R answers the amount during the interview.
All figures are in MW, which is 10,000 Korean won.

[1] Less than 2,000 MW
[3] About 2,000 MW
[5] More than 2,000 MW

SKIP PATTERN CHECKPOINT: NON-MONETARY TRANSFER GIVEN TO OTHER FAMILY MEMBER IF R MADE NON-MONETARY TRANSFER IN B163, GO TO B177. IF R DID NOT MAKE NON-MONETARY TRANSFER, GO TO B179.

B177. What was the type of non-monetary transfers you given to RECEIVER'S NAME CHOSEN FROM B161? Please check all that apply

[1] Leisure (e.g., travel)
[2] Health-related products (e.g., vitamins, equipments, etc)
[3] Household items
[4] Electronics
[5] Dining out and food
→ Go to B179 (for [1]–[5])
[6] Other

B178. Please specify other.

----

* ADL / IADL *

B179. Are there any members of your family over the age 10 (spouse, parents, parents of spouse, siblings and/or children) who are unable to carry out activities of daily living (ADL)? Activities of daily living refer to everyday routines such as eating, dressing, bathing or using the toilet, etc. Please identify all members of family with ADL difficulties.
(Select from the list displayed by CAPI)

[2] Spouse
[3] Mother
[4] Father
[5] Mother-in-law
[6] Father-in-law
[7 ~ 26] Children
[27 ~ 46] Sibling
[47] 배우자의 형제자매
[48] 자녀의 배우자
[49] 손자녀
[50] 기타 다른 친인척
[52] 일상생활수행능력 어려운 사람 없음 → B183

B180. Did you provide (names listed in B179) any help with activities of daily living during the past 12 months (not calendar year)? If so, who was helped?
(Select from the list displayed by CAPI)

[2] Spouse
[3] Mother
[4] Father
[5] Mother-in-law
[6] Father-in-law
[7 ~ 26] Children
[27 ~ 46] Sibling
[47] Siblings of spouse
[48] Spouses of children
[49] Grandchildren
[50] Other relatives
[53] Did not help (They were helped by other helpers, etc.) → Go to B183

LOOP CHECKPOINT : ADL CARE RECEIVER REPEAT TRANSFER QUESTIONS, B181 ~ B182, FOR EACH PERSON R PROVIDED CARE.

B181. During the past 12 months (not calendar year), roughly how many hours per week did you help out name chosen from B180?

___________hours per week (range: 1~168)

B182. How many weeks did you provide such care to name chosen from B180 during the past 12 months?

IWER: Calculate 1 month as 4 weeks, 6 months as 26 weeks, 1 year as 52 weeks

___________weeks (range: 1~52)

B183. Did you help your any of your family members (spouse, parents, parents of spouse, siblings and/or children) who are not living with you with other things such as household chores, errands, transportation, grocery shopping, financial management, etc.? If you did, who was helped? Please identify all family members whom you helped out during the past 12 months.
(Select from the list displayed by CAPI)

[3] Mother
[1] Father
[2] Mother-in-law
[3] Father-in-law
[4 ~ 26] Children
[27 ~ 46] Sibling
[47] Siblings of spouse
[48] Spouses of children
[49] Grandchildren
[50] Other relatives
[54] None → Go to B186

LOOP CHECKPOINT: IADL CARE RECEIVER REPEAT TRANSFER QUESTIONS, B184 ~ B185, FOR EACH PERSON R PROVIDED CARE IN B183.

B184. During the past 12 months (not calendar year), roughly how many hours per week did you help out name chosen from B180?

___________hours per week (range: 1~168)

B185. How many weeks did you provide such care to name chosen from B180 during the past 12 months?

IWER: Calculate 1 month as 4 weeks, 6 months as 26 weeks, 1 year as 52 weeks.

___________weeks (range: 1~52)

B186. IWER: HOW OFTEN DID R RECEIVE ASSISTANCE WITH ANSWERS IN SECTION Bb – FAMILY (PARENTS & SIBLINGS) AND FAMILY TRANSFER?

[1] Never
[2] A few times
[3] Most or all of time
→ Go to Section Ca.
[4] The Section was done by a proxy reporter.

B190. IWER: WHAT IS THE PROXY'S RELATIONSHIP TO R? IF UNKNOWN, PLEASE ASK THE PROXY. What is your relationship to R?

[1] Spouse
[2] Mother
[3] Father
[4] Mother-in-law
[5] Father-in-law
[6] Sibling
[7] Brother-in-law, sister-in-law
[8] Child
[9] Spouse of child
[10] Grandchild
[11] Other relative
[12] Helper or other non-relative
→ Go to Section Ca.

C. HEALTH

Ca. HEALTH STATUS

Ca. HEALTH STATUS SURVEY STRUCTURE

C001, C142 : SUBJECTIVE HEALTH STATUS

C002-C003 : DISABILITY DIAGNOSIS FROM A DOCTOR

C004 : LIMITATION OF DAILY ACTIVITY(WORK) FOR HEALTH PROBLEM

C005-C042 : CHRONICAL ILLNESS - WHETHER OR NOT DIAGNOSE ILLNESS, DATE OF DIAGNOSIS, TAKING ANY MEDICATION AND TREATMENT, LIMITATION OF DAILY ACTIVITY

- C005-C008 : HIGH BLOOD PRESSURE
- C009-C012 : DIABETES
- C013-C018 : CANCER(MALIGNANT TUMOR) - PART OF OCCURING CANDER
- C019-C022 : LUNG DISEASE
- C023-C026 : LIVER DISEASE
- C027-C030 : HEART ILLNESS
- C031-C034 : STROKE
- C035-C038 : PSYCHIC PROBLEM
- C039-C042 : ARTHRITIS OR RHEUMATISM

C043-C052 : ACCIDENT/FALLING/FRACTURE

- C043-C045 : TRAFFIC ACCIDENT
- C046-C052 : FALL AND FRACTURE

C053-C061 : PROSTATE ILLNESS - WHETHER OR NOT DIAGNOSE ILLNESS, DATE OF DIAGNOSIS, TAKING ANY MEDICATION AND TREATMENT, LIMITATION OF DAILY ACTIVITY

Ca. HEALTH STATUS SURVEY STRUCTURE (Continued)

C053-C056 : PROSTATE ILLNESS(MEN ONLY)
C057-C061 : URINE PROBLEM(WOMEN ONLY)

C062-C074 : EYESIGHT /HEARING/DENTAL HEALTH

C062-C069 : EYESIGHT PROBLEM
C070-C072 : HEARING PROBLEM
C073-C074 : DENTAL HEALTH -WEARING DENTURES, CHEWING

C076-C090 : PART OF PAIN, DEGREE OF PAIN, DIFFICULTY OF DAILY ACTIVITY

C091-C092 : OTHER ILLNESS AND HEALTH PROBLEM

C093-C095 : MEASURING WEIGH, HEIGH

C096-C130 : HEALTH BEHAVIOR - EXERCISE/NUTRITION / SMOKING / DRINKING

C096-C100 : EXERCISE - REGULAR or IRREGULAR / FREQUENCY / DURATION ETC.
C101-C102 : NUTRITION
C103-C108 : SMOKING - REGULAR or IRREGULAR / FREQUENCY / DURATION ETC.
C109-C130 : DRINKING - REGULAR or IRREGULAR / FREQUENCY / DURATION ETC.

C131-C141 : DEPRESSION

C143 : CHECKPOINT OF INTERVIEWER

SKIP PATTERN CHECKPOINT: SELF-REPORTED HEALTH STATUS TWO SCALES ARE USED TO MEASURE SELF-REPORTED HEALTH STATUS. R WILL BE ASKED TO RATE THEIR HEALTH STATUS TWICE ONCE AT THE BEGINNING OF THIS SECTION, AND AGAIN AT THE END OF THE SECTION IN A RANDOM ORDER.
- IF R IS RANDOMLY ASSIGNED TO ORDER 1(SEC_CA_LIST=1), GO TO C001.
- IF R IS RANDOMLY ASSIGNED TO ORDER 2(SEC_CA_LIST=2), GO TO C142A.

Logic: C001 and C142 Randomly ordered

C001. Next I have some questions about your health. Would you say your health is very good, good, fair, poor or very poor?

[1] Very good
[2] Good
[3] Fair
[4] Poor
[5] Very poor

C142. Next I have some questions about your health. Would you say your health is excellent, very good, good, fair, or poor?

[1] Excellent
[2] Very good
[3] Good
[4] Fair
[5] Poor

C002. Have you ever received disability diagnosis from a doctor?

[1] Yes [5] No → Go to C004

C003. What was the impairment or health problem? Please list all that apply.

[1] Physically handicapped
[2] Brain damage
[3] Vision problem
[4] Hearing problem
[5] Speech impediment
[6] Kidney trouble
[7] Heart problem
[8] Psychiatric problems
[9] Autism

C004. Does this impairment or health problem limit the kind or amount of paid work you can do?

[1] Yes, very much so
[2] Yes, some degree
[3] No, not much
[4] No, not at all

C005. Has a doctor ever told you that you have high blood pressure or hypertension?

[1] Yes [5] No → Go to C009

C006. In what year and month was your hypertension first diagnosed?

IWER: Mark the year and month using six digits. For example, January 2006 should be marked as 200601.

________________________ (range: 190000~200612)

C007. In order to lower your blood pressure, are you currently taking any medication or other treatments?

[1] Yes [5] No

C008. Does the high blood pressure or hypertension limit your daily activities?

[1] Yes [5] No

C009. Has a doctor ever told you that you have diabetes or high blood sugar?

[1] Yes [5] No → Go to C013

C010. In what year and month was that first diagnosed?

IWER: Mark the year and month using six digits. For example, January 2006 should be marked as 200601.

_________________________ (range: 190000~200612)

C011. Are you now using medication that you swallow or using insulin injections to treat or control your diabetes?

[1] Yes [5] No

C012. Does your diabetes limit your daily activities?

[1] Yes [5] No

C013. Has a doctor ever told you that you have cancer or a malignant tumor, excluding minor skin cancers?

[1] Yes [5] No → Go to C019

C014. In what year and month was your cancer or a malignant tumor, excluding minor skin cancers, first diagnosed?

IWER: Mark the year and month using six digits. For example, January 2006 should be marked as 200601.

_________________________ (range: 190000~200612)

C015. In which organ or part of your body do you have cancer?

[1] Liver
[2] Stomach
[3] Lung
[4] Colon
[5] Thyroid
[6] Breast
[7] Cervix
[8] Ovary
→ Go to C017 (for [1]–[8])
[9] Other

C016. Please specify other.

____

C017. Are you now taking any medication to alleviate symptoms (pain, nausea, rash, etc.) or receiving treatment such as chemotherapy?

[1] Yes   [5] No

C018. Does your cancer limit your daily activities?

[1] Yes   [5] No

C019. Has a doctor ever told you that you have chronic lung disease such as chronic bronchitis or emphysema?

[1] Yes   [5] No → Go to C023

C020. In what year and month was your chronic lung disease first diagnosed?

IWER: Mark the year and month using six digits. For example, January 2006 should be marked as 200601.

_________________________ (range: 190000~200612)

C021. Are you now taking medication or other treatment for your lung condition?

[1] Yes   [5] No

C022. Does your lung condition limit your daily activities?

[1] Yes [5] No

C023. Has a doctor ever told you that you have liver disease? (all types of liver disease except fatty liver)

[1] Yes [5] No → Go to C027

C024. In what year and month was your liver disease first diagnosed?

IWER: Mark the year and month using six digits. For example, January 2006 should be marked as 200601.

_________________________ (range: 190000~200612)

C025. Are you now taking medication or other treatment for your liver disease?

[1] Yes [5] No

C026. Does your liver condition limit your daily activities?

[1] Yes [5] No

C027. Has a doctor ever told you that you had a heart attack, coronary heart disease, angina, congestive heart failure, or other heart problems?

[1] Yes [5] No → Go to C031

C028. In what year and month was your heart problem first diagnosed?

IWER: Mark the year and month using six digits. For example, January 2006 should be marked as 200601.

_________________________ (range: 190000 ~ 200612)

C029. Are you now taking or carrying medication or receiving treatment for your heart problem?

[1] Yes [5] No

C030. Does your heart condition limit your daily activities?

[1] Yes [5] No

C031. Has a doctor ever told you that you had a stroke?

[1] Yes
[3] Possible stroke or transient ischemic attack
[5] No → Go to C035

C032. In what year and month was your stroke first diagnosed?

IWER: Mark the year and month using six digits. For example, January 2006 should be marked as 200601.

__________________________ (range: 190000 ~ 200612)

C033. Are you now taking any medications because of your stroke or its complications?

[1] Yes [5] No

C034. Does your condition limit your daily activities?

[1] Yes [5] No

C035. Has a doctor ever told you that you have any emotional, nervous, or psychiatric problems?

[1] Yes [5] No → Go to C039

C036. In what year and month was your emotional, nervous, or psychiatric problems first diagnosed?

IWER: Mark the year and month using six digits. For example, January 2006 should be marked as 200601.

__________________________ (range: 190000 ~ 200612)

C037. Do you now take tranquilizers, antidepressants, sedatives or sleeping pills or get psychiatric or psychological treatment for your problems?

[1] Yes [5] No

C038. Does your condition limit your daily activities?

[1] Yes [5] No

C039. Has a doctor ever told you that you have arthritis or rheumatism?

[1] Yes [5] No → Go to C043

C040. In what year and month was your arthritis or rheumatism first diagnosed?

IWER: Mark the year and month using six digits. For example, January 2006 should be marked as 200601.

_________________________ (range: 190000 ~ 200612)

C041. Are you currently taking any medication or other treatments for your arthritis or rheumatism?

[1] Yes [5] No

C042. Does your arthritis limit your daily activities?

[1] Yes [5] No

C043. Have you ever been in a traffic accident and received medical treatment?

[1] Yes [5] No → Go to C046

C044. In what year and month, were you injured by a traffic accident? If you have been in more than two accidents, please tell me about the most recent one.

IWER: Mark the year and month using six digits. For example, January 2006 should be marked as 200601.

_________________________ (range: 190000 ~ 200612)

C045. Does your injury caused by the traffic accident limit your daily activities?

[1] Yes [5] No

C046. Have you fallen down in the last two years?

[1] Yes [5] No → Go to C051

C047. How many times did you fall in the last two years?

__________times (range: 1~100)

C048. In your most recent fall, did you injure yourself seriously enough to need medical treatment?

[1] Yes [5] No

C049. Have you ever fractured your hip?

[1] Yes [5] No

C050. Does your injury or fracture due to the fall limit your daily activities?

[1] Yes [5] No

C051. Do you worry about falling down?

[1] Not at all
[3] A little bit
[5] A lot

C052. Are there any activities that you refrain from due to the fear of falling down?

[1] Yes [5] No

SKIP PATTERN CHECKPOINT: PROSTATE ILLNESS / INCONTINENCE IF R IS MALE, GO TO C053.
IF R IS FEMALE, GO TO C057.

C053. Have you ever been diagnosed with a prostate illness such as prostate hyperplasia?

[1] Yes [5] No → Go to C062

C054. In what year and month was your prostate illness first diagnosed?

IWER: Record the year and month using six digits. For example, January 2006 should be marked as 200601.

__________________________ (range: 190000 ~ 200612)

C055. Are you now taking mediation or other treatment for your prostate illness?
[1] Yes [5] No

C056. Does your prostate condition limit your daily activities?
[1] Yes → Go to C062 [5] No → Go to C062

C057. This might not be easy to talk about, but during the last 12 months, have you lost any amount of urine beyond your control?
[1] Yes [5] No → Go to C062

C058. On about how many days in the last month have you lost any urine?

_________ days (range: 0~31) → Go to C061
[98] Don't know
[99] Refuse to answer } → Go to C059

C059. Was that more than 5 days?
[1] Yes → Go to C060 [5] No → Go to C061

C060. Was that more than 15 days?
[1] Yes [5] No

C061. Have you ever used any absorbent products such as pads, special garments, sanitary napkins, or toilet paper?
[1] Yes [5] No

C062. Now I have some questions about your eyesight. Do you usually wear glasses or corrective lens?
[1] Yes
[3] Legally blind → Go to C070
[5] No

C063. Is your eyesight very good, good, fair, poor, or very poor (using/not using glasses or corrective lens as usual)?
[1] Very good
[2] Good
[3] Fair
[4] Poor
[5] Very poor

C064. How good is your eyesight for seeing things at a distance, like recognizing a friend across the street?

[1] Very good
[2] Good
[3] Fair
[4] Poor
[5] Very poor

C065. How good is your eyesight for seeing things up close, like reading ordinary newspaper print?

[1] Very good
[2] Good
[3] Fair
[4] Poor
[5] Very poor

C066. Have you ever had cataract surgery?

[1] Yes [5] No → C068

C067. Have you had cataract surgery on both eyes or just one?

[1] One eye only
[5] Both eyes

C068. Has a doctor ever treated you for glaucoma?

[1] Yes [5] No

C069. Does your eyesight limit your daily activities?

[1] Yes [5] No

C070. Now I have some questions about your hearing. Do you ever wear a hearing aid?

[1] Yes [5] No

C071. Is your hearing very good, good, fair, poor, or very poor using a hearing aid as usual?

[1] Very good
[2] Good
[3] Fair
[4] Poor
[5] Very poor

C072. Does your hearing limit your daily activities?

[1] Yes [5] No

C073. Now I have some questions about your dental health. Do you wear dentures?

[1] Yes [5] No

C074. How well can you chew solid foods such as meat or apples without the help of dentures?

[1] Very well
[2] Pretty well
[3] Fair
[4] Not well
[5] Not at all

C075. How easily can you chew solid foods such as meat or apples wearing dentures?

[1] Very well
[2] Pretty well
[3] Fair
[4] Not well
[5] Not at all

C076. Now I have some questions about body pain. On what part of your body do you feel pain? Please list all parts of body you are currently feeling pain.

[1] Head (Headache) → Go to C077
[2] Shoulder → Go to C078
[3] Arm → Go to C079
[4] Wrist → Go to C080
[5] Fingers → Go to C081
[6] Chest → Go to C082
[7] Stomach (Stomachache) → Go to C083
[8] Back → Go to C084
[9] Buttocks → Go to C085
[10] Leg → Go to C086
[11] Knees → Go to C087
[12] Ankle → Go to C088
[13] Toes → Go to C089
[14] No pain → Go to C091

C077. How bad is the headache?

[1] Mild
[3] Moderate
[5] Severe

C078. How bad is the shoulder pain?
[1] Mild
[3] Moderate
[5] Severe

C079. How bad is the arm pain?
[1] Mild
[3] Moderate
[5] Severe

C080. How bad is the wrist pain?
[1] Mild
[3] Moderate
[5] Severe

C081. How bad is the finger pain?
[1] Mild
[3] Moderate
[5] Severe

C082. How bad is the chest pain?
[1] Mild
[3] Moderate
[5] Severe

C083. How bad is the stomachache?
[1] Mild
[3] Moderate
[5] Severe

C084. How bad is the back pain?
[1] Mild
[3] Moderate
[5] Severe

C085. How bad is the buttocks pain?

[1] Mild
[3] Moderate
[5] Severe

C086. How bad is the leg pain?

[1] Mild
[3] Moderate
[5] Severe

C087. How bad is the knee pain?

[1] Mild
[3] Moderate
[5] Severe

C088. How bad is the ankle pain?

[1] Mild
[3] Moderate
[5] Severe

C089. How bad is the toe pain?

[1] Mild
[3] Moderate
[5] Severe

C090. Does the pain make it difficult for you to do daily activities?

[1] Yes [5] No

C091. Are there any other medical diseases or conditions that are important to your health now that we have not talked about?

[1] Yes [5] No → Go to C093

C092. What illness is that?

C093. About how much do you weigh?

__________kilograms (range: 30~200)

C094. Have you gained or lost 5 or more kilograms in the last year?

[1] Yes, I gained weight
[2] Yes, I lost weight
[3] Yes, I gained and lost weight
[4] Yes, I lost and gained weight
[5] No

C095. About how tall are you?

________centimeters (range: 70~210)

C096. The next few questions are about exercise. Do you work out more than once a week?

[1] Yes → Go to C098 [5] No

C097. What is the main reason for you not being able to exercise regularly?

[1] Too busy
[2] No space or place to work out
[3] Too lazy
[4] Do not like exercise
[5] Never thought about doing exercise

} → Go to C101

C098. How often do you work out per week?

_________times / per week (range: 1 ~ 97)

C099. For how long do you work out per session?

IWER: Calculate one hour as 60 minutes. For example, mark 150 minutes for 2 hours and 30 minutes.

_________minutes (range: 1 ~ 168)

C100. How long have you been working out regularly?
[1] Less than 3 months
[2] 4-6 months
[3] 7 months-1 year
[4] 1-2 years
[5] 3-4 years
[6] 5-6 years
[7] More than 7 years

C101. Now, I am going to ask you about the meals you had for the last two days. Did you have three meals yesterday? Choose all that apply.
[1] I had breakfast
[2] I had lunch
[3] I had dinner
[4] I didn't eat anything

C102. Did you have three meals the day before yesterday? Choose all that apply
[1] I had breakfast
[2] I had lunch
[3] I had dinner
[4] I didn't eat anything

C103. Have you ever smoked more than 5 packs of cigarettes (100 cigarettes)?
[1] Yes [5] No → Go to C109

C104. Do you smoke cigarettes now?
[1] Yes [5] No → Go to C106

C105. About how many cigarettes or packs do you usually smoke in a day now?
IWER: One pack is 20 cigarettes. For example, mark 30 for one and a half packs

___________cigarettes / day (ragne: 1 ~ 100)

C106. In what year and month did you first start smoking?
IWER: Enter year and month in 6 digits. If R doesn't recall the month, enter 00. For example, if R answers 20 years ago and doesn't remember the month, enter 198600.

_________________________ (range: 190000 ~ 200612)

C107. When you were smoking the most, about how many cigarettes or packs did you usually smoke in a day?

IWER: One pack is 20 cigarettes. For example, mark 30 for one and a half packs.

___________cigarettes / day (range: 1~200)

C108. In what year and month did you stop smoking?

IWER: Enter year and month in 6 digits. If R doesn't recall the month, enter 00. For example, if R answers 20 years ago and doesn't remember the month, enter 198600.

_________________________ (range: 190000~200612)

C109. Do you ever drink any alcoholic beverages, such as beer, wine, or liquor?

[1] Yes → Go to C113 [5] No

C110. Have you never had alcoholic beverages due to some reasons (e.g., religious, health-related, etc), or you used to have some drinks in the past?

[1] No, I never had a drink → Go to C131
[5] Yes, I used to drink

C111. Do you still drink?

[1] Yes, I drink from time to time → Go to C113
[5] No, I quit drinking → C112 ~ C114, then go to C131

C112. In what year and month did you quit drinking?

IWER: Record year and month in 6 digits. For example, if was 20 years ago and March, enter 198603. If R doesn't remember the month, record 00.

_________________________ (range: 190000 ~ 200612)

C113. When did you start drinking?

IWER: Record year and month in 6 digits. For example, if was 10 years ago and March, enter 198603. If R doesn't remember the month, record 00.

_________________________ (range: 190000 ~ 200612)

C114. How many years have you been drinking alcoholic beverages altogether? If you quit drinking for a while, please do not count the years that you did not drink.

[1] 1 – 5 years
[2] 6 – 10 years
[3] 11 – 20 years
[4] 21 – 30 years
[5] 31 – 40 years
[6] More than 41 years

C115. Now, I am going to ask you how often and how much you drink during the past year. Please tell me how often you drank per month, and how much you drank at a time on average. I will repeat the questions for different types of alcoholic beverage.

[1] Confrim

C116. Soju (Korean liquor)

[1] None or less than once a month → Go to C118
[2] Once a month
[3] 2 – 3 times a month
[4] Once a week
[5] 2 – 3 times a week
[6] 4 – 6 times a week
[7] Once a day
[8] More than twice a day

C117. How many glasses of Soju do you drink at a time? (1bottle = 6.5 glasses, 1 glass = 50cc)

_________ glasses

C118. Beer

[1] None or less than once a month → Go to C120
[2] Once a month
[3] 2-3 times a month
[4] Once a week
[5] 2 – 3 times a week
[6] 4 – 6 times a week
[7] Once a day
[8] More than twice a day

C119. How many glasses of beer do you drink at a time? (1bottle = 2.5 mugs, 1mug = 220cc)

_________ glasses

C120. Makgeolli (rice wine)

[1] None or less than once a month → Go to C122
[2] Once a month
[3] 2 – 3 times a month
[4] Once a week
[5] 2 – 3 times a week
[6] 4 – 6 times a week
[7] Once a day
[8] More than twice a day

C121. How many glasses of Makgeolli do you drink at a time? (1bottle = 7 glasses, 1 glass = 240cc)

_________ glasses

C122. Whisky and other liquors

[1] None or less than once a month → Go to C124
[2] Once a month
[3] 2 – 3 times a month
[4] Once a week
[5] 2 – 3 times a week
[6] 4 – 6 times a week
[7] Once a day
[8] More than twice a day

C123. How many glasses of whisky or liquor do you drink at a time? (1 bottle = 23 glasses, 1 glass = 30cc)

_________ glasses

C124. Wine

[1] None or less than once a month → Go to C126
[2] Once a month
[3] 2-3 times a month
[4] Once a week
[5] 2- – 3 times a week
[6] 4 – 6 times a week
[7] Once a day
[8] More than twice a day

C125. How many glasses of wine do you drink at a time? (1 bottle = 8 glasses, 1 glass = 90cc)

_________ glasses

C126. Have you ever felt that you should quit drinking?

[1] Yes [5] No

C127. Has anyone criticized your drinking?

[1] Yes [5] No → Go to C129

C128. Have people ever annoyed you by criticizing your drinking?

[1] Yes [5] No

C129. Have you ever felt bad or guilty about drinking?

[1] Yes [5] No

C130. Have you ever taken a drink first thing in the morning to steady your nerves or get rid of a hangover?

[1] Yes [5] No

* Depression *

C131. Have you ever had feelings of being sad, blue, or depressed for two weeks or more during the past year?

[1] Yes
[3] Did not feel depressed because I was taking anti-depressant medication → Go to C142
[5] No

C132. Next I will ask about how you felt and behaved during the last week. Please think of how often you felt or behaved like followings. During the last week, how often did you lose interest in most things?

[1] Very rarely (less than one day)
[2] Sometimes (1 – 2 days)
[3] Often (3 – 4 days)
[4] Almost always (5 – 7 days)

C133. During the last week, how often did you have trouble concentrating?

[1] Very rarely (less than one day)
[2] Sometimes (1 – 2 days)
[3] Often (3 – 4 days)
[4] Almost always (5 – 7 days)

C134. During the last week, how often did you feel depressed?

[1] Very rarely (less than one day)
[2] Sometimes (1 – 2 days)
[3] Often (3 – 4 days)
[4] Almost always (5 – 7 days)

C135. During the last week, how often did you feel tired out or low in energy?

[1] Very rarely (less than one day)
[2] Sometimes (1 – 2 days)
[3] Often (3 – 4 days)
[4] Almost always (5 – 7 days)

C136. How was your last week? How often did you feel pretty good?

[1] Very rarely (less than one day)
[2] Sometimes (1 – 2 days)
[3] Often (3 – 4 days)
[4] Almost always (5 – 7 days)

C137. During the last week, how often were you afraid of something?

[1] Very rarely (less than one day)
[2] Sometimes (1 – 2 days)
[3] Often (3 – 4 days)
[4] Almost always (5 – 7 days)

C138. During the last week, how often did you have trouble falling asleep?

[1] Very rarely (less than one day)
[2] Sometimes (1 – 2 days)
[3] Often (3 – 4 days)
[4] Almost always (5 – 7 days)

C139. How often did you feel you were overall satisfied last week?
[1] Very rarely (less than one day)
[2] Sometimes (1 – 2 days)
[3] Often (3 – 4 days)
[4] Almost always (5 – 7 days)

C140. How often did you feel alone last week?
[1] Very rarely (less than one day)
[2] Sometimes (1 – 2 days)
[3] Often (3 – 4 days)
[4] Almost always (5 – 7 days)

C141. How often have you felt down on yourself, no good or worthless last week?
[1] Very rarely (less than one day)
[2] Sometimes (1 – 2 days)
[3] Often (3 – 4 days)
[4] Almost always (5 – 7 days)

SKIP PATTERN CHECKPOINT: SELF-REPORTED HEALTH STATUS TWO SCALES ARE USED TO MEASURE SELF-REPORTED HEALTH STATUS. R WILL BE ASKED TO RATE THEIR HEALTH STATUS TWICE ONCE AT THE BEGINNING OF THIS SECTION, AND AGAIN AT THE END OF THE SECTION IN A RANDOM ORDER.
– IF R IS RANDOMLY ASSIGNED TO ORDER 1(SEC_CA_LIST=1), GO TO C001.
– IF R IS RANDOMLY ASSIGNED TO ORDER 2(SEC_CA_LIST=2), GO TO C142A.

Logic: C001 and C142 are randomly ordered

C001. Next I have some questions about your health. Would you say your health is very good, good, fair, poor or very poor?
[1] Very good
[2] Good
[3] Fair
[4] Poor
[5] Very poor

C142. How would you rate your health status? Would you say your health is excellent, very good, good, fair, or poor?
[1] Excellent
[2] Very good
[3] Good
[4] Fair
[5] Poor

C143. IWER: HOW OFTEN DID R RECEIVE ASSISTANCE ANSWERING SECTION Ca-HEALTH STATUS?

[1] Never
[2] A few times
[3] Most or all of the time
} → Go to Section Cb.
[4] The Section was done by a proxy reporter

C144. IWER: WHAT IS THE PROXY'S RELATIONSHIP TO R? IF UNKNOWN, PLEASE ASK THE PROXY. What is your relationship to R?

[1] Spouse
[2] Mother
[3] Father
[4] Mother-in-law
[5] Father-in-law
[6] Sibling
[7] Brother-in-law, sister-in-law
[8] Child
[9] Spouse of child
[10] Grandchild
[11] Other relative
[12] Helper or other non-relative
→ Go to Section Cb.

Cb. FUNCTIONAL LIMITATIONS AND HELPERS

Cb. FUNCTIONAL LIMITATIONS AND HELPERS SURVEY STRUCTURE

Cb01-Cb07 : ABILITY OF DAILY ACTIVITY(ADL)

- DRESSING, WASHING FACE / HAIR / BRUSHING TEETH, BATHING / SHOWERING, EATING, GETTING OUT OF THE BED, USING THE TOILET, CONTROLLING URINATION

Cb08-Cb17 : ABILITY OF INSTRUMENTAL DAILY ACTIVITY (IADL)

- PERSONAL GROOMING, HOUSEHOLD CHORES, PREPARING MEALS, DOING LAUNDRY, GOING OUT IN A SHORT DISTANCE, USING TRANSPORTATION, SHOPPING, MANAGING THE MONEY, MAKING PHONE CALL, TAKING MEDICATIONS

Cb18-Cb32 : R'S HELPERS FOR DAILY ACTIVITY (FIRST / SECOND / THIRD HELPERS)

- NAME OF HELPER, RELATIONSHIP WITH RESPONDENT, DAYS AND HOURS OF HELP (MONTH), PAYING FOR HELPING

Cb18-Cb21 : A PERSON THAT HELP MOST FOR DAILY ACTIVITY
Cb22-Cb25 : A PERSON THAT HELP SECOND MOST FOR DAILY ACTIVITY
Cb26-Cb29 : OTHER PERSON THAT HELP FOR DAILY ACTIVITY
Cb30-Cb31 : COST OF HELP, A PERSON OF PAYING THE COST OF HELP
Cb32-Cb33 : A PERSON THAT HELP RESPONDENT IN THE FUTURE, RELATIONSHIP TO RESPONDENT

Cb34 : CHECK POINT OF INTERVIEWER

* ADL *

Cb01. We need to understand difficulties people may have with various activities because of a health or physical problem and help from others they need doing those activities. Please tell me whether you have any difficulty doing each of the everyday activities that I read to you during the last week. Exclude any difficulties that you expect to last less than three months.
Because of health and memory problems, do you have any difficulty with dressing? Dressing includes taking clothes out from a closet, putting them on, buttoning up, and fastening the belt.

[1] No, I don't need any help.
[3] Yes, I need help to some extent.
[5] Yes, I need help in every respect.

Cb02. Do you have any difficulty with washing your face and hair and brushing your teeth?
[1] No, I don't need any help.
[3] Yes, I need help to some extent.
[5] Yes, I need help in every respect.

Cb03. Do you have any difficulty with bathing or showering?
[1] No, I don't need any help.
[3] Yes, I need help to some extent.
[5] Yes, I need help in every respect.

Cb04. Do you have any difficulty with eating, such as cutting up your food?
IWER: By eating, we mean eating food by oneself when it is ready.
[1] No, I don't need any help.
[3] Yes, I need help to some extent.
[5] Yes, I need help in every respect.

Cb05. Do you have any difficulty with getting out of bed and walking across a room? You may use equipment or devices to get out of bed and walk across a room.
[1] No, I don't need any help.
[3] Yes, I need help to some extent.
[5] Yes, I need help in every respect.

Cb06. Do you have any difficulties with using the toilet, getting up and down?
[1] No, I don't need any help.
[3] Yes, I need help to some extent.
[5] Yes, I need help in every respect.

Cb07. Do you have any difficulties with controlling urination and defecation? You may use a catheter (conduit) or a pouch by yourself.
[1] No, I don't need any help.
[3] Yes, I need help to some extent.
[5] Yes, I need help in every respect.

* IADL *

Cb08. Here are few other activities that some people have difficult because of a physical, mention, emotional, or memory problem. Please tell me whether you have any difficulty doing each activity I name. Exclude any difficulties that you expect to last less than three months.

Do you have any difficulties with personal grooming?

IWER: By personal grooming, we mean brushing hair, putting on make-up, shaving, and clipping nails/toenails.

[1] No, I don't need any help.
[3] Yes, I need help to some extent.
[5] Yes, I need help in every respect.

Cb09. Do you have any difficulties with doing household chores?

IWER: By doing household chores, we mean house cleaning, doing dishes, making the bed, and aranging the house. If R cannot mop the floor, but can scrub, or R cannot fold heavy bedding, but is able to do light ones, then mark [3]

[1] No, I don't need any help.
[3] Yes, I need help to some extent.
[5] Yes, I need help in every respect.

Cb10. Do you need any help with preparing hot meals?

IWER: By preparing hot meals, we mean preparing ingredients, cooking, and serving food. If another person prepares ingredients or if R can cook rice, but is not able to prepare side dishes, then mark [3]

[1] No, I don't need any help.
[3] Yes, I need help to some extent.
[5] Yes, I need help in every respect.

Cb11. Do you have any difficulty with doing laundry?

IWER: By doing laundry, we mean laundering either using machine or by hand and drying. If R cannot do laundry of large items but is able to do small items such as underwear and socks by hand, then mark [3]

[1] No, I don't need any help.
[3] Yes, I need help to some extent.
[5] Yes, I need help in every respect.

Cb12. Do you have any difficulty with taking a walk or going out in a short distance without using transportation (for example, going to a neighborhood store on foot)?

IWER: Enter [1] if R uses assistance device such as a cane. Enter [3] if R needs to ride a wheelchair.

[1] No, I don't need any help.
[3] Yes, I need help to some extent.
[5] Yes, I need help in every respect.

Cb13. Do you have any difficulty with using transportation, such as buses, subways, taxies, and cars?

[1] No, I don't need any help.
[3] Yes, I need help to some extent.
[5] Yes, I need help in every respect.

Cb14. Do you have any difficulties with shopping? By shopping, we mean deciding what to buy and paying for it.

[1] No, I don't need any help.
[3] Yes, I need help to some extent.
[5] Yes, I need help in every respect.

Cb15. Do you have any difficulties with managing your money, such as paying your bills, keeping track of expenses, or managing assets?

IWER: Enter [3] if someone else manages assets.

[1] No, I don't need any help.
[3] Yes, I need help to some extent.
[5] Yes, I need help in every respect.

Cb16. Do you have any difficulties with making phone calls?

IWER: Enter [3] if R can only dial numbers s/he knows.

[1] No, I don't need any help.
[3] Yes, I need help to some extent.
[5] Yes, I need help in every respect.

Cb17. Do you have any difficulties with taking medications? By taking medications, we mean taking the right portion of medication right on time.

[1] No, I don't need any help.
[3] Yes, I need help to some extent.
[5] Yes, I need help in every respect.

* ADL / IADL HELPER *

SKIP PATTERN CHECKPOINT: FUNCTIONAL DIFFICULTIES - IF R NEEDS ANY HELP IN Cb01~Cb17 (BOTH HELP TO SOME EXTENT, [3], AND IN EVERY RESPECT, [5]), GO TO Cb18.
- IF R DOES NOT NEED ANY HELP, GO TO Cb32.

Cb18. Who most often helps you with (dressing, washing, bathing, eating, getting out of bed, using toilet, controlling urination and defecation, grooming, doing the chores, preparing hot meals, doing laundry, going out, using transportations, shopping, managing money, making phone calls, taking medications)?
(Select from the list displayed by CAPI)

[2] Spouse
[3] Mother
[4] Father
[5] Mother-in-law
[6] Father-in-law
[7 ~ 26] Children
[27 ~ 46] Sibling
[47] Brother-in-law, sister-in-law
[48] Spouse of child
[49] Grandchild
[50] Other relative
[55] Helper or other non-relative
[56] No one helped → Go to Cb32

Cb19. During the last month, on about how many days did helper's name chosen from Cb18 help you?

____________ days (range: 1~31)

Cb20. On the days helper's name chosen from Cb18 helps you, about how many hours per day is that?
IWER: less than an hour=1

____________ hours (range: 1~24)

Cb21. Is helper's name chosen from Cb18 paid to help you?

[1] Yes [5] No

Cb22. Who second most often helps you with (dressing, washing, bathing, eating, getting out of bed, using toilet, controlling urination and defecation, grooming, cooking, doing laundry, running errands, using public transportation, shopping, managing money, making phone calls, and taking medicine)?
(Select from the list displayed by CAPI)

[2] Spouse
[3] Mother

[4] Father
[5] Mother-in-law
[6] Father-in-law
[7 ~ 26] Children
[27 ~ 46] Sibling
[47] Brother-in-law, sister-in-law
[48] Spouse of child
[49] Grandchild
[50] Other relative
[55] Helper or other non-relative
[57] No one else helped → Go to Cb30

Cb23. During the last month, on about how many days did helper's name chosen from Cb22 help you?

____________ days in last month (range: 1 ~ 31)

Cb24. On the days helper's name chosen from Cb22 helps you, about how many hours per day is that?

IWER: less than an hour = 1

____________ hours (range: 1~24)

Cb25. Is helper's name chosen from Cb22 paid to help you?

[1] Yes [5] No

Cb26. Is there any other person helped you? If so, who most often helps you next? (Select from the list displayed by CAPI)

[2] Spouse
[3] Mother
[4] Father
[5] Mother-in-law
[6] Father-in-law
[7 ~ 26] Children
[27~46] Sibling
[47] Brother-in-law, sister-in-law
[48] Spouse of child
[49] Grandchild
[50] Other relative
[55] Helper or other non-relative
[58] No one else helped → Go to Cb30

Cb27. During the last month, on about how many days did helper's name chosen from Cb26 help you?

____________ days (range: 1~31)

Cb28. On the days helper's name chosen from Cb26 helps you, about how many hours per day is that?

IWER: less than an hour = 1

____________ hours (range: 1~24)

Cb29. Was helper's name chosen from Cb26 paid to help you?

[1] Yes [5] No

SKIP PATTERN CHECKPOINT: PAID HELPER

- IF R PAID FOR HELP IN Cb21, Cb25, OR Cb29, GO TO Cb30.
- IF R DID NOT PAY FOR HELP, GO TO Cb32.

Cb30. About how much in total did you pay for the help during the past month?

______________ MW (10,000 Korean won) (range: 1~997)

Cb31. Who paid this cost most? Please choose one person who paid the most. (Select from the list displayed by CAPI)

[1] R
[2] Spouse
[3] Mother
[4] Father
[5] Mother-in-law
[6] Father-in-law
[7 ~ 26] Children
[27 ~ 46] Sibling
[47] Brother-in-law, sister-in-law
[48] Spouse of child
[49] Grandchild
[50] Other relative
[51] Other

Cb32. Suppose in the future, you needed help with basic daily activities like eating or dressing. Do you have relatives or friends (besides your spouse/partner) who would be willing and able to help you over a long period of time?

[1] Yes [5] No → Go to Cb34

Cb33. What is the relationship to you of that person or persons?

IWER: CHOOSE ALL THAT APPLY

(Select from the list displayed by CAPI)

[3] Mother
[4] Father
[5] Mother-in-law
[6] Father-in-law
[7 ~ 26] Children
[27 ~ 46] Sibling
[47] Brother-in-law, sister-in-law
[48] Spouse of child
[49] Grandchild
[50] Other relative
[51] Other
[59] Volunteer or Employee of facility
[60] Paid helper

Cb34. IWER: HOW OFTEN DID R RECEIVE ASSISTANCE WITH ANSWERS IN SECTION Cb – FUNCTIONAL LIMITATIONS AND HELPERS?

[1] Never
[2] A few times
[3] Most or all of the time
→ Go to Section Cc.
[4] The Section was done by a proxy reporter.

Cb35. IWER: WHAT IS THE PROXY'S RELATIONSHIP TO R? IF UNKNOWN, PLEASE ASK THE PROXY. What is your relationship to R?

[1] Spouse
[2] Mother
[3] Father
[4] Mother-in-law
[5] Father-in-law
[6] Sibling
[7] Brother-in-law, sister-in-law
[8] Child
[9] Spouse of child
[10] Grandchild
[11] Other relative
[12] Helper or other non-relative
→ Go to Section Cc.

Cc. HEALTH INSURANCE AND SERVICES

Cc. HEALTH INSURANCE AND SERVICES SURVEY STRUCTURE

Cc01-Cc16 : HEALTH SECURITY

Cc01-Cc04 : NATIONAL HEALTH INSURANCE(EMPLOYER/COMMUNITY), MEDICAL AID PROGRAM (TYPE 1/TYPE 2)

Cc05-Cc08 : NATIONAL HEALTH PREMIUM - PAYER FOR NATIONAL HEALTH PREMIUMS, THE AMOUNT OF INSURANCE PREMIUM, MISSING PAYMENT, TERMS OF MISSING PAYMENT

Cc09 : MEDICAL AID PROGRAM - TYPE 1/TYPE 2

Cc10-Cc12 : PRIVATE HEALTH INSURANCE - ENROLLMENT, NUMBERS OF INSURANCE ENROLLED, THE AMOUNT OF INSURANCE PREMIUM

Cc13-Cc16 : HEALTH CHECK - BASIC/SECOND FREE HEALTH CHECK, OTHER HEALTH CHECK

Cc17-Cc39 : UTILIZATION OF MEDICAL FACILITY

Cc17-Cc26 : HOSPITALIZATION - TIMES AND TERMS OF HOSPITALIZATION, KIND OF FACILITIES, COST OF HOSPITALIZATION, CARETAKERS DAYS AND TIMES OF CARING, AMOUNT OF PAYING FOR CARING

Cc27-Cc28 : DENTAL CLINIC - TIMES OF VISITING, AMOUNT OF PAYING MEDICAL TREATMENT

Cc29: PUBLIC HEALTH CLINIC - TIMES OF VISITING

Cc30-Cc31 : ORIENTAL CLINIC - TIMES OF VISITING, AMOUNT OF PAYING MEDICAL TREATMENT

Cc32-Cc33 : OTHER VISITING TO A DENTIST, PUBLIC, AND ORIENTAL HEALTH CLINIC - TIMES OF VISITING, AMOUNT OF PAYING MEDICAL TREATMENT

Cc34-Cc35 : DROP OF MEDICALLY TRAINED PERSON SUCH AS DOCTORS AND NURSE - TIMES OF DROP, AMOUNT OF PAYING MEDICAL TREATMENT

Cc36-Cc37 : PRESCRIPTION MEDICATION - REGULAR PRESCRIPTION MEDICATION, AMOUNT OF PAYING FOR PRESCRIPTION MEDICATION

Cc38-Cc39 : OTHER MEDICAL ASSISTANCE EQUIPMENT - MEDICAL ASSISTANCE EQUIPMENT SUCH AS HEARING AIDS OR WHEELCHAIRS, AMOUNT OF PAYMENT

Cc40 : CHECK POINT OF INTERVIEWER

Cc01. The next questions are about health insurance, both public and private. First, in public insurance, there are two types of public health insurance: National Health Insurance and Medical Aid. National Health Insurance Program is a public health insurance program for all Koreans residing in the Korean territory. Medical Aid Program is a public health insurance program for the awardees of the National Merit Award and other honorary people as well as people with low incomes.
Are you currently covered by the National Health Insurance Program or Medical Aid Program?

[1] National Health Insurance
[5] Medical Aid Program → Go to Cc09
[8] Don't know → Go to Cc10
[9] Refuse to answer → Go to Cc10

Cc02. There are two ways to enroll in the National Health Insurance Program: through current employer or community. How did you enroll in NHI, through current employer or community?

[1] Through current (R's or R's family's) employer
[5] Through community → Go to Cc04

Cc03. If you are insured through your employer, are you the insured person or a dependent family member?

[1] Insured person
[5] Dependent family member } → Go to Cc05

Cc04. If you are enrolled through community, are you the household head?

[1] Yes [5] No

Cc05. Who is currently paying for your heath insurance premium?

[1] You
[2] Spouse
[3] Child / ren
[4] Son / daughter-in-law
[5] Other relatives

Cc06. Not including co - pays, how much do you, yourself, or the person who is currently paying, pay in premiums for this plan? (Unit: 10,000 Korean Won)

_________________________MW (range: 1~120)

Cc07. Have you or the person who is currently paying, missed the payment last month?

IWER: IF R DOESN'T KNOW OR REFUSES TO ANSWER, GO TO Cc10.

[1] Yes [5] No → Go to Cc10

Cc08. For how many months have you missed payments?

__________Months (range: 1~203) → Go to Cc10

Don't know
Refuse to answer } → Go to Cc10

Cc09. Is it Type 1 Medical Aid Program or Type 2 Medical Aid Program?

[1] Type 1
[5] Type 2

Cc10. Are you covered by any private health insurance?

[1] Yes [5] No → Go to Cc13

Cc11. How many private health insurance do you have?

____________ (range: 1~30)

Cc12. What is the total amount of insurance premium you pay each month for these private health insurance? (Unit: 10,000 Korean Won)

____________ MW (range: 0~997)

Cc13. Have you received the basic medical checkup covered by the National Health Insurance or Medical Aid Program in the past 2 years?

[1] Yes → Go to Cc15 [5] No

Cc14. Why did you not receive a medical checkup?

[1] Wanted to have a medical checkup, but unable to because I had trouble getting around
[2] Didn't expect any problem, as I was fine in the previous checkup
[3] Too busy, couldn't have time
[4] I couldn't trust the results
[5] I was afraid of the results
[6] Even if an illness is detected, no adequate treatment is available
[7] Didn't see a need
[8] I didn't know it is free

Cc15. Have you ever received any further health check covered by the National Health Insurance or Medical Aid Program due to problems found in the basic medical exam?

[1] Yes [5] No

Cc16. Have you had any other medical checkup using your out-of-pocket money in the past two years?

[1] Yes [5] No

Cc17. The next questions are about health services you received in the past year. Have you been a patient overnight in a hospital, nursing home, convalescent home, or other long-term health care facility during the past 12 months? If yes, how many times have you been an overnight patient last year?

IWER: Hospitalization for the purpose of long-term stay rather than medical treatment is excluded.

____________ Number of Times (range: 0~52) (IF R HAS NOT BEEN HOSPITALIZED, GO TO Cc27)

Cc18. Please recall the last time you were hospitalized last year. Which kind of facilities have you been in?

[1] Hospital
[5] Long-term care hospital

Cc19. How many nights did you stay in this hospital?

____________ Number of Nights (range: 1~365)

Cc20. About how much did you, yourself, pay out-of-pocket for this hospitalization? Please exclude the amount covered by private health insurance or other family members paid for. (Unit: 10,000 Korean Won)

____________ MW (Range: 0~9997)

Cc21. The next questions will be about the caretakers during your most recent hospitalization. Who took care of you when you were a patient in the medical facilities? Please do not include those who simply visited.

[1] Spouse
[2] Mother
[1] Father
[2] Mother-in-law
[3] Father-in-law
[7 ~ 26] Children
[27 ~ 46] Sibling
[47] Brother-in-law, sister-in-law
[48] Spouse of child
[49] Grandchild
[50] Other relative
[59] Volunteer or employee of facility
[61] Paid helper
[62] Other

(Items [1] through [62]) → Go to Cc23

[63] None → Go to Cc27

Cc22. Please specify other.

____

Cc23. Who took care of you most when you were hospitalized?

[2] Spouse
[3] Mother
[1] Father
[2] Mother-in-law
[3] Father-in-law
[7 ~ 26] Children
[27 ~ 46] Sibling
[47] Brother-in-law, sister-in-law
[48] Spouse of child
[49] Grandchild
[50] Other relative
[59] Volunteer or employee of facility
[61] Paid helper
[62] Other

Cc24. How long did the care provider chosen from Cc23 take care of you?

____________ days (range: 1 ~ 365)

Cc25. How many hours per day did the care provider chosen from Cc23 take care of you?

____________ hours (range: 1 ~ 24)

Cc26. If any, how much did you, yourself, pay for the care per day? Please do no include the amount covered by private insurance plan or other family members such as children or parents. (Unit: 10,000 Korean Won per day)

____________ MW (range: 0~50)

Cc27. In the last 12 months, have you seen a dentist for dental care, including dentures? If you did, how many times have you seen a dentist during the last year?

____________ Number of Times (range: 0~365) **(IF R HAS NOT BEEN TO, GO TO Cc29.)**

Cc28. About how much did you pay out-of-pocket for dental bills last year? Please do no include the amount covered by private insurance plan or other family members such as children or parents. (Unit: 10,000 Korean Won)

____________ MW (range: 0~9997)

Cc29. In the past 12 months, have you seen a doctor at a public health clinic? If you did, how many times have you seen a doctor at a public health clinic?

____________ Number of Times (range: 0~365)

Cc30. In the past 12 months, have you seen a doctor at an oriental clinic? If you did, how many times?

____________ Number of Times (range: 0~365) **(IF R HAS NOT BEEN TO, GO TO Cc32.)**

Cc31. How much did you, yourself, pay out-of-pocket for oriental clinic bills last year? Please do no include the amount covered by private insurance plan or other family members such as children or parents. (Unit: 10,000 Korean Won)

____________ MW (range: 0~9997)

Cc32. (Excluding hospitalization and the visits to a dentist, public health clinic, and oriental health clinic) In the past 12 months, have you ever visited a doctor's office, including emergency room and hospital outpatient office? If you did, how many times have you visited last year?

____________ Number of Times (range: 0~365) **(IF R HAS NOT BEEN TO, GO TO Cc34.)**

Cc33. About how much did you yourself pay out-of-pocket for the visit last year? Please do no include the amount covered by private insurance plan or other family members such as children or parents. (Unit: 10,000 Korean Won)

____________ MW (range: 0~9997)

Cc34. In the past 12 months, has any doctors, nurses, or other medically−trained person come to your home and provided care?

If so, how many times did the medically−trained person come to your home and provide care last year?

IWER: Medically-trained persons include professional nurses, visiting nurse's aides, physical or occupational therapists, chemotherapists, and respiratory oxygen therapists. Only include help given to r, not help for r when is a care giver for someone else

____________ Number of Times (range: 0 ~ 365) (IF R HAS NOT RECEIVED HOME CARE, GO TO Cc36.)

Cc35. How much did you pay out-of-pocket for in-home medical care last year? Please do not include the amount covered by private insurance plan or other family members such as children or parents. (Unit: 10,000 Korean Won)

____________ MW (range: 0 ~ 9997)

Cc36. In the past 12 months, have you regularly take prescription medication?

[1] Yes [5] No → Go to Cc38

Cc37. About how much have you paid out-of-pocket for these prescriptions last year? Please do not include the amount covered by private insurance plan or other family members such as children or parents. (Unit: 10,000 Korean Won)

____________ MW (range: 0 ~ 9997)

Cc38. In the past 12 months, have you purchased a medical assistance device or equipment, such as hearing aids or wheelchairs?

[1] Yes [5] No → Go to Cc40

Cc39. How much in total did you pay out-of-pocket for these equipments last year? Please do no include the amount covered by private insurance plan or other family members such as children or parents. (Unit: 10,000 Korean Won)

____________ MW (range: 0 ~ 9997)

Cc40. IWER: HOW OFTEN DID R RECEIVE ASSISTANCE WITH ANSWERS IN SECTION Cc-HEALTH INSURANCE AND SERVICES?

[1] Never
[2] A few times
[3] Most or all the time
} → Go to Section Cd.
[4] The Section was done by a proxy reporter.

Cc41. IWER: WHAT IS THE PROXY'S RELATIONSHIP TO R? IF UNKNOWN, PLEASE ASK THE PROXY. What is your relationship to R?

[1] Spouse
[2] Mother
[3] Father
[4] Mother-in-law
[5] Father-in-law
[6] Sibling
[7] Brother-in-law, sister-in-law
[8] Child
[9] Spouse of child
[10] Grandchild
[11] Other relative
[12] Helper or other non-relative
→ Go to Section Cd.

Cd. COGNITION

Cd. COGNITION SURVEY STRUCTURE

Cd01-Cd05 : AWARENESS

Cd01-Cd03 : AWARENESS OF TIME – YEAR / MONTH / DAY, DAY OF WEEK, SEASON
Cd04-Cd05 : AWARENESS OF PLACE – CURRENT PLACE, ADDRESS

Cd06, Cd12 : SHORT AND LONG TERM MEMORY TESTS

Cd07-Cd11 : CONCENTRATIONS AND CALCULATION ABILITY

Cd13-Cd14 : A USE OF HAVING ITEMS

Cd15 : LISTEN AND REPEAT ABILITY

Cd16-Cd19 : CARRYING OUT THE ORDERS – FOLDING AND REVERSING PAPER, READING SENTENCE, WRITING, DRAWING

Cd20 : CHECK POINT OF INTERVIEWER

Cd50. Before we go on to the next Section, I'd like to check if this is a self or proxy interview. Are you R's FIRST, LAST NAME?

[1] Yes　　[5] No → Go to Cd51

Cd01. Now I'm going to ask several simple questions. Some may be easy and some may be hard to answer. Please try to answer as honestly as you can. Are you ready? Please tell me today's date.

IWER: R doesn't have to answer in this order. If R is an elderly person and marked the date by lunar calendar, that date is correct if it matches with the solar calendar. You can check the accuracy, using the converter.

[1] One of the month / day / year combination is correct
[2] Two of the month / day / year combination are correct
[3] All of the month / day / year combination are correct
[5] All of the month / day / year combination are incorrect

Cd02. Please tell me which day of week is today. Is it Monday, Tuesday, Wednesday, Thursday, Friday, Saturday, or Sunday?

[1] Day of week OK
[5] Day of week not OK

Cd03. What is the current season (among Spring, Summer, Fall, or Winter)?

[1] Season OK
[5] Season not OK

Cd04. What is this place used for?

IWER: PLAUSIBLE ANSWERS ARE SPECIFIC ANSWERS SUCH AS HOSPITAL, MARKET, LIVING ROOM, HOUSE, APARTMENT, NURSING HOME, ETC.

[1] Correct
[5] Not correct

Cd05. What is your address? Please tell me the city, Gun, Dong, APT #/Street Number.

[1] One of the city / gun / dong / street address combination is correct
[2] Two of the city / gun / dong / street address combination are correct
[3] Three of the city / gun / dong / street address combination are correct
[4] All of the city / gun / dong / street address combination is correct
[5] All of the city / gun / dong / street address combination is incorrect

Cd06. Please listen carefully as I read three words because I cannot repeat them. When I finish, I will ask you to recall aloud as many of the words as you can, in any order. Are you ready?

IWER: [1] Tidy up the surroundings and start. [2] Assigned words are displayed only to the IWER. [3] Read the items at a slow, steady rate and stop for one second before reading the next word [4] If R is unable to recall all 3 words or can recall only a few words, repeat the words up to 5 times including the first try until he/she can recall all of them. [5] Stop the process even after the Respondent can't recall the words after 5 tries. [6] Answers given by repetition should be marked as incorrect.

[1] Only one of the three words is correct, in any order
[2] Two of the three words are correct, in any order
[3] All of the three words are correct, in any order
[5] None of the three words is correct

Logic : List shows randomly
LIST 1: airplane, pencil, pine tree
LIST 2: snowman, chair, peach
LIST 3: teacher, factory, washing machine
LIST 4: chopsticks, apple, hanger
LIST 5: bat, shoes, sea water

Cd07. Try to remember the words I just read to you. I'll ask you to recall them later. Read once more if R did not recall any of the words, up to 3 times and go on. If R does not recall, ensure them it is OK so R would feel comfortable.
Let's try some subtraction of numbers this time. What are 100 minus 7 equal to?

IWER: For respondents who are unable to solve complex calculations, mark '0 (incorrect answer)' for questions Cd07a ~ Cd11a. Ask simple questions such as "What is 100 minus 30?" or "What is 10 minus 3?" for about 3 minutes and no longer.

Cd08. And 7 from that?

IWER: Try to persuade if R refuses to answer. Record the exact number R says. Record 0 for 'don't know' or 'refuse to answer'

Cd09. And 7 from that?

IWER: Try to persuade if R refuses to answer. Record the exact number R says. Record 0 for 'don't know' or 'refuse to answer'

Cd10. And 7 from that?

IWER: Try to persuade if R refuses to answer. Record the exact number R says. Record 0 for 'don't know' or 'refuse to answer'

Cd11. And 7 from that?

IWER: Try to persuade if R refuses to answer. Record the exact number R says. Record 0 for 'don't know' or 'refuse to answer'

Cd12. A little while ago, I read you a list of words and you repeated the ones you could remember. Please tell me any of the words that you remember now.

[1] Only one of the three words is correct, in any order
[2] Two of the three words are correct, in any order
[3] All of the three words are correct, in any order
[5] None of the three words is correct

Cd13. IWER: Pointing to item #1 What is this?

IWER: Items can be anything from cell phones, gloves, hats and rings that can be within close reach. The elderly tend to call all types of writing instruments 'pencils' so consider the answer correct if they say a pen as a pencil.

[1] Correct [5] Incorrect

Cd14. IWER: Pointing to item #2 What is this?

IWER: Clocks for watches can be considered as correct.

[1] Correct [5] Incorrect

Cd15. Now you have to exactly repeat what I say. Are you ready? Please listen carefully.

IWER: [1] Tidy up the surroundings. [2] Read the sentences aloud at a slow, steady rate. [3] Except for R having no teeth or problems in oral structure, mark 'incorrect' if the pronunciation is unclear. Points aren't usually given if failing on the first try but succeeded on the second try. If the IWER determines that the respondent was distracted on the first try, get him/her to pay attention and repeat the question. Mark 'correct' if the right answer is given.

[1] Correct [5] Incorrect

Logic : List shows randomly

Sentence 1 : To see is to believe
Sentence 2 : Check the dead fire again
Sentence 3 : The Great Mountain is high but under the sky
Sentence 4 : God watch over our land forever
Sentence 5 : Thousand miles of range and river land

Cd16. IWER holds one A4 paper Now, listen carefully and follow my direction. Are you ready?

When I give you a piece of paper, please turn it over, fold it in half, and give it back to me.

IWER: [1] Tidy up the surroundings (especially the front) so it doesn't interfere with the respondent. [2] Do not repeat the question in the middle of the process. [3] Do not give out the paper in advance. [4] Directions can be repeated if the respondent seems unable to understand the directions or if the respondent asks you to repeat the directions. In this case, the paper first given out should be collected and the process should start over.

[1] One of the turning / folding / returning actions is completed successfully
[2] Two of the turning / folding / returning actions are completed successfully
[3] All of the turning / folding / returning actions are completed successfully
[5] None of the turning / folding / returning actions is completed successfully

Cd17. IWER: Make sure R doesn't see the test paper with the words "Close your eyes" written before asking the question. Make sure that someone doesn't read the sentence to R. I'll show you a sentence. Please read the sentence aloud and act it out.

[1] R completed only one task (either reading or action of closing eyes)
[3] R did both tasks
[5] R did not complete any task

Cd18. IWER: Give R a pen and point to the blank part of the paper. Please write one sentence about how you're feeling today or today's weather.

IWER: Spelling error is OK, as long as you can understand the meanings of the sentence written

[1] Wrote a sentence
[5] Couldn't write a sentence

Cd19. IWER: Show the picture of two pentagons overlapped Do you see this picture? Please draw that picture on this paper.

[1] Drew the picture
[5] Failed to draw the picture

Cd20. IWER: HOW OFTEN DID R RECEIVE ASSISTANCE WITH ANSWERS IN SECTION Cd – COGNITION?

[1] never
[2] a few times
[3] most or all of the time

} → Go to Section Ce.

Cd51. IWER: SECTION Cd – COGNITION CANNOT BE DONE BY A PROXY REPORTER.

[1] CONTINUE → Go to Section Ce.

Ce. GRIP STRENGTH

Ce. GRIP STRENGTH SURVEY STRUCTURE

Ce01-Ce06 : PREPARING FOR GRIP STRENGTH

Ce01 : DOMINANT HAND(RIGHT/LEFT HAND)
Ce02 : RESPONDENT'S APPROVAL FOR MEASURING GRIP STRENGTH
Ce03-Ce04 : MAKE SURE THE SAFETY – SURGERY AND EXPERIENCING SWELLING, SEVERE PAIN OR INJURY, HAND HAVING INJURY OR PAINS
Ce05-Ce06 : THE REASON OF NOT COMPLETING THE GRIP STRENGTH

Ce07-Ce10 : GRASP POWER SCORE

Ce07-Ce08 : RIGHT HAND SCORE
Ce09-Ce10 : LEFT HAND SCORE

Ce11-Ce12 : (INTERVIEWER CONFIRM IT) R'S POSITION, SUPPORTING WHILE PERFORMING TEST

Ce13 : CHECK POINT OF INTERVIEWER

Ce50. Before we go on to the next Section, I like to check if this is a self or proxy interview. Are you R's FIRST, LAST NAME?

[1] Yes
[5] No → Go to Cd51

Ce01. Now I would like to assess the strength of your hand in a gripping action. Which is your dominant hand?

[1] Right hand
[3] Left hand
[5] Both hands equally dominant

Ce02. I will ask you to squeeze this handle as hard as you can, just for a couple of seconds and then let go. I will take alternately two measurements from your right and your left hand. Are you willing to do this test?

[1] Yes
[3] Willing but unable to do the test } → Go to Ce05
[5] No } → Go to Ce05

Ce03. Before we begin, I'd like to make sure it is safe for you to do this test. Have you had any surgery within the last 6 months or experienced any swelling, inflammation, severe pain, or injury in one or both hands that might prevent you from gripping?

[1] No apparent restriction → Go to Ce07
[1] Yes, recent surgery
[2] Yes, sever pain
[3] Yes, swelling or inflammation
[4] Other problems

Ce04. Which hand hurts?

[1] Respondent is unable to use of both hands → Go to Ce05
[3] Respondent is unable to use right hand → Go to Ce09
[5] Respondent is unable to use left hand → Go to Ce07

Ce05. IWER: WHY DIDN'T R COMPLETE THE GRIP STRENGTH TEST? (SELECT ALL THAT APPLY)

[1] R felt it would not be safe
[2] IWER felt it would not be safe
[3] R refused
[4] R tried but was unable to complete test
[5] R did not understand the instructions
[6] R had surgery, injury or swelling on both hands in past 6 months
} → Go to Ce13
[7] Other

Ce06. Please specify other.

__________________________ → Go to Ce13

Ce07. Now, I'm going to measure your RIGHT HAND. Please put your elbow onto your side and make a right angle around your elbow. Make sure the scale of the dynamometer is set to zero. Ready? Squeeze it now. (Unit: Kg.)

FIRST READING _____________

Ce08. Now, I'm going to measure your RIGHT HAND again. Please set the scale of the dynamometer to zero, Squeeze it now. (Unit: Kg.)

SECOND READING _____________

SKIP PATTERN CHECKPOINT: LEFT HAND IF R IS UNABLE TO USE LEFT HAND IN Ce04, GO TO Ce11.

Ce09. Now, I'm going to measure your LEFT HAND, Please put your elbow onto your side and make a right angle around your elbow. Make sure the scale of the dynamometer is set to zero. Ready? Squeeze it now. (Unit: Kg.)

FIRST READING _____________

Ce10. Now, I'm going to measure your LEFT HAND again, Please set the scale of the dynamometer to zero. Squeeze it now. (Unit: Kg.)

SECOND READING _____________

Ce11. IWER: What was the R's position for this test?

[1] standing
[3] sitting
[5] lying down

Ce12. IWER: Did R receive a support while performing this test?

[1] Yes [5] No

Ce13. IWER: How much effort did R give to this measurement?

[1] R gave full effort
[2] R was prevented from giving full effort by illness, pain, symptoms or discomfort
[3] R did not appear to give full effort without an obvious reason
[4] R did not measure for a personal reason

} → Go to Section D.

Ce51. IWER: SECTION Ce – GRIP STRENGTH CANNOT BE DONE BY A PROXY REPORTER.

[1] CONTINUE → Go to Section D.

D. EMPLOYMENT

D. EMPLOYMENT SURVEY STRUCTURE

D001-D010 : CURRENT EMPLOYMENT SITUATION

- WHETHER OR NOT WORK CURRENTLY, MAIN JOB, REASON OF RESTRICTING THE WORK, JOB SEARCHING ACTIVITY, RETIREMENT

D011 : CHECK POINT OF INTERVIEWER

D101-D206 : EMPLOYED

D101-D107 : WORKPLACE - NAME OF WORKPLACE, LOCATION, TYPE OF BUSINESS, NUMBERS OF EMPLOYEE
D108 : DATE OF EMPLOYMENT
D109-D155 : INFORMATION ABOUT JOB
D109-D113 : TYPE OF WORK, POSITION / OFFICIAL TITLE, DATE OF HOLDING THE CURRENT JOB TITLE, NUMBERS OF EMPLOYEE OF UNDER SUPERVISION
D114 : EMPLOYMENT PATTERN - REGULAR/TEMPORARY/CASUAL
D115 : PATTERN OF WORKING HOUR - FULL TIME/PART TIME
D116-D120 : EMPLOYMENT CONTRACT
D121-D124 : EXPECTATION CURRENT JOB - POSSIBILITY OF WORK DURATION, PERIOD OF WORK DURATION
D125 : SOURCE OF WAGE/INCOME
D126-D129 : RETIREMENT / STOPPING THE JOB - MANDATORY RETIREMENT AGE, CUSTOMARY RETIREMENT AGE
D130-D132 : WORKING HOURS, WORKING DAYS
D133-D147 : SUGGESTIONS THE ADJUSTING WORKING INCOME AND HOURS
D148-D151 : REGULAR SCHEDULED OFF-DAY - APPOINTMENT OF REGULAR SCHEDULED OFF-DAY, DAYS OF VACATION/PAID SICK LEAVE, DAYS OF MISSING WORK FOR HEALTH PROBLEM
D152-D154 : METHOD OF WAGE PAYMENT
D155 : AVERAGE MONTHLY WAGE/INCOME
D156-D168 : ENROLMENT OF SOCIAL INSURANCE AND FRINGE BENEFITS
D156-D163 : NATIONAL HEALTH INSURANCE, EMPLOYMENT INSURANCE, WORKER'S INSURANCE, RETIREMENT BENEFITS SYSTEM
D164-D168 : FRINGE BENEFITS - MEAL SUBSIDY, SUBSIDY FOR CHILDREN, MORTGAGE FOR HOME PURCHASE, SUBSIDY FOR LOST WAGES, SUBSIDY FOR PREMIUM OF PERSONAL PENSION
D169-D175 : LABOR UNION
D176-D195 : JOB SATISFACTION
D196-D198 : HOPPING JOB PATTERN
D199 : RETIREMENT PLAN
D200-D205 : ENGAGING IN OTHER JOB OR - NUMBERS OF JOB, REASON OF ENGAGING IN OTHER JOB , WORKING HOURS, WAGE/INCOME
D206 : CHECK POINT OF INTERVIEWER

D. EMPLOYMENT SECTION SURVEY STRUCTURE (Continued)

D301-D362 : SELF-EMPLOYED

- D301-D302 : REASON OF RUNNING OWN'S BUSINESS
- D303-D312 : WORKPLACE - NAME OF WORKPLACE, CO-WORKER, SHARE OF OWNERSHIP, LOCATION, TYPE OF BUSINESS, NUMBERS OF EMPLOYEE
- D313 : DATE OF STARTING BUSINESS
- D314 : TYPE OF WORK
- D315 : WAGE/INCOME
- D316-D317 : WORKING HOURS, WORKING DAYS
- D318-D332 : SUGGESTIONS THE ADJUSTING WORKING INCOME AND HOURS
- D333-D334 : REGULAR OFF-DAY, DAYS OF MISSING WORK FOR HEALTH PROBLEM
- D335-D351 : SUGGESTIONS ABOUT CURRENT JOB AND JOB SATISFACTION
- D352-D354 : HOPPING JOB PATTERN
- D355 : RETIREMENT PLAN
- D356-D361 : ENGAGING IN OTHER JOB OR - NUMBERS OF JOB, REASON OF ENGAGING IN OTHER JOB, WORKING HOURS, WAGE / INCOME
- D362 : CHECK POINT OF INTERVIEWER

D401-D452 : UNPAID FAMILY WORKER

- D401-D402 : REASON OF WORKING ON UNPAID FAMILY WORKER
- D403-D405 : WORKPLACE - NAME OF WORKPLACE, LOCATION, TYPE OF BUSINESS
- D406 : DATE OF STARTING THIS JOB
- D407 : TYPE OF WORK
- D408-D411 : INFORMATION OF EMPLOYEE - NUMBERS OF EMPLOYE, OTHER UNPAID FAMILY WORKER
- D412 : REPRESENTATIVE OF WORKPLACE AND RELATIONSHIP TO RESPONDENT
- D413-D414 : ANNUAL SALES(INCOME)
- D415-D416 : WORKING HOURS , WORKING DAYS
- D417-D425 : SUGGESTIONS ABOUT WORKING HOURS
- D426-D427 : REGULAR OFF-DAY, DAYS OF MISSING WORK FOR HEALTH PROBLEM
- D428-D443 : SUGGESTIONS ABOUT CURRENT JOB AND SATISFACTION
- D444-D446 : JOB IN THE FUTURE - IDEAL EMPLOYMENT PATTERN, TYPE OF BUSINESS, WAGE / INCOME
- D447 : RETIREMENT PLAN
- D448-D451 : ENGAGING IN OTHER JOB OR - NUMBERS OF JOB, REASON OF ENGAGING IN OTHER JOB
- D452 : CHECK POINT OF INTERVIEWER
- D501-D532 : UNEMPLOYED
- D501-D502 : FINDING JOB LAST WEEK/LAST MONTH
- D503-D504 : WILLING TO FINDING WORK, WILLING TO WORK IF THERE IS APPROPRIATE JOB
- D505-D508 : MAIN REASON DO NOT HAVING JOB
- D509-D510 : MAIN REASON LOOKING FOR JOB
- D511-D517 : ACTIVITIES TO FIND A JOB - METHODS TO FIND A JOB, DATE OF STARTING FINDING A JOB, HOURS OF SPENDING JOB HUNTING PER WEEK
- D518-D521 : HOPPING JOB TYPES
- D522-D529 : DIFFICULTY OF FINDING JOB ACTIVITIES
- D530 : PREVIOUS WORK EXPERIENCE
- D532 : CHECK POINT OF INTERVIEWER

D601-D622 : RETIRED

- D601-D608 : INFORMATION OF RETIREMENT
  - D601 : TIME OF RETIREMENT
  - D602-D605 : REASON OF RETIREMENT
  - D606-D608 : SPOUSE'S EMPLOYMENT SITUATION WHEN RESPONDENT WAS RETIRED

D. EMPLOYMENT SECTION SURVEY STRUCTURE (Continued)

D609-D615 : INFORMATION OF PASTIME WORK
-TYPE OF WORK, PLACE OF WORKING, THE DATE OF STARTING WORK, AVERAGE WORKING HOURS PER WEEK, MONTHLY INCOME
D616-D622 : HOPPING JOB FOR PAY REGARDLESS OF RETIREMENT STATUS

D701-D722 : THE LAST JOB

D701 : EMPLOYMENT STATUS
D702-D703 : DAYS OF STARTING AND STOPPING WORK
D704-D709 : INFORMATION ABOUT WORKPLACE - NAME OF WORKPLACE, LOCATION, KIND OF BUSINESS, NUMBERS OF EMPLOYEE
D710 : SORT OF WORK
D711- D712 : TYPE OF WORKING HOUR(FULL TIME/PART TIME), WORKING HOURS PER WEEK
D713 : AVERAGE MONTHLY INCOME
D714-D721 : INFORMATION OF STOP WORKING
- ENROLLMENT OF NATIONAL PUBLIC PENSION SYSTEM, REASON OF STOP WORKING, RETIREMENT BENEFITS
D722 : CHECK POINT OF INTERVIEWER

D001. Are you currently employed? Being employed refers to working for an employer, self-employed, or working for family or relative's business.

[1] Yes
[5] No → Go to D005

D002. We would like to ask about your current primary job. Which of the following best describes your primary job?

[1] I am employed by another person or company and receive a wage. → Go to D101
[2] I am self employed. → Go to D301
[3] I help my family / relative's business without payment for 18 hours or more per week. → Go to D401
[4] I help my family / relative's business without payment for less than 18 hours a week.

D003. What is the reason you work less than 18 hours a week, not more?

[1] Health problems
[2] Childrearing or housework
[3] Studentship
[4] Wanted to take a break
[5] Not enough work to do
} → Go to D005
[6] Other

D004. Please specify other.

D005. Are you looking for a job?

[1] Yes [5] No → Go to D010

D006. Are you able to work if you are offered a job right now?

[1] Yes → D501 [5] No

D007. Why are you unable to work, despite having found a job?

[1] Health problems
[2] Childrearing or housework
[3] Studentship
[4] Wanted to take a break
[5] Business in difficulties
} → Go to D009
[6] Other

D008. Please specify other.

D009. Have you worked for the purpose of earning an income?

[1] Yes → Go to D701 [5] No → Go to D011

D010. What is your current retirement status? Have you worked before but currently retired, or have you worked before and intend to work in the future?

IWER: Retired in this context shall refer to having stopped all income-related activities and presently not working or engaging in small pastime work, and having no intention of engaging in anything more serious than small pastime work.

[1] Worked before but currently retired → Go to D601
[3] Worked before and intend to work in the future but currently not looking for a job → Go to D701
[5] Never had a job before → Go to D011

D011. IWER: HOW OFTEN DID R RECEIVE ASSISTANCE WITH ANSWERS IN SECTION D – EMPLOYMENT?

[1] never
[2] a few times
[3] most or all of the time
} → Go to Section E.
[4] The Section is done by a proxy reporter.

D012. IWER: WHAT IS THE PROXY'S RELATIONSHIP TO R? IF UNKNOWN, PLEASE ASK THE PROXY What is your relationship to R?

[1] Spouse
[2] Mother
[3] Father
[4] Mother-in-law
[5] Father-in-law
[6] Sibling
[7] Brother-in-law, sister-in-law
[8] Child
[9] Spouse of child
[10] Grandchild
[11] Other relative
[12] Helper or other non-relative

→ Go to Section E.

* Employed workers D101~D206 *

D101. What is the (company's / organization's) name you work for? Please state specifically.

IWER: If there is no name, please describe the products or services related to the business.

D102. Where is your work place?

IWER: Press the Enter key then find the location

D103. What kind of business or industry do you work in – that is, what do they make or do at the place where you work?

IWER: Type of business

D104. About how many employees work for this company or organization at all locations? What is the total number of employees including those at the head office, branch office, branches, plants and field?

_____________people (range: 1 ~ 99997) → Go to D106

[98] Don't know
[99] Refuse to answer

D105. If you do not know the exact numbers, which of the following is the closest approximation?

[1] Fewer than 5
[2] 5 to 9 people
[3] 10 to 29 people
[4] 30 to 49 people
[5] 50 to 99 people
[6] 100 to 299 people
[7] 300 to 499 people
[8] 500 to 999 people
[9] More than 1,000 people

D106. About how many employees work for this company / organization at the location where you work?

______________people (range: 1 ~ 9997) → Go to D108
[98] Don't know
[99] Refuse to answer

D107. If you do not know the exact numbers, which of the following is the closest approximation?

[1] Fewer than 5 people
[2] 5 to 9 people
[3] 10 to 29 people
[4] 30 to 49 people
[5] 50 to 99 people
[6] 100 to 299 people
[7] 300 to 499 people
[8] 500 to 999 people
[9] More than 1,000 people

D108. When did you start working for your current company or organization?

IWER: Enter the year and month using 6 digits. For example, mark 200601 for January 2005. If the month is not clear, enter 00

______________ (range: 190000 ~ 200612)

D109. What sort of work do you do now?

IWER: Type of work

____

D110. What is the official title of your job now?

IWER: Mark 0 if there is no official rank or title.

____

D111. Since when have you been holding the current job title?

IWER: Enter the year and month using 6 digits. For example, mark 200601 for January 2005. If the month is not clear, enter 00.

__________________________ (range: 190000 ~ 200612)

D112. Are you at a position to supervise others?

[1] Yes [5] No → Go to D114

D113. How many people are there under your supervision?

[1] fewer than 5 people
[2] 6~10 people
[3] 11~15 people
[4] 16~30 people
[5] 31~99 people
[6] more than 100 people

D114. What kind of appointment do you have? Are you a regular worker, a temporary worker, or a casual worker?

IWER:
- Regular worker: Workers with an employment contract of more than one year or cases in which workers may choose to work as long as one likes without a predefined contract period
- Temporary worker: Workers with an employment contract of more than one month and less than one year or cases in which the work is expected to be completed within one year, although there is no predefined period of the employment contract (Yet, cases in which one works in one company for a long time and/or expects to work continuously in the future, but has a contract of less than one year are considered as temporary workers)
- Casual worker: Workers with an employment contract of less than one month, who are hired and paid on a daily basis, or compensated for the work done in various locations without a designated workplace

[1] Regular worker
[3] Temporary worke
[5] Casual worker

D115. Do you work part time or full time?

IWER:
Part time: Cases in which one works part time or as a side job, works less hours than other employees who perform the same job, or is paid on an hourly basis
Full time: Cases in which one works full working days as opposed to part time

[1] Part time
[5] Full time

D116. Is there an agreed period of employment (labor contract period) or the period in which you can continue to work at your current job?

[1] Defined
[5] Not defined → Go to D119

D117. How long is the defined period of employment?

_________________________ months (range: 1 ~ 60)

D118. Has the current employment contract ever been renewed?

[1] Yes
[5] No
} → Go to D120

D119. If the employment period is not defined, do you work for a few days or a few weeks for as long as there is work available?

IWER: Please answer 'no' if R's work lasts more than one month.

[1] Yes
[5] No

D120. Did you receive a written contract from your current employer?

[1] Yes
[5] No

D121. Do you think you could keep your current job unless the company closes down due to business difficulties or you are laid off?

[1] Yes → Go to D125
[5] No

D122. How long do you expect to work for your current company / organization?

[1] Less than one year
[2] One to two years
[3] Two to three years
[4] More than three years } → Go to D125

D123. Why do you expect so?

[1] Because the predefined contract period will expire
[2] Because typically the contract expires (although there's no written contract)
[3] Because I was hired under the condition that I would resign upon the request of employer
[4] Because the current job / project will be completed
[5] Because the person I am substituting will return to work
[6] Because I can only work during certain seasons
[7] Because I plan to find another job that better suits my job aptitude, ability, and preference
[8] Because I will reach at retirement age set by regulations / practice
[9] Because of family care responsibility, poor health, etc.
} → Go to D125
[10] Other

D124. Please specify other.

D125. Do you receive your wage from the employer you currently work for or from the dispatch / contract company?

IWER: Dispatched Worker can receive wages from either dispatch company or contract company. If R receives wages from dispatch company, and in this case R has to observe the Worker Dispatching Law. If R receives wages from the contract company, R is not obligated to the Worker Dispatching Law.

[1] Employer
[3] Dispatch Company
[5] Contract Company

D126. Is there a mandatory retirement age at your current workplace?

[1] Yes
[5] No → Go to D128

D127. What is the mandatory retirement age?

IWER: If the retirement age is different according to position, ask the retirement age that applies to R.

__________years old (range: 45 ~ 80)

D128. Are there cases where employees retire upon reaching a certain age regardless of regulations?

[1] Yes [5] No → Go to D130

D129. What is the customary retirement age?

__________years old (range: 45~97)

D130. Are there regular work schedule at your current workplace?

[1] Yes [5] No

D131. About how many days a week do you usually work for the business?

__________days (range: 1~7)

D132. About how many hours a week do you work, excluding lunch time?

__________hours (range: 1 ~ 160)

D133. How do you consider working schedule at your current job including regular and extra hours?

[1] Too long
[3] Appropriate → Go to D148
[5] Too short → Go to D141

D134. If you consider it too long, would you like to reduce it even if your earnings were reduced?

[1] Yes [5] No → Go to D148

D135. If you would like shorter working hours, how would you like to reduce?

[1] some decrease in number of hours per week
[5] some % decrease from total working hours → Go to D137

D136. How many hours per week would you reduce?

___________hours / week (range: 1 ~ 997)

D137. What is the % you would reduce out of total working hours?

___________% (range: 1 ~ 997)

D138. If you could reduce working hours as you wished, how much pay cut would you be willing to take?

[1] Cut by small amount per week
[5] Cut by several % out of total wage → D140
[98] Don't know → D148
[99] Refusal to answer → D148

D139. How much pay cut per week can you take? (Unit: 10,000 Korean won / week)

___________MW (range: 1 ~ 9997)

D140. What % from your total wage can be cut?

___________% (range: 1 ~ 9997) → Go to D148

D141. If you consider your work hour is too short, would you like to increase it with the increased payment?

[1] Yes [5] No → Go to D148

D142. If you would like increased payment, how would you like to increase?

[1] Some increase in number of hours per week
[5] Some % increase from total working hours → Go to D144

D143. How many hours per week would you increase?

___________hours / week (range: 1 ~ 997) → Go to D145

D144. What % from your total working hours would you increase?

___________% (range: 1 ~ 997)

D145. If you could increase your working hours, how much increase in wage do you expect?

[1] some amount increase in wage per week
[5] some % increase from total income → Go to D147

D146. How much increase in wage do you expect per week? (Unit: 10,000 Korean won)

___________MW (range: 1 ~ 9997) → Go to D148

D147. What % of total wage has to be increased?

___________% (range: 1 ~ 997)

D148. Do you have regularly scheduled off-day? If you do, how many off-days do you have per month?

[1] None
[2] one day per month
[3] 2~3 days per month
[4] 4~5 days per month
[5] 6~7 days per month
[6] More than eight days per month

D149. Except for national holidays, how many days of paid vacation do you have this year at your current workplace?

IWER: Mark 0 if there is no paid vacation.

___________days (range: 0 ~ 365)

D150. How many days of paid sick leave do you have this year at your current workplace?

___________days (range: 0 ~ 997)

SKIP PATTERN CHECKPOINT: WORK EXPERIENCE IF R STARTED WORKING FOR THE BUSINESS BEFORE JAN 2005 IN D108, GO TO D151. IF NOT, GO TO D152.

D151. How many days of work did you miss last year due to health problems?

IWER: Mark 0 if R didn't miss work.

___________days

D152. How is your wage paid? Is it regularly paid, contract based, performance based, or other? If it is regularly paid, please tell me how often you receive your wages. Do you have yearly contract, monthly, weekly, daily, or hourly? Please select one.

[1] Yearly salary
[2] Monthly salary
[3] Weekly salary
[4] Daily salary
[5] Hourly salary
[6] Contract-based
[7] Performance-based (no basic salary)
→ Go to D154 (for [1]–[7])
[8] Other

D153. Please specify other.

____

D154. Is your wage seniority–based? That is, wage is directly linked to the number of years you have worked?

[1] Yes [5] No

D155. How much are you paid after taxes and other deductions per month? (Unit: 10,000 Korean won)

IWER: Please list all except for the amount deducted such as tax, public pension, medical insurance and employment insurance premium.

_________________________MW (range: 1 ~ 99997)

D156. We are interested in the benefits you receive from your current employer. First, are you currently enrolled in the National Public Pension system? If so, are you enrolled through your current employer? Or, are you enrolled in the Occupational Pension Plan for Public Workers, Teachers, and Soldiers? Or, are you enrolled in National Public Pension System through community?

[1] Yes, enrolled in the National Pension System through current employer
[2] Yes, enrolled in the Occupational Pension Plan (for public servants, teachers and soldiers)
[3] Yes, enrolled in the National Pension System through community
[4] No, not enrolled in the National Pension System

D157. Are you currently enrolled in National Health Insurance through your employer? Or you are currently enrolled in the National Health Insurance, but not through your currently employer? Or are you not currently enrolled in the National Health Insurance?

[1] Yes, enrolled in the National Health Insurance through current employer
[3] Yes, enrolled in the National Health Insurance, but not through current employer
[5] No, not enrolled in the National Health Insurance

D158. Does your employer provide Employment Insurance? Employment insurance provides protection again job loss.

[1] Yes [5] No

D159. Does your employer provide Workers' Insurance? Workers' insurance provides protection against job-related accidents and injury.

[1] Yes [5] No

D160. Are you included in a pension or retirement plan through your current work? If you are, what kinds of retirement plan do you have? Is it under retirement allowance scheme, retirement insurance scheme, or retirement pension scheme?

IWER:
- Retirement Allowance Scheme: A retiree will receive a lump-sum payment at the time of separation from work.
- Retirement Insurance scheme: An employer puts aside retirement funds in an insurance company (outside of the employer). Insurance company will then pay the retirees the benefits either in the form of annuity payments of lump-sum payment.
- Retirement Pension scheme: A retiree will receive annuity payments after the retirement age of 55.

[1] Yes, Retirement Allowance Scheme
[2] Yes, Retirement Insurance Scheme
[3] Yes, Retirement Pension Scheme → Go to D163
[4] No, no retirement plan → Go to D164

D161. Have you ever taken out some money from your retirement savings from your current workplace?

[1] Yes [5] No → Go to D164

D162. In what year and month, did you take out the money from the retirement savings?

IWER: Mark the year and month in 6 digits. For example, mark 200601 for January 2006. If the month is not clear, enter 00.

_________________________ (range: 190000 ~ 200612) → Go to D164

D163. Now, I would like to know what type of plan this is. In the defined benefits plan, benefits are pre-determined, usually based on a formula involving age, years of service and salary. In the defined contribution plans, money is accumulated as employer makes pre-determined amount of contribution. Is your plan Defined Benefits or Defined Contributions?

[1] Defined Benefits (DB)
[5] Defined Contributions (DC)

D164. The following are fringe benefits which may be provided by a company. Please answer if the following is provided by your current workplace. Do you get a meal subsidy?

[1] Yes [5] No

D165. Does your employer provide an education subsidy for children?

[1] Yes [5] No

D166. Does your employer provide a mortgage for home purchase?

[1] Yes [5] No

D167. Does your employer provide some subsidy for lost wages in the case of a union strike?

[1] Yes [5] No

D168. Does your employer pay for insurance premium of your personal pension?

IWER: Personal pension refers to privately owned retirement savings, not including National Public Pension.

[1] Yes [5] No

D169. Is there a labor union at your current workplace?

[1] Yes [5] No → Go to D175

D170. Are you qualified to join a labor union?

[1] Yes [5] No → Go to D175

D171. Did you join the labor union?

[1] Yes [5] No → Go to D173

D172. If you are a member, was membership mandatory?

[1] Yes
[5] No
} → Go to D176

D173. If you are not a member, why did you not become a member?

[1] Not satisfied with the union activities
[2] Dissuaded by family, friends, colleagues, etc.
[3] Dissuaded by employer
[4] Did not feel the need
} → Go to D176
[5] Other

D174. Please specify other.

____

D175. Would you become a member if one existed?

[1] Yes [5] No

D176. Please answer to what extent the following applies to your current job. Please tell me whether you strongly agree, agree, disagree, or strongly disagree.
My job requires lots of physical effort.

[1] Strongly agree
[2] Agree
[3] Disagree
[4] Strongly disagree

D177. My job requires lifting heavy loads.
[1] Strongly agree
[2] Agree
[3] Disagree
[4] Strongly disagree

D178. My job requires stooping, kneeling, or crouching.
[1] Strongly agree
[2] Agree
[3] Disagree
[4] Strongly disagree

D179. My job requires good eyesight.
[1] Strongly agree
[2] Agree
[3] Disagree
[4] Strongly disagree

D180. My job requires intense concentration and attention to details.
[1] Strongly agree
[2] Agree
[3] Disagree
[4] Strongly disagree

D181. My job requires skill in dealing with people.
[1] Strongly agree
[2] Agree
[3] Disagree
[4] Strongly disagree

D182. My job requires me to work with computers.
[1] Strongly agree
[2] Agree
[3] Disagree
[4] Strongly disagree

D183. My job requires me to do more challenging tasks than it used to.
[1] Strongly agree
[2] Agree
[3] Disagree
[4] Strongly disagree

D184. In promotion, seniority is important at my company.
[1] Strongly agree
[2] Agree
[3] Disagree
[4] Strongly disagree

D185. My co-workers make older workers feel that they ought to retire before the retirement age.
[1] Strongly agree
[2] Agree
[3] Disagree
[4] Strongly disagree

D186. My employer would let older workers move to less demanding job with less pay if they wanted to.
[1] Strongly agree
[2] Agree
[3] Disagree
[4] Strongly disagree

D187. My salary is adequate.
[1] Strongly agree
[2] Agree
[3] Disagree
[4] Strongly disagree

D188. My job security is good.
[1] Strongly agree
[2] Agree
[3] Disagree
[4] Strongly disagree

D189. I am satisfied with the work environment of my job.
[1] Strongly agree
[2] Agree
[3] Disagree
[4] Strongly disagree

D190. I am satisfied with the work I do at my current job.
[1] Strongly agree
[2] Agree
[3] Disagree
[4] Strongly disagree

D191. My job involves a lot of stress.
[1] Strongly agree
[2] Agree
[3] Disagree
[4] Strongly disagree

D192. All things considered, I am satisfied with my current job.
[1] Strongly agree
[2] Agree
[3] Disagree
[4] Strongly disagree

D193. Considering my educational attainment, the level of my current job is
[1] Very low
[2] Low
[3] Appropriate
[4] High
[5] Very high

D194. Considering my skills and expertise, the level of my current job is
[1] Very low
[2] Low
[3] Appropriate
[4] High
[5] Very high

D195. Do you plan to continue working at your current job? Please tell me if you want to stay at the current job, switch to a different job, or quit the job. If you want to stay at the current job, do you want to keep the same level of work load, do you want work more, add another job, or do you want work less?

[1] Yes, I plan to continue my current job, as it is → D199
[2] Yes, I plan to continue my current job, but want to increase work load → D199
[3] Yes, I plan to continue my current job, but like to add another job
[4] Yes, I plan to continue my current job, but like to work less → D199
[5] No, I like to take a different job
[6] No, I like to stop working altogether → D199

D196. What would be the ideal employment status for you?

[1] Full time wage worker
[3] Part time wage worker
[5] Self – employed

D197. What do you want to do for this new job? Please describe specifically.

D198. What would be the minimum wage or salary per month? (Unit: 10,000 Korean won)

IWER: Mark 0 if R says the salary doesn't matter.

_________________________MW (range: 0 ~ 99997)

D199. Now I want to ask about your retirement plans. At what age do you plan to retire? By retirement, I mean you stop working all together or do only small pastime work.

IWER: Mark 0 if R plans to keep working as long as he/she is physically capable.

_________________________years old (range: 0 ~ 97)

D200. Are you engaged in other job(s) than the above-mentioned main job?

IWER: Only include paid jobs. Activities without pay such as voluntary work are not included.

[1] Yes [5] No → Go to D206

D201. How many jobs do you currently have including the main job? (Unit: Number)

_________________________ (range: 2 ~ 97)

D202. What are the reasons for having other job(s) than your main job?

IWER: Please state the most important reason only.

[1] To earn more money
[2] To help society or others
[3] For my health
[4] Because it is the work I wanted to do
[5] For self-development
[6] To utilize spare time
[7] To prepare myself when I quit my main job
} → Go to D204
[8] Other

D203. Please specify other.

____

D204. How many hours a week do you usually work on this other job / these other jobs? (Unit: weekly hours)

_________________________hours (range: 0 ~ 97)

D205. About how much per month do you earn from this other job / these other jobs? (Unit: 10,000 Korean won)

_________________________MW (range: 1 ~ 99997)

D206. IWER: HOW OFTEN DID R RECEIVE ASSISTANCE WITH ANSWERS IN SECTION D－EMPLOYMENT?

[1] Never
[2] A few times
[3] Most or all the time
} → Go to Section E.
[4] The Section was done by a proxy reporter.

D207. IWER : WHAT IS THE PROXY'S RELATIONSHIP TO R? IF UNKNOWN, PLEASE ASK THE PROXY. What is your relationship to R?

[1] Spouse
[2] Mother
[3] Father
[4] Mother-in-law
[5] Father-in-law
[6] Sibling
[7] Brother-in-law, sister-in-law
[8] Child
[9] Spouse of child
[10] Grandchild
[11] Other relative
[12] Helper or other non-relative
→ Go to Section E.

* Self－employed workers D301~D362 *

D301. What is the most important reason for you to run your own business, rather than getting a wage job? Please choose the most important reason.

[1] Because I can earn more money
[2] Because it is the work I wanted to do
[3] Because it gives me more flexibility
[4] Because of tax benefits
[5] Because I couldn't get a wage job I wanted (e.g., inadequate salary, work environment, etc)
[6] Because it was difficult to get a wage job
([1]–[6] → Go to D303)
[7] Other

D302. Please specify other.

D303. What is the (company's / organization's) name you run?

IWER: Mark 0 if there is no name.

D304. Do you have any business partners?

[1] Yes
[5] No → Go to D307

D305. How many partners do you have?

_________________________people (range: 1 ~ 97)

D306. What is your share of ownership in this business?

_________________________% (range: 0 ~ 997)

D307. Where is the place? Please tell me the address.

____

D308. Which industry do you work in – that is, what does your company do or make?

IWER: Type of business the company does

____

D309. Does your company have any paid employees? Please include family workers if they are paid in salaries/wages.

[1] Yes [5] No → Go to D312

D310. How many employees are on the payroll? Please exclude those temporarily hired.

IWER: Mark 0 if there are no employees.

_______________people (range: 0 ~ 99997) → Go to D312

[98] Don't know

[99] Refuse to answer

D311. Of the following, what would be the closest approximation?

[1] Fewer than 5 people
[2] 5~9 people
[3] 10~29 people
[4] 30~49 people
[5] 50~99 people
[6] 100~299 people
[7] 300~499 people
[8] 500~999 people
[9] More than 1,000 people

D312. Do you have any family members, relatives or friends who work for your business without a pay? If so, how many?

IWER: Mark 0 if there is none.

_________________________people (range: 0 ~ 997)

D313. In what year and month did you start working for this business?

IWER: Mark the year and month in 6 digits. For example, mark 200601 for January 2006. If the month is not clear, enter 00.

_________________________ (range: 190000 ~ 200612)

D314. What sort of work do you do in your business?

____

D315. How much profit per month do you earn from this business? Please state the net profits excluding expenses. (Unit: 10,000 Korean won)

IWER: Mark 0 if there is no net income, and mark 999997 if running a deficit.

_________________________MW (range: 0 ~ 999997)

D316. How many days per week do you work?

_________________________days (range: 1 ~ 7)

D317. How many hours per week do you work excluding lunch time, break, etc.?

_________________________hours (range: 1 ~ 160)

D318. Do you think of your work hour? Is it too long, appropriate, or too short?

[1] Too long
[3] Appropriate → Go to D333
[5] Too short → Go to D326

D319. If you consider it too long, would you like to reduce it even if your earnings were reduced?

[1] Yes [5] No → Go to D333

D320. If you would like shorter working hours, how much do you like to cut?

[1] some decrease in number of hours per week
[5] some % decrease from total working hour → Go to D322

D321. How many hours per week would you reduce?

_________________________hours (range: 1~997) → Go to D323

D322. What % of total working hour would you reduce?

_________________________% (range: 1 ~ 997)

D323. If you could reduce working hours as you wished, how much pay cut would you willing to take?

[1] Cut by small amount per week
[5] Cut by several % of the total income → Go to D325

D324. How much pay cut per week can you take? (Unit: 10,000 Korean won / week)

_________________________MW (range: 1 ~ 9997) → Go to D333

D325. What % from your total income can be cut?

_________________________% (range: 1 ~ 997) → Go to D333

D326. If you consider your work hour is too short, would you like to increase it with the increased payment?

[1] Yes [5] No → Go to D333

D327. If you would like increased payment, how would you like to increase?

[1] Some increase in number of hours per week → Go to D328
[5] Some % increase of the total working hours → Go to D329

D328. How many hours per week would you increase?

_________________________hours / week (range: 1 ~ 997)

D329. What % from your total working hour would you increase?

_________________________% (range: 1 ~ 997)

D330. If you could increase your working hours, how much increase in income do you expect?

[1] about small amount per week
[5] about several % of the total income → Go to D332

D331. How much income increase per week would have to be? (Unit: 10,000 Korean won)

_________________________MW (range: 1 ~ 9997) → Go to D333

D332. What % of total income has to be increased?

_________________________% (range: 1 ~ 997)

D333. Do you have regularly scheduled off-day? If so, how many days per month?

[1] None
[2] One day per month
[3] 2~3 days per month
[4] 4~5 days per month
[5] 6~7 days per month
[6] More than eight per month

SKIP PATTERN CHECKPOINT : WORK EXPERIENCE
- IF R STARTED WORKING FOR THE BUSINESS BEFORE JAN 2005 IN D313, GO TO D334.
- IF NOT, GO TO D335.

D334. How many days of work did you miss last year due to health problems?

IWER: Mark 0 if R didn't miss any work days.

__________________________days (range: 0 ~ 365)

D335. Please answer to what extent the following applies to your current job. Please tell me whether you strongly agree, agree, disagree, or strongly disagree.
My job requires lots of physical effort.

[1] Strongly agree
[2] Agree
[3] Disagree
[4] Strongly disagree

D336. My job requires lifting heavy loads.

[1] Strongly agree
[2] Agree
[3] Disagree
[4] Strongly disagree

D337. My job requires stooping, kneeling, or crouching.

[1] Strongly agree
[2] Agree
[3] Disagree
[4] Strongly disagree

D338. My job requires good eyesight.

[1] Strongly agree
[2] Agree
[3] Disagree
[4] Strongly disagree

D339. My job requires intense concentration and attention to details.
[1] Strongly agree
[2] Agree
[3] Disagree
[4] Strongly disagree

D340. My job requires skill in dealing with people.
[1] Strongly agree
[2] Agree
[3] Disagree
[4] Strongly disagree

D341. My job requires me to work with computers.
[1] Strongly agree
[2] Agree
[3] Disagree
[4] Strongly disagree

D342. My job requires me to do more challenging tasks than it used to.
[1] Strongly agree
[2] Agree
[3] Disagree
[4] Strongly disagree

D343. My salary is adequate.
[1] Strongly agree
[2] Agree
[3] Disagree
[4] Strongly disagree

D344. My job security is good.
[1] Strongly agree
[2] Agree
[3] Disagree
[4] Strongly disagree

D345. I am satisfied with the work environment of my job.

[1] Strongly agree
[2] Agree
[3] Disagree
[4] Strongly disagree

D346. I am satisfied with the work I do at my current job.

[1] Strongly agree
[2] Agree
[3] Disagree
[4] Strongly disagree

D347. My job involves a lot of stress.

[1] Strongly agree
[2] Agree
[3] Disagree
[4] Strongly disagree

D348. All things considered, I am satisfied with my current job.

[1] Strongly agree
[2] Agree
[3] Disagree
[4] Strongly disagree

D349. Considering my educational attainment, the level of my current job is

[1] Very low
[2] Low
[3] Appropriate
[4] High
[5] Very high

D350. Considering my skills and expertise, the level of my current job is

[1] Very low
[2] Low
[3] Appropriate
[4] High
[5] Very high

D351. Do you plan to continue working at your current job? Please tell me if you want to stay at the current job, switch to a different job, or quit the job. If you want to stay at the current job, do you want to keep the same level of work load, do you want work more, add another job, or do you want work less?

[1] Yes, I plan to continue my current job, as it is → D355
[2] Yes, I plan to continue my current job, but want to increase work load → D355
[3] Yes, I plan to continue my current job, but like to add another job
[4] Yes, I plan to continue my current job, but like to work less → D355
[5] No, I like to take a different job.
[6] No, I like to stop working altogether → D355

D352. What would be the ideal employment status for your new job?

[1] Full time wage worker
[3] Part time wage worker
[5] Self-employed

D353. What do you want to do for your new job? Please describe specifically.

____

D354. What would be the minimum wage or income per month? (Unit:10,000 Korean won)

IWER: Mark 0 if R says the salary doesn't matter.

_________________________MW (range: 0 ~ 99997)

D355. Now I want to ask about your retirement plans. At what age do you plan to retire? By retirement, I mean you stop working all together or do only small pastime work.

IWER: Mark 0 if R plans to keep working as long as he/she is physically capable.

_________________________years old (range: 0 ~ 97)

D356. Are you engaged in other job(s) than the above-mentioned main job?

IWER: Only include paid jobs. Activities without pay such as voluntary works are not included.

[1] Yes [5] No → Go to D362

D357. How many jobs do you have including your main job?

__________ (range: 2 ~ 97)

D358. What are the reasons for doing other job(s) than your main job? Please state the most important reason only.

[1] Because the income from my main job is insufficient
[2] To earn pocket money
[3] To help society or others
[4] For my health
[5] For self-development
[6] To utilize spare time
→ Go to D360 (options 1–6)
[7] Other

D359. Please specify.

____

D360. How many hours per week do you usually work on this other job/these other jobs?

__________ hours (range: 0 ~ 97)

D361. About how much per month do you earn from this other job/these other jobs? Unit

__________ MW

D362. IWER: HOW OFTEN DID R RECEIVE ASSISTANCE WITH ANSWERS IN SECTION

[1] Never
[2] A few times
[3] Most or all the time
[4] The Section was done by a proxy reporter.

D363. IWER: WHAT IS THE PROXY'S RELATIONSHIP TO R? I What is your relationship to R?

[1] Spouse
[2] Mother
[3] Father
[4] Mother-in-law
[5] Father-in-law
[6] Sibling
[7] Brother-in-law, sister-in-law
[8] Child
[9] Spouse of child
[10] Grandchild
[11] Other relative
[12] Helper or other non-relative

＊ Non－wage but working in family enterprise D401-D453 ＊

D401. What is the reason you didn't get a wage job and chose to work on the current job? Please state the most important reason only.

[1] Because I can earn more money
[2] Because it is the work I wanted to do
[3] Because it gives me more flexibility
[4] Because I couldn't get the job I wanted (e.g., salary and work environment)
[5] Because it was difficult to get a wage job
→ [1]–[5] Go to D403
[6] Other

D402. Please specify other.

____

D403. What is the (company's / organization's) name you work for? Please state specifically.

IWER: Mark 0 if there is no name.

____

D404. Where is your work place?

IWER: Press the Enter key then find the location

D405. What kind of business or industry do you work in－that is, what do they make or do at the place where you work?

____

D406. In which year and month did you start working for your current company or organization?

IWER: Mark the year and month in 6 digits. For example, mark 200601 for January 2006. If the month is not clear, enter 00.

_________________________ (range: 190000 ~ 200612)

D407. What sort of work do you do now?

____

D408. Are there any paid employees in your current workplace? Please include family or relatives if they are regularly paid.

[1] Yes [5] No → Go to D411

D409. About how many employees work for this company or organization, excluding those temporarily hired?

__________people (range: 0 ~ 99997) → Go to D411

[98] Don't know
[99] Refuse to answer } → Go to D410

D410. Of the following, which one is the closest approximation?

[1] Fewer than 5 people
[2] 5~9 people
[3] 10~29 people
[4] 30~49 people
[5] 50~99 people
[6] 100~299 people
[7] 300~499 people
[8] 500~999 people
[9] More than 1,000 people

D411. Are there any other family, relatives or friends who work more than 18 hours a week without being paid in your workplace? How many non-paid workers are there including yourself?

IWER: family or relatives, including R, who are working without payment.

__________people (range: 1 ~ 97)

D412. Who is the representative of your current workplace? What is his / her relationship to you? For example, he is your father.

____

D413. How much was the last calendar year's annual sales? In case of agricultural and fisheries industry, please include net income (including expenses) from sales of agricultural and fisheries' products. (Unit: 10,000 Korean won)

__________MW → Go to D415

[98] Don't know
[99] Refuse to answer } → Go to D414

D414. Of the following, which number is the closest approximation to annual sales figure?
[1] Less than 1,000 MW (10,000 Korean won)
[2] 1,000~3,000 MW (10,000 Korean won)
[3] 3,000~5,000 MW (10,000 Korean won)
[4] 5,000~8,000 MW (10,000 Korean won)
[5] 8,000~10,000 MW (10,000 Korean won)
[6] 10,000~30,000 MW (10,000 Korean won)
[7] 30,000~50,000 MW (10,000 Korean won)
[8] 50,000~100,000 MW (10,000 Korean won)
[9] More than 100,000 MW (10,000 Korean won)

D415. About how many hours per week do you work, excluding lunch time, break time, etc.?

__________ hours / week (range: 18 ~ 997)

D416. About how many days per week do you work for this business?

__________ days / week (range: 1 ~ 7)

D417. How do you consider your work schedule is?
[1] Too long
[3] Appropriate → Go to D426
[5] Too short → Go to D422

D418. If you consider it too long, would you like to reduce working hours?
[1] Yes [5] No → Go to D426

D419. If you would like to reduce, how much of a reduction would you like?
[1] Some decrease in number of hours per week
[5] Some % decrease from the total working hours → Go to D421

D420. How many hours per week would you reduce?

__________ hours / week (range: 1 ~ 997) → Go to D426

D421. What is the % you would reduce out of total working hours?

__________% (range: 1 ~ 997) → Go to D426

D422. If you consider your work schedule too short, would you like to increase it?

[1] Yes [5] No → Go to D426

D423. If you would like to increase it, how much?

[1] Would like to increase a few hours per week
[5] Would like to increase % out of total working hours → Go to D425

D424. How many hours per week would you like to increase?

__________hours (range: 1 ~ 997) → Go to D426

D425. What % from your total working hours would you like to increase?

__________% (range: 1~997)

D426. Do you have a regularly scheduled off-day? If so, which day(s) of month is / are your off – days?

[1] Yes, every Saturday and Sunday
[2] Yes, every Sunday and three Saturdays per month
[3] Yes, every Sunday and two Saturdays per month
[4] Yes, every Sunday and one Saturday per month
[5] Yes, one day of the week excluding Sunday
[6] Yes, every Sunday
[7] No, I don't have a set schedule
[8] No, I don't take a day off

D427. How many days of work did you miss last year(2005) due to health problems?

__________days (range: 0 ~ 365)

D428. Please answer to what extent the following applies to your current job. Please tell me whether you strongly agree, agree, disagree, or strongly disagree.
My job requires lots of physical effort.

[1] Strongly agree
[2] Agree
[3] Disagree
[4] Strongly disagree

D429. My job requires lifting heavy loads.

[1] Strongly agree
[2] Agree
[3] Disagree
[4] Strongly disagree

D430. My job requires stooping, kneeling, or crouching.

[1] Strongly agree
[2] Agree
[3] Disagree
[4] Strongly disagree

D431. My job requires good eyesight.

[1] Strongly agree
[2] Agree
[3] Disagree
[4] Strongly disagree

D432. My job requires intense concentration and attention to details.

[1] Strongly agree
[2] Agree
[3] Disagree
[4] Strongly disagree

D433. My job requires skill in dealing with people.

[1] Strongly agree
[2] Agree
[3] Disagree
[4] Strongly disagree

D434. My job requires me to work with computers.
[1] Strongly agree
[2] Agree
[3] Disagree
[4] Strongly disagree

D435. My job requires me to do more challenging tasks than it used to.
[1] Strongly agree
[2] Agree
[3] Disagree
[4] Strongly disagree

D436. I am satisfied with the work environment of my job.
[1] Strongly agree
[2] Agree
[3] Disagree
[4] Strongly disagree

D437. I am satisfied with the work I do at my current job.
[1] Strongly agree
[2] Agree
[3] Disagree
[4] Strongly disagree

D438. My job involves a lot of stress.
[1] Strongly agree
[2] Agree
[3] Disagree
[4] Strongly disagree

D439. All things considered, I am satisfied with my current job.
[1] Strongly agree
[2] Agree
[3] Disagree
[4] Strongly disagree

D440. How helpful do you think your working as an unpaid family worker to your household income?

[1] Very helpful
[2] Somewhat helpful
[3] Fair
[4] Not helpful
[5] Not at all helpful

D441. Considering my educational attainment, the level of my current job is

[1] Very helpful
[2] Somewhat helpful
[3] Fair
[4] Not helpful
[5] Not at all helpful

D442. Considering my skills and expertise, the level of my current job is

[1] Very helpful
[2] Somewhat helpful
[3] Fair
[4] Not helpful
[5] Not at all helpful

D443. Do you plan to continue working at your current job? Please tell me if you want to stay at the current job, switch to a different job, or quit the job. If you want to stay at the current job, do you want to keep the same level of work load, do you want work more, add another job, or do you want work less?

[1] Yes, I plan to continue my current job, as it is → Go to D447
[2] Yes, I plan to continue my current job, but want to increase work load → Go to D447
[3] Yes, I plan to continue my current job, but like to add another job
[4] Yes, I plan to continue my current job, but like to work less → Go to D447
[5] No, I like to take a different job
[6] No, I like to stop working altogether → Go to D447
[98] Don't know → Go to D447
[99] Refuse to answer → Go to D447

D444. What would be the ideal employment status for your new job?

[1] Full time wage worker
[3] Part time wage worker
[5] Self – employed

D445. What do you want to do for your new job? Please describe specifically.

D446. What would be the minimum wage or income per month for this job?

IWER: Mark 0 if R says the salary doesn't matter.

-------------------------- (range: 0~99997)

D447. Now I want to ask about your retirement plans. At what age do you plan to retire? By retirement, I mean you stop working all together or do only small pastime work.

IWER: Mark 0 if R plans to keep working as long as he/she is physically capable.

_____________years old (range: 0 ~ 97)

D448. Are you engaged in other job(s) than the above-mentioned main job? Include paid jobs only. Activities without pay such as voluntary work are not included.

[1] Yes [5] No → Go to D452

D449. How many jobs do you have including your main job? Please include paid work only.

_____________ (range: 2 ~ 97)

D450. What are the reasons for having side job(s)? Please state the most important reason only.

[1] Because income from the main job is insufficient
[2] To earn pocket money
[3] To help society or others
[4] For my health
[5] For self-development
[6] To utilize spare time

} → Go to D452

[7] Other

D451. Please specify other.

D452. IWER: HOW OFTEN DID R RECEIVE ASSISTANCE WITH ANSWERS IN SECTION D – EMPLOYMENT?

[1] Never
[2] A few times
[3] Most or all of the times
} → Go to Section E.
[4] The Section was done by a proxy reporter

D453. IWER: WHAT IS THE PROXY'S RELATIONSHIP TO R? IF UNKNOWN, PLEASE ASK THE PROXY. What is your relationship to R?

[1] Spouse
[2] Mother
[3] Father
[4] Mother-in-law
[5] Father-in-law
[6] Sibling
[7] Brother-in-law, sister-in-law
[8] Child
[9] Spouse of child
[10] Grandchild
[11] Other relative
[12] Helper or other non-relative
→ Go to Section E.

* Unemployed D501-D533 *

D501. Have you been doing anything to find work last week?

[1] Yes → Go to D503 [5] No

D502. Have you been doing anything to find work during the last four weeks?

[1] Yes [5] No → Go to D504

D503. Would you have been able to work last week if there was an appropriate job or work?

[1] Yes → D509 [5] No → Go to D507

D504. Would you be willing to work if there is an appropriate job?

[1] Yes [5] No → Go to D507

D505. What is the main reason you were not looking for work although you like to get a job?

[1] I believe nothing available for my major or professional background
[2] I believe nothing available with my desired wage level or working condition
[3] I don't believe I can find work
[4] I don't have needed skills, education or other capabilities
[5] I have family / child responsibilities
[6] I have to take care of the household chores
[7] Because of poor health or disability
([1]–[7] → Go to D701)
[8] Other

D506. Please specify other.

____________________________ → Go to D701

D507. What is the main reason you are limiting the work to less than 18 hours per week?

[1] Informal care responsibilities
[2] Housework
[3] No financial need
[4] Old age
[5] Health problems
[6] Wish to take a break
([1]–[6] → Go to D701)
[7] Other

D508. Please specify other.

____________________________ → Go to D701

D509. What is the main reason you are looking for a job?

[1] To earn living expenses
[2] Due to the unemployment of spouse / family member
[3] To earn pocket money
[4] For self-development
[5] To utilize spare time
([1]–[5] → Go to D511)
[6] Other

D510. Please specify other.

____

D511. What are the two most important activities you are currently engaged in to find a job? First: ______

[1] Working with public employment agency
[2] Working with private staffing agency
[3] Search through advertisements in newspapers, TV and billboards
[4] Search through the Internet
[5] Asking friends, family, or relatives
→ Go to D513 (for 1–5)
[6] Other

D512. Please specify other.

____

D513. What is the second most important things you are doing to find work? Second: ______

[1] Working with public employment agency
[2] Working with private staffing agency
[3] Search through advertisements in newspapers, TV and billboards
[4] Search through the Internet
[5] Asking friends, family or relatives
→ Go to D515 (for 1–5)
[6] Other

D514. Please specify other.

____

D515. Since when have you been job-hunting? Please specify the year and month.

IWER: Mark the year and month in 6 digits. For example, mark 200601 for January 2006. If the month is not clear, enter 00.

____________________________ (range: 190000~200612)

D516. How many hours per week do you usually spend on job-hunting?

IWER: Mark 1 for less than 1 hour.

___________hours per week (range: 1 ~ 997)

D517. Specifically, how many time did you submit a resume, talk to potential employer, and engage in other specific job searching activities since you started looking for a job?

___________times (range: 0 ~ 97)

D518. Where would you like to work? Please state the type of job / business you are interested in.

____

D519. What do you want to do for your new job? Please describe specifically.

____

D520. Do you like to work part-time, full-time, or self-employed?

[1] Full time wage worker
[3] Part time wage worker
[5] Self-employed

D521. What would be the minimum wage or income per month for this job? (Unit: 10,000 Korean Won / month)

___________MW (range: 0 ~ 99997)

D522. The following are common difficulties in finding jobs. To what extent do you agree to the following difficulties?
There is a shortage of jobs.

[1] Strongly agree
[2] Agree
[3] Fair
[4] Disagree
[5] Strongly disagree

D523. There is not enough recruitment information around.

[1] Strongly agree
[2] Agree
[3] Fair
[4] Disagree
[5] Strongly disagree

D524. I don't have needed education, skills or expertise.
[1] Strongly agree
[2] Agree
[3] Fair
[4] Disagree
[5] Strongly disagree

D525. I don't have needed experience.
[1] Strongly agree
[2] Agree
[3] Fair
[4] Disagree
[5] Strongly disagree

D526. The wage offered is too low.
[1] Strongly agree
[2] Agree
[3] Fair
[4] Disagree
[5] Strongly disagree

D527. The working environment and hours are not suitable.
[1] Strongly agree
[2] Agree
[3] Fair
[4] Disagree
[5] Strongly disagree

D528. Old age makes employment difficult.
[1] Strongly agree
[2] Agree
[3] Fair
[4] Disagree
[5] Strongly disagree

D529. ASK THIS QUESTION ONLY IF R IS FEMALE Being a woman makes employment difficult.
[1] Strongly agree
[2] Agree
[3] Fair
[4] Disagree
[5] Strongly disagree

D530. Now I want to ask about your retirement plans. At what age do you plan to retire? By retirement, I mean you stop working all together or do only small pastime work.

IWER: Mark 0 if R plans to keep working as long as he/she is physically capable

___________years old (range: 0 ~ 97)

D531. Have you ever worked as a wage earner, run your own business or helped your family or relatives by working more than 18 hours per week without being paid?

[1] Yes → Go to D701 [5] No

D532. IWER: HOW OFTEN DID R RECEIVE ASSISTANCE WITH ANSWERS IN SECTION D – EMPLOYMENT?

[1] Never
[2] A few times
[3] Most or all of the times
} → Go to Section E.
[4] The Section was done by a proxy reporter.

D533. IWER: WHAT IS THE PROXY'S RELATIONSHIP TO R? IF UNKNOWN, PLEASE ASK THE PROXY. What is your relationship to R?

[1] Spouse
[2] Mother
[3] Father
[4] Mother-in-law
[5] Father-in-law
[6] Sibling
[7] Brother-in-law, sister-in-law
[8] Child
[9] Spouse of child
[10] Grandchild
[11] Other relative
[12] Helper or other non-relative
→ Go to Section E.

* Retired D601-D622 *

D601. In what year and month did you retire?

IWER: Mark the year and month in 6 digits. For example, mark 200601 for January 2006. If the month is not clear, enter 00.

________________________ (range: 190000 ~ 200612)

D602. What was the most important reason why you retired?

[1] Having enough income to get by
[2] Having enough income from spouse
[3] Didn't like to continue to work
[4] To spend more time on leisure
[5] To do volunteer work or to pursue hobby
[6] Due to poor health
[7] Due to the poor health of spouse
[8] Due to health issues of other family members
[9] Due to childrearing or housekeeping
[10] Could not find another job
[11] Mandatory retirement
→ Go to D604 (for [1]–[11])
[12] Other

D603. Please specify other.

D604. What was the second most important reason why you retired?
Second: ______

[1] Having enough income to get by
[2] Having enough income from spouse
[3] Didn't like to continue to work
[4] To spend more time on leisure
[5] To do volunteer work or to pursue hobby
[6] Due to poor health
[7] Due to the poor health of spouse
[8] Due to health issues of other family members
[9] Due to childrearing or housekeeping
[10] Could not find other work
→ Go to D606 (for [1]–[10])
[11] Other

D605. Please specify other.?

D606. Did you have a spouse when you retired?

[1] Yes   [5] No → Go to D609

D607. Did your spouse work at the time of your retirement? Were your spouse employed, self-employed, working for family or relative without a pay for more than or less than 18 hours, unemployed and looking for a job, unemployed but not looking for a job, or retired?

[1] Employed and received a wage
[2] Running his/her own business
[3] Helping family or relative's business without pay for more than 18 hours a week
[4] Helping family or relative's business without pay for less than 18 hours a weekL
[5] Unemployed and looking for a job
[6] Unemployed and not looking for a job
[7] Retired
} → Go to D609
[8] Other

D608. Please specify 'other'.

____

D609. Are you currently working for pay?

[1] Yes [5] No → Go to D616

D610. You have answered that you have pastime job. What kind of job do you have?

____

D611. Where is your work place?

IWER: Press the Enter key then find the location

____

D612. Since when have you worked on this job?

IWER: Mark the year and month in 6 digits. For example, mark 200601 for January 2006. If the month is not clear, enter 00.

_________________________ (range: 190000 ~ 200612)

D613. How many days per week do you usually work for your pastime job?

An average of __________ days per week (range: 0 ~ 7)

D614. How many hours per week do you usually work at your pastime job?

An average of ___________ hours per week (range: 0 ~ 97)

D615. What is your monthly income from the pastime work? (Unit: 10,000 Korean won)

IWER: Mark 0 if there is no net income, and mark 999997 if running a deficit.

___________MW (range: 0 ~ 999997)

D616. Do you want to work for pay regardless of retirement status?

[1] Yes [5] No → Go to D621

D617. What are the reasons?

[1] I want to keep working until my health allows me to
[2] I need the extra money/want to help family finance
[3] Society still needs my knowledge/expertise
[4] To maintain good health both physically and psychologically
[5] Bored at home
[6] Other

([1]–[5] → Go to D619)

D618. Please specify other.

____

D619. If that is the case, what sort of work do you want to have?

[1] A small pastime job I can do regardless of income
[2] Part time work that allows me to earn some pocket money
[3] Full time work that allows me to earn my living expenses
[4] Self-employed (business start-up)
[5] Other

([1]–[4] → Go to D621)

D620. Please specify other.

____

D621. All in all, would you say that your retirement has turned out to be very satisfying, moderately satisfying, or not at all satisfying?

[1] Very satisfying
[3] Moderately satisfying
[5] Not satisfying

D622. Comparing before and after retirement, would you say you like after retirement to be better than, about the same as, or worse than before retirement?

[1] Better
[3] About the same
[5] Worse

} → Go to D701

* The Last Job R had D701-723 *

D701. The next questions are about the last job you had. We're interested in your situation near the end of this job. What kind of appointment did you hold at that time? Were you a wage earner, self-employed, or working for family business without a pay? If you were a wage earner, were you a regular worker, a temporary worker, or a casual worker? If you were self-employed, did you hire any employees or not?

IWER:
- Regular worker: Workers with an employment contract of more than one year or cases in which workers may choose to work as long as one likes without a predefined contract period
- Temporary worker: Workers with an employment contract of more than one month and less than one year or cases in which the work is expected to be completed within one year, although there is no predefined period of the employment contract (Yet, cases in which one works in one company for a long time and/or expects to work continuously in the future, but has a contract of less than one year are considered as temporary workers)
- Casual worker: Workers with an employment contract of less than one month, who are hired and paid on a daily basis, or compensated for the work done in various locations without a designated workplace

[1] Regular wage worker
[2] Temporary wage worker
[3] Casual wage worker
[4] Self-employed with employees
[5] Self-employed without employees
[6] Family business worker without pay

D702. In which year and month did you start working for that job?

IWER: Mark the year and month in 6 digits. For example, mark 200601 for January 2006. If the month is not clear, enter 00.

_________________________ (range: 190000 ~ 200612)

D703. In which year and month did you stop working that job?

IWER: Mark the year and month in 6 digits. For example, mark 200601 for January 2006. If the month is not clear, enter 00.

_________________________ (range: 190000 ~ 200612)

D705. What was the name of company or business?

____

D706. Where is your work place?

IWER: Press the Enter key then find the location

D707. What kind of business or industry did you work in, that is, what did they make or do at the place you worked?

____

D708. About how many employees worked for that company or organization at all locations (including those at the head office, branch offices, branches, plants and field)?

____________ people (ragne: 0 ~ 99997) → Go to D710

[98] Don't know

[99] Refuse to answer

D709. Which of the following is the closest approximation?

[1] Fewer than 5 people

[2] 5~9 people

[3] 10~29 people

[4] 30~49 people

[5] 50~99 people
[6] 100~299 people
[7] 300~499 people
[8] 500~999 people
[9] More than 1,000 people

D710. What sort of work did you do?

____

D711. Did you work full-time or part-time?
[1] Full time
[5] Part time

D712. How many hours a week did you usually work for this employer/in this business?

____________ hours per week (range: 1 ~ 997)

D713. What was the monthly income from this job? (Unit: 10,000 Korean Won)
IWER: Mark 0 if there is no net income, and mark 999997 if running a deficit.

____________ MW (range: 0 ~ 999997)

D714. Were you enrolled in the National Public Pension system at that time? If so, were you enrolled through your last employer? Or, were you enrolled in the Occupational Pension Plan for Public Workers, Teachers, and Soldiers? Or, were you enrolled in National Public Pension System through community?
[1] Yes, enrolled in the National Pension System through current employer
[2] Yes, enrolled in the Occupational Pension Plan for public servants, teachers and soldiers)
[3] Yes, enrolled in the National Pension System through community
[4] No, not enrolled in the National Pension System

D715. Why did you stop working at this business / leave that employer?
[1] I did not want to quit, but I had no choice
[5] I wanted to quit → Go to D718

D716. Then, which of the following best describes your situation?

[1] Business closed due to bankruptcy, etc
[2] Contract expired
[3] Income / compensation was too low
[4] The workload was too small
[5] The work was temporary or did not have a good prospect
[6] Business was slow
[7] To start up a new business
[8] Unfavorable working conditions / hours
[9] Family responsibilities, such as child care, household work, care for the sick
[10] Poor health
[11] Old age
[12] There was a better job
[13] Lay off
[14] Recommended resignation
[15] Honorary(Early) Retirement
[16] Retirement (at the mandatory retirement age)

([1]–[16]) → Go to D718

[17] Others

D717. Please specify other.

____

D718. Did you receive or expect to receive retirement benefits (based on the regulation or collective bargaining agreement)?

[1] Yes   [5] No → D722

D719. How much retirement allowance were you supposed to receive? (Unit: 10,000 Korean won)

_______________MW (range: 0 ~ 9999997)

D720. What was the amount that you actually received as retirement allowance? (Unit: 10,000 Korean won)

IWER: Mark 0 if R didn't receive retirement allowance.

_______________MW (range: 0 ~ 9999997)

D721. Did you receive any payments other than the legal retirement benefits from your last job? (For example, early retirement compensation or retirement condolence payment) (Unit: 10,000 Korean Won)

IWER: Mark 0 if R hasn't received any other payments.

_______________MW (range: 0~9999997)

D722. IWER: HOW OFTEN DID R RECEIVE ASSISTANCE WITH ANSWERS IN SECTION D – EMPLOYMENT?

[1] Never
[2] A few times
[3] Most or all the time
} → Go to Section E.
[4] The Section was done by a proxy reporter.

D723. IWER: WHAT IS THE PROXY'S RELATIONSHIP TO R? IF UNKNOWN, PLEASE ASK THE PROXY. What is your relationship to R?

[1] Spouse
[2] Mother
[3] Father
[4] Mother-in-law
[5] Father-in-law
[6] Sibling
[7] Brother-in-law, sister-in-law
[8] Child
[9] Spouse of child
[10] Grandchild
[11] Other relative
[12] Helper or other non-relative
→ Go to Section E.

E. INCOME

E. INCOME SURVEY STRUCTURE

E001-E032 : WAGE

- E001-E009 : WAGE
- E010-E018 : INCOME FROM SELF-EMPLOYED WORK(INCOME FROM YOUR OWN BUSINESS
- E019-E025 : INCOME AGRICULTURAL AND FISHING BUSINESS
- E026-E032 : INCOME FROM SIDE JOB

E033-E069 : BENEFITS FROM NATIONAL PENSION SYSTEM

- E033-E043 : BENEFITS FROM NATIONAL PENSION SYSTEM
- E044-E054 : BENEFITS FROM OCCUPATIONAL PENSION SYSTEM(PUBLIC OR PRIVATE SCHOOL TEACHER'S PENSION, GOVERNMENT EMPLOYEE'S PENSION, MILITARY PENSION)
- E055-E069 : INCOME FROM PRIVATE PENSION

E070-E118 : SOCIAL SECURITY BENEFIT

- E070 : THE KIND OF SOCIAL SECURITY BENEFIT
- E071-E080 : UNEMPLOYED BENEFIT
- E081-E090 : WORKER'S COMPENSATION FROM AND KIND OF COMPENSATION(LUMP-SUM PAYMENT OR MONTHLY BENEFITS)
- E091-E099 : NATIONAL BASIC LIVING SECURITY SCHEME(GENERAL BENEFITS AND WORK-RELATED BENEFIT)
- E100-E110 : VETERAN'S BENEFIT
- E111-E118 : OTHER SOCIAL WELFARE BENEFITS

E119-E125 : OTHER INCOME FROM ALIMONY, CHILD SUPPORT, ODD JOBS AND SO ON

E126-E131 : TOTAL AMOUNT OF HOUSEHOLD MEMBERS INCOME IN LAST YEAR

E132 : CHECK POINT OF INTERVIEWER

* WAGE *

E001. Did you do any work for pay in LAST CALEDAR YEAR(2005)?

[1] Yes [5] No → Go to E010

E002. Which months did you get paid last year? Please choose all that apply.

[1] January 2005
[2] February 2005
[3] March 2005
[4] April 2005
[5] May 2005
[6] June 2005
[7] July 2005
[8] August 2005
[9] September 2005
[10] October 2005
[11] November 2005
[12] December 2005
[13] All year round

E003. How much per month did it amount to? (Unit: 10,000 Korean won)

______________MW (range: 1 ~ 99997)

Don't know
Refuse to answer } → Go to E005~E009 unfolding

E004. So you received a total of (amount calculated from E002 and E003) in last calendar year(2005), is this correct?

IWER: Calculate the total amount of wage that R received for the last calendar year using the provided monthly average

[1] Yes → Go to E010 [5] No → Go to E005~E009 unfolding

Don't know
Refuse to answer } → Go to E005~E009 unfolding

E005 ~ E009. UNFOLDING BRACKET QUESTIONS

E005. Did it amount to a total of less than, about equal to or more than 600 MW (10,000 Korean won)?

[1] Less than 600 MW
[3] About 600 MW
[5] More than 600 MW

E006. Did it amount to a total of less than, about equal to, or more than 1,200 MW (10,000 Korean won)?

[1] Less than 1,200 MW
[3] About 1,200 MW
[5] More than 1,200 MW

E007. Did it amount to a total of less than, about equal to or more than 2,400 MW (10,000 Korean won)?

[1] Less than 2,400 MW
[3] About 2,400 MW
[5] More than 2,400 MW

E008. Did it amount to a total of less than, about equal to or more than 6,000 MW (10,000 Korean won)?

[1] Less than 6,000 MW
[3] About 6,000 MW
[5] More than 6,000 MW

E009. Did it amount to a total of less than, about equal to, or more than 12,000 MW (10,000 Korean won)?

[1] Less than 12,000 MW
[3] About 12,000 MW
[5] More than 12,000 MW

E010. Did you earn any income from your own business in 2005? Income earned through agricultural and fisheries business is excluded.

[1] Yes [5] No → Go to E019

E011. Which months did you earn income from your own business? Please choose all that apply.

[1] January 2005
[2] February 2005
[3] March 2005
[4] April 2005
[5] May 2005
[6] June 2005
[7] July 2005
[8] August 2005
[9] September 2005
[10] October 2005
[11] November 2005
[12] December 2005
[13] All year round

E012. How much per month did it amount to? (Unit: 10,000 Korean won)

______________MW (range: 1 ~ 99997)

[98] Don't know
[99] Refuse to answer } → Go to E014~E018 unfolding

E013. So you earned a total of (amount calculated from E011 and E012) in last calendar year(2005), is this correct?

[1] Yes → Go to E019 [5] No → Go to E014 ~ E018 unfolding

Don't know
Refuse to answer } → Go to E014~E018 unfolding

E014 ~ E018. UNFOLDING BRACKET QUESTIONS

E014. Did it amount to a total of less than, about equal to or more than 600 MW (10,000 Korean won)?

[1] Less than 600 MW
[3] About 600 MW
[5] More than 600 MW

E015. Did it amount to a total of less than, about equal to or more than 1,200 MW (10,000 Korean won)?

[1] Less than 1,200 MW
[3] About 1,200 MW
[5] More than 1,200 MW

E016. Did it amount to a total of less than, about equal to or more than 2,400 MW (10,000 Korean won)?

[1] Less than 2,400 MW
[3] About 2,400 MW
[5] More than 2,400 MW

E017. Did it amount to a total of less than, about equal to or more than 6,000 MW (10,000 Korean won)?

[1] Less than 6,000 MW
[3] About 6,000 MW
[5] More than 6,000 MW

E018. Did it amount to a total of less than, about equal to, or more than 12,000 MW (10,000 Korean won)?

[1] Less than 12,000 MW
[3] About 12,000 MW
[5] More than 12,000 MW

E019. Did you earn any income through agricultural and fisheries business in 2005?

[1] Yes [5] No → Go to E026

E020. How much did you earn from agricultural and fisheries business in last calendar year (2005)? (Unit: 10,000 Korean won)

IWER: After taxes and other deductions and excluding expenses. Wage earners in the agricultural and fisheries industry excluded.

______________MW (range: 1 ~ 99997) → Go to E026

Don't know
Refuse to answer } → Go to E021~E025 unfolding

E021 ~ E025. UNFOLDING BRACKET QUESTIONS

E021. Did it amount to a total of less than, about equal to or more than 600 MW (10,000 Korean won)?

IWER: Go back to E020 and record if R answers the amount during the interview. All figures are in MW, which is 10,000 Korean won.

[1] Less than 600 MW
[3] About 600 MW
[5] More than 600 MW

E022. Did it amount to a total of less than, about equal to, or more than 1,200 MW (10,000 Korean won)?

IWER: Go back to E020 and record if R answers the amount during the interview. All figures are in MW, which is 10,000 Korean won.

[1] Less than 1,200 MW
[3] About 1,200 MW
[5] More than 1,200 MW

E023. Did it amount to a total of less than, about equal to or more than 2,400 MW (10,000 Korean won)?

IWER: Go back to E020 and record if R answers the amount during the interview. All figures are in MW, which is 10,000 Korean won.

[1] Less than 2,400 MW
[3] About 2,400 MW
[5] More than 2,400 MW

E024. Did it amount to a total of less than, about equal to or more than 6,000 MW (10,000 Korean won)?

IWER: Go back to E020 and record if R answers the amount during the interview. All figures are in MW, which is 10,000 Korean won.

[1] Less than 6,000 MW
[3] About 6,000 MW
[5] More than 6,000 MW

E025. Did it amount to a total of less than, about equal to or more than 12,000 MW (10,000 Korean won)?

IWER: Go back to E020 and record if R answers the amount during the interview. All figures are in MW, which is 10,000 Korean won.

[1] Less than 12,000 MW
[3] About 12,000 MW
[5] More than 12,000 MW

E026. Did you earn any income from a side job in LAST CALENDAR YEAR(2005)?

[1] Yes
[5] No → Go to E033
Don't know
Refuse to answer } → Go to E028~E032 unfolding

E027. How much did you earn from this job in Last Calendar Year(2005)? (Unit: 10,000 Korean Won)

______________MW (range: 1 ~ 99997) → Go to E033
[98] Don't know
[99] Refuse to answer } → Go to E028~E032 unfolding

E028 ~ E032. UNFOLDING BRACKET QUESTIONS

E028. Did it amount to a total of less than, about equal to or more than 300 MW (10,000 Korean won)?

IWER: Go back to E027 and record if R answers the amount during the interview. All figures are in MW, which is 10,000 Korean won.

[1] Less than 300 MW
[3] About 300 MW
[5] More than 300 MW

E029. Did it amount to a total of less than, about equal to or more than 600 MW (10,000 Korean won)?

IWER: Go back to E027 and record if R answers the amount during the interview. All figures are in MW, which is 10,000 Korean won.

[1] Less than 600 MW
[3] About 600 MW
[5] More than 600 MW

E030. Did it amount to a total of less than, about equal to or more than 1,200 MW (10,000 Korean won)?

IWER: Go back to E027 and record if R answers the amount during the interview. All figures are in MW, which is 10,000 Korean won.

[1] Less than 1,200 MW
[3] About 1,200 MW
[5] More than 1,200 MW

E031. Did it amount to a total of less than, about equal to or more than 2,400 MW (10,000 Korean won)?

IWER: Go back to E027 and record if R answers the amount during the interview. All figures are in MW, which is 10,000 Korean won.

[1] Less than 2,400 MW
[3] About 2,400 MW
[5] More than 2,400 MW

E032. Did it amount to a total of less than, about equal to or more than 6,000 MW (10,000 Korean won)?

IWER: Go back to E027 and record if R answers the amount during the interview. All figures are in MW, which is 10,000 Korean won.

[1] Less than 6,000 MW
[3] About 6,000 MW
[5] More than 6,000 MW

E033. Did you receive any benefits from the National Pension System in Last Calendar Year(2005)? National Pension benefits include Old Age Annuities, Annuities for Disabled Persons, Survival Benefits, and Death Benefits.

[1] Only received monthly income
[2] Only received lump sum payment → Go to E036
[3] Received both
[4] No → Go to E044

E034. In which months did you receive your monthly pension last year(2005)?

[1] January 2005
[2] February 2005
[3] March 2005
[4] April 2005
[5] May 2005
[6] June 2005
[7] July 2005
[8] August 2005
[9] September 2005
[10] October 2005
[11] November 2005
[12] December 2005
[13] All year round

E035. How much per month did it amount to? (Unit: 10,000 Korean won)

______________MW (range: 1 ~ 99997)
[98] Don't know
[99] Refuse to answer } → Go to E038~E042 unfolding

E036. How much pension did you receive last year(2005) in lump sum payment? (Unit: 10,000 Korean won)

______________MW (range: 1 ~ 99997)
[98] Don't know
[99] Refuse to answer } → Go to E038~E042 unfolding

E037. So you received a total of __________ (calculated using E034, E035, and E036) in LAST CALENDAR YEAR(2005), is this correct?

[1] Yes → Go to E043 [1] No → Go to E038~E042 unfolding
[98] Don't know
[99] Refuse to answer } → Go to E038~E042 unfolding

E038 ~ E042. UNFOLDING BRACKET QUESTIONS

E038. Did it amount to a total of less than, about equal to or more than 120 MW (10,000 Korean won)?

IWER: All figures are in MW, which is 10,000 Korean won.

[1] Less than 120 MW
[3] About 120 MW
[5] More than 120 MW

E039. Did it amount to a total of less than, about equal to or more than 240 MW (10,000 Korean won)?

IWER: All figures are in MW, which is 10,000 Korean won.]

[1] Less than 240 MW
[3] About 240 MW
[5] More than 240 MW

E040. Did it amount to a total of less than, about equal to or more than 480 MW (10,000 Korean won)?

IWER: All figures are in MW, which is 10,000 Korean won.

[1] Less than 480 MW
[3] About 480 MW
[5] More than 480 MW

E041. Did it amount to a total of less than, about equal to or more than 600 MW (10,000 Korean won)?

IWER: All figures are in MW, which is 10,000 Korean won.

[1] Less than 600 MW
[3] About 600 MW
[5] More than 600 MW

E042. Did it amount to a total of less than, about equal to, or more than 1,200 MW (10,000 Korean won)?

IWER: All figures are in MW, which is 10,000 Korean won.

[1] Less than 1,200 MW
[3] About 1,200 MW
[5] More than 1,200 MW

E043. When did you start receiving the National Pension benefits?

IWER: Mark the year and month in 6 digits. For example, mark 200601 for January 2006. If the month is not clear, enter 00.

__________________________ (range: 190000 ~ 200612)

E044. Did you receive any income from Occupational Pension in Last Calendar Year (in 2005)? Occupational Pension includes government employees' pension, military personnel pension, and private school teachers' pension. Did you receive your income every month or in lump sum?

[1] Only received monthly income
[2] Only received lump sum payment → Go to E047
[3] Received both
[4] No → Go to E055

E045. In which months did you receive pension benefits?

[1] January 2005
[2] February 2005
[3] March 2005
[4] April 2005
[5] May 2005
[6] June 2005
[7] July 2005
[8] August 2005
[9] September 2005
[10] October 2005
[11] November 2005
[12] December 2005
[13] All year round

E046. How much per month did it amount to? (Unit: 10,000 Korean won)

______________MW (range: 1 ~ 99997) → Go to E047
[98] Don't know
[99] Refuse to answer } → Go to E049~E053 unfolding

E047. How much pension for special occupation did you receive in lump sum Last Year (2005)? (Unit: 10,000 Korean won)

______________MW (range: 1 ~ 99997)
[98] Don't know
[99] Refuse to answer } → Go to E049~E053 unfolding

E048. So you received a total of (amount calculated from E045, E046, and E047) in LAST CALENDAR YEAR(2005), is this correct?

[1] Yes → Go to E054 [5] No → Go to E049~E053 unfolding
Don't know
Refuse to answer } → Go to E049~E053 unfolding

E049 ~ E053. UNFOLDING BRACKET QUESTIONS

E049. Did it amount to a total of less than, about equal to or more than 300 MW (10,000 Korean won)?

[1] Less than 300 MW
[3] About 300 MW
[5] More than 300 MW

E050. Did it amount to a total of less than, about equal to, or more than 600 MW (10,000 Korean won)?

[1] Less than 600 MW
[3] About 600 MW
[5] More than 600 MW

E051. Did it amount to a total of less than, about equal to or more than 1,200 MW (10,000 Korean won)?

[1] Less than 1,200 MW
[3] About 1,200 MW
[5] More than 1,200 MW

E052. Did it amount to a total of less than, about equal to or more than 2,400 MW (10,000 Korean won)?

[1] Less than 2,400 MW
[3] About 2,400 MW
[5] More than 2,400 MW

E053. Did it amount to a total of less than, about equal to or more than 6,000 MW (10,000 Korean won)?

[1] Less than 6,000 MW
[3] About 6,000 MW
[5] More than 6,000 MW

E054. When did you start receiving the occupational pension benefits?

IWER: Mark the year and month in 6 digits. For example, mark 200601 for January 2006. If the month is not clear, enter 00.

__________________________ (range: 190000 ~ 200612)

E055. Aside from income so far mentioned (NATIONAL PENSION, OCCUPATIONAL PENSION), did you receive any income from private pension in last calendar year?

[1] Only received monthly income
[2] Only received lump sum payment → Go to E058
[3] Received both
[4] No → Go to E070

E056. In which months did you receive pension benefits?

[1] January 2005
[2] February 2005
[3] March 2005
[4] April 2005
[5] May 2005
[6] June 2005
[7] July 2005
[8] August 2005
[9] September 200
[10] October 2005
[11] November 2005
[12] December 2005
[13] All year round

E057. How much per month did it amount to? (Unit: 10,000 Korean won)

______________MW (range: 1~99997) → Go to E058

Don't know
Refuse to answer } → Go to E060~E064 unfolding

E058. How much private pension did you receive in lump sum in LAST CALENDAR YEAR (2005)? (Unit: 10,000 Korean won)

______________MW (range: 1 ~ 99997)]

Don't know
Refuse to answer } → Go to E060~E064 unfolding

E059. So you received a total of (amount calculated from E056, E057 and E058) in LAST CALENDAR YEAR(2005), is this correct?

[1] Yes → Go to E065 [5] No → Go to E060~E064 unfolding

Don't know
Refuse to answer } → Go to E060~E064 unfolding

E060 ~ E064. UNFOLDING BRACKET QUESTIONS

E060. Did it amount to a total of less than, about equal to or more than 120 MW (10,000 Korean won)?

[1] Less than 120 MW
[3] About 120 MW
[5] More than 120 MW

E061. Did it amount to a total of less than, about equal to, or more than 240 MW (10,000 Korean won)?

[1] Less than 240 MW
[3] About 240 MW
[5] More than 240 MW

E062. Did it amount to a total of less than, about equal to or more than 360 MW (10,000 Korean won)?

[1] Less than 360 MW
[3] About 360 MW
[5] More than 360 MW

E063. Did it amount to a total of less than, about equal to or more than 600 MW (10,000 Korean won)?

[1] Less than 600 MW
[3] About 600 MW
[5] More than 600 MW

E064. Did it amount to a total of less than, about equal to or more than 1,200 MW (10,000 Korean won)?

[1] Less than 1,200 MW
[3] About 1,200 MW
[5] More than 1,200 MW

E065. In what year and month did you start to receive that annuity payment?

IWER: Mark the year and month in 6 digits. For example, mark 200601 for January 2006. If the month is not clear, enter 00.

_________________________ (range: 190000 ~ 200612)

E066. Will this payment continue as long as you live?

[1] Yes [5] No

E067. For how many years will it continue?

______________years (range: 0 ~ 997)

E068. If you should die, will the income from the annuity stop, stay the same, or reduced?

[1] Stop
[2] Stay the same
[3] Continue at a reduced level
} → Go to E070
[4] Other

E069. Please specify other.

____

E070. Did you receive any of the following social security benefits last year (2005)? Please list all.

[1] Unemployment compensation
[2] Workers' compensation → Go to E081
[3] National Basic Living Security Scheme → Go to E091
[4] Veteran Benefits → Go to E100
[5] Other welfare benefit → Go to E111
[6] No → Go to E119

E071. How did you receive Unemployment Compensation in LAST CALENDAR YEAR (2005)? Did you receive monthly benefits, lump-sum payment, or both? Unemployment compensation includes job-seeking allowance, extended benefits, and early reemployment benefit, as provided from Employment Insurance.

[1] Only received monthly income
[2] Only received lump sum payment → Go to E074
[3] Received both

E072. In which months did you receive unemployment benefits?

[1] January 2005
[2] February 2005
[3] March 2005
[4] April 2005
[5] May 2005
[6] June 2005

[7] July 2005
[8] August 2005
[9] September 2005
[10] October 2005
[11] November 2005
[12] December 2005
[13] All year round

E073. How much per month did it amount to? (10,000－Korean won)

______________MW (range: 1 ~ 99997)
[98] Don't know
[99] Refuse to answer } → Go to E076~E080 unfolding

E074. How much unemployment compensation did you receive in lump sum in 2005? (Unit: 10,000 Korean won)

______________MW
[98] Don't know
[99] Refuse to answer } → Go to E076~E080 unfolding

E075. So you received a total of (amount calculated from E072, E073 and E074) in LAST CALENDAR YEAR(2005), is this correct?

[1] Yes → Go to E081
[5] No
[98] Don't know
[99] Refuse to answer } → Go to E076~E080 unfolding

E076 ~ E080. UNFOLDING BRACKET QUESTIONS

E076. Did it amount to a total of less than, about equal to or more than 100 MW (10,000 Korean won)?

[1] Less than 100 MW
[3] About 100 MW
[5] More than 100 MW

E077. Did it amount to a total of less than, about equal to or more than 200 MW (10,000 Korean won)?

[1] Less than 200 MW
[3] About 200 MW
[5] More than 200 MW

E078. Did it amount to a total of less than, about equal to or more than 400 MW (10,000 Korean won)?

[1] Less than 400 MW
[3] About 400 MW
[5] More than 400 MW

E079. Did it amount to a total of less than, about equal to or more than 600 MW (10,000 Korean won)?

[1] Less than 600 MW
[3] About 600 MW
[5] More than 600 MW

E080. Did it amount to a total of less than, about equal to or more than 800 MW (10,000 Korean won)?

[1] Less than 800 MW
[3] About 800 MW
[5] More than 800 MW

E081. How did you receive income from Workers' Compensation in LAST CALENDAR YEAR (2005)? Did you receive monthly benefits, lump sum payment, or both? Workers' compensation from Industrial Accident Compensation Insurance includes wage-replacement benefits, disability benefits, and survivors' benefits.

[1] Only received monthly income
[2] Only received lump sum payment → Go to E084
[3] Received both

E082. In which months did you receive workers' compensation?

[1] January 2005
[2] February 2005
[3] March 2005
[4] April 2005
[5] May 2005
[6] June 2005
[7] July 2005
[8] August 2005
[9] September 2005
[10] October 2005
[11] November 2005
[12] December 2005
[13] All year round

E083. How much per month did it amount to? (Unit: 10,000 Korean won)

_______________MW (range: 1 ~ 99997)

[98] Don't know
[99] Refuse to answer } → Go to E86~E090 unfolding

E084. How much workers' compensation did you receive in lump sum in LAST CALENDAR YEAR(2005)? (Unit: 10,000 Korean won)

_______________MW (range: 1 ~ 99997)

[98] Don't know
[99] Refuse to answer } → Go to E86~E090 unfolding

E085. So you received a total of (amount calculated from E082, E083, and E084) in LAST CALENDAR YEAR(2005), is this correct?

[1] Yes → Go to E091
[5] No
[98] Don't know
[99] Refuse to answer } → Go to E86~E090 unfolding

E086 ~ E090. UNFOLDING BRACKET QUESTIONS

E086. Did it amount to a total of less than, about equal to or more than 300 MW (10,000 Korean won)?

[1] Less than 300 MW
[3] About 300 MW
[5] More than 300 MW

E087. Did it amount to a total of less than, about equal to, or more than 600 MW (10,000 Korean won)?

[1] Less than 600 MW
[3] About 600 MW
[5] More than 600 MW

E088. Did it amount to a total of less than, about equal to or more than 1,200 MW (10,000 Korean won)?

[1] Less than 1,200 MW
[3] About 1,200 MW
[5] More than 1,200 MW

E089. Did it amount to a total of less than, about equal to or more than 2,400 MW (10,000 Korean won)?

[1] Less than 2,400 MW
[3] About 2,400 MW
[5] More than 2,400 MW

E090. Did it amount to a total of less than, about equal to or more than 6,000 MW (10,000 Korean won)?

[1] Less than 6,000 MW
[3] About 6,000 MW
[5] More than 6,000 MW

E091. You said that you received benefits from the National Basic Living Security Scheme (i.e., Supplemental Social Security Benefits). In which months did you receive the benefits?

[1] January 2005
[2] February 2005
[3] March 2005
[4] April 2005
[5] May 2005
[6] June 2005
[7] July 2005
[8] August 2005
[9] September 2005
[10] October 2005
[11] November 2005
[12] December 2005
[13] All year round

E092. How much per month did it amount to? (Unit: 10,000 Korean won)

______________MW (range: 1 ~ 99997)

[98] Don't know
[99] Refuse to answer } → Go to E094~E098 unfolding

E093. So you received a total of (amount calculated from E091 and E092) in LAST CALENDAR YEAR(2005), is this correct?

[1] Yes → E099
[5] No
[98] Don't know
[99] Refuse to answer } → Go to E094~E098 unfolding

E094 ~ E098. UNFOLDING BRACKET QUESTIONS

E094. Did it amount to a total of less than, about equal to or more than 120 MW (10,000 Korean won)?

[1] Less than 120 MW
[3] About 120 MW
[1] More than 120 MW

E095. Did it amount to a total of less than, about equal to or more than 240 MW (10,000 Korean won)?

[1] Less than 240 MW
[3] About 240 MW
[5] More than 240 MW

E096. Did it amount to a total of less than, about equal to or more than 480 MW (10,000 Korean won)?

[1] Less than 480 MW
[3] About 480 MW
[5] More than 480 MW

E097. Did it amount to a total of less than, about equal to or more than 600 MW (10,000 Korean won)?

[1] Less than 600 MW
[3] About 600 MW
[5] More than 600 MW

E098. Did it amount to a total of less than, about equal to, or more than 1,200 MW (10,000 Korean won)?

[1] Less than 1,200 MW
[3] About 1,200 MW
[5] More than 1,200 MW

E099. When did you start receiving benefits from the National Basic Living Security Scheme?

IWER: Mark the year and month in 6 digits. For example, mark 200601 for January 2006. If the month is not clear, enter 00.

_________________________ (range: 190000 ~ 200612)

E100. How did you receive veteran's benefits in LAST CALENDAR YEAR(2005)? Did you receive monthly benefits, lump-sum payments, or both? Veteran's benefits include benefits to patriots and their surviving families, pension and nursing allowances for wounded policemen and soldiers.

[1] Only received monthly income
[2] Only received lump sum payment → Go to E103
[3] Received both

E101. In which months did you receive veteran's benefits?

[1] January 2005
[2] February 2005
[3] March 2005
[4] April 2005
[5] May 2005
[6] June 2005
[7] July 2005
[8] August 2005
[9] September 2005
[10] October 2005
[11] November 2005
[12] December 2005
[13] All year round

E102. How much per month did it amount to? (Unit: 10,000 Korean won)

______________MW (range: 1~99997)
[98] Don't know
[99] Refuse to answer } → Go to E105~E109 unfolding

E103. How much veteran's benefits did you receive in lump sum in 2005? (Unit: 1,000-Korean won)

______________MW (range: 1 ~ 99997)
[98] Don't know
[99] Refuse to answer } → Go to E105~E109 unfolding

E104. So you received a total of (amount calculated from E101, E102, and E103) in LAST CALENDAR YEAR(2005), is this correct?

[1] Yes → Go to E110
[5] No
[98] Don't know
[99] Refuse to answer } → Go to E105~E109 unfolding

E105 ~ E109. UNFOLDING BRACKET QUESTIONS

E105. Did it amount to a total of less than, about equal to or more than 120 MW (10,000 Korean won)?
[1] Less than 120 MW
[3] About 120 MW
[5] More than 120 MW

E106. Did it amount to a total of less than, about equal to or more than 240 MW (10,000 Korean won)?
[1] Less than 240 MW
[3] About 240 MW
[5] More than 240 MW

E107. Did it amount to a total of less than, about equal to or more than 480 MW (10,000 Korean won)?
[1] Less than 480 MW
[3] About 480 MW
[5] More than 480 MW

E108. Did it amount to a total of less than, about equal to or more than 600 MW (10,000 Korean won)?
[1] Less than 600 MW
[3] About 600 MW
[5] More than 600 MW

E109. Did it amount to a total of less than, about equal to or more than 1,200 MW (10,000 Korean won)?
[1] Less than 1,200 MW
[3] About 1,200 MW
[5] More than 1,200 MW

E110. When did you start receiving veteran's benefit?

IWER: Mark the year and month in 6 digits. For example, mark 200601 for January 2006. If the month is not clear, enter 00.

_________________________ (range: 190000~200612)

E111. In which months did you receive other welfare benefits?

[1] January 2005
[2] February 2005
[3] March 2005
[4] April 2005
[5] May 2005
[6] June 2005
[7] July 2005
[8] August 2005
[9] September 2005
[10] October 2005
[11] November 2005
[12] December 2005
[13] All year round

E112. How much per month did it amount? (Unit: 10,000 Korean won)

_______________MW (range: 1 ~ 99997)

[98] Don't know
[99] Refuse to answer
} → Go to E114~E118 unfolding

E113. So you received a total of (amount calculated from E111 and E112) in LAST CALENDAR YEAR(2005), is this correct?

[1] Yes → E119
[5] No
[98] Don't know
[99] Refuse to answer
} → E114~E118 unfolding

E114 ~ E118. UNFOLDING BRACKET QUESTIONS

E114. Did it amount to a total of less than, about equal to or more than 50 MW (10,000 Korean won)?

[1] Less than 50 MW
[3] About 50 MW
[5] More than 50 MW

E115. Did it amount to a total of less than, about equal to or more than 100 MW (10,000 Korean won)?

[1] Less than 100 MW
[3] About 100 MW
[5] More than 100 MW

E116. Did it amount to a total of less than, about equal to or more than 200 MW (10,000 Korean won)?
[1] Less than 200 MW
[3] About 200 MW
[5] More than 200 MW

E117. Did it amount to a total of less than, about equal to or more than 300 MW (10,000 Korean won)?
[1] Less than 300 MW
[3] About 300 MW
[5] More than 300MW

E118. Did it amount to a total of less than, about equal to or more than 600 MW (10,000 Korean won)?
[1] Less than 600 MW
[3] About 600 MW
[5] More than 600 MW

E119. Other than income you have already told me about, did you receive any other income from alimony, child support, consulting fees, odd jobs, and so forth in LAST CALENDAR YEAR(2005)?

IWER: PLEASE DO NOT INCLUDE INCOME FROM REAL ESTATE, FARM, BUSINESS, RENT FROM HOME OR SECOND HOME AS THE RELATED QUESTIONS WILL BE ASKED IN ASSETS SECTION.

[1] Yes [5] No → Go to E126

E120. How much in total did you receive these other sources in LAST CALENDAR YEAR (2005), after taxes and other deductions? (Unit: 10,000 Korean won)

_______________MW (range: 1 ~ 99997) → Go to E126
[98] Don't know
[99] Refuse to answer } → Go to E121~E125 unfolding

E121 ~ E125. UNFOLDING BRACKET QUESTIONS

E121. Did it amount to a total of less than, about equal to or more than 120 MW (10,000 Korean won)?
[1] Less than 120 MW
[3] About 120 MW
[5] More than 120 MW

E122. Did it amount to a total of less than, about equal to or more than 240 MW (10,000 Korean won)?

[1] Less than 240 MW
[3] About 240 MW
[5] More than 240 MW

E123. Did it amount to a total of less than, about equal to or more than 480 MW (10,000 Korean won)?

[1] Less than 480 MW
[3] About 480 MW
[5] More than 480 MW

E124. Did it amount to a total of less than, about equal to or more than 600 MW (10,000 Korean won)?

[1] Less than 600 MW
[3] About 600 MW
[5] More than 600 MW

E125. Did it amount to a total of less than, about equal to or more than 1,200 MW (10,000 Korean won)?

[1] Less than 1,200 MW
[3] About 1,200 MW
[5] More than 1,200 MW

E126. Tell me about all the income that you and your household members received in LAST CALENDAR YEAR(2005). Roughly, what was the total household income during the LAST YEAR(2005) after taxes and other deductions? (Unit: 10,000 Korean won)

______________MW (range: 0 ~ 99997) → Go to E132

[98] Don't know
[99] Refuse to answer } → Go to E127~E131 unfolding

E127 ~ E131. UNFOLDING BRACKET QUESTIONS

E127. Did it amount to a total of less than, about equal to or more than 1,000 MW (10,000 Korean won)?

[1] Less than 1,000 MW
[3] About 1,000 MW
[5] More than 1,000 MW

E128. Did it amount to a total of less than, about equal to, or more than 2,000 MW (10,000 Korean won)?

[1] Less than 2,000 MW
[3] About 2,000 MW
[5] More than 2,000 MW

E129. Did it amount to a total of less than, about equal to or more than 3,000 MW (10,000 Korean won)?

[1] Less than 3,000 MW
[3] About 3,000 MW
[5] More than 3,000 MW

E130. Did it amount to a total of less than, about equal to or more than 5,000 MW (10,000 Korean won)?

[1] Less than 5,000 MW
[3] About 5,000 MW
[5] More than 5,000 MW

E131. Did it amount to a total of less than, about equal to or more than 10,000 MW (10,000 Korean won)?

[1] Less than 10,000 MW
[3] About 10,000 MW
[5] More than 10,000 MW

E132. IWER: HOW OFTEN DID R RECEIVE ASSISTANCE WITH ANSWERS IN SECTION E - INCOME?

[1] Never
[2] A few times
[3] Most or all of the times
} → Go to Section F.
[4] he Section was done by a proxy reporter

E133. IWER: WHAT IS THE PROXY'S RELATIONSHIP TO R? IF UNKNOWN, PLEASE ASK THE PROXY. What is your relationship to R?

[1] Spouse
[2] Mother
[3] Father
[4] Mother-in-law
[5] Father-in-law
[6] Sibling
[7] Brother-in-law, sister-in-law

[8] Child
[9] Spouse of child
[10] Grandchild
[11] Other relative
[12] Helper or other non-relative
→ Go to Section F.

F. ASSETS AND DEBTS

F. ASSETS AND DEBTS SURVEY STRUCTURE

F001-F048 : CURRENT HOUSING OWNERSHIP

- F001-F002 : HOUSE OWNERSHIP TYPE
- F003-F030 : OWN HOUSE
- F031-F036 : JEON-SE(LEASE ON A DEPOSIT BASIS WITHOUT MONTHLY RENT / MONTHLY RENT WITH A DEPOSIT)
- F037-F048 : WOL-SE(MONTHLY RENT OR MONTHLY RENT WITHOUT A DEPOSIT)

F049-F083 : REAL ESTATE EXCEPT CURRENT HOME

- F049-F076 : REAL ESTATE - OWNERSHIP, LEASING/RENTING
- F077-F083 : BUSINESS/FARM

F084-F171 : FINANCIAL ASSETS

F084-F111 : FINANCIAL ASSETS - CASH AND BANK SAVINGS, STOCK / TRUSTS / MUTUAL FUNDS, BONDS

- F112-F137 : INSURANCE - TERM LIFE INSURANCE, WHOLE LIFE INSURANCE, ANNUITY INSURANCE
- F138-F144 : MONEY LENDING OTHER FROM PERSONAL LOAN
- F145-F157 : GYE(PRIVATE SAVING CLUB)
- F158-F171 : OTHER FINANCIAL ASSETS

F172-F185 : OTHER ASSETS - TRANSPORTATION, OTHER NON-FINANCIAL ASSETS

F186-F196 : INHERITANCE(RECEIVED MONEY OR PROPERTY IN THE FORM OF INHERITANCE, A TRUST FUND, OR AN INSURANCE SETTLEMENT)

F197-F225 : DEBTS

F226-F232 : TOTAL ASSETS OF HOUSE MEMBERS

F233 : CHECKPOINT OF INTERVIEWER

F001. Do you (and your spouse / partner) own your home, rent it, or what?

[1] Own → Go to F003
[2] Lease on a deposit basis without monthly rent (i.e., JEON-SE) → Go to F031
[3] Monthly rent with a deposit (i.e., JEON-WOL-SE) → Go to F037
[4] Monthly rent without a deposit (i.e., WOL-SE) → Go to F043
[5] Other

F002. Please specify other.

__________________________ → Go to F049

* Housing *

F003. Is the deed to the house (you are currently residing) under your name, your spouse's name, your family member's, or someone else's name?

[1] R's name
[5] Other's name including R's spouse

F004. Are multiple persons' names listed on the house deed? If so, how many names are listed there?

IWER: if multiple person's names are NOT listed, please enter 0

____________ people (range: 0 ~ 20)

F005. What is its present value? I mean, what would it bring if it were sold today? (Unit: 10,000 Korean won)

__________ MW (range: 1 ~ 9999997) → Go to F011

[98] Don't know
[99] Refuse to answer } → Go to F006~F010 unfolding

F006 ~ F010. UNFOLDING BRACKET QUESTIONS

F006. Would it amount to less than, about equal to or more than 1,000 MW (10,000 Korean won)?

[1] Less than 1,000 MW
[3] About 1,000 MW
[5] More than 1,000 MW

F007. Would it amount to less than, about equal to, or more than 5,000 MW (10,000 Korean won)?

[1] Less than 5,000 MW
[3] About 5,000 MW
[5] More than 5,000 MW

F008. Would it amount to less than, about equal to or more than 10,000 MW (10,000 Korean won)?

[1] Less than 10,000 MW
[3] About 10,000 MW
[5] More than 10,000 MW

F009. Would it amount to less than, about equal to or more than 50,000 MW (10,000 Korean won)?

[1] Less than 50,000 MW
[3] About 50,000 MW
[5] More than 50,000 MW

F010. Would it amount to a total of less than, about equal to or more than 100,000 MW (10,000 Korean won)?

[1] Less than 100,000 MW
[3] About 100,000 MW
[5] More than 100,000 MW

F011. Did you get a loan to purchase the house you are currently living in? How much do you still owe on the loan? (Unit: 10,000 Korean won)

_____________MW (range: 0 ~ 9999997)

F012. Are you (and your spouse) currently leasing part of your current residence to other people? Please choose all that apply.

[1] Leasing on a deposit basis (i.e., *JEON-SE*)
[2] Leasing on a secured monthly rent basis (i.e., *JEON-WOL-SE*)
[3] Leasing on a monthly rent basis (i.e., *WOL-SE*)
[4] Not leasing → Go to F049

Logic: F012 R's responses [1], [1] [2], [1] [3] or [1] [2] [3] then go to F013

F013. How much was JEON-SE security deposit you received? Please tell me only the amount of security deposit you received by leasing the house you are currently living in. (Unit: 10,000 Korean won)

_____________MW (range: 1 ~ 999997) → Go to F019

[98] Don't know
[99] Refuse to answer } → Go to F014~F018 unfolding

F014 ~ F018. UNFOLDING BRACKET QUESTIONS

F014. Did it amount to less than, about equal to or more than 1,000 MW (10,000 Korean won)?

[1] Less than 1,000 MW
[3] About 1,000 MW
[5] More than 1,000 MW

F015. Did it amount to less than, about equal to or more than 3,000 MW (10,000 Korean won)?

[1] Less than 3,000 MW
[3] About 3,000 MW
[5] More than 3,000 MW

F016. Did it amount to less than, about equal to or more than 5,000 MW (10,000 Korean won)?

[1] Less than 5,000 MW
[3] About 5,000 MW
[5] More than 5,000 MW

F017. Did it amount to less than, about equal to or more than 10,000 MW (10,000 Korean won)?

[1] Less than 10,000 MW
[3] About 10,000 MW
[5] More than 10,000 MW

F018. Did it amount to a total of less than, about equal to or more than 30,000 MW (10,000 Korean won)?

[1] Less than 30,000 MW
[3] About 30,000 MW
[5] More than 30,000 MW

Logic : – F012 R' responses F012 then go to F049
– F012 R's responses F012 [2], [1] [2], [2] [3] or [1] [2] [3] then go to F019, not then go to F025

F019. How much was the safety deposit you received? Please state only the amount of deposit you received from leasing the house you are currently living in. (Unit: 10,000 Korean won)

______________MW (range: 1 ~ 999997) → Go to F025

[98] Don't know
[99] Refuse to answer } → Go to F020~F024 unfolding

F020 ~ F024. UNFOLDING BRACKET QUESTIONS

F020. Did it amount to less than, about equal to or more than 300 MW (10,000 Korean won)?
[1] Less than 300 MW
[3] About 300 MW
[5] More than 300 MW

F021. Did it amount to less than, about equal to or more than 500 MW (10,000 Korean won)?
[1] Less than 500 MW
[3] About 500 MW
[5] More than 500 MW

F022. Did it amount to less than, about equal to or more than 1,000 MW (10,000 Korean won)?
[1] Less than 1,000 MW
[3] About 1,000 MW
[5] More than 1,000 MW

F023. Did it amount to less than, about equal to or more than 5,000 MW (10,000 Korean won)?
[1] Less than 5,000 MW
[3] About 5,000 MW
[5] More than 5,000 MW

F024. Did it amount to a total of less than, about equal to or more than 10,000 MW (10,000 Korean won)?
[1] Less than 10,000 MW
[3] About 10,000 MW
[5] More than 10,000 MW

Logic: - F012 R' responses [2] or [1] [2] then go to F025
- F012 R' responses [3], [1] [3], [2] [3] or [1] [2] [3] then go to F025. Not then go to F049

F025. How much is the monthly rent you received? Please state only the amount of rent you received from leasing the house you are currently living in. (Unit: 10,000 Korean won)

______________MW (range: 1 ~ 999997) → Go to F049

[98] Don't know
[99] Refuse to answer } → Go to F026~F030 unfolding

F026 ~ F030. UNFOLDING BRACKET QUESTIONS

F026. Did it amount to less than, about equal to or more than 10 MW (10,000 Korean won)?
[1] Less than 10 MW
[3] About 10 MW
[5] More than 10 MW

F027. Did it amount to less than, about equal to or more than 30 MW (10,000 Korean won)?
[1] Less than 30 MW
[3] About 30 MW
[5] More than 30 MW

F028. Did it amount to less than, about equal to or more than 50 MW (10,000 Korean won)?
[1] Less than 50 MW
[3] About 50 MW
[5] More than 50 MW

F029. Did it amount to less than, about equal to or more than 100 MW (10,000 Korean won)?
[1] Less than 100 MW
[3] About 100 MW
[5] More than 100 MW

F030. Did it amount to a total of less than, about equal to or more than 300 MW (10,000 Korean won)?
[1] Less than 300 MW
[3] About 300 MW
[5] More than 300 MW

SKIP PATTERN CHECKPOINT : JEON－SE IF R OWNS THE CURRENT RESIDENCE, GO TO F049.
IF R LIVES IN THE CURRENT RESIDENCE ON A DEPOSIT BASIS WITHOUT MONTHLY RENT ('JEON－SE'), GO TO F031.
IF R LIVES IN THE CURRENT RESIDENCE BY PAYING MONTHLY RENT WITH A SECURITY DEPOSIT ('JEON－WOL－SE'), GO TO F037.
IF R LIVES IN THE CURRENT RESIDENCE BY PAYING MONTHLY RENT WITHOUT A SECURITY DEPOSIT ('WOL－SE'), GO TO F043

Logic: F001 R's responses [2] then go to F031

F031. How much JEON-SE security deposit did you make for the current residence? (Unit: 10,000 Korean won)

______________MW (range: 1 ~ 999997) → Go to F049
[98] Don't know
[99] Refuse to answer
} → Go to F032~F036 unfolding

F032 ~ F036. UNFOLDING BRACKET QUESTIONS

F032. Did it amount to less than, about equal to or more than 1,000 MW (10,000 Korean won)?
[1] Less than 1,000 MW
[3] About 1,000 MW
[5] More than 1,000 MW

F033. Did it amount to less than, about equal to or more than 3,000 MW (10,000 Korean won)?
[1] Less than 3,000 MW
[3] About 3,000 MW
[5] More than 3,000 MW

F034. Did it amount to less than, about equal to or more than 5,000 MW (10,000 Korean won)?
[1] Less than 5,000 MW
[3] About 5,000 MW
[5] More than 5,000 MW

F035. Did it amount to less than, about equal to or more than 10,000 MW (10,000 Korean won)?
[1] Less than 10,000 MW
[3] About 10,000 MW
[5] More than 10,000 MW

F036. Did it amount to a total of less than, about equal to or more than 30,000 MW (10,000 Korean won)?

[1] Less than 30,000 MW
[3] About 30,000 MW
[5] More than 30,000 MW

Logic: F001 R's responses [3] then go to F037

F037. How much safety deposit did you pay for your current residence? (Unit: 10,000 Korean won)

______________MW (range: 1 ~ 999997) → Go to F043
[98] Don't know
[99] Refuse to answer } → Go to F038~F042 unfolding

F038 ~ F042. UNFOLDING BRACKET QUESTIONS

F038. Did it amount to less than, about equal to or more than 300 MW (10,000 Korean won)?

[1] Less than 300 MW
[3] About 300 MW
[5] More than 300 MW

F039. Did it amount to less than, about equal to or more than 500 MW (10,000 Korean won)?

[1] Less than 500 MW
[3] About 500 MW
[5] More than 500 MW

F040. Did it amount to less than, about equal to or more than 1,000 MW (10,000 Korean won)?

[1] Less than 1,000 MW
[3] About 1,000 MW
[5] More than 1,000 MW

F041. Did it amount to less than, about equal to or more than 3,000 MW (10,000 Korean won)?

[1] Less than 3,000 MW
[3] About 3,000 MW
[5] More than 3,000 MW

F042. Did it amount to a total of less than, about equal to or more than 6,000 MW (10,000 Korean won)?

[1] Less than 6,000 MW
[3] About 6,000 MW
[5] More than 6,000 MW

F043. How much do you pay for the monthly rent? (Unit: 10,000 Korean won)

______________MW (range: 1 ~ 999997) → Go to F049

[98] Don't know
[99] Refuse to answer } → Go to F044~F048 unfolding

F044 ~ F048. UNFOLDING BRACKET QUESTIONS

F044. Did it amount to less than, about equal to or more than 10 MW (10,000 Korean won)?
- [1] Less than 10 MW
- [3] About 10 MW
- [5] More than 10 MW

F045. Did it amount to less than, about equal to or more than 30 MW (10,000 Korean won)?
- [1] Less than 30 MW
- [3] About 30 MW
- [5] More than 30 MW

F046. Did it amount to less than, about equal to or more than 50 MW (10,000 Korean won)?
- [1] Less than 50 MW
- [3] About 50 MW
- [5] More than 50 MW

F047. Did it amount to less than, about equal to or more than 100 MW (10,000 Korean won)?
- [1] Less than 100 MW
- [3] About 100 MW
- [5] More than 100 MW

F048. Did it amount to a total of less than, about equal to or more than 300 MW (10,000 Korean won)?
- [1] Less than 300 MW
- [3] About 300 MW
- [5] More than 300 MW

F049. Do you have any real estate, such as land, rental real estate, a partnership, or money owed to you on a land contract or mortgage except your current home?

[1] Yes [5] No → Go to F084

F050. If you sold all that and then paid off any debts on it, about how much would you get? (Unit: 10,000 Korean won)

______________MW (range: 1 ~ 9999997) → Go to F056

[98] Don't know
[99] Refuse to answer } → Go to F051~F055 unfolding

F051 ~ F055. UNFOLDING BRACKET QUESTIONS

F051. Would it amount to less than, about equal to or more than 1,000 MW (10,000 Korean won)?
[1] Less than 1,000 MW
[3] About 1,000 MW
[5] More than 1,000 MW

F052. Would it amount to less than, about equal to, or more than 5,000 MW (10,000 Korean won)?
[1] Less than 5,000 MW
[3] About 5,000 MW
[5] More than 5,000 MW

F053. Would it amount to less than, about equal to or more than 10,000 MW (10,000 Korean won)?
[1] Less than 10,000 MW
[3] About 10,000 MW
[5] More than 10,000 MW

F054. Would it amount to less than, about equal to or more than 50,000 MW (10,000 Korean won)?
[1] Less than 50,000 MW
[3] About 50,000 MW
[5] More than 50,000 MW

F055. Would it amount to a total of less than, about equal to or more than 100,000 MW (10,000 Korean won)?
[1] Less than 100,000 MW
[3] About 100,000 MW
[5] More than 100,000 MW

F056. Are you currently leasing a house, building, field or land other than your current residence? Please exclude any leasing arrangement of your current residence.

[1] Yes [5] No → Go to F063

F057. Did you receive any safety deposits by leasing [that property / those properties]? How much in total did you receive as safety deposits? (Unit: 10,000 Korean won)

IWER: Mark 0 if there is no security deposit

______________MW (range: 0 ~ 999997) → Go to F063

[98] Don't know
[99] Refuse to answer } → Go to F058~F062 unfolding

F058 ~ F062. UNFOLDING BRACKET QUESTIONS

F058. Did it amount to less than, about equal to or more than 1,000 MW (10,000 Korean won)?

[1] Less than 1,000 MW
[3] About 1,000 MW
[5] More than 1,000 MW

F059. Did it amount to less than, about equal to or more than 3,000 MW (10,000 Korean won)?

[1] Less than 3,000 MW
[3] About 3,000 MW
[5] More than 3,000 MW

F060. Did it amount to less than, about equal to or more than 5,000 MW (10,000 Korean won)?

[1] Less than 5,000 MW
[3] About 5,000 MW
[5] More than 5,000 MW

F061. Did it amount to less than, about equal to or more than 10,000 MW (10,000 Korean won)?

[1] Less than 10,000 MW
[3] About 10,000 MW
[5] More than 10,000 MW

F062. Did it amount to a total of less than, about equal to or more than 30,000 MW (10,000 Korean won)?

[1] Less than 30,000 MW
[3] About 30,000 MW
[5] More than 30,000 MW

F063. Have you earned any regular income or rent such as monthly rent or land rent from all of your properties including your current residence in LAST CALENDAR YEAR? Please exclude safety deposits received.

[1] Yes [5] No → Go to F070

F064. After taxes and other deductions, about how much in total did you receive as rental income in LAST CALENDAR YEAR(2005)? (Unit: 10,000 Korean won)

IWER: DO NOT INCLUDE RENTAL INCOME FROM A BUSINESS OR FARM R OWNS. DO NOT INCLUDE CAPITAL GAINS DUE TO PRICE INCREASE

______________MW (range: 1 ~ 999997) → Go to F070

[98] Don't know
[99] Refuse to answer
} → Go to F065~F069 unfolding

F065 ~ F069. UNFOLDING BRACKET QUESTIONS

F065. Did it amount to less than, about equal to or more than 1,000 MW (10,000 Korean won)?
[1] Less than 1,000 MW
[3] About 1,000 MW
[5] More than 1,000 MW

F066. Did it amount to less than, about equal to or more than 3,000 MW (10,000 Korean won)?
[1] Less than 3,000 MW
[3] About 3,000 MW
[5] More than 3,000 MW

F067. Did it amount to less than, about equal to or more than 5,000 MW (10,000 Korean won)?
[1] Less than 5,000 MW
[3] About 5,000 MW
[5] More than 5,000 MW

F068. Did it amount to less than, about equal to or more than 10,000 MW (10,000 Korean won)?
[1] Less than 10,000 MW
[3] About 10,000 MW
[5] More than 10,000 MW

F069. Did it amount to a total of less than, about equal to or more than 30,000 MW (10,000 Korean won)?
[1] Less than 30,000 MW
[3] About 30,000 MW
[5] More than 30,000 MW

F070. Are you currently renting a house, building, field or land other than your current residence?
[1] Yes [5] No → Go to F077

F071. How much in total did you pay as security deposit (for both *JEON－SE* and *JEON－WOL－SE*) for these rental arrangements? (Unit: 10,000 Korean won)

______________MW (range: 1 ~ 999997) → Go to F077

[98] Don't know
[99] Refuse to answer } → Go to F072~F076 unfolding

F072 ~ F076. UNFOLDING BRACKET QUESTIONS

F072. Did it amount to less than, about equal to or more than 1,000 MW (10,000 Korean won)?

[1] Less than 1,000 MW
[3] About 1,000 MW
[5] More than 1,000 MW

F073. Did it amount to less than, about equal to or more than 3,000 MW (10,000 Korean won)?

[1] Less than 3,000 MW
[3] About 3,000 MW
[5] More than 3,000 MW

F074. Did it amount to less than, about equal to or more than 5,000 MW (10,000 Korean won)?

[1] Less than 5,000 MW
[3] About 5,000 MW
[5] More than 5,000 MW

F075. Did it amount to less than, about equal to or more than 10,000 MW (10,000 Korean won)?

[1] Less than 10,000 MW
[3] About 10,000 MW
[5] More than 10,000 MW

F076. Did it amount to a total of less than, about equal to or more than 30,000 MW (10,000 Korean won)?

[1] Less than 30,000 MW
[3] About 30,000 MW
[5] More than 30,000 MW

F077. Do you own part or all of a business or farm?

[1] Yes [5] No → Go to F084

F078. If you sold all that and then paid off any debts on it, about how much would you get? (Unit: 10,000 Korean won)

IWER: The real estate value mentioned earlier is excluded.

_____________MW (range: 1 ~ 999997) → Go to F084

[98] Don't know
[99] Refuse to answer } → Go to F079~F083 unfolding

F079 ~ F083. UNFOLDING BRACKET QUESTIONS

F079. Would it amount to less than, about equal to or more than 1,000 MW (10,000 Korean won)?

[1] Less than 1,000 MW
[3] About 1,000 MW
[5] More than 1,000 MW

F080. Would it amount to less than, about equal to or more than 5,000 MW (10,000 Korean won)?

[1] Less than 5,000 MW
[3] About 5,000 MW
[5] More than 5,000 MW

F081. Would it amount to less than, about equal to or more than 10,000 MW (10,000 Korean won)?

[1] Less than 10,000 MW
[3] About 10,000 MW
[5] More than 10,000 MW

F082. Would it amount to a total of less than, about equal to or more than 30,000 MW (10,000 Korean won)?

[1] Less than 30,000 MW
[3] About 30,000 MW
[5] More than 30,000 MW

F083. Would it amount to a total of less than, about equal to or more than 50,000 MW (10,000 Korean Won)?

[1] Less than 50,000 MW
[3] About 50,000 MW
[5] More than 50,000 MW

F084. Next we have some questions about your financial assets. Financial assets include cash over 50 MW (10,000-Korean won), bank savings, stocks / trusts / mutual funds, bonds,

insurance, private money lending, mutual savings club, etc. Do you have over 50MW (10,000 Korean won) cash in hand or in checking accounts that yield low interests while highly convertible to cash?

[1] Yes [5] No → Go to F091

F085. If you added up all cash and checking accounts, about how much would they amount to right now? (Unit: 10,000 Korean won)

______________MW (range: 50 ~ 999997) → Go to F091

[98] Don't know
[99] Refuse to answer } → Go to F086~F090 unfolding

F086 ~ F090. UNFOLDING BRACKET QUESTIONS

F086. Would it amount to less than, about equal to or more than 100 MW (10,000 Korean won)?
[1] Less than 100 MW
[3] About 100 MW
[5] More than 100 MW

F087. Would it amount to less than, about equal to, or more than 500 MW (10,000 Korean won)?
[1] Less than 500 MW
[3] About 500 MW
[5] More than 500 MW

F088. Would it amount to less than, about equal to or more than 1,000 MW (10,000 Korean won)?
[1] Less than 1,000 MW
[3] About 1,000 MW
[5] More than 1,000 MW

F089. Would it amount to less than, about equal to or more than 5,000 MW (10,000 Korean won)?
[1] Less than 5,000 MW
[3] About 5,000 MW
[5] More than 5,000 MW

F090. Would it amount to a total of less than, about equal to or more than 10,000 MW (10,000 Korean won)?
[1] Less than 10,000 MW
[3] About 10,000 MW
[5] More than 10,000 MW

F091. Do you have any money in installment deposits, certificates of deposits, and other savings accounts that yield relatively high interests with less liquidity compared to checking accounts?

[1] Yes [5] No → Go to F098

F092. If you added up all such accounts, about how much would they amount to right now? (Unit: 10,000 Korean won)

______________MW (range: 1 ~ 99999997) → Go to F098

[98] Don't know
[99] Reuse to answer
} → Go to F093~F097 unfolding

F093 ~ F097. UNFOLDING BRACKET QUESTIONS

F093. Would it amount to less than, about equal to or more than 500 MW (10,000 Korean won)?

[1] Less than 500 MW
[3] About 500 MW
[5] More than 500 MW

F094. Would it amount to less than, about equal to, or more than 1,000 MW (10,000 Korean won)?

[1] Less than 1,000 MW
[3] About 1,000 MW
[5] More than 1,000 MW

F095. Would it amount to less than, about equal to or more than 5,000 MW (10,000 Korean won)?

[1] Less than 5,000 MW
[3] About 5,000 MW
[5] More than 5,000 MW

F096. Would it amount to less than, about equal to or more than 10,000 MW (10,000 Korean won)?

[1] Less than 10,000 MW
[3] About 10,000 MW
[5] More than 10,000 MW

F097. Would it amount to a total of less than, about equal to or more than 30,000 MW (10,000 Korean won)?

[1] Less than 30,000 MW
[3] About 30,000 MW
[5] More than 30,000 MW

F098. Do you have any shares of stocks or mutual funds?

[1] Yes [5] No → Go to F105

F099. If you sold all these stocks and mutual funds today, about how much would you have? (Unit: 10,000 Korean won)

_______________MW (range: 1 ~ 999997) → Go to F105

[98] Don't know
[99] Refuse to answer } → Go to F100~F104 unfolding

F100 ~ F104. UNFOLDING BRACKET QUESTIONS

F100. Would it amount to less than, about equal to or more than 500 MW (10,000 Korean won)?

[1] Less than 500 MW
[3] About 500 MW
[5] More than 500 MW

F101. Would it amount to less than, about equal to, or more than 1,000 MW (10,000 Korean won)?

[1] Less than 1,000 MW
[3] About 1,000 MW
[5] More than 1,000 MW

F102. Would it amount to less than, about equal to or more than 5,000 MW (10,000 Korean won)?

[1] Less than 5,000 MW
[3] About 5,000 MW
[5] More than 5,000 MW

F103. Would it amount to less than, about equal to or more than 10,000 MW (10,000 Korean won)?

[1] Less than 10,000 MW
[3] About 10,000 MW
[5] More than 10,000 MW

F104. Would it amount to a total of less than, about equal to or more than 30,000 MW (10,000 Korean won)?

[1] Less than 30,000 MW
[3] About 30,000 MW
[5] More than 30,000 MW

F105. Do you have any corporate, municipal, government, or foreign bonds?

[1] Yes [5] No → Go to F112

F106. If you sold all those bonds, about how much would you have? (Unit: 10,000 Korean won)

______________MW (range: 1 ~ 999997) → Go to F112

[98] Don't know
[99] Refuse to answer } → Go to F107~F111 unfolding

F107 ~ F111. UNFOLDING BRACKET QUESTIONS

F107. Would it amount to less than, about equal to or more than 500 MW (10,000 Korean won)?

[1] Less than 500 MW
[3] About 500 MW
[5] More than 500 MW

F108. Would it amount to less than, about equal to, or more than 1,000 MW (10,000 Korean won)?

[1] Less than 1,000 MW
[3] About 1,000 MW
[5] More than 1,000 MW

F109. Would it amount to less than, about equal to or more than 5,000 MW (10,000 Korean won)?

[1] Less than 5,000 MW
[3] About 5,000 MW
[5] More than 5,000 MW

F110. Would it amount to less than, about equal to or more than 10,000 MW (10,000 Korean won)?

[1] Less than 10,000 MW
[3] About 10,000 MW
[5] More than 10,000 MW

F111. Would it amount to a total of less than, about equal to or more than 30,000 MW (10,000 Korean won)?

[1] Less than 30,000 MW
[3] About 30,000 MW
[5] More than 30,000 MW

F112. Do you have any other insurance than health insurance we talked about earlier? I mean, do you have any term life insurance, whole life insurance, and annuity insurance?

> IWER: – Term Life Insurance: A type of life insurance which covers a certain period of time.
> – Whole Life Insurance: A type of life insurance which covers until the subscriber dies rather than a certain period of time.
> – Annuity Insurance: A type of life insurance in which the subscriber is being paid a certain amount until he/she dies or a certain period of time.

[1] Yes　　[5] No → Go to F138

F113. How many term life insurance policies do you currently have? Term life insurance refers to a life insurance which covers a certain period of time.

IWER: Mark 0 if R doesn't have any term life insurance.

______________ number of insurance (range: 0 ~ 5)

LOOP CHECKPOINT : TERM LIFE INSURANCE REPEAT QUESTION F114 ~ F117 ACCORDING TO THE NUMBER OF TERM LIFE INSURANCE GIVEN IN F113.

F114. What is the monthly premium for your term life insurance policy? (Unit: 10,000 Korean won)

______________MW (range: 1 ~ 999997)

F115. How often do you pay for term life insurance policy?

[1] Every month → Go to F117　　[5] Other

F116. Please specify other.

____

F117. In which year and month did you obtain the term life insurance policy?

IWER: Mark the year and month in 6 digits. For example, mark 200601 for January 2006. If the month is not clear, enter 00.

______________ (range: 190000 ~ 200612)

F118. How many whole life insurance policies do you have? Whole life insurance refers to a life insurance which covers until he/she dies.

IWER: Mark 0 if you are not covered by whole life insurance.

______________ number of insurance (range: 0 ~ 5)

LOOP CHECKPOINT : WHOLE LIFE INSURANCE REPEAT QUESTION F119 ~ F123 ACCORDING TO THE NUMBER OF WHOLE LIFE INSURANCE GIVEN IN F118.

F119. What is the premium for the whole life insurance policy? (Unit: 10,000 Korean won)

______________MW (range: 1 ~ 999997)

F120. How often do you pay for whole life insurance policy?

[1] Every month → Go to F122

[5] Other

F121. Please specify other.

____

F122. In which year and month did you obtain whole life insurance policy?

IWER: Mark the year and month in 6 digits. For example, mark 200601 for January 2006. If the month is not clear, enter 00.

______________ (range: 190000 ~ 200612)

F123. What is the face value of whole life insurance policy, that is, the amount of money the beneficiary would get if you were to die? (in case of natural death only – not accidental death) (Unit: 10,000 Korean won)

______________MW (range: 1 ~ 999997)

F124. How many annuity insurance policies do you have? Annuity insurance refers to a life insurance which the subscriber is being paid until he / she dies or for a certain period of time.

IWER: Mark 0 if you don't have any annuity insurances.

______________number of policies (range: 0 ~ 5)

LOOP CHECKPOINT : ANNUITY INSURANCE REPEAT QUESTION F125 ~ F130 ACCORDING TO THE NUMBER OF ANNUITY INSURANCE GIVEN IN F124.

F125. What is the premium you pay for the annuity insurance policy? (Unit: 10,000 Korean won)

______________MW (range: 1 ~ 999997)

F126. How often do you pay for annuity insurance policy?

[1] Every month → Go to F128　　[5] Other

F127. Please specify other.

____

F128. In which year and month did you obtain the annuity insurance policy?

IWER: Mark the year and month in 6 digits. For example, mark 200601 for January 2006. If the month is not clear, enter 00.

______________ (range: 190000 ~ 200612)

F129. At what age do you expect to start receiving the annuity from your annuity insurance policy?

______________years old (range: 45 ~ 65)

F130. What is the average monthly annuity payment you expect to receive from your annuity insurance? (Unit: 10,000 Korean won)

______________MW (range: 1 ~ 999997)

F131. Among those term－life, whole－life, and annuity insurance policies you have mentioned earlier, how many have you paid up?

______________ (range: 0 ~ 20)

F132. What is the average monthly annuity payment you expect to receive in total from all the annuity insurance policies you have? (Unit: 10,000 Korean won)

______________MW (range: 1 ~ 9997) → Go to F138

[98] Don't know
[99] Refuse to answer } → Go to F133~F137 unfolding

F133 ~ F137. UNFOLDING BRACKET QUESTIONS

F133. Would it amount to less than, about equal to or more than 50 MW (10,000 Korean won)?
[1] Less than 50 MW
[3] About 50 MW
[5] More than 50 MW

F134. Would it amount to less than, about equal to or more than 100 MW (10,000 Korean won)?
[1] Less than 100 MW
[3] About 100 MW
[5] More than 100 MW

F135. Would it amount to less than, about equal to or more than 300 MW (10,000 Korean won)?
[1] Less than 300 MW
[3] About 300 MW
[5] More than 300 MW

F136. Would it amount to less than, about equal to or more than 500 MW (10,000 Korean won)?
[1] Less than 500 MW
[3] About 500 MW
[5] More than 500 MW

F137. Would it amount to a total of less than, about equal to or more than 1,000 MW (10,000 Korean won)?
[1] Less than 1,000 MW
[3] About 1,000 MW
[5] More than 1,000 MW

* Owing *

F138. Does anyone owe you any money from personal loans you have made to others, including family members?

IWER: Only include loans that will be repaid

[1] Yes   [5] No → Go to F145

F139. If you added up all those loans, about how much would they amount to right now? (Unit: 10,000 Korean won)

______________MW (range: 1 ~ 999997) → Go to F145
[98] Don't know
[99] Refuse to answer } → Go to F140~F144 unfolding

F140 ~ F144. UNFOLDING BRACKET QUESTIONS

F140. Would it amount to less than, about equal to or more than 100 MW (10,000 Korean won)?
[1] Less than 100 MW
[3] About 100 MW
[5] More than 100 MW

F141. Would it amount to less than, about equal to, or more than 500 MW (10,000 Korean won)?
[1] Less than 500 MW
[3] About 500 MW
[5] More than 500 MW

F142. Would it amount to less than, about equal to or more than 1,000 MW (10,000 Korean won)?
[1] Less than 1,000 MW
[3] About 1,000 MW
[5] More than 1,000 MW

F143. Would it amount to less than, about equal to or more than 5,000 MW (10,000 Korean won)?
[1] Less than 5,000 MW
[3] About 5,000 MW
[5] More than 5,000 MW

F144. Would it amount to a total of less than, about equal to or more than 10,000 MW (10,000 Korean won)?
[1] Less than 10,000 MW
[3] About 10,000 MW
[5] More than 10,000 MW

F145. Are you currently a member of any private savings club (Gye)?
[1] Yes   [5] No → Go to F158

F146. How much money have you saved through these savings club (Gye) so far? (Refers to asset value; Exclude the amount that you have already taken out) (Unit: 10,000 Korean won)

______________MW (range: 0 ~ 99997) → Go to F152
[98] Don't know
[99] Refuse to answer } → Go to F147~F151 unfolding

F147 ~ F151. UNFOLDING BRACKET QUESTIONS

F147. Did it amount to less than, about equal to or more than 500 MW (10,000 Korean won)?

[1] Less than 500 MW
[3] About 500 MW
[5] More than 500 MW

F148. Did it amount to less than, about equal to or more than 1,000 MW (10,000 Korean won)?

[1] Less than 1,000 MW
[3] About 1,000 MW
[5] More than 1,000 MW

F149. Did it amount to less than, about equal to or more than 3,000 MW (10,000 Korean won)?

[1] Less than 3,000 MW
[3] About 3,000 MW
[5] More than 3,000 MW

F150. Did it amount to less than, about equal to or more than 5,000 MW (10,000 Korean won)?

[1] Less than 5,000 MW
[3] About 5,000 MW
[5] More than 5,000 MW

F151. Did it amount to a total of less than, about equal to or more than 10,000 MW (10,000 Korean won)?

[1] Less than 10,000 MW
[3] About 10,000 MW
[5] More than 10,000 MW

F152. Have you taken out from the mutual savings club (*Gye*) and still have some money owed? If so, how much do you still owe? (Unit: 10,000 Korean won)

_____________MW (range: 0 ~ 99997) → Go to F158

[98] Don't know
[99] Refuse to answer } → Go to F153~F157 unfolding

F153 ~ F157. UNFOLDING BRACKET QUESTIONS

F153. Does it amount to less than, about equal to or more than 500 MW (10,000 Korean won)?

[1] Less than 500 MW
[3] About 500 MW
[5] More than 500 MW

F154. Does it amount to less than, about equal to or more than 1,000 MW (10,000 Korean won)?

[1] Less than 1,000 MW
[3] About 1,000 MW
[5] More than 1,000 MW

F155. Does it amount to less than, about equal to or more than 3,000 MW (10,000 Korean won)?

[1] Less than 3,000 MW
[3] About 3,000 MW
[5] More than 3,000 MW

F156. Does it amount to less than, about equal to or more than 5,000 MW (10,000 Korean won)?

[1] Less than 5,000 MW
[3] About 5,000 MW
[5] More than 5,000 MW

F157. Does it amount to a total of less than, about equal to or more than 10,000 MW (10,000 Korean won)?

[1] Less than 10,000 MW
[3] About 10,000 MW
[5] More than 10,000 MW

F158. (Aside from anything you have already told me about) Do you have any other financial assets?

[1] Yes [5] No → Go to F166

F159. What type of financial assets is it?

\---

F160. What is the current value of these financial assets? If you sold all of them, about how much would you have? (Unit: 10,000 Korean won)

______________MW (rane: 1 ~ 999997) → Go to F166
[98] Don't know
[99] Refuse to answer } → Go to F161~F165 unfolding

F161 ~ F165. UNFOLDING BRACKET QUESTIONS

F161. Would it amount to less than, about equal to or more than 500 MW (10,000 Korean won)?
[1] Less than 500 MW
[3] About 500 MW
[5] More than 500 MW

F162. Would it amount to less than, about equal to, or more than 1,000 MW (10,000 Korean won)?
[1] Less than 1,000 MW
[3] About 1,000 MW
[5] More than 1,000 MW

F163. Would it amount to less than, about equal to or more than 3,000 MW (10,000 Korean won)?
[1] Less than 3,000 MW
[3] About 3,000 MW
[5] More than 3,000 MW

F164. Would it amount to less than, about equal to or more than 5,000 MW (10,000 Korean won)?
[1] Less than 5,000 MW
[3] About 5,000 MW
[5] More than 5,000 MW

F165. Would it amount to a total of less than, about equal to or more than 10,000 MW (10,000 Korean won)?
[1] Less than 10,000 MW
[3] About 10,000 MW
[5] More than 10,000 MW

F166. During the PAST YEAR(2005), how much interest per month did you earn from the financial assets you mentioned? (Unit: 10,000 Korean won)

______________MW (range: 0 ~ 99997) → Go to F172
[98] Don't know
[99] Refuse to answer } → Go to F167~F171 unfolding

F167 ~ F171. UNFOLDING BRACKET QUESTIONS

F167. Did it amount to less than, about equal to or more than 50 MW (10,000 Korean won)?
- [1] Less than 50 MW
- [3] About 50 MW
- [5] More than 50 MW

F168. Did it amount to less than, about equal to or more than 100 MW (10,000 Korean won)?
- [1] Less than 100 MW
- [3] About 100 MW
- [5] More than 100 MW

F169. Did it amount to less than, about equal to or more than 300 MW (10,000 Korean won)?
- [1] Less than 300 MW
- [3] About 300 MW
- [5] More than 300 MW

F170. Did it amount to less than, about equal to or more than 500 MW (10,000 Korean won)?
- [1] Less than 500 MW
- [3] About 500 MW
- [5] More than 500 MW

F171. Did it amount to a total of less than, about equal to or more than 1,000 MW (10,000 Korean won)?
- [1] Less than 1,000 MW
- [3] About 1,000 MW
- [5] More than 1,000 MW

F172. Do you own any vehicles, such as cars, trucks, a trailer, a boat, or any other means of transportation?

[1] Yes　　　[5] No → Go to F179

F173. If you sold all means of transportation you own today, how much would you get? What is the market value of all transportation means you own? (Unit: 10,000 Korean won)

______________MW → Go to F179

[98] Don't know
[99] Refuse to answer } → Go to F174~F178 unfolding

F174 ~ F178. UNFOLDING BRACKET QUESTIONS

F174. Would it amount to less than, about equal to or more than 500 MW (10,000 Korean won)?
[1] Less than 500 MW
[3] About 500 MW
[5] More than 500 MW

F175. Would it amount to less than, about equal to or more than 1,000 MW (10,000 Korean won)?
[1] Less than 1,000 MW
[3] About 1,000 MW
[5] More than 1,000 MW

F176. Would it amount to less than, about equal to or more than 3,000 MW (10,000 Korean won)?
[1] Less than 3,000 MW
[3] About 3,000 MW
[5] More than 3,000 MW

F177. Would it amount to less than, about equal to or more than 5,000 MW (10,000 Korean won)?
[1] Less than 5,000 MW
[3] About 5,000 MW
[5] More than 5,000 MW

F178. Would it amount to a total of less than, about equal to or more than 10,000 MW (10,000 Korean won)?
[1] Less than 10,000 MW
[3] About 10,000 MW
[5] More than 10,000 MW

F179. (Aside from those vehicles) Do you own any other assets, such as valuables, paintings, antiques, golf membership, or any assets that you haven’t already told us about?
[1] Yes [5] No → Go to F186

F180. What are they worth together? (Unit: 10,000 Korean won)

______________MW (range: 1 ~ 999997) → Go to F186
[98] Don’t know
[99] Refuse to answer
} → Go to F181~F185 unfolding

F181 ~ F185. UNFOLDING BRACKET QUESTIONS

F181. Does it amount to less than, about equal to or more than 500 MW (10,000 Korean won)?

[1] Less than 500 MW
[3] About 500 MW
[5] More than 500 MW

F182. Does it amount to less than, about equal to or more than 1,000 MW (10,000 Korean won)?

[1] Less than 1,000 MW
[3] About 1,000 MW
[5] More than 1,000 MW

F183. Does it amount to less than, about equal to or more than 3,000 MW (10,000 Korean won)?

[1] Less than 3,000 MW
[3] About 3,000 MW
[5] More than 3,000 MW

F184. Does it amount to less than, about equal to or more than 5,000 MW (10,000 Korean won)?

[1] Less than 5,000 MW
[3] About 5,000 MW
[5] More than 5,000 MW

F185. Does it amount to a total of less than, about equal to or more than 10,000 MW (10,000 Korean won)?

[1] Less than 10,000 MW
[3] About 10,000 MW
[5] More than 10,000 MW

F186. Have you (ever) received money or property in the form of an inheritance, a trust fund, or an insurance settlement?

[1] Yes
[5] No → Go to F197

F187. Regarding the largest amount of inheritance or transfer you received, in what form did you receive it? Was it an inheritance, an insurance settlement, or what?

[1] Insurance settlement
[2] Pension settlement
[3] Real estate
[4] Cash or financial assets
} → Go to F189
[5] Other

F188. Please specify other.

____

F189. From whom was that (inheritance / transfer) from? Please specify the relationship of giver to you?

[1] Spouse
[2] Mother
[3] Father
[4] Mother-in-law
[5] Father-in-law
[6] Sibling
[7] Brother-in-law, sister-in-law
[8] Child
[9] Spouse of child
[10] Grandchild
[11] Other relative

F190. In which year and month did you receive the inheritance/transfer from F189?

IWER: Mark the year and month using six digits. For example, mark 200601 for January 2006. If the month is not clear, enter 00.

______________ (range: 190000 ~ 200612)

F191. About how much did you receive? (Unit: 10,000 Korean won)

______________MW (range: 1 ~ 999997) → Go to F197

[98] Don't know
[99] Refuse to answer } → Go to F192~F196 unfolding

F192 ~ F196. UNFOLDING BRACKET QUESTIONS

F192. Did it amount to less than, about equal to or more than 1,000 MW (10,000 Korean won)?

[1] Less than 1,000 MW
[3] About 1,000 MW
[5] More than 1,000 MW

F193. Did it amount to less than, about equal to or more than 3,000 MW (10,000 Korean won)?

[1] Less than 3,000 MW
[3] About 3,000 MW
[5] More than 3,000 MW

F194. Did it amount to less than, about equal to or more than 5,000 MW (10,000 Korean won)?
[1] Less than 5,000 MW
[3] About 5,000 MW
[5] More than 5,000 MW

F195. Did it amount to less than, about equal to or more than 10,000 MW (10,000 Korean won)?
[1] Less than 10,000 MW
[3] About 10,000 MW
[5] More than 10,000 MW

F196. Did it amount to a total of less than, about equal to or more than 30,000 MW (10,000 Korean won)?
[1] Less than 30,000 MW
[3] About 30,000 MW
[5] More than 30,000 MW

F197. Next are questions about debts. Please respond to each debt category that applies to you. Debts include loans from financial institutions, personal loans from family and friends, suretyship obligations, and other debts.
First, do you have any loans from financial institution, such as banks, insurance companies, securities companies, or credit card companies?
[1] Yes [5] No → Go to F204

F198. About how much would that amount to? (Unit: 10,000 Korean won)

______________MW (range: 1 ~ 999997) → Go to F204
[98] Don't know
[99] Refuse to answer } → Go to F199~F203 unfolding

F199 ~ F203. UNFOLDING BRACKET QUESTIONS

F199. Did it amount to less than, about equal to or more than 1,000 MW (10,000 Korean won)?
[1] Less than 1,000 MW
[3] About 1,000 MW
[5] More than 1,000 MW

F200. Did it amount to less than, about equal to or more than 3,000 MW (10,000 Korean won)?
[1] Less than 3,000 MW
[3] About 3,000 MW
[5] More than 3,000 MW

F201. Did it amount to less than, about equal to or more than 5,000 MW (10,000 Korean won)?
[1] Less than 5,000 MW
[3] About 5,000 MW
[5] More than 5,000 MW

F202. Did it amount to less than, about equal to or more than 10,000 MW (10,000 Korean won)?
[1] Less than 10,000 MW
[3] About 10,000 MW
[5] More than 10,000 MW

F203. Did it amount to a total of less than, about equal to or more than 30,000 MW (10,000 Korean won)?
[1] Less than 30,000 MW
[3] About 30,000 MW
[5] More than 30,000 MW

F204. (Aside from the loans from financial institutions or credit card companies that you have already told me) Do you have any personal loans from relatives, friends or others?
[1] Yes [5] No → Go to F211

F205. About how much would that amount to? (Unit: 10,000 Korean won)

_____________MW (range: 1 ~ 99997) → Go to F211
[98] Don't know
[99] Refuse to answer } → Go to F206~F210 unfolding

F206 ~ F210. UNFOLDING BRACKET QUESTIONS

F206. Would it amount to less than, about equal to or more than 300 MW (10,000 Korean won)?
[1] Less than 300 MW
[3] About 300 MW
[5] More than 300 MW

F207. Would it amount to less than, about equal to or more than 500 MW (10,000 Korean won)?
[1] Less than 500 MW
[3] About 500 MW
[5] More than 500 MW

F208. Would it amount to less than, about equal to or more than 1,000 MW (10,000 Korean won)?

[1] Less than 1,000 MW
[3] About 1,000 MW
[5] More than 1,000 MW

F209. Would it amount to less than, about equal to or more than 3,000 MW (10,000 Korean won)?

[1] Less than 3,000 MW
[3] About 3,000 MW
[5] More than 3,000 MW

F210. Would it amount to a total of less than, about equal to or more than 6,000 MW (10,000 Korean won)?

[1] Less than 6,000 MW
[3] About 6,000 MW
[5] More than 6,000 MW

F211. And do you currently stand surety for the loans of relative, friend, or other?

[1] Yes [5] No → Go to F218

F212. About how much would that liability amount to? (Unit: 10,000 Korean won)

______________MW (range: 1 ~ 99997) → Go to F218

[98] Don't know
[99] Refuse to answer } → Go to F213~F217 unfolding

F213 ~ F217. UNFOLDING BRACKET QUESTIONS

F213. Would it amount to less than, about equal to or more than 500 MW (10,000 Korean won)?

[1] Less than 500 MW
[3] About 500 MW
[5] More than 500 MW

F214. Would it amount to less than, about equal to or more than 1,000 MW (10,000 Korean won)?

[1] Less than 1,000 MW
[3] About 1,000 MW
[5] More than 1,000 MW

F215. Would it amount to less than, about equal to or more than 3,000 MW (10,000 Korean won)?
[1] Less than 3,000 MW
[3] About 3,000 MW
[5] More than 3,000 MW

F216. Would it amount to less than, about equal to or more than 5,000 MW (10,000 Korean won)?
[1] Less than 5,000 MW
[3] About 5,000 MW
[5] More than 5,000 MW

F217. Would it amount to a total of less than, about equal to or more than 10,000 MW (10,000 Korean won)?
[1] Less than 10,000 MW
[3] About 10,000 MW
[5] More than 10,000 MW

F218. And do you have any other loans that we haven't talked about?
[1] Yes [5] No → Go to F226

F219. What type of debts is it?

F220. About how much in total would that amount to? (Unit: 10,000 Korean won)

______________MW (range: 1 ~ 99997) → Go to F226
[98] Don't know
[99] Refuse to answer } → Go to F221~F225 unfolding

F221 ~ F225. UNFOLDING BRACKET QUESTIONS

F221. Would it amount to less than, about equal to or more than 300 MW (10,000 Korean won)?
[1] Less than 300 MW
[3] About 300 MW
[5] More than 300 MW

F222. Would it amount to less than, about equal to or more than 500 MW (10,000 Korean won)?
[1] Less than 500 MW
[3] About 500 MW
[5] More than 500 MW

F223. Would it amount to less than, about equal to or more than 1,000 MW (10,000 Korean won)?
[1] Less than 1,000 MW
[3] About 1,000 MW
[5] More than 1,000 MW

F224. Would it amount to less than, about equal to or more than 3,000 MW (10,000 Korean won)?
[1] Less than 3,000 MW
[3] About 3,000 MW
[5] More than 3,000 MW

F225. Would it amount to a total of less than, about equal to or more than 6,000 MW (10,000 Korean won)?
[1] Less than 6,000 MW
[3] About 6,000 MW
[5] More than 6,000 MW

F226. Do any other household members younger than 45 living in your household have more than 500 MW (10,000－Korean won) total in bank account, other financial assets, vehicles, properties, or other investments?
[1] Yes [5] No → Go to F233

F227. About how much altogether would their net-worth amount to? (DO NOT INCLUDE ASSETS OF R OR OTHER HOUSEHOLD MEMBERS AGED OVER 45) (Unit: 10,000 Korean won)
IWER: Please state the net asset value minus any debts held.

_____________MW (range: 1 ~ 999997) → Go to F233
[98] Don't know
[99] Refuse to answer } → Go to F228~F232 unfolding

F228 ~ F232. UNFOLDING BRACKET QUESTIONS

F228. Would it amount to less than, about equal to or more than 1,000 MW (10,000 Korean won)?
[1] Less than 1,000 MW
[3] About 1,000 MW
[5] More than 1,000 MW

F229. Would it amount to less than, about equal to or more than 3,000 MW (10,000 Korean won)?
[1] Less than 3,000 MW
[3] About 3,000 MW
[5] More than 3,000 MW

F230. Would it amount to less than, about equal to or more than 5,000 MW (10,000 Korean won)?
[1] Less than 5,000 MW
[3] About 5,000 MW
[5] More than 5,000 MW

F231. Would it amount to less than, about equal to or more than 10,000 MW (10,000 Korean won)?
[1] Less than 10,000 MW
[3] About 10,000 MW
[5] More than 10,000 MW

F232. Would it amount to a total of less than, about equal to or more than 30,000 MW (10,000 Korean won)?
[1] Less than 30,000 MW
[3] About 30,000 MW
[5] More than 30,000 MW

F233. IWER: HOW OFTEN DID R RECEIVE ASSISTANCE WITH ANSWERS IN SECTION F-ASSETS AND DEBTS?
[1] Never
[2] A few times
[3] Most or all the time
} → Go to Section G.
[4] The Section was done by a proxy reporter.

F234. IWER: WHAT IS THE PROXY'S RELATIONSHIP TO R? IF UNKNOWN, PLEASE ASK THE PROXY. What is your relationship to R?
[1] Spouse
[2] Mother
[3] Father
[4] Mother-in-law
[5] Father-in-law
[6] Sibling
[7] Brother-in-law, sister-in-law
[8] Child
[9] Spouse of child
[10] Grandchild
[11] Other relative
[12] Helper or other non-relative
→ Go to Section G.

G. EXPECTATIONS AND LIFE SATISFACTION

G. EXPECTATIONS AND LIFE SATISFACTION SURVEY STRUCTURE

G001-G024 : SUBJECTIVE EXPECTATIONS

- G001-G006 : SUBJECTIVE EXPECTATIONS ABOUT ECONOMICAL SITUATION
  - G001 : CHANCE OF LEAVING AN INHERITANCE
  - G002 : CHANCE OF RECEIVING AN INHERITANCE
  - G003-G005 : EXPECTED WORK PERIOD
  - G006 : WORK IN THE FUTURE
- G007-G014 : LIFE EXPECTANCY BY AGE
- G015-G024 : EXPECTATION TO GOVERNMENT
  - G015 : LIVING STANDARD IMPROVEMENT IN THE FUTURE
  - G016-G018 : NATIONAL PENSION SYSTEM - ENROLLMENT, EXPECTED DAYS OF RECEIVING BENEFITS, THE AMOUNT OF BENEFITS
  - G019 : SOCIAL/ECONOMICAL SITUATION OF CHILDREN'S GENERATION
  - G020 : CHANCES THAT THE GOVERNMENT WILL PROVIDE OLD AGE SUPPORT
  - G021 : CHANCES THAT SOUTH AND NORTH KOREA WILL RE-UNIFIED
  - G022 : CHANCES THAT KOREAN ECONOMY EXPERIENCE MAJOR DEPRESSION
  - G023 : CHANCES THAT KOREAN REAL MARKET WILL BE STABILIZED ECONOMY EXPERIENCE MAJOR DEPRESSION
  - G024 : PROPENSITY TO MONEY CONSUMING

G025-G029 : LIFE SATISFACTION

- G025 : SATISFACTION WITH HEALTH STATUS
- G026 : SATISFACTION WITH ECONOMIC STATUS
- G027-G028 : SATISFACTION WITH RELATIONSHIP - SPOUSE, CHILDREN
- G029 : RELATIVE QUALITY OF LIFE

G030 : CHECK POINT OF INTERVIEWER

G031 : SURVEY FINISHED.

G001. Next we would like to ask your opinion about how likely you think various events might be. When I ask a question I'd like for you to give me a number from 0 to 100, where "0" means that you think there is absolutely no chance, and "100" means that you think the event is absolutely sure to happen. If it's hard to answer in numbers, take a look at the picture I show you and answer in accordance to that. Including property and other valuables that you might own, what are the chances that you will leave an inheritance totaling 10,000 MW (10,000 Korean won) or more? Please mark the level of chances you agree to the above statement using the scale below. 0 means absolutely no chance and 100 means absolutely certain.

0 10 20 30 40 50 60 70 80 90 100

SKIP PATTERN CHECKPOINT: INHERITANCE IF AT LEAST ONE OF R'S PARENTS IS ALIVE, GO TO G002.
OTHERWISE GO TO G003.

G002. How about the chances that you will receive an inheritance totaling 10,000 MW (10,000 Korean Won) or more? Please mark the level of chances you agree to the above statement using the scale below. 0 means absolutely no chance and 100 means absolutely certain.

0 10 20 30 40 50 60 70 80 90 100

SKIP PATTERN CHECKPOINT : EXPECTED WORK PERIOD IF R IS BETWEEN THE AGE OF 45~49 AND CURRENTLY WORKING, GO TO G003.
OTHERWISE, GO TO G004.

G003. What do you think the chances are that you will be working full-time after you reach age 55? Please mark the chances that you agree to the above statement using the scale below. 0 means absolutely no chance and 100 means absolutely certain.

0 10 20 30 40 50 60 70 80 90 100

**SKIP PATTERN CHECKPOINT : EXPECTED WORK PERIOD IF R IS BETWEEN THE AGE OF 50~54 AND CURRENTLY WORKING, GO TO G004.
OTHERWISE, GO TO G005.**

G004. What do you think the chances are that you will be working full−time after you reach age 60? Please mark the level of chances that you agree to the above statement using the scale below. 0 means absolutely no chance and 100 means absolutely certain.

0 10 20 30 40 50 60 70 80 90 100

**SKIP PATTERN CHECKPOINT : EXPECTED WORK PERIOD IF R IS 55 YEARS OLD AND CURRENTLY WORKING, GO TO G005.
OTHERWISE, GO TO G006.**

G005. What are the chances that you will be working for the present job for the next 5 years? Please mark the level of chances you agree to the above statement using the scale below. 0 means absolutely no chance and 100 means absolutely certain.

0 10 20 30 40 50 60 70 80 90 100

**SKIP PATTERN CHECKPOINT : EXPECTED WORK PERIOD IF R IS NOT CURRENTLY WORKING, GO TO G006.
OTHERWISE, GO TO G007.**

G006. What are the chances that you will be working for pay at some time in the future? Please mark the level of chances you agree to the above statement using the scale below. 0 means absolutely no chance and 100 means absolutely certain.

0 10 20 30 40 50 60 70 80 90 100

SKIP PATTERN CHECKPOINT: LIFE EXPECTANCY, G007 ~ G014 DEPENDING ON R'S AGE, R WILL BE ASKED TO ANSWER ONE OF THE FOLLOWING LIFE EXPECTANCY QUESTIONS: G007 ~ G014.

IF R IS UNDER THE AGE 64, GO TO G007.
IF R IS BETWEEN THE AGE OF 65~69, GO TO G008.
IF R IS BETWEEN THE AGE OF 70~74, GO TO G009.
IF R IS BETWEEN THE AGE OF 75~79, GO TO G010.
IF R IS BETWEEN THE AGE OF 80~84, GO TO G011.
IF R IS BETWEEN THE AGE OF 85~94, GO TO G012.
IF R IS BETWEEN THE AGE OF 95~99, GO TO G013.
IF R IS OVER THE AGE 100, GO TO G014.

G007. What is the percent chance that you will live to be 75 or more? Please mark the level of chances you agree to the above statement using the scale below. 0 means absolutely no chance and 100 means absolutely certain.

0 10 20 30 40 50 60 70 80 90 100

G008. What is the percent chance that you will live to be 80 or more? Please mark the level of chances you agree to the above statement using the scale below. 0 means absolutely no chance and 100 means absolutely certain.

0 10 20 30 40 50 60 70 80 90 100

G009. What is the percent chance that you will live to be 85 or more? Please mark the level of chances you agree to the above statement using the scale below. 0 means absolutely no chance and 100 means absolutely certain.

0 10 20 30 40 50 60 70 80 90 100

G010. What is the percent chance that you will live to be 90 or more? Please mark the level of chances you agree to the above statement using the scale below. 0 means absolutely no chance and 100 means absolutely certain.

0 10 20 30 40 50 60 70 80 90 100

G011. What is the percent chance that you will live to be 95 or more? Please mark the level of chances you agree to the above statement using the scale below. 0 means absolutely no chance and 100 means absolutely certain.

0 10 20 30 40 50 60 70 80 90 100

G012. What is the percent chance that you will live to be 100 or more? Please mark the level of chances you agree to the above statement using the scale below. 0 means absolutely no chance and 100 means absolutely certain.

0 10 20 30 40 50 60 70 80 90 100

G013. What is the percent chance that you will live to be 105 or more? Please mark the level of chances you agree to the above statement using the scale below. 0 means absolutely no chance and 100 means absolutely certain.

0 10 20 30 40 50 60 70 80 90 100

G014. What is the percent chance that you will live to be 110 or more? Please mark the level of chances you agree to the above statement using the scale below. 0 means absolutely no chance and 100 means absolutely certain.

0 10 20 30 40 50 60 70 80 90 100

G015. What are the chances that your financial situation will be worse? Please mark the level of chances you agree to the above statement using the scale below. 0 means absolutely no chance and 100 means absolutely certain.

0 10 20 30 40 50 60 70 80 90 100

G016. Are you a beneficiary of National Public Pension System?

[1] Yes [5] No → Go to G019

G017. When will you start receiving National Public Pension benefits?

[1] 55 – 59 years old
[2] 60 – 64 years old
[3] 64 – 69 years old
[4] Over 70 years old

G018. How much do you expect to receive? Please answer it using the present value. (Unit: 10,000 Korean won)

______________MW (range: 1 ~ 997)

G019. What are the chances that our children's generation will live in better economic / social situation than your generation does? Please mark the level of chances you agree to the above statement using the scale below. 0 means absolutely no chance and 100 means absolutely certain.

0 10 20 30 40 50 60 70 80 90 100

G020. What are the chances that the government will provide old age support when you get older? Please mark the level of chances you agree to the above statement using the scale below. 0 means absolutely no chance and 100 means absolutely certain.

0 10 20 30 40 50 60 70 80 90 100

G021. What are the chances that South and North Korea will be re-united in the next 10 years? Please mark the level of chances you agree to the above statement using the scale below. 0 means absolutely no chance and 100 means absolutely certain.

0 10 20 30 40 50 60 70 80 90 100

G022. What do you think are the chances that Korean economy will experience a major depression sometime during the next 10 years? Please mark the level of chances you agree to the above statement using the scale below. 0 means absolutely no chance and 100 means absolutely certain.

0 10 20 30 40 50 60 70 80 90 100

G023. What do you think are the chances that Korean real estate market will be stabilized within the next 10 years? Please mark the level of chances you agree to the above statement using the scale below. 0 means absolutely no chance and 100 means absolutely certain.

0 10 20 30 40 50 60 70 80 90 100

G024. Suppose that you suddenly earned 1,000 MW (10,000-Korean won). Where would you spend the money? Mark your answer after reviewing the example below. All the money can be spent in one spot.

Where	Amount
G024A. Savings or investment	
G024B. Pay debt	
G024C. Give to children or relatives or donate	
G024D. Buy house, car, furniture, electric appliances etc.	
G024E. Travel and other leisure activities	
Total<br>(must add up to 1,000 MW (10,000-Korean won)	1,000 MW<br>(10,000－Korean won)

G025. Now I'm going to ask you about satisfaction with life. Please answer how much you are satisfied with the followings compared to your contemporaries. 0 means absolutely dissatisfied and 100 means absolutely satisfied.
How satisfied are you with your health?

0 10 20 30 40 50 60 70 80 90 100

G026. How satisfied are you with your economic status?

0 10 20 30 40 50 60 70 80 90 100

G027. How satisfied are you with the relationship with your spouse?

0 10 20 30 40 50 60 70 80 90 100

G028. How satisfied are you with the relationship with your children?

0 10 20 30 40 50 60 70 80 90 100

G029. In overall, how satisfied are you with your quality of life (or how happy you feel)?

0 10 20 30 40 50 60 70 80 90 100

G030. IWER: HOW OFTEN DID R RECEIVE ASSISTANCE WITH ANSWERS IN SECTION G – EXPECTATIONS AND LIFE SATISFACTION?

[1] Never
[2] A few times
[3] Most or all the time
} → Go to G032
[4] The Section was done by a proxy reporter.

G031. IWER: WHAT IS THE PROXY'S RELATIONSHIP TO R? IF UNKNOWN, PLEASE ASK THE PROXY. What is your relationship to R?

[1] Spouse
[2] Mother
[3] Father
[4] Mother-in-law

[5] Father-in-law
[6] Sibling
[7] Brother-in-law, sister-in-law
[8] Child
[9] Spouse of child
[10] Grandchild
[11] Other relative
[12] Helper or other non-relative

G032. This is the end of the survey. We truly appreciate your time and participation in our study. As noted earlier, the contents of this survey will be kept strictly confidential. We prepared a small gift as a token of our gratitude. Again, thank you very much for the interview.

[1] Confirm

KLoSA

Korean Longitudinal Study of Ageing

2006 KLoSA Wave1 Questionnaire

KOREA LABOR INSTITUTE
