# Supplementary material for: Which type of social activities may reduce cognitive decline in the elderly?: a longitudinal population-based study
Source: BMC Geriatr. 2016 Sep 27;16:165. doi: 10.1186/s12877-016-0343-x (PMC5039914; doi:10.1186/s12877-016-0343-x)
Supplement: Additional file 3: — 2wave KLoSA Questionnaire English version_2008. (PDF 1412 kb) [file 12877_2016_343_MOESM3_ESM.pdf]

2008 KLoSA

Wave 2 Questionnaire

SURVIVAL INTERVIEW

TABLE OF CONTENTS

- CV. COVER SCREEN -- 2
- A. DEMOGRAPHICS -- 6
- Ba. FAMILY AND FAMILY TRANSFER (CHILDREN AND GRAND CHILDREN) ------------------------------15
- Bb. FAMILY AND FAMILY TRANSFER (PARENTS AND SIBLINGS) --34
- C1. HEALTH STATUS ---83
- C2. FUNTIONAL LIMITATIONS AND HELPERS ---110
- C3. HEALTH INSURANCE AND SERVICES --117
- C4. COGNITION --129
- C5. GRIP STRENGTH ---134
- D. EMPLOYMENT --137
- E. INCOME AND CONSUMPTION --211
- F. ASSETS AND DEBTS ---251
- G. EXPECTATIONS AND LIFE SATISFACTION ---298

CV. COVERSCREEN

Logic

■ COVERSCREEN section is answered by one panel member in the household.

■ Any panel member who has moved out or set up a separate household (distinguished in GPMS) is given a new household ID to indicate that fact.

■ If a panel member has died, skip COVERSCREEN section and conduct an exit interview with a proxy respondent.

CV001. We conduct the 2nd Wave of the KLoSA. Thank you very much for your co-operation in this survey. You will be asked about the changes since the previous interview. Is [a respondent name] your name?

① Yes →Go to CV003 ⑤ No

CV002. What is your name?

____

CV003. Your gender is [a respondent's gender]. Isn't it?

① Yes →Go to CV004 ⑤ No

CV018. What is your gender?

① Male

⑤ Female

CV004. Does non-panel household member live in your house?

The definition of household members: the ones who reside in the same housing unit and related with blood or marriage (co-habitation included).

[The followings are considered as a household member]

① a spouse residing separately due to the workplace, etc.

② child(ren) who is(are) financially dependent but residing separately due to the education, work, etc.

③ relatives who reside together

[The followings are NOT considered as a household member]

① unmarried child(ren) who is(are) financially independent and residing separately/married child(ren) who resides(reside) separately

② temporarily visiting or residing relative(s)

③ non-relatives such as a housemaid, chauffeur, or boarder

④ family who reside separately due to the army service, working or studying abroad, or long - term institutionalization

① Yes ⑤ No →Go to CV013

Logic

■ Loop through CV005 ~CV011 as many times as the number of non-panel household members.

CV005. What is his/her name?

____

CV006. What is [CV005_HH MEMBER's name]'s gender?

[IWER: Ask only if distinguishing gender is difficult and if not, make an educated guess]

① Male

⑤ Female

CV007. What is [CV005_HH MEMBER's name]'s relationship to you?

⓪ Input End

① Father/mother

② Father-in-law/mother-in-law

③ Spouse

④ Son/daughter

⑤ Son-in-law/daughter-in-law

⑥ Brother/sister

⑦ Grandchild

⑧ Child of brother/sister

⑨ Brother-in-law/sister-in-law

⑩ Child of brother-in-law/sister-in-law

⑪ Spouse of grandchild

⑫ Great-grandchild

⑬ Others

CV009. In what year was [CV005_HH MEMBER's name] born?

_____________ [range:1900~2008]

CV010. What is [CV005_HH MEMBER's name]'s marital status?

[IWER: common-law marriage is considered as married]

① Married or living with a spouse

③ Separated / Divorced / Widowed

⑤ Never married

CV011. Dose any other non-panel household live with you?

① Yes →Go to CV013 ⑤ No

Logic

■ CV005 ~ CV011 Non-panel household member

CV012. Currently, you live total [the number of non-panel household member]. Dose any other non-panel household live with you?

[IWER: Total Check. You have talked about non-panel household members [number of non-panel household members].

① Yes, add non-panel household member → CV011 ⑤ No

Logic

■ If CV013=⑤, then go to CV017. Until CV013=⑤, loop CV013~CV016.

CV013. Have you moved since the previous interviews?

- If the respondent has changed actual living place(but address is not changed officially), it has to admit moving

① Yes

⑤ No →Go to CV017

CV014. When did you move? (unit: year and month combined into 6 digits)

[IWER:

- "If you have moved twice or more, please start with the most recent one."

- Enter the year and month using 6 digits. For example, mark 200801 for January 2008. If the month is not clear, enter 200800.]

____________________ [range:200600~200812]

CV015. Why did you move?

① Because R or R's spouse moved to a new job/workplace →Go to CV013

② Because any family member other than R or R's spouse moved to a new job/workplace → Go to CV013

③ Due to education for children or grandchildren →Go to CV013

④ To seek a better residential environment →Go to CV013

⑤ To sell up the house and invest that money in other assets →Go to CV013

⑥ To sell up the house and spend that money on living expenses →Go to CV013

⑦ (In case of deposit-based rental house or monthly rental house) Due to the expiration of the contract →Go to CV013

(97) Others

CV016. If others, please specify.

____

A. DEMOGRAPHICS

A001. First, I will confirm the basic information you provided in the previous interview. Is your date of birth (yyyy/mm/dd)?

[IWER: The date of birth is based on the actual date of birth not the one stated in the resident registration card or family register.]

① Yes →Go to A004

⑤ No

A002. If no, please tell me your exact date of birth.

[IWER: For those who respond by a zodiac sign, refer to the index card provided.]

_____Year _____Month _____Day

A003. Is your date of birth according to the solar calendar or the lunar calendar?

① Solar calendar

⑤ Lunar calendar

- Level of Education

A004. In the previous interview you responded that you had completed [level of education] and [graduation status]. Is this correct?

① Yes →Go to A008

⑤ No

A005. What is the highest level of education you have completed?

① No formal education (illiterate) →Go to A008

② No formal education (capable of reading) →Go to A008

③ Elementary school →Go to A007

④ Middle school →Go to A007

⑤ High school →Go to A007

⑥ Two-year college →Go to A007

⑦ College grad →Go to A007

⑧ Post college (Master) →Go to A007

⑨ Post college (PhD) →Go to A007

(97) Others

(-9) Don't know →Go to A011

(-8) Refuse to answer →Go to A011

A006. If others, please specify.

___________________ →Go to A008

A007. Did you graduate, drop out or obtain an equivalent qualification by passing the qualification exam though not completing the school? Please answer only about the qualification you acquired through graduation or the qualification exam except the one you are now pursuing.

① Currently attending

② Graduated

③ Dropped out

④ Completed

⑤ Obtained an equivalent qualification through the qualification exam

A008. Have you attained further formal education since the previous interview?

[IWER: In case of 'currently attending', choose '⑤ No' because R has not earned a degree yet.]

① Yes

⑤ No →Go to A011

A009. What is the level of formal education you have attained?

③ Elementary school →Go to A011

④ Middle school →Go to A011

⑤ High school →Go to A011

⑥ Two-year college →Go to A011

⑦ College grad →Go to A011

⑧ Post college (Master) →Go to A011

⑨ Post college(PhD) →Go to A011

(97) Others

(-9) Don't know →Go to A011

(-8) Refuse to answer →Go to A011

A010. If others, please specify.

____

- Marital Status

A011. In the previous interview you responded that your marital status was [marital status]. Is this

correct?

[IWER: This question is intended to check R's marital status at the time of the previous interview, not now.]

① Yes →Go to A013

⑤ No

A012. What was your exact marital status at that time?

① Married or living with a partner

② Separated

③ Divorced

④ Widowed or missing (dispersed family)

⑤ Never married

Logic

■ If A013=①, then go to A014. Until A013=⑤, loop A013~A015.

A013. Has your marital status changed since the previous interview?

[IWER: Marital status includes divorce, separation by death, separation, marriage, etc.]

① Yes

⑤ No →Go to A016

A014. How has it changed? Please describe all the changes in a time sequence.

[IWER: "For example, if you got divorced and then married again, please say divorce first."]

① Married

② Separated

③ Divorced

④ Widowed

A015. When did you get [response in A014]? (unit: year and month combined into 6 digits)

[IWER: Enter the year and month using 6 digits. For example, mark 200701 for January 2007. If the month is not clear, enter 200700.]

____________________ [range:200600~200811]

Logic

■ If R has a spouse now, then go to A016.

A016. Did your current spouse participate in the previous KLoSA survey?

① Yes →Go to A030

⑤ NO →Go to A017 (If R has the same spouse now as at the time of the previous interview) or A018(If R has a new spouse)

Logic

■ A017~A029 are questions to get basic information on R's spouse. If the spouse is currently married to R and non-panel, then go to A017~A029.

■ If R's spouse is a panel member, then go to A030.

A017. Is your spouse's name [spouse's name]?

[IWER: If the spouse's name is not showed up on the screen, check '⑤ No' and mark the information to next item.]

① Yes →Go to A019

⑤ NO

A018. If no, what is your spouse's name?

[IWER: if the R does not want to tell the spouse's name, just write on 'a spouse'.]

____

A019. Is your spouse's year of birth [year of birth]?

[IWER: For those who respond by a zodiac sign, refer to the index card provided]

① Yes →Go to A021

⑤ NO

Logic

■ If R has the same spouse now as at the time of the previous interview, then go to A021~A029. If R has a new spouse, then go to A020, A022~A024, A028~A029.

A020. If no, please tell me your spouse [spouse's name]'s exact year of birth again.

I am going to ask about your spouse [spouse's name]'s year of birth and level of education.

_____year [range:1900~1990]

Logic

■ If R has the same spouse now as at the time of the previous interview, then go to A021. If R has a

new spouse, then go to A022

A021. You responded that your spouse [spouse's name] had completed [level of education] and [graduation status]. Is this correct?

[IWER: If the spouse's name is not showed up on the screen, check '⑤ No' and mark the information to next item.]

① Yes →go to A025

⑤ No

A022. What is the highest level of education your spouse has completed?

① No formal education (illiterate) →Go to A025

② No formal education(capable of reading) →Go to A025

③ Elementary school →Go to A024

④ Middle school →Go to A024

⑤ High school →Go to A024

⑥ Two-year College →Go to A024

⑦ College grad →Go to A024

⑧ Post college (Master) →Go to A024

⑨ Post college(PhD) →Go to A024

(97) Others

(98) Don't know →Go to A025

(99) Refuse to answer →Go to A025

A023. If others, please specify.

___________________ →Go to A025

A024. Did your spouse graduate, drop out or obtain an equivalent qualification by passing the qualification exam though not completing the school?

① Currently attending

② Graduated

③ Dropped out

④ Completed

⑤ Obtained an equivalent qualification through the qualification exam

Logic

■ If R has the same spouse now as at the time of the previous interview, then go to A025. If R has a

new spouse, then go to A028.

A025. Has your spouse attained further formal education since the previous interview?

① Yes

⑤ NO →Go to A028

A026. What is the level of formal education your spouse has attained?

③ Elementary school →Go to A028

④ Middle school →Go to A028

⑤ High school →Go to A028

⑥ Two-year College →Go to A028

⑦ College grad →Go to A028

⑧ Post college (Master) →Go to A028

⑨ Post college (PhD) →Go to A028

(97) Others

(-9) Don't know →Go to A028

(-8) Refuse to answer →Go to A028

A027. If others, please specify.

____

A028. Now I would like to know about your spouse's current employment situation. Please answer if your spouse is currently employed, temporarily laid off, unemployed, looking for work, retired, or has never been employed. If she/he is employed, please answer if she/he works full time or part time.
[IWER: Retirement refers to having stopped doing income-earning activities and presently not working or engaging only in pastime work, and having no intention of engaging in anything more serious than pastime work as long as there is no special change in circumstances.]

① Full-time worker →Go to A030

② Part-time worker →Go to A030

③ Temporarily laid off on sick or other leave →Go to A030

④ Unemployed and looking for work →Go to A030

⑤ Retired →Go to A030

⑥ Homemaker →Go to A030

(97) Others

A029. If others, please specify.

____

- Religion and Social Relationship and Social Activity

A030.Do you have a religion? If you have, what kind of religion do you have?

① No religion →Go to A032

② Protestantism →Go to A032

③ Catholicism →Go to A032

④ Buddhism →Go to A032

⑤ Won Buddhism →Go to A032

⑥ Others

A031. If others, please specify.

____

A032. Do you have any friends or relatives who live nearby and have a close relationship with you? If so, how often do you meet them in person? Please refer to the person you meet most often.

① Almost every day (more than 4 times per week)

② Once a week

③ 2-3 times a week

④ Once a month

⑤ Twice a month (every two weeks)

⑥ Once or twice a year

⑦ Three or four times a year (once every three or four months)

⑧ Five or six times a year (every two months)

⑨ Almost never a year

⑩ No close friend or relative

A033. Are you a member of any of the following organizations, clubs, or societies?
Please select all that apply.

① Religious groups →Go to A035

② Social clubs (e.g. private savings club, senior citizens' club, etc) →Go to A035

③ Leisure/culture/sports related groups (class for the elderly, etc.) →Go to A035

④ Alumni associations, hometown communities, family councils →Go to A035

⑤ Volunteer groups →Go to A035

⑥ Political parties, NGOs, interest groups →Go to A035

⑦ Others

⑧ None →Go to A036

(-9) Don't know →Go to A036

(-8) Refuse to answer →Go to A036

A034. If others, please specify.

____

Logic

■ Repeat A035 as many times as the number chosen in A033.

A035. As for the organization [chosen in A033] of which you are a member, how often do you engage in its activities?

① Almost every day (more than 4 times per week)

② Once a week

③ 2-3 times a week

④ Once a month

⑤ Twice a month (every two weeks)

⑥ Once or twice a year

⑦ Three or four times a year (once every three or four months)

⑧ Five or six times a year (every two months)

⑨ Almost never a year

⑩ Almost never

A036. [IWER: How often did R receive assistance in answering section A-DEMOGRAPHICS?]

① Never → Go to section Ba.

② A few times → Go to section Ba.

③ Most or all of the time → Go to section Ba.

④ The section was done by a proxy respondent

A037. What is the proxy's relationship to R? If unknown, please ask the proxy. What is your relationship to R?

① Spouse

② Mother

③ Father

④ Mother-in-law

⑤ Father-in-law

⑥ Brother/sister

⑦ Brother-in-law/sister-in-law

⑧ Son/daughter

⑨ Son-in-law/Daughter-in-law

⑩ Grandchild

⑪ Other relatives

⑫ Helper or other non-relatives

→Go to section Ba.

Ba. FAMILY AND FAMILY TRANSFER (CHILDREN & GRANDCHILDREN)

Logic

■ If R answered 'Refuse to answer/Don't know' in the previous interview, then go to Ba003. Else, go to Ba001.

■ Among the couple, only the representative respondent answerable for CHILDREN section is asked Ba001~Ba017.

Ba001. Now I am going to ask you about your children. In the previous interview, you responded that you had [no. of children] children. Is this correct?

[IWER: According to the previous interview, if the number of children is same but member of children is different, check '⑤ No']

① Yes →Go to Ba003

⑤ No

Ba002. How many children did you have at the time of the previous interview?

(unit: number of children) _____

Ba003. How many children are currently alive? (unit: number of children)

[IWER: Any child who is not confirmed dead since he/she is missing or has run away from home, etc., is considered currently alive.]

_____ →Go to Ba004(If Ba001=① and the number of children reported in the previous interview>Ba003)

→Go to Ba006(If Ba001=⑤ or if R answered 'Don't know' or 'Refuse to answer' about the number of children in the previous interview or if the number of children has increased)

→Go to Ba008(If Ba001=① and the number of children reported in the previous interview=Ba003)

→Go to Ba068(If the number of children reported in the previous interview=0 and Ba003=0)

Don't know →Go to Ba068

Refuse to answer →Go to Ba068

Logic

■ If the number of children reported in the previous interview>Ba003, then go to Ba004.

Ba004. Who are those dead children? Please list all of them. (Select multiple responses.)
01~10 [child's name]

Logic
■ Repeat Ba005 as many times as the number of children reported in Ba005.

Ba005. When did [child's name selected in Ba004] die? (unit: year and month combined into 6 digits)
[IWER: Enter the year and month using 6 digits. For example, mark 200701 for January 2007. If the month is not clear, enter 200700.]
____________________ [range:200600~200812]

Logic
■ If (Ba001=⑤ and Ba003>=1), or (R answered 'Don't know/Refuse to answer' about the number of children in the previous interview and Ba003>=1), or (the number of children has increased since the previous interview), go to Ba006. Else, then go to Ba008.
■ Loop through the following questions as many times as the number of children reported in Ba003 or the number in Ba003 – the number of children reported in the previous interview.

Ba006. What is your child's name? (Please start with the eldest, in birth order)
[IWER: Please double check the spelling of the name, if the same name is used in OVERSCREEN. If R does not provide the child's name, describe him/her as 'the first child', 'the second child', etc.]

____

Ba007. Is [child's name] a son or daughter?
① Son
⑤ Daughter

Ba008. How old is [child's name]? (unit: an age)
[IWER:
- For those who respond by a zodiac sign, refer to the index card provided.
- Current age based on the response given in the previous interview(____year____month____day): [child's age] years old]
______years old

Ba009. What is the highest level of education [child's name] has completed?
[IWER: Response in the previous interview(____year____month____day) : [child's level of education and graduation status]]

① No formal education (illiterate) →Go to Ba012

② No formal education(capable of reading) →Go to Ba012

③ Elementary school →Go to Ba011

④ Middle school →Go to Ba011

⑤ High school →Go to Ba011

⑥ Two- year college →Go to Ba011

⑦ College grad →Go to Ba011

⑧ Post college (Master) →Go to Ba011

⑨ Post college (PhD) → Go to Ba011

(97) Others

Ba010. If others, please specify.

[IWER: Response in the previous interview(____year____month____day) : [child's level of education and graduation status]]

____

Ba011. Did he/she graduate, drop out or obtain an equivalent qualification by passing the qualification exam though not completing the school?

[IWER: Response in the previous interview(____year____month____day) : [child's level of education and graduation status]]

① Currently attending

② Graduated

③ Dropped out

④ Completed

⑤ Obtained an equivalent qualification through the qualification exam

Ba012. Does [child's name] work for pay?

[IWER: Response in the previous interview(____year____month____day) : [whether the child works for pay]]

① Yes

⑤ No

Ba013. Is [child's name] currently co-residing with you?

[IWER:

- If the child lives away from home due to study or work, he/she is not co-residing with R.

- Response in the previous interview(____year____month____day) : [co-residing/not co-residing]]

① Yes

⑤ No

Ba014. At the time of the previous interview, you [were /weren't co-residing] with [child's name]. Which months did you live with him/her in the last calendar year (2007)? (Select multiple responses)

① January 2007

② February 2007

③ March 2007

④ April 2007

⑤ May 2007

⑥ June 2007

⑦ July 2007

⑧ August 2007

⑨ September 2007

⑩ October 2007

⑪ November 2007

⑫ December 2007

Ba015. Does [child's name] own a house?

[IWER: Response in the previous interview(____year____month____day) : [whether the child owns a house]]

① Yes

⑤ No

Logic

■ If the child is aged 18 or over, then go to Ba016~Ba017.

Ba016. What is [child's name]'s marital status?

[IWER: Response in the previous interview(____year____month____day) : [child's marital status]]

① Currently married or living with a partner

② Separated

③ Divorced

④ Widowed or missing (Dispersed family)

⑤ Never been married

-9 Don't know

-8 Refuse to answer

Ba017. Does [child's name] have any children? If so, how many?

[IWER: Response in the previous interview(____year____month____day) : [number of children] persons]
______ [range:0~10]

● Distance, frequency of contact, transfer with R's children who do not live together (every children)

Logic

■ If Ba013=⑤, then go to Ba018~Ba020. Each member of the couple is asked these questions.

Ba018. How close does [child's name] live to you?

① Within a 30-minute distance by public transportation

② Within a 1-hour distance by public transportation

③ Within a 2-hour distance by public transportation

④ More than a 2-hour distance by public transportation

Ba019. How often do you meet [child's name] in person?

① Almost every day (more than 4 times per week)

② Once a week

③ 2~3 times a week

④ Once a month

⑤ Twice a month (every two weeks)

⑥ Once or twice a year

⑦ Three or four times a year (once every three or four months)

⑧ Five or six times a year (every two months)

⑨ Almost never a year

⑩ Never

Ba020. How often do you contact [child's name] by phone, mail or e-mail?

① Almost every day (more than 4 times per week)

② Once a week

③ 2~3 times a week

④ Once a month

⑤ Twice a month (every two weeks)

⑥ Once or twice a year

⑦ Three or four times a year (once every three or four months)

⑧ Five or six times a year (every two months

⑨ Almost never a year

⑩ Never

Logic

■ Among the couple, only the representative respondent answerable for CHILDREN section is asked Ba021~Ba067 and Ba068.

■ If R did not live with his/her child for more than one month in 2007, go to Ba021~Ba052.

Ba021. In the last calendar year (2007), did you and your spouse receive from [child's name] any gifts or monetary transfers, such as pocket money, living expenses or medical expenses while living separately? If so, in what form was it received? Please select all that apply. (Select multiple responses.)

* POINT*
- By financial help we mean giving money, helping pay bills, or covering specific types of costs, such as those for medical care or insurance, schooling, down payment for a home, rent, etc., but not counting any shared housing or shared food. The financial help can be considered support, a gift or a loan.
- Regular monetary transfer refers to the case in which you received monetary transfers regularly in a certain time interval (e.g. each month, every two months), such as monthly allowances.
- Occasional monetary transfer refers to the case in which you received monetary transfers without any regularity, such as paying for medical bills or schooling and occasional allowances.
- Monetary transfers include receiving cash as well as bills paid for your behalf. For example, your family member pays for your medical bills, insurance, tuition and so on.
- Non-monetary transfer refers to gifts and goods received.

① Yes, received regular monetary transfers →Go to Ba022

② Yes, received occasional monetary transfers →Go to Ba022 or Ba029

③ Yes, received non-monetary transfers (Ex- gifts, food, clothes, home appliances etc) →Go to Ba028 or Ba029 or Ba035

⑤ No, I did not receive any financial help →Go to Ba037

Logic

■ If Ba021=①, ① ②, ① ③ , ① ② ③, then go to Ba022.

Ba022. [non co-residing]What was the monthly average amount of regular financial pocket money, living expenses, medical expenses etc, you received from [child's name] in the last calendar year (2007)? (10,000 Korean won)

_____MW [range:1~9997] →Go to Ba28

Don't know →Go to Ba023~Ba027 unfolding

Refuse to answer →Go to Ba023~Ba027 unfolding

< Notes on unfolding bracket questions >

- When R says "don't know" or "refuse to answer" to financial questions (i.e., family transfer, health care expenditure, income, and assets, R will be asked "unfolding bracket questions."
- In all unfolding bracket questions, a threshold is presented, and R is asked whether the amount is "less than," "about equal to," or "more than" the threshold.
- There are 5 thresholds for each set of unfolding questions: thresholds 1, 2, 3, 4, and 5, in increasing order.
- R is asked a maximum of 3 questions, presented in the following 3 scenarios.
- R is randomly assigned to 1 of these 3 scenarios.

< Ba023~Ba027. Unfolding bracket questions >

Ba023. Did it amount to less than, about equal to or more than 10 MW (10,000 Korean won)?

[IWER: Go back to Ba022 and record if R answers the amount during the interview.]

① Less than 10 MW

③ About 10 MW

⑤ More than 10 MW

Ba024. Did it amount to less than, about equal to or more than 30 MW (10,000 Korean won)?

[IWER: Go back to Ba022 and record if R answers the amount during the interview.]

① Less than 30 MW

③ About 30 MW

⑤ More than 30 MW

Ba025. Did it amount to less than, about equal to or more than 50 MW (10,000 Korean won)?

[IWER: Go back to Ba022 and record if R answers the amount during the interview.]

① Less than 50 MW

③ About 50 MW

⑤ More than 50 MW

Ba026. Did it amount to less than, about equal to or more than 100 MW (10,000 Korean won)?

[IWER: Go back to Ba022 and record if R answers the amount during the interview.]

① Less than 100 MW

③ About 100 MW

⑤ More than 100 MW

Ba027. Did it amount to less than, about equal to or more than 200 MW (10,000 Korean won)?

[IWER: Go back to Ba022 and record if R answers the amount during the interview.]

① Less than 200 MW

③ About 200 MW

⑤ More than 200 MW

Ba028. Which month(s) did you receive regular monetary transfers from [child's name] in the last calendar year (2007)? Please choose all that apply. (Select multiple responses)

[IWER: "If you alternated between co-residing and not co-residing in 2007, please answer only in relation to the transfers you received while not living together, excluding those you received while living together."]

① January 2007

② February 2007

③ March 2007

④ April 2007

⑤ May 2007

⑥ June 2007

⑦ July 2007

⑧ August 2007

⑨ September 2007

⑩ October 2007

⑪ November 2007

⑫ December 2007

Logic

■ If Ba021=②, ② ①, ② ③, ① ② ③, then go to Ba029.

Ba029. [non co-residing]What was the total amount of occasional monetary transfers you received from [child's name] in the last calendar year (2007)? (unit: 10,000 Korean won)

______MW [range:0~99997] →Go to Ba035

Don't know →Go to Ba030~Ba034 unfolding

Refuse to answer →Go to Ba030~Ba034 unfolding

< Ba030~Ba034. Unfolding bracket questions >

Ba030. Did it amount to less than, about equal to or more than 100 MW (10,000 Korean won)?

[IWER: Go back to Ba029 and record if R answers the amount during the interview.]

① Less than 100 MW

③ About 100 MW

⑤ More than 100 MW

Ba031. Did it amount to less than, about equal to or more than 300 MW (10,000 Korean won)?

[IWER: Go back to Ba029 and record if R answers the amount during the interview.]

① Less than 300 MW

③ About 300 MW

⑤ More than 300 MW

Ba032. Did it amount to less than, about equal to or more than 500 MW (10,000 Korean won)?

[IWER: Go back to Ba029 and record if R answers the amount during the interview.]

① Less than 500 MW

③ About 500 MW

⑤ More than 500 MW

Ba033. Did it amount to less than, about equal to or more than 1,000 MW (10,000 Korean won)?

[IWER: Go back to Ba029 and record if R answers the amount during the interview.]

① Less than 1,000 MW

③ About 1,000 MW

⑤ More than 1,000 MW

Ba034. Did it amount to less than, about equal to or more than 2,000 MW (10,000 Korean won)?

[IWER: Go back to Ba029 and record if R answers the amount during the interview.]

① Less than 2,000 MW

③ About 2,000 MW

⑤ More than 2,000 MW

Logic

■ If Ba021=③, ① ③, ② ③, ① ② ③, then go to Ba035.

Ba035. What was the type of non-monetary transfer you received from [child's name] in the last calendar year (2007)? (Select multiple responses.)

① Leisure (e.g. travel) →Go to Ba037

② Health-related products (e.g. vitamins, equipment, etc.) →Go to Ba037

③ Household items →Go to Ba037

④ Electronics →Go to Ba037

⑤ Dining out and food →Go to Ba037

⑥ Others

Ba036. If others, please specify.

____

Ba037. In the last calendar year (2007), did you and your spouse give [child's name] any gifts or monetary transfers, such as pocket money, living expenses, medical expenses or tuition while living separately? If so, in what form was it given? Please select all that apply. (Select multiple responses.)

① Yes, made regular monetary transfers →Go to Ba038

② Yes, made occasional monetary transfers →Go to Ba038 or Ba045

③ Yes, made non-monetary transfers (Ex- gifts, food, clothes, home appliances etc) →Go to Ba038 or Ba045 or Ba051

⑤ No, I did not give any financial help →Go to Ba053

Logic

■ If Ba037=①, ① ②, ① ③, ① ② ③, then go to Ba038.

Ba038. [non co-residing]What was the monthly average amount of regular monetary transfers you gave to [child's name] in the last calendar year (2007)? (unit: 10,000 Korean won)
[IWER: Monthly average amount of regular monetary transfers: For example if the R supported 1,200,000 won for six months, mark '20MW']

______MW [range:1~9997] →Go to Ba044

Don't know →Go to Ba039~Ba043 unfolding

Refuse to answer →Go to Ba039~Ba043 unfolding

< Ba039~Ba043. Unfolding bracket questions >

Ba039. Did it amount to less than, about equal to or more than 10 MW (10,000 Korean won)?

[IWER: Go back to Ba038 and record if R answers the amount during the interview.]

① Less than 10 MW

③ About 10 MW

⑤ More than 10 MW

Ba040. Did it amount to less than, about equal to or more than 30 MW (10,000 Korean won)?

[IWER: Go back to Ba038 and record if R answers the amount during the interview.]

① Less than 30 MW

③ About 30 MW

⑤ More than 30 MW

Ba041. Did it amount to less than, about equal to or more than 50 MW (10,000 Korean won)?

[IWER: Go back to Ba038 and record if R answers the amount during the interview.]

① Less than 50 MW

③ About 50 MW

⑤ More than 50 MW

Ba042. Did it amount to less than, about equal to or more than 100 MW (10,000 Korean won)?

[IWER: Go back to Ba038 and record if R answers the amount during the interview.]

① Less than 100 MW

③ About 100 MW

⑤ More than 100 MW

Ba043. Did it amount to less than, about equal to or more than 200 MW (10,000 Korean won)?

[IWER: Go back to Ba038 and record if R answers the amount during the interview.]

① Less than 200 MW

③ About 200 MW

⑤ More than 200 MW

Ba044. Which month(s) did you make regular monetary transfers to [child's name] in the last calendar year (2007)? Please choose all that apply. (Select multiple responses)

[IWER: "If you alternated between co-residing and not co-residing in 2007, please answer only in relation to the transfers you provided while not living together, excluding those you provided while living together."]

① January 2007

② February 2007

③ March 2007

④ April 2007

⑤ May 2007

⑥ June 2007

⑦ July 2007

⑧ August 2007

⑨ September 2007

⑩ October 2007

⑪ November 2007

⑫ December 2007

Logic

■ If Ba037= ②, ② ③, ① ②, ① ② ③, then go to Ba045.

Ba045. [non co-residing]What was the total amount of occasional monetary transfers given to [child's name] in the last calendar year (2007)? (unit: 10,000 Korean won)

______MW [range:0~99997] →Go to Ba051
Don't know →Go to Ba046~Ba050 unfolding

Refuses to answer →Go to Ba046~Ba050 unfolding

< Ba046~Ba050. Unfolding bracket questions >

Ba046. Did it amount to less than, about equal to or more than 100 MW (10,000 Korean won)?

[IWER: Go back to Ba045 and record if R answers the amount during the interview.]

① Less than 100 MW

③ About 100 MW

⑤ More than 100 MW

Ba047. Did it amount to less than, about equal to or more than 300 MW (10,000 Korean won)?

[IWER: Go back to Ba045 and record if R answers the amount during the interview.]

① Less than 300 MW

③ About 300 MW

⑤ More than 300 MW

Ba048. Did it amount to less than, about equal to or more than 500 MW (10,000 Korean won)?

[IWER: Go back to Ba045 and record if R answers the amount during the interview.]

① Less than 500 MW

③ About 500 MW

⑤ More than 500 MW

Ba049. Did it amount to less than, about equal to or more than 1,000 MW (10,000 Korean won)?

[IWER: Go back to Ba045 and record if R answers the amount during the interview.]

① Less than 1,000 MW

③ About 1,000 MW

⑤ More than 1,000 MW

Ba050. Did it amount to less than, about equal to or more than 2,000 MW (10,000 Korean won)?
[IWER: Go back to Ba045 and record if R answers the amount during the interview.]
① Less than 2,000 MW
③ About 2,000 MW
⑤ More than 2,000 MW

Logic
■ If Ba037=③, ① ③, ② ③, ① ② ③, then go to Ba051.

Ba051. What was the type of non-monetary transfer you gave to [child's name] in the last calendar year (2007)? (Select multiple responses.)
① Leisure (e.g. travel) →Go to Ba053
② Health-related products (e.g. vitamins, equipment, etc.) →Go to Ba053
③ Household items →Go to Ba053
④ Electronics →Go to Ba053
⑤ Dining out and food →Go to Ba053
⑥ Others

Ba052. If others, please specify.

____

- Transfer with R's children who live together

Logic
■ If R lived with his/her child for more than one month in 2007, then go to Ba053~Ba066.

Ba053. In the last calendar year (2007), did you and your spouse receive any monetary transfers from [child's name] while living together?
[IWER:
- Provision of dwelling space, food, etc., is excluded.
- Monetary transfers include pocket money, medical expenses, travel expenses, business funds, etc.]
① Yes
⑤ No →Go to Ba060

Ba054. [co-residing]If so, what was the total amount of monetary transfers received in the last

calendar year (2007)? (unit: 10,000 Korean won)

______MW [range:0~99997] →Go to Ba060
Don't know →Go to Ba055~Ba059 unfolding

Refuses to answer →Go to Ba055~Ba059 unfolding

< Ba055~Ba059. Unfolding bracket questions >

Ba055. Did it amount to less than, about equal to or more than 100 MW (10,000 Korean won)?

[IWER: Go back to Ba054 and record if R answers the amount during the interview.]

① Less than 100 MW

③ About 100 MW

⑤ More than 100 MW

Ba056. Did it amount to less than, about equal to or more than 300 MW (10,000 Korean won)?

[IWER: Go back to Ba054 and record if R answers the amount during the interview.]

① Less than 300 MW

③ About 300 MW

⑤ More than 300 MW

Ba057. Did it amount to less than, about equal to or more than 500 MW (10,000 Korean won)?

[IWER: Go back to Ba054 and record if R answers the amount during the interview.]

① Less than 500 MW

③ About 500 MW

⑤ More than 500 MW

Ba058. Did it amount to less than, about equal to or more than 1,000 MW (10,000 Korean won)?

[IWER: Go back to Ba054 and record if R answers the amount during the interview.]

① Less than 1,000 MW

③ About 1,000 MW

⑤ More than 1,000 MW

Ba059. Did it amount to less than, about equal to or more than 2,000 MW (10,000 Korean won)?

[IWER: Go back to Ba054 and record if R answers the amount during the interview.]

① Less than 2,000 MW

③ About 2,000 MW

⑤ More than 2,000 MW

Ba060. In the last calendar year (2007), did you and your spouse give any monetary transfers to [child's name] while living together?

[IWER:

- Provision of dwelling space, food, etc., is excluded.

- Monetary transfers include pocket money, medical expenses, travel expenses, business funds, etc.]

① Yes

⑤ No →Go to Ba067

Ba061. [co-residing]If so, what was the total amount of monetary transfers given in the last calendar year (2007)? (unit: 10,000 Korean won)

______MW [range:0~99997] →Go to Ba067
Don't know →Go to Ba062~Ba066 unfolding

Refuses to answer →Go to Ba062~Ba066 unfolding

< Ba062~Ba066. Unfolding bracket questions >

Ba062. Did it amount to less than, about equal to or more than 100 MW (10,000 Korean won)?

[IWER: Go back to Ba061 and record if R answers the amount during the interview.]

① Less than 100 MW

③ About 100 MW

⑤ More than 100 MW

Ba063. Did it amount to less than, about equal to or more than 300 MW (10,000 Korean won)?

[IWER: Go back to Ba061 and record if R answers the amount during the interview.]

① Less than 300 MW

③ About 300 MW

⑤ More than 300 MW

Ba064. Did it amount to less than, about equal to or more than 500 MW (10,000 Korean won)?

[IWER: Go back to Ba061 and record if R answers the amount during the interview.]

① Less than 500 MW

③ About 500 MW

⑤ More than 500 MW

Ba065. Did it amount to less than, about equal to or more than 1,000 MW (10,000 Korean won)?

[IWER: Go back to Ba061 and record if R answers the amount during the interview.]

① Less than 1,000 MW

③ About 1,000 MW

⑤ More than 1,000 MW

Ba066. Did it amount to less than, about equal to or more than 2,000 MW (10,000 Korean won)?
[IWER: Go back to Ba061 and record if R answers the amount during the interview.]
① Less than 2,000 MW
③ About 2,000 MW
⑤ More than 2,000 MW

- Use of money that R provides to R's children

Logic
■ Ba067 is asked with regard to both children who co-resided and those who did not.
■ No data in Ba038, Ba045, Ba061→then go to Ba068

Ba067. You have said that you and your spouse gave [child's name] approximately [transfer amount] MW (10,000 Korean won) in the last calendar year (2007). Among these, how much accounts for educational expenses, wedding expenses, business expenses, medical expenses, and other expenses?
[IWER: The sum of all items must be equal to [(Ba038× Ba044)+Ba045+Ba061] MW.]

ITEMS	AMOUNT(unit: 10,000 Korean won)
Educational expenses	
Wedding expenses	
Business expenses	
Medical expenses	
Other expenses	
Total	[(Ba038× Ba044)+Ba045+Ba061] MW

- Ask about grandchild(ren): Total number of grandchildren(including dead/missing grandchildren), Ask about care labor

Logic
■ Ba068 is a loop value of Ba017. If Ba068<= R's total number of grandchildren reported in Ba017, check for errors.
■ Each member of the couple is asked Ba069~Ba080.

Ba068. Now I'd like to ask you a few questions about grandchildren. Altogether, how many living grandchildren do you have? Please include those whose parent is dead or missing. (unit: number of grandchildren)

_____ [range:0~100] * 0 →Go to Ba081

Logic

■ If Ba068>=1, then go to Ba069.

Ba069. Did you take care of any of those grandchildren before he/she reached the age of 10?

[IWER: Please enter '① Yes' if R took care of a grandchild under the age of 10, even in case the grandchild is now aged 10 or over.]

① Yes

⑤ No →Go to Ba075

Ba070. If so, how many grandchildren did you take care of? [unit: number of grandchildren]

_____ [range:1~10]

[IWER: It is possible to enter a maximum of ten grandchildren R took care of. If the number exceeds 10, R is asked to choose the ten he/she helped most to take care of.]

Logic

■ Loop through Ba071~Ba074 as many times as the number of grandchildren reported in Ba070.

Ba071. What is the name of the grandchild you took care of? (Please start with the eldest, in birth order)

[IWER: If R does not provide the grandchild's name, describe him/her as 'the first grandchild of the first daughter', 'the first grandchild of the second son', etc.]

____

Ba072. Whose child is [grandchild's name in Ba071]?

[IWER: In case of grandchildren whose parent is dead or missing, enter '⑪ Others'.]

①~⑩ Children list (living children)

⑪ Others (missing or dead children)

Ba073. How long did you take care of [grandchild's name in Ba071]? (unit: a year)

_____year [range:0~9]

[IWER:

- If the period of taking care of a grandchild is less than 1 year, enter 0.

- It is possible to enter a maximum of 9 grandchildren because any grandchild taken care of before the age of 10 can be included.]

Ba074. On average how many hours per week did you care for [grandchild's name in Ba071] during that period? (unit: an average hour per week)
[IWER: If R took care of two or more grandchildren at the same time, R is advised to answer separately for each grandchild not to double-count care hours. For example, if R cared for two grandchildren for 10 hours, enter 5 hours for each.]
Average____hours per week [range:1~168]

Ba075. Did you take care of any of your grandchildren under the age of 10 during the past one year?
[IWER: Past one year means a period of one year prior to now, not 2007.]

① Yes
⑤ No →Go to Ba81

Ba076. How many grandchildren did you take care of during the past one year?
[IWER: It is possible to enter a maximum of five grandchildren R took care of. If the number exceeds 5, R is asked to choose the five he/she helped most to take care of.]
_____ [range:1~5]

Logic
■ Loop through Ba077~Ba080 as many times as the number of grandchildren reported in Ba076.

Ba077. What is the name of the grandchild you took care of? (Please start with the eldest, in birth order)
[IWER: If R does not provide the grandchild's name, describe him/her as 'the first grandchild of the first daughter', 'the first grandchild of the second son', etc.]

Ba078. Whose child is [grandchild's name in Ba077]?
[IWER: In case of grandchildren whose parent is dead or missing, enter '⑪ Others'.]
①~⑩ Children list (living children)
⑪ Others (missing or dead children)

Ba079. How long did you take care of [grandchild's name in Ba077] during the past one year?
[IWER: Calculate 1 month as 4 weeks, 6 months as 26 weeks, 1 year as 52 weeks. Past one year means a period of one year prior to now, not 2007.]
_____ weeks [range:1~52]

Ba080. On average how many hours per week did you spend on caring for [grandchild's name in

Ba077] during the past one year? (unit: average number of hours per week)
[IWER:
- Past one year means a period of one year prior to now, not 2007.
- If R took care of two or more grandchildren at the same time, R is advised to answer separately for each grandchild not to double-count care hours. For example, if R cared for two grandchildren for 10 hours, enter 5 hours for each.]
Average____hours per week [range:1~168]

Ba081. [IWER: How often did R receive assistance in answering section Ba-FAMILY AND FAMILY TRANSFER (CHILDREN & GRANDCHILDREN)?]
① Never →Go to section Bb.
② A few times →Go to section Bb.
③ Most or all of the time →Go to section Bb.
④ The section was done by a proxy respondent.

Ba082. What is the proxy's relationship to R? If unknown, please ask the proxy. What is your relationship to R?
① Spouse
② Mother
③ Father
④ Mother-in-law
⑤ Father-in-law
⑥ Brother/sister
⑦ Brother-in-law/sister-in-law
⑧ Son/daughter
⑨ Son-in-law/Daughter-in-law
⑩ Grandchild
⑪ Other relatives
⑫ Helper or other non-relatives
→Go to section Bb.

Bb. FAMILY AND FAMILY TRANSFER
(PARENTS & SIBLINGS)

- Information about R's siblings(survival and living with R)

Logic

■ Loop through Bb001~003 as many times as the number of siblings reported in the previous interview. If R did not have any siblings in the previous interview, then go to Bb004.

Bb001. Now I am going to ask you about your sibling, [sibling's name]. Is [sibling's name] currently alive?

[IWER: This question is intended to find out whether R's sibling has died or not.]

① Yes →Go to Bb003

⑤ No (passed away)

Bb002. When did [sibling's name] pass away?

[IWER: Enter the year and month using 6 digits. For example, mark 200701 for January 2007. If the month is not clear, enter 200700.]

____________________ [range:190000~200812] →Go to Bb004

Bb003. Does [sibling's name] live with you?

① Yes

⑤ No

- Whether R's parents died or not + Basic information.

Logic

■ In the previous interview, R's father and mother were alive: Bb004~Bb015

■ In the previous interview, only R's father was alive: Bb004~Bb009

■ In the previous interview, only R's mother was alive: Bb010~Bb015

■ In the previous interview, R's father and mother died: Bb131

Bb004. Now I have some questions about your father. Is [father's name] currently alive?

[IWER: This question is intended to find out whether R's father has died or not.]

① Yes →Go to Ba006

⑤ No (passed away)

Bb005. When did he die?

[IWER: Enter the year and month using 6 digits. For example, mark 200701 for January 2007. If the month is not clear, enter 200700.]

____________________ →Go to Bb010

Bb006. Does your father work for pay?

① Yes

⑤ No

Bb007. Does your father own a house?

① Yes

⑤ No

Bb008. Does your father live with you?

① Yes

④ No

Bb009. At the time of the previous interview, you [were/weren't co-residing] with your father. Which months did you live with him in the last calendar year (2007)? (Select multiple responses)

① January 2007

② February 2007

③ March 2007

④ April 2007

⑤ May 2007

⑥ June 2007

⑦ July 2007

⑧ August 2007

⑨ September 2007

⑩ October 2007

⑪ November 2007

⑫ December 2007

Bb010. Now I have some questions about your mother. Is [mother's name] currently alive?

[IWER: This question is intended to find out whether R's mother has died or not.]

① Yes →Go to Ba012

⑤ No (passed away)

Bb011. When did she die?

[IWER: Enter the year and month using 6 digits. For example, mark 200701 for January 2007. If the month is not clear, enter 200700.]

____________________ →Go to Bb061

Bb012. Does your mother work for pay?

① Yes

⑤ No

Bb013. Does your mother own a house?

① Yes

⑤ No

Bb014. Does your mother live with you?

① Yes

⑤ No

Bb015. At the time of the previous interview, you [were co-residing/weren't co-residing] with your mother. Which months did you live with her in the last calendar year (2007)? (Select multiple responses)

① January 2007

② February 2007

③ March 2007

④ April 2007

⑤ May 2007

⑥ June 2007

⑦ July 2007

⑧ August 2007

⑨ September 2007

⑩ October 2007

⑪ November 2007

⑫ December 2007

- Contact with R's parents

Logic

- If Bb004=①, Bb010=①, Bb008=⑤ and Bb014=⑤, then go to Bb016~Bb022.
- If (Bb004=①, Bb008=⑤ and Bb010=⑤) or Bb016=⑤, then go to Bb055~Bb060.

- If (Bb004=⑤, Bb010=① and Bb014=⑤) or Bb016=⑤, then go to Bb093~Bb098.
- If Bb004=⑤ and Bb010=⑤, then go to Bb131.

■ If Bb004=①, Bb010=①, Bb008=⑤ and Bb014=⑤, then go to Bb016~Bb022.

Bb016. Do your parents live together?

[IWER: If R's parents are separated or either of them is hospitalized for illness, enter '⑤ No'.]

① Yes

⑤ No → Go to Bb055

Bb017. Do they live by themselves, or with other children (R's sibling)?

① By themselves →Go to Bb020

③ With other children

⑤ Others →Go to Bb019

Bb018. With whom do they live? If they live with multiple children, please identify all co-residents.

27～46 Sibling list →Go to Bb020

Bb019. If others, please specify.

____

Bb020. How close do you live to your parents?

① Within a 30-minute distance by public transportation

② Within a 1-hour distance by public transportation

③ Within a 2-hour distance by public transportation

④ More than a 2-hour distance by public transportation

Bb021. How often do you meet your parents in person?

① Almost every day (more than 4 times per week)

② Once a week

③ 2~3 times a week

④ Once a month

⑤ Twice a month (every two weeks)

⑥ Once or twice a year

⑦ Three or four times a year (once every three or four months)

⑧ Five or six times a year (every two months)

⑨ Almost never a year

⑩ Never

Bb022. How often do you contact your parents by phone, mail, or e-mail?

① Almost every day (more than 4 times per week)

② Once a week

③ 2~3 times a week

④ Once a month

⑤ Twice a month (every two weeks)

⑥ Once or twice a year

⑦ Three or four times a year (once every three or four months)

⑧ Five or six times a year (every two months)

⑨ Almost never a year

⑩ Never

- Transfer to R's parents who did not live with R.

Logic

■ If R did not live with his/her parents for more than one month in 2007, then go to Bb023~Bb054.

Bb023. Not counting any shared housing or shared food, did you receive any financial help from your parents in the last calendar year (2007)? If you did, what kind of help did you receive from your parents? Did you receive regular monetary transfers, occasional monetary transfers, or non-monetary transfers? (Select multiple responses)

* POINT *
- By financial help we mean giving money, helping pay bills, or covering specific types of costs such as those for medical care or insurance, schooling, down payment for a home, rent, etc., but not counting any shared housing or shared food. The financial help can be considered support, a gift or a loan.<br>- Regular monetary transfer refers to the case in which you received monetary transfers regularly in a certain time interval (e.g. each month, every two months), such as monthly allowances.<br>- Occasional monetary transfer refers to the case in which you received monetary transfers without any regularity, such as paying for medical bills or schooling and occasional allowances.<br>- Monetary transfers include receiving cash as well as bills paid for your behalf. For example, your family member pays for your medical bills, insurance, tuition and so on.<br>- Non-monetary transfer refers to gifts and goods received.

① Yes, received regular monetary transfers →Go to Bb024

② Yes, received occasional monetary transfers →Go to Ba024 or Bb031

③ Yes, received non-monetary transfers (Ex- gifts, food, clothes, home appliances etc) →Go to Ba024 or Bb031 or Bb037

⑤ No, I did not receive any financial help →Go to Ba039

Logic

■ If Bb023=①, ① ②, ① ③, ① ② ③, then go to Bb024.

Bb024. [non co-residing]What was the monthly average amount of regular monetary transfers you received from your parents in the last calendar year (2007)? (unit: 10,000 Korean won)
_____MW [range:1~9997] →Go to Bb030

Don't know →Go to Bb025~Bb029 unfolding

Refuse to answer →Go to Bb025~Bb029 unfolding

< Bb025~Bb029. Unfolding bracket questions >

Bb025. Did it amount to less than, about equal to or more than 10 MW (10,000 Korean won) in the last calendar year (2007)?

[IWER: Go back to Bb024 and record if R answers the amount during the interview.]

① Less than 10 MW

③ About 10 MW

⑤ More than 10 MW

Bb026. Did it amount to less than, about equal to or more than 30 MW (10,000 Korean won) in the last calendar year (2007)?

[IWER: Go back to Bb024 and record if R answers the amount during the interview.]

① Less than 30 MW

③ About 30 MW

⑤ More than 30 MW

Bb027. Did it amount to less than, about equal to or more than 50 MW (10,000 Korean won) in the last calendar year (2007)?

[IWER: Go back to Bb024 and record if R answers the amount during the interview.]

① Less than 50 MW

③ About 50 MW

⑤ More than 50 MW

Bb028. Did it amount to less than, about equal to or more than 100 MW (10,000 Korean won) in the last calendar year (2007)?
[IWER: Go back to Bb024 and record if R answers the amount during the interview.]
① Less than 100 MW
③ About 100 MW
⑤ More than 100 MW

Bb029. Did it amount to less than, about equal to or more than 200 MW (10,000 Korean won) in the last calendar year (2007)?
[IWER: Go back to Bb024 and record if R answers the amount during the interview.]
① Less than 200 MW
③ About 200 MW
⑤ More than 200 MW

Bb030. Which months did you receive regular monetary transfers from your parents in the last calendar year (2007)? Please choose all that apply. (Select multiple responses)
[IWER: "If you alternated between co-residing and not co-residing in 2007, please answer only in relation to the transfers you received while not living together, excluding those you received while living together."]
① January 2007
② February 2007
③ March 2007
④ April 2007
⑤ May 2007
⑥ June 2007
⑦ July 2007
⑧ August 2007
⑨ September 2007
⑩ October 2007
⑪ November 2007
⑫ December 2007

Logic
■ If Bb023=②, ② ③, ① ②, ① ② ③, then go to Bb031.

Bb031. [non co-residing]What was the total amount of occasional monetary transfers you received from your parents in the last calendar year (2007)?(unit: 10,000 Korean won)

______MW [range:0~99997] →Go to Bb037
Don't know →Go to Bb032~Bb036 unfolding

Refuse to answer →Go to Bb032~Bb036 unfolding

< Bb032~Bb036. Unfolding bracket questions >

Bb032. Did it amount to less than, about equal to or more than 100 MW (10,000 Korean won) in the last calendar year (2007)?

[IWER: Go back to Bb031 and record if R answers the amount during the interview.]

① Less than 100 MW

③ About 100 MW

⑤ More than 100 MW

Bb033. Did it amount to less than, about equal to or more than 300 MW (10,000 Korean won) in the last calendar year (2007)?

[IWER: Go back to Bb031 and record if R answers the amount during the interview.]

① Less than 300 MW

③ About 300 MW

⑤ More than 300 MW

Bb034. Did it amount to less than, about equal to or more than 500 MW (10,000 Korean won) in the last calendar year (2007)?

[IWER: Go back to Bb031 and record if R answers the amount during the interview.]

① Less than 500 MW

③ About 500 MW

⑤ More than 500 MW

Bb035. Did it amount to less than, about equal to or more than 1,000 MW (10,000 Korean won) in the last calendar year (2007)?

[IWER: Go back to Bb031 and record if R answers the amount during the interview.]

① Less than 1,000 MW

③ About 1,000 MW

⑤ More than 1,000 MW

Bb036. Did it amount to less than, about equal to or more than 2,000 MW (10,000 Korean won) in the last calendar year (2007)?

[IWER: Go back to Bb031 and record if R answers the amount during the interview.]

① Less than 2,000 MW

③ About 2,000 MW

⑤ More than 2,000 MW

Logic

■ If Bb023= ③, ① ③, ② ③, ① ② ③, then go to Bb037.

Bb037. What was the type of non-monetary transfer you received from your parents in the last calendar year (2007)? Please choose all that apply. (Select multiple responses.)

① Leisure (e.g. travel) →Go to Bb039

② Health-related products (e.g. vitamins, equipment, etc.) →Go to Bb039

③ Household items →Go to Bb039

④ Electronics →Go to Bb039

⑤ Dining out and food →Go to Bb039

⑥ Others

Bb038. If others, please specify.

____

Bb039. Not counting any shared housing or shared food, did you give any financial help to your parents in the last calendar year (2007)?

① Yes, made regular monetary transfers →Go to Bb040

② Yes, made occasional monetary transfers →Go to Bb040 or Bb047

③ Yes, made non-monetary transfers (Ex- gifts, food, clothes, home appliances etc) →Go to Bb040 or Bb047 or Bb053

⑤ No, I did not give any financial help →Go to Bb131

Logic

■ If Bb039= ①, ① ②, ① ③, ① ② ③, then go to Bb040.

Bb040. [non co-residing]What was the monthly average amount of regular monetary transfers you gave to your parents in the last calendar year (2007)? (unit: 10,000 Korean won)
_____MW [range:1~9997] →Go to Bb046

Don't know →Go to Bb041~Bb045 unfolding

Refuse to answer →Go to Bb041~Bb045 unfolding

< Bb041~Bb045. Unfolding bracket questions >

Bb041. Did it amount to less than, about equal to or more than 10 MW (10,000 Korean won) in the last calendar year (2007)?

[IWER: Go back to Bb040 and record if R answers the amount during the interview.]

① Less than 10 MW

③ About 10 MW

⑤ More than 10 MW

Bb042. Did it amount to less than, about equal to or more than 30 MW (10,000 Korean won) in the last calendar year (2007)?

[IWER: Go back to Bb040 and record if R answers the amount during the interview.]

① Less than 30 MW

③ About 30 MW

⑤ More than 30 MW

Bb043. Did it amount to less than, about equal to or more than 50 MW (10,000 Korean won) in the last calendar year (2007)?

[IWER: Go back to Bb040 and record if R answers the amount during the interview.]

① Less than 50 MW

③ About 50 MW

⑤ More than 50 MW

Bb044. Did it amount to less than, about equal to or more than 100 MW (10,000 Korean won) in the last calendar year (2007)?

[IWER: Go back to Bb040 and record if R answers the amount during the interview.]

① Less than 100 MW

③ About 100 MW

⑤ More than 100 MW

Bb045. Did it amount to less than, about equal to or more than 200 MW (10,000 Korean won) in the last calendar year (2007)?

[IWER: Go back to Bb040 and record if R answers the amount during the interview.]

① Less than 200 MW

③ About 200 MW

⑤ More than 200 MW

Bb046. Which months did you give regular monetary transfers to your parents in the last calendar year (2007)? Please choose all that apply. (Select multiple responses)

[IWER: “If you alternated between co-residing and not co-residing in 2007, please answer only in relation to the transfers you provided while not living together, excluding those you provided while living together.”]

① January 2007

② February 2007

③ March 2007

④ April 2007

⑤ May 2007

⑥ June 2007

⑦ July 2007

⑧ August 2007

⑨ September 2007

⑩ October 2007

⑪ November 2007

⑫ December 2007

Logic

■ If Bb039=②, ② ③, ① ②, ① ② ③, then go to Bb047.

Bb047. [non co-residing]What was the total amount of occasional monetary transfers given to your parents in the last calendar year (2007)? (unit: 10,000 Korean won)

______MW [range:0~99997] →Go to Bb053
Don’t know →Go to Bb048~Bb052 unfolding

Refuse to answer →Go to Bb048~Bb052 unfolding

< Bb048~Bb052. Unfolding bracket questions >

Bb048. Did it amount to less than, about equal to or more than 100 MW (10,000 Korean won) in the last calendar year (2007)?
[IWER: Go back to Bb047 and record if R answers the amount during the interview.]

① Less than 100 MW

③ About 100 MW

⑤ More than 100 MW

Bb049. Did it amount to less than, about equal to or more than 300 MW (10,000 Korean won) in the last calendar year (2007)?
[IWER: Go back to Bb047 and record if R answers the amount during the interview.]

① Less than 300 MW

③ About 300 MW

⑤ More than 300 MW

Bb050. Did it amount to less than, about equal to or more than 500 MW (10,000 Korean won) in the last calendar year (2007)?
[IWER: Go back to Bb047 and record if R answers the amount during the interview.]

① Less than 500 MW

③ About 500 MW

⑤ More than 500 MW

Bb051. Did it amount to less than, about equal to or more than 1,000 MW (10,000 Korean won) in the last calendar year (2007)?
[IWER: Go back to Bb047 and record if R answers the amount during the interview.]

① Less than 1,000 MW

③ About 1,000 MW

⑤ More than 1,000 MW

Bb052. Did it amount to less than, about equal to or more than 2,000 MW (10,000 Korean won) in the last calendar year (2007)?
[IWER: Go back to Bb047 and record if R answers the amount during the interview.]

① Less than 2,000 MW

③ About 2,000 MW

⑤ More than 2,000 MW

Logic

■ If Bb039=③, ① ③, ② ③, ① ② ③, then go to Bb053.

Bb053. What was the type of non-monetary transfer you gave to your parents in the last calendar year (2007)? (Select multiple responses)

① Leisure (e.g. travel) →Go to Bb178 or Bb131

② Health-related products (e.g. vitamins, equipment, etc) →Go to Bb178 or Bb131

③ Household items →Go to Bb178 or Bb131

④ Electronics →Go to Bb178 or Bb131

⑤ Dining out and food →Go to Bb178 or Bb131

⑥ Others

Bb054. If others, please specify.

____________________→Go to Bb178 or Bb131

- Transfer to R's parents who lived with R.

Logic

■ If R lived with his/her parents for more than one month in 2007, then go to Bb178~Bb191.

Bb178. In the last calendar year (2007), did you receive any monetary transfers from your parents while living together?

[IWER:

- Provision of residential space, food, etc., is excluded.

- Monetary transfers include pocket money, medical expenses, travel expenses, business expenses, etc.]

① Yes

⑤ No →Go to Bb185

Bb179. [co-residing]If so, what was the total amount of monetary transfers you received from your parents in the last calendar year (2007)? (unit: 10,000 Korean won)

____MW [range:0~99997]
Don't know →Go to Bb180~Bb184 unfolding

Refuse to answer →Go to Bb180~Bb184 unfolding

< Bb180~Bb184. Unfolding bracket questions >

Bb180. Did it amount to less than, about equal to or more than 100 MW (10,000 Korean won)?

[IWER: Go back to Bb179 and record if R answers the amount during the interview.]

① Less than 100 MW

③ About 100 MW

⑤ More than 100 MW

Bb181.Did it amount to less than, about equal to or more than 300 MW (10,000 Korean won)?

[IWER: Go back to Bb179 and record if R answers the amount during the interview.]

① Less than 300 MW

③ About 300 MW

⑤ More than 300 MW

Bb182. Did it amount to less than, about equal to or more than 500 MW (10,000 Korean won)?

[IWER: Go back to Bb179 and record if R answers the amount during the interview.]

① Less than 500 MW

③ About 500 MW

⑤ More than 500 MW

Bb183. Did it amount to less than, about equal to or more than 1,000 MW (10,000 Korean won)?
[IWER: Go back to Bb179 and record if R answers the amount during the interview.]
① Less than 1,000 MW
③ About 1,000 MW
⑤ More than 1,000 MW

Bb184. Did it amount to less than, about equal to or more than 2,000 MW (10,000 Korean won)?
[IWER: Go back to Bb179 and record if R answers the amount during the interview.]
① Less than 2,000 MW
③ About 2,000 MW
⑤ More than 2,000 MW

Bb185. In the last calendar year (2007), did you give any monetary transfers to your parents while living together?
[IWER:
- Provision of dwelling space, food, etc., is excluded.
- Monetary transfers include pocket money, medical expenses, travel expenses, business funds, etc.]
① Yes
⑤ No →Go to Bb131

Bb186. [co-residing]If so, what was the total amount of monetary transfers you gave to your parents in the last calendar year (2007)? (unit: 10,000 Korean won)

______MW [range:0~99997] →Go to Bb131
Don't know →Go to Bb187~Bb191 unfolding
Refuse to answer →Go to Bb187~Bb191 unfolding

< Bb187~Bb191. Unfolding bracket questions >
Bb187. Did it amount to less than, about equal to or more than 100 MW (10,000 Korean won)?
[IWER: Go back to Bb186 and record if R answers the amount during the interview.]
① Less than 100 MW
③ About 100 MW
⑤ More than 100 MW

Bb188.Did it amount to less than, about equal to or more than 300 MW (10,000 Korean won)?
[IWER: Go back to Bb186 and record if R answers the amount during the interview.]
① Less than 300 MW

③ About 300 MW

⑤ More than 300 MW

Bb189. Did it amount to less than, about equal to or more than 500 MW (10,000 Korean won)?

[IWER: Go back to Bb186 and record if R answers the amount during the interview.]

① Less than 500 MW

③ About 500 MW

⑤ More than 500 MW

Bb190. Did it amount to less than, about equal to or more than 1,000 MW (10,000 Korean won)?

[IWER: Go back to Bb186 and record if R answers the amount during the interview.]

① Less than 1,000 MW

③ About 1,000 MW

⑤ More than 1,000 MW

Bb191. Did it amount to less than, about equal to or more than 2,000 MW (10,000 Korean won)?

[IWER: Go back to Bb186 and record if R answers the amount during the interview.]

① Less than 2,000 MW

③ About 2,000 MW

⑤ More than 2,000 MW

- Contact with R's father.

Logic

■ If (Bb004=①, Bb008=⑤ and Bb010=⑤) or Bb016=⑤, then go to Bb055~Bb060.

Bb055. Does your father live by himself or with other children [R's sibling]?

① By himself →Go to Bb058

③ With other children

⑤ Others →Go to Bb057

Bb056. With whom does he live? Please list all, if he lives with multiple children.

[IWER: Choose all that apply.]

27~46 Sibling list →Go to Bb058

Bb057. If others, please specify.

Bb058. How close do you live to your father?

① Within a 30-minute distance by public transportation

② Within a 1-hour distance by public transportation

③ Within a 2-hour distance by public transportation

④ More than a 2-hour distance by public transportation

Bb059. How often do you meet your father in person?

① Almost every day (more than 4 times per week)

② Once a week

③ 2~3 times a week

④ Once a month

⑤ Twice a month (every two weeks)

⑥ Once or twice a year

⑦ Three or four times a year (once every three or four months)

⑧ Five or six times a year (every two months)

⑨ Almost never a year

⑩ Never

Bb060. How often do you contact your father by phone, mail, or e-mail?

① Almost every day (more than 4 times per week)

② Once a week

③ 2~3 times a week

④ Once a month

⑤ Twice a month (every two weeks)

⑥ Once or twice a year

⑦ Three or four times a year (once every three or four months)

⑧ Five or six times a year (every two months)

⑨ Almost never a year

⑩ Never

- Transfer to R's father.

Logic

■ If R did not live with his/her father for more than one month in 2007, then go to Bb061~Bb092.

Bb061. Not counting any shared housing or shared food, did you receive any financial help from your father in the last calendar year (2007)? If you did, what kind of help did you receive from your father? Did you receive regular monetary transfers, occasional monetary transfers, or non-monetary transfers?

Please choose all that apply.

* POINT *
- By financial help we mean giving money, helping pay bills, or covering specific types of costs, such as those for medical care or insurance, schooling, down payment for a home, rent, etc., but not counting any shared housing or shared food. The financial help can be considered support, a gift or a loan.
- Regular monetary transfer refers to the case in which you received monetary transfers regularly in a certain time interval (e.g. each month, every two months), such as monthly allowances.
- Occasional monetary transfer refers to the case in which you received monetary transfers without any regularity, such as paying for medical bills or schooling and occasional allowances.
- Monetary transfers include receiving cash as well as bills paid for your behalf. For example, your family member pays for your medical bills, insurance, tuition and so on.
- Non-monetary transfer refers to gifts and goods received

① Yes, received regular monetary transfers →Go to Bb062

② Yes, received occasional monetary transfers →Go to Bb062 or Bb069

③ Yes, received non-monetary transfers (Ex- gifts, food, clothes, home appliances etc) →Go to Bb062 or Bb069 or Bb075

⑤ No, I did not receive any financial help →Go to Bb077

Logic

■ If Bb061=①, ① ②, ① ③, ① ② ③, then go to Bb062.

Bb062. [non co-residing]What was the monthly average amount of regular monetary transfers you received from your father in the last calendar year (2007)? (unit: 10,000 Korean won)

_____MW [range:1~9997] →Go to Bb068

Don't know →Go to Bb063~Bb067 unfolding

Refuse to answer →Go to Bb063~Bb067 unfolding

< Bb063~Bb067. Unfolding bracket questions >

Bb063. Did it amount to less than, about equal to or more than 10 MW (10,000 Korean won) in the last calendar year (2007)?
[IWER: Go back to Bb062 and record if R answers the amount during the interview.]

① Less than 10 MW

③ About 10 MW

⑤ More than 10 MW

Bb064. Did it amount to less than, about equal to or more than 30 MW (10,000 Korean won) in the last calendar year (2007)?
[IWER: Go back to Bb062 and record if R answers the amount during the interview.]

① Less than 30 MW

③ About 30 MW

⑤ More than 30 MW

Bb065. Did it amount to less than, about equal to or more than 50 MW (10,000 Korean won) in the last calendar year (2007)?
[IWER: Go back to Bb062 and record if R answers the amount during the interview.]

① Less than 50 MW

③ About 50 MW

⑤ More than 50 MW

Bb066. Did it amount to less than, about equal to or more than 100 MW (10,000 Korean won) in the last calendar year (2007)?
[IWER: Go back to Bb062 and record if R answers the amount during the interview.]

① Less than 100 MW

③ About 100 MW

⑤ More than 100 MW

Bb067. Did it amount to less than, about equal to or more than 200 MW (10,000 Korean won) in the last calendar year (2007)?
[IWER: Go back to Bb062 and record if R answers the amount during the interview.]

① Less than 200 MW

③ About 200 MW

⑤ More than 200 MW

Bb068. Which months did you receive regular monetary transfers from your father in the last calendar year (2007)? Please choose all that apply. (Select multiple responses)

[IWER: "If you alternated between co-residing and not co-residing in 2007, please answer only in relation to the transfers you received while not living together, excluding those you received while living together."]

① January 2007

② February 2007

③ March 2007

④ April 2007

⑤ May 2007

⑥ June 2007

⑦ July 2007

⑧ August 2007

⑨ September 2007

⑩ October 2007

⑪ November 2007

⑫ December 2007

Logic

■ If Bb061=②, ② ③, ① ②, ① ② ③, then go to Bb069.

Bb069. What was the total amount of occasional monetary transfers you received from your father in the last calendar year (2007)? (unit: 10,000 Korean won)

______MW [range:0~99997]→Go to Bb075
Don't know →Go to Bb070~Bb074 unfolding

Reuse to answer →Go to Bb070~Bb074 unfolding

< Bb070~Bb074. Unfolding bracket questions >

Bb070. Did it amount to less than, about equal to or more than 100 MW (10,000 Korean won) in the last calendar year (2007)?
[IWER: Go back to Bb069 and record if R answers the amount during the interview.]

① Less than 100 MW

③ About 100 MW

⑤ More than 100 MW

Bb071. Did it amount to less than, about equal to or more than 300 MW (10,000 Korean won) in the last calendar year (2007)?
[IWER: Go back to Bb069 and record if R answers the amount during the interview.]

① Less than 300 MW

③ About 300 MW

⑤ More than 300 MW

Bb072. Did it amount to less than, about equal to or more than 500 MW (10,000 Korean won) in the last calendar year (2007)?
[IWER: Go back to Bb069 and record if R answers the amount during the interview.]

① Less than 500 MW

③ About 500 MW

⑤ More than 500 MW

Bb073. Did it amount to less than, about equal to or more than 1,000 MW (10,000 Korean won) in the last calendar year (2007)?
[IWER: Go back to Bb069 and record if R answers the amount during the interview.]

① Less than 1,000 MW

③ About 1,000 MW

⑤ More than 1,000 MW

Bb074. Did it amount to less than, about equal to or more than 2,000 MW (10,000 Korean won) in the last calendar year (2007)?
[IWER: Go back to Bb069 and record if R answers the amount during the interview.]

① Less than 2,000 MW

③ About 2,000 MW

⑤ More than 2,000 MW

Logic

■ If Bb061=③, ① ③, ② ③, ① ② ③, then go to Bb075.

Bb075. What was the type of non-monetary transfer you received from your father in the last calendar year (2007)? (Select multiple responses)
① Leisure (e.g. travel) →Go to Bb077

② Health-related products (e.g. vitamins, equipment, etc) →Go to Bb077

③ Household items →Go to Bb077

④ Electronics →Go to Bb077

⑤ Dining out and food →Go to Bb077

⑥ Others

Bb076. If others, please specify.

____

Bb077. Not counting any shared housing or shared food, did you give any financial help to your father in the last calendar year (2007)? (Select multiple responses)

① Yes, made regular monetary transfers →Go to Bb078

② Yes, made occasional monetary transfers →Go to Bb078 or Bb085

③ Yes, made non-monetary transfers (Ex- gifts, food, clothes, home appliances etc) →Go to Bb078 or Bb085 or Bb091

⑤ No, I did not give any financial help →Go to Bb093

.

Logic

■ If Bb077=①, ① ②, ① ③, ① ② ③, then go to Bb078

Bb078. [non co-residing]What was the monthly average amount of regular monetary transfers you gave to your father in the last calendar year (2007)? (unit: 10,000 Korean won)
_____MW [range:1~9997] →Go to Bb084

Don't know →Go to Bb079~Bb083 unfolding

Refuse to answer →Go to Bb079~Bb083 unfolding

< Bb079~Bb083. Unfolding bracket questions >

Bb079. Did it amount to less than, about equal to or more than 10 MW (10,000 Korean won) in the last calendar year (2007)?
[IWER: Go back to Bb078 and record if R answers the amount during the interview.]

① Less than 10 MW

③ About 10 MW

⑤ More than 10 MW

Bb080. Did it amount to less than, about equal to or more than 30 MW (10,000 Korean won) in the last calendar year (2007)?
[IWER: Go back to Bb078 and record if R answers the amount during the interview.]

① Less than 30 MW

③ About 30 MW

⑤ More than 30 MW

Bb081. Did it amount to less than, about equal to or more than 50 MW (10,000 Korean won) in the last calendar year (2007)?
[IWER: Go back to Bb078 and record if R answers the amount during the interview.]

① Less than 50 MW

③ About 50 MW

⑤ More than 50 MW

Bb082. Did it amount to less than, about equal to or more than 100 MW (10,000 Korean won) in the last calendar year (2007)?
[IWER: Go back to Bb078 and record if R answers the amount during the interview.]

① Less than 100 MW

③ About 100 MW

⑤ More than 100 MW

Bb083. Did it amount to less than, about equal to or more than 200 MW (10,000 Korean won) in the last calendar year (2007)?
[IWER: Go back to Bb078 and record if R answers the amount during the interview.]

① Less than 200 MW

③ About 200 MW

⑤ More than 200 MW

Bb084. Which months did you give regular monetary transfers to your father in the last calendar year (2007)? Please choose all that apply. (Select multiple responses)
[IWER: "If you alternated between co-residing and not co-residing in 2007, please answer only in relation to the transfers you provided while not living together, excluding those you provided while living together."]

① January 2007

② February 2007

③ March 2007

④ April 2007

⑤ May 2007

⑥ June 2007

⑦ July 2007

⑧ August 2007

⑨ September 2007

⑩ October 2007

⑪ November 2007

⑫ December 2007

Logic

■ If Bb077=②, ② ③, ① ②, ① ② ③, then go to Bb085.

Bb085. [non co-residing]What was the total amount of occasional monetary transfers given to your father in the last calendar year (2007)? (unit: 10,000 Korean won)

______MW [range:0~99997] →Go to Bb091
Don't know →Go to Bb086~Bb090 unfolding

Refuse to answer →Go to Bb086~Bb090 unfolding

< Bb086~Bb090. Unfolding bracket questions >

Bb086. Did it amount to less than, about equal to or more than 100 MW (10,000 Korean won) in the last calendar year (2007)?
[IWER: Go back to Bb085 and record if R answers the amount during the interview.]

① Less than 100 MW

③ About 100 MW

⑤ More than 100 MW

Bb087. Did it amount to less than, about equal to or more than 300 MW (10,000 Korean won) in the last calendar year (2007)?
[IWER: Go back to Bb085 and record if R answers the amount during the interview.]

① Less than 300 MW

③ About 300 MW

⑤ More than 300 MW

Bb088. Did it amount to less than, about equal to or more than 500 MW (10,000 Korean won) in the last calendar year (2007)?
[IWER: Go back to Bb085 and record if R answers the amount during the interview.]

① Less than 500 MW

③ About 500 MW

⑤ More than 500 MW

Bb089. Did it amount to less than, about equal to or more than 1,000 MW (10,000 Korean won) in the last calendar year (2007)?
[IWER: Go back to Bb085 and record if R answers the amount during the interview.]

① Less than 1,000 MW

③ About 1,000 MW

⑤ More than 1,000 MW

Bb090. Did it amount to less than, about equal to or more than 2,000 MW (10,000 Korean won) in the last calendar year (2007)?
[IWER: Go back to Bb085 and record if R answers the amount during the interview.]

① Less than 2,000 MW

③ About 2,000 MW

⑤ More than 2,000 MW

Logic

■ If Bb077=③, ① ③, ② ③, ① ② ③, then go to Bb091.

Bb091. What was the type of non-monetary transfer you gave to your father in the last calendar year (2007)? Please choose all that apply. (Select multiple responses)

① Leisure (e.g. travel) →Go to Bb192 or Bb093

② Health-related products (e.g. vitamins, equipment, etc) →Go to Bb192 or Bb093

③ Household items →Go to Bb192 or Bb093

④ Electronics →Go to Bb192 or Bb093

⑤ Dining out and food →Go to Bb192 or Bb093

⑥ Others

Bb092. If others, please specify.

__________________ →Go to Bb192 or Bb093

- Transfer to R's father who lived with R.

Logic

■ If R lived with his/her father for more than one month in 2007, then go to Bb192~Bb205.

Bb192. In the last calendar year (2007), did you receive any monetary transfers from your father while living together?

[IWER:

- Provision of dwelling space, food, etc., is excluded.

- Monetary transfers include pocket money, medical expenses, travel expenses, business expenses, etc.]

① Yes

⑤ No →Go to Bb199

Bb193. [co-residing]If so, what was the total amount of monetary transfers you received from your father in the last calendar year (2007)? (unit: 10,000 Korean won)

______MW [range:0~99997] →Go to Bb199

Don't know →GO to Bb194~Bb198 unfolding

Refuse to answer →GO to Bb194~Bb198 unfolding

< Bb194~Bb198. Unfolding bracket questions >

Bb194. Did it amount to less than, about equal to or more than 100 MW (10,000 Korean won) in the last calendar year (2007)?

[IWER: Go back to Bb193 and record if R answers the amount during the interview.]

① Less than 100 MW

③ About 100 MW

⑤ More than 100 MW

Bb195. Did it amount to less than, about equal to or more than 300 MW (10,000 Korean won) in the last calendar year (2007)?

[IWER: Go back to Bb193 and record if R answers the amount during the interview.]

① Less than 300 MW

③ About 300 MW

⑤ More than 300 MW

Bb196. Did it amount to less than, about equal to or more than 500 MW (10,000 Korean won) in the last calendar year (2007)?

[IWER: Go back to Bb193 and record if R answers the amount during the interview.]

① Less than 500 MW

③ About 500 MW

⑤ More than 500 MW

Bb197. Did it amount to less than, about equal to or more than 1,000 MW (10,000 Korean won) in the last calendar year (2007)?

[IWER: Go back to Bb193 and record if R answers the amount during the interview.]

① Less than 1,000 MW

③ About 1,000 MW

⑤ More than 1,000 MW

Bb198. Did it amount to less than, about equal to or more than 2,000 MW (10,000 Korean won) in the last calendar year (2007)?

[IWER: Go back to Bb193 and record if R answers the amount during the interview.]

① Less than 2,000 MW

③ About 2,000 MW

⑤ More than 2,000 MW

Bb199. In the last calendar year (2007), did you give any monetary transfers to your father while living together?

[IWER:

- Provision of dwelling space, food, etc., is excluded.

- Monetary transfers include pocket money, medical expenses, travel expenses, business expenses, etc.]

① Yes

⑤ No →Go to Bb131

Bb200. [co-residing]If so, what was the total amount of monetary transfers you gave to your father in the last calendar year (2007)? (unit: 10,000 Korean won)

_

______MW [range:0~99997] →Go to Bb131
Don't know →Go to Bb201~Bb205 unfolding

Refuse to answer →Go to Bb201~Bb205 unfolding

< Bb201~Bb205. Unfolding bracket questions >

Bb201. Did it amount to less than, about equal to or more than 100 MW (10,000 Korean won)?

[IWER: Go back to Bb200 and record if R answers the amount during the interview.]

① Less than 100 MW

③ About 100 MW

⑤ More than 100 MW

Bb202. Did it amount to less than, about equal to or more than 300 MW (10,000 Korean won)?

[IWER: Go back to Bb200 and record if R answers the amount during the interview.]

① Less than 300 MW

③ About 300 MW

⑤ More than 300 MW

Bb203. Did it amount to less than, about equal to or more than 500 MW (10,000 Korean won)?

[IWER: Go back to Bb200 and record if R answers the amount during the interview.]

① Less than 500 MW

③ About 500 MW

⑤ More than 500 MW

Bb204. Did it amount to less than, about equal to or more than 1,000 MW (10,000 Korean won)?

[IWER: Go back to Bb200 and record if R answers the amount during the interview.]

① Less than 1,000 MW

③ About 1,000 MW

⑤ More than 1,000 MW

Bb205. Did it amount to less than, about equal to or more than 2,000 MW (10,000 Korean won)?

[IWER: Go back to Bb200 and record if R answers the amount during the interview.]

① Less than 2,000 MW

④ About 2,000 MW

⑤ More than 2,000 MW

- Contact with R's mother.

Logic

■ If (Bb004=⑤, Bb010=①, and Bb014=⑤) or Bb016=⑤, then go to Bb093~Bb098.

Bb093. Does your mother live by herself or with other children [R’s sibling]?

① By herself →Go to Bb096

③ With other children

⑤ Others →Go to Bb095

Bb094. With whom does she live? Please list all, if she lives with multiple children.

[IWER: Choose all that apply]

27～46 Sibling list →Go to Bb096

Bb095. If others, please specify.

____

Bb096. How close do you live to your mother?

① Within a 30-minute distance by public transportation

② Within a 1-hour distance by public transportation

③ Within a 2-hour distance by public transportation

④ More than a 2-hour distance by public transportation

Bb097. How often do you meet your mother in person?

① Almost every day (more than 4 times per week)

② Once a week

③ 2~3 times a week

④ Once a month

⑤ Twice a month (every two weeks)

⑥ Once or twice a year

⑦ Three or four times a year (once every three or four months)

⑧ Five or six times a year (every two months)

⑨ Almost never a year

⑩ Never

Bb098. How often do you contact your mother by phone, mail, or e-mail?

① Almost every day (more than 4 times per week)

② Once a week

③ 2~3 times a week

④ Once a month

⑤ Twice a month (every two weeks)

⑥ Once or twice a year

⑦ Three or four times a year (once every three or four months)

⑧ Five or six times a year (every two months)

⑨ Almost never a year

⑩ Never

● Transfer to R's mother.

Logic

■ **If R did not live with his/her mother for more than one month in 2007, then go to Bb099~Bb130.**

Bb099. Not counting any shared housing or shared food, did you receive any financial help from your mother in the last calendar year (2007)? If you did, what kind of help did you receive from your mother? Did you receive regular monetary transfers, occasional monetary transfers, or non-monetary transfers? Please choose all that apply. (Select multiple responses)

* POINT *
- By financial help we mean giving money, helping pay bills, or covering specific types of costs such as those for medical care or insurance, schooling, down payment for a home, rent, etc., but not counting any shared housing or shared food. The financial help can be considered support, a gift or a loan.<br>- Regular monetary transfer refers to the case in which you received monetary transfers regularly in a certain time interval (e.g. each month, every two months), such as monthly allowances.<br>- Occasional monetary transfer refers to the case in which you received monetary transfers without any regularity, such as paying for medical bills or schooling and occasional allowances.<br>- Monetary transfers include receiving cash as well as bills paid for your behalf. For example, your family member pays for your medical bills, insurance, tuition and so on.<br>- Non-monetary transfer refers to gifts and goods received

① Yes, received regular monetary transfers →Go to Bb100

② Yes, received occasional monetary transfers →Go to Bb100 or Bb107

③ Yes, received non-monetary transfers (Ex- gifts, food, clothes, home appliances etc) →Go to Bb100 or Bb107 or Bb113

⑤ No, I did not receive any financial help →Go to Bb115

Logic

■ If Bb099=①, ① ②, ① ③, ① ② ③, then go to Bb100.

Bb100. [non co-residing]What was the monthly average amount of regular monetary transfers you received from your mother in the last calendar year (2007)? (unit: 10,000 Korean won)
_____MW [range:1~9997] →Go to Bb106

Don't know →Bb101~Bb105 unfolding

Refuse to answer →Bb101~Bb105 unfolding

< Bb101~Bb105. Unfolding bracket questions >

Bb101. Did it amount to less than, about equal to or more than 10 MW (10,000 Korean won) in the last calendar year (2007)?
[IWER: Go back to Bb100 and record if R answers the amount during the interview.]

① Less than 10 MW

③ About 10 MW

⑤ More than 10 MW

Bb102. Did it amount to less than, about equal to or more than 30 MW (10,000 Korean won) in the last calendar year (2007)?
[IWER: Go back to Bb100 and record if R answers the amount during the interview.]

① Less than 30 MW

③ About 30 MW

⑤ More than 30 MW

Bb103. Did it amount to less than, about equal to or more than 50 MW (10,000 Korean won) in the last calendar year (2007)?
[IWER: Go back to Bb100 and record if R answers the amount during the interview.]

① Less than 50 MW

③ About 50 MW

⑤ More than 50 MW

Bb104. Did it amount to less than, about equal to or more than 100 MW (10,000 Korean won) in the last calendar year (2007)?
[IWER: Go back to Bb100 and record if R answers the amount during the interview.]

① Less than 100 MW

③ About 100 MW

⑤ More than 100 MW

Bb105. Did it amount to less than, about equal to or more than 200 MW (10,000 Korean won) in the last calendar year (2007)?

[IWER: Go back to Bb100 and record if R answers the amount during the interview.]

① Less than 200 MW

③ About 200 MW

⑤ More than 200 MW

Bb106. Which months did you receive regular monetary transfers from your mother in the last calendar year (2007)? Please choose all that apply. (Select multiple responses)

[IWER: “If you alternated between co-residing and not co-residing in 2007, please answer only in relation to the transfers you received while not living together, excluding those you received while living together.”]

① January 2007

② February 2007

③ March 2007

④ April 2007

⑤ May 2007

⑥ June 2007

⑦ July 2007

⑧ August 2007

⑨ September 2007

⑩ October 2007

⑪ November 2007

⑫ December 2007

Logic

■ If Bb099=②, ② ③, ① ②, ① ② ③, then go to Bb107.

Bb107. What was the total amount of occasional monetary transfers you received from your mother in the last calendar year (2007)? (unit: 10,000 Korean won)

______MW [range:0~99997] →Go to Bb113

Don’t know →Bb108~Bb112 unfolding

Refuse to answer →Bb108~Bb112 unfolding

< Bb108~Bb112. Unfolding bracket questions >

Bb108. Did it amount to less than, about equal to or more than 100 MW (10,000 Korean won) in the last calendar year (2007)?

[IWER: Go back to Bb107 and record if R answers the amount during the interview.]

① Less than 100 MW

③ About 100 MW

⑤ More than 100 MW

Bb109. Did it amount to less than, about equal to or more than 300 MW (10,000 Korean won) in the last calendar year (2007)?
[IWER: Go back to Bb107 and record if R answers the amount during the interview.]

① Less than 300 MW

③ About 300 MW

⑤ More than 300 MW

Bb110. Did it amount to less than, about equal to or more than 500 MW (10,000 Korean won) in the last calendar year (2007)?
[IWER: Go back to Bb107 and record if R answers the amount during the interview.]

① Less than 500 MW

③ About 500 MW

⑤ More than 500 MW

Bb111. Did it amount to less than, about equal to or more than 1,000 MW (10,000 Korean won) in the last calendar year (2007)?
[IWER: Go back to Bb107 and record if R answers the amount during the interview.]

① Less than 1,000 MW

③ About 1,000 MW

⑤ More than 1,000 MW

Bb112. Did it amount to less than, about equal to or more than 2,000 MW (10,000 Korean won) in the last calendar year (2007)?
[IWER: Go back to Bb107 and record if R answers the amount during the interview.]

① Less than 2,000 MW

③ About 2,000 MW

⑤ More than 2,000 MW

Logic

■ If Bb099=③, ① ③, ② ③, ① ② ③, then go to Bb113.

Bb113. What was the type of non-monetary transfer you received from your parents in the last calendar year (2007)? Please check all that apply.

① Leisure (e.g. travel) →Go to Bb115

② Health-related products (e.g. vitamins, equipment, etc) →Go to Bb115

③ Household items →Go to Bb115

④ Electronics →Go to Bb115

⑤ Dining out and food →Go to Bb115

⑥ Others

Bb114. If others, please specify.

____

Bb115. Not counting any shared housing or shared food, did you give any financial help to your mother in the last calendar year (2007)?

① Yes, made regular monetary transfers →Go to Bb116

② Yes, made occasional monetary transfers →Go to Bb116 or Bb123

③ Yes, made non-monetary transfers (Ex- gifts, food, clothes, home appliances etc) →Go to Bb116 or Bb123 or Bb129

⑤ No, I did not give any financial help →Go to Bb131

Logic

■ If Bb115=①, ① ②, ① ③, ① ② ③, then go to Bb116.

Bb116. [non co-residing]What was the monthly average amount of regular monetary transfers you gave to your mother in the last calendar year (2007)? (unit: 10,000 Korean won)
_____MW [range:1~9997] →Go to Bb122

Don't know →Go to Bb117~Bb121 unfolding

Refuse to answer →Go to Bb117~Bb121 unfolding

< Bb117~Bb121. Unfolding bracket questions >

Bb117. Did it amount to less than, about equal to or more than 10 MW (10,000 Korean won) in the last calendar year (2007)?
[IWER: Go back to Bb116 and record if R answers the amount during the interview.]

① Less than 10 MW

③ About 10 MW

⑤ More than 10 MW

Bb118. Did it amount to less than, about equal to or more than 30 MW (10,000 Korean won) in the last calendar year (2007)?
[IWER: Go back to Bb116 and record if R answers the amount during the interview.]

① Less than 30 MW

③ About 30 MW

⑤ More than 30 MW

Bb119. Did it amount to less than, about equal to or more than 50 MW (10,000 Korean won) in the last calendar year (2007)?
[IWER: Go back to Bb116 and record if R answers the amount during the interview.]

① Less than 50 MW

③ About 50 MW

⑤ More than 50 MW

Bb120. Did it amount to less than, about equal to or more than 100 MW (10,000 Korean won) in the last calendar year (2007)?
[IWER: Go back to Bb116 and record if R answers the amount during the interview.]

① Less than 100 MW

③ About 100 MW

⑤ More than 100 MW

Bb121. Did it amount to less than, about equal to or more than 200 MW (10,000 Korean won) in the last calendar year (2007)?
[IWER: Go back to Bb116 and record if R answers the amount during the interview.]

① Less than 200 MW

③ About 200 MW

⑤ More than 200 MW

Bb122. Which months did you give regular monetary transfers to your mother in the last calendar year (2007)? Please check all that apply. (Select multiple responses)
[IWER: "If you alternated between co-residing and not co-residing in 2007, please answer only in relation to the transfers you provided while not living together, excluding those you provided while living together."]

① January 2007

② February 2007

③ March 2007

④ April 2007

⑤ May 2007

⑥ June 2007

⑦ July 2007

⑧ August 2007

⑨ September 2007

⑩ October 2007

⑪ November 2007

⑫ December 2007

Logic

■ If Bb115=②, ② ③, ① ②, ① ② ③, then go to Bb123.

Bb123. [non co-residing]What was the total amount of occasional monetary transfers you gave to your mother in the last calendar year (2007)? (unit: 10,000 Korean won)

______MW [range:0~99997] →Go to Bb129
Don't know → Go to Bb124~Bb128 unfolding

Refuse to answer → Go to Bb124~Bb128 unfolding

< Bb124~Bb128. Unfolding bracket questions >

Bb124. Did it amount to less than, about equal to or more than 100 MW (10,000 Korean won) in the last calendar year (2007)?
[IWER: Go back to Bb123 and record if R answers the amount during the interview.]

① Less than 100 MW

③ About 100 MW

⑤ More than 100 MW

Bb125. Did it amount to less than, about equal to or more than 300 MW (10,000 Korean won) in the last calendar year (2007)?
[IWER: Go back to Bb123 and record if R answers the amount during the interview.]

① Less than 300 MW

③ About 300 MW

⑤ More than 300 MW

Bb126. Did it amount to less than, about equal to or more than 500 MW (10,000 Korean won) in the last calendar year (2007)?
[IWER: Go back to Bb123 and record if R answers the amount during the interview.]

① Less than 500 MW

③ About 500 MW

⑤ More than 500 MW

Bb127. Did it amount to less than, about equal to or more than 1,000 MW (10,000 Korean won) in the last calendar year (2007)?
[IWER: Go back to Bb123 and record if R answers the amount during the interview.]

① Less than 1,000 MW

③ About 1,000 MW

⑤ More than 1,000 MW

Bb128. Did it amount to less than, about equal to or more than 2,000 MW (10,000 Korean won) in the last calendar year (2007)?
[IWER: Go back to Bb123 and record if R answers the amount during the interview.]

① Less than 2,000 MW

③ About 2,000 MW

⑤ More than 2,000 MW

Logic

■ If Bb115=③, ① ③, ② ③, ① ② ③, then go to Bb129.

Bb129. What was the type of non-monetary transfer you gave to your mother in the last calendar year (2007)? Please choose all that apply. (Select multiple responses.)

① Leisure (e.g. travel) →Go to Bb206 or Bb131

② Health-related products (e.g. vitamins, equipment, etc) →Go to Bb206 or Bb131

③ Household items →Go to Bb206 or Bb131

④ Electronics →Go to Bb206 or Bb131

⑤ Dining out and food →Go to Bb206 or Bb131

⑥ Others

Bb130. If others, please specify.

__________________ →Go to Bb206 or Bb131

- Transfer to R's mother who lived with R.

Logic

■ If R lived with his/her mother for more than one month in 2007, then go to Bb206~Bb219.

Bb206. In the last calendar year (2007), did you receive any monetary transfers from your mother while living together?

[IWER:

- Provision of dwelling space, food, etc., is excluded.

- Monetary transfers include pocket money, medical expenses, travel expenses, business expenses, etc.]

① Yes

⑤ No → Go to Bb213

Bb207. [co-residing]If so, what was the total amount of monetary transfers you received from your mother in the last calendar year (2007)? (unit: 10,000 Korean won)

______MW [range:0~99997]→Go to Bb213
Don't know → Bb208~Bb212 unfolding

Refuse to answer → Bb208~Bb212 unfolding

< Bb208~Bb212. Unfolding bracket questions >

Bb208. Did it amount to less than, about equal to or more than 100 MW (10,000 Korean won)?

[IWER: Go back to Bb207 and record if R answers the amount during the interview.]

① Less than 100 MW

③ About 100 MW

⑤ More than 100 MW

Bb209. Did it amount to less than, about equal to or more than 300 MW (10,000 Korean won)?

[IWER: Go back to Bb207 and record if R answers the amount during the interview.]

① Less than 300 MW

③ About 300 MW

⑤ More than 300 MW

Bb210. Did it amount to less than, about equal to or more than 500 MW (10,000 Korean won)?

[IWER: Go back to Bb207 and record if R answers the amount during the interview.]

① Less than 500 MW

③ About 500 MW

⑤ More than 500 MW

Bb211. Did it amount to less than, about equal to or more than 1,000 MW (10,000 Korean won)?

[IWER: Go back to Bb207 and record if R answers the amount during the interview.]

① Less than 1,000 MW

③ About 1,000 MW

⑤ More than 1,000 MW

Bb212. Did it amount to less than, about equal to or more than 2,000 MW (10,000 Korean won)?

[IWER: Go back to Bb207 and record if R answers the amount during the interview.]

① Less than 2,000 MW

③ About 2,000 MW

⑤ More than 2,000 MW

Bb213. In the last calendar year (2007), did you give any monetary transfers to your mother while living together?

[IWER:

- Provision of dwelling space, food, etc., is excluded.

- Monetary transfers include pocket money, medical expenses, travel expenses, business expenses, etc.]

① Yes

⑤ No → Go to Bb131

Bb214. [co-residing]If so, what was the total amount of monetary transfers you gave to your mother in the last calendar year (2007)? (unit: 10,000 Korean won)

______MW [range:0~99997] →Go to Bb131
Don't know → Go to Bb215~Bb219 unfolding

Refuse to answer → Go to Bb215~Bb219 unfolding

< Bb215~Bb219. Unfolding bracket questions >

Bb215. Did it amount to less than, about equal to or more than 100 MW (10,000 Korean won)?

[IWER: Go back to Bb214 and record if R answers the amount during the interview.]

① Less than 100 MW

③ About 100 MW

⑤ More than 100 MW

Bb216. Did it amount to less than, about equal to or more than 300 MW (10,000 Korean won)?

[IWER: Go back to Bb214 and record if R answers the amount during the interview.]

① Less than 300 MW

③ About 300 MW

⑤ More than 300 MW

Bb217. Did it amount to less than, about equal to or more than 500 MW (10,000 Korean won)?

[IWER: Go back to Bb214 and record if R answers the amount during the interview.]

① Less than 500 MW

③ About 500 MW

⑤ More than 500 MW

Bb218. Did it amount to less than, about equal to or more than 1,000 MW (10,000 Korean won)?

[IWER: Go back to Bb214 and record if R answers the amount during the interview.]

① Less than 1,000 MW

③ About 1,000 MW

⑤ More than 1,000 MW

Bb219. Did it amount to less than, about equal to or more than 2,000 MW (10,000 Korean won)?
[IWER: Go back to Bb214 and record if R answers the amount during the interview.]

① Less than 2,000 MW

③ About 2,000 MW

④ More than 2,000 MW

- Transfer to other family member

Bb131. Not counting any shared housing or shared food, did you receive financial help from any family member, other than your parents and children, (e.g. parents-in-law, siblings, siblings-in-law, grandchildren, etc.) in the last calendar year (2007)?

* POINT *

- By financial help we mean giving money, helping pay bills, or covering specific types of costs, such as those for medical care or insurance, schooling, down payment for a home, rent, etc., but not counting any shared housing or shared food. The financial help can be considered support, a gift or a loan.
- Regular monetary transfer refers to the case in which you received monetary transfers regularly in a certain time interval (e.g. each month, every two months), such as monthly allowances.
- Occasional monetary transfer refers to the case in which you received monetary transfers without any regularity, such as paying for medical bills or schooling and occasional allowances.
- Monetary transfers include receiving cash as well as bills paid for your behalf. For example, your family member pays for your medical bills, insurance, tuition and so on.
- Non-monetary transfer refers to gifts and goods received.

① Yes, I received financial help.

⑤ No, I did not receive any financial help →Go to Bb150

Bb132. From whom did you receive financial help? Please identify all transfer givers. (Select multiple responses)
[IWER: R is asked only about the monetary/non-monetary transfers or gifts he/she received from other family members while not living with them for more than one month in the last calendar year (2007).]

⑤ Mother-in-law →Go to Bb134

⑥ Father-in-law →Go to Bb134

㉗ ~ ㊻ Sibling list →Go to Bb134

㊼ Bother-in-law/sister-in-law →Go to Bb134

㊽ Son-in-law/daughter-in-law →Go to Bb134

㊾ Grandchildren

㊿ Other relatives

Bb133. If others, please specify.

____

Logic

■ **Loop through Bb134 ~Bb149 as many times as the number of transfer givers reported in Bb132.**

Bb134. What kind of financial help did you receive from [giver's name chosen in Bb132]? Please choose all that apply. (Select multiple responses)

① Regular monetary transfers →Go to Bb135

② Occasional monetary transfers →Go to Bb135 or Bb142

③ Non-monetary transfers (Ex- gifts, food, clothes, home appliances etc) →Go to Bb135 or Bb142 or Bb148

Logic

■ **If Bb134=①, ① ②, ① ③, ① ② ③, then go to Bb135.**

Bb135. [non co-residing]What was the monthly average amount of regular monetary transfers you received from [giver's name chosen in Bb132] in the last calendar year (2007)? (unit: 10,000 Korean won)
_____MW [range:1~9997] →Go to Bb141

Don't know →Go to Bb136~Bb140 unfolding

Refuse to answer →Go to Bb136~Bb140 unfolding

< Bb136~Bb140. Unfolding bracket questions >

Bb136. Did it amount to less than, about equal to or more than 10 MW (10,000 Korean won) in the last calendar year (2007)?
[IWER: Go back to Bb135 and record if R answers the amount during the interview.]

① Less than 10 MW

③ About 10 MW

⑤ More than 10 MW

Bb137. Did it amount to less than, about equal to or more than 30 MW (10,000 Korean won) in the last calendar year (2007)?
[IWER: Go back to Bb135 and record if R answers the amount during the interview.]

① Less than 30 MW

③ About 30 MW

⑤ More than 30 MW

Bb138. Did it amount to less than, about equal to or more than 50 MW (10,000 Korean won) in the last calendar year (2007)?
[IWER: Go back to Bb135 and record if R answers the amount during the interview.]

① Less than 50 MW

③ About 50 MW

⑤ More than 50 MW

Bb139. Did it amount to less than, about equal to or more than 100 MW (10,000 Korean won) in the last calendar year (2007)?
[IWER: Go back to Bb135 and record if R answers the amount during the interview.]

① Less than 100 MW

③ About 100 MW

⑤ More than 100 MW

Bb140. Did it amount to less than, about equal to or more than 200 MW (10,000 Korean won) in the last calendar year (2007)?
[IWER: Go back to Bb135 and record if R answers the amount during the interview.]

① Less than 200 MW

③ About 200 MW

⑤ More than 200 MW

Bb141. Which months did you receive regular monetary transfers from [giver's name chosen in Bb132] in the last calendar year (2007)? Please choose all that apply. (Select multiple responses)
[IWER: "If you alternated between co-residing and not co-residing in 2007, please answer only in relation to the transfers you received while not living together, excluding those you received while living together."]

① January 2007

② February 2007

③ March 2007

④ April 2007

⑤ May 2007

⑥ June 2007

⑦ July 2007

⑧ August 2007

⑨ September 2007

⑩ October 2007

⑪ November 2007

⑫ December 2007

Logic

■ If Bb134=②, ② ③, ① ②, ① ② ③, then go to Bb142.

Bb142. [non co-residing]What was the total amount of occasional monetary transfers you received from [giver's name chosen in Bb132] in the last calendar year (2007)? (unit: 10,000 Korean won)

______MW [range:0~99997] →Go to Bb148
Don't know →Go to Bb143~Bb147 unfolding

Refuse to answer →Go to Bb143~Bb147 unfolding

< Bb143~Bb147. Unfolding bracket questions >

Bb143. Did it amount to less than, about equal to or more than 100 MW (10,000 Korean won) in the last calendar year (2007)?
[IWER: Go back to Bb142 and record if R answers the amount during the interview.]

① Less than 100 MW

③ About 100 MW

⑤ More than 100 MW

Bb144. Did it amount to less than, about equal to or more than 300 MW (10,000 Korean won) in the last calendar year (2007)?
[IWER: Go back to Bb142 and record if R answers the amount during the interview.]

① Less than 300 MW

③ About 300 MW

⑤ More than 300 MW

Bb145. Did it amount to less than, about equal to or more than 500 MW (10,000 Korean won) in the last calendar year (2007)?
[IWER: Go back to Bb142 and record if R answers the amount during the interview.]

① Less than 500 MW

③ About 500 MW

⑤ More than 500 MW

Bb146. Did it amount to less than, about equal to or more than 1,000 MW (10,000 Korean won) in the

last calendar year (2007)?
[IWER: Go back to Bb142 and record if R answers the amount during the interview.]

① Less than 1,000 MW

③ About 1,000 MW

⑤ More than 1,000 MW

Bb147. Did it amount to less than, about equal to or more than 2,000 MW (10,000 Korean won) in the last calendar year (2007)?
[IWER: Go back to Bb142 and record if R answers the amount during the interview.]

① Less than 2,000 MW

③ About 2,000 MW

⑤ More than 2,000 MW

Logic

■ If Bb134=③, ① ③, ② ③, ① ② ③, then go to Bb148.

Bb148. What was the type of non-monetary transfer you received from [giver's name chosen in Bb132] in the last calendar year (2007)? Please check all that apply. (Select multiple responses.)

① Leisure (e.g. travel) →Go to Bb150

② Health-related products (e.g. vitamins, equipment, etc) →Go to Bb150

③ Household items →Go to Bb150

④ Electronics →Go to Bb150

⑤ Dining out and food →Go to Bb150

⑥ Others

Bb149. If others, please specify.

____

Bb150. In the last calendar year (2007), did you give financial help to any family members, other than your parents and children, (e.g. parents-in-law, siblings, siblings-in-law, grandchildren, etc.) while living separately? Please select all that apply. (Select multiple responses.)
[IWER: R is asked only about the monetary/non-monetary transfers or gifts he/she gave other family members while not living with them for more than one month in the last calendar year (2007).]

① Yes, I gave financial help.

⑤ No, I did not give any financial help. →Go to Bb169

Bb151. To whom did you give financial help? Please identify all transfer receivers.
[IWER: R is asked only about the monetary/non-monetary transfers or gifts he/she gave other family

members while not living with them for more than one month in the last calendar year (2007).]

⑤ Mother-in-law →Go to Bb153

⑥ Father-in-law →Go to Bb153

㉗ ~ ㊻ Sibling list →Go to Bb153

㊼ Bother-in-law/sister-in-law →Go to Bb153

㊽ Son-in-law/daughter-in-law →Go to Bb153

㊾ Grandchildren →Go to Bb153

㊿ Other relatives

Bb152. If others, please specify.

____

Logic

■ Loop through Bb153~Bb168 as many times as the number of transfer receivers reported in Bb151.

Bb153. What kind of financial help did you give to [receiver's name chosen in Bb151]? Please choose all that apply. (Select multiple responses)

① Regular monetary transfers →Go to Bb154

② Occasional monetary transfers →Go to Bb154 or Bb161

③ Non-monetary transfers (Ex- gifts, food, clothes, home appliances) →Go to Bb154 or Bb161 or Bb167

Logic

■ If Bb153=①, ① ②, ① ③, ① ② ③, then go to Bb154.

Bb154. [non co-residing]What was the monthly average amount of regular monetary transfers you gave to [receiver's name chosen in Bb151] in the last calendar year (2007)? (unit: 10,000 Korean won)

_____MW [range:1~9997] →Go to Bb160

Don't know →Go to Bb155~Bb159 unfolding

Refuse to answer →Go to Bb155~Bb159 unfolding

< Bb155~Bb159. Unfolding bracket questions >

Bb155. Did it amount to less than, about equal to or more than 10 MW (10,000 Korean won) in the last calendar year (2007)?
[IWER: Go back to Bb154 and record if R answers the amount during the interview.]

① Less than 10 MW

③ About 10 MW

⑤ More than 10 MW

Bb156. Did it amount to less than, about equal to or more than 30 MW (10,000 Korean won) in the last calendar year (2007)?
[IWER: Go back to Bb154 and record if R answers the amount during the interview.]

① Less than 30 MW

③ About 30 MW

⑤ More than 30 MW

Bb157. Did it amount to less than, about equal to or more than 50 MW (10,000 Korean won) in the last calendar year (2007)?
[IWER: Go back to Bb154 and record if R answers the amount during the interview.]

① Less than 50 MW

③ About 50 MW

⑤ More than 50 MW

Bb158. Did it amount to less than, about equal to or more than 100 MW (10,000 Korean won) in the last calendar year (2007)?
[IWER: Go back to Bb154 and record if R answers the amount during the interview.]

① Less than 100 MW

③ About 100 MW

⑤ More than 100 MW

Bb159. Did it amount to less than, about equal to or more than 200 MW (10,000 Korean won) in the last calendar year (2007)?
[IWER: Go back to Bb154 and record if R answers the amount during the interview.]

① Less than 200 MW

③ About 200 MW

⑤ More than 200 MW

Bb160. Which months did you give regular monetary transfers to [receiver's name chosen in Bb151] in the last calendar year (2007)? Please choose all that apply. (Select multiple responses)

[IWER: "If you alternated between co-residing and not co-residing in 2007, please answer only in relation to the transfers you provided while not living together, excluding those you provided while living together."]

① January 2007

② February 2007

③ March 2007

④ April 2007

⑤ May 2007

⑥ June 2007

⑦ July 2007

⑧ August 2007

⑨ September 2007

⑩ October 2007

⑪ November 2007

⑫ December 2007

Logic

■ If Bb153=②, ② ③, ① ②, ① ② ③, then go to Bb161.

Bb161. [non co-residing]What was the total amount of occasional monetary transfers you gave to [receiver's name chosen in Bb151] in the last calendar year (2007)? (unit: 10,000 Korean won)

______MW [range:0~99997] →Go to Bb167
Don't know →Go to Bb162~Bb166 unfolding

Refuse to answer →Go to Bb162~Bb166 unfolding

< Bb162~Bb166. Unfolding bracket questions >

Bb162. Did it amount to less than, about equal to or more than 100 MW (10,000 Korean won) in the last calendar year (2007)?
[IWER: Go back to Bb161 and record if R answers the amount during the interview.]

① Less than 100 MW

③ About 100 MW

⑤ More than 100 MW

Bb163. Did it amount to less than, about equal to or more than 300 MW (10,000 Korean won) in the last calendar year (2007)?
[IWER: Go back to Bb161 and record if R answers the amount during the interview.]

① Less than 300 MW

③ About 300 MW

⑤ More than 300 MW

Bb164. Did it amount to less than, about equal to or more than 500 MW (10,000 Korean won) in the last calendar year (2007)?
[IWER: Go back to Bb161 and record if R answers the amount during the interview.]

① Less than 500 MW

③ About 500 MW

⑤ More than 500 MW

Bb165. Did it amount to less than, about equal to or more than 1,000 MW (10,000 Korean won) in the last calendar year (2007)?
[IWER: Go back to Bb161 and record if R answers the amount during the interview.]

① Less than 1,000 MW

③ About 1,000 MW

⑤ More than 1,000 MW

Bb166. Did it amount to less than, about equal to or more than 2,000 MW (10,000 Korean won) in the last calendar year (2007)?
[IWER: Go back to Bb161 and record if R answers the amount during the interview.]

① Less than 2,000 MW

③ About 2,000 MW

⑤ More than 2,000 MW

Logic

■ If Bb153=③, ① ③, ② ③, ① ② ③, then go to Bb167.

Bb167. What was the type of non-monetary transfer you gave to [receiver's name chosen in Bb151] in the last calendar year (2007)? Please check all that apply. (Select multiple responses.)

① Leisure (e.g. travel) →Go to Bb169

② Health-related products (e.g. vitamins, equipment, etc) →Go to Bb169

③ Household items →Go to Bb169

④ Electronics →Go to Bb169

⑤ Dining out and food →Go to Bb169

⑥ Others

Bb168. If others, please specify.

____

- The provision of care for the ADL/IADL

Bb169. Are there any family members over the age of 10 (spouse, parents, siblings, children, and spouse's parents and siblings) who are unable to carry out activities of daily living (ADL)? Activities of daily living refer to everyday routines, such as dressing, washing face and hair/brushing teeth, taking a bath or shower, eating, getting out of bed and walking out of the room, using the toilet and controlling urination or defecation. Please identify all family members with ADL difficulties. (Select multiple responses.)

② Spouse

③ Mother

④ Father

⑤ Mother-in-law

⑥ Father-in-law

⑦ ~ ㉖ Children

㉗ ~ ㊻ Sibling

㊼ Brother-in-law/sister-in-law

㊽ Son-in-law/daughter-in-law

㊾ Grandchild

㊿ Other relatives

(52) No member with ADL difficulties →Go to Bb173

Bb170. Did you care for any of [names listed in Bb169] during the past one year (not calendar year)? If so, whom did you help? Please choose all family members you cared for. (Select multiple responses.)

[IWER: Past one year means a period of one year prior to now, not 2007.]

② Spouse

③ Mother

④ Father

⑤ Mother-in-law

⑥ Father-in-law

⑦ ~ ㉖ Children

㉗ ~ ㊻ Sibling

㊼ Brother-in-law/sister-in-law

㊽ Son-in-law/sister-in-law

㊾ Grandchild

㊿ Other relatives

(53) Did not help (They were helped by other helpers, etc.) →Go to Bb173

Logic

■ Loop through Bb171~Bb172 as many times as the number of people chosen in Bb170.

Bb171. Roughly how many hours per week did you care for [name chosen in Bb170] during the past one year? (unit: an average hour per week)

[IWER: Past one year means a period of one year prior to now, not 2007.]

Average_____hours per week [range:1~168]

Bb172. How many weeks did you care for [name chosen in Bb170] during the past one year? (unit: a week)
[IWER: Calculate 1 month as 4 weeks, 6 months as 26 weeks, 1 year as 52 weeks. For example, 5 months is converted into 20 weeks, and enter 30 weeks for 7 months because it is equal to 6 months (26 weeks) plus 1 month (4 weeks). Past one year refers to a period of one year prior to now, not 2007.]
_______weeks [range:1~52]

Bb173. Did you help any of your family members over the age of 10 (spouse, parents, parents-in-law, siblings and/or children) who are not living with you with the various activities he/she is unable to do due to physical or mental weakness (e.g. personal grooming, cleaning and tidying up the house, making bed and washing dishes, etc., preparing meals, doing laundry, going out a short distance, using transportation, shopping, managing money, making and taking phone calls, and taking medications) during the past one year? If you did, whom did you help? Please choose all family members you helped. (Select multiple responses.)
[IWER: Past one year means a period of one year prior to now, not 2007.]
② Spouse
③ Mother
④ Father
⑤ Mother-in-law
⑥ Father-in-law
⑦ ~ ㉖ Children
㉗ ~ ㊻ Sibling
㊼ Brother-in-law/sister-in-law
㊽ Son-in-law/sister-in-law
㊾ Grandchild
㊿ Other relatives
(54) Did not help (They were helped by other helpers, etc.) →Go to Bb176

Logic
■ Loop through Bb174~Bb175 as many times as the number of people chosen in Bb173.

Bb174. Roughly how many hours per week did you help out [name chosen in Bb173] during the past one year? (unit: an average hour per week)

[IWER: Past one year means a period of one year prior to now, not 2007.]

Average_____hours per week [range:1~168]

Bb175. How many weeks did you care for [name chosen inBb173] during the past one year?
[IWER: Calculate 1 month as 4 weeks, 6 months as 26 weeks, 1 year as 52 weeks. For example, 5 months is converted into 20 weeks, and enter 30 weeks for 7 months because it is equal to 6 months (26 weeks) plus 1 month (4 weeks). Past one year refers to a period of one year prior to now, not 2007.]
_______weeks [range:1~52]

Bb176. How often did R receive assistance in answering section Bb-FAMILY AND FAMILY TRANSFER (PARENTS & SIBLINGS)?
① Never →Go to section C1.
② A few times →Go to section C1.
③ Most or all of the time →Go to section C1.
④ The section was done by a proxy respondent.

Bb177. What is the proxy's relationship to R? If unknown, please ask the proxy. What is your relationship to R?
① Spouse
② Mother
③ Father
④ Mother-in-law
⑤ Father-in-law
⑥ Brother/sister
⑦ Brother-in-law/sister-in-law
⑧ Son/daughter
⑨ Son-in-law/Daughter-in-law
⑩ Grandchild
⑪ Other relatives
⑫ Helper or other non-relatives
→Go to section C1.

C1. HEALTH STATUS

Logic

■ C001 and C152 are asked at the start and end of HEALTH section, but in a random order.

C001. Next I have some questions about your health. Would you say your health is excellent, very good, good, fair, or poor?

① Excellent

② Very good

③ Good

④ Fair

⑤ Poor

C152. Next I have some questions about your health. Would you say your health is very good, good, fair, poor or very poor?

① Very good

② Good

③ Fair

④ Poor

⑤ Very poor

C002. How has your health status changed since the previous interview?

① Greatly improved

② Improved

③ Same

④ Worsened

⑤ Greatly worsened

Logic

■ If R said in the previous interview that he/she didn't receive a disability diagnosis, then go to C003. Else, go to C005.

C003. Have you ever received a disability diagnosis from a doctor since the previous interview?

① Yes, received a disability diagnosis

⑤ No, haven't received a disability diagnosis →Go to C005

C004. What was your type of disability? Please choose all that apply.

① Physical disability

② Disability of brain lesion

③ Visual disability

④ Hearing disability

⑤ Speech disability

⑥ Kidney dysfunction

⑦ Cardiac dysfunction

⑧ Mental disorder

⑨ Developmental disorder (autism)

C005. Does your health condition hamper you doing work?

① Yes, very much so

② Yes, to some degree

③ No, not much

④ No, not at all

- Chronic Disease

Logic

■ If R said that he/she didn't receive a diagnosis of high blood pressure or 'Don't know' or 'Refuse to answer' in the previous interview, then go to C006. If R received such a diagnosis, then go to C008

C006. Have you ever received a diagnosis of high blood pressure from a doctor since the previous interview? (unit: year and month combined into 6 digits)

① Yes

⑤ No →Go to C011

C007 When were you first diagnosed with hypertension?

[IWER: Enter the year and month using 6 digits. For example, mark 200901 for January 2007. If the month is not clear, enter 200700.]

____________________ [range:200600~200812] →Go to C009

C008. How have your symptoms of high blood pressure changed since the previous interview?

① Completely recovered

② Improved

③ Same

④ Worsened

⑤ Greatly worsened

C009. Are you currently taking any medication or receiving treatment to lower your blood pressure?

① Yes

⑤ No

C010. Does your high blood pressure limit your daily activities?

① Yes

⑤ No

Logic

■ If R said that he/she didn't receive a diabetes diagnosis or 'Don't know' or 'Refuse to answer' in the previous interview, then go to C011. If R received such a diagnosis, then go to C013.

C011. Have you ever received a diagnosis of diabetes or high blood sugar from a doctor since the previous interview?

① Yes

⑤ No →Go to C016

C012. When were you first diagnosed with diabetes or high blood sugar? (unit: year and month combined into 6 digits)

[IWER: Enter the year and month using 6 digits. For example, mark 200701 for January 2007. If the month is not clear, enter 200700.]

____________________ [range:200600~200812] →Go to C014

C013. How have your symptoms of diabetes or high blood sugar changed since the previous interview?

① Completely recovered

② Improved

③ Same

④ Worsened

⑤ Greatly worsened

C014. Are you currently taking any medication or receiving treatment to treat your diabetes or stabilize your blood sugar level?

① Yes

⑤ No

C015. Does your diabetes limit your daily activities?

① Yes

⑤ No

Logic

■ If R said that he/she didn't receive a diagnosis of cancer or a malignant tumor or 'Don't know' or 'Refuse to answer' in the previous interview, then go to C016. If R received such a diagnosis, then go to C020.

C016. Have you ever received a diagnosis of cancer or a malignant tumor (excluding minor skin cancer) from a doctor since the previous interview?

① Yes

⑤ No →Go to C023

C017. When were you first diagnosed with cancer or a malignant tumor, excluding minor skin cancer? (unit: year and month combined into 6 digits)

[IWER: Enter the year and month using 6 digits. For example, mark 200701 for January 2007. If the month is not clear, enter 200700.]

______________ [range: 200600~200812]

C018. In which organ or part of your body do you have cancer?

① Liver →Go to C021

② Stomach →Go to C021

③ Lung →Go to C021

④ Colon →Go to C021

⑤ Thyroid →Go to C021

⑥ Breast →Go to C021

⑦ Cervix →Go to C021

⑧ Ovary →Go to C021

⑨ Others

C019. If others, please specify.

___________________ →Go to C021

C020. How have your symptoms of cancer or malignant tumor (excluding minor skin cancer) changed since the previous interview?

① Completely recovered

② Improved

③ Same

④ Worsened

⑤ Greatly worsened

C021. Are you currently taking any medication to alleviate your symptoms (pain, nausea, rash, etc.) or receiving cancer treatment such as chemotherapy?

① Yes

⑤ No

C022. Does your cancer limit your daily activities?

① Yes

⑤ No

Logic

■ If R said that he/she didn't receive a diagnosis of chronic lung disease, such as bronchitis or emphysema, or 'Don't know' or 'Refuse to answer' in the previous interview, then go to C023. If R received such a diagnosis, then go to C025.

C023. Have you ever received a diagnosis of chronic lung disease, such as bronchitis or emphysema, from a doctor since the previous interview?

① Yes

⑤ No →Go to C028

C024. When were you first diagnosed with chronic lung disease, such as bronchitis or emphysema? (unit: year and month combined into 6 digits)

[IWER: Enter the year and month using 6 digits. For example, mark 200701 for January 2007. If the month is not clear, enter 200700.]

______________ [range:200600~200812] →Go to C026

C025. How have your symptoms of chronic lung disease, such as bronchitis or emphysema, changed since the previous interview?

① Completely recovered

② Improved

③ Same

④ Worsened

⑤ Greatly worsened

C026. Are you currently taking any medication or receiving treatment in relation to your lung disease?
① Yes
⑤ No

C027. Does your lung condition limit your daily activities?
① Yes
⑤ No

Logic
■ If R said that he/she didn't receive a diagnosis of liver disease (all types of liver disease except fatty liver) or 'Don't know' or 'Refuse to answer' in the previous interview, then go to C028. If R received such a diagnosis, then go to C030.

C028. Have you ever received a diagnosis of liver disease from a doctor since the previous interview? (All types of liver disease except fatty liver)
① Yes
⑤ No →Go to C033

C029. When were you first diagnosed with liver disease? (unit: year and month combined into 6 digits) [IWER: Enter the year and month using 6 digits. For example, mark 200701 for January 2007. If the month is not clear, enter 200700.]
______________ [range:200600~200812] →Go to C031

C030. How have your symptoms of liver disease (All types of liver disease except fatty liver) changed since the previous interview?
① Completely recovered
② Improved
③ Same
④ Worsened
⑤ Greatly worsened

C031. Are you currently taking any medication or receiving treatment due to your liver disease?
① Yes
⑤ No

C032. Does your liver disease limit your daily activities?
① Yes

⑤ No

Logic

■ If R said that he/she didn't receive a diagnosis of a heart attack, angina pectoris, myocardial infarction, congestive heart failure or any other heart disease, or 'Don't know' or 'Refuse to answer' in the previous interview, then go to C033. If R received such a diagnosis, then go to C035.

C033. Have you ever received a diagnosis of a heart attack, angina pectoris, myocardial infarction, congestive heart failure or other heart disease from a doctor since the previous interview?

① Yes

⑤ No →Go to C038

C034. When were you first diagnosed with such heart disease?

[IWER: Enter the year and month using 6 digits. For example, mark 200701 for January 2007. If the month is not clear, enter 200700.]

______________ [range:200600~200812] →Go to C036

C035. How have your symptoms of heart disease changed since the previous interview?

① Completely recovered

② Improved

③ Same

④ Worsened

⑤ Greatly worsened

C036. Are you currently taking any medication or receiving treatment due to your heart disease?

① Yes

⑤ No

C037. Does your heart disease limit your daily activities?

① Yes

⑤ No

Logic

■ If R said that he/she didn't receive a diagnosis of cerebrovascular disease or 'Don't know' or 'Refuse to answer' in the previous interview, then go to C038. If R received such a diagnosis, then go to C040.

C038. Have you ever received a diagnosis of cerebrovascular disease (cerebral apoplexy, cerebral hemorrhage, cerebral infarction, etc.) from a doctor since the previous interview?

① Yes

③ Possible stroke or transient ischemic attack

⑤ No →Go to C043

C039. When were you first diagnosed with cerebrovascular disease? (unit: year and month combined into 6 digits)

[IWER: Enter the year and month using 6 digits. For example, mark 200701 for January 2007. If the month is not clear, enter 200700.]

______________ [range:200600~200812] →Go to C041

C040. How have your symptoms of cerebrovascular disease changed since the previous interview?

① Completely recovered

② Improved

③ Same

④ Worsened

⑤ Greatly worsened

C041. Are you currently taking any medication or receiving treatment due to your cerebrovascular disease or its complications?

① Yes

⑤ No

C042. Does your cerebrovascular disease limit your daily activities?

① Yes

⑤ No

Logic

■ If R said that he/she didn't receive a diagnosis of emotional, nervous, or psychiatric problems or 'Don't know' or 'Refuse to answer' in the previous interview, then go to C043. If R received such a diagnosis, then go to C045.

C043. Have you ever received a diagnosis of any emotional (depression, anxiety disorder, etc.), nervous (insomnia, too much stress, etc.), or psychiatric (mental disorder, difficulties in maintaining interpersonal relationships, etc.) problems from a doctor since the previous interview?

① Yes

⑤ No →Go to C048

C044. When were you first diagnosed with such problems? (unit: year and month combined into 6 digits)

[IWER: Enter the year and month using 6 digits. For example, mark 200701 for January 2007. If the month is not clear, enter 200700.]

______________ [range:200600~200812] →Go to C046

C045. How have your symptoms of emotional, nervous, or psychiatric problems changed since the previous interview?

① Completely recovered

② Improved

③ Same

④ Worsened

⑤ Greatly worsened

C046. Are you currently taking tranquilizers, antidepressants, sedatives or sleeping pills or receiving psychiatric or psychological treatment?

① Yes

⑤ No

C047. Do your emotional, nervous or psychiatric problems limit your daily activities?

① Yes

⑤ No

Logic

■ If R said that he/she didn't receive a diagnosis of arthritis or rheumatism or 'Don't know' or 'Refuse to answer' in the previous interview, then go to C048. If R received such a diagnosis, then go to C050.

C048. Have you ever received a diagnosis of arthritis or rheumatism from a doctor since the previous interview?

① Yes

⑤ No →Go to C053

C049. When were you first diagnosed with arthritis or rheumatism? (unit: year and month combined into 6 digits)

[IWER: Enter the year and month using 6 digits. For example, mark 200701 for January 2007. If the month is not clear, enter 200700.]

______________ [range:200600~200812] →Go to C051

C050.How have your symptoms of arthritis or rheumatism changed since the previous interview?

① Completely recovered

② Improved

③ Same

④ Worsened

⑤ Greatly worsened

C051. Are you currently taking any medication or receiving treatment for your arthritis or rheumatism?

① Yes

⑤ No

C052. Does your arthritis or rheumatism limit your daily activities?

① Yes

⑤ No

C053. Have you ever been in a traffic accident and received medical treatment since the previous interview?

① Yes

⑤ No →Go to C056

C054. When did you have the traffic accident? If you had more than two accidents, please answer about the most recent one. (unit: year and month combined into 6 digits)

[IWER: Enter the year and month using 6 digits. For example, mark 200701 for January 2007. If the month is not clear, enter 200700.]

___________________ [range:200600~200812]

C055. Does your injury caused by the traffic accident limit your daily activities?

① Yes

⑤ No

C056. Have you ever fallen down since the previous interview?

① Yes

⑤ No →Go to C061

C057. How many times have you had a fall accident since the previous interview? (unit: a time)

______times [range:1~ 100]

C058. In your most recent fall, did you injure yourself seriously enough to need medical treatment?

① Yes

⑤ No

C059. Have you ever fractured your hip due to a fall accident?

① Yes

⑤ No

C060. Does your injury or fracture due to the fall limit your daily activities?

① Yes

⑤ No

C061. How much do you usually worry about falling down?

① Not at all

③ A little bit

⑤ A lot

C062. Are there any activities that you refrain from doing due to the fear of falling down?

① Yes

⑤ No

Logic

■ If R is male, then go to C063~C067(Prostatic disease).

■ If R said that he didn't receive a diagnosis of prostate disease or 'Don't know' or 'Refuse to answer' in the previous interview, then go to C063. If R received such a diagnosis, then go to C065.

C063. Have you ever received a diagnosis of prostate disease from a doctor since the previous interview?

① Yes

⑤ No →Go to C074

C064. When were you first diagnosed with prostate disease? (unit: year and month combined into 6 digits)

[IWER: Enter the year and month using 6 digits. For example, mark 200701 for January 2007. If the month is not clear, enter 200700.]

______________ [range:200600~200812] →Go to C066

C065. How have your symptoms of prostate disease changed since the previous interview?

① Completely recovered

② Improved

③ Same

④ Worsened

⑤ Greatly worsened

C066. Are you currently taking any mediation or receiving treatment for your prostate disease?

① Yes

⑤ No

C067. Does your prostate disease limit your daily activities?

① Yes →Go to C074

⑤ No →Go to C074

Logic

■ If R is female, then go to C068~C073(Urinary incontinence disease).

■ If R said that she didn't receive a diagnosis of urinary incontinence or 'Don't know' or 'Refuse to answer' in the previous interview, then go to C068. If R received such a diagnosis, then go to C069.

C068. The following questions may not be easy to answer. I would be grateful if you could answer them as honestly as possible. Did you ever experience urinary incontinence during the past one year?

① Yes →Go to C070

⑤ No →Go to C074

C069. How have your symptoms of urinary incontinence changed since the previous interview?

① Completely recovered

② Improved

③ Same

④ Worsened

⑤ Greatly worsened

C070. How many days did you experience urinary inconsistence last month? (unit: a day)

_____days [range: 0~ 31] →Go to C073

[98]Don't know →Go to C071

[99]Refuse to answer →Go to C071

C071. Was that more than 5 days?

① Yes →Go to C072

⑤ No →Go to C073

C072. Was that more than 15 days?

① Yes

⑤ No

C073. Did you ever use any absorbent products, such as pads, special underwear, sanitary napkins, or toilet paper?

① Yes

⑤ No

C074. Now I have some questions about your eyesight. Do you usually wear glasses or corrective lens?

① Yes

③ Visually disabled (blind)→Go to C082

⑤ No

C075. How good is your eyesight (including corrected vision)?

① Very good

② Good

③ Fair

④ Poor

⑤ Very poor

C076. How good is your eyesight (including corrected vision) for seeing things at a distance, like recognizing a friend across a street?

① Very good

② Good

③ Fair

④ Poor

⑤ Very poor

C077. How good is your eyesight (including corrected vision) for seeing things up close, like reading a newspaper?

① Very good

② Good

③ Fair

④ Poor

⑤ Very poor

C078. Have you ever had a cataract surgery since the previous interview?

① Yes

⑤ No →Go to C080

C079. Have you had a cataract surgery on both eyes or just one?

① One eye only

⑤ Both eyes

C080. Has a doctor ever treated you for glaucoma since the previous interview?

① Yes

⑤ No

C081. Does your eyesight limit your daily activities?

① Yes

⑤ No

C082. Now I have some questions about your hearing. Do you wear a hearing aid?

① Yes

⑤ No

C083. How good is your hearing? If you use a hearing aid, answer about your hearing when you wear it.

① Very good

② Good

③ Fair

④ Poor

⑤ Very poor

C084. Does your hearing limit your daily activities?

① Yes

⑤ No

C085. Now I have some questions about your dental health. Do you wear dentures?

① Yes

⑤ No

C086. How easily can you chew solid foods, such as meat or apples, without the help of dentures?

① Very well

② Pretty well

③ Fair

④ Not well

⑤ Not at all

Logic

■ If C085=①, then go to C087.

C087. How well can you chew solid foods, such as meat or apples, when wearing dentures?

① Very well

② Pretty well

③ Fair

④ Not well

⑤ Not at all

C088. Now I have some questions about body pain. In what part of your body do you feel pain? Please list all body parts in which you are currently feeling pain. (Select multiple responses, but not with 14.)

① Head (Headache) →Go to C089

② Shoulder →Go to C090

③ Arm →Go to C091

④ Wrist →Go to C092

⑤ Fingers →Go to C093

⑥ Chest →Go to C094

⑦ Stomach (Stomachache) →Go to C095

⑧ Back →Go to C096

⑨ Buttocks →Go to C097

⑩ Leg →Go to C098

⑪ Knee →Go to C099

⑫ Ankle →Go to C100

⑬ Toes →Go to C101

⑭ No pain →Go to C103

C089. How bad is the headache?

① Mild

③ Moderate

⑤ Severe

C090. How bad is the shoulder pain?

① Mild

③ Moderate

⑤ Severe

C091. How bad is the arm pain?

① Mild

③ Moderate

⑤ Severe

C092. How bad is the wrist pain?

① Mild

③ Moderate

⑤ Severe

C093. How bad is the finger pain?

① Mild

③ Moderate

⑤ Severe

C094. How bad is the chest pain?

① Mild

③ Moderate

⑤ Severe

C095. How bad is the stomachache?

① Mild

③ Moderate

⑤ Severe

C096. How bad is the back pain?

① Mild

③ Moderate

⑤ Severe

C097. How bad is the buttocks pain?

① Mild

③ Moderate

⑤ Severe

C098. How bad is the leg pain?

① Mild

③ Moderate

⑤ Severe

C099. How bad is the knee pain?

① Mild

③ Moderate

⑤ Severe

C100. How bad is the ankle pain?

① Mild

③ Moderate

⑤ Severe

C101. How bad is the toe pain?

① Mild

③ Moderate

⑤ Severe

C102. Does the pain make it difficult for you to do daily activities?

① Yes

⑤ No

C103. Do you have any other disease or health problem we have not talked about so far?

① Yes

⑤ No →Go to C105

C104. What is that disease or health problem?

____

C105. How much do you weigh? (unit: Kilogram)

_____kilograms [range:30~200]

C106. Did you gain or lose 5 or more kilograms during the past one year?

① Yes, I gained weight

② Yes, I lost weight

③ Yes, I gained weight and then lost it again

④ Yes, I lost weight and then gained it again

⑤ Changelessness

C107. How tall are you? (unit: Centimeter)

_____centimeters [range:70~210]

C108. The next few questions are about exercise. Do you work out more than once a week?

① Yes →Go to C111

⑤ No

C109. What is the main reason that you cannot exercise regularly?

① Too busy →Go to C114

② No space or place to work out →Go to C114

③ Too lazy →Go to C114

④ Do not like exercise →Go to C114

⑤ Never thought about doing exercise →Go to C114

⑥ Others

C110. If others, please specify.

____

C111. How often do you work out per week? (unit : a time/per week)

_____times / per week [range:1~97]

C112. How long do you work out per session? (unit : a minutes)

[IWER: Calculate one hour as 60 minutes. For example, mark 150 minutes for 2 hours and 30 minutes.]

_____minutes [range:1~1440]

C113. How long have you been working out regularly?

① Less than 3 months

② 4~6 months

③ More than 7 months ~ less than 1 year

④ 1~2 years

⑤ 3~4 years

⑥ 5~6 years

⑦ More than 7 years

C114. Now I am going to ask you about the meals you had for the last two days. Did you have three meals yesterday? Choose all that apply. (Select multiple responses.)

[IWER: Caution! Choose only the meals R actually had.]

① I had breakfast

② I had lunch

③ I had dinner

④ I did not eat anything

C115. Did you have three meals the day before yesterday? Choose all that apply. (Select multiple responses.)

[IWER: Caution! Choose only the meals R actually had.]

① I had breakfast

② I had lunch

③ I had dinner

④ I did not eat anything

Logic

■ If (R was identified as a smoker in the previous interview and C116=①) or (R was identified as a

non-smoker in the previous interview and C116=①), then go to C117. If R was identified as a smoker in the previous interview and C116=⑤, then go to C121. If R was identified as a non-smoker in the previous interview and C116=⑤, then go to C122.

C116. Have you smoked more than 5 packs of cigarettes (100 cigarettes) in total since the previous interview?

① Yes →Go to C117

⑤ No →Go to C121 or C122

Logic

■ If C116=①, then go to C117.

C117. Do you smoke cigarettes now?

① Yes

⑤ No

Logic

■ If R was identified as a smoker in the previous interview and C116=①, then go to C118.

C118. When did you first start smoking?

[IWER: Calculate the year and month after hearing R's response. For example, enter 200803 if R started smoking in March 2008.]

__________ [range: 200600~200812]

Logic

■ If C117=①, then go to C119.

C119. How many cigarettes or packs do you usually smoke in a day now? (unit: a cigarette)

[IWER: One pack is 20 cigarettes. For example, mark 30 for one and a half packs]

_____cigarettes / days [range:1~100] →Go to C122

Logic

■ If C117=⑤, then go to C120.

C120. When you were smoking the most, how many cigarettes or packs did you usually smoke in a day?

[IWER: One pack is 20 cigarettes. For example, mark 30 for one and a half packs.]

_____cigarettes / days [range:1~200]

Logic

■ If (R was identified as a smoker in the previous interview and C116=⑤) or C117=⑤, then go to C121.

C121. When did you stop smoking? (unit: year and month combined into 6 digits)

[IWER: Calculate the year and month after hearing R's response. For example, enter 200803 if R quitted smoking in March 2008.]

__________ [range:200600~200812] →Go to C122

Logic

■ If R answered in the previous interview that he/she drank and C122=①, then go to C125.

If R answered in the previous interview that he/she did not drink and C122=①, then go to C124.

If R answered in the previous interview that he/she drank and C122=⑤, then go to C123.

If R answered in the previous interview that he/she did not drink and C122=⑤, then go to C141.

C122. Do you sometimes or often drink any alcoholic beverages, such as beer, wine, or liquor?

① Yes →Go to C124 (Non-drinker(2006)) or C125(Drinker(2006))

⑤ No →Go to C123 (Drinker(2006)) or C141(Non-drinker(2006))

Logic

■ If R answered in the previous interview that he/she drank and C122=⑤, then go to C123.

C123. When did you quit drinking? (unit: year and month combined into 6 digits)

[IWER: Calculate the year and month after hearing R's response. For example, enter 200803 if R quitted smoking in March 2008.]

________________ [range:200600~200812] →Go to C141

Logic

■ If R answered in the previous interview that he/she did not drink and C122=①, then go to C124.

C124. When did you start drinking? (unit: year and month combined into 6 digits)

[IWER: Calculate the year and month after hearing R's response. For example, enter 200803 if R started drinking in March 2008.]

________________ [range:200600~200812]

C125. Now, I am going to ask you how often and how much you drank during the past one year. Please answer how often you drank per month, and how much you drank at a time on average. I will repeat the questions for different types of alcoholic beverages.

① Continue

C126. How often did you drink Soju(Korean distilled spirits) in a month?

① None or less than once a month →Go to C128

② Once a month

③ 2~3 times a month

④ Once a week

⑤ 2~3 times a week

⑥ 4~6 times a week

⑦ Once a day

⑧ More than twice a day

C127. How many glasses of Soju(Korean distilled spirits) did you drink at a time? (unit: a glass)

[1bottle = 6.5 glasses, a glass of Soju(Korean distilled spirits) = 50cc]

Average_______glasses

C128. How often did you drink beer in a month?

① None or less than once a month →Go to C130

② Once a month

③ 2~3 times a month

④ Once a week

⑤ 2~3 times a week

⑥ 4~6 times a week

⑦ Once a day

⑧ More than twice a day

C129. How many glasses of beer did you drink at a time? (unit: a glass)

[IWER: If R answered a bottle, ask size of a bottle(500cc or 1.5L). And then calculate the number of glasses by a glass of beer(220cc). For example, two bottles of beer are about four glasses]

[1bottle = 2.5 glasses of beer, a glass of beer = 220cc]

Average_______glasses

C130. How often did you drink Makgeolli(unstrained rice wine) in a month?

① None or less than once a month →Go to C132

② Once a month

③ 2~3 times a month

④ Once a week

⑤ 2~3 times a week

⑥ 4~6 times a week

⑦ Once a day

⑧ More than twice a day

C131. How many glasses of Makgeolli(unstrained rice wine) did you drink at a time?

[IWER: 1 bottle = 7 glasses, a glass of Makgeolli(unstrained rice wine) = 240cc]

Average_______glasses

C132. How often did you drink whisky and other liquors in a month?

① None or less than once a month →Go to C134

② Once a month

③ 2~3 times a month

④ Once a week

⑤ 2~3 times a week

⑥ 4~6 times a week

⑦ Once a day

⑧ More than twice a day

C133. How many glasses of whisky or liquor did you drink at a time?

[IWER: a glass of whisky = 30cc, calculate the number of a glass by size of a bottle]

Average_______glasses

C134. How often did you drink wine in a month?

① None or less than once a month →Go to C136

② Once a month

③ 2~3 times a month

④ Once a week

⑤ 2~3 times a week

⑥ 4~6 times a week

⑦ Once a day

⑧ More than twice a day

C135. How many glasses of wine did you drink at a time?

[IWER: 1 bottle = 8 glasses, a glass of wine = 90cc]

Average_______glasses

C136. Have you ever felt that you should quit drinking?

① Yes

⑤ No

C137. Has anyone complained about your drinking habit?

① Yes

⑤ No →Go to C139

C138. Have people ever annoyed you by complaining about your drinking habit?

① Yes

⑤ No

C139. Have you ever felt bad or guilty about drinking?

① Yes

⑤ No

C140. Have you ever taken a drink first thing in the morning to calm your nerves or get rid of a hangover?

① Yes

⑤ No

- Depression

C141. Did you ever have sad or depressed feelings lasting for two weeks or more during the past one year?

① Yes

③ Did not feel depressed because I was taking anti-depressants →Go to C152

⑤ No

C142. Next I will ask you about your feelings and behavior during the last week. Please answer how often you felt or behaved in the following ways. How often did you lose interest in most things last week?

(CED-D 10: I was bothered by things that usually do not bother me.)

① Very rarely (less than one day)
② Sometimes (1~2 days)
③ Often (3~4 days)
④ Almost always (5~7 days)

C143. How often did you have trouble concentrating last week?
(CED-D 10: I had trouble keeping my mind on what I was doing.)
① Very rarely (less than one day)
② Sometimes (1~2 days)
③ Often (3~4 days)
④ Almost always (5~7 days)

C144. How often did you feel depressed last week?
(CED-D 10: I felt depressed.)
① Very rarely (less than one day)
② Sometimes (1~2 days)
③ Often (3~4 days)
④ Almost always (5~7 days)

C145. How often did you feel tired out or low in energy last week?
① Very rarely (less than one day)
② Sometimes (1~2 days)
③ Often (3~4 days)
④ Almost always (5~7 days)

C146. How was your last week? How often did you feel pretty good?
① Very rarely (less than one day)
② Sometimes (1~2 days)
③ Often (3~4 days)
④ Almost always (5~7 days)

C147. How often were you afraid of something last week?
(CED-D 10: I felt fearful.)
① Very rarely (less than one day)
② Sometimes (1~2 days)
③ Often (3~4 days)
④ Almost always (5~7 days)

C148. How often did you have trouble falling asleep last week?

(CED-D 10 : my sleep was restless.)

① Very rarely (less than one day)

② Sometimes (1~2 days)

③ Often (3~4 days)

④ Almost always (5~7 days)

C149. How often did you feel satisfied overall last week?

① Very rarely (less than one day)

② Sometimes (1~2 days)

③ Often (3~4 days)

④ Almost always (5~7 days)

C150. How often did you feel alone last week?

(CED-D 10: I felt lonely.)

① Very rarely (less than one day)

② Sometimes (1~2 days)

③ Often (3~4 days)

④ Almost always (5~7 days)

C151. How often did you feel down, no good or worthless last week?

(CED-D 10: I could not "get going".)

① Very rarely (less than one day)

② Sometimes (1~2 days)

③ Often (3~4 days)

④ Almost always (5~7 days)

C153. [IWER: How often did R receive assistance in answering section C1-HEALTH STATUS?]

① Never →Go to section C2.

② A few times →Go to section C2.

③ Most or all of the time →Go to section C2.

④ The section was done by a proxy respondent.

C154. What is the proxy's relationship to R? If unknown, please ask the proxy. What is your relationship to R?

① Spouse

② Mother

③ Father

④ Mother-in-law

⑤ Father-in-law

⑥ Brother/sister

⑦ Brother-in-law/sister-in-law

⑧ Son/daughter

⑨ Son–in–law/Daughter–in–law

⑩ Grandchild

⑪ Other relatives

⑫ Helper or other non-relatives

→Go to section C2.

C2. FUNCTIONAL LIMITATIONS AND HELPERS

- ADL

C201. Now I would like to know how much you need someone to help you with very simple daily activities. Please give your response based on your activities over the past one week. If you are now receiving help with doing such activities due to an illness or injury but expect the situation to last less than three months, then answer that you don't need help.
Do you have difficulties with dressing, including taking clothes out of a closet and buttoning or zipping up? Do you need someone to help you with that?
① No, I don't need any help.
③ Yes, I need help to some extent.
⑤ Yes, I need help in every respect.

C202. Do you have difficulties in washing your face and hair and brushing your teeth? Do you need someone to help you with that?
① No, I don't need any help.
③ Yes, I need help to some extent.
⑤ Yes, I need help in every respect.

C203. Do you have difficulties with bathing or showering (You may not scrub your back alone.)? Do you need someone to help you with that?
① No, I don't need any help.
③ Yes, I need help to some extent.
⑤ Yes, I need help in every respect.

C204. Do you have difficulties with eating (You may use forks or other tools.)? Do you need someone to help you with that?
① No, I don't need any help.
③ Yes, I need help to some extent.
⑤ Yes, I need help in every respect.

C205. Do you have difficulties in getting out of bed and walking out of the room (You may use equipment or devices to get up.)? Do you need someone to help you with that?
① No, I don't need any help.

③ Yes, I need help to some extent.

⑤ Yes, I need help in every respect.

C206. Do you have difficulties in using the toilet (including taking off clothes and cleaning oneself after defecating or urinating. You may use a portable toilet.). Do you need someone to help you with that?

① No, I don't need any help.

③ Yes, I need help to some extent.

⑤ Yes, I need help in every respect.

C207. Do you have difficulties in controlling urination and defecation (You may use a catheter (conduit) or a pouch by yourself.)? Do you need someone to help you with that?

① No, I don't need any help.

③ Yes, I need help to some extent.

⑤ Yes, I need help in every respect.

- IADL

C208. Now I will ask questions about a bit more difficult activities. Please give your response based on your activities over the past one week. If you are temporarily receiving help with doing such activities for a certain reason but expect the situation to last less than three months, then answer that you don’t need help.

Do you have difficulties with personal grooming (including brushing hair, clipping nails/toenails, putting on make-up (female) and shaving (male))? Do you need someone to help you with that?

① No, I don't need any help.

③ Yes, I need help to some extent.

⑤ Yes, I need help in every respect.

C209. Do you need someone to help you with doing housework, such as cleaning or tidying up the house, making bed and washing dishes?

[IWER: If R cannot mop the floor, but can scrub, or R cannot fold heavy bedding, but is able to do light ones, then mark ③.]

① No, I don't need any help.

③ Yes, I need help to some extent.

⑤ Yes, I need help in every respect.

C210. Do you need someone to help you with preparing meals? Preparing meals means preparing ingredients, cooking, and setting the table.
[IWER: If another person prepares ingredients or if R can cook rice, but is unable to prepare side dishes, then mark ③.]
① No, I don't need any help.
③ Yes, I need help to some extent.
⑤ Yes, I need help in every respect.

C211. Do you need someone to help you with doing laundry, that is, doing laundry using a washing machine or by hand and hanging out the wash to dry?
[IWER: If R is unable to launder large items but can do small items, such as underwear and socks, or if R can do laundry but is unable to hang out the wash, then mark ③.]
① No, I don't need any help.
③ Yes, I need help to some extent.
⑤ Yes, I need help in every respect.

C212. Do you need someone to help you with going out a short distance without using transportation?
[IWER: Enter ① if R uses assistance devices, such as a cane. Enter ③ if R needs to ride a wheelchair.]
① No, I don't need any help.
③ Yes, I need help to some extent.
⑤ Yes, I need help in every respect.

C213. Do you need someone to help you with going out using transportation, such as buses, subways, taxies, and cars?
① No, I don't need any help.
③ Yes, I need help to some extent.
⑤ Yes, I need help in every respect.

C214. Do you need someone to help you with shopping, that is, deciding what to buy, paying money and getting change?
① No, I don't need any help.
③ Yes, I need help to some extent.
⑤ Yes, I need help in every respect.

C215. Do you need someone to help you with managing money, such as managing small sums of pocket money, savings accounts and other assets?

[IWER: Enter ③ if large sums of money is managed by someone else.]

① No, I don't need any help.

③ Yes, I need help to some extent.

⑤ Yes, I need help in every respect.

C216. Do you need someone to help you with making or taking phone calls?

[IWER: Enter ③ if R can only dial the numbers she/he knows.]

① No, I don't need any help.

③ Yes, I need help to some extent.

⑤ Yes, I need help in every respect.

C217. Do you need someone to help you with taking medications, that is, taking the right portion of medication right on time?

① No, I don't need any help.

③ Yes, I need help to some extent.

⑤ Yes, I need help in every respect.

- ADL/IADL HELPER

Logic

■ If R selected any answer other than ① in any of C201 ~ C217, then go to C218.

■ If R answered ① to all of C201 ~ C217, then go to C224.

Logic

■ Loop through C218~222 up to five times. If C218=56, then go to C223. (However, if C218=56 in the first loop, then go to C224.)

C218. If you need help with activities of daily living, name the five people who help you the most in order. Who is the person that comes [1st/2nd/..] in helping you? Please state the person's name or relationship with you.

② Spouse

③ Mother

④ Father

⑤ Mother-in-law

⑥ Father-in-law

⑦ ~ ㉖ Children

㉗ ~ ㊻ Sibling

㊼ Brother-in-law/sister-in-law

㊽ Son-in-law/sister-in-law

㊾ Grandchild

㊿ Other relatives

(52) Voluntary worker

(53) Publicly provided helper

(54) Personally hired helper

(55) Helper or other non-relatives

(56) No one helped →Go to C223 (In the first loop, go to C224)

C219. How many days did [name selected in C218] help you during the past one month? (unit: a day)
______days [range:1~31]

C220. How many hours per day does [name selected in C218] usually help you? (unit: an hour)
[IWER: less than an hour=1]
______hours [range:1~24]

C221. Is [name selected in C218] paid to help you?

① Yes

⑤ No →Go to C224

C222. How much did you pay [name selected in C218] in the past one month? (unit: 10,000 Korean won)
____________MW [range:1~997] →C224
Don't know →Go to C218
Refuse to answer →Go to C218

Logic
■ If there is a loop total of C222, then go to C223.

C223. You have said that you paid [C222] MW (10,000 Korean won) to your helpers in the past one month. How much did you pay out-of-pocket? Also, how much did your relatives or private health insurances pay on behalf of you? Please include in the private health insurance category any payments that you made and were reimbursed by private health insurances. (unit: 10,000 Korean won) Categories are ① out-of-pocket payments (C223a), ② payments by relatives including children and parents (C223b), ③ payments reimbursed by private health insurances (C223c).

[IWER: The sum of all items must be equal to [C222] MW.]

ITEMS		AMOUNT
C223a	Out-of-pocket payments	
C223b	Payments by relatives including children and parents	
C223c	Payments reimbursed by private health insurances	
Total		[C222] MW

C224. Who is likely to help you if you need help with activities of daily living, such as eating, dressing, or using the bathroom, over a long period of time in the future? Please select all that apply.

① Spouse

② Parents

③ Son

④ Daughter

⑤ Son-in-law/daughter-in-law

⑥ Sibling

⑦ Grandchildren

⑧ Others

⑨ Voluntary worker

⑩ Publicly provided helper

⑪ Personally hired helper

⑫ No helper

C225. [IWER: How often did R receive assistance in answering section C2-FUNCTIONAL LIMITATIONS AND HELPERS?]

① Never →Go to section C3.

② A few times →Go to section C3.

③ Most or all of the time →Go to section C3.

④ The section was done by a proxy respondent.

C226. What is the proxy's relationship to R? If unknown, please ask the proxy. What is your relationship to R?

① Spouse

② Mother

③ Father

④ Mother-in-law

⑤ Father-in-law

⑥ Brother/sister

⑦ Brother-in-law/sister-in-law

⑧ Son/daughter

⑨ Son–in–law/Daughter–in–law

⑩ Grandchild

⑪ Other relatives

⑫ Helper or other non-relatives

→Go to section C3.

C3. HEALTH INSURANCE AND SERVICES

- Health insurance

C301. The next questions are about health insurance, both public and private. First, in public insurance, there are two types of public health insurance: National Health Insurance and Medical Aid. The National Health Insurance System is a public health insurance system for all Koreans residing in the Korean territory. The Medical Aid System is a public health insurance system for the awardees of the National Merit Award and other honorary people as well as people with low incomes. Are you currently covered by the National Health Insurance System or the Medical Aid System?

① National Health Insurance System (current employer/ community)

⑤ Medical Aid System (Type 1/Type 2)→Go to C309

Don't know →Go to C310

Refuse to answer →Go to C310

C302. There are two ways to enroll in the National Health Insurance System: through the current employer or community. How did you enroll in NHI, through your current employer or community?

① Through (R's or R's family's) current employer

⑤ Through the community →Go to 304

C303. If you are insured through your employer, are you the insured person or a dependent family member?

① Insured person →Go to 305

⑤ Dependent family member →Go to 305

C304. If you are enrolled through the community, are you the household head?

① Yes

⑤ No

C305. Who is currently paying your health insurance premiums?

① You

② Spouse

③ Son/daughter

④ Son–in–law/daughter-in-law

⑤ Other relatives

C306. What is the average monthly amount of insurance premiums paid into the National Health Insurance System? (unit: 10,000 Korean Won)
[IWER: less than ten thousands won = “ten thousands won”]

_____MW [range:1~120]

C307. Have you, or the household member who is currently paying your insurance premiums, failed to pay health insurance premiums?
① Yes
⑤ No →Go to C310
Don’t know →Go to C310
Refuse to answer →Go to C310

C308. How many months have you, or the household member who is currently paying your insurance premiums, failed to pay health insurance premiums? (unit : a month)
______Months [range:1~203] → Go to C310
Don’t know →Go to C310
Refuse to answer →Go to C310

C309. Is it Type 1 Medical Aid or Type 2 Medical Aid?
① Type 1
⑤ Type 2

C310. Are you covered by any private health insurance to compensate for hospital expenses? Private health insurance means every health insurance, regardless of its type, even including those whose term expired. Please exclude health-related special policy conditions included in pension insurance or whole life insurance.
① Yes
⑤ No →Go to C313

C311. How many private health insurances do you have? Please tell me only those with you as a beneficiary.
______ [range:1~30]

C312. What is the total amount of insurance premiums you pay each month for these private health insurances? (unit: 10,000 Korean Won)

_____MW [range:0~997]

C313. Have you received the basic medical checkup covered by the National Health Insurance System or the Medical Aid System in the past two years?

① Yes →Go to C316

⑤ No

C314. Why did you not receive the medical checkup?

① Wanted to receive the medical checkup, but unable to because I had trouble getting around →Go to C317

② Did not expect any problem, as I was fine in the previous checkup →Go to C317

③ Too busy, could not have time →Go to C317

④ I could not trust the results →Go to C317

⑤ I was afraid of the results →Go to C317

⑥ Even if an illness is detected, no adequate treatment is available →Go to C317

⑦ Did not see a need →Go to C317

⑧ I did not know it is free →Go to C317

⑨ Others

C315. If others, please specify.

⑨ ____________________ →Go to C317

C316. Have you ever received any further medical checkup covered by the National Health Insurance System or the Medical Aid System due to problems found in the basic medical checkup?

① Yes

⑤ No

C317. Have you had any other medical checkup using your out-of-pocket money in the past two years?

① Yes

⑤ No

- Hospitalization

Logic

■ If C318=0, then go to C330.

C318. The following questions are about the health care services you have received since the previous interview. Were you ever hospitalized at a hospital, nursing hospital, convalescence clinic, or any other long-term health care facilities after the previous interview? If so, how many times were you hospitalized? (unit: a time)
[IWER: If R never be hospitalized, mark '0']
______time [range:0~12]

Logic
■ Loop through the following questions as many times as the number entered in C318.

C319. In which health care facility were you hospitalized during your [1st/2nd/..] hospitalization?
① General hospital
③ Government-designated nursing hospital
⑤ Other nursing hospitals

C320. How many days did you stay at [C319]? (unit: a day)
[IWER: For example, enter 180 days for 6 months, and 450 days for 1 year and 3 months.]
_____days [range:1~730]

C321. You have said that you were hospitalized for [C320] days. At that time, how much did you pay for your hospitalization (medical treatment)? (unit: 10,000 Korean won)
_____MW [range:1~9997]
Don't know →Go to C323
Refuse to answer →Go to C323

Logic
■ If there is a loop total of C321, then go to C322.

C322. You have said that you paid [C321] MW (10,000 Korean won) for your [C318] rounds of hospitalization (medical treatment). How much did you pay out-of-pocket? Also, how much did your relatives or private health insurances pay on behalf of you? Please include in the private health insurance category any payments that you made and were reimbursed by private health insurances. (unit: 10,000 Korean won) Categories are ① out-of-pocket payments (C322a), ② payments by relatives including children and parents (C322b), ③ payments reimbursed by private health insurances (C322c).
[IWER: The sum of all items must be equal to [C321] MW.]

ITEMS		AMOUNT
C322a	Out-of-pocket payments	
C322b	Payments by relatives including children and parents	
C322c	Payments reimbursed by private health insurances	
Total		[C321] MW

Logic

■ If C323=(57), then go to C319.

C323. Did anybody take care of you during your [1st/2nd/..] stay at the health care facility? If so, among the following people who took care of you? Please do not include those who simply visited. (Select multiple responses.)

② Spouse →Go to C325

③ Mother →Go to C325

④ Father →Go to C325

⑤ Mother-in-law →Go to C325

⑥ Father-in-law →Go to C325

⑦ ~ ㉖ Children →Go to C325

㉗ ~ ㊻ Sibling →Go to C325

㊼ Brother-in-law/sister-in-law →Go to C325

㊽ Son-in-law/daughter-in-law →Go to C325

㊾ Grandchild →Go to C325

㊿ Other relatives →Go to C325

(52) Voluntary worker →Go to C325

(53) Publicly provided helper →Go to C325

(54) Personally hired helper →Go to C325

(55) Others

(57) No one helped →Go to C319

C324. If others, please specify.

____

Logic

■ Loop through C325~328 as many times as the number of people chosen in C323.

C325. How many days did the caregiver chosen in [C323] take care of you?

[IWER: For example, enter 180 days for 6 months, and 450 days for 1 year and 3 months.]

_____days [range:1~730]

C326. How many hours per day did the caregiver chosen in [C323] take care of you? (unit: a time)

_____hours [range:1~24]

C327. Did you pay [C323] for taking care of you?

① Yes

⑤ No →Go to C325

C328. How much did you pay [C323] for taking care of you? (unit: 10,000 Korean won)

_____MW [range:1~997]

Don't know →Go to C325

Refuse to answer →Go to C325

Logic

■ If there is a loop total of C328, then go to C329.

C329. You have said that you paid [C328] MW (10,000 Korean won) for the caregiver services. How much did you pay out-of-pocket? Also, how much did your relatives or private health insurances pay on behalf of you? Please include in the private health insurance category any payments that you made and were reimbursed by private health insurances. (unit: 10,000 Korean won) Categories are ① out-of-pocket payments (C329a), ② payments by relatives including children and parents (C329b), ③ payments reimbursed by private health insurances (C329c).

[IWER: The sum of all items must be equal to [C328] MW.]

ITEMS		AMOUNT
C329a	Out-of-pocket payments	
C329b	Payments by relatives including children and parents	
C329c	Payments reimbursed by private health insurances	
Total		[C328] MW

- Dental Treatment

Logic

■ If C330=0, then go to C333.

C330. Have you ever received dental care (including denture care) since the previous interview? If so, how many times did you go to a dental hospital/clinic? (unit: a time)

[IWER: Enter 0 if R has never made such a visit.]

_____time [range:0~730]

C331. You have said that you received dental care [C330] times since the previous interview. If so, how much did you pay for your dental care? (unit: 10,000 Korean won)

_____MW [range:1~9997]

Don't know →Go to C333

Refuse to answer →Go to C333

C332. You have said that you paid [C331] MW (10,000 Korean won) for your dental care. How much did you pay out-of-pocket? Also, how much did your relatives or private health insurances pay on behalf of you? Please include in the private health insurance category any payments that you made and were reimbursed by private health insurances. (unit: 10,000 Korean won) Categories are ① out-of-pocket payments (C332a), ② payments by relatives including children and parents (C332b), ③ payments reimbursed by private health insurances (C332c).

[IWER: The sum of all items must be equal to [C331] MW.]

ITEMS		AMOUNT
C332a	Out-of-pocket payments	
C332b	Payments by relatives including children and parents	
C332c	Payments reimbursed by private health insurances	
Total		[C331] MW

- Public Health Clinic

C333. Have you ever visited a public health clinic since the previous interview? If so, how many times did you go to a public health clinic? (unit: a time)

[IWER: Enter 0 if R has never made such a visit.]

_____time [range:0~730]

- Oriental Medicine Clinic

Logic

■ If C334=0, then go to C337

C334. Have you ever visited an oriental medicine clinic since the previous interview? If so, how many times did you go to an oriental medicine clinic? (unit: a time)

[IWER: Enter 0 if R has never made such a visit.]
_____time [range:0~730]

C335. You have said that you went to an oriental medicine clinic [C334] times since the previous interview. If so, how much did you pay for your medical care including the purchase of oriental medicine? (unit: 10,000 Korean won)
_____MW [range:1~9997]
Don't know →Go to C337
Refuse to answer →Go to C337

C336. You have said that you paid [C335] MW (10,000 Korean won) for care at the oriental medicine clinic including the purchase of oriental medicine. How much did you pay out-of-pocket? Also, how much did your relatives or private health insurances pay on behalf of you? Please include in the private health insurance category any payments that you made and were reimbursed by private health insurances. (unit: 10,000 Korean won) Categories are ① out-of-pocket payments (C336a), ② payments by relatives including children and parents (C336b), ③ payments reimbursed by private health insurances (C336c).
[IWER: The sum of all items must be equal to [C335] MW.]

ITEMS		AMOUNT
C336a	Out-of-pocket payments	
C336b	Payments by relatives including children and parents	
C336c	Payments reimbursed by private health insurances	
Total		[C335] MW

- Other Outpatient Medical Treatment

Logic
■ If C337=0, then go to C340.

C337. Except for your visits to oriental medicine clinics, public health clinics and dental clinics, and hospitalization, have you been to a local clinic or hospital as an outpatient or to an emergency room since the previous interview? If so, how many times did you go there? (unit: a time)
[IWER: Enter 0, if R has never made such a visit.]
_____time [range:0~730]

C338. You have said that you went to a local clinic or hospital [C337] times since the previous interview. If so, how much did you pay for your outpatient care? (unit: 10,000 Korean won)

_____MW [range:1~9997]

Don't know →Go to C340

Refuse to answer →Go to C340

C339. You have said that you paid [C338] MW (10,000 Korean won) for your outpatient care. How much did you pay out-of-pocket? Also, how much did your relatives or private health insurances pay on behalf of you? Please include in the private health insurance category any payments that you made and were reimbursed by private health insurances. (unit: 10,000 Korean won) Categories are ① out-of-pocket payments (C339a), ② payments by relatives including children and parents (C339b), ③ payments reimbursed by private health insurances (C339c).

[IWER: The sum of all items must be equal to [C338] MW.]

ITEMS		AMOUNT
C339a	Out-of-pocket payments	
C339b	Payments by relatives including children and parents	
C339c	Payments reimbursed by private health insurances	
Total		[C338] MW

- House Call

Logic

■ If C340=0, then go to C343.

C340. Has a health care provider visited your house to give you medical treatment since the previous interview? If so, how many times did you receive such a house call? (unit: a time)

[IWER: Health care provider refers to nurses, visiting nurse assistants, physical therapists, chemical therapists and oxygen therapists. In other words, caregiver services for the purpose of taking care of patients not of treating them are excluded. If R never received such a visit, then enter 0.]

_____time [range:0~730]

C341. You have said that you received [C340] house calls since the previous interview. How much did you pay for your house calls? (unit: 10,000 Korean won)

_____MW [range:1~9997]

Don't know →Go to C343

Refuse to answer →Go to C343

C342. You have said that you paid [C341] MW (10,000 Korean won) for your house calls. How much did you pay out-of-pocket? Also, how much did your relatives or private health insurances pay on

behalf of you? Please include in the private health insurance category any payments that you made and were reimbursed by private health insurances. (unit: 10,000 Korean won) Categories are ① out-of-pocket payments (C342a), ② payments by relatives including children and parents (C342b), ③ payments reimbursed by private health insurances (C342c).

[IWER: The sum of all items must be equal to [C341] MW.]

ITEMS		AMOUNT
C342a	Out-of-pocket payments	
C342b	Payments by relatives including children and parents	
C342c	Payments reimbursed by private health insurances	
Total		[C341] MW

- Prescription Drug

C343. Have you ever taken prescription medications on a regular basis since the previous interview? (For example, long term medications such as antidiabetic or hypertension medicine)

① Yes

⑤ No →Go to C346

C344. How much did you pay for your regular prescription medications since the previous interview? (unit: 10,000 Korean won)

_____MW [range:1~9997]

Don't know →Go to C346

Refuse to answer →Go to C346

C345. You have said that you paid [C344] MW (10,000 Korean won) for your prescription medications. How much did you pay out-of-pocket? Also, how much did your relatives or private health insurances pay on behalf of you? Please include in the private health insurance category any payments that you made and were reimbursed by private health insurances. (unit: 10,000 Korean won) Categories are ① out-of-pocket payments (C345a), ② payments by relatives including children and parents (C345b), ③ payments reimbursed by private health insurances (C345c).

[IWER: The sum of all items must be equal to [C344] MW.]

ITEMS		AMOUNT
C345a	Out-of-pocket payments	
C345b	Payments by relatives including children and parents	
C345c	Payments reimbursed by private health insurances	
Total		[C344] MW

- Other Medical Assistance Equipment

C346. Have you ever purchased (leased) any medical assistance equipment since the previous interview? For example, it refers to medical assistance equipment for treatment purposes, such as hearing aids or wheelchairs.

① Yes

⑤ No →Go to C349

C347. How much did you pay for purchasing (leasing) the medical assistance equipment since the previous interview? (unit: 10,000 Korean won)

_____MW [range:1~9997]

Don't know →Go to C349

Refuse to answer →Go to C349

C348. You have said that you paid [C347] MW (10,000 Korean won) for purchasing (leasing) the medical assistance equipment. How much did you pay out-of-pocket? Also, how much did your relatives or private health insurances pay on behalf of you? Please include in the private health insurance category any payments that you made and were reimbursed by private health insurances. (unit: 10,000 Korean won) Categories are ① out-of-pocket payments (C348a), ② payments by relatives including children and parents (C348b), ③ payments reimbursed by private health insurances (C348c).

[IWER: The sum of all items must be equal to [C347] MW.]

ITEMS		AMOUNT
C348a	Out-of-pocket payments	
C348b	Payments by relatives including children and parents	
C348c	Payments reimbursed by private health insurances	
Total		[C347] MW

C349. [IWER: How often did R receive assistance in answering section C3–HEALTH INSURANCE AND SERVICES?]

① Never →Go to section C4.

② A few times →Go to section C4.

③ Most or all of the time →Go to section C4.

④ The section was done by a proxy respondent.

C350. What is the proxy's relationship to R? If unknown, please ask the proxy. What is your relationship to R?

① Spouse

② Mother

③ Father

④ Mother-in-law

⑤ Father-in-law

⑥ Brother/sister

⑦ Brother-in-law/sister-in-law

⑧ Son/daughter

⑨ Son–in–law/Daughter–in–law

⑩ Grandchild

⑪ Other relatives

⑫ Helper or other non-relatives

→Go to section C4.

C4. COGNITION

C450. Before we go on to the next section, I would like to check if you are a proxy respondent. Are you [R's name]?

① Yes

⑤ No → Go to C451

C401. Now I'm going to ask several simple questions. Some may be easy and some may be hard to answer. Please try to answer as honestly as you can. Are you ready? Please tell me today's date. [IWER: R doesn't have to answer in this order. If R is an elderly person and says the date according to the lunar calendar and it matches with the solar calendar, that date is considered correct. Check the accuracy, using the converter.]

① One of the month / day / year combination is correct

② Two of the month / day / year combination are correct

③ All of the month / day / year combination are correct

⑤ All of the month / day / year combination are incorrect

C402. What day of the week is it today? Is it Monday, Tuesday, Wednesday, Thursday, Friday, Saturday, or Sunday?

① Correct

⑤ Incorrect

C403. What is the current season (among Spring, Summer, Fall, or Winter)?

① Correct

⑤ Incorrect

C404. What is this place used for?

[IWER: If R gives a specific description of the place, such as my home, family house, general house, senior citizens' center, hospital, a place to live, a place to treat sick people, oo apartment, oo village, his/her answer is considered correct.]

① Correct

⑤ Incorrect

C405. What is your address? Please tell me the city, Gun, Dong, APT #/Street Number.

[IWER: It is possible to repeat the question if R has missed one of the four components. It is also OK

to ask a question containing the name of the present location (e.g. Is here Busan/Gyeongsang-do?).]
① One of the city / gun / dong / street address combination is correct
② Two of the city / gun / dong / street address combination are correct
③ Three of the city / gun / dong / street address combination are correct
④ All of the city / gun / dong / street address combination are correct
⑤ All of the city / gun / dong / street address combination are incorrect

Logic
■ Lists are shown randomly.
LIST 1: airplane, pencil, pine tree
LIST 2: snowman, chair, peach
LIST 3: teacher, factory, washing machine
LIST 4: chopsticks, apple, hanger
LIST 5: bat, shoes, sea water

C406. Please listen carefully as I read three words because I cannot repeat them. When I finish, I will ask you to recall aloud as many of the words as you can, in any order. Are you ready?
[IWER: ① Tidy up the surroundings and start. ② Assigned words are displayed only to the IWER. ③ Read the items at a slow and steady pace and stop for one second before reading the next word ④ If R is unable to recall all three words or can recall only a few words, repeat the words up to 5 times including the first try until he/she can recall all of them. ⑤ Stop the process even after the R can't recall the words after 5 tries. ⑥ Answers given by repetition should be treated as incorrect ones.]
① Only one of the three words is correct, in any order
② Two of the three words are correct, in any order
③ All of the three words are correct, in any order
⑤ None of the three words is correct

C407. Try to remember the words I just read to you. I'll ask you to recall them later. [Read once more if R did not recall any of the words, up to 3 times and go on. If R does not recall, reassure him/her that it is OK to make him/her feel comfortable.] Let's do some subtraction this time. What are 100 minus 7?
[IWER: If R is unable to solve complex calculations, mark '0 (incorrect answer)' for all of questions C407 ~ C411. Instead, ask simple questions such as "What is 100 minus 30?" or "What is 10 minus 3?" for about 3 minutes, but no longer.]
____(Mark the number what the R said)

C408. And 7 from that?

[IWER: Try to persuade if R refuses to answer. Record the exact number R says. Record 0 in case of 'don't know' or 'refuse to answer'.]

____(Mark the number what the R said)

C409. And 7 from that?

[IWER: Try to persuade if R refuses to answer. Record the exact number R says. Record 0 in case of 'don't know' or 'refuse to answer'.]

____(Mark the number what the R said)

C410. And 7 from that?

[IWER: Try to persuade if R refuses to answer. Record the exact number R says. Record 0 in case of 'don't know'or 'refuse to answer'.]

____(Mark the number what the R said)

C411. And 7 from that?

[IWER: Try to persuade if R refuses to answer. Record the exact number R says. Record 0 in case of 'don't know'or 'refuse to answer'.]

____(Mark the number what the R said)

Logic

■ The words shown in C406 are displayed again for the IWER. They must be the same words as before.

C412. A little while ago, I read you a list of words and you repeated the ones you could remember. Please tell me any of the words that you remember now.

① Only one of the three words is correct, in any order

② Two of the three words are correct, in any order

③ All of the three words are correct, in any order

⑤ None of the three words is correct

C413. [IWER: Pointing to item #1] What is this?

[IWER: The item can be anything within close reach, such as a cell phone, gloves, a hat and a ring. The elderly tend to call all types of writing instruments 'pencil', so consider the answer correct if they say 'pencil' instead of 'pen'.]

① Correct

⑤ Incorrect

C414. [IWER: Pointing to item #2] What is this?

[IWER: If R calls a watch “clock”, the answer is considered correct.]

① Correct

⑤ Incorrect

Logic

■ Lists are shown randomly.

Sentence 1: To see is to believe

Sentence 2: Check the dead fire again

Sentence 3: The Great Mountain is high but under the sky

Sentence 4: God watch over our land forever

Sentence 5: Thousand miles of range and river land

C415. Now you have to exactly repeat what I say. Are you ready? Please listen carefully.

[IWER: ① Tidy up the surroundings. ② Read the sentences aloud at a slow and steady pace. ③ Mark 'Incorrect' if the pronunciation is unclear, except when R has no teeth or problems in oral structure. In principle, no point should be given if R has failed on the first try but succeeded on the second try. However, if R is deemed to have been distracted on the first try, the IWER may get him/her to pay attention and repeat the question. Mark 'Correct' if the right answer is given.]

① Correct

⑤ Incorrect

C416. [IWER: holds one A4 paper.] Now, listen carefully and follow my directions. Are you ready? When I give you a piece of paper, please turn it over, fold it in half, and give it back to me.

[IWER:

① Tidy up the surroundings (especially the front) so it doesn't interfere with R.

② Do not repeat the question in the middle of the process.

③ Do not give out the paper in advance.

④ Directions can be repeated if R seems unable to understand the directions or if R asks you to repeat the directions. In this case, the paper given out should be collected and the process should start over.]

① One of the turning / folding / returning actions is completed successfully

② Two of the turning / folding / returning actions are completed successfully

③ All of the turning / folding / returning actions are completed successfully

⑤ None of the turning / folding / returning actions are completed successfully

C417. [IWER: Make sure R doesn't see the test paper with “close your eyes” written on it before asking the question. Make sure that someone does not read the sentence to R.] I will show you a sentence. Please read the sentence aloud and act it out.

① R completed only one task (either reading or action of closing eyes)

③ R did both tasks

⑤ R did not complete any task

C418. [IWER: Give R a pen and point to the blank part of the paper.] Please write one sentence about how you’re feeling today or about today’s weather.

① Wrote a sentence

⑤ Couldn’t write a sentence

C419. [IWER: Show the picture of two pentagons overlapped.] Do you see this picture? Please draw that picture on this paper.

① Drew the picture

⑤ Failed to draw the picture

C420. [IWER: How often did R receive assistance in answering section C4-COGNITION?]

① never →Go to section C5.

② a few times →Go to section C5.

③ most or all of the time →Go to section C5.

C451. [IWER: Section C4-COGNITION cannot be done by a proxy respondent.]

① CONTINUE →Go to section C5.

C5. GRIP STRENGTH

C550. Before we go on to the next section, I would like to check if you are a proxy respondent. Are you [R's name]?

① Yes

⑤ No →Go to C551

C501. Now I would like to measure the strength of your hand in a gripping action. Which is your dominant hand?

① Right hand

③ Left hand

⑤ Both hands equally dominant

C502. I will ask you to squeeze this handle as hard as you can, just for a couple of seconds and then let go. I will take alternately two measurements from your right and left hands. Are you willing to do this test?

① Yes

③ Willing but unable to do the test →Go to C505

⑤ No →Go to C505

C503. Before we begin, I'd like to make sure it is safe for you to do this test. Have you had any surgery within the last six months, or experienced any swelling, inflammation, severe pain, or injury in one or both hands that might prevent you from gripping?

① No apparent restriction →Go to C507

③ Yes, recent surgery

⑤ Yes, sever pain

⑦ Yes, swelling or inflammation

⑨ Other problems

C504. Which hand hurts?

① R is unable to use both hands →Go to C505

③ R is unable to use right hand →Go to C509

⑤ R is unable to use left hand →Go to C507

C505. [IWER: Why didn't R complete the grip strength test?] (Select all that apply.)

① R felt it would not be safe →Go to C513

② IWER felt it would not be safe →Go to C513

③ R refused →Go to C513

④ R tried but was unable to complete the test →Go to C513

⑤ R did not understand the instructions →Go to C513

⑥ R had surgery, injury or swelling on both hands in the past six months →Go to C513

⑦ Others

C506. If others, please specify.

____________________ →Go to C513

C507. Now, I'm going to measure your RIGHT HAND. Please put your elbow onto your side and make a right angle around your elbow. Make sure the scale of the dynamometer is set to zero. Ready? Squeeze it now. (unit: Kg)

[IWER: R is asked to take off his/her ring or watch for safety.]

FIRST READING ______ [range: 0~50]

C508. Now, I'm going to measure your RIGHT HAND again. Please set the scale of the dynamometer to zero, Squeeze it now. (unit: Kg.)

SECOND READING _____ [range: 0~50]

Logic

■ If C504=⑤, then go to C511.

C509. Now, I'm going to measure your LEFT HAND, Please put your elbow onto your side and make a right angle around your elbow. Make sure the scale of the dynamometer is set to zero. Ready? Squeeze it now. (unit: Kg.)

[IWER: R is asked to take off his/her ring or watch for safety.]

FIRST READING ______ [range: 0~50]

C510. Now, I'm going to measure your LEFT HAND again, Please set the scale of the dynamometer to zero. Squeeze it now. (unit: Kg.)

SECOND READING _____ [range: 0~50]

C511. [IWER: What was R's position for this test?]

① standing

③ sitting

⑤ lying down

C512. [IWER: Did R receive a support while performing this test?]

① Yes

⑤ No

C513. [IWER: How much effort did R give to this measurement?]

① R gave full effort →Go to section D.

② R was prevented from giving full effort by illness, pain, symptoms or discomfort →Go to section D.

③ R did not appear to give full effort without an obvious reason →Go to section D.

④ R did not measure for a personal reason →Go to section D.

C551. [IWER: Section C5–GRIP STRENGTH cannot be done by a proxy respondent.]

① CONTINUE →Go to section D.

D. EMPLOYMENT

- Current Employment Status

Logic

■ R cannot answer 'Don't know' or' Refuse to answer' to D001.

D001. Are you currently employed for income? Being employed refers to working for an employer, being self-employed, or working for your family or relative's business.

① Yes

⑤ No →Go to D005

Logic

■ R cannot answer 'Don't know' or' Refuse to answer' to D002.

D002. I would like to ask about your current primary job. Which of the following best describes your primary job?

① I am employed by another person or a company and receive wages. →Go to D007 or D008

② I am self-employed. →Go to D007 or D008

③ I help my family / relative's business without pay for 18 hours or more per week. →Go to D007 or D008

④ I help my family / relative's business without pay for less than 18 hours a week.

D003. What is the reason you work less than 18 hours a week without income?

① Health problems →Go to D005

② Childcare or housework →Go to D005

③ Attending school →Go to D005

④ Wanted to take a break →Go to D005

⑤ Not enough work to do →Go to D005

⑥ Others

D004. If others, please specify.

____

Logic

■ R cannot answer 'Don't know' or' Refuse to answer' to D005.

D005. Are you looking for a job?

① Yes

⑤ No

Logic

■ R cannot answer 'Don't know' or' Refuse to answer' to D006.

D006. Which of the following best describes your status with regard to working in the labor market?
[IWER: Retirement refers to having stopped doing income-earning activities and presently not working or engaging only in pastime work, and having no intention of engaging in anything more serious than pastime work as long as there is no special change in circumstances.]

① Never had a job before

③ Currently retired (no intent to work unless circumstances change)

⑤ Intend to work if the wage and working conditions meet my expectations and circumstances are good

⑦ Intend to work if the wage and working conditions meet my expectations

⑨ Intend to work even if the wage and working conditions are slightly below my expectations

- Job history

Logic

■ If R answered in the previous interview that he/she was working, then go to D007. If R answered in the previous interview that he/she was not working, then go to D008.

■ If R answered in the previous interview that he/she was working, then go to D007.

D007. Now I am going to ask you about your job change since the previous interview. In the previous interview, you responded that you were working as a [job title: occupation] at [name of workplace: industry]. Is this correct?

[IWER:

- Moving to another workplace is considered a job change. However, a change of workplace name is excluded.

- All other cases are considered a title/position change within the same job.]

① Yes →(wage workers) →Go to D023, (non-wage worker) →Go to D033

⑤ No →Go to D009

Logic

■ If R answered in the previous interview that he/she was not working, then go to D008.

D008. Now I am going to ask you about your job change since the previous interview. In the previous interview, you responded that you were not working. Is this correct?

① Yes →Go to D043

⑤ No

D009. Please state the work you were doing at the time of the previous interview. What kind of work did you do? (Employment status)

① Employed by another person or a company and received wages

③ Self-employed or unpaid family worker

⑤ Not working at the time of the previous interview →Go to D043

D010. Which industry was the workplace engaged in?

① Agriculture/forestry →Go to D015

② Fishing →Go to D015

③ Mining and quarrying →Go to D015

④ Manufacturing

⑤ Electricity, gas, and water supply →Go to D015

⑥ Construction →Go to D015

⑦ Wholesale and retail trade →Go to D012

⑧ Hotels and restaurants →Go to D013

⑨ Transport →Go to D015

⑩ Post and telecommunications →Go to D015

⑪ Financial and insurance activities →Go to D015

⑫ Real estate and renting and leasing →Go to D015

⑬ Other activities →Go to D014

D011. Specifically speaking, which industrial classification best describes the workplace?

① Manufacture of food products and beverages

② Manufacture of textiles, wearing apparel, luggage, and footwear

③ Manufacture of metal and machinery materials and products

④ Manufacture of electronic and electric materials and products

⑤ Manufacture of motor vehicles and transport equipment products

⑥ Manufacture of other products

→Go to D015

D012. Specifically speaking, which industrial classification best describes the workplace?

① Sale of motor vehicles

② Wholesale

③ Retail trade

→Go to D015

D013. Specifically speaking, which industrial classification best describes the workplace?

① Hotels

② Restaurants

→Go to D015

D014. Specifically speaking, which industrial classification best describes the workplace?

① Business services

② Public administration, defense, and compulsory social security

③ Education

④ Health and social work

⑤ Recreation, culture, and sports related services

⑥ Other community, repair, and personal services

⑦ Housekeeping services

⑧ International organizations

D015. What kind of work did you do in the workplace?

① Managers →Go to D020

② (Associate) Professionals →Go to D020

③ Clerks

④ Service workers →Go to D017

⑤ Sales workers →Go to D020

⑥ Skilled agricultural, forestry, and fishery workers →Go to D018

⑦ Craft and related trades workers →Go to D020

⑧ Equipment, machine operating and assembling workers →Go to D020

⑨ Elementary workers →Go to D019

⑩ Career soldiers →Go to D020

D016. Specifically speaking, which occupational classification best describes your work?

① General office clerks

② Customer services clerks

→Go to D020

D017. Specifically speaking, which occupational classification best describes your work?

① Personal services related workers

② Cooking and food service workers

③ Travel and transport related workers

④ Protective service workers

→Go to D020

D018. Specifically speaking, which occupational classification best describes your work?

① Skilled agricultural and livestock workers

② Skilled forestry workers

③ Skilled fishery workers

→Go to D020

D019. Specifically speaking, which occupational classification best describes your work?

① Housekeeping, cleaning, and laundry workers

② Building management and security related workers

③ Delivery, moving, and meter reading workers

④ Sanitation workers

⑤ Other services related elementary workers

⑥ Agriculture, forestry, and fishery related elementary workers

⑦ Manufacturing related elementary workers

⑧ Mining and construction related elementary workers

⑨ Transport related elementary workers

Logic

■ If D009=①, then go to D020~D022.

D020. What was your official position or job title?

[IWER: Mark 0 if there is no official position or title.]

⓪ No position or job title

① Staff member

② Assistant foreman

③ Foreman

④ Assistant manager

⑤ Manager

⑥ Deputy general manager

⑦ General manager

⑧ Board member

D021. What was your employment status?

* POINT *
- Regular worker: Workers with an employment contract of more than one year or cases in which one may choose to work as long as one likes without a predefined contract period<br>- Temporary worker: Workers with an employment contract of more than one month and less than one year or cases in which the work is expected to be completed within one year, although there is no predefined period of the employment contract (Yet, those who work in a company for a long time and/or expect to work continuously in the future, but have a contract of less than one year are considered as temporary workers.)<br>- Casual worker: Workers with an employment contract of less than one month, who are hired and paid on a daily basis, or compensated for the work done in various locations without a designated workplace

① Regular worker

③ Temporary worker

⑤ Casual worker

D022. Did you work part time or full time?

* POINT *
- Part time: Cases in which one works part time or as a side job, works less hours than other employees who perform the same job, or is paid on an hourly basis<br>- Full time: Cases in which one works full working days as opposed to part time

① Part time

⑤ Full time

Logic

■ If R was identified as a wage worker in the previous interview or D009=①, then go to D023~D032, D701~D704, D711~D717.

■ If R was identified as a self-employed/unpaid family worker in the previous interview or D009=③, then go to D033~D035, D711~D717.

Logic

■ If R was identified as a wage worker in the previous interview or D009=①, then go to D23.

■ If D024=⑤, then go to D027.

Logic

■ (As loop=1) If D007=①, then go to D023.

D023. (As loop=1)You responded in the previous interview that your position or job title was ______. Please select a position/job title that best describes ______ from the following. (workplace in the previous interview: [name of workplace, job title])

⓪ No position or job title

① Staff member

② Assistant foreman

③ Foreman

④ Assistant manager

⑤ Manager

⑥ Deputy general manager

⑦ General manager

⑧ Board member

D024. (As loop=1) Did your position or job title change within the workplace since the previous interview? (Workplace in the previous interview: [name of workplace, job title])
(As loop>=2) Did your position or job title change again after that? (workplace in the previous interview (2006/2008): [name of workplace, job title])
① Yes
⑤ No →Go to D027

D025. What was your new position or job title? (Workplace in the previous interview: [name of workplace, job title])
⓪ No position or job title
① Staff member
② Assistant foreman
③ Foreman
④ Assistant manager
⑤ Manager

⑥ Deputy general manager

⑦ General manager

⑧ Board member

D026. When were you appointed to the new position or title? (unit: year and month combined into 6 digits, workplace in the previous interview: [name of workplace, job title])
[IWER: Enter the year and month using 6 digits. For example, mark 200701 for January 2007. If the month is not clear, enter 200700.]

____

Logic
■ If D027=⑤, then go to D030.

D027. (As loop=1)You responded in the previous interview that your employment status was ______. Did your employment status change within the workplace since then? (Workplace in the previous interview: [name of workplace, job title])
(As loop>=2) Did your employment status change again after that? (Workplace in the previous interview: [name of workplace, job title])
① Yes
⑤ No →Go to D030

D028. What was your new employment status? (Workplace in the previous interview: [name of workplace, job title])

* POINT*
- Regular worker: Workers with an employment contract of more than one year or cases in which one may choose to work as long as one likes without a predefined contract period<br>- Temporary worker: Workers with an employment contract of more than one month and less than one year or cases in which the work is expected to be completed within one year, although there is no predefined period of the employment contract (Yet, those who work in a company for a long time and/or expect to work continuously in the future, but have a contract of less than one year are considered as temporary workers)<br>- Casual worker: Workers with an employment contract of less than one month, who are hired and paid on a daily basis, or compensated for the work done in various locations without a designated workplace

① Regular worker

③ Temporary worker

⑤ Casual worker

D029. When did your employment status change? (unit: year and month combined into 6 digits, workplace in the previous interview: [name of workplace, job title])
[IWER: Enter the year and month using 6 digits. For example, mark 200701 for January 2007. If the month is not clear, enter 200700.]

____

Logic
■ If D030=⑤, then go to D701.

D030. (As loop=1)You responded in the previous interview that your working hours was _______. Did your working hours change within the workplace since then? (Workplace in the previous interview: [name of workplace, job title])
(As loop>=2) Did your working hours change again after that? (Workplace in the previous interview: [name of workplace, job title])
① Yes
⑤ No →Go to D701

D031. Did you work part time or full time? (Workplace in the previous interview: [name of workplace, job title])

* POINT *
- Part time: Cases in which one works part time or as a side job, works less hours than other employees who perform the same job, or is paid on an hourly basis
- Full time: Cases in which one works full working days as opposed to part time

① Part time

⑤ Full time

D032. When did your working hours change? (unit: year and month combined into 6 digits, workplace in the previous interview: [name of workplace, job title])
[IWER: Enter the year and month using 6 digits. For example, mark 200701 for January 2007. If the month is not clear, enter 200700.]

____

Logic

■ If D701=⑤, then go to D711.

D701. (As loop=1) Did you move to a new location within the same workplace since the previous interview? (Workplace in the previous interview): [name of workplace, job title])

(As loop>=2) Did you move again to a new location after that? (Workplace in the previous interview: [name of workplace, job title])

① Yes

⑤ No →Go to D711

D702. When did you move to a new location? (unit: year and month combined into 6 digits, workplace in the previous interview: [name of workplace, job title])

[IWER: Enter the year and month using 6 digits. For example, mark 200701 for January 2007. If the month is not clear, enter 200700.]

____

D703. How many employees worked in the new location including yourself? (unit: a person)

__________People [range:1~9997]

D704. Then what is the closest approximation to the actual number of employees that worked in the new location?

① Fewer than 5 people

② 5 to 9 people

③ 10 to 29 people

④ 30 to 49 people

⑤ 50 to 99 people

⑥ 100 to 299 people

⑦ 300 to 499 people

⑧ 500 to 999 people

⑨ More than 1,000 people

Logic

■ If R was identified as a self-employed/unpaid family worker in the previous interview, then go to D033.

■ If D033=⑤, then go to D711.

D033. (As loop=1) You responded in the previous interview that your employment status was ______. Did your employment status change within the workplace since then? (Workplace in the previous interview): [name of workplace, job title])
(As loop>=2) Did your employment status change again after that? (Workplace in the previous interview): [name of workplace, job title])
[IWER: Change in employment status means a change from "self-employed to unpaid family worker" or a change from "unpaid family worker to self-employed (employer)".]
① Yes
⑤ No →Go to D711

D034. What was your new employment status? (Workplace in the previous interview: [name of workplace, job title])
① Self-employed (employer) to unpaid family worker
⑤ Unpaid family worker to self-employed (employer)

D035. When did your employment status change? (unit: year and month combined into 6 digits, workplace in the previous interview: [name of workplace, job title])
[IWER: Enter the year and month using 6 digits. For example, mark 200701 for January 2007. If the month is not clear, enter 200700.]

____

Logic
■ If D711=⑤, then go to D036.

D711. (As loop=1) You responded in the previous interview that the kind of work you did in the workplace was ___. Did your work change within the workplace since then? (Workplace in the previous interview): [name of workplace, job title])
(As loop>=2) Did your work change again after that? (Workplace in the previous interview): [name of workplace, job title])
[IWER: Only changes between major occupational groups are considered work changes.]
① Yes
⑤ No →Go to D036

D712. What was your new work? (Workplace in the previous interview): [name of workplace, job title])
[IWER: Use a memo function to write on detail contents]
① Managers →Go to D0717
② (Associate) Professionals →Go to D0717

③ Clerks →Go to D0713

④ Service workers →Go to D0714

⑤ Sales workers →Go to D0717

⑥ Skilled agricultural, forestry, and fishery workers →Go to D0715

⑦ Craft and related trades workers →Go to D0717

⑧ Equipment, machine operating and assembling workers →Go to D0717

⑨ Elementary workers →Go to D0716

⑩ Career soldiers →Go to D0717

D713. Specifically speaking, which occupational classification best describes your new work?

[IWER: Use a memo function to write on detail contents]

① General office clerks

② Customer services clerks

D714. Specifically speaking, which occupational classification best describes your new work?

[IWER: Use a memo function to write on detail contents]

① Personal services related workers

② Cooking and food service workers

③ Travel and transport related workers

④ Protective service workers

D715. Specifically speaking, which occupational classification best describes your new work?

[IWER: Use a memo function to write on detail contents]

① Skilled agricultural and livestock workers

② Skilled forestry workers

③ Skilled fishery workers

D716. Specifically speaking, which occupational classification best describes your new work?

[IWER: Use a memo function to write on detail contents]

① Housekeeping, cleaning, and laundry workers

② Building management and security related workers

③ Delivery, moving, and meter reading workers

④ Sanitation workers

⑤ Other services related elementary workers

⑥ Agriculture, forestry, and fishery related elementary workers

⑦ Manufacturing related elementary workers

⑧ Mining and construction related elementary workers

⑨ Transport related elementary workers

D717. When did your work change? (unit: year and month combined into 6 digits, workplace in the previous interview): [name of workplace, job title])
[IWER: Enter the year and month using 6 digits. For example, mark 200701 for January 2007. If the month is not clear, enter 200700.]

____

Logic
■ R cannot answer 'Don't know' or' Refuse to answer' to D0036.

D036. Are you currently working in the workplace you responded you were working in the previous interview? (workplace in the previous interview): [name of workplace, job title])
[IWER: If R moved to another workplace, he/she is considered having a different job.]
① Yes →Go to D085
⑤ No

D037. When did you quit this job? (unit: year and month combined into 6 digits)
[IWER: Enter the year and month using 6 digits. For example, mark 200701 for January 2007. If the month is not clear, enter 200700.]
___________________ [range:200600 ~ 200812]

D038. Why did you quit?
(in the case of wage workers)
① Bankruptcy, closure, temporary shutdown, etc. of business
② Layoff, dehiring, voluntary early retirement, etc.
③ Contract expired
④ Retirement (at the mandatory retirement age)
⑤ Too low wage
⑥ Work was temporary or did not have a good prospect
⑦ Aptitude, knowledge, and skills did not match well
⑧ Long working hours/unfavorable working conditions
⑩ Marriage, childbirth, childcare, etc.
⑪ Care for sick family member
⑪ Found a better job
⑫ To start up a new business

⑬ Poor health

⑭ Wanted to take a break

⑮ Others

(in the case of non-wage worker)

① Business was slow

② Work did not have a good prospect

③ Aptitude, knowledge, and skills did not match well

④ Long working hours/unfavorable working conditions

⑤ Marriage, childbirth, childcare, etc.

⑥ Moved house

⑦ Care for sick family member

⑧ Found a paid job

⑨ To start up a new business

⑩ For unpaid assistance in family business

⑪ Poor health

⑫ Wanted to take a break

⑬ Others

Logic

■ If R was identified as a wage worker in the previous interview or D009=①, then go to D039~D042.

D039. Did you receive or expect to receive legal retirement benefits (or benefits based on collective bargaining agreement, employment rules, etc.) when you quit this job?

① Received or expected to receive

⑤ Not applicable →Go to D041

D040. How much did you receive? (unit: 10,000 Korean won)

___MW [range:0~9997]

D041. Did you receive any payments other than legal retirement benefits (for example, early retirement compensation or retirement condolence payments)?

① Yes

⑤ No →Go to D043

D042. How much did you receive? (unit: 10,000 Korean won)

___MW [range:0~9997]

Logic

■ R cannot answer ‘Don’t know’ or’ Refuse to answer’ to D043.

■ If D008=① or D036=⑤, then go to D043.

D043. Did you find a new job since the previous interview?

① Yes

⑤ No →Go to D085

Logic

■ Until D081=① or D084=⑤, loop D044~D084.

D044. Did the new job involve being employed by another person and receiving wages? Or did you do your own business or assist your family business? (Employment status)

[IWER: “Please list your jobs in the order in which you have changed them since the previous interview. For example, if you were a wage worker between March and September 2007, and then became an unpaid family worker from October 2007, you should list wage worker → unpaid family worker.”]

① Employed by another person or a company and received wage

⑤ Self-employed or unpaid family worker

D045. When did you start this job? (unit: year and month combined into 6 digits)

[IWER: Enter the year and month using 6 digits. For example, mark 200701 for January 2007. If the month is not clear, enter 200700.]

___________________ [range:200600 ~ 200812]

D046. Which industry was the workplace engaged in?

[IWER: Use a memo function to write on detail contents]

① Agriculture/forestry →Go to D051

② Fishing →Go to D051

③ Mining and quarrying →Go to D051

④ Manufacturing

⑤ Electricity, gas, and water supply →Go to D051

⑥ Construction →Go to D051

⑦ Wholesale and retail trade →Go to D048

⑧ Hotels and restaurants →Go to D049

⑨ Transport →Go to D051

⑩ Post and telecommunications →Go to D051

⑪ Financial and insurance activities →Go to D051

⑫ Real estate and renting and leasing →Go to D051

⑬ Other activities →Go to D050

D047. Specifically speaking, which industrial classification best describes the workplace?

[IWER: Use a memo function to write on detail contents]

① Manufacture of food products and beverages

② Manufacture of textiles, wearing apparel, luggage, and footwear

③ Manufacture of metal and machinery materials and products

④ Manufacture of electronic and electric materials and products

⑤ Manufacture of motor vehicles and transport equipment products

⑥ Manufacture of other products

→Go to D051

D048. Specifically speaking, which industrial classification best describes the workplace?

[IWER: Use a memo function to write on detail contents]

① Sale of motor vehicles

② Wholesale

③ Retail trade

→Go to D051

D049. Specifically speaking, which industrial classification best describes the workplace?

[IWER: Use a memo function to write on detail contents]

① Lodging industry

② Restaurants

→Go to D051

D050. Specifically speaking, which industrial classification best describes the workplace?

[IWER: Use a memo function to write on detail contents]

① Business services

② Public administration, defense, and compulsory social security

③ Education

④ Health and social work

⑤ Recreation, culture, and sports related services

⑥ Other community, repair, and personal services

⑦ Housekeeping services

⑧ International organizations

→Go to D051

D051. What kind of work did you do in the workplace?

[IWER: Use a memo function to write on detail contents]

① Managers →(wage workers) Go to D056, (non-wage worker) Go to D071

② (Associate) Professionals →(wage workers) Go to D056, (non-wage worker) Go to D071

③ Clerks

④ Service workers →Go to D053

⑤ Sales workers →(wage workers) Go to D056, (non-wage worker) Go to D071

⑥ Skilled agricultural, forestry, and fishery workers →Go to D054

⑦ Craft and related trades workers →(wage workers) Go to D056, (non-wage worker) Go to D071

⑧ Equipment, machine operating and assembling workers →(wage workers) Go to D056, (non-wage worker) Go to D071

⑨ Elementary workers →Go to D055

⑩ Career soldiers →(wage workers) Go to D056, (non-wage worker) Go to D071

D052. Specifically speaking, which occupational classification best describes your job?

[IWER: Use a memo function to write on detail contents]

① General office clerks

② Customer services clerks

→(wage workers) Go to D056, (non-wage worker) Go to D071

D053. Specifically speaking, which occupational classification best describes your job?

[IWER: Use a memo function to write on detail contents]

① Personal services related workers

② Cooking and food service workers

③ Travel and transport related workers

④ Protective service workers

→(wage workers) Go to D056, (non-wage worker) Go to D071

D054. Specifically speaking, which occupational classification best describes your job?

[IWER: Use a memo function to write on detail contents]

① Skilled agricultural and livestock workers

② Skilled forestry workers

③ Skilled fishery workers
→(wage workers) Go to D056, (non-wage worker) Go to D071

D055. Specifically speaking, which occupational classification best describes your job?
[IWER: Use a memo function to write on detail contents]
① Housekeeping, cleaning, and laundry workers
② Building management and security related workers
③ Delivery, moving, and meter reading workers
④ Sanitation workers
⑤ Other services related elementary workers
⑥ Agriculture, forestry, and fishery related elementary workers
⑦ Manufacturing related elementary workers
⑧ Mining and construction related elementary workers
⑨ Transport related elementary workers
→(wage workers) Go to D056, (non-wage worker) Go to D071

Logic
■ If D044=①(wage workers), then go to D056~D070, D731~D734, D741~D747.

D056. How many employees worked at all locations of the workplace including the head office, branch offices, branches, plants, and the field? (unit: a person, workplace since [D045]: [Industry D046] [Occupation D051])
_____________people [range:1~9997] →Go to D721

D057. Then what is the closest approximation to the actual number of employees that worked at all locations of the workplace including the head office, branch offices, branches, plants, and the field? (unit: number of people, workplace since [D045]: [Industry D046] [Occupation D051])
① Fewer than 5 people
② 5 to 9 people
③ 10 to 29 people
④ 30 to 49 people
⑤ 50 to 99 people
⑥ 100 to 299 people
⑦ 300 to 499 people
⑧ 500 to 999 people
⑨ More than 1,000 people

D721. How many employees worked at the location where you worked?

_____people [range:1~9997] →Go to D058

D722. Then what is the closest approximation to the actual number of employees that worked at the location where you worked?

① Fewer than 5 people

② 5 to 9 people

③ 10 to 29 people

④ 30 to 49 people

⑤ 50 to 99 people

⑥ 100 to 299 people

⑦ 300 to 499 people

⑧ 500 to 999 people

⑨ More than 1,000 people

D058. What was your position or job title at the workplace? (Workplace since [D045]: [Industry D046] [Occupation D051])

⓪ No position or job title

① Staff member

② Assistant foreman

③ Foreman

④ Assistant manager

⑤ Manager

⑥ Deputy general manager

⑦ General manager

⑧ Board member

D059. What was your employment status? (workplace since [D045]: [Industry D046] [Occupation D051])

* POINT*

- Regular worker: Workers with an employment contract of more than one year or cases in which one may choose to work as long as one likes without a predefined contract period
- Temporary worker: Workers with an employment contract of more than one month and less than one year or cases in which the work is expected to be completed within one year, although there is no predefined period of the employment contract (Yet, those who work in a company for a long time and/or expect to work continuously in the future, but have a contract of less than one year are considered as temporary workers.)
- Casual worker: Workers with an employment contract of less than one month, who are hired and paid on a daily basis, or compensated for the work done in various locations without a designated workplace

① Regular worker

③ Temporary worker

⑤ Casual worker

D060. Did you work part time or full time? (workplace since [D045]: [Industry D046] [Occupation D051])

* POINT*

- Part time: Cases in which one works part time or as a side job, works less hours than other employees who perform the same job, or is paid on an hourly basis
- Full time: Cases in which one works full working days as opposed to part time

① Part time

⑤ Full time

D061. Did your position / job title, employment status, working hours, work, or location change at this workplace? (Workplace since [D045]: [Industry D046] [Occupation D051])

[IWER: Enter 'Yes' if one of the following changed: job position/title, employment status, working hours, work or location.]

① Yes

⑤ No →Go to D081

Logic

■ If D062=⑤, then go to D065.

D062. (As loop=1) You have responded that your position or job title was [D058]. Did your position or

job title change since then? (workplace since [D045]: [Industry D046] [Occupation D051])
(As loop>=2) Did your position or job title change again after that? (workplace since [D045]: [Industry D046] [Occupation D051])
① Yes
⑤ No →Go to D065

D063. What was your new position or job title? (workplace since [D045]: [Industry D046] [Occupation D051])
⓪ No position or job title
① Staff member
② Assistant foreman
③ Foreman
④ Assistant manager
⑤ Manager
⑥ Deputy general manager
⑦ General manager
⑧ Board member

D064. When were you appointed to the new position or title? (unit: year and month combined into 6 digits, workplace since [D045]: [Industry D046] [Occupation D051])
[IWER: Enter the year and month using 6 digits. For example, mark 200701 for January 2007. If the month is not clear, enter 200700.]

____

Logic
■ If D065=⑤, then go to D068.

D065. (As loop=1) You have responded that your employment status was [D059]. Did your employment status change within the workplace since then? (workplace since [D045]: [Industry D046] [Occupation D051])
(As loop>=2) Did your employment status change again after that? (workplace since [D045]: [Industry D046] [Occupation D051])
① Yes
⑤ No →Go to D068

D066. What was your new employment status? (workplace since [D045]: [Industry D046] [Occupation D051])

* POINT*
- Regular worker: Workers with an employment contract of more than one year or cases in which one may choose to work as long as one likes without a predefined contract period
- Temporary worker: Workers with an employment contract of more than one month and less than one year or cases in which the work is expected to be completed within one year, although there is no predefined period of the employment contract (Yet, those who work in a company for a long time and/or expect to work continuously in the future, but have a contract of less than one year are considered as temporary workers.)
- Casual worker: Workers with an employment contract of less than one month, who are hired and paid on a daily basis, or compensated for the work done in various locations without a designated workplace

① Regular worker
③ Temporary worker
⑤ Casual worker

D067. When did your employment status change? (unit: year and month combined into 6 digits, workplace since [D045]: [Industry D046] [Occupation D051])
[IWER: Enter the year and month using 6 digits. For example, mark 200701 for January 2007. If the month is not clear, enter 200700.]

____

Logic
■ If D068=⑤, then go to D731.

D068. (As loop=1) You have responded that your working hours was [D060]. Did your working hours change since then? (workplace since [D045]: [Industry D046] [Occupation D051])
(As loop>=2) Did your working hours change again after that? (workplace since [D045]: [Industry D046] [Occupation D051])
① Yes
⑤ No →Go to D731

D069. Did you work part time or full time? (workplace since [D045]: [Industry D046] [Occupation D051])

* POINT*<br>- Part time: Cases in which one works part time or as a side job, works less hours than other employees who perform the same job, or is paid on an hourly basis<br>- Full time: Cases in which one works full working days as opposed to part time

① Part time

⑤ Full time

D070. When did your working hours change? (unit: year and month combined into 6 digits, workplace since [D045]: [Industry D046] [Occupation D051])

[IWER: Enter the year and month using 6 digits. For example, mark 200701 for January 2007. If the month is not clear, enter 200700.]

____

Logic

■ If D731=⑤, then go to D741.

D731. (As loop=1) Did you move to a new location within the same workplace? (Workplace since [D045]: [Industry D046] [Occupation D051])

(As loop>=2) Did you move again to a new location after that? (Workplace since [D045]: [Industry D046] [Occupation D051])

① Yes

⑤ No →Go to D741

D732. When did you move to a new location? (unit: year and month combined into 6 digits, workplace since [D045]: [Industry D046] [Occupation D051])

[IWER: Enter the year and month using 6 digits. For example, mark 200701 for January 2007. If the month is not clear, enter 200700.]

____

D733. How many employees worked in the new location? (unit: a person)

_________people [range:1~9997]

D734. Then what is the closest approximation to the actual number of employees that worked in the new location?

① Fewer than 5 people

② 5 to 9 people

③ 10 to 29 people

④ 30 to 49 people

⑤ 50 to 99 people

⑥ 100 to 299 people

⑦ 300 to 499 people

⑧ 500 to 999 people

⑨ More than 1,000 people

Logic

■ If D044=⑤(non-wage worker), then go to D071~D080, D741~D747.

D071. What was your employment status? (workplace since [D045]: [Industry D046] [Occupation D051])

① self-employed (employer)

⑤ Unpaid family worker

D072. How many officially paid employees did you have? Please exclude temporary workers employed in farming seasons or busy times (unit: a person, workplace since [D045]: [Industry D046] [Occupation D051])

[IWER: Mark 0 if there was no employee.]

__________People [range:0~9997] →Go to D074

D073. Then what is the closest approximation to the actual number of employees in your business? (Workplace since [D045]: [Industry D046] [Occupation D051])

① Fewer than 5 people

② 5 to 9 people

③ 10 to 29 people

④ 30 to 49 people

⑤ 50 to 99 people

⑥ 100 to 299 people

⑦ 300 to 499 people

⑧ 500 to 999 people

⑨ More than 1,000 people

D074. Did you have unpaid family workers other than officially paid employees? If so, please state the number including yourself. (unit: a person, workplace since [D045]: [Industry D046] [Occupation

D051])

[IWER: Mark 0 if there was no employee.]

_______People [range:0~20]

Logic

■ If D071=①(self-employed workers), then go to D075~D077.

D075. Did you have any business partner? (Workplace since [D045]: [Industry D046] [Occupation D051])

① Yes

⑤ No →Go to D078

D076. How many partners did you have? Please state the number excluding yourself. (unit: a person, workplace since [D045]: [Industry D046] [Occupation D051])

_____people [range:1~20]

D077. What was your share of ownership in this business? (unit: %, workplace since [D045]: [Industry D046] [Occupation D051])

_____% [range:1~99]

Logic

■ If D078=⑤, then go to D741.

D078. (As loop=1) Did your employment status change within the workplace since then? (Workplace since [D045]: [Industry D046] [Occupation D051])/ (As loop>=2) Did your employment status change again after that? (Workplace since [D045]: [Industry D046] [Occupation D051])

[IWER: Change in employment status means a change from "self-employed to unpaid family worker" or a change from "unpaid family worker to self-employed (employer)".]

① Yes

⑤ No →Go to D741

D079. What was your new employment status? (Workplace since [D045]: [Industry D046] [Occupation D051])

① Self-employed (employer) to unpaid family worker

⑤ Unpaid family worker to self-employed (employer)

D080. When did your employment status change? (unit: year and month combined into 6 digits, workplace since [D045]: [Industry D046] [Occupation D051])
[IWER: Enter the year and month using 6 digits. For example, mark 200701 for January 2007. If the month is not clear, enter 200700.]

____

Logic
■ If D741=⑤, then go to D081.

D741. (As loop=1) You have responded that your job was [D051]. Did your work change within the workplace since then? (workplace since [D045]: [Industry D046] [Occupation D051])/ (As loop>=2) Did your work change within the workplace again after that?
[IWER: Only changes between major occupational groups are considered work changes.]
① Yes
⑤ No →Go to D081

D742. What was your new work? (workplace since [D045]: [Industry D046] [Occupation D051])
[IWER: Use a memo function to write on detail contents]
① Managers →Go to D747
② (Associate) Professionals →Go to D747
③ Clerks →Go to D743
④ Service workers →Go to D744
⑤ Sales workers →Go to D747
⑥ Skilled agricultural, forestry, and fishery workers →Go to D745
⑦ Craft and related trades workers →Go to D747
⑧ Equipment, machine operating and assembling workers →Go to D747
⑨ Elementary workers →Go to D746
⑩ Career soldiers →Go to D747

D743. Specifically speaking, which occupational classification best describes your work?
[IWER: Use a memo function to write on detail contents]
① General office clerks
② Customer services clerks
→Go to D747

D744. Specifically speaking, which occupational classification best describes your work?

[IWER: Use a memo function to write on detail contents]

① Personal services related workers

② Cooking and food service workers

③ Travel and transport related workers

④ Protective service workers

→Go to D747

D745. Specifically speaking, which occupational classification best describes your work?

[IWER: Use a memo function to write on detail contents]

① Skilled agricultural and livestock workers

② Skilled forestry workers

③ Skilled fishery workers

→Go to D747

D746. Specifically speaking, which occupational classification best describes your work?

[IWER: Use a memo function to write on detail contents]

① Housekeeping, cleaning, and laundry workers

② Building management and security related workers

③ Delivery, moving, and meter reading workers

④ Sanitation workers

⑤ Other services related elementary workers

⑥ Agriculture, forestry, and fishery related elementary workers

⑦ Manufacturing related elementary workers

⑧ Mining and construction related elementary workers

⑨ Transport related elementary workers

→Go to D747

D747. When did your work change? (unit: year and month combined into 6 digits, workplace since [D045]: [Industry D046] [Occupation D051])

[IWER: Enter the year and month using 6 digits. For example, mark 200701 for January 2007. If the month is not clear, enter 200700.]

____

Logic

■ R cannot answer ‘Don’t know’ or’ Refuse to answer’ to D081.

D081. Are you currently working in this workplace? (Workplace since [D045]: [Industry D046]

[Occupation D051])
[IWER:
- In case of wage workers, moving to another workplace is regarded as having a different job.
- In case of non-wage worker, changing business content is regarded as having a different job.
- Changing employment status from wage worker to non-wage worker or vice versa is regarded as having a different job.]
① Yes →Go to D085
⑤ No

D751. How many days per week did you work?
_____days [range:1~7]

D752. How many hours per week did you work excluding lunch time, break, etc.?
_____hours [range:1~160]

Logic
■ If D044=① or D071=①, then go to D753. If D071=⑤ (unpaid family workers), then go to D082.

D753. How much profit per month did you earn from this business? Please state a net profit excluding expenses.
[IWER:
- “Please state profits excluding legally required deductions, such as taxes, national pension contributions, health insurance premiums and employment insurance premiums.”
- Mark 0 if R was self-employed and made no net profit, and enter 999997 if R ran a deficit.]

______MW [range:0~999997]

D082. When did you quit this job? (unit: year and month combined into 6 digits, workplace since [D045]: [Industry D046] [Occupation D051])
[IWER: Enter the year and month using 6 digits. For example, mark 200701 for January 2007. If the month is not clear, enter 200700.]
_______________ [range:200600~200812]

D083. Why did you quit? (workplace since [D045]: [Industry D046] [Occupation D051])
(in the case of wage workers)
① Bankruptcy, closure, temporary shutdown, etc., of business
② Layoff, dehiring, voluntary early retirement, etc.

③ Contract expired

④ Retirement (at the mandatory retirement age)

⑤ Too low wage

⑥ Work was temporary or did not have a good prospect

⑦ Aptitude, knowledge, and skills did not match well

⑧ Long working hours/unfavorable working conditions

⑨ Marriage, childbirth, childcare, care for sick family member, etc.

⑩ Study or military service

⑪ Found a better job

⑫ To start up a new business

⑬ Poor health

⑭ Wanted to take a break

⑮ Others

(in the case of non-wage worker)

① Business was slow

② Work did not have a good prospect

③ Aptitude, knowledge, and skills did not match well

④ Long working hours/unfavorable working conditions

⑤ Marriage, childbirth, childcare, care for sick family member, etc.

⑥ Moved house

⑦ Study or military service

⑧ Found a paid job

⑨ To start up a new business

⑩ For unpaid assistance in family business

⑪ Poor health

⑫ Wanted to take a break

⑬ Others

Logic

■ R cannot answer 'Don't know' or' Refuse to answer' to D084.

D084. Did you take a different job after you quit? (workplace since [D045]: [Industry D046] [Occupation D051])

① Yes →Go to D044

⑤ No

- Current job: Wage worker

Logic

- If D002=①, then go to a section of wage worker.
- Only a new wage worker can answer this section.

Logic

■ If D043=①(a new wage worker), then go to D101~D114.

D101. Is the promised period of employment (employment contract period) or the period in which you can continue to work at your current job fixed?

① Fixed

⑤ Not fixed →Go to D104

D102. How long is the fixed period of employment? (unit: a month)

[IWER: If R answered a year, calculate to months]

_____months [range:1~60]

D103. Has the current employment contract ever been renewed?

① Yes →Go to D105

⑤ No (In case of first contract) →Go to D105

D104. If the employment period is not fixed, do you work for a few days or a few weeks when there is work available?

[IWER: Mark 'No' if R’s work lasts more than one month.]

① Yes

⑤ No

D105. Did you receive a written employment contract from your current employer?

① Yes

⑤ No

D106. Do you think you could keep your current job unless the company closes down due to business difficulties or you are laid off?

① Yes →Go to D110

⑤ No

D107. How long do you expect to work at your current workplace?

① Less than one year

② One to two years

③ Two to three years →Go to D110

④ More than three years →Go to D110

D108. Why do you expect so?

① Because the fixed contract period will expire →Go to D110

② Because typically the contract expires (although there's no written contract) →Go to D110

③ Because I was hired on the condition that I will resign upon the request of the employer →Go to

④ Because the current job / project will be completed →Go to D110

⑤ Because the person I am replacing will return to work →Go to D110

⑥ Because I can only work during certain seasons →Go to D110

⑦ Because I plan to find another job that better suits my job aptitude, ability, and preference →Go to

⑧ Because I will reach the retirement age set by regulations / practice →Go to D110

⑨ Because of family care responsibility, poor health, etc. →Go to D110

⑩ Others

D109. If others, please specify.

____

D110. Do you receive your wages from the employer you currently work for or from a dispatch / contract company?

* POINT*
- Dispatch company worker: those who are employed by a dispatch company (temporary agency), but working in the workplace of a person using their services, pursuant to the Act on the Protection of Dispatched Workers. Their wages and employment status are controlled by the dispatch company, but with regard to the way they provide services and work-related instructions, they are under the direction and supervision of the using company.<br>- Contract company worker: those who are employed by a contract company, but working in the workplace of a person using their services. They are not subject to the Act on the Protection of Dispatched Workers, and with regard to wages, employment status, work-related instructions, and so on, they are under the direction and supervision of the contract company. They include contract cleaning workers, contract security guards, etc.

① Employer
③ Dispatch Company
⑤ Contract Company

D111. Is there a mandatory retirement age at your current workplace?
① Yes
⑤ No →Go to D113

D112. What is the mandatory retirement age? (unit: an age)
[IWER:
- Count a person's age in full, not Korean age(same as American age).
- If the retirement age is different depending on position, ask the retirement age that applies to R.]

_____years old [range:45~80]

D113. Are there cases where employees retire upon reaching a certain age regardless of regulations?
① Yes
⑤ No →Go to D115

D114. What is the customary retirement age? (unit: an age)
[IWER: Count a person's age in full, not Korean age.]
_____years old [range:45~97]

- The most of wage worker

D115. Are you in a position to supervise others?
① Yes
⑤ No →Go to D117

D116. How many people are there under your supervision?
① fewer than 5 people
② 6~10 people
③ 11~15 people
④ 16~30 people
⑤ 31~99 people

⑥ more than 100 people

D117.Are there fixed working hours at your current workplace?

① Yes

⑤ No

D118. About how many days a week do you usually work? (unit: a day)

_____days [range:1~7]

D119. About how many hours a week do you work, excluding lunch time? (unit: an hour/per week)

_____hours [range:1~160]

D120. What do you think of your current working hours including regular and overtime hours?

① Too long

③ Appropriate →Go to D127

⑤ Too short →Go to D124

D121. If you consider your working hours too long, would you like to reduce them even if your earnings are reduced?

① Yes

⑤ No →Go to D127

D122. What % of your total working hours would you like to reduce? (unit: %)

_____% [range:1~99]

D123. What % cut from your total earnings are you willing to accept? (unit: %)

_____% [range:1~99] →Go to D127

D124. If you consider your working hours too short, are you willing to increase them if your earnings increase as a result?

① Yes

⑤ No →Go to D127

D125. What % of your total working hours would you like to increase?

[IWER: Maximum is 100. if R answered up to 100%, just mark 100]

_____% [range:1~100]

D126. What % of your total earnings has to be increased?

_____% [range:1~99]

D127. Do you have regularly scheduled days off? If you do, how many days off do you have per month?

① None

② one day per month

③ 2~3 days per month

④ 4~5 days per month

⑤ 6~7 days per month

⑥ More than 8 days per month

D128. Except for national holidays, how many days of paid leave do you have this year at your current workplace? (unit: a day)

[IWER: Mark 0 if there is no paid leave.]

_____days [range:0~365]

D129. How many days of paid sick leave do you have this year at your current workplace?

_____days [range:0~365]

Logic

■ If R started working at his/her current workplace before JAN 2007, go to D130. If not, go to D131.

D130. How many days of work did you miss due to health problems last year?

[IWER: Mark 0 if R didn't miss any work day.]

_____days [range:0~365]

D131.On what basis is your wage determined? Please select the most important one.

① Yearly basis →Go to D133

② Monthly basis →Go to D133

③ Weekly basis →Go to D133

④ Daily basis →Go to D133

⑤ Hourly basis →Go to D133

⑥ Contract-based →Go to D133

⑦ Performance-based (no basic salary) →Go to D133

⑧ Others →Go to D133

D132. If others, please specify.

____

D133. Is your wage seniority-based? That is, wage is directly linked to the number of years you have worked?

① Yes

⑤ No

D134. How much are you paid per month? (unit: 10,000 Korean won)

[IWER: "Please say the total amount excluding legally required deductions, such as taxes, national pension contributions, health insurance premiums, and employment insurance premiums."]

_____MW [range:1~9997]

D135. We are interested in the benefits you receive from your current employer. First, are you currently enrolled in the Public Pension System? If so, are you enrolled through your current employer? Or, are you enrolled in the Occupational Pension Plan for Public Workers, Teachers, and Soldiers? Or, are you enrolled in the National Pension System through the community?

① Yes, enrolled in the National Pension System through the current employer

② Yes, enrolled in the Occupational Pension Plan (for public servants, teachers and soldiers)

③ Yes, enrolled in the National Pension System through the community

④ No, not enrolled in the National Pension System

D136. Are you currently enrolled in the National Health Insurance through your employer? Or you are currently enrolled in the National Health Insurance, but not through your current employer? Or are you not currently enrolled in the National Health Insurance?

① Yes, enrolled in the National Health Insurance through the current employer

③ Yes, enrolled in the National Health Insurance, but not through the current employer

⑤ No, not enrolled in the National Health Insurance

D137. Does your employer provide the Employment Insurance? The Employment Insurance provides protection against job loss.

① Yes

⑤ No

D138. Does your employer provide the Industrial Accident Compensation Insurance? The Industrial Accident Compensation Insurance provides protection against job-related accidents and injuries.

① Yes

⑤ No

D139. Are you covered by a pension or retirement plan through your current work? If you are, what kind of retirement plan do you have? Is it under the retirement allowance scheme, retirement insurance scheme, or retirement pension scheme?

* POINT*
- Retirement Insurance Scheme: An employer puts aside retirement funds in an insurance company (outside the company). The insurance company will then pay the retirees the benefits either in the form of annuity payments or lump-sum payments.<br>- Retirement Pension Scheme: A retiree will receive annuity payments after the retirement age of 55.

① Yes, Retirement Allowance Scheme

② Yes, Retirement Insurance Scheme

③ Yes, Retirement Pension Scheme →Go to D142

④ No, no retirement plan →Go to D143

D140. Have you ever taken out some money from your retirement savings at your current workplace?

① Yes

⑤ No →Go to D143

D141. When did you take out money from your retirement savings?

[IWER: Enter the year and month using 6 digits. For example, mark 200901 for January 2007. If the month is not clear, enter 200700.]

__________ [range:190000~200812) →Go to D143

D142. Is your plan Defined Benefits or Defined Contributions?

* POINT*
- Defined Benefits: Benefits are pre-determined, usually based on a formula involving age, years of service and salary.

- Defined Contributions: Money is accumulated as the employer makes pre-determined amount of contributions.

① Defined Benefits (DB)

⑤ Defined Contributions (DC)

D143. The following is fringe benefits which may be provided by a company. Please answer if the following is provided by your current workplace. Do you get meal allowances?

① Yes

⑤ No

D144. Does your employer support children's school expenses?

① Yes

⑤ No

D145. Does your employer provide a loan for house purchase?

① Yes

⑤ No

D146. Does your employer compensate for lost wages in the case of temporary shutdown?

① Yes

⑤ No

D147. Does your employer pay insurance premiums for your personal pension plan?

[IWER: Personal pension refers to privately owned retirement savings, not including the National Pension.]

① Yes

⑤ No

D148. Is there a labor union at your current workplace?

① Yes

⑤ No →Go to D154

D149. Are you entitled to join a labor union?

① Yes

⑤ No →Go to D154

D150. Are you a member of a labor union?

① Yes

⑤ No →Go to D152

D151. If you are a member, was membership mandatory?

① Yes →Go to D155

⑤ No →Go to D155

D152. If you are not a member, why did you not become a member?

① Not satisfied with union activities →Go to D155

② Dissuaded by my family, friends, colleagues, etc. →Go to D155

③ Dissuaded by my employer →Go to D155

④ Did not feel the need →Go to D155

⑤ Others

D153. If others, please specify.

____

D154. Would you become a member if a labor union existed or you deserve to apply to a labor union?

① Yes

⑤ No

D155. Please answer to what extent the following applies to your current job. Please tell me whether you strongly agree, agree, disagree, or strongly disagree. My job requires lots of physical effort.

① Strongly agree

② Agree

③ Disagree

④ Strongly disagree

D156. My job requires lifting heavy loads.

① Strongly agree

② Agree

③ Disagree

④ Strongly disagree

D157. My job requires stooping, kneeling, or crouching.

① Strongly agree

② Agree

③ Disagree

④ Strongly disagree

D158. My job requires good eyesight.

① Strongly agree

② Agree

③ Disagree

④ Strongly disagree

D159. My job requires intense concentration and attention to details.

① Strongly agree

② Agree

③ Disagree

④ Strongly disagree

D160. My job requires skills in dealing with people.

① Strongly agree

② Agree

③ Disagree

④ Strongly disagree

D161. My job requires me to work with computers.

① Strongly agree

② Agree

③ Disagree

④ Strongly disagree

D162. My job requires me to do more challenging tasks than it used to.

① Strongly agree

② Agree

③ Disagree

④ Strongly disagree

D163. In promotion, seniority is important at my company.

① Strongly agree

② Agree

③ Disagree

④ Strongly disagree

D164. My co-workers make older workers feel that they ought to retire before the retirement age.

① Strongly agree

② Agree

③ Disagree

④ Strongly disagree

D165. My employer would let older workers move to less demanding job with less pay if they wanted to.

① Strongly agree

② Agree

③ Disagree

④ Strongly disagree

D166. My salary is adequate.

① Strongly agree

② Agree

③ Disagree

④ Strongly disagree

D167. My job security is good.

① Strongly agree

② Agree

③ Disagree

④ Strongly disagree

D168. I am satisfied with the work environment of my job.

① Strongly agree

② Agree

③ Disagree

④ Strongly disagree

D169. I am satisfied with the work I do at my current job.

① Strongly agree

② Agree

③ Disagree

④ Strongly disagree

D170. My job involves a lot of stress.

① Strongly agree

② Agree

③ Disagree

④ Strongly disagree

D171. All things considered, I am satisfied with my current job.

① Strongly agree

② Agree

③ Disagree

④ Strongly disagree

D172. Considering my educational attainment, the level of my current job is

① Very low

② Low

③ Appropriate

④ High

⑤ Very high

D173. Considering my skills and expertise, the level of my current job is

① Very low

② Low

③ Appropriate

④ High

⑤ Very high

D174. Do you plan to continue working at your current job? Please tell me if you want to stay at the current job, switch to a different job, or quit the job. If you want to stay at the current job, do you want to keep the same level of work load, work more, add another job, or work less?

① Yes, I plan to continue my current job, as it is →D178

② Yes, I plan to continue my current job, but want to increase work load →D178

③ Yes, I plan to continue my current job, but want to add another job

④ Yes, I plan to continue my current job, but want to work less →D178

⑤ No, I would like to take a different job

⑥ No, I would like to stop working altogether →D178

D175. What would be the ideal employment status for you?

① Full-time wage worker

③ Part-time wage worker

⑤ Self-employed

D176. What do you want to do for your new job? Please select from below.

① Managers

② (Associate) Professionals

③ Clerks

④ Service workers

⑤ Sales workers

⑥ Skilled agricultural, forestry, and fishery workers

⑦ Craft and related trades workers

⑧ Equipment, machine operating and assembling workers

⑨ Elementary workers

⑩ Career soldiers

D177. What would be the minimum wage or salary per month? (Unit: 10,000 Korean won)

[IWER: Mark 0 if R says salary does not matter.]

_____MW [range:0~99997]

D178. Now I want to ask about your retirement plans. At what age do you plan to retire?

[IWER:

- Count a person's age in full, not Korean age(same as American age).

- Mark 0 if R plans to keep working as long as he/she is physically capable.

- Retirement refers to having stopped doing income-earning activities and presently not working or engaging only in pastime work, and having no intention of engaging in anything more serious than pastime work as long as there is no special change in circumstances.]

_____years old [range:0~97]

D179. Are you engaged in other job(s) except the above-mentioned main job?

[IWER: Only include paid jobs. Activities without pay, such as community services, are not included.]

① Yes

⑤ No →Go to D185

D180. How many jobs do you currently have including the main job? (unit: number)

_____ [range:2~7]

D181. What are the reasons for having other job(s) than your main job?

[IWER: Please state the most important reason only.]

① To earn more money →Go to D183

② For my health →Go to D183

③ Because it is the work I wanted to do →Go to D183

④ For self-development →Go to D183

⑤ To prepare myself when I quit my main job →Go to D183

⑥ To utilize spare time →Go to D183

⑦ To help society or other people →Go to D183

⑧ Others

D182. If others, please specify.

____

D183. How many hours a week do you usually work on this other job / these other jobs?(unit: an average hour per week)

_____hours [range:1~97]

D184. On average how much per month do you earn from this other job / these other jobs?(Unit: 10,000 Korean won)

_____ MW [range:1~99997]

D185. [IWER: How often did R receive assistance in answering section D-EMPLOYMENT?]

① Never →Go to section E.

② A few times →Go to section E.

③ Most or all of the time →Go to section E.

④ The section was done by a proxy respondent.

D186. What is the proxy's relationship to R? If unknown, please ask the proxy. What is your relationship to R?

① Spouse

② Mother

③ Father

④ Mother-in-law

⑤ Father-in-law

⑥ Brother/sister

⑦ Brother-in-law/sister-in-law

⑧ Son/daughter

⑨ Son-in-law/Daughter-in-law

⑩ Grandchild

⑪ Other relatives

⑫ Helper or other non-relatives

→Go to section E.

- Current job: Self-employed worker

Logic

- If D002=②, then go to a section of self-employed worker.
- Only a new self-employed worker can answer this section.

Logic

■ If D043=①(a new self-employed worker), then go to D201~D211.

D201. What is the most important reason for you to run your own business, rather than getting a paid job? Please choose the most important reason.

① Because I can earn more money →Go to D203

② Because it is the work I wanted to do →Go to D203

③ Because it gives me more flexibility →Go to D203

④ Because of tax benefits →Go to D203

⑤ Because I couldn't get the job I wanted. (e.g. inadequate salary, work environment, etc) →Go to D203

⑥ Because it was difficult to get a paid job →Go to D203

⑦ Others

D202. If others, please specify.

____

Logic

■ If D203=0(non-initial expenses), then go to D204, If D203>0(existing initial expenses), then go to D205

D203. How much did you spend or invest to start up this business? (unit: 10,000 Korean won)

___MW [range:0~999997]

D204. What is the reason for not needing initial expenses?

___________________→Go to D206

D205. How did you finance your business expenses? Please make sure the sum of all items is 100%. [IWER: ① R’s and his/her spouse's savings, ② Help or borrowings from R’s and his/her spouse’s parents, ③ Help or borrowings from R’s children/grandchildren, ④ Borrowings from R’s and his/her relatives, ⑤ Borrowings from other individuals, ⑥ Partner, ⑦ Loans from banks and other financial institutions, ⑧ Government subsidy, ⑨ Others]

NO	ITEMS	Percentage(%)
205a	Own/spouse's savings	
205b	Help or borrowings from parents/spouse’s parents,	
205c	Help or borrowings from children/grandchildren	
205d	Borrowings from relatives/spouse’ relatives,	
205e	Borrowings from other individuals	
250f	Partner,	
205g	Loans from banks and other financial institutions	
205h	Government subsidy	
205i	Others	
	Total	100%

D206. Next I will ask you about the general difficulties people may face in starting a new business. Please answer which one you belonged to. How difficult was it to secure sufficient financing?

① Very difficult

② Difficult

③ Fair

④ Easy

⑤ Very easy

D207. How difficult was it to secure technology?

① Very difficult

② Difficult

③ Fair

④ Easy

⑤ Very easy

D208. How difficult was it to secure appropriate human resources?

① Very difficult

② Difficult

③ Fair

④ Easy

⑤ Very easy

D209. How difficult was it to deal with administrative procedures (approval, business start-up support, etc.)?

① Very difficult

② Difficult

③ Fair

④ Easy

⑤ Very easy

D210. How difficult was it to choose the industry?

① Very difficult

② Difficult

③ Fair

④ Easy

⑤ Very easy

D211. How difficult was it to choose the business location?

① Very difficult

② Difficult

③ Fair

④ Easy

① Very easy

- The most of self-employed workers can answer this section.

D212. How much is the current monthly sales of your business? For the agricultural and fisheries industry, please include revenues (including expenses) from the sales of agricultural and fisheries' products. (Unit: 10,000 Korean won)

[IWER:

- Enter 0 if there were no sales in present

______MW [range:0~999997] →Go to D214
Don't know →Go to D213

Refuse to answer →Go to D213

D213. What is the closest approximation to the current monthly sales figure?

① Less than 100 MW

② 100~300 MW

③ 300~500 MW

④ 500~800 MW

⑤ 800~1,000 MW

⑥ 1,000~3,000 MW

⑦ 3,000~5,000 MW

⑧ 5,000~10,000 MW

⑨ 10,000 MW or more

D214. How much profit per month do you earn from this business? Please state a net profit excluding expenses. (Unit: 10,000 Korean won)
[IWER:

- Mark 0 if there is no net profit, and mark 999997 if R is running a deficit.

______MW [range:0~999997]

D215. How many days per week do you work? (unit: a day)
_____days [range:1~7]

D216. How many hours per week do you work excluding lunch time, break, etc.? (unit: an hour per week)
_____hours [range:1~160]

D217. Do you have fixed and regular working hours?

① Yes →Go to D220

⑤ No

D218. How did you determine the working hours?

① Working hours were determined given the nature of the business →Go to D220

② Working hours were determined depending on workloads →Go to D220

③ Working hours were determined depending on sales figures →Go to D220

④ Others

D219. Specifically speaking, what criteria did you use to determine the working hours?

____

D220. What do you think of your current working hours? Is it too long, appropriate, or too short?

① Too long

③ Appropriate →Go to D227

⑤ Too short →Go to D224

D221. If you consider your working hours too long, would you like to reduce them even if your earnings are reduced?

① Yes

⑤ No →Go to D227

D222. What % of your total working hours would you like to reduce? (unit: %)

_____% [range:1~99]

D223. What % cut from your total earnings are you willing to accept? (unit: %)

_____% [range:1~99] →Go to D227

D224. If you consider your working hours too short, are you willing to increase them if your earnings increase as a result?

① Yes

⑤ No →Go to D227

D225. What % of your total working hours would you like to increase? (unit: %)

_____% [range:1~99]

D226. What % of your total earnings has to be increased? (unit: %)

_____% [range:1~99]

D227. Do you have regularly scheduled days off? If so, how many days off do you have per month?

① None

② One day per month

③ 2~3 days per month

④ 4~5 days per month

⑤ 6~7 days per month

⑥ More than 8 per month

Logic

■ If R started his/her current business before JAN 2007, go to D228. If not, go to D229.

D228. How many days of work did you miss due to health problems last year? (unit: a day)

[IWER: Mark 0 if R didn't miss any work day.]

_____days [range:0~365]

D229. Please answer to what extent the following applies to your current job. Please tell me whether you strongly agree, agree, disagree, or strongly disagree. My job requires lots of physical effort.

① Strongly agree

② Agree

③ Disagree

④ Strongly disagree

D230. My job requires lifting heavy loads.

① Strongly agree

② Agree

③ Disagree

④ Strongly disagree

D231. My job requires stooping, kneeling, or crouching.

① Strongly agree

② Agree

③ Disagree

④ Strongly disagree

D232. My job requires good eyesight.

① Strongly agree

② Agree

③ Disagree

④ Strongly disagree

D233. My job requires intense concentration and attention to details.

① Strongly agree

② Agree

③ Disagree

④ Strongly disagree

D234. My job requires skills in dealing with people.

① Strongly agree

② Agree

③ Disagree

④ Strongly disagree

D235. My job requires me to work with computers.

① Strongly agree

② Agree

③ Disagree

④ Strongly disagree

D236. My job requires me to do more challenging tasks than it used to.

① Strongly agree

② Agree

③ Disagree

④ Strongly disagree

D237. My salary is adequate.

① Strongly agree

② Agree

③ Disagree

④ Strongly disagree

D238. My job security is good.

① Strongly agree

② Agree

③ Disagree

④ Strongly disagree

D239. I am satisfied with the work environment of my job.

① Strongly agree

② Agree

③ Disagree

④ Strongly disagree

D240. I am satisfied with the work I do at my current job.

① Strongly agree

② Agree

③ Disagree

④ Strongly disagree

D241. My job involves a lot of stress.

① Strongly agree

② Agree

③ Disagree

④ Strongly disagree

D242. All things considered, I am satisfied with my current job.

① Strongly agree

② Agree

③ Disagree

④ Strongly disagree

D243. Considering my educational attainment, the level of my current job is

① Very low

② Low

③ Appropriate

④ High

⑤ Very high

D244. Considering my skills and expertise, the level of my current job is

① Very low

② Low

③ Appropriate

④ High

⑤ Very high

D245. Do you plan to continue working at your current job? Please tell me if you want to stay at the current job, switch to a different job, or quit the job. If you want to stay at the current job, do you want to keep the same level of work load, work more, add another job, or work less?

① Yes, I plan to continue my current job, as it is →Go to D249

② Yes, I plan to continue my current job, but want to increase work load →Go to D249

③ Yes, I plan to continue my current job, but want to add another job

④ Yes, I plan to continue my current job, but want to work less →Go to D249

⑤ No, I would like to take a different job.

⑥ No, I would like to stop working altogether →Go to D249

D246. What would be the ideal employment status for your new job?

① Full time wage worker

③ Part time wage worker

⑤ Self-employed

D247. What do you want to do for your new job?

① Managers

② (Associate) Professionals

③ Clerks

④ Service workers

⑤ Sales workers

⑥ Skilled agricultural, forestry, and fishery workers

⑦ Craft and related trades workers

⑧ Equipment, machine operating and assembling workers

⑨ Elementary workers

⑩ Career soldiers

D248. What would be the minimum wage or income per month? (Unit: 10,000 Korean won/average per month)

[IWER:

- Mark 0 if R says salary doesn't matter.

___MW [range:0~99997]

D249. Now I want to ask about your retirement plans. At what age do you plan to retire? (unit: an age)

[IWER:

- Count a person's age in full, not Korean age(same as American age).

- Mark 0 if R plans to keep working as long as he/she is physically capable.

- Retirement refers to having stopped doing income-earning activities and presently not working or engaging only in pastime work, and having no intention of engaging in anything more serious than pastime work as long as there is no special change in circumstances.]

_____years old [range:0~97]

D250. Are you engaged in other job(s) than the above-mentioned main job?

[IWER: Only include paid jobs. Activities without pay, such as community services, are not included.]

① Yes

⑤ No →Go to D256

D251. How many jobs do you have including your main job?

_____ [range:2~7]

D252. What are the reasons for having other job(s) than your main job? Please state the main reason only.

① To earn more money →Go to D254

② For my health →Go to D254

③ Because it is the work I wanted to do →Go to D254

④ For self-development →Go to D254

⑤ To utilize spare time →Go to D254

⑥ To prepare myself when I quit my main job →Go to D254

⑦ To help society or other people →Go to D254

⑧ Others

D253. If others, please specify.

____

D254. How many hours per week do you usually work on this other job/these other jobs? (unit: an hour)

_____hours [range: 1~97]

D255. On average how much per month do you earn from this other job/these other jobs? (Unit:10,000 Korean won)

_______MW [range: 1~9997]

D256. [IWER: How often did R receive assistance in answering section D-EMPLOYMENT?]

① Never →Go to section E.

② A few times →Go to section E.

③ Most or all of the time →Go to section E.

④ The section was done by a proxy respondent.

D257. What is the proxy's relationship to R? If unknown, please ask the proxy. What is your relationship to R?

① Spouse

② Mother

③ Father

④ Mother-in-law

⑤ Father-in-law

⑥ Brother/sister

⑦ Brother-in-law/sister-in-law

⑧ Son/daughter

⑨ Son-in-law/Daughter-in-law

⑩ Grandchild

⑪ Other relatives

⑫ Helper or other non-relatives

→Go to section E.

- Current job : Unpaid family worker

Logic

■ If D002=③, then go to a section of unpaid family worker.

■ Only a new unpaid family worker can answer this section.

Logic

■ If D043=①(a new unpaid family worker), then go to D301~D302.

D301. What is the main reason you didn't get a paid job and chose to work on the current job?

① Because I can earn more money →Go to D303

② Because it is the work I wanted to do →Go to D303

③ Because it gives me more flexibility →Go to D303

④ Because I couldn't get the job I wanted (e.g. salary and work environment) →Go to D303

⑤ Because it was difficult to get a paid job →Go to D303

⑥ Others

D302. If others, please specify.

____

- The most of new unpaid family workers can answer this section.

D303. Who is the representative of your current workplace?

① Spouse →Go to D305
② Parents →Go to D305
③ Parents-in-law →Go to D305
④ Brother/sister →Go to D305
⑤ Brother-in-law/sister-in-law →Go to D305
⑥ Children and grandchildren →Go to D305
⑦ Other relatives →Go to D305
⑧ Others

D304. Specifically speaking, who is the representative of your current workplace?

____

D305. How much is the current monthly sales of your business? For the agricultural and fisheries industry, please include revenues (including expenses) from the sales of agricultural and fisheries' products. (Unit: 10,000 Korean won)
[IWER: Enter 0 if there were no sales in present.]

_____MW [range:0~999997] →Go to D307
Don't know →Go to D306
Refuse to answer →Go to D306

D306. What is the closest approximation to the present sales figure?
① Less than 100 MW
② 100~300 MW
③ 300~500 MW
④ 500~800 MW
⑤ 800~1,000 MW
⑥ 1,000~3,000 MW

⑦ 3,000~5,000 MW

⑧ 5,000~10,000 MW

⑨ 10,000 MW or more

D307. How many hours per week do you work, excluding lunch time, break time, etc.? (unit: an average hour per week)

[IWER: It is possible to enter 18 hours or more. If R says 17 hours or less, go back to D002 and mark 'I help my family / relative's business without pay for less than 18 hours a week'.]

_____hours / week [range:18~160]

D308. Do you have fixed and regular working hours?

① Yes →Go to D311

⑤ No

D309. How did you determine the working hours?

① Working hours were determined given the nature of the business →Go to D311

② Working hours were determined depending on workloads →Go to D311

③ Working hours were determined depending on sales figures →Go to D311

④ Others

D310. Specifically speaking, what criteria did you use to determine the working hours?

____

D311. How many days per week do you work for this business? (unit: a day)

Average_____days / per week [range:1~7]

D312. What do you think of your current working hours?

① Too long

③ Appropriate →Go to D317

⑤ Too short →Go to D315

D313. If you consider your working hours too long, would you like to reduce them?

① Yes

⑤ No →Go to D317

D314. What % of your total working hours would you like to reduce? (unit : %)

__________% [range:1~99] →Go to D317

D315. If you consider your working hours too short, are you willing to increase them?

① Yes

⑤ No →Go to D317

D316. What % of your total working hours would you like to increase? (unit : %)

_____% [range:1~99]

D317. Do you have regularly scheduled days off?

① No

② 1 Day per month

③ 2 to 3 days per month

④ 4 to 5 days per month

⑤ 6 to 7 days per month

⑥ 8 days or more per month

D318. How many days of work did you miss due to health problems last year (2007)? (unit: a day)

_____days [range:0~365]

D319. Please answer to what extent the following applies to your current job. Please tell me whether you strongly agree, agree, disagree, or strongly disagree. My job requires lots of physical effort.

① Strongly agree

② Agree

③ Disagree

④ Strongly disagree

D320. My job requires lifting heavy loads.

① Strongly agree

② Agree

③ Disagree

④ Strongly disagree

D321. My job requires stooping, kneeling, or crouching.

① Strongly agree

② Agree

③ Disagree

④ Strongly disagree

D322. My job requires good eyesight.

① Strongly agree

② Agree

③ Disagree

④ Strongly disagree

D323. My job requires intense concentration and attention to details.

① Strongly agree

② Agree

③ Disagree

④ Strongly disagree

D324. My job requires skills in dealing with people.

① Strongly agree

② Agree

③ Disagree

④ Strongly disagree

D325. My job requires me to work with computers.

① Strongly agree

② Agree

③ Disagree

④ Strongly disagree

D326. My job requires me to do more challenging tasks than it used to.

① Strongly agree

② Agree

③ Disagree

④ Strongly disagree

D327. I am satisfied with the work environment of my job.

① Strongly agree

② Agree

③ Disagree

④ Strongly disagree

D328. I am satisfied with the work I do at my current job.

① Strongly agree

② Agree

③ Disagree

④ Strongly disagree

D329. My job involves a lot of stress.

① Strongly agree

② Agree

③ Disagree

④ Strongly disagree

D330. All things considered, I am satisfied with my current job.

① Strongly agree

② Agree

③ Disagree

④ Strongly disagree

D331. How helpful do you think your working as an unpaid family worker is to your household income?

① Very helpful

② Somewhat helpful

③ Fair

④ Not helpful

⑤ Not at all helpful

D332. Considering my educational attainment, the level of my current job is

① Very low

② Low

③ Appropriate

④ High

⑤ Very high

D333. Considering my skills and expertise, the level of my current job is

① Very low

② Low

③ Appropriate

④ High

⑤ Very high

D334. Do you plan to continue working at your current job? Please tell me if you want to stay at the current job, switch to a different job, or quit the job. If you want to stay at the current job, do you want to keep the same level of work load, work more, add another job, or work less?

① Yes, I plan to continue my current job, as it is →Go to D338

② Yes, I plan to continue my current job, but want to increase work load →Go to D338

③ Yes, I plan to continue my current job, but want to add another job

④ Yes, I plan to continue my current job, but want to work less →Go to D338

⑤ No, I would like to take a different job

⑥ No, I would like to stop working altogether →Go to D338

Don't know →Go to D338

Refuse to answer →Go to D338

D335. What would be the ideal employment status for your new job?

① Full time wage worker

③ Part time wage worker

⑤ Self-employed

D336. What do you want to do for your new job? Please select from below.

① Managers

② (Associate) Professionals

③ Clerks

④ Service workers

⑤ Sales workers

⑥ Skilled agricultural, forestry, and fishery workers

⑦ Craft and related trades workers

⑧ Equipment, machine operating and assembling workers

⑨ Elementary workers

D337. What would be the minimum wage or income per month for this job?

[IWER:

- Mark 0 if R says salary doesn't matter.

___MW [range: 0~99997]

D338. Now I want to ask about your retirement plans. At what age do you plan to retire? (unit: an age)
[IWER:
- Mark 0 if R plans to keep working as long as he/she is physically capable.
- Retirement refers to having stopped doing income-earning activities and presently not working or engaging only in pastime work, and having no intention of engaging in anything more serious than pastime work as long as there is no special change in circumstances.]
_____years old [range:0~97]

D339. Are you engaged in other job(s) than the above-mentioned main job? Include paid jobs only. Activities without pay, such as community services, are not included.
① Yes
⑤ No →Go to D345

D340. How many jobs do you have including your main job? Please include paid jobs only.
______ [range: 2~7]

D341. What are the reasons for having other job(s) than your main job? Please state the main reason only.
① To earn more money →Go to D343
② For my health →Go to D343
③ Because it is the work I wanted to do →Go to D343
④ For self-development →Go to D343
⑤ To utilize spare time →Go to D343
⑥ To prepare myself when I quit my main job →Go to D343
⑦ To help society or other people →Go to D343
⑧ Others

D342. If others, please specify.
____

D343. How many hours a week do you usually work on this other job / these other jobs? (unit: an average hour per week)
_____hours / week [range:1~97]

D344. How much do you earn per month on average from this other job / these other jobs? (Unit: 10,000 Korean won)
_____MW [range:1~9997]

D345. [IWER: How often did R receive assistance in answering section D-EMPLOYMENT?]
① Never →Go to section E.
② A few times →Go to section E.
③ Most or all of the time →Go to section E.
④ The section was done by a proxy respondent.

D346. What is the proxy's relationship to R? If unknown, please ask the proxy. What is your relationship to R?
① Spouse
② Mother
③ Father
④ Mother-in-law
⑤ Father-in-law
⑥ Brother/sister
⑦ Brother-in-law/sister-in-law
⑧ Son/daughter
⑨ Son-in-law/Daughter-in-law
⑩ Grandchild
⑪ Other relatives
⑫ Helper or other non-relatives
→Go to section E.

- Current job: a job seeker/an unemployed person

Logic
■ If R has answered D006, go to D401.

D401. Did you ever seek a job to earn money in the past week?
① Yes →Go to D403
⑤ No

D402. Did you ever seek a job to earn money in the past four weeks?
① Yes
⑤ No →Go to D404

D403. Would you have been able to work last week if there had been an appropriate job or work?

① Yes →Go to D410

⑤ No →Go to D405

D404. Are you willing to work if there is an appropriate job?

① Yes →Go to D407

⑤ No

Logic

■ After R has answered D405 (except D405=⑧)

If D006=①, then go to D601~D613

If D006=③, then go to D501~D510

If D006=⑤, ⑦ or ⑨, then go to D422

D405. What is the main reason you are not willing to work even if there is a job? Please select the main reason only.

① Need to care for a family member

② Housework

③ No financial need

④ Old age

⑤ Poor health

⑥ Wish to take a break

⑦ (After retirement/quitting a job) Do not want to work

⑧ Others

Logic

■ After R has answered D406

If D006=①, then go to D601~D613

If D006=③, then go to D501~D510

If D006=⑤, ⑦ or ⑨, then go to D422

D406. If others, please specify.

____

D407. What is the main reason you were not looking for work although you like to get a job?

① I believe there would be no job that matches my major or professional background →Go to D409

② I believe there would be no job that meets my desired wage level or working conditions →Go to

D409

③ I don't believe I can find work →Go to D409

④ I don't have needed skills, education or other capabilities →Go to D409

⑤ I need to care for a family member →Go to D409

⑥ Housework →Go to D409

⑦ Poor health or disability →Go to D409

⑧ Others

D408. If others, please specify.

____

D409. Did you ever seek a job to earn money prior to the past four weeks?

① Yes →Go to D412

⑤ No →Go to D422

D410. What is the main reason you are looking for a job?

① To earn a living→Go to D412

② Due to the unemployment of spouse / family member →Go to D412

③ To earn pocket money →Go to D412

④ For self-development →Go to D412

⑤ To utilize spare time →Go to D412

⑥ Others

D411. If others, please specify.

____

D412. When did you start looking for a job after the previous interview? (unit: year and month combined into 6 digits)

[IWER: Enter the year and month using 6 digits. For example, mark 200701 for January 2007. If the month is not clear, enter 200700.]

______________ [range: 200600~200812] →Go to D413 or D414

Logic

■ If D409=①, then go to D413.

D413. When did you stop looking for a job? (unit: year and month combined into 6 digits)
[IWER: Enter the year and month using 6 digits. For example, mark 200701 for January 2007. If the month is not clear, enter 200700.]
____________________ [range: 200600~200812]

D414. On average how many hours per week did you spend on looking for a job when you were engaged in job search activities? (unit: an average hour per week)
[IWER: Mark 1 for less than 1 hour.]
Average________hours per week [range:1~160]

D415. How did you look for a job when you were engaged in job search activities? Please state the two most important activities in order. What is the activity you were most engaged in? First: _____
① Search through a public employment agency(Job Center of Ministry of Employment and Labor, Human Resources Bank of the aged, Senior Biz Plaza, Function Senior Club etc.) →Go to D417
② Search through a private employment agency →Go to D417
③ Search through advertisements in newspapers, TV and billboards →Go to D417
④ Search through the Internet →Go to D417
⑤ Search through friends, family members, or relatives →Go to D417
⑥ Others

D416. If others, please specify.

____

D417. What is the second activity that you were engaged in? Please state the two most important activities in order. Second: _____
① Search through a public employment agency(Job Center of Ministry of Employment and Labor, Human Resources Bank of the aged, Senior Biz Plaza, Function Senior Club etc.) →Go to D419
② Search through a private employment agency →Go to D419
③ Search through advertisements in newspapers, TV and billboards →Go to D419
④ Search through the Internet →Go to D419
④ Search through friends, family members, or relatives →Go to D419
⑤ Others

D418. If others, please specify.

____

Logic

■ If D419=0, then go to D422.

D419. Did you find a job or were you offered a job (workplace, business, work, etc.) when you were engaged in job search activities? If so, how many times? (unit: a time)
[IWER: Mark 0 if there is none.]
______ [range:0~97]

D420. What is the reason for not working in the job found and offered?
① Not hired
② Wage and working conditions were not desirable
③ Expertise, experience, skills, and aptitude did not match well
④ Physically demanding
⑤ Poor health
⑥ Housework, childcare, etc.
⑦ Others

D421.If others, please specify.
____

D422. You described your work status as [D006] at the beginning of this section. If so, what would be the ideal employment status for you?

* POINT*
- Part time: Cases in which one works part time or as a side job, works less hours than other employees who perform the same job, or is paid on an hourly basis<br>- Full time: Cases in which one works full working days as opposed to part time

① Full time wage worker →Go to D425
③ Part time wage worker
⑤ Self-employed →Go to D425

D423. Why do you want a part time job? Please state the main reason only.
① Childcare and housework
② Cannot find a full time job
③ Poor health

④ Do not want to work long hours (full time)
⑤ Others

D424. If others, please specify.

____

D425. Which workplace do you want to work in? Please state the industry that you would like to work in.
① Agriculture/forestry
② Fishing
③ Mining and quarrying
④ Manufacturing
⑤ Electricity, gas, and water supply
⑥ Construction
⑦ Wholesale and retail trade
⑧ Hotels and restaurants
⑨ Transport
⑩ Post and telecommunications
⑪ Financial and insurance activities
⑫ Real estate and renting and leasing
⑬ Other activities

D426. Specifically speaking, what kind of work do you want to do in the workplace?
① Managers
② (Associate) Professionals
③ Clerks
④ Service workers
⑤ Sales workers
⑥ Skilled agricultural, forestry, and fishery workers
⑦ Craft and related trades workers
⑧ Equipment, machine operating and assembling workers
⑨ Elementary workers

D427. What would be the minimum wage or income per month for this job? (unit: 10,000 Korean Won / month)
[IWER:

- Mark 0 if R says salary does n’t matter.

Average monthly_______MW [range:0~99997]

D428. The following is common difficulties in finding jobs. To what extent do you agree to the following difficulties?

There is a shortage of jobs.

① Strongly agree

② Agree

③ Fair

④ Disagree

⑤ Strongly disagree

D429. There is not enough recruitment information around.

① Strongly agree

② Agree

③ Fair

④ Disagree

⑤ Strongly disagree

D430. I don’t have needed education, skills or expertise.

① Strongly agree

② Agree

③ Fair

④ Disagree

⑤ Strongly disagree

D431. I don’t have needed experience.

① Strongly agree

② Agree

③ Fair

④ Disagree

⑤ Strongly disagree

D432. The wage offered is too low.

① Strongly agree

② Agree

③ Fair

④ Disagree

⑤ Strongly disagree

D433. The working environment and hours are not suitable.

① Strongly agree

② Agree

③ Fair

④ Disagree

⑤ Strongly disagree

D434. Old age makes employment difficult.

① Strongly agree

② Agree

③ Fair

④ Disagree

⑤ Strongly disagree

Logic

■ If R is female, then go to D435.

■ After D435, if D006=①, then go to D601~D613.

■ After D435, if D006=③, then go to D501~D510.

D435. Being a woman makes employment difficult.

① Strongly agree

② Agree

③ Fair

④ Disagree

⑤ Strongly disagree

Logic

■ If D006=⑤, ⑦ or ⑨, then go to D436.

D436. Now I want to ask about your retirement plans. At what age do you plan to retire? (unit: an age)

[IWER:

- Count a person's age in full, not Korean age(same as American age).

- Mark 0 if R plans to keep working as long as he/she is physically capable.

- Retirement refers to having stopped doing income-earning activities and presently not working or

engaging only in pastime work, and having no intention of engaging in anything more serious than pastime work as long as there is no special change in circumstances.]

_____years old [range:0~97]

D437. [IWER: How often did R receive assistance in answering section D-EMPLOYMENT?]

① Never →Go to section E.

② A few times →Go to section E.

③ Most or all of the time →Go to section E.

④ The section was done by a proxy respondent.

D438. What is the proxy's relationship to R? If unknown, please ask the proxy. What is your relationship to R?

① Spouse

② Mother

③ Father

④ Mother-in-law

⑤ Father-in-law

⑥ Brother/sister

⑦ Brother-in-law/sister-in-law

⑧ Son/daughter

⑨ Son-in-law/Daughter-in-law

⑩ Grandchild

⑫ Other relatives

⑬ Helper or other non-relatives

→Go to section E.

- Retiree

Logic

■ If D006=③, then go to a section of retiree.

- Only a new retiree can answer this section.

Logic

■ If D036=⑤ and D043=⑤(a new retiree) or D084=⑤, then go to D501~D508

D501. When did you retire after the previous interview? (unit: year and month combined into 6 digits) [IWER: Enter the year and month using 6 digits. For example, mark 200701 for January 2007. If the

month is not clear, enter 200700.]

__________________ [range:200600 ~ 200812]

D502. What was the most important reason you retired? First:___

① Having enough income to get by → Go to D504

② Having enough income from spouse → Go to D504

③ Didn't like to continue to work → Go to D504

④ To spend more time on leisure → Go to D504

⑤ To do volunteer work or to pursue hobby → Go to D504

⑥ Poor health → Go to D504

⑦ Poor health of spouse → Go to D504

⑧ Poor health of other family members →Go to D504

⑨ Housework or childcare →Go to D504

⑩ Could not find another job →→Go to D504

⑪ Mandatory retirement →Go to D504

⑫ Others

D503. If others, please specify.

____

D504. What was the second most important reason you retired? Second:___

① Having enough income to get by →Go to D506

② Having enough income from spouse →Go to D506

③ Did not like to continue to work →Go to D506

④ To spend more time on leisure →Go to D506

⑤ To do volunteer work or to pursue hobby →Go to D506

⑥ Poor health →Go to D506

⑦ Poor health of spouse →Go to D506

⑧ Poor health of other family members →Go to D506

⑨ Housework or childcare →Go to D506

⑩ Could not find another job →Go to D506

⑪ Others

D505. If others, please specify.

____

D506. Did you have a spouse when you retired?

① Yes

⑤ No →Go to D509

D507. What was your spouse's economic activity status at the time of your retirement?

① Employed and receiving wages →Go to D509

② Running his/her own business →Go to D509

③ Helping family or relative's business without pay for more than 18 hours a week →Go to D509

④ Helping family or relative's business without pay for less than 18 hours a week →Go to D509

⑤ Unemployed and looking for a job →Go to D509

⑥ Unemployed and not looking for a job →Go to D509

⑦ Retired →Go to D509

⑧ Others

D508. If others, please specify.

____

- The most retirees can answer this section.

D509. All in all, would you say that your retirement has turned out to be very satisfying, moderately satisfying, or not at all satisfying?

① Very satisfying

③ Moderately satisfying

⑤ Not satisfying

D510. Comparing before and after retirement, would you say your life after retirement is better than, about the same as, or worse than, before retirement?

① Better →Go to D601

③ About the same →Go to D601

⑤ Worse →Go to D601

- A pastime job

Logic

■ If D006=① or ③, then go to a section of pastime job.

D601. Are you currently engaged in a paid pastime job?

① Yes

⑤ No →Go to D607

D602. You have responded that you have a pastime job. Now I will ask you about your pastime job. What kind of work are you doing?

____

D603. Since when have you worked at this job? (unit: year and month combined into 6 digits)

[IWER: Enter the year and month using 6 digits. For example, mark 200701 for January 2007. If the month is not clear, enter 200700.]

_____ [range:190000~200812]

D604. How many days per week do you usually work at your pastime job? (unit: a day)

An average of______ days per week [range:0~7]

D605. How many hours per week do you usually work at your pastime job? (unit: an hour)

An average of________ hours per week [range:0~97]

D606. What is your monthly income from the pastime work? (Unit: 10,000 Korean won)

[IWER:

- Mark 0 if there is no net income, and mark 999997 if R is running a deficit.]

___MW [range:0~99997]

D607. Do you want to work for pay regardless of retirement status?

① Yes

⑤ No →Go to D612

D608. What is the reason?

① I want to keep working until my health allows me to →Go to D610

② I need extra money/want to help family finance →Go to D610

③ Society still needs my knowledge/expertise →Go to D610

④ To maintain good health both physically and psychologically →Go to D610

⑤ Bored at home →Go to D610

⑥ Others

D609. If others, please specify.

____

D610. If that is the case, what sort of work do you want to have?

① A pastime job I can do regardless of income →Go to D612

② Part time work that allows me to earn some pocket money →Go to D612

③ Full time work that allows me to earn a living →Go to D612

④ Self-employed (business start-up) →Go to D612

⑤ Others

D611. If others, please specify.

____

D612. [IWER: How often did R receive assistance in answering section D-EMPLOYMENT?]

① Never →Go to section E.

② A few times →Go to section E.

③ Most or all of the time →Go to section E.

④ The section was done by a proxy respondent.

D613. What is the proxy's relationship to R? If unknown, please ask the proxy. What is your relationship to R?

① Spouse

② Mother

③ Father

④ Mother-in-law

⑤ Father-in-law

⑥ Brother/sister

⑦ Brother-in-law/sister-in-law

⑧ Son/daughter

⑨ Son-in-law/Daughter-in-law

⑩ Grandchild

⑪ Other relatives

⑫ Helper or other non-relatives

→Go to section E.

E. INCOME AND CONSUMPTION

- Wage income

E001. Did you earn any income by being employed by another person or in a workplace in the last calendar year (2007)?

① Yes

⑤ No →Go to E010

E002. Which months did you get paid in the last calendar year (2007)? Please choose all that apply. (Select multiple responses)

① January 2007

② February 2007

③ March 2007

④ April 2007

⑤ May 2007

⑥ June 2007

⑦ July 2007

⑧ August 2007

⑨ September 2007

⑩ October 2007

⑪ November 2007

⑫ December 2007

E003. What was your average monthly wage in the last calendar year (2007) excluding various costs or taxes? (unit: 10,000 Korean won)

[IWER: In previous interview, an average monthly wage income: About (PL) 10,000 Korean won]

___MW [range:1~99997]

Don't know →Go to E005~E009 unfolding

Refuse to answer →Go to E005~E009 unfolding

E004. So you received a total of (amount calculated based on E002 and E003) in wages in the last calendar year (2007). Is this correct?

[IWER: In previous interview, the total of wage income: About (PL) 10,000 Korean won]

① Yes →Go to E010

⑤ No →Go to E005~E009 unfolding

Don't know →Go to E005~E009 unfolding

Refuse to answer →Go to E005~E009 unfolding

< E005~E009. Unfolding bracket questions >

E005. Did it amount to a total of less than, about equal to or more than 600 MW (10,000 Korean won) in the last calendar year (2007)?

① Less than 600 MW

③ About 600 MW

⑤ More than 600 MW

E006. Did it amount to a total of less than, about equal to, or more than 1,200 MW (10,000 Korean won)? in the last calendar year (2007)

① Less than 1,200 MW

③ About 1,200 MW

⑤ More than 1,200 MW

E007. Did it amount to a total of less than, about equal to or more than 2,400 MW (10,000 Korean won) in the last calendar year (2007)?

① Less than 2,400 MW

③ About 2,400 MW

⑤ More than 2,400 MW

E008. Did it amount to a total of less than, about equal to or more than 6,000 MW (10,000 Korean won) in the last calendar year (2007)?

① Less than 6,000 MW

③ About 6,000 MW

⑤ More than 6,000 MW

E009. Did it amount to a total of less than, about equal to, or more than 12,000 MW (10,000 Korean won) in the last calendar year (2007)?

① Less than 12,000 MW

③ About 12,000 MW

⑤ More than 12,000 MW

- Business(self-employed) income

E010. Did you earn any income from self-employment or running your own business in the last calendar year (2007)? Income from agricultural and fisheries business is excluded.

① Yes

⑤ No →Go to E019

E011. Which months did you earn income from self-employment or running your own business in the last calendar year (2007)? Please choose all that apply. (Select multiple responses)

① January 2007

② February 2007

③ March 2007

④ April 2007

⑤ May 2007

⑥ June 2007

⑦ July 2007

⑧ August 2007

⑨ September 2007

⑩ October 2007

⑪ November 2007

⑫ December 2007

E012. What was your average monthly net income in the last calendar year (2007) excluding various costs and taxes? (unit: 10,000 Korean won)

[IWER: In previous interview, an average monthly net income: About (PL) 10,000 Korean won]

___MW [range:0~99997]

Don't know →Go to E014~E018 unfolding

Refuse to answer →Go to E014 ~ E018 unfolding

E013. So you earned a total of (amount calculated based on E011 and E012) in the last calendar year (2007). Is this correct?

[IWER: In previous interview, the total of income: About (PL) 10,000 Korean won]

① Yes →Go to E019

⑤ No →Go to E014~E0a18 unfolding

Don't know →Go to E014~E018 unfolding

Refuse to answer →Go to E014~E018 unfolding

< E014~E018. Unfolding bracket questions >

E014. Did it amount to a total of less than, about equal to or more than 600 MW (10,000 Korean won) in the last calendar year (2007)?
① Less than 600 MW

③ About 600 MW

⑤ More than 600 MW

E015. Did it amount to a total of less than, about equal to or more than 1,200 MW (10,000 Korean won) in the last calendar year (2007)?
① Less than 1,200 MW

③ About 1,200 MW

⑤ More than 1,200 MW

E016. Did it amount to a total of less than, about equal to or more than 2,400 MW (10,000 Korean won) in the last calendar year (2007)?
① Less than 2,400 MW

③ About 2,400 MW

⑤ More than 2,400 MW

E017. Did it amount to a total of less than, about equal to or more than 6,000 MW (10,000 Korean won) in the last calendar year (2007)?
① Less than 6,000 MW

③ About 6,000 MW

⑤ More than 6,000 MW

E018. Did it amount to a total of less than, about equal to, or more than 12,000 MW (10,000 Korean won) in the last calendar year (2007)?
① Less than 12,000 MW

③ About 12,000 MW

⑤ More than 12,000 MW

- Agriculture and fishery income

E019. Did you earn any income from agricultural and fisheries business in the last calendar year (2007)?

① Yes

⑤ No →Go to E026

E020. How much did you earn from agricultural and fisheries business in the last calendar year (2007)? (unit: 10,000 Korean won)

[IWER:

- “Please state net income obtained by subtracting operating costs and various taxes from the

total revenue." (Wage earners in the agricultural and fisheries industries are excluded.)

- In previous interview, agriculture and fishery income: About (PL) 10,000 Korean won]

___MW [range0~99997]→Go to E026
Don't know →Go to E021~E025 unfolding

Refuse to answer →Go to E021~E025 unfolding

< E021~E025. Unfolding bracket questions >

E021. Did it amount to a total of less than, about equal to or more than 600 MW (10,000 Korean won) in the last calendar year (2007)?
[IWER: Go back to E020 and record if R answers the amount during the interview.]

① Less than 600 MW

③ About 600 MW

⑤ More than 600 MW

E022. Did it amount to a total of less than, about equal to, or more than 1,200 MW (10,000 Korean won) in the last calendar year (2007)?
[IWER: Go back to E020 and record if R answers the amount during the interview.]

① Less than 1,200 MW

③ About 1,200 MW

⑤ More than 1,200 MW

E023. Did it amount to a total of less than, about equal to or more than 2,400 MW (10,000 Korean won) in the last calendar year (2007)?
[IWER: Go back to E020 and record if R answers the amount during the interview.]

① Less than 2,400 MW

③ About 2,400 MW

⑤ More than 2,400 MW

E024. Did it amount to a total of less than, about equal to or more than 6,000 MW (10,000 Korean won) in the last calendar year (2007)?
[IWER: Go back to E020 and record if R answers the amount during the interview.]

① Less than 6,000 MW

③ About 6,000 MW

⑤ More than 6,000 MW

E025. Did it amount to a total of less than, about equal to or more than 12,000 MW (10,000 Korean won) in the last calendar year (2007)?
[IWER: Go back to E020 and record if R answers the amount during the interview.]

① Less than 12,000 MW

③ About 12,000 MW

⑤ More than 12,000 MW

- Side job income

E026. Did you earn any income from a side job in the last calendar year (2007)?

① Yes

⑤ No →Go to E033

Don't know →Go to E028~E032 unfolding

Refuse to answer →Go to E028~E032 unfolding

E027. How much did you earn from your side job in the last calendar year (2007)? Please state the amount of income excluding various costs or taxes. (unit: 10,000 Korean Won)

[IWER: In previous interview, side job income: About (PL) 10,000 Korean won]

___MW →Go to E033

Don't know →Go to E028~E032 unfolding

Refuse to answer →Go to E028~E032 unfolding

< E028~E032. unfolding bracket questions >

E028. Did it amount to a total of less than, about equal to or more than 300 MW (10,000 Korean won) in the last calendar year (2007)?

① Less than 300 MW

③ About 300 MW

⑤ More than 300 MW

E029. Did it amount to a total of less than, about equal to or more than 600 MW (10,000 Korean won) in the last calendar year (2007)?

① Less than 600 MW

③ About 600 MW

⑤ More than 600 MW

E030. Did it amount to a total of less than, about equal to or more than 1,200 MW (10,000 Korean won) in the last calendar year (2007)?

① Less than 1,200 MW

③ About 1,200 MW

⑤ More than 1,200 MW

E031. Did it amount to a total of less than, about equal to or more than 2,400 MW (10,000 Korean won) in the last calendar year (2007)?

① Less than 2,400 MW

③ About 2,400 MW

⑤ More than 2,400 MW

E032. Did it amount to a total of less than, about equal to or more than 6,000 MW (10,000 Korean won) in the last calendar year (2007)?

① Less than 6,000 MW

③ About 6,000 MW

⑤ More than 6,000 MW

- The National Pension System Benefit

E033. Did you receive any benefits from the National Pension System in the last calendar year (2007)? If so, did you receive your benefits monthly or in a lump-sum? National pensions include old age pension, disability pension, survivors' pension, and lump-sum death payments.

① Only received monthly benefits

② Only received lump-sum benefits →Go to E036

③ Received both

④ No →Go to E044

E034. Which months did you receive your monthly national pension benefits in the last calendar year (2007)? (Select multiple responses)

① January 2007

② February 2007

③ March 2007

④ April 2007

⑤ May 2007

⑥ June 2007

⑦ July 2007

⑧ August 2007

⑨ September 2007

⑩ October 2007

⑪ November 2007

⑫ December 2007

E035. What was the average amount of national pension benefits received per month in the last calendar year (2007)? (unit: 10,000 Korean won)

[IWER: In previous interview, an average monthly national pension benefit: About (PL) 10,000 Korean won]

___MW [range:1~99997]
Don't know →Go to E038~E042 unfolding

Refuse to answer →Go to E038~E042 unfolding

Logic

■ If E033=①, then go to E037.

■ If E033=② or ③, then go to E036.

E036. What was the total amount of national pension benefits you received in a lump sum in the last calendar year (2007)? (unit: 10,000 Korean won)

[IWER: In previous interview, the total of national pension benefit(a lump sum): About (PL) 10,000 Korean won]

___MW [range:1~99997]
Don't know →Go to E038~E042 unfolding

Refuse to answer →Go to E038~E042 unfolding

E037. So you received a total of ____MW(calculated based on E034, E035, and E036) from the National Pension System in the last calendar year (2007). Is this correct?

[IWER: In previous interview, the total of national pension benefit: About (PL) 10,000 Korean won]

① Yes →Go to E043

⑤ No →Go to E038~E042 unfolding

Don't know →Go to E038~E042 unfolding

Refuse to answer →Go to E038~E042 unfolding

< E038~E042. Unfolding bracket questions >

E038. Did it amount to a total of less than, about equal to or more than 120 MW (10,000 Korean won) in the last calendar year (2007)?
① Less than 120 MW

③ About 120 MW

⑤ More than 120 MW

E039. Did it amount to a total of less than, about equal to or more than 240 MW (10,000 Korean won) in the last calendar year (2007)?
① Less than 240 MW

③ About 240 MW

⑤ More than 240 MW

E040. Did it amount to a total of less than, about equal to or more than 480 MW (10,000 Korean won)

in the last calendar year (2007)?
① Less than 480 MW

③ About 480 MW

⑤ More than 480 MW

E041. Did it amount to a total of less than, about equal to or more than 600 MW (10,000 Korean won) in the last calendar year (2007)?
① Less than 600 MW

③ About 600 MW

⑤ More than 600 MW

E042. Did it amount to a total of less than, about equal to, or more than 1,200 MW (10,000 Korean won) in the last calendar year (2007)?
① Less than 1,200 MW

③ About 1,200 MW

⑤ More than 1,200 MW

E043. When did you start receiving national pension benefits? (unit: year and month combined into 6 digits)

[IWER:

- Enter the year and month using 6 digits. For example, mark 200701 for January 2007. If the month is not clear, enter 200700.
- In previous interview, the time of receiving national pension benefit: (PL) year (PL) month]

______________ [range:198800~200812]

- The Occupational Pension System Benefit

E044. Did you receive any benefits from the Occupational Pension System in the last calendar year (2007)? Occupational pensions includes government employees' pension, military personnel pension, and private school teachers' pension. Did you receive your benefits monthly or in a lump sum?

① Only received monthly benefits

② Only received lump-sum benefits →Go to E047

③ Received both

④ No →Go to E055

E045. Which months did you receive your occupational pension benefits in the last calendar year (2007)? (Select multiple responses)

① January 2007

② February 2007

③ March 2007

④ April 2007

⑤ May 2007

⑥ June 2007

⑦ July 2007

⑧ August 2007

⑨ September 2007

⑩ October 2007

⑪ November 2007

⑫ December 2007

E046. What was the average amount of occupational pension benefits received per month in the last calendar year (2007)? (unit: 10,000 Korean won)
[IWER: In previous interview, an average monthly occupational pension benefit: About (PL) 10,000 Korean won]

___MW [range:1~9997] →Go to E047
Don't know →Go to E049~E053 unfolding

Refuse to answer →Go to E049~E053 unfolding

Logic

■ If E044=①, then go to E048.

■ If E044=② or ③, then go to E047.

E047. What was the total amount of occupational pension benefits you received in a lump sum in the last calendar year (2007)? (unit: 10,000 Korean won)
[IWER: In previous interview, the total of occupational pension benefit(a lump sum): About (PL) 10,000 Korean won]

__________MW [range:1~99997]

Don't know →Go to E049~E053 unfolding

Refuse to answer →Go to E049~E053 unfolding

E048. So you received a total of (amount calculated based on E045, E046, and E047) from the Occupational Pension System in the last calendar year (2007). Is this correct?
[IWER: In previous interview, the total of occupational pension benefit: About (PL) 10,000 Korean won]

① Yes →Go to E054

⑤ No →Go to E049～E053 unfolding

Don't know →Go to E049~E053 unfolding

Refuse to answer →Go to E049~E053 unfolding

< E049~E053. Unfolding bracket questions >

E049. Did it amount to a total of less than, about equal to or more than 300 MW (10,000 Korean won) in the last calendar year (2007)?
① Less than 300 MW

③ About 300 MW

⑤ More than 300 MW

E050. Did it amount to a total of less than, about equal to, or more than 600 MW (10,000 Korean won) in the last calendar year (2007)?
① Less than 600 MW

③ About 600 MW

⑤ More than 600 MW

E051. Did it amount to a total of less than, about equal to or more than 1,200 MW (10,000 Korean won) in the last calendar year (2007)?
① Less than 1,200 MW

③ About 1,200 MW

⑤ More than 1,200 MW

E052. Did it amount to a total of less than, about equal to or more than 2,400 MW (10,000 Korean won) in the last calendar year (2007)?
① Less than 2,400 MW

③ About 2,400 MW

⑤ More than 2,400 MW

E053. Did it amount to a total of less than, about equal to or more than 6,000 MW (10,000 Korean won) in the last calendar year (2007)?
① Less than 6,000 MW

③ About 6,000 MW

⑤ More than 6,000 MW

E054. When did you start receiving occupational pension benefits? (unit: year and month combined into 6 digits)

[IWER:

- Enter the year and month using 6 digits. For example, mark 200701 for January 2007. If the

month is not clear, enter 200700.

- In previous interview, the time of receiving occupational pension benefit: (PL) year (PL) month]

______________ [range:190000~200812]

- Private Pension Benefit

E055. Aside from public pensions, such as national pension and occupational pension, did you receive any benefits from a private pension plan in the last calendar year (2007)?

① Only received monthly benefits

② Only received lump-sum benefits →Go to E058

③ Received both

④ No →Go to E070

E056. Which months did you receive your private pension benefits in the last calendar year (2007)? (Select multiple responses)

① January 2007

② February 2007

③ March 2007

④ April 2007

⑤ May 2007

⑥ June 2007

⑦ July 2007

⑧ August 2007

⑨ September 2007

⑩ October 2007

⑪ November 2007

⑫ December 2007

E057.What was the average amount of private pension benefits received per month in the last calendar year (2007)? (unit: 10,000 Korean won)

[IWER: In previous interview, an average monthly private pension benefit: About (PL) 10,000 Korean won]

______MW [range:1~99997] →Go to E058

Don't know →Go to E060~E064 unfolding

Refuse to answer →Go to E060~E064 unfolding

Logic

■ If E055=①, then go to E059.

■ If E055=② or ③, then go to E058.

E058. What was the total amount of private pension benefits you received in a lump sum in the last calendar year (2007)? (unit: 10,000 Korean won)

[IWER: In previous interview, the total of private pension benefit(a lump sum): About (PL) 10,000 Korean won]

___MW [range:1~99997]
Don't know →Go to E060~E064 unfolding

Refuse to answer →Go to E060~E064 unfolding

E059. So you received a total of (amount calculated based on E056, E057 and E058) in private pension benefits in the last calendar year (2007). Is this correct?

[IWER: In previous interview, the total of private pension benefit: About (PL) 10,000 Korean won]

① Yes →Go to E065

⑤ No →Go to E060~E064 unfolding

Don't know →Go to E060~E064 unfolding

Refuse to answer →Go to E060~E064 unfolding

< E060~E064. Unfolding bracket questions >

E060. Did it amount to a total of less than, about equal to or more than 120 MW (10,000 Korean won) in the last calendar year (2007)?

① Less than 120 MW

③ About 120 MW

⑤ More than 120 MW

E061. Did it amount to a total of less than, about equal to or more than 240 MW (10,000 Korean won) in the last calendar year (2007)?

① Less than 240 MW

③ About 240 MW

⑤ More than 240 MW

E062. Did it amount to a total of less than, about equal to or more than 360 MW (10,000 Korean won) in the last calendar year (2007)?

① Less than 360 MW

③ About 360 MW

⑤ More than 360 MW

E063. Did it amount to a total of less than, about equal to or more than 600 MW (10,000 Korean won) in the last calendar year (2007)?
① Less than 600 MW

③ About 600 MW

⑤ More than 600 MW

E064. Did it amount to a total of less than, about equal to or more than 1,200 MW (10,000 Korean won) in the last calendar year (2007)?
① Less than 1,200 MW

③ About 1,200 MW

⑤ More than 1,200 MW

E065. When did you start receiving private pension benefits? (unit: year and month combined into 6 digits)

[IWER:

- Enter the year and month using 6 digits. For example, mark 200701 for January 2007. If the month is not clear, enter 200700.
- In previous interview, the time of receiving national pension benefit: (PL) year (PL) month]

____________________ [range: 190000~200812]

E066. Will this payment continue as long as you live?

① Yes →Go to E068

⑤ No

E067. How many years will it continue? (unit: a year)

_____years [range:0~997]

E068. If you should die, will the pension benefits stop, stay the same, or be reduced?

① Stop →Go to E070

② Stay the same →Go to E070

③ Continue at a reduced level →Go to E070

④ Others

E069. If others, please specify.

____

- Social Security Benefit

E070. Did you receive any of the following social security benefits in the last calendar year (2007)?

Please choose all that apply. (Select multiple responses, but with 6.)

① Unemployment benefits

② Industrial accident compensation →Go to E081

③ National basic living security benefits →Go to E091

④ Veterans' benefits →Go to E100

⑤ Other welfare benefits →Go to E111

⑥ No →Go to E119

E071. How did you receive unemployment benefits in the last calendar year (2007)? Did you receive monthly benefits, lump-sum benefits, or both? Unemployment benefits include job-seeking benefits, extended benefits, and early reemployment benefits.

① Only received monthly benefits

⑤ Received both

E072. Which months did you receive unemployment benefits in the last calendar year (2007)? (Select multiple responses, but only up to 8 responses can be chosen.)

① January 2007

② February 2007

③ March 2007

④ April 2007

⑤ May 2007

⑥ June 2007

⑦ July 2007

⑧ August 2007

⑨ September 2007

⑩ October 2007

⑪ November 2007

⑫ December 2007

E073.What was the average amount of unemployment benefits received per month in the last calendar year (2007)? (unit: 10,000 Korean won)

[IWER: In previous interview, an average monthly unemployment benefit: About (PL) 10,000 Korean won]

___MW [range:1~99997]

Don't know →Go to E076~E080 unfolding

Refuse to answer →Go to E076~E080 unfolding

Logic

■ If E071=①, then go to E075.

■ If E071=⑤, then go to E074.

E074. What was the total amount of unemployment benefits you received in a lump sum in the last calendar year (2007)? (unit: 10,000 Korean won)

[IWER: In previous interview, the total of unemployment benefit(a lump sum): About (PL) 10,000 Korean won]

___MW [range:1~99997]
Don't know →Go to E076~E080 unfolding

Refuse to answer →Go to E076~E080 unfolding

E075. So you received a total of (amount calculated based on E072, E073 and E074) in unemployment benefits in the last calendar year (2007). Is this correct?

[IWER: In previous interview, the total of unemployment benefit: About (PL) 10,000 Korean won]

① Yes →Go to E081

⑤ No →Go to E076~E080 unfolding

Don't know →Go to E076~E080 unfolding

Refuse to answer →Go to E076~E080 unfolding

< E076~E080. Unfolding bracket questions >

E076. Did it amount to a total of less than, about equal to or more than 100 MW (10,000 Korean won) in the last calendar year (2007)?
① Less than 100 MW

③ About 100 MW

⑤ More than 100 MW

E077. Did it amount to a total of less than, about equal to or more than 200 MW (10,000 Korean won) in the last calendar year (2007)?
① Less than 200 MW

③ About 200 MW

⑤ More than 200 MW

E078. Did it amount to a total of less than, about equal to or more than 400 MW (10,000 Korean won) in the last calendar year (2007)?
① Less than 400 MW

③ About 400 MW

⑤ More than 400 MW

E079. Did it amount to a total of less than, about equal to or more than 600 MW (10,000 Korean won) in the last calendar year (2007)?
① Less than 600 MW

③ About 600 MW

⑤ More than 600 MW

E080. Did it amount to a total of less than, about equal to or more than 800 MW (10,000 Korean won) in the last calendar year (2007)?
① Less than 800 MW

③ About 800 MW

⑤ More than 800 MW

Logic

■ If E070=②, then go to E081.

E081. How did you receive benefits and compensation from the industrial accident compensation insurance in the last calendar year (2007)? Did you receive monthly benefits, lump-sum benefits, or both? Industrial accident compensation includes wage-replacement benefits, disability benefits, and survivors' benefits.

① Only received monthly benefits

② Only received lump-sum benefits →Go to E084

③ Received both

Logic

■ If E081=① or ③, then go to E082.

■ If E081=②, then go to E084.

E082. Which months did you receive industrial accident compensation in the last calendar year (2007)? (Select multiple responses)

① January 2007

② February 2007

③ March 2007

④ April 2007

⑤ May 2007

⑥ June 2007

⑦ July 2007

⑧ August 2007

⑨ September 2007

⑩ October 2007

⑪ November 2007

⑫ December 2007

E083.What was the average amount of industrial accident compensation received per month in the last calendar year (2007)? (unit: 10,000 Korean won)

[IWER: In previous interview, an average monthly industrial accident compensation: About (PL) 10,000 Korean won]

___MW [range:1~99997]

Don't know →Go to E086~E090 unfolding

Refuse to answer →Go to E086~E090 unfolding

Logic

■ If E081=①, then go to E085.

■ If E081=② or ③, then go to E084.

E084. What was the total amount of industrial accident compensation you received in a lump sum in the last calendar year (2007)? (unit: 10,000 Korean won)

[IWER: In previous interview, the total of industrial accident compensation(a lump sum): About (PL) 10,000 Korean won]

___MW [range:1~99997]

Don't know →Go to E086~E090 unfolding

Refuse to answer →Go to E086~E090 unfolding

E085. So you received a total of (amount calculated based on E082, E083, and E084) in industrial accident compensation in the last calendar year (2007). Is this correct?

[IWER: In previous interview, the total of industrial accident compensation: About (PL) 10,000 Korean won]

① Yes →Go to E091

⑤ No →Go to E086~E090 unfolding

Don't know →Go to E086~E090 unfolding

Refuse to answer →Go to E086~E090 unfolding

< E086~E090. Unfolding bracket questions >

E086. Did it amount to a total of less than, about equal to or more than 300 MW (10,000 Korean won) in the last calendar year (2007)?

① Less than 300 MW

③ About 300 MW

⑤ More than 300 MW

E087. Did it amount to a total of less than, about equal to or more than 600 MW (10,000 Korean won) in the last calendar year (2007)?
① Less than 600 MW

③ About 600 MW

⑤ More than 600 MW

E088. Did it amount to a total of less than, about equal to or more than 1,200 MW (10,000 Korean won) in the last calendar year (2007)?
① Less than 1,200 MW

③ About 1,200 MW

⑤ More than 1,200 MW

E089. Did it amount to a total of less than, about equal to or more than 2,400 MW (10,000 Korean won) in the last calendar year (2007)?
① Less than 2,400 MW

③ About 2,400 MW

⑤ More than 2,400 MW

E090. Did it amount to a total of less than, about equal to or more than 6,000 MW (10,000 Korean won) in the last calendar year (2007)?
① Less than 6,000 MW

③ About 6,000 MW

⑤ More than 6,000 MW

Logic

■ If E070=③, then go to E091.

E091. You have said that you received national basic living security benefits (general and self-reliance benefits) in the last calendar year (2007). Which months did you receive national basic living security benefits in the last calendar year (2007)? (Select multiple responses)

① January 2007

② February 2007

③ March 2007

④ April 2007

⑤ May 2007

⑥ June 2007

⑦ July 2007

⑧ August 2007

⑨ September 2007

⑩ October 2007

⑪ November 2007

⑫ December 2007

E092.What was the average amount of national basic living security benefits received per month in the last calendar year (2007)? (unit: 10,000 Korean won)
[IWER: In previous interview, an average monthly national basic living security benefit: About (PL) 10,000 Korean won]

___MW [range:1~99997]
Don't know →Go to E094~E098 unfolding

Refuse to answer →Go to E094~E098 unfolding

E093. So you received a total of (amount calculated based on E091 and E092) in national basic living security benefits in the last calendar year (2007). Is this correct?
[IWER: In previous interview, the total of national basic living security benefit: About (PL) 10,000 Korean won]

① Yes →Go to E099

⑤ No →Go to E094~E098 unfolding

Don't know →Go to E094~E098 unfolding

Refuse to answer →Go to E094~E098 unfolding

< E094~E098. Unfolding bracket questions >

E094. Did it amount to a total of less than, about equal to or more than 120 MW (10,000 Korean won) in the last calendar year (2007)?
① Less than 120 MW

③ About 120 MW

⑤ More than 120 MW

E095. Did it amount to a total of less than, about equal to or more than 240 MW (10,000 Korean won) in the last calendar year (2007)?
① Less than 240 MW

③ About 240 MW

⑤ More than 240 MW

E096. Did it amount to a total of less than, about equal to or more than 480 MW (10,000 Korean won) in the last calendar year (2007)?
① Less than 480 MW

③ About 480 MW

⑤ More than 480 MW

E097. Did it amount to a total of less than, about equal to or more than 600 MW (10,000 Korean won) in the last calendar year (2007)?
① Less than 600 MW

③ About 600 MW

⑤ More than 600 MW

E098. Did it amount to a total of less than, about equal to or more than 1,200 MW (10,000 Korean won) in the last calendar year (2007)?
① Less than 1,200 MW

③ About 1,200 MW

⑤ More than 1,200 MW

E099. When did you start receiving national basic living security benefits? (unit: year and month combined into 6 digits)

[IWER:

- Enter the year and month using 6 digits. For example, mark 200701 for January 2007. If the month is not clear, enter 200700.
- In previous interview, the time of receiving national pension benefit: (PL) year (PL) month]

__________________ [range:190000~200812]

Logic

■ If E070=④, then go to E100.

E100. How did you receive veterans' benefits in the last calendar year (2007)? Did you receive monthly benefits, lump-sum benefits, or both? Veterans' benefits include benefits for patriots and their surviving families, and pensions and nursing allowances for wounded policemen and soldiers.

① Only received monthly benefits

② Only received lump-sum benefits →Go to E103

③ Received both

E101. Which months did you receive veterans' benefits in the last calendar year (2007)? (Select multiple responses)

① January 2007

② February 2007

③ March 2007

④ April 2007

⑤ May 2007

⑥ June 2007

⑦ July 2007

⑧ August 2007

⑨ September 2007

⑩ October 2007

⑪ November 2007

⑫ December 2007

E102. What was the average amount of veterans' benefits received per month in the last calendar year (2007)? (unit: 10,000 Korean won)

[IWER: In previous interview, an average monthly veteran's benefit: About (PL) 10,000 Korean won]

___MW [range:1~99997]
Don't know →Go to E105~E109 unfolding

Refuse to answer →Go to E105~E109 unfolding

Logic

■ If E100=①, then go to E104.

■ If E100=② or ③, then go to E103.

E103. What was the total amount of veterans' benefits you received in a lump sum in the last calendar year (2007)? (unit: 10,000 Korean won)

[IWER: In previous interview, the total of veteran's benefit(a lump sum): About (PL) 10,000 Korean won]

___MW [range:1~99997]
Don't know →Go to E105~E109 unfolding

Refuse to answer →Go to E105~E109 unfolding

E104. So you received a total of (amount calculated based on E101, E102, and E103) in veterans' benefits in the last calendar year (2007). Is this correct?

[IWER: In previous interview, the total of veteran's benefit: About (PL) 10,000 Korean won]

① Yes →Go to E110

⑤ No →Go to E105~E109 unfolding

Don't know →Go to E105~E109 unfolding

Refuse to answer →Go to E105~E109 unfolding

< E105~E109. Unfolding bracket questions >

E105. Did it amount to a total of less than, about equal to or more than 120 MW (10,000 Korean won) in the last calendar year (2007)?
① Less than 120 MW

③ About 120 MW

⑤ More than 120 MW

E106. Did it amount to a total of less than, about equal to or more than 240 MW (10,000 Korean won) in the last calendar year (2007)?
① Less than 240 MW

③ About 240 MW

⑤ More than 240 MW

E107. Did it amount to a total of less than, about equal to or more than 480 MW (10,000 Korean won) in the last calendar year (2007)?
① Less than 480 MW

③ About 480 MW

⑤ More than 480 MW

E108. Did it amount to a total of less than, about equal to or more than 600 MW (10,000 Korean won) in the last calendar year (2007)?
① Less than 600 MW

③ About 600 MW

⑤ More than 600 MW

E109. Did it amount to a total of less than, about equal to or more than 1,200 MW (10,000 Korean won) in the last calendar year (2007)?
① Less than 1,200 MW

③ About 1,200 MW

⑤ More than 1,200 MW

E110. When did you start receiving veterans' benefits? (unit: year and month combined into 6 digits)

[IWER: Enter the year and month using 6 digits. For example, mark 200701 for January 2007. If the month is not clear, enter 200700.]

____________________ [range:190000~200812]

Logic

■ If E070=⑤, then go to E111.

E111. Which months did you receive other welfare benefits in the last calendar year (2009)? (Select multiple responses)

① January 2007

② February 2007

③ March 2007

④ April 2007

⑤ May 2007

⑥ June 2007

⑦ July 2007

⑧ August 2007

⑨ September 2007

⑩ October 2007

⑪ November 2007

⑫ December 2007

E112. What was the average amount of other welfare benefits received per month in the last calendar year (2007)? (unit: 10,000 Korean won)

[IWER: In previous interview, an average monthly other welfare benefit: About (PL) 10,000 Korean won]

___MW [range:1~99997]

Don’t know →Go to E114~E118 unfolding

Refuse to answer →Go to E114~E118 unfolding

E113. So you received a total of (amount calculated based on E111 and E112) in other welfare benefits in the last calendar year (2007). Is this correct?

[IWER: In previous interview, the total of other welfare benefit: About (PL) 10,000 Korean won]

① Yes →go to E119

⑤ No →Go to E114~E118 unfolding

Don’t know →Go to E114~E118 unfolding

Refuse to answer →Go to E114~E118 unfolding

< E114~E118. Unfolding bracket questions >

E114. Did it amount to a total of less than, about equal to or more than 50 MW (10,000 Korean won) in the last calendar year (2007)?

① Less than 50 MW

③ About 50 MW

⑤ More than 50 MW

E115. Did it amount to a total of less than, about equal to or more than 100 MW (10,000 Korean won) in the last calendar year (2007)?
① Less than 100 MW

③ About 100 MW

⑤ More than 100 MW

E116. Did it amount to a total of less than, about equal to or more than 200 MW (10,000 Korean won) in the last calendar year (2007)?
① Less than 200 MW

③ About 200 MW

⑤ More than 200 MW

E117. Did it amount to a total of less than, about equal to or more than 300 MW (10,000 Korean won) in the last calendar year (2007)?
① Less than 300 MW

③ About 300 MW

⑤ More than 300MW

E118. Did it amount to a total of less than, about equal to or more than 600 MW (10,000 Korean won) in the last calendar year (2007)?
① Less than 600 MW

③ About 600 MW

⑤ More than 600 MW

- Other Income

E119. Aside from the income you have talked about so far, did you have any other income in the last calendar year (2007)? Please exclude income from property and financial assets.

[IWER: Other income includes income from alimony and copyright and patent fees. It does not include income from property, house rent, etc., and financial income from bank accounts, securities, bonds, etc. Questions about these sources of income will be asked in ASSETS section.]

① Yes

⑤ No →Go to E126

E120. What was the total amount of other income earned in the last calendar year (2007), after taxes and other deductions? (unit: 10,000 Korean won)

[IWER: In previous interview, other income: About (PL) 10,000 Korean won]

___MW [range:1~99997] →Go to E126
Don't know →Go to E121~E125 unfolding

Refuse to answer →Go to E121~E125 unfolding

< E121~E125. Unfolding bracket questions >

E121. Did it amount to a total of less than, about equal to or more than 120 MW (10,000 Korean won) in the last calendar year (2007)?
[IWER: Go back to E119 and record if R answers the amount during the interview.]

① Less than 120 MW

③ About 120 MW

⑤ More than 120 MW

E122. Did it amount to a total of less than, about equal to or more than 240 MW (10,000 Korean won) in the last calendar year (2007)?
[IWER: Go back to E119 and record if R answers the amount during the interview.]

① Less than 240 MW

③ About 240 MW

⑤ More than 240 MW

E123. Did it amount to a total of less than, about equal to or more than 480 MW (10,000 Korean won) in the last calendar year (2007)?
[IWER: Go back to E119 and record if R answers the amount during the interview.]

① Less than 480 MW

③ About 480 MW

⑤ More than 480 MW

E124. Did it amount to a total of less than, about equal to or more than 600 MW (10,000 Korean won) in the last calendar year (2007)?
[IWER: Go back to E119 and record if R answers the amount during the interview.]

① Less than 600 MW

③ About 600 MW

⑤ More than 600 MW

E125. Did it amount to a total of less than, about equal to or more than 1,200 MW (10,000 Korean won) in the last calendar year (2007)?
[IWER: Go back to E119 and record if R answers the amount during the interview.]

① Less than 1,200 MW

③ About 1,200 MW

⑤ More than 1,200 MW

- Household Income

E126. What was the total amount of income your household members, including you, earned in the

last calendar year (2007) after taxes and other deductions? (unit: 10,000 Korean won)

[IWER: In previous interview, the total of household income: About (PL) 10,000 Korean won]

___MW [range:1~99997] →Go to E132
Don’t know →Go to E127~E131 unfolding

Refuse to answer →Go to E127~E131 unfolding

< E127~E131. Unfolding bracket questions >

E127. Did it amount to a total of less than, about equal to or more than 1,000 MW (10,000 Korean won) in the last calendar year (2007)?
[IWER: Go back to E126 and record if R answers the amount during the interview.]

① Less than 1,000 MW

③ About 1,000 MW

⑤ More than 1,000 MW

E128. Did it amount to a total of less than, about equal to or more than 2,000 MW (10,000 Korean won) in the last calendar year (2007)?
[IWER: Go back to E126 and record if R answers the amount during the interview.]

① Less than 2,000 MW

③ About 2,000 MW

⑤ More than 2,000 MW

E129. Did it amount to a total of less than, about equal to or more than 3,000 MW (10,000 Korean won) in the last calendar year (2007)?
[IWER: Go back to E126 and record if R answers the amount during the interview.]

① Less than 3,000 MW

③ About 3,000 MW

⑤ More than 3,000 MW

E130. Did it amount to a total of less than, about equal to or more than 5,000 MW (10,000 Korean won) in the last calendar year (2007)?
[IWER: Go back to E126 and record if R answers the amount during the interview.]

① Less than 5,000 MW

③ About 5,000 MW

⑤ More than 5,000 MW

E131. Did it amount to a total of less than, about equal to or more than 10,000 MW (10,000 Korean won) in the last calendar year (2007)?
[IWER: Go back to E126 and record if R answers the amount during the interview.]

① Less than 10,000 MW

③ About 10,000 MW
⑤ More than 10,000 MW

E132. [IWER: How often did R receive assistance in answering section E-INCOME?]
① Never →Go to section E2 or F.
② A few times →Go to section E2 or F.
③ Most or all of the times →Go to section E2 or F.
④ The section was done by a proxy respondent.

E133. What is the proxy's relationship to R? If unknown, please ask the proxy. What is your relationship to R?
① Spouse
② Mother
③ Father
④ Mother-in-law
⑤ Father-in-law
⑥ Brother/sister
⑦ Brother-in-law/sister-in-law
⑧ Son/daughter
⑨ Son-in-law/Daughter-in-law
⑩ Grandchild
⑪ Other relatives
⑫ Helper or other non-relatives
→Go to section E2 or F.

■ E2. Consumption

Logic
■ If R has answered CV020, go to E2. Consumption

E201. Now I am going to ask you about your living costs and related items in the last calendar year (2007). Related items include food and dining out, public and private education, housing, medical, and clothing expenditures. First, what was your household's average monthly living cost in the last calendar year (2007)? (unit: 10,000 Korean won)
_____MW [range:0~9997] →Go to E207
Don't know →Go to E202~E206 unfolding
Refuse to answer →Go to E202~E206 unfolding

< E202~E206. Unfolding bracket questions >

E202. Did it amount to a total of less than, about equal to or more than 50 MW (10,000 Korean won) in the last calendar year (2007)?

[IWER: Go back to E201 and record if R answers the amount during the interview.]

① Less than 50 MW

③ About 50 MW

⑤ More than 50 MW

E203. Did it amount to a total of less than, about equal to or more than 100 MW (10,000 Korean won) in the last calendar year (2007)?

[IWER: Go back to E201 and record if R answers the amount during the interview.]

① Less than 100 MW

③ About 100 MW

⑤ More than 100 MW

E204. Did it amount to a total of less than, about equal to or more than 200 MW (10,000 Korean won) in the last calendar year (2007)?

[IWER: Go back to E201 and record if R answers the amount during the interview.]

① Less than 200 MW

③ About 200 MW

⑤ More than 200 MW

E205. Did it amount to a total of less than, about equal to or more than 300 MW (10,000 Korean won) in the last calendar year (2007)?

[IWER: Go back to E201 and record if R answers the amount during the interview.]

① Less than 300 MW

③ About 300 MW

⑤ More than 300 MW

E206. Did it amount to a total of less than, about equal to or more than 500 MW (10,000 Korean won) in the last calendar year (2007)?

[IWER: Go back to E201 and record if R answers the amount during the interview.]

① Less than 500 MW

③ About 500 MW

⑤ More than 500 MW

E207. What was your household's average monthly expenditure on food in the last calendar year (2007)? (unit: 10,000 Korean won)

_____MW [range:0~9997] →Go to E213

Don't know →Go to E208~E212 unfolding

Refuse to answer →Go to E208~E212 unfolding

< E208~E212. Unfolding bracket questions >

E208. Did it amount to a total of less than, about equal to or more than 10 MW (10,000 Korean won) in the last calendar year (2007)?

[IWER: Go back to E207 and record if R answers the amount during the interview.]

① Less than 10 MW

③ About 10 MW

⑤ More than 10 MW

E209. Did it amount to a total of less than, about equal to or more than 30 MW (10,000 Korean won) in the last calendar year (2007)?

[IWER: Go back to E207 and record if R answers the amount during the interview.]

① Less than 30 MW

③ About 30 MW

⑤ More than 30 MW

E210. Did it amount to a total of less than, about equal to or more than 40 MW (10,000 Korean won) in the last calendar year (2007)?

[IWER: Go back to E207 and record if R answers the amount during the interview.]

① Less than 40 MW

③ About 40 MW

⑤ More than 40 MW

E211. Did it amount to a total of less than, about equal to or more than 60 MW (10,000 Korean won) in the last calendar year (2007)?

[IWER: Go back to E207 and record if R answers the amount during the interview.]

① Less than 60 MW

③ About 60 MW

⑤ More than 60 MW

E212. Did it amount to a total of less than, about equal to or more than 100 MW (10,000 Korean won) in the last calendar year (2007)?

[IWER: Go back to E207 and record if R answers the amount during the interview.]

① Less than 100 MW

③ About 100 MW

⑤ More than 100 MW

E213. What was your household's average monthly expenditure on dining out in the last calendar year (2007)? (unit: 10,000 Korean won)

[IWER: Every meal eaten outside of the home is included in the costs of dining out.]

_____MW [range:0~9997] →Go to E219

Don't know →Go to E214~E218 unfolding

Refuse to answer →Go to E214~E218 unfolding

< E214~E218. Unfolding bracket questions >

E214. Did it amount to a total of less than, about equal to or more than 5 MW (10,000 Korean won) in the last calendar year (2007)?

[IWER: Go back to E213 and record if R answers the amount during the interview.]

① Less than 5 MW

③ About 5 MW

⑤ More than 5 MW

E215. Did it amount to a total of less than, about equal to or more than 10 MW (10,000 Korean won) in the last calendar year (2007)?

[IWER: Go back to E213 and record if R answers the amount during the interview.]

① Less than 10 MW

③ About 10 MW

⑤ More than 10 MW

E216. Did it amount to a total of less than, about equal to or more than 20 MW (10,000 Korean won) in the last calendar year (2007)?

[IWER: Go back to E213 and record if R answers the amount during the interview.]

① Less than 20 MW

③ About 20 MW

⑤ More than 20 MW

E217. Did it amount to a total of less than, about equal to or more than 30 MW (10,000 Korean won) in the last calendar year (2007)?

[IWER: Go back to E213 and record if R answers the amount during the interview.]

① Less than 30 MW

③ About 30 MW

⑤ More than 30 MW

E218. Did it amount to a total of less than, about equal to or more than 50 MW (10,000 Korean won) in the last calendar year (2007)?

[IWER: Go back to E213 and record if R answers the amount during the interview.]

① Less than 50 MW

③ About 50 MW

⑤ More than 50 MW

E219. What was your household's average monthly expenditure on public education (tuition, textbooks, materials, etc.) in the last calendar year (2007)? (unit: 10,000 Korean won)

[IWER: Boarding expenses incurred during the course of education are included in housing costs, so record them in E231.]

_____MW [range:0~9997] →Go to E225

Don't know →Go to E220~E224 unfolding

Refuse to answer →Go to E220~E224 unfolding

< E220~E224. Unfolding bracket questions >

E220. Did it amount to a total of less than, about equal to or more than 5 MW (10,000 Korean won) in the last calendar year (2007)?

[IWER: Go back to E219 and record if R answers the amount during the interview.]

① Less than 5 MW

③ About 5 MW

⑤ More than 5 MW

E221. Did it amount to a total of less than, about equal to or more than 10 MW (10,000 Korean won) in the last calendar year (2007)?

[IWER: Go back to E219 and record if R answers the amount during the interview.]

① Less than 10 MW

③ About 10 MW

⑤ More than 10 MW

E222. Did it amount to a total of less than, about equal to or more than 30 MW (10,000 Korean won) in the last calendar year (2007)?

[IWER: Go back to E219 and record if R answers the amount during the interview.]

① Less than 30 MW

③ About 30 MW

⑤ More than 30 MW

E223. Did it amount to a total of less than, about equal to or more than 50 MW (10,000 Korean won) in the last calendar year (2007)?

[IWER: Go back to E219 and record if R answers the amount during the interview.]

① Less than 50 MW

③ About 50 MW

⑤ More than 50 MW

E224. Did it amount to a total of less than, about equal to or more than 100 MW (10,000 Korean won) in the last calendar year (2007)?

[IWER: Go back to E219 and record if R answers the amount during the interview.]

① Less than 100 MW

③ About 100 MW

⑤ More than 100 MW

E225. What was your household's average monthly expenditure on private education (private educational institutions, tutoring, textbooks, materials, etc.) in the last calendar year (2007)? (unit: 10,000 Korean won)

_____MW [range:0~9997] →Go to E231

Don't know →Go to E226~E230 unfolding

Refuse to answer →Go to E226~E230 unfolding

< E226~E230. Unfolding bracket questions >

E226. Did it amount to a total of less than, about equal to or more than 10 MW (10,000 Korean won) in the last calendar year (2007)?

[IWER: Go back to E225 and record if R answers the amount during the interview.]

① Less than 10 MW

③ About 10 MW

⑤ More than 10 MW

E227. Did it amount to a total of less than, about equal to or more than 20 MW (10,000 Korean won) in the last calendar year (2007)?

[IWER: Go back to E225 and record if R answers the amount during the interview.]

① Less than 20 MW

③ About 20 MW

⑤ More than 20 MW

E228. Did it amount to a total of less than, about equal to or more than 50 MW (10,000 Korean won) in the last calendar year (2007)?

[IWER: Go back to E225 and record if R answers the amount during the interview.]

① Less than 50 MW

③ About 50 MW

⑤ More than 50 MW

E229. Did it amount to a total of less than, about equal to or more than 100 MW (10,000 Korean won) in the last calendar year (2007)?

[IWER: Go back to E225 and record if R answers the amount during the interview.]

① Less than 100 MW

③ About 100 MW

⑤ More than 100 MW

E230. Did it amount to a total of less than, about equal to or more than 200 MW (10,000 Korean won) in the last calendar year (2007)?

[IWER: Go back to E225 and record if R answers the amount during the interview.]

① Less than 200 MW

③ About 200 MW

⑤ More than 200 MW

E231. What was your household's average monthly expenditure on housing (monthly rent, utilities including electricity, water and gas, etc.) in the last calendar year (2007)? (unit: 10,000 Korean won)

[IWER: Boarding expenses are included in housing costs.]

_____ MW [range:0~9997] →Go to E237

Don't know →Go to E232~E236 unfolding

Refuse to answer →Go to E232~E236 unfolding

< E232~E236. Unfolding bracket questions >

E232. Did it amount to a total of less than, about equal to or more than 5 MW (10,000 Korean won) in the last calendar year (2007)?

[IWER: Go back to E231 and record if R answers the amount during the interview.]

① Less than 5 MW

③ About 5 MW

⑤ More than 5 MW

E233. Did it amount to a total of less than, about equal to or more than 10 MW (10,000 Korean won) in the last calendar year (2007)?
[IWER: Go back to E231 and record if R answers the amount during the interview.]
① Less than 10 MW
③ About 10 MW
⑤ More than 10 MW

E234. Did it amount to a total of less than, about equal to or more than 20 MW (10,000 Korean won) in the last calendar year (2007)?
[IWER: Go back to E231 and record if R answers the amount during the interview.]
① Less than 20 MW
③ About 20 MW
⑤ More than 20 MW

E235. Did it amount to a total of less than, about equal to or more than 50 MW (10,000 Korean won) in the last calendar year (2007)?
[IWER: Go back to E231 and record if R answers the amount during the interview.]
① Less than 50 MW
③ About 50 MW
⑤ More than 50 MW

E236. Did it amount to a total of less than, about equal to or more than 100 MW (10,000 Korean won) in the last calendar year (2007)?
[IWER: Go back to E231 and record if R answers the amount during the interview.]
① Less than 100 MW
③ About 100 MW
⑤ More than 100 MW

E237. What was your household's average monthly medical expenditure (excluding health insurance premiums) in the last calendar year (2007)? (unit: 10,000 Korean won)
_____MW [range:0~9997] →Go to E243
Don't know →Go to E238~E242 unfolding
Refuse to answer →Go to E238~E242 unfolding

< E238~E242. Unfolding bracket questions >

E238. Did it amount to a total of less than, about equal to or more than 5 MW (10,000 Korean won) in the last calendar year (2007)?

[IWER: Go back to E237 and record if R answers the amount during the interview.]

① Less than 5 MW

③ About 5 MW

⑤ More than 5 MW

E239. Did it amount to a total of less than, about equal to or more than 10 MW (10,000 Korean won) in the last calendar year (2007)?

[IWER: Go back to E237 and record if R answers the amount during the interview.]

① Less than 10 MW

③ About 10 MW

⑤ More than 10 MW

E240. Did it amount to a total of less than, about equal to or more than 20 MW (10,000 Korean won) in the last calendar year (2007)?

[IWER: Go back to E237 and record if R answers the amount during the interview.]

① Less than 20 MW

③ About 20 MW

⑤ More than 20 MW

E241. Did it amount to a total of less than, about equal to or more than 50 MW (10,000 Korean won) in the last calendar year (2007)?

[IWER: Go back to E237 and record if R answers the amount during the interview.]

① Less than 50 MW

③ About 50 MW

⑤ More than 50 MW

E242. Did it amount to a total of less than, about equal to or more than 100 MW (10,000 Korean won) in the last calendar year (2007)?

[IWER: Go back to E237 and record if R answers the amount during the interview.]

① Less than 100 MW

③ About 100 MW

⑤ More than 100 MW

E243. What was your household's average monthly expenditure on clothing (clothes, shoes, etc.) in the last calendar year (2007)? (unit: 10,000 Korean won)

_____MW [range:0~9997] →Go to E249

Don't know →Go to E244~E248 unfolding

Refuse to answer →Go to E244~E248 unfolding

< E244~E248. Unfolding bracket questions >

E244. Did it amount to a total of less than, about equal to or more than 5 MW (10,000 Korean won) in the last calendar year (2007)?

[IWER: Go back to E243 and record if R answers the amount during the interview.]

① Less than 5 MW

③ About 5 MW

⑤ More than 5 MW

E245. Did it amount to a total of less than, about equal to or more than 10 MW (10,000 Korean won) in the last calendar year (2007)?

[IWER: Go back to E243 and record if R answers the amount during the interview.]

① Less than 10 MW

③ About 10 MW

⑤ More than 10 MW

E246. Did it amount to a total of less than, about equal to or more than 20 MW (10,000 Korean won) in the last calendar year (2007)?

[IWER: Go back to E243 and record if R answers the amount during the interview.]

① Less than 20 MW

③ About 20 MW

⑤ More than 20 MW

E247. Did it amount to a total of less than, about equal to or more than 30 MW (10,000 Korean won) in the last calendar year (2007)?

[IWER: Go back to E243 and record if R answers the amount during the interview.]

① Less than 30 MW

③ About 30 MW

⑤ More than 30 MW

E248. Did it amount to a total of less than, about equal to or more than 50 MW (10,000 Korean won) in the last calendar year (2007)?

[IWER: Go back to E243 and record if R answers the amount during the interview.]

① Less than 50 MW

③ About 50 MW

⑤ More than 50 MW

Logic

■ If E126 < E201, then go to E249.

E249. How did you cover your living costs when you didn't have enough money (when living costs exceeded income)? Select three methods in order of priority.

[IWER: Cancelling an insurance policy or withdrawing money from a private savings club earlier than scheduled falls into the category of '⑧ Cancelled savings, deposits or installment savings'. Raising rent falls into the category of '⑥ Sold property or raised house rent'.]

First:_____

Second:_____

Third:_____

① Used bank loans or overdrafts

② Relied on cash advances

③ Borrowed money from relatives

④ Borrowed money from friends and neighbors

⑤ Used private loans

⑥ Sold property or raised house rent

⑦ Reduced housing costs

⑧ Cancelled savings, deposits or installment savings

⑨ Sold financial assets including stocks and bonds

⑩ Sold the car, durables or valuables such as gold and silver

⑪ Others

E250. If others, please specify.

____

- Savings

E251. I am going to ask you about savings. What was your household's average monthly savings in the last calendar year (2007)? Savings include general savings, installment savings, insurance, personal pension and private savings club (Gye). (unit: 10,000 Korean won)

_____MW [range:0~9997] →Go to E257

Don't know →Go to E252~E256 unfolding

Refuse to answer →Go to E252~E256 unfolding

< E252~E256. Unfolding bracket questions >

E252. Did it amount to a total of less than, about equal to or more than 20 MW (10,000 Korean won) in the last calendar year (2007)?

[IWER: Go back to E251 and record if R answers the amount during the interview.]

① Less than 20 MW

③ About 20 MW

⑤ More than 20 MW

E253. Did it amount to a total of less than, about equal to or more than 50 MW (10,000 Korean won) in the last calendar year (2007)?

[IWER: Go back to E251 and record if R answers the amount during the interview.]

① Less than 50 MW

③ About 50 MW

⑤ More than 50 MW

E254. Did it amount to a total of less than, about equal to or more than 100 MW (10,000 Korean won) in the last calendar year (2007)?

[IWER: Go back to E251 and record if R answers the amount during the interview.]

① Less than 100 MW

③ About 100 MW

⑤ More than 100 MW

E255. Did it amount to a total of less than, about equal to or more than 200 MW (10,000 Korean won) in the last calendar year (2007)?

[IWER: Go back to E251 and record if R answers the amount during the interview.]

① Less than 200 MW

③ About 200 MW

⑤ More than 200 MW

E256. Did it amount to a total of less than, about equal to or more than 500 MW (10,000 Korean won) in the last calendar year (2007)?

[IWER: Go back to E251 and record if R answers the amount during the interview.]

① Less than 500 MW

③ About 500 MW

⑤ More than 500 MW

E257. [IWER: How often did R receive assistance in answering section E-INCOME AND CONSUMPTION?]

① Never →Go to section F.

② A few times →Go to section F.

③ Most or all of the time →Go to section F.

④ The section was done by a proxy respondent.

E258. What is the proxy's relationship to R? If unknown, please ask the proxy. What is your relationship to R?

① Spouse

② Mother

③ Father

④ Mother-in-law

⑤ Father-in-law

⑥ Brother/sister

⑦ Brother-in-law/sister-in-law

⑧ Son/daughter

⑨ Son-in-law/Daughter-in-law

⑩ Grandchild

⑪ Other relatives

⑫ Helper or other non-relatives

→Go to section F.

F. ASSETS AND DEBTS

F001. Are you living in your own house or a rented house?

[IWER: "Please tell me what is the tenure type(own house, Deposit-based rental house without monthly rent, monthly rental house with or without a deposit) of the house you are currently living in not under whose name it is." For example, if R is living in his/her child's house (house under the name of his/her child), the tenure type is R's own house.]

① Own house (house owned by R or R's relative) →Go to F004

② Deposit-based rental house without monthly rent (i.e., JEON-SE) →Go to F033

③ Monthly rental house with a deposit (i.e., JEON-WOL-SE) →Go to F041

④ Monthly rental house without a deposit (i.e., WOL-SE) →Go to F049

⑤ Others

F002. If others, please specify.

____

Logic

■ If CV013=⑤ and (response to F001 in the previous interview≠response to F001 in this interview), then go to F003.

- After asking F003, (if F001=①), then go to F004.
- After asking F003, (if F001=②), then go to F033.
- After asking F003, (if F001=③), then go to F041.
- After asking F003, (if F001=④), then go to F049
- After asking F003, (if F001=⑤), then go to F057.

F003. The tenure type ([own house / Deposit-based rental house without monthly rent / monthly rental house with a deposit / monthly rental house without a deposit/others]) of the house you are currently residing in is different from your response in the previous interview. Did the tenure type of your house change after the previous interview?

① Yes

⑤ No

- Housing(own house)

Logic

■ If F001=①, then go to F004.

F004. Is the house you are currently residing in under your name?

① Yes, R's name (including joint ownership)

② No, other's name – spouse →Go to F007

③ No, other's name – (grand)children →Go to F007

④ No, other's name – other family members →Go to F007

⑤ No, other's name – other persons →Go to F007

F005. Is the house you are currently living in is under multiple persons' names? If so, how many names are listed on the house deed? (unit: a person)

[IWER:

- If R's name is also listed, he/she should be counted in. For example, R and his/her spouse are both listed on the house deed, enter 2.

- If the house is not under multiple persons' names, enter 0.]

_____people [range:0~20]

Logic

■ If F005>=2, then go to F006. If not, then go to F007.

F006. Then, how much is your share (of the house you are currently residing in)? (unit: %)

_____% [range:1~99]

F007. What is its present price? What would it bring if it were sold today? (unit: 10,000 Korean won)

______MW [range:0~9999997] →Go to F013

Don't know →Go to F008~F012 unfolding

Refuse to answer →Go to F008~F012 unfolding

<F008~F012. Unfolding bracket questions >

F008. Would it amount to less than, about equal to or more than 1,000 MW (10,000 Korean won)?

[IWER: Go back to F007 and record if R answers the amount during the interview.]

① Less than 1,000 MW

③ About 1,000 MW

⑤ More than 1,000 MW

F009. Would it amount to less than, about equal to, or more than 5,000 MW (10,000 Korean won)?
[IWER: Go back to F007 and record if R answers the amount during the interview.]
① Less than 5,000 MW
③ About 5,000 MW
⑤ More than 5,000 MW

F010. Would it amount to less than, about equal to or more than 10,000 MW (10,000 Korean won)?
[IWER: Go back to F007 and record if R answers the amount during the interview.]
① Less than 10,000 MW
③ About 10,000 MW
⑤ More than 10,000 MW

F011. Would it amount to less than, about equal to or more than 50,000 MW (10,000 Korean won)?
[IWER: Go back to F007 and record if R answers the amount during the interview.]
① Less than 50,000 MW
③ About 50,000 MW
⑤ More than 50,000 MW

F012. Would it amount to less than, about equal to or more than 100,000 MW (10,000 Korean won)?
[IWER: Go back to F007 and record if R answers the amount during the interview.]
① Less than 100,000 MW
③ About 100,000 MW
⑤ More than 100,000 MW

F013. Did you get a loan or incur a debt to purchase the house you are currently living in? How much do you still owe on the loan? (unit: 10,000 Korean won)
[IWER:
- "Please tell me only the amount of loan or debt you have."
- If R has no loan or debt, enter 0.

______MW [range:0~9999997]

F014. Are you (and your spouse) currently leasing parts of your current house to other people? Please choose all that apply.
① Leasing on a deposit basis (i.e., JEON-SE)
② Leasing on a secured monthly rent basis (i.e., JEON-WOL-SE)
③ Leasing on a monthly rent basis (i.e., WOL-SE)

④ Not leasing →Go to F057

Logic

■ If F014=①, ① ②, ① ③, ① ② ③, then go to F015.

F015. How much security deposit of JEON-SE(Deposit-based rental house without monthly rent) did you receive? Please state only the amount of security deposit you received by leasing parts of the house you are currently living in. (unit: 10,000 Korean won)

______MW [range:0~999997] →Go to F021
Don't know →Go to F016~F020 unfolding

[Refuse to answer →Go to F016~F020 unfolding

< F016~F020 Unfolding bracket questions >

F016. Did it amount to less than, about equal to or more than 1,000 MW (10,000 Korean won)?

[IWER: Go back to F015 and record if R answers the amount during the interview.]

① Less than 1,000 MW

③ About 1,000 MW

⑤ More than 1,000 MW

F017. Did it amount to less than, about equal to or more than 3,000 MW (10,000 Korean won)?

[IWER: Go back to F015 and record if R answers the amount during the interview.]

① Less than 3,000 MW

③ About 3,000 MW

⑤ More than 3,000 MW

F018. Did it amount to less than, about equal to or more than 5,000 MW (10,000 Korean won)?

[IWER: Go back to F015 and record if R answers the amount during the interview.]

① Less than 5,000 MW

③ About 5,000 MW

⑤ More than 5,000 MW

F019. Did it amount to less than, about equal to or more than 10,000 MW (10,000 Korean won)?

[IWER: Go back to F015 and record if R answers the amount during the interview.]

① Less than 10,000 MW

③ About 10,000 MW

⑤ More than 10,000 MW

F020. Did it amount to less than, about equal to or more than 30,000 MW (10,000 Korean won)?

[IWER: Go back to F015 and record if R answers the amount during the interview.]

① Less than 30,000 MW

③ About 30,000 MW

⑤ More than 30,000 MW

Logic

■ If F014=②, ① ②, ② ③, ① ② ③, then go to F021. If not, then go to F027.

F021. How much security deposit of JEON-WOL-SE(Monthly rental house with a deposit) did you receive? Please state only the amount of security deposit you received by leasing parts of the house you are currently living in. (unit: 10,000 Korean won).

______MW [range:1~999997]→Go to F027

Don't know →Go to F022~F026 unfolding

Refuse to answer →Go to F022~F026 unfolding

< F022~F026. Unfolding bracket questions >

F022. Did it amount to less than, about equal to or more than 300 MW (10,000 Korean won)?

[IWER: Go back to F021 and record if R answers the amount during the interview.]

① Less than 300 MW

③ About 300 MW

⑤ More than 300 MW

F023. Did it amount to less than, about equal to or more than 500 MW (10,000 Korean won)?

[IWER: Go back to F021 and record if R answers the amount during the interview.]

① Less than 500 MW

③ About 500 MW

⑤ More than 500 MW

F024. Did it amount to less than, about equal to or more than 1,000 MW (10,000 Korean won)?

[IWER: Go back to F021 and record if R answers the amount during the interview.]

① Less than 1,000 MW

③ About 1,000 MW

⑤ More than 1,000 MW

F025. Did it amount to less than, about equal to or more than 5,000 MW (10,000 Korean won)?

[IWER: Go back to F021 and record if R answers the amount during the interview.]

① Less than 5,000 MW

③ About 5,000 MW

⑤ More than 5,000 MW

F026. Did it amount to less than, about equal to or more than 10,000 MW (10,000 Korean won)?

[IWER: Go back to F021 and record if R answers the amount during the interview.]

① Less than 10,000 MW

③ About 10,000 MW

⑤ More than 10,000 MW

Logic

■ If F014=②, ① ②, then go to F027.

■ If F014=③, ① ③, ② ③, ① ② ③, then go to F027. If not, then go to F057.

F027. What is the average amount of rent you receive per month? Please state only the amount of rent you receive from leasing parts of the house you are currently living in. (unit: 10,000 Korean won)

______MW [range:1~999997]→Go to F057

Don't know →Go to F028~F032 unfolding

Refuse to answer →Go to F028~F032 unfolding

< F028~F032. Unfolding bracket questions >

F028. Does it amount to less than, about equal to or more than 10 MW (10,000 Korean won)?

[IWER: Go back to F027 and record if R answers the amount during the interview.]

① Less than 10 MW

③ About 10 MW

⑤ More than 10 MW

F029. Does it amount to less than, about equal to or more than 30 MW (10,000 Korean won)?

[IWER: Go back to F027 and record if R answers the amount during the interview.]

① Less than 30 MW

③ About 30 MW

⑤ More than 30 MW

F030. Does it amount to less than, about equal to or more than 50 MW (10,000 Korean won)?

[IWER: Go back to F027 and record if R answers the amount during the interview.]

① Less than 50 MW

③ About 50 MW

⑤ More than 50 MW

F031. Does it amount to less than, about equal to or more than 100 MW (10,000 Korean won)?

[IWER: Go back to F027 and record if R answers the amount during the interview.]

① Less than 100 MW

③ About 100 MW

⑤ More than 100 MW

F032. Does it amount to less than, about equal to or more than 300 MW (10,000 Korean won)?

[IWER: Go back to F027 and record if R answers the amount during the interview.]

① Less than 300 MW

③ About 300 MW

⑤ More than 300 MW

- Housing(JEON-SE: Deposit-based rental house without monthly rent)

Logic

■ If F001=②, then go to F033.

F033. Is the JEON-SE contract (of the house you are currently residing in) under your name?

① Yes, R's name (including joint ownership)

② No, other's name - spouse →Go to F035

③ No, other's name - (grand)children →Go to F035

④ No, other's name - other family members →Go to F035

⑤ No, other's name - other persons →Go to F035

F034. Is the JEON-SE contract under multiple persons' names? If so, how many names are listed on the contract? (unit: a person)

[IWER:

- If R's name is also listed, he/she should be counted in. For example, R and his/her spouse are both listed on the contract, enter 2.

- If the contract is not under multiple persons' names, enter 0.]

____people [range:0~20]

F035. How much JEON-SE security deposit did you pay on your current house? (unit: 10,000 Korean won)

______MW [range:0~999997]→Go to F057
Don't know →Go to F036~F040 unfolding

Refuse to answer →Go to F036~F040 unfolding

< F036~F040. Unfolding bracket questions >

F036. Did it amount to less than, about equal to or more than 1,000 MW (10,000 Korean won)?

[IWER: Go back to F035 and record if R answers the amount during the interview.]

① Less than 1,000 MW

③ About 1,000 MW

⑤ More than 1,000 MW

F037. Did it amount to less than, about equal to or more than 3,000 MW (10,000 Korean won)?

[IWER: Go back to F035 and record if R answers the amount during the interview.]

① Less than 3,000 MW

③ About 3,000 MW

⑤ More than 3,000 MW

F038. Did it amount to less than, about equal to or more than 5,000 MW (10,000 Korean won)?

[IWER: Go back to F035 and record if R answers the amount during the interview.]

① Less than 5,000 MW

③ About 5,000 MW

⑤ More than 5,000 MW

F039. Did it amount to less than, about equal to or more than 10,000 MW (10,000 Korean won)?

[IWER: Go back to F035 and record if R answers the amount during the interview.]

① Less than 10,000 MW

③ About 10,000 MW

⑤ More than 10,000 MW

F040. Did it amount to less than, about equal to or more than 30,000 MW (10,000 Korean won)?

[IWER: Go back to F035 and record if R answers the amount during the interview.]

① Less than 30,000 MW

③ About 30,000 MW

⑤ More than 30,000 MW

- Housing(WOL-SE: Monthly rental house without a deposit)

Logic

■ If F001=③, then go to F041.

F041. Is the monthly rent contract (of the house you are currently residing in) under your name?

① Yes, R's name (including joint ownership)

② No, other's name – spouse →Go to F043

③ No, other's name – (grand)children →Go to F043

④ No, other's name – other family members →Go to F043

⑤ No, other's name – other persons →Go to F043

F042. Is the monthly rent contract under multiple persons' names? If so, how many names are listed on the contract? (unit: a person)

[IWER:

- If R's name is also listed, he/she should be counted in. For example, R and his/her spouse are both listed on the contract, enter 2.

- If the contract is not under multiple persons' names, enter 0.]

____people [range:0~20]

F043. How much security deposit did you pay on your current house? (unit: 10,000 Korean won)

______MW [range: 1~999997]→Go to F049

Don't know →Go to F044~F048 unfolding

Refuse to answer →Go to F044~F048 unfolding

< F044 ~ F048. Unfolding bracket questions >

F044. Did it amount to less than, about equal to or more than 300 MW (10,000 Korean won)?

[IWER: Go back to F043 and record if R answers the amount during the interview.]

① Less than 300 MW

③ About 300 MW

⑤ More than 300 MW

F045. Did it amount to less than, about equal to or more than 500 MW (10,000 Korean won)?

[IWER: Go back to F043 and record if R answers the amount during the interview.]

① Less than 500 MW

③ About 500 MW

⑤ More than 500 MW

F046. Did it amount to less than, about equal to or more than 1,000 MW (10,000 Korean won)?

[IWER: Go back to F043 and record if R answers the amount during the interview.]

① Less than 1,000 MW

③ About 1,000 MW

⑤ More than 1,000 MW

F047. Did it amount to less than, about equal to or more than 3,000 MW (10,000 Korean won)?

[IWER: Go back to F043 and record if R answers the amount during the interview.]

① Less than 3,000 MW

③ About 3,000 MW

⑤ More than 3,000 MW

F048. Did it amount to less than, about equal to or more than 6,000 MW (10,000 Korean won)?

[IWER: Go back to F043 and record if R answers the amount during the interview.]

① Less than 6,000 MW

③ About 6,000 MW

⑤ More than 6,000 MW

Logic

■ If F001=④, then go to F049.

F049. How much rent do you pay per month? (unit: 10,000 Korean won)

_____MW [range:1~999997] →Go to F055

Don't know →Go to F050~F054 unfolding

Refuse to answer →Go to F050~F054 unfolding

< F050 ~ F054. Unfolding bracket questions >

F050. Does it amount to less than, about equal to or more than 10 MW (10,000 Korean won)?

[IWER: Go back to F049 and record if R answers the amount during the interview.]

① Less than 10 MW

③ About 10 MW

⑤ More than 10 MW

F051. Does it amount to less than, about equal to or more than 30 MW (10,000 Korean won)?

[IWER: Go back to F049 and record if R answers the amount during the interview.]

① Less than 30 MW

③ About 30 MW

⑤ More than 30 MW

F052. Does it amount to less than, about equal to or more than 50 MW (10,000 Korean won)?

[IWER: Go back to F049 and record if R answers the amount during the interview.]

① Less than 50 MW

③ About 50 MW

⑤ More than 50 MW

F053. Does it amount to less than, about equal to or more than 100 MW (10,000 Korean won)?

[IWER: Go back to F049 and record if R answers the amount during the interview.]

① Less than 100 MW

③ About 100 MW

⑤ More than 100 MW

F054. Does it amount to less than, about equal to or more than 300 MW (10,000 Korean won)?

[IWER: Go back to F049 and record if R answers the amount during the interview.]

① Less than 300 MW

③ About 300 MW

⑤ More than 300 MW

Logic

■ If R has moved to another house or the tenure type has changed and the amount reported in the previous interview> the amount reported in this interview(i.e. The amount in this interview is more than 20% below that in the previous interview.), go to F055. If not, then go to F057.

F055. The price of the house you are currently residing in has changed since the previous interview. What did you do with the difference? Please select the one for which you used the largest share of it.

[IWER: If the difference is caused by misreporting in the previous interview, enter '⑤ Others', and then go to F056 and write. After then write on wrong data(previous interview) and new modified data.]

ITEMS	Previous interview(2006)	This interview (2008)
Type of housing tenure	[Own/Lease on a deposit basis without monthly rent(i.e. JEON-SE)/ Monthly rent with a deposit(i.e. JEON-WOL-SE)/Monthly rent without a deposit(i.e. WOL-SE)/Others]	[Own/Lease on a deposit basis without monthly rent(i.e. JEON-SE)/Monthly rent with a deposit(i.e. JEON-WOL-SE)/Monthly rent without a deposit(i.e. WOL-SE)/Other]

Value	[PL1] MW	[PL2] MW
Difference	[PL1-PL2] MW	

① Invested in financial assets or savings accounts →Go to F057

② Invested in other business →Go to F057

③ Used for living expenses →Go to F057

④ Transfer →Go to F057

⑤ Others

F056. If others, please specify.

____

- Property Asset

F057. Do you have any other property assets, except your current house, such as houses or buildings, commercial property, studio apartments (i.e.Officetel), second houses, forest land, land, apartments or Officetel ownership, condominium membership? Please exclude the house you are currently living in.

① Yes

⑤ No →Go to F078

F058. If you sold all those property assets today and then paid off any debts on them, how much would you get? (unit: 10,000 Korean won)

______MW [range:1~9999997]→Go to F064

Don't know →Go to F059~F063 unfolding

Refuse to answer →Go to F059~F063 unfolding

< F059~F063. Unfolding bracket questions >

F059. Would it amount to less than, about equal to or more than 1,000 MW (10,000 Korean won)?

[IWER: Go back to F058 and record if R answers the amount during the interview.]

① Less than 1,000 MW

③ About 1,000 MW

⑤ More than 1,000 MW

F060. Would it amount to less than, about equal to, or more than 5,000 MW (10,000 Korean won)?

[IWER: Go back to F058 and record if R answers the amount during the interview.]

① Less than 5,000 MW

③ About 5,000 MW

⑤ More than 5,000 MW

F061. Would it amount to less than, about equal to or more than 10,000 MW (10,000 Korean won)?

[IWER: Go back to F058 and record if R answers the amount during the interview.]

① Less than 10,000 MW

③ About 10,000 MW

⑤ More than 10,000 MW

F062. Would it amount to less than, about equal to or more than 50,000 MW (10,000 Korean won)?

[IWER: Go back to F058 and record if R answers the amount during the interview.]

① Less than 50,000 MW

③ About 50,000 MW

⑤ More than 50,000 MW

F063. Would it amount to less than, about equal to or more than 100,000 MW (10,000 Korean won)?

[IWER: Go back to F058 and record if R answers the amount during the interview.]

① Less than 100,000 MW

③ About 100,000 MW

⑤ More than 100,000 MW

F064. Are you currently leasing to another person any house or building, second house, studio apartment, forest land, land, etc., except your current house? Please exclude any leasing arrangement of the house you are currently living in.

① Yes

⑤ No →Go to F071

F065. Did you receive any security deposit from the tenant (person who rented the property)? What was the total amount of security deposit you received? (unit: 10,000 Korean won)

[IWER:

- Mark 0 if there is no security deposit.

______MW [range:0~999997] →Go to F071

Don't know →Go to F066~F070 unfolding

Refuse to answer →Go to F066~F070 unfolding

< F066~F070. Unfolding bracket questions >

F066. Did it amount to less than, about equal to or more than 1,000 MW (10,000 Korean won)?

[IWER: Go back to F065 and record if R answers the amount during the interview.]

① Less than 1,000 MW

③ About 1,000 MW

⑤ More than 1,000 MW

F067. Did it amount to less than, about equal to or more than 3,000 MW (10,000 Korean won)?

[IWER: Go back to F065 and record if R answers the amount during the interview.]

① Less than 3,000 MW

③ About 3,000 MW

⑤ More than 3,000 MW

F068. Did it amount to less than, about equal to or more than 5,000 MW (10,000 Korean won)?

[IWER: Go back to F065 and record if R answers the amount during the interview.]

① Less than 5,000 MW

③ About 5,000 MW

⑤ More than 5,000 MW

F069. Did it amount to less than, about equal to or more than 10,000 MW (10,000 Korean won)?

[IWER: Go back to F065 and record if R answers the amount during the interview.]

① Less than 10,000 MW

③ About 10,000 MW

⑤ More than 10,000 MW

F070. Did it amount to less than, about equal to or more than 30,000 MW (10,000 Korean won)?

[IWER: Go back to F065 and record if R answers the amount during the interview.]

① Less than 30,000 MW

③ About 30,000 MW

⑤ More than 30,000 MW

F071. Did you earn any regular income, such as monthly rent or land rent, from your property including the house you were living in in the last calendar year (2007)? Please exclude the security deposits received.

① Yes

⑤ No →Go to F078

F072. What was the total amount of such rental income you earned in the last calendar year (2007) after various costs and deductions? Do not include rental income from the business or farm you owned. Do not include capital gains due to price increase. (unit: 10,000 Korean won)

_____MW [range:0~999997] →Go to F078

Don't know →Go to F073~F077 unfolding

Refuse to answer →Go to F073~F077 unfolding

< F073~F077. Unfolding bracket questions >

F073. Did it amount to less than, about equal to or more than 1,000 MW (10,000 Korean won) in the last calendar year (2007)?

[IWER: Go back to F072 and record if R answers the amount during the interview.]

① Less than 1,000 MW

③ About 1,000 MW

⑤ More than 1,000 MW

F074. Did it amount to less than, about equal to or more than 3,000 MW (10,000 Korean won) in the last calendar year (2007)?

[IWER: Go back to F072 and record if R answers the amount during the interview.]

① Less than 3,000 MW

③ About 3,000 MW

⑤ More than 3,000 MW

F075. Did it amount to less than, about equal to or more than 5,000 MW (10,000 Korean won) in the last calendar year (2007)?

[IWER: Go back to F072 and record if R answers the amount during the interview.]

① Less than 5,000 MW

③ About 5,000 MW

⑤ More than 5,000 MW

F076. Did it amount to less than, about equal to or more than 10,000 MW (10,000 Korean won) in the last calendar year (2007)?

[IWER: Go back to F072 and record if R answers the amount during the interview.]

① Less than 10,000 MW

③ About 10,000 MW

⑤ More than 10,000 MW

F077. Did it amount to less than, about equal to or more than 30,000 MW (10,000 Korean won) in the

last calendar year (2007)?

[IWER: Go back to F072 and record if R answers the amount during the interview.]

① Less than 30,000 MW

③ About 30,000 MW

⑤ More than 30,000 MW

F078. Are you currently renting from another person any house or building, forest land, land, etc., except your current house?

[IWER: Deposit-based/monthly rental for the purpose of study (children's study away from home, etc.) is included.]

① Yes

⑤ No →Go to F085

F079. What was the total amount of security deposit you paid to the landlord for these rental arrangements [both JEON-SE(Deposit-based rental house without monthly rent) and JEON-WOL-SE(Monthly rental house with a deposit)]? (unit: 10,000 Korean won)

[IWER:

- The house R is currently living in is excluded.

______MW [range:1~999997] →Go to F085

Don't know →Go to F080~F084 unfolding

Refuse to answer →Go to F080~F084 unfolding

< F080~F084. Unfolding bracket questions >

F080. Did it amount to less than, about equal to or more than 1,000 MW (10,000 Korean won)?

[IWER: Go back to F079 and record if R answers the amount during the interview.]

① Less than 1,000 MW

③ About 1,000 MW

⑤ More than 1,000 MW

F081. Did it amount to less than, about equal to or more than 3,000 MW (10,000 Korean won)?

[IWER: Go back to F079 and record if R answers the amount during the interview.]

① Less than 3,000 MW

③ About 3,000 MW

⑤ More than 3,000 MW

F082. Did it amount to less than, about equal to or more than 5,000 MW (10,000 Korean won)?

[IWER: Go back to F079 and record if R answers the amount during the interview.]

① Less than 5,000 MW

③ About 5,000 MW

⑤ More than 5,000 MW

F083. Did it amount to less than, about equal to or more than 10,000 MW (10,000 Korean won)?

[IWER: Go back to F079 and record if R answers the amount during the interview.]

① Less than 10,000 MW

③ About 10,000 MW

⑤ More than 10,000 MW

F084. Did it amount to less than, about equal to or more than 30,000 MW (10,000 Korean won)?

[IWER: Go back to F079 and record if R answers the amount during the interview.]

① Less than 30,000 MW

③ About 30,000 MW

⑤ More than 30,000 MW

F085. Do you run or invest in a business or farm?

① Yes

⑤ No →Go to F092

F086.What is the current value of the business or farm you are running? (unit: 10,000 Korean won)

[IWER:

- The property value mentioned earlier is excluded.

______MW [range:0~999997] →Go to F092
Don't know →Go to F087~F091 unfolding

Refuse to answer →Go to F087~F091 unfolding

< F087~F091. Unfolding bracket questions >

F087. Does it amount to less than, about equal to or more than 1,000 MW (10,000 Korean won)?

[IWER: Go back to F086 and record if R answers the amount during the interview.]

① Less than 1,000 MW

③ About 1,000 MW

⑤ More than 1,000 MW

F088. Does it amount to less than, about equal to or more than 5,000 MW (10,000 Korean won)?

[IWER: Go back to F086 and record if R answers the amount during the interview.]

① Less than 5,000 MW

③ About 5,000 MW

⑤ More than 5,000 MW

F089. Does it amount to less than, about equal to or more than 10,000 MW (10,000 Korean won)?

[IWER: Go back to F086 and record if R answers the amount during the interview.]

① Less than 10,000 MW

③ About 10,000 MW

⑤ More than 10,000 MW

F090. Does it amount to less than, about equal to or more than 30,000 MW (10,000 Korean won)?

[IWER: Go back to F086 and record if R answers the amount during the interview.]

① Less than 30,000 MW

③ About 30,000 MW

⑤ More than 30,000 MW

F091. Does it amount to less than, about equal to or more than 50,000 MW (10,000 Korean Won)?

[IWER: Go back to F086 and record if R answers the amount during the interview.]

① Less than 50,000 MW

③ About 50,000 MW

⑤ More than 50,000 MW

Logic

■ If the amount reported in the previous interview>the amount reported in this interview(i.e. The amount in this interview is more than 20% below that in the previous interview.), go to F092. If not, then go to F094.

F092. The price of your property assets other than the house you are currently residing in has changed since the previous interview. What is the reason for the difference? Select one main reason.

[IWER: If the difference is caused by misreporting in the previous interview, enter '⑦ Others', and then go to F093. After then write on wrong data(previous interview) and new modified data.]

Items	Previous interview(____year)	This interview (2008)
Property assets except the house you are living in	[PL1] MW	[F058] MW
Security deposits put down on rental property except the house	[PL2] MW	[F079] MW

you are living in		
Business or Farm	[PL3] MW	[F086] MW
Total	[PL1+PL2+PL3] MW	[F058+F079+F086] MW
Difference	[(PL1+PL2+PL3)-(F058+F079+F086)] MW	

① Sold and invested in financial assets or savings accounts

② Sold and invested in currently occupied property

③ Sold and invested in other business

④ Sold and used for living expenses

⑤ Property price fell

⑥ Transfer

⑦ Others

F093. If others, please specify.

____

- Financial Asset

F094. Next I have some questions about your financial assets. Financial assets include cash over 50 MW (10,000-Korean won), bank savings, stocks / trust funds / mutual funds, bonds, insurance, private money lending, private savings club, etc.

Do you have over 50MW (10,000 Korean won) cash in hand or in checking accounts that yield low interest, but are highly convertible to cash?

① Yes

⑤ No →Go to F101

F095. What is the total amount of such cash and savings? (unit: 10,000 Korean won)

______MW [range:0~999997] →Go to F101

Don't know →Go to F096~F100 unfolding

Refuse to answer →Go to F096~F100 unfolding

< F096~F100. Unfolding bracket questions>

F096. Does it amount to less than, about equal to or more than 100 MW (10,000 Korean won)?

[IWER: Go back to F095 and record if R answers the amount during the interview.]

① Less than 100 MW

③ About 100 MW

⑤ More than 100 MW

F097. Does it amount to less than, about equal to, or more than 500 MW (10,000 Korean won)?

[IWER: Go back to F095 and record if R answers the amount during the interview.]

① Less than 500 MW

③ About 500 MW

⑤ More than 500 MW

F098. Does it amount to less than, about equal to or more than 1,000 MW (10,000 Korean won)?

[IWER: Go back to F095 and record if R answers the amount during the interview.]

① Less than 1,000 MW

③ About 1,000 MW

⑤ More than 1,000 MW

F099. Does it amount to less than, about equal to or more than 5,000 MW (10,000 Korean won)?

[IWER: Go back to F095 and record if R answers the amount during the interview.]

① Less than 5,000 MW

③ About 5,000 MW

⑤ More than 5,000 MW

F100. Does it amount to less than, about equal to or more than 10,000 MW (10,000 Korean won)?

[IWER: Go back to F095 and record if R answers the amount during the interview.]

① Less than 10,000 MW

③ About 10,000 MW

⑤ More than 10,000 MW

F101. Do you have any money in installment savings, mutual savings, certificates of deposits, and other savings accounts that yield relatively high interest, but have less liquidity compared to checking accounts?

① Yes

⑤ No →Go to F108

F102. What is the total amount of such savings? (unit: 10,000 Korean won)

______MW [range:0~9999997]→Go to F108

Don't know →Go to F103~F107 unfolding

Refuse to answer →Go to F103~F107 unfolding

< F103~F107. Unfolding bracket questions >

F103. Does it amount to less than, about equal to or more than 500 MW (10,000 Korean won)?

[IWER: Go back to F102 and record if R answers the amount during the interview.]

① Less than 500 MW

③ About 500 MW

⑤ More than 500 MW

F104. Does it amount to less than, about equal to, or more than 1,000 MW (10,000 Korean won)?

[IWER: Go back to F102 and record if R answers the amount during the interview.]

① Less than 1,000 MW

③ About 1,000 MW

⑤ More than 1,000 MW

F105. Does it amount to less than, about equal to or more than 5,000 MW (10,000 Korean won)?

[IWER: Go back to F102 and record if R answers the amount during the interview.]

① Less than 5,000 MW

③ About 5,000 MW

⑤ More than 5,000 MW

F106. Does it amount to less than, about equal to or more than 10,000 MW (10,000 Korean won)?

[IWER: Go back to F102 and record if R answers the amount during the interview.]

① Less than 10,000 MW

③ About 10,000 MW

⑤ More than 10,000 MW

F107. Does it amount to less than, about equal to or more than 30,000 MW (10,000 Korean won)?

[IWER: Go back to F102 and record if R answers the amount during the interview.]

① Less than 30,000 MW

③ About 30,000 MW

⑤ More than 30,000 MW

F108. Do you have any stocks or investment trust funds (mutual funds)?

① Yes

⑤ No →Go to F115

F109. If you sold all those stocks and mutual funds today, how much would you get? (unit: 10,000 Korean won)

______MW [range:0~999997]→Go to F115
Don't know →Go to F110~F114 unfolding

Refuse to answer →Go to F110~F114 unfolding

< F110~F114. Unfolding bracket questions >

F110. Would it amount to less than, about equal to or more than 500 MW (10,000 Korean won)?

[IWER: Go back to F109 and record if R answers the amount during the interview.]

① Less than 500 MW

③ About 500 MW

⑤ More than 500 MW

F111. Would it amount to less than, about equal to, or more than 1,000 MW (10,000 Korean won)?

[IWER: Go back to F109 and record if R answers the amount during the interview.]

① Less than 1,000 MW

③ About 1,000 MW

⑤ More than 1,000 MW

F112. Would it amount to less than, about equal to or more than 5,000 MW (10,000 Korean won)?

[IWER: Go back to F109 and record if R answers the amount during the interview.]

① Less than 5,000 MW

③ About 5,000 MW

⑤ More than 5,000 MW

F113. Would it amount to less than, about equal to or more than 10,000 MW (10,000 Korean won)?

[IWER: Go back to F109 and record if R answers the amount during the interview.]

① Less than 10,000 MW

③ About 10,000 MW

⑤ More than 10,000 MW

F114. Would it amount to less than, about equal to or more than 30,000 MW (10,000 Korean won)?

[IWER: Go back to F109 and record if R answers the amount during the interview.]

① Less than 30,000 MW

③ About 30,000 MW

⑤ More than 30,000 MW

F115. Do you have any corporate, municipal, government, or foreign bonds?

① Yes

⑤ No →Go to F122

F116. If you sold all those bonds today, how much would you get? (unit: 10,000 Korean won)

______MW [range:1~999997]→Go to F122
Don’t know →Go to F117~F121 unfolding

Refuse to answer →Go to F117~F121 unfolding

< F117~F121. Unfolding bracket questions >

F117. Would it amount to less than, about equal to or more than 500 MW (10,000 Korean won)?

[IWER: Go back to F116 and record if R answers the amount during the interview.]

① Less than 500 MW

③ About 500 MW

⑤ More than 500 MW

F118. Would it amount to less than, about equal to, or more than 1,000 MW (10,000 Korean won)?

[IWER: Go back to F116 and record if R answers the amount during the interview.]

① Less than 1,000 MW

③ About 1,000 MW

⑤ More than 1,000 MW

F119. Would it amount to less than, about equal to or more than 5,000 MW (10,000 Korean won)?

[IWER: Go back to F116 and record if R answers the amount during the interview.]

① Less than 5,000 MW

③ About 5,000 MW

⑤ More than 5,000 MW

F120. Would it amount to less than, about equal to or more than 10,000 MW (10,000 Korean won)?

[IWER: Go back to F116 and record if R answers the amount during the interview.]

① Less than 10,000 MW

③ About 10,000 MW

⑤ More than 10,000 MW

F121. Would it amount to less than, about equal to or more than 30,000 MW (10,000 Korean won)?
[IWER: Go back to F116 and record if R answers the amount during the interview.]
① Less than 30,000 MW
③ About 30,000 MW
⑤ More than 30,000 MW

● Insurance(Term Life, Whole Life, Annuity)

F122. I am going to ask you about insurance. Except the health-related coverage insurance you have talked about earlier, which is intended to insure you against illnesses or accidents, do you have any saving insurance whose aim is to accrue savings and pensions? Saving insurance includes term life insurance, whole life insurance, and annuity insurance.

* POINT*<br>- Term Life Insurance: A type of life insurance which covers the subscriber for a limited period of time.<br>- Whole Life Insurance: A type of life insurance which covers the subscriber until his/her death rather than for a limited period of time.<br>- Annuity Insurance: A type of life insurance in which the subscriber is paid a certain amount each year until his/her death or for a certain period of time.

① Yes
⑤ No →Go to F138

F123. How many term life insurance policies do you have? Term life insurance refers to life insurance which covers the subscriber for a limited period of time.
[IWER: Mark 0 if R has no term life insurance.]
_____number of insurance [range:0~5] * 0(zero) →Go to F127

Logic
■ Loop through F124~F126 as many times as the number of term life insurance policies reported in F123.

F124. How often do you pay insurance premiums for your term life insurance policy?
① Monthly
② Twice a year (every 6 months)
③ Once a year

④ Single payment (full payment)

F125. What is the amount of insurance premiums you pay for your term life insurance policy? (unit: 10,000 Korean won).

[IWER: “Please state the amount you pay in each installment period. If you make a single payment (full payment), please state the full amount.”]

_______MW [range:1~999997]

F126. In what year and month did you take out the term life insurance policy? (unit: year and month combined into 6 digits)

[IWER: Enter the year and month using 6 digits. For example, mark 200701 for January 2007. If the month is not clear, enter 200700.]

__________ [range: 190000~200812]

F127. How many whole life insurance policies do you have? Whole life insurance refers to life insurance which covers the subscriber until his/her death.

[IWER: Mark 0 if R has no whole life insurance.]

_____number of insurance [range:0~5] →Go to F132 ⋆ 0(zero)

Logic

■ **Loop through F128~F131 as many times as the number of whole life insurance policies reported in F127.**

F128. How often do you pay insurance premiums for your whole life insurance policy?

① Monthly

② Twice a year (every 6 months)

③ Once a year

④ Single payment (full payment)

F129. What is the amount of insurance premiums you pay for your whole life insurance policy? (unit: 10,000 Korean won)

[IWER: “Please state the amount you pay in each installment period. If you make a single payment (full payment), please state the full amount.”]

_______MW [range: 1~999997]

F130. In what year and month did you take out the whole life insurance policy? (unit: year and month combined into 6 digits)

[IWER: Enter the year and month using 6 digits. For example, mark 200701 for January 2007. If the month is not clear, enter 200700.]

_________ [range: 190000~200812]

F131. What is the face value of the whole life insurance policy, that is, the amount of money the beneficiary would get if you were to die? (in case of natural death only-not accidental death) (unit: 10,000 Korean won)

______MW [range: 1~999997]

F132. How many annuity insurance policies do you have? Annuity insurance refers to life insurance in which the subscriber is paid a certain amount each year until his/her death or for a certain period of time.

[IWER: Mark 0 if R has no annuity insurance.]

____number of policies [range:0~5] →Go to F138 * 0(zero)

Logic

■ Loop through F133~F137 as many times as the number of annuity insurance policies reported in F132.

F133. How often do you pay insurance premiums for your annuity insurance policy?

① Monthly

② Twice a year (every 6 months)

③ Once a year

④ Single payment (full payment)

F134. What is the amount of insurance premiums you pay for your annuity insurance policy? (unit: 10,000 Korean won)

[IWER: "Please state the amount you pay in each installment period. If you make a single payment (full payment), please state the full amount."]

______MW [range: 1~999997]

F135. In what year and month did you take out the annuity insurance policy? (unit: year and month combined into 6 digits)

[IWER: Enter the year and month using 6 digits. For example, mark 200701 for January 2007. If the month is not clear, enter 200700.]

_________ [range:190000~200812]

F136. At what age do you expect to start receiving annuity payments from your annuity insurance policy?

_____years old [range: 45~65]

F137. What is the average monthly amount of annuity payments you expect to receive from your annuity insurance policy? (unit: 10,000 Korean won)

_______MW [range: 1~999997]

- Lending money

F138. Are you lending money to another person, including family members?

[IWER: Only include money that will be repaid.]

① Yes

⑤ No →Go to F145

F139.What is the total amount of money you are lending? (unit: 10,000 Korean won)

______MW [range: 1~999997] →Go to F145
Don't know →Go to F140~F144 unfolding

Refuse to answer →Go to F140~F144 unfolding

< F140~F144. Unfolding bracket questions >

F140. Does it amount to less than, about equal to or more than 100 MW (10,000 Korean won)?

[IWER: Go back to F139 and record if R answers the amount during the interview.]

① Less than 100 MW

③ About 100 MW

⑤ More than 100 MW

F141. Does it amount to less than, about equal to, or more than 500 MW (10,000 Korean won)?

[IWER: Go back to F139 and record if R answers the amount during the interview.]

① Less than 500 MW

③ About 500 MW

⑤ More than 500 MW

F142. Does it amount to less than, about equal to or more than 1,000 MW (10,000 Korean won)?

[IWER: Go back to F139 and record if R answers the amount during the interview.]

① Less than 1,000 MW

③ About 1,000 MW

⑤ More than 1,000 MW

F143. Does it amount to less than, about equal to or more than 5,000 MW (10,000 Korean won)?
[IWER: Go back to F139 and record if R answers the amount during the interview.]
① Less than 5,000 MW
③ About 5,000 MW
⑤ More than 5,000 MW

F144. Does it amount to less than, about equal to or more than 10,000 MW (10,000 Korean won)?
[IWER: Go back to F139 and record if R answers the amount during the interview.]
① Less than 10,000 MW
③ About 10,000 MW
⑤ More than 10,000 MW

- Private saving club(Gye)

F145. Are you currently a member of any private savings club (Gye)?
① Yes
⑤ No →Go to F158

F146. How much money have you saved through the private savings club (Gye) so far? Exclude the amount that you have already withdrawn. (unit: 10,000 Korean won)
[IWER:
  - If R joins several private savings clubs, enter the total amount.

______MW [range:0~99997] →Go to F152
Don't know →Go to F147~F151 unfolding
Refuse to answer →Go to F147~F151 unfolding

< F147~F151. Unfolding bracket questions >
F147. Does it amount to less than, about equal to or more than 500 MW (10,000 Korean won)?
[IWER: Go back to F146 and record if R answers the amount during the interview.]
① Less than 500 MW
③ About 500 MW
⑤ More than 500 MW

F148. Does it amount to less than, about equal to or more than 1,000 MW (10,000 Korean won)?
[IWER: Go back to F146 and record if R answers the amount during the interview.]

① Less than 1,000 MW

③ About 1,000 MW

⑤ More than 1,000 MW

F149. Does it amount to less than, about equal to or more than 3,000 MW (10,000 Korean won)?

[IWER: Go back to F146 and record if R answers the amount during the interview.]

① Less than 3,000 MW

③ About 3,000 MW

⑤ More than 3,000 MW

F150. Does it amount to less than, about equal to or more than 5,000 MW (10,000 Korean won)?

[IWER: Go back to F146 and record if R answers the amount during the interview.]

① Less than 5,000 MW

③ About 5,000 MW

⑤ More than 5,000 MW

F151. Does it amount to less than, about equal to or more than 10,000 MW (10,000 Korean won)?

[IWER: Go back to F146 and record if R answers the amount during the interview.]

① Less than 10,000 MW

③ About 10,000 MW

⑤ More than 10,000 MW

F152. Have you taken money out of the private savings club (Gye)? And do you still owe some money? If so, how much do you still owe? (unit: 10,000 Korean won)

[IWER:

- If R joins several private savings clubs, enter the total amount.

______MW [range:0~99997] →Go to F158

Don't know →Go to F153~F157 unfolding

Refuse to answer →Go to F153~F157 unfolding

< F153~F157. Unfolding bracket questions >

F153. Does it amount to less than, about equal to or more than 500 MW (10,000 Korean won)?

[IWER: Go back to F152 and record if R answers the amount during the interview.]

① Less than 500 MW

③ About 500 MW

⑤ More than 500 MW

F154. Does it amount to less than, about equal to or more than 1,000 MW (10,000 Korean won)?
[IWER: Go back to F152 and record if R answers the amount during the interview.]
① Less than 1,000 MW
③ About 1,000 MW
⑤ More than 1,000 MW

F155. Does it amount to less than, about equal to or more than 3,000 MW (10,000 Korean won)?
[IWER: Go back to F152 and record if R answers the amount during the interview.]
① Less than 3,000 MW
③ About 3,000 MW
⑤ More than 3,000 MW

F156. Does it amount to less than, about equal to or more than 5,000 MW (10,000 Korean won)?
[IWER: Go back to F152 and record if R answers the amount during the interview.]
① Less than 5,000 MW
③ About 5,000 MW
⑤ More than 5,000 MW

F157. Does it amount to less than, about equal to or more than 10,000 MW (10,000 Korean won)?
[IWER: Go back to F152 and record if R answers the amount during the interview.]
① Less than 10,000 MW
③ About 10,000 MW
⑤ More than 10,000 MW

- Other financial asset

F158. Aside from what you have already told me about, do you have any other financial assets?
① Yes
⑤ No →Go to F166

F159. What types of financial assets are they?
[IWER: R has been asked about ① financial assets (cash and bank deposits, savings deposits, stocks / investment trust funds / mutual funds, bonds), ② insurance (term life insurance, whole life insurance, annuity insurance), ③ money lent to another person, and ④ Gye (private savings club). If R's response to this question falls into any of the four categories mentioned above, go back to F158 and mark '⑤ No'.]

F160. What is the current value of those financial assets? If you sold them today, how much would you get? (unit: 10,000 Korean won)

______MW [range:1~999997]→Go to F166
Don’t know →Go to F161~F165 unfolding

Refuse to answer →Go to F161~F165 unfolding

< F161~F165. Unfolding bracket questions >

F161. Would it amount to less than, about equal to or more than 500 MW (10,000 Korean won)?

[IWER: Go back to F160 and record if R answers the amount during the interview.]

① Less than 500 MW

③ About 500 MW

⑤ More than 500 MW

F162. Would it amount to less than, about equal to, or more than 1,000 MW (10,000 Korean won)?

[IWER: Go back to F160 and record if R answers the amount during the interview.]

① Less than 1,000 MW

③ About 1,000 MW

⑤ More than 1,000 MW

F163. Would it amount to less than, about equal to or more than 3,000 MW (10,000 Korean won)?

[IWER: Go back to F160 and record if R answers the amount during the interview.]

① Less than 3,000 MW

③ About 3,000 MW

⑤ More than 3,000 MW

F164. Would it amount to less than, about equal to or more than 5,000 MW (10,000 Korean won)?

[IWER: Go back to F160 and record if R answers the amount during the interview.]

① Less than 5,000 MW

③ About 5,000 MW

⑤ More than 5,000 MW

F165. Would it amount to less than, about equal to or more than 10,000 MW (10,000 Korean won)?

[IWER: Go back to F160 and record if R answers the amount during the interview.]

① Less than 10,000 MW

③ About 10,000 MW

⑤ More than 10,000 MW

F166. During the last calendar year (2007), how much interest per month did you earn from the financial assets you have mentioned so far? (unit: 10,000 Korean won)

[IWER: the financial assets are cash (more than 50 MW), saving (high interest rate but limited deposit and withdrawal money), stock or investment trust, bonds, deposit insurance, lending money, Gye(private saving club, other financial asset etc..]

______MW [range:0~99997] →Go to F172

Don't know →Go to F167~F171 unfolding

Refuse to answer →Go to F167~F171 unfolding

< F167~F171. Unfolding bracket questions >.

F167. Did it amount to less than, about equal to or more than 50 MW (10,000 Korean won) in the last calendar year (2007)?

[IWER: Go back to F166 and record if R answers the amount during the interview.]

① Less than 50 MW

③ About 50 MW

⑤ More than 50 MW

F168. Did it amount to less than, about equal to or more than 100 MW (10,000 Korean won) in the last calendar year (2007)?

[IWER: Go back to F166 and record if R answers the amount during the interview.]

① Less than 100 MW

③ About 100 MW

⑤ More than 100 MW

F169. Did it amount to less than, about equal to or more than 300 MW (10,000 Korean won) in the last calendar year (2007)?

[IWER: Go back to F166 and record if R answers the amount during the interview.]

① Less than 300 MW

③ About 300 MW

⑤ More than 300 MW

F170. Did it amount to less than, about equal to or more than 500 MW (10,000 Korean won) in the last calendar year (2007)?

[IWER: Go back to F166 and record if R answers the amount during the interview.]

① Less than 500 MW

③ About 500 MW

⑤ More than 500 MW

F171. Did it amount to less than, about equal to or more than 1,000 MW (10,000 Korean won) in the last calendar year (2007)?
[IWER: Go back to F166 and record if R answers the amount during the interview.]

① Less than 1,000 MW

③ About 1,000 MW

⑤ More than 1,000 MW

Logic

■ If the amount reported in the previous interview>the amount reported in this interview(i.e. The amount in this interview is more than 20% below that in the previous interview.), go to F172. If not, then go to F174.

F172. The total amount of your financial assets has changed since the previous interview. What is the reason for the difference? Select one main reason.
[IWER: If the difference is caused by misreporting in the previous interview, enter '⑦ Others', and then go to F173. After then write on wrong data(previous interview) and new modified data]

ITEMS	previous interview	This interview (2008)
Cash or savings	[PL1] MW	[F095] MW
Savings or installment savings	[PL2] MW	[F102] MW
Stocks and investment trust funds (mutual funds)	[PL3] MW	[F109] MW
Bonds	[PL4] MW	[F116] MW
Money lent to another person	[PL5] MW	[F139] MW
Gye(private savings club)	[PL6] MW	[F146] MW
Other financial assets	[PL7] MW	[F160] MW
Total	[(PL1+⋯+PL7)] MW	[(F095+F102+⋯+F160)] MW
Difference	[(PL1+⋯+PL7)−(F095+F102+⋯+F160)] MW	

① Sold and invested in other financial assets or savings accounts →Go to F174

② Sold and invested in property →Go to F174

③ Sold and invested in other business →Go to F174

④ Sold and used for living expenses →Go to F174

⑤ Change of rate of return change →Go to F174

⑥ Transfer →Go to F174

⑦ Others

F173. If others, please specify.

____

- **Other assets**

F174. Do you own any vehicles, such as cars, trucks, trailers and boats?

① Yes

⑤ No →Go to F181

F175. If you sold all the vehicles you own today, how much would you get? (unit: 10,000 Korean won)

______MW [range: 1~999997]→Go to F181

Don’t know →Go to F176~F180 unfolding

Refuse to answer →Go to F176~F180 unfolding

< F176~F180. Unfolding bracket questions >

F176. Would it amount to less than, about equal to or more than 500 MW (10,000 Korean won)?

[IWER: Go back to F175 and record if R answers the amount during the interview.]

① Less than 500 MW

③ About 500 MW

⑤ More than 500 MW

F177. Would it amount to less than, about equal to or more than 1,000 MW (10,000 Korean won)?

[IWER: Go back to F175 and record if R answers the amount during the interview.]

① Less than 1,000 MW

③ About 1,000 MW

⑤ More than 1,000 MW

F178. Would it amount to less than, about equal to or more than 3,000 MW (10,000 Korean won)?

[IWER: Go back to F175 and record if R answers the amount during the interview.]

① Less than 3,000 MW

③ About 3,000 MW

⑤ More than 3,000 MW

F179. Would it amount to less than, about equal to or more than 5,000 MW (10,000 Korean won)?

[IWER: Go back to F175 and record if R answers the amount during the interview.]

① Less than 5,000 MW

③ About 5,000 MW

⑤ More than 5,000 MW

F180. Would it amount to less than, about equal to or more than 10,000 MW (10,000 Korean won)?

[IWER: Go back to F175 and record if R answers the amount during the interview.]

① Less than 10,000 MW

③ About 10,000 MW

⑤ More than 10,000 MW

F181. Aside from the vehicles, etc., you have already told me about, do you own any other non-financial assets, such as valuables, paintings, antiques and golf membership?

① Yes

⑤ No →Go to F188

F182. What is the total value of those assets? (unit: 10,000 Korean won)

______MW [range: 1~999997]→Go to F188
Don't know →Go to F183~F187 unfolding

Refuse to answer →Go to F183~F187 unfolding

< F183~F187. Unfolding bracket questions >

F183. Does it amount to less than, about equal to or more than 500 MW (10,000 Korean won)?

[IWER: Go back to F182 and record if R answers the amount during the interview.]

① Less than 500 MW

③ About 500 MW

⑤ More than 500 MW

F184. Does it amount to less than, about equal to or more than 1,000 MW (10,000 Korean won)?

[IWER: Go back to F182 and record if R answers the amount during the interview.]

① Less than 1,000 MW

③ About 1,000 MW

⑤ More than 1,000 MW

F185. Does it amount to less than, about equal to or more than 3,000 MW (10,000 Korean won)?

[IWER: Go back to F182 and record if R answers the amount during the interview.]

① Less than 3,000 MW

③ About 3,000 MW

⑤ More than 3,000 MW

F186. Does it amount to less than, about equal to or more than 5,000 MW (10,000 Korean won)?

[IWER: Go back to F182 and record if R answers the amount during the interview.]

① Less than 5,000 MW

③ About 5,000 MW

⑤ More than 5,000 MW

F187. Does it amount to less than, about equal to or more than 10,000 MW (10,000 Korean won)?

[IWER: Go back to F182 and record if R answers the amount during the interview.]

① Less than 10,000 MW

③ About 10,000 MW

⑤ More than 10,000 MW

- Inheritance/Donation

F188. Have you ever received any inheritance or donation in the form of property, financial assets, etc.?

[IWER:

- If R has ever received even a small amount of inheritance or donation, mark ① Yes.

- For example, they are property, cash or financial assets, insurance money, pension benefits, etc.]

① Yes

⑤ No →Go to F199

F189. Among the inheritances you have received, please tell me the one with the largest present value (including those that are not currently owned by you.). In what form did you receive it?

① Insurance money →Go to F191

② Pension benefits →Go to F191

③ Property →Go to F191

④ Cash or financial assets →Go to F191

⑤ Others

F190. If others, please specify.

F191. From whom did you receive that inheritance [F189 or F190] ? Please specify the relationship of the giver to you?

① Spouse

② Mother

③ Father

④ Mother-in-law

⑤ Father-in-law

⑥ Brother/sister

⑦ Brother-in-law/sister-in-law

⑧ Son/daughter

⑨ Son-in-law/Daughter-in-law

⑩ Grandchild

⑪ Other relatives

⑫ Other individuals

F192. When did you receive the inheritance [F189 or F190]? (unit: year and month combined into 6 digits)

[IWER: Enter the year and month using 6 digits. For example, mark 200701 for January 2007. If the month is not clear, enter 200700.]

_____ [range: 190000~200812]

F193. What was the value of the inheritance at the time you received it? Please specify the amount. (unit: 10,000 Korean won)

______MW [range: 1~999997]→Go to F199

Don't know →Go to F194~F198 unfolding

Refuse to answer →Go to F194~F198 unfolding

< F194~F198. Unfolding bracket questions >

F194. Did it amount to less than, about equal to or more than 1,000 MW (10,000 Korean won)?

[IWER: Go back to F193 and record if R answers the amount during the interview.]

① Less than 1,000 MW

③ About 1,000 MW

⑤ More than 1,000 MW

F195. Did it amount to less than, about equal to or more than 3,000 MW (10,000 Korean won)?

[IWER: Go back to F193 and record if R answers the amount during the interview.]

① Less than 3,000 MW

③ About 3,000 MW

⑤ More than 3,000 MW

F196. Did it amount to less than, about equal to or more than 5,000 MW (10,000 Korean won)?

[IWER: Go back to F193 and record if R answers the amount during the interview.]

① Less than 5,000 MW

③ About 5,000 MW

⑤ More than 5,000 MW

F197. Did it amount to less than, about equal to or more than 10,000 MW (10,000 Korean won)?

[IWER: Go back to F193 and record if R answers the amount during the interview.]

① Less than 10,000 MW

③ About 10,000 MW

⑤ More than 10,000 MW

F198. Did it amount to less than, about equal to or more than 30,000 MW (10,000 Korean won)?

[IWER: Go back to F193 and record if R answers the amount during the interview.]

① Less than 30,000 MW

③ About 30,000 MW

⑤ More than 30,000 MW

F242. Including what you have mentioned earlier, what do you think is the present value of all the inheritances you've received? (unit: 10,000 Korean won)

______MW [range: 1~999997] →Go to F199
Don’t know →Go to F243~F247 unfolding

Refuse to answer →Go to F243~F247 unfolding

< F243~F247. Unfolding bracket questions >

F243. Would it amount to less than, about equal to or more than 1,000 MW (10,000 Korean won)?

[IWER: Go back to F242 and record if R answers the amount during the interview.]

① Less than 1,000 MW

③ About 1,000 MW

⑤ More than 1,000 MW

F244. Would it amount to less than, about equal to, or more than 3,000 MW (10,000 Korean won)?

[IWER: Go back to F242 and record if R answers the amount during the interview.]

① Less than 3,000 MW

③ About 3,000 MW

⑤ More than 3,000 MW

F245. Would it amount to less than, about equal to, or more than 5,000 MW (10,000 Korean won)?

[IWER: Go back to F242 and record if R answers the amount during the interview.]

① Less than 5,000 MW

③ About 5,000 MW

⑤ More than 5,000 MW

F246. Would it amount to less than, about equal to or more than 10,000 MW (10,000 Korean won)?

[IWER: Go back to F242 and record if R answers the amount during the interview.]

① Less than 10,000 MW

③ About 10,000 MW

⑤ More than 10,000 MW

F247. Would it amount to less than, about equal to or more than 30,000 MW (10,000 Korean won)?

[IWER: Go back to F242 and record if R answers the amount during the interview.]

① Less than 30,000 MW

③ About 30,000 MW

⑤ More than 30,000 MW

- Debt

F199. Next are questions about debts. Please respond to each debt category that applies to you. Debts include loans from financial institutions, credit card loans and cash advances, personal borrowings, suretyship obligations, and other debts. First, do you have any loans from financial institution, such as banks, insurance companies, securities companies, or credit card companies?

[IWER: The loan R has taken for financing his/her current house is excluded.]

① Yes

⑤ No →Go to F206

F200. What is the total amount of your loans from financial institutions? (unit: 10,000 Korean won)

______MW [range: 1~999997]→Go to F206

Don't know →Go to F201~F205 unfolding

Refuse to answer →Go to F201~F205 unfolding

< F201~F205. Unfolding bracket questions >

F201. Does it amount to less than, about equal to or more than 1,000 MW (10,000 Korean won)?

[IWER: Go back to F200 and record if R answers the amount during the interview.]

① Less than 1,000 MW

③ About 1,000 MW

⑤ More than 1,000 MW

F202. Does it amount to less than, about equal to or more than 3,000 MW (10,000 Korean won)?

[IWER: Go back to F200 and record if R answers the amount during the interview.]

① Less than 3,000 MW

③ About 3,000 MW

⑤ More than 3,000 MW

F203. Does it amount to less than, about equal to or more than 5,000 MW (10,000 Korean won)?

[IWER: Go back to F200 and record if R answers the amount during the interview.]

① Less than 5,000 MW

③ About 5,000 MW

⑤ More than 5,000 MW

F204. Does it amount to less than, about equal to or more than 10,000 MW (10,000 Korean won)?

[IWER: Go back to F200 and record if R answers the amount during the interview.]

① Less than 10,000 MW

③ About 10,000 MW

⑤ More than 10,000 MW

F205. Does it amount to less than, about equal to or more than 30,000 MW (10,000 Korean won)?

[IWER: Go back to F200 and record if R answers the amount during the interview.]

① Less than 30,000 MW

③ About 30,000 MW

⑤ More than 30,000 MW

F206. Aside from the loans from financial institutions or credit card companies that you have already told me, do you have any borrowings from relatives, friends or others?

① Yes

⑤ No →Go to F213

F207. What is the total amount of such borrowings? (unit: 10,000 Korean won)

______MW [range:1~99997]→Go to F213
Don't know →Go to F208~F212 unfolding

Refuse to answer →Go to F208~F212 unfolding

< F208~F212. Unfolding bracket questions >

F208. Does it amount to less than, about equal to or more than 300 MW (10,000 Korean won)?

[IWER: Go back to F207 and record if R answers the amount during the interview.]

① Less than 300 MW

③ About 300 MW

⑤ More than 300 MW

F209. Does it amount to less than, about equal to or more than 500 MW (10,000 Korean won)?

[IWER: Go back to F207 and record if R answers the amount during the interview.]

① Less than 500 MW

③ About 500 MW

⑤ More than 500 MW

F210. Does it amount to less than, about equal to or more than 1,000 MW (10,000 Korean won)?

[IWER: Go back to F207 and record if R answers the amount during the interview.]

① Less than 1,000 MW

③ About 1,000 MW

⑤ More than 1,000 MW

F211. Does it amount to less than, about equal to or more than 3,000 MW (10,000 Korean won)?

[IWER: Go back to F207 and record if R answers the amount during the interview.]

① Less than 3,000 MW

③ About 3,000 MW

⑤ More than 3,000 MW

F212. Does it amount to less than, about equal to or more than 6,000 MW (10,000 Korean won)?

[IWER: Go back to F207 and record if R answers the amount during the interview.]

① Less than 6,000 MW

③ About 6,000 MW

⑤ More than 6,000 MW

F213. And do you currently stand surety for the debts of your relative, friend, or others?

① Yes

⑤ No →Go to F220

F214.What is the total amount of the debts guaranteed by you? (unit: 10,000 Korean won)

______MW [range:0~99997]→Go to F220
Don't know →Go to F215~F219 unfolding

Refuse to answer →Go to F215~F219 unfolding

< F215~F219. Unfolding bracket questions >

F215. Does it amount to less than, about equal to or more than 500 MW (10,000 Korean won)?

[IWER: Go back to F214 and record if R answers the amount during the interview.]

① Less than 500 MW

③ About 500 MW

⑤ More than 500 MW

F216. Does it amount to less than, about equal to or more than 1,000 MW (10,000 Korean won)?

[IWER: Go back to F214 and record if R answers the amount during the interview.]

① Less than 1,000 MW

③ About 1,000 MW

⑤ More than 1,000 MW

F217. Does it amount to less than, about equal to or more than 3,000 MW (10,000 Korean won)?

[IWER: Go back to F214 and record if R answers the amount during the interview.]

① Less than 3,000 MW

③ About 3,000 MW

⑤ More than 3,000 MW

F218. Does it amount to less than, about equal to or more than 5,000 MW (10,000 Korean won)?

[IWER: Go back to F214 and record if R answers the amount during the interview.]

① Less than 5,000 MW

③ About 5,000 MW

⑤ More than 5,000 MW

F219. Does it amount to less than, about equal to or more than 10,000 MW (10,000 Korean won)?

[IWER: Go back to F214 and record if R answers the amount during the interview.]

① Less than 10,000 MW

③ About 10,000 MW

⑤ More than 10,000 MW

F220. Aside from the debt categories mentioned above, do you have any other debts?

① Yes

⑤ No →Go to F228

F221. What types of debts are they?

[IWER: R has been asked about ① loans from financial institutions, ② borrowings from relatives, friends or others, and ③ surety for the debts of relatives, friends, or others. If R's response to this question falls into any of the three debt categories mentioned above, go back to F220 and mark '⑤ No.']

____

F222. What is the total amount of your other debts? (unit: 10,000 Korean won)

______MW [range: 1~99997]→Go to F228
Don't know →Go to F223~F227 unfolding

Refuse to answer →Go to F223~F227 unfolding

< F223~F227. Unfolding bracket questions >

F223. Does it amount to less than, about equal to or more than 300 MW (10,000 Korean won)?

[IWER: Go back to F222 and record if R answers the amount during the interview.]

① Less than 300 MW

③ About 300 MW

⑤ More than 300 MW

F224. Does it amount to less than, about equal to or more than 500 MW (10,000 Korean won)?

[IWER: Go back to F222 and record if R answers the amount during the interview.]

① Less than 500 MW

③ About 500 MW

⑤ More than 500 MW

F225. Does it amount to less than, about equal to or more than 1,000 MW (10,000 Korean won)?

[IWER: Go back to F222 and record if R answers the amount during the interview.]

① Less than 1,000 MW

③ About 1,000 MW

⑤ More than 1,000 MW

F226. Does it amount to less than, about equal to or more than 3,000 MW (10,000 Korean won)?

[IWER: Go back to F222 and record if R answers the amount during the interview.]

① Less than 3,000 MW

③ About 3,000 MW

⑤ More than 3,000 MW

F227. Does it amount to less than, about equal to or more than 6,000 MW (10,000 Korean won)?

[IWER: Go back to F222 and record if R answers the amount during the interview.]

① Less than 6,000 MW

③ About 6,000 MW

⑤ More than 6,000 MW

- Household Asset

F228. What is the total amount of assets your household members, including you, have? (unit: 10,000 Korean won)

______MW [range:0~999997]→Go to F234
Don't know →Go to F229~F233 unfolding

Refuse to answer →Go to F229~F233 unfolding

< F229~F233. Unfolding bracket questions >

F229. Does it amount to less than, about equal to or more than 1,000 MW (10,000 Korean won)?

[IWER: Go back to F228 and record if R answers the amount during the interview.]

① Less than 1,000 MW

③ About 1,000 MW

⑤ More than 1,000 MW

F230. Does it amount to less than, about equal to or more than 5,000 MW (10,000 Korean won)?

[IWER: Go back to F228 and record if R answers the amount during the interview.]

① Less than 5,000 MW

③ About 5,000 MW

⑤ More than 5,000 MW

F231. Does it amount to less than, about equal to or more than 10,000 MW (10,000 Korean won)?

[IWER: Go back to F228 and record if R answers the amount during the interview.]

① Less than 10,000 MW

③ About 10,000 MW

⑤ More than 10,000 MW

F232. Does it amount to less than, about equal to or more than 50,000 MW (10,000 Korean won)?

[IWER: Go back to F228 and record if R answers the amount during the interview.]

① Less than 50,000 MW

③ About 50,000 MW

⑤ More than 50,000 MW

F233. Does it amount to less than, about equal to or more than 100,000 MW (10,000 Korean won)?

[IWER: Go back to F228 and record if R answers the amount during the interview.]

① Less than 100,000 MW

③ About 100,000 MW

⑤ More than 100,000 MW

- Household debt

F234. What is the total amount of debts your household members, including you, have? (unit: 10,000 Korean won)

______MW [range:0~999997]→Go to F240
Don't know →Go to F235~F239 unfolding

Refuse to answer →Go to F235~F239 unfolding

< F235~F239. Unfolding bracket questions >

F235. Does it amount to less than, about equal to or more than 500 MW (10,000 Korean won)?

[IWER: Go back to F234 and record if R answers the amount during the interview.]

① Less than 500 MW

③ About 500 MW

⑤ More than 500 MW

F236. Does it amount to less than, about equal to or more than 3,000 MW (10,000 Korean won)?

[IWER: Go back to F234 and record if R answers the amount during the interview.]

① Less than 3,000 MW

③ About 3,000 MW

⑤ More than 3,000 MW

F237. Does it amount to less than, about equal to or more than 15,000 MW (10,000 Korean won)?
[IWER: Go back to F234 and record if R answers the amount during the interview.]
① Less than 15,000 MW
③ About 15,000 MW
⑤ More than 15,000 MW

F238. Does it amount to less than, about equal to or more than 50,000 MW (10,000 Korean won)?
[IWER: Go back to F234 and record if R answers the amount during the interview.]
① Less than 50,000 MW
③ About 50,000 MW
⑤ More than 50,000 MW

F239. Does it amount to less than, about equal to or more than 100,000 MW (10,000 Korean won)?
[IWER: Go back to F234 and record if R answers the amount during the interview.]
① Less than 100,000 MW
③ About 100,000 MW
⑤ More than 100,000 MW

F240. [IWER: How often did R receive assistance in answering section F-ASSETS AND DEBTS?]
① Never →Go to section G.
② A few times →Go to section G.
③ Most or all of the time →Go to section G.
④ The section was done by a proxy respondent.

F241. What is the proxy's relationship to R? If unknown, please ask the proxy. What is your relationship to R?
① Spouse
② Mother
③ Father
④ Mother-in-law
⑤ Father-in-law
⑥ Brother/sister
⑦ Brother-in-law/sister-in-law
⑧ Son/daughter
⑨ Son-in-law/Daughter-in-law
⑩ Grandchild

⑪ Other relatives

⑫ Helper or other non-relatives

→Go to section G.

G. EXPECTATIONS AND LIFE SATISFACTION

- The National Pension System and Occupational Pension System

Logic

■ (D135(if R is a wage worker, whether the national pension system enrolled or not)=③ or ④) or (E033(whether R received benefits from the national pension system or not)=④ and E044(whether R received benefits from the occupational pension system or not)=④)

G001. Are you currently enrolled in the National Pension System or the Occupational Pension System (for public servants, teachers, soldiers, and postal workers) and paying insurance premiums?

⓪ Receiving public pension benefits from this year →Go to G006

① Currently enrolled and paying premiums →Go to G006

② Currently enrolled but officially granted a waiver of premiums

③ Currently enrolled but not paying premiums

④ Not enrolled or not applicable (Never paid premiums) →Go to G004

G002. What is the reason you have been granted a waiver of premiums or are not paying premiums despite being enrolled in the National Pension System or the Occupational Pension System?

① Unemployment or discontinued business →Go to G006

② Lack of income →Go to G006

③ Lack of trust in the National Pension System or the Occupational Pension System →Go to G006

④ Others

G003. Please specify the reason.

______________________________ →Go to G006

G004. Why are you not enrolled?

① Old age →Go to G010

② Never involved in income-earning activities →Go to G010

③ Recipient of other social security benefits such as national basic living security benefits and veterans' benefits →Go to G010

④ Lack of income →Go to G010

⑤ Others

G005. Please specify the reason.

______________________________ →Go to G010

G006. How long have you paid premiums?

[IWER: “Please state the period of contribution.”]

① Less than 1 year

② More than 1 year and less than 5 years

③ More than 5 years and less than 10 years

④ More than 10 years and less than 20 years

⑤ More than 20 years

G007. When do you start receiving national pension benefits or occupational pension benefits?

① 55 - 59 years old

② 60 - 64 years old

③ 65 - 69 years old

④ Over 70 years old

G008. If you receive national pension benefits or occupational pension benefits from the government, how much do you expect to receive per month? Please answer in terms of their present value. (Unit: 10,000 Korean won)

_____MW [range:1~500]

G009. Have you ever received information on the amount of pension benefits from the Pension Corporation or checked it on the internet?

① Yes

⑤ No

- The Basic Old-Age Pension System

G053. Now I am going to ask you about the Basic Old-Age Pension System. The Basic Old-Age Pension System was newly established in 2008, and is currently supporting approximately 70% of the population over the age of 65 by paying them up to 13 MW per person to cover their basic living expenses. Have you applied for the Basic Old-Age Pension System?

[IWER: if R applied for 2 years, mark last year. For instance, if R applied both in 2007 and 2008, enter “[3] Yes, I applied in 2008”

① Yes, I applied in 2007

③ Yes, I applied in 2008

⑤ No, I haven't applied →Go to G057

G054. If so, are you receiving basic old-age pension benefits?

① Yes, currently receiving

③ No, but will receive upon reaching the pension age (birth month)

⑤ No, not entitled

G055. How much are you receiving per month?

[IWER:

- Enter 10,000 won unit first and then 1,000 won unit. In case of 'Don't know/Refuse to answer', only 10,000 won unit can be entered, and the minimum amount of benefits is 20,000 won.

__________ Won [range: 20,000~140,000]

G056. Are the basic old-age pension benefits provided to you alone or to you and your spouse together?

① Provided to the individual

⑤ Provided to the couple

G057. What is the reason for not applying for the Basic Old-Age Pension System?

① Didn't think I would get it →Go to G059

③ Didn't know about it (application period, eligibility requirements, etc.) →Go to G059

⑤ Others

G058. If others, please specify.

____

G059. Do you have any plan to apply for the Basic Old-Age Pension System?

① Yes

⑤ No

- Inheritance I: Leaving inheritance

G010. Next I am going to ask your opinion about how likely you think various events are to happen. When I ask a question I'd like you to give me a number from 0 to 100, where "0" means that you think the event will never happen, and "100" means that you think it will surely happen. If it's hard to

answer in numbers, take a look at the picture I show you and answer in accordance to that.
"Considering all the property and other valuables that I own, I am likely to leave an inheritance totaling 10,000 MW (10,000 Korean won) or more."
[IWER: "Please indicate how much you agree to the above statement using the scale below." 0 means absolutely no chance and 100 means absolutely certain.]

0 10 20 30 40 50 60 70 80 90 100

- Inheritance II: Receiving inheritance

Logic

■ If at least one of R's parents is alive, go to G011. Otherwise, go to G012.

G011. I am likely to receive an inheritance totaling 10,000 MW (10,000 Korean Won) or more.
[IWER: "Please indicate how much you agree to the above statement using the scale below." 0 means absolutely no chance and 100 means absolutely certain.]

0 10 20 30 40 50 60 70 80 90 100

- Working Expectation by Age

Logic

■ If R is aged 45~49 and currently working, go to G012. Otherwise, go to G013.

G012. I am likely to continue working at my present job until the age of 55.
[IWER: "Please indicate how much you agree to the above statement using the scale below." 0 means absolutely no chance and 100 means absolutely certain.]

0 10 20 30 40 50 60 70 80 90 100

Logic

■ If R is aged 50~54 and currently working, go to G013. Otherwise, go to G014.

G013. I am likely to continue working at my present job until the age of 60.

[IWER: “Please indicate how much you agree to the above statement using the scale below.” 0 means absolutely no chance and 100 means absolutely certain.]

0 10 20 30 40 50 60 70 80 90 100

Logic

■ If R is aged 55 or over and currently working, go to G014. Otherwise, go to G015.

G014. I am likely to be working at my present job for the next 5 years.

[IWER: “Please indicate how much you agree to the above statement using the scale below.” 0 means absolutely no chance and 100 means absolutely certain.]

0 10 20 30 40 50 60 70 80 90 100

Logic

■ If R is not currently working, go to G015. Otherwise, go to G016.

G015. I am likely to be working for pay at some time in the future.

[IWER: “Please indicate how much you agree to the above statement using the scale below.” 0 means absolutely no chance and 100 means absolutely certain.]

0 10 20 30 40 50 60 70 80 90 100

- Desired Life Time by Age

Logic

■ If R is aged 64 or under, go to G016. Otherwise go to G017.

G016. I am likely to live to be 75 or more.

[IWER: “Please indicate how much you agree to the above statement using the scale below.” 0 means absolutely no chance and 100 means absolutely certain.]

0 10 20 30 40 50 60 70 80 90 100

Logic

■ If R is aged 65~69, go to G017. Otherwise go to G018.

G017. I am likely to live to be 80 or more.

[IWER: "Please indicate how much you agree to the above statement using the scale below." 0 means absolutely no chance and 100 means absolutely certain.]

0 10 20 30 40 50 60 70 80 90 100

Logic

■ If R is aged 70~74, go to G018. Otherwise go to G019.

G018. I am likely to live to be 85 or more.

[IWER: "Please indicate how much you agree to the above statement using the scale below." 0 means absolutely no chance and 100 means absolutely certain.]

0 10 20 30 40 50 60 70 80 90 100

Logic

■ If R is aged 75~79, go to G019. Otherwise go to G020.

G019. I am likely to live to be 90 or more.

[IWER: "Please indicate how much you agree to the above statement using the scale below." 0 means absolutely no chance and 100 means absolutely certain.]

0 10 20 30 40 50 60 70 80 90 100

Logic

■ If R is aged 80~84, go to G020. Otherwise go to G021.

G020. I am likely to live to be 95 or more.

[IWER: “Please indicate how much you agree to the above statement using the scale below.” 0 means absolutely no chance and 100 means absolutely certain.]

0 10 20 30 40 50 60 70 80 90 100

Logic

■ If R is aged 85~94, go to G021. Otherwise go to G022.

G021. I am likely to live to be 100 or more.

[IWER: “Please indicate how much you agree to the above statement using the scale below.” 0 means absolutely no chance and 100 means absolutely certain.]

0 10 20 30 40 50 60 70 80 90 100

Logic

■ If R aged 95~99, go to G022. Otherwise go to G023.

G022. I am likely to live to be 105 or more.

[IWER: “Please indicate how much you agree to the above statement using the scale below.” 0 means absolutely no chance and 100 means absolutely certain.]

0 10 20 30 40 50 60 70 80 90 100

Logic

■ If R is aged 100 or over, go to G023. Otherwise go to G024.

G023. I am likely to live to be 110 or more.

[IWER: “Please indicate how much you agree to the above statement using the scale below.” 0 means absolutely no chance and 100 means absolutely certain.]

0 10 20 30 40 50 60 70 80 90 100

- Expectation about the Standard of living and the Government (Silver Life, the Unification of North Korean and South Korean, Real Estate)

G024. My living standards are likely to be lower.

[IWER: “Please indicate how much you agree to the above statement using the scale below.” 0 means absolutely no chance and 100 means absolutely certain.]

0 10 20 30 40 50 60 70 80 90 100

G025. Our children’s generation is likely to live in a better economic/social situation than our generation.

[IWER: “Please indicate how much you agree to the above statement using the scale below.” 0 means absolutely no chance and 100 means absolutely certain.]

0 10 20 30 40 50 60 70 80 90 100

G026. The government is likely to provide security for my life in old age.

[IWER: “Please indicate how much you agree to the above statement using the scale below.” 0 means absolutely no chance and 100 means absolutely certain.]

0 10 20 30 40 50 60 70 80 90 100

G027. The National Pension System is likely to help stabilize my life in old age.

[IWER: “Please indicate how much you agree to the above statement using the scale below.” 0 means absolutely no chance and 100 means absolutely certain.]

0 10 20 30 40 50 60 70 80 90 100

G028. The Basic Old-Age Pension System is likely to help stabilize my life in old age.

**The Basic Old-Age Pension System** was newly established in 2008, and is currently supporting approximately 70% of the population over the age of 65. A single receiver can be supported up to 9 MW and a couple of receiver can be supported up to 15MW to cover their basic living expenses.

[IWER: “Please indicate how much you agree to the above statement using the scale below.” 0 means absolutely no chance and 100 means absolutely certain.]

0 10 20 30 40 50 60 70 80 90 100

G029. The National Health Insurance System is likely to help stabilize my life in old age.

[IWER: “Please indicate how much you agree to the above statement using the scale below.” 0 means absolutely no chance and 100 means absolutely certain.]

0 10 20 30 40 50 60 70 80 90 100

G030. The Elderly Long-term Care Insurance System is likely to help stabilize my life in old age.

‘The Elderly Long-term Care Insurance System was newly introduced in 2008, and provides long-term care for the elderly at home or nursing care facilities, who have difficulty getting around.

[IWER: “Please indicate how much you agree to the above statement using the scale below.” 0 means absolutely no chance and 100 means absolutely certain.]

0 10 20 30 40 50 60 70 80 90 100

G031. South and North Koreas are likely to be united in the next 10 years.

[IWER: “Please indicate how much you agree to the above statement using the scale below.” 0 means absolutely no chance and 100 means absolutely certain.]

0 10 20 30 40 50 60 70 80 90 100

G032. The Korean economy is likely to face a serious downturn sometime in the next 10 years.
[IWER: “Please indicate how much you agree to the above statement using the scale below.” 0 means absolutely no chance and 100 means absolutely certain.]

0 10 20 30 40 50 60 70 80 90 100

G033. Korea’s property market is likely to stabilize in the next 10 years.
[IWER: “Please indicate how much you agree to the above statement using the scale below.” 0 means absolutely no chance and 100 means absolutely certain.]

0 10 20 30 40 50 60 70 80 90 100

G034. Suppose that you were given 1,000 MW (10,000-Korean won). What would you do with that money? Please answer after looking at the following list. You can spend all that money on one item.

ITEMS	AMOUNT
1. Savings or investment	
2. Pay debt	
3. Give it to children or relatives or donate it to society	
4. Buy a house, car, furniture, electric appliances, etc.	
5. Travel and other leisure activities	
Total<br>(The sum must be equal to1,000 MW (10,000-Korean won))	1,000 MW<br>(10,000 Korean won)

- Quality of Life(QOL)

G035. Now I’m going to ask you about life satisfaction. Please answer how much you are satisfied with the following compared to your contemporaries. How satisfied are you with your health?
[IWER: 0 means absolutely dissatisfied and 100 means absolutely satisfied.]

0 10 20 30 40 50 60 70 80 90 100

G036. How satisfied are you with your economic status?

[IWER: 0 means absolutely dissatisfied and 100 means absolutely satisfied.]

0 10 20 30 40 50 60 70 80 90 100

Logic

■ If R has a spouse, then go to G037

G037. How satisfied are you with your relationship with your spouse?

[IWER: 0 means absolutely dissatisfied and 100 means absolutely satisfied.]

0 10 20 30 40 50 60 70 80 90 100

Logic

■ If R has answered that he/she has living children, then go to G038.

G038. How satisfied are you with your relationship with your children?

[IWER: 0 means absolutely dissatisfied and 100 means absolutely satisfied.]

0 10 20 30 40 50 60 70 80 90 100

G039. How satisfied are you with your overall quality of life (or how happy you feel)?

[IWER: 0 means absolutely dissatisfied and 100 means absolutely satisfied.]

0 10 20 30 40 50 60 70 80 90 100

G040. What do you think is your socioeconomic status?

① Top of high

② Bottom of high

③ Top of middle

④ Bottom of middle

⑤ Top of low

⑥ Bottom of low

G041. How much pocket money do you spend each month? (unit: 10,000 Korean won)

Average_____MW per month [range:0~997]

G042. Have you taken a journey, tour or picnic in the past one year? If so, approximately how many times? (unit: number)

[IWER: If there is none, enter 0.]

_____times in the past one year [range:0~100]

G043. How much did you pay out of pocket for these activities? (unit: 10,000 Korean won)

[IWER: If there is none, enter 0.]

_____MW in the past one year [range:0~9997]

G044. Have you been to cinemas, theatres, concerts, art galleries, or sports events in the past one year? If so, approximately how many times? (unit: number)

[IWER: If there is none, enter 0.]

_____times in past one year [range:0~100]

G045. How much did you pay out of pocket for these activities? (unit: 10,000 Korean won)

[IWER: If there is none, enter 0.]

_____MW in past one year [range:0~9997]

G046. Have you participated in hobby or recreation related activities or programs in the past one year? If so, approximately how many hours per month? Please state the average number of hours per month. (unit: hours)

[IWER: If there is none, enter 0.]

Average_____hours per month [range:0~200]

G047. How much did you pay out of pocket for these activities? Please state the average amount of costs per month. (unit: 10,000 Korean won)
[IWER: If there is none, enter 0.]
Average_____MW per month [range:0~997]

G048. Have you participated in education programs for individual development in the past one year? If so, approximately how many hours per month? Please state the average number of hours per month. (unit: hours)
[IWER: If there is none, enter 0.]
Average_____hours per month [range:0~200]

G049. How much did you pay out of pocket for these activities? Please say the average amount of costs per month. (unit: 10,000 Korean won)
[IWER: If there is none, enter 0.]
Average_____MW per month [range:0~997]

G050. Have you participated in volunteer activities in the past one year? If so, approximately how many hours per month? (unit: hours)
[IWER: If there is none, enter 0.]
Average_____hours per month [range:0~200]

G051. [IWER: How often did R receive assistance in answering section G- EXPECTATIONS AND LIFE SATISFACTION?]
① Never → the end of the interview
② A few times → the end of the interview
③ Most or all of the time → the end of the interview
④ The section was done by a proxy respondent.

G052. What is the proxy's relationship to R? If unknown, please ask the proxy. What is your relationship to R?
① Spouse
② Mother
③ Father
④ Mother-in-law
⑤ Father-in-law
⑥ Brother/sister
⑦ Brother-in-law/sister-in-law

⑧ Son/daughter

⑨ Son-in-law/Daughter-in-law

⑩ Grandchild

⑪ Other relatives

⑪ Helper or other non-relatives

→ The end of the interview

2008 KLoSA

Wave 2 Questionnaire

EXIT INTERVIEW

TABLE OF CONTENTS

- Xa. INFORMATION ABOUT RESPONDENT/ DEATH ------------------------- 313
- Xc. HEALTH -- 316
- Xd. EMPLOYMENT -- 346
- Xf. ASSETS AND DEBTS --- 354

Xa. INFORMATION ABOUT RESPONDENT/ DEATH

Xa001. I have some questions about you. What is your name?

____

Xa002. What is your relationship to the person who died?

① Spouse

② Mother

③ Father

④ Mother-in-law

⑤ Father-in-law

⑥ Brother/sister

⑦ Brother-in-law/sister-in-law

⑧ Son/daughter

⑨ Son-in-law/Daughter-in-law

⑩ Grandchild

⑪ Other relatives

⑫ Friend

⑬ Helper or other non-relatives →Go to Xa004

Logic

■ If Xa002≠⑬, then go to Xa003.

Xa003. (If R is a friend or relative) How long did you know him/her?

__________years [range:1~120]

Xa004. How often did you meet him/her during the one year before he/she died?

① Almost every day (more than 4 times per week)

② Once a week

③ 2~3 times a week

④ Once a month

⑤ Twice a month (every two weeks)

⑥ Once or twice a year

⑦ Three or four times a year (once every three or four months)

⑧ Five or six times a year (every two months)

⑨ Almost never a year

⑩ Never

Xa005. I am going to ask you about [his/her name]'s death. How long was he/she sick before he/she died?

① (He/she) was not sick, but died suddenly.

② Less than one day

③ 1day~1week

④ 1week~1month

⑤ 1 month~6 months

⑥ 6 months~1 year

⑦ 1 year~5 years

⑧ More than 5 years

Xa006. Was his/her death expected?

① Unexpected

③ Somewhat expected because he/she was sick, but it came suddenly.

⑤ Expected

Xa007. What was the main cause of his/her death according to the death certificate?

____

Xa008. What was the manner of his/her death?

① Death from an illness →Go to Xa010

③ Violent death

⑤ Others or unknown →GO to Xa010

Xa009. Which of the following best describes his/her violent death?

① Traffic accident

② Accidental poisoning

③ Accidental fall

④ Accidental drowning

⑤ Suicide

⑥ Homicide

⑦ Other accidental deaths

Xa010. What is the date of his/her death?

____year ____month ____day

Xa011. Where did he/she die?

① At home →Go to section Xc.

③ At his/her child's or relative's house →Go to section Xc.

⑤ In a hospital →Go to section Xc.

⑦ at a nursing home/elderly care facility →Go to section Xc.

⑨ Others

Xa012. If others, please specify.

__________________ →Go to section Xc.

Xc. HEALTH

Xc101. What was the chronic disease he/she was suffering from when he/she died? Please choose all that apply. (Select multiple responses.)

① Stroke →Go to Xc103

② Hypertension →Go to Xc103

③ Diabetes →Go to Xc103

④ Cancer →Go to Xc103

⑤ Lung disease →Go to Xc103

⑥ Heart disease →Go to Xc103

⑦ Liver disease →Go to Xc103

⑧ Dementia or memory impairment →Go to Xc103

⑨ Mental disease (depression) →Go to Xc103

⑩ Fracture or aftereffects of traffic accident →Go to Xc103

⑪ Arthritis →Go to Xc103

⑫ Others

⑬ None →Go to Xc103

Xc102. If others, please specify.

____

Xc103. Did he/she use an oxygen mask just before he/she died?

① Yes

⑤ No

Logic

■ If he/she was reported to receive a diagnosis of cancer in the previous interview and Xc104=④, then go toXc104~Xc109. If not, go to Xc110.

Xc104. You have said that he/she was suffering from cancer. Did he/she go to the hospital for cancer treatment after the previous interview?

① Yes

⑤ No →Go to Xc108

Xc105. Did he/she receive cancer treatment, such as anticancer drug, surgery and radiation therapy, after the previous interview?

① Yes

⑤ No →Go to Xc108

Xc106. What kinds of cancer treatment did he/she receive from a doctor in the hospital? Please choose all that apply. (Select multiple responses.)

① Anticancer injections or anticancer drugs →Go to Xc108

② Surgery or biopsy →Go to Xc108

③ Radiation therapy →Go to Xc108

④ Drugs other than anticancer drugs (medicine to relieve pain, vomiting and rash) →Go to Xc108

⑤ Didn't receive treatment →Go to Xc108

⑥ Others

Xc107. If others, please specify.

____

Xc108. In which body part was he/she last found to develop cancer?

[IWER: Record directly the site of cancer, and then enter the relevant code using the table below. R can choose a maximum of three answers.]

<CODE TABLE >

01	Malignant neoplasm of lip, oral cavity and pharynx
02	Malignant neoplasm of esophagus
03	Malignant neoplasm of stomach
04	Malignant neoplasm of colon
05	Malignant neoplasm of anal canal, rectum and anus
06	Malignant neoplasm of liver and intrahepatic bile ducts
07	Malignant neoplasm of pancreas
08	Malignant neoplasm of other digestive organs
09	Malignant neoplasm of larynx
10	Malignant neoplasm of trachea, bronchus and lung

11	Malignant neoplasm of other sites in the respiratory system and intrahoracic organs
12	Malignant neoplasm of bone and articular cartilage
13	Malignant neoplasm of skin
14	Malignant neoplasm of breast
15	Malignant neoplasm of cervix uteri
16	Malignant neoplasm of other uterus parts
17	Malignant neoplasm of prostate
18	Malignant neoplasm of bladder
19	Malignant neoplasm of ureter
20	Malignant neoplasm of eyes and adnexa
21	Malignant neoplasm of brain
22	Malignant neoplasm of other parts of the central nervous system
23	Malignant neoplasm of other, ill-defined, secondary, unspecified and multiple sites
24	Hodgkin lymphoma
25	Non-hodgkin lymphoma
26	Leukemia
27	Malignant neoplasm of other lymph and related tissues
28	Others

NO	Site of cancer	CODE
1		
2		
3		

Don't know →Go to Xc110

Refuse to answer →Go to Xc110

Xc109. When was the last cancer site found? (unit: year and month combined into 6 digits)
[IWER: Enter the year and month using 6 digits. For example, mark 200701 for January 2007. If the month is not clear, enter 200700.]
_______________ [range: 200600~200812]

Logic
■ **If he/she was reported to receive a diagnosis of chronic lung disease in the previous interview and Xc101=⑤, then go to Xc110~Xc111. If not, go to Xc112.**

Xc110. You have said that he/she was suffering from chronic lung disease (chronic bronchitis, emphysema, chronic obstructive pulmonary disease, etc). Did he/she receive a diagnosis of chronic lung disease from a doctor?
① Yes
⑤ No →Go to Xc112

Xc111. When did he/she receive his/her most recent diagnosis of chronic lung disease? (unit: year and month combined into 6 digits)
[IWER: Enter the year and month using 6 digits. For example, mark 200701 for January 2007. If the month is not clear, enter 200700.]
_________ [range: 200600~200812]

Logic
■ **If he/she was reported to receive a diagnosis of cardiovascular disease in the previous interview and Xc101=⑥, then go to Xc112~Xc117. If not, go to Xc118.**

Xc112. You have said that he/she was suffering from heart disease (coronary artery disease, angina pectoris, congestive heart failure). Did he/she receive a diagnosis of heart disease from a doctor?
① Yes
⑤ No →Go to Xc114

Xc113.When did he/she receive his/her most recent diagnosis of heart disease? (unit: year and month combined into 6 digits)
[IWER: Enter the year and month using 6 digits. For example, mark 200701 for January 2007. If the month is not clear, enter 200700.]
_________ [range: 200600~200812]

Xc114. Did he/she ever have a heart attack, cardiac arrest or myocardial infarction after the previous interview?

① Yes

⑤ No →Go to Xc116

Xc115. When did he/she have his/her most recent heart attack, cardiac arrest or myocardial infarction? (unit: year and month combined into 6 digits)

[IWER: Enter the year and month using 6 digits. For example, mark 200701 for January 2007. If the month is not clear, enter 200700.]

_________ [range: 200600~200812]

Xc116. Did he/she ever have a heart surgery after the previous interview?

① Yes

⑤ No

Xc117. Did he/she ever receive special treatment because of his/her heart problems after the previous interview?

[IWER: Special treatment refers to arterial or venous catheter insertion surgery, angiography, cardiovascular transplantation surgery, etc.]

① Yes

⑤ No

Logic

■ **If he/she was reported to receive a diagnosis of a stroke in the previous interview and Xc101=①, then go to Xc118~Xc121.If not, go to Xc201.**

Xc118. You have said that he/she was suffering from a stroke. Did he/she receive a diagnosis of a stroke from a doctor?

① Yes

⑤ No →Go to Xc120

Xc119. When did he/she receive his/her most recent diagnosis of a stroke? (unit: year and month combined into 6 digits)

[IWER: Enter the year and month using 6 digits. For example, mark 200701 for January 2007. If the month is not clear, enter 200700.]

_________ [range: 200600~200812]

Xc120. Did he/she ever have a stroke after the previous interview?

① Yes

⑤ No →Go to Xc201

Xc121. When did he/she have his/her most recent stroke? (unit: year and month combined into 6 digits)

[IWER: Enter the year and month using 6 digits. For example, mark 200701 for January 2007. If the month is not clear, enter 200700.]

__________ [range: 200600~200812] →Go to Xc201

Xc201. Now I have some questions about cognitive function. Did he/she have memory problems (cognitive dysfunction) during the one month before his/her death?

① Yes

⑤ No →Go to Xc205

Logic

■ If Xc201=①, then go to Xc202~Xc204.

Xc202. When did his/her memory problems begin? (unit: an age)

______age [range:45~130]

Xc203. Did he/she receive a diagnosis of memory problems (memory impairment, dementia) from a doctor?

① Yes

⑤ No

Xc204. Did he/she take any prescription medication due to memory problems?

① Yes

⑤ No

Xc205. How was his/her memory during the one month before his/her death?

① Very good

③ Good

⑤ Moderate

⑦ Bad

⑨ Very bad

Xc206. Now I have some questions about problem behaviors and cognitive functions. Did he/she have the following symptoms during the two years before his/her death?
[Problem behavior] He/she got lost in the place he/she knew well.
① Yes
⑤ No

Xc207. [Problem behavior] He/she wandered off the street, without finding way back home.
① Yes
⑤ No

Xc208. [Problem behavior] He/she could not be left alone at home.
① Yes
⑤ No

Xc209. [Problem behavior] He/she had auditory hallucinations or hallucinations.
① Yes
⑤ No

Xc210. [Cognitive function] He/she was confused about or didn’t know year, date and time. (morning, afternoon, evening, etc.)
① Yes
⑤ No

Xc211. [Cognitive function] He/she was confused about or didn’t know the place (home, hospital, etc) where he/she was.
① Yes
⑤ No

Xc212. [Cognitive function] He/she was not good at money, numerical and date calculations.
① Yes
⑤ No

Xc213. [Cognitive function] He/she didn't remember what happened or what he/she said a little while ago.
① Yes
⑤ No

Xc214. [Cognitive function] He/she didn’t remember what he/she said a few days ago.

① Yes

⑤ No

Xc215. [Cognitive function] He/she could not use familiar home appliances (TV, radio, etc.) alone.

① Yes

⑤ No

Xc216. [Cognitive function] It was impossible for him/her to use and learn new things.

① Yes

⑤ No

Xc217. [Cognitive function] He/she didn't understand the contents when watching television dramas, movies, news, etc., or when listening to the radio.

① Yes →Go to Xc301

⑤ No →Go to Xc301

Xc301. Now I have some questions about fall and pain. Did he/she ever fall down or trip over something after the previous interview?

① Yes

⑤ No →Go to Xc304

Logic

■ If Xc301=①, then go to Xc302~Xc303.

Xc302. How many times did he/she fall? (unit: a time)

_______times [range:1~730]

Xc303. Did he/she injure himself/herself seriously enough to need medical treatment?

① Yes

⑤ No

Xc304. Did he/she ever experience a fracture between the previous interview and his/her death?

① Yes

⑤ No →Go to Xc306

Xc305. Did he/she ever fracture his/her hip after the previous interview?

① Yes

⑤ No

Xc306. Did he/she suffer from pain during the one year before his/her death?

① Yes

⑤ No

Xc307. Now I have some questions about physical symptoms and health behavior, such as smoking and drinking.

Did he/she smoke after the previous interview?

① Yes

⑤ No

Xc308. Did he/she drink alcoholic beverages after the previous interview?

① Yes

③ No

⑤ could not drink

Xc309. How much did he/she weigh when he/she died? (unit: kg)

_________kg [range:30~200]

Xc310. Did he/she gain or lose a lot of weight during the two years before his/her death?

[IWER: Weight gain or loss means a change of more than 5 kg.]

① Yes, lost a lot

③ Yes, gained a lot

⑤ No

Xc311. Did he/she have difficulty breathing during the one month before his/her death?

① Yes

⑤ No

Xc312. Did he/she say that he/she had no appetite during the one month before his/her death?

① Yes

⑤ No

Xc313. Did he/she often vomit during the one month before his/her death?

① Yes

⑤ No

Xc314. Was he/she unable to keep himself/herself steady and use his/her limbs comfortably during the one month before his/her death?

① Yes

⑤ No

Xc315. Did he/she feel depressed during the one month before his/her death?

① Yes

⑤ No

Xc316. Did he/she periodically fall into a semiconscious or confused state during the one month before his/her death?

① Yes

⑤ No

Xc317. Did he/she feel seriously tired and exhausted during the one month before his/her death?

① Yes

⑤ No

Xc318. Did he/she have difficulty getting up and waking up or often lose his/her consciousness during the one month before his/her death?

① Yes

⑤ No

Xc319. Did he/she keep coughing or have much sputum during the one month before his/her death?

① Yes

⑤ No

Xc320. Was he/she unable to control his/her anger or feelings or did he/she use to explode emotionally during the one month before his/her death?

① Yes

⑤ No

Xc321. Was he/she unable to control urination and defecation during the one month before his/her death?

① Yes →Go to Xc401

⑤ No →Go to Xc401

Xc401. How many days was he/she bedridden for more than half the day during the three months before his/her death? (unit: a day)
________days [range:0~93]

Xc402. Did he/she need help with dressing during the three months before his/her death?
① Yes, needed help
⑤ No, didn't need help

Xc403. Did he/she need help with washing him/her face and hair and brushing him/her teeth during the three months before his/her death?
① Yes, needed help
⑤ No, didn't need help

Xc404. Did he/she need help with bathing or showering during the three months before his/her death?
① Yes, needed help
⑤ No, didn't need help

Xc405. Did he/she need help with eating during the three months before his/her death?
① Yes, needed help
⑤ No, didn't need help

Xc406. Did he/she need help with getting out of bed and walking out of the room during the three months before his/her death?
① Yes, needed help
⑤ No, didn't need help

Xc407. Did he/she need help with using the toilet (taking off one's clothes and cleaning oneself after urinating and defecating) during the three months before his/her death?
① Yes, needed help
⑤ No, didn't need help

Xc408. Did he/she need help with controlling urination and defecation during the three months before his/her death?
① Yes, needed help
⑤ No, didn't need help

Logic

■ If R has answered yes to at least one of Xc402~Xc408, then go to Xc409~Xc416.

Xc409. If he/she needed help with activities of daily living, who helped him/her the most? Please state the help giver's relationship to him/her.

② Spouse →Go to Xc411

③ Mother →Go to Xc411

④ Father →Go to Xc411

⑤ Mother-in-law →Go to Xc411

⑥ Father-in-law →Go to Xc411

⑦~㉖. Children →Go to Xc411

㉗~㊻. Sibling →Go to Xc411

㊼ Brother-in-law/sister-in-law →Go to Xc411

㊽ Son-in-law/daughter-in-law →Go to Xc411

㊾ Grandchild →Go to Xc411

㊿ Other relatives →Go to Xc411

(52) Voluntary worker →Go to Xc411

(53) Publicly provided helper →Go to Xc411

(54) Personally hired helper →Go to Xc411

(55) Others

(56) No helper →Go to Xc417

Xc410. If others, please specify.

____

Xc411. Did [Xc409] receive money for helping him/her?

① Yes

⑤ No

Xc412. Did [Xc409] have to take care of or nurse another person?

① Yes

⑤ No

Xc413. When [Xc409] was not able to take care of him/her due to illness or any other reason, was there any person who took care of him/her instead of [Xc409]?

① Yes

⑤ No →Go to Xc417

Xc414. How many people helped or were able to help him/her instead of [Xc409]?
_______ [range:1~97]

Xc415. Who helped or was able to help him/her instead of [Xc409]? Please state his/her relationship to the person who died.
[IWER: R may choose up to two persons in the order of priority.]
② Spouse →Go to Xc417
③ Mother →Go to Xc417
④ Father →Go to Xc417
⑤ Mother-in-law →Go to Xc417
⑥ Father-in-law →Go to Xc417
⑦~㉖. Children →Go to Xc417
㉗~㊻. Sibling →Go to Xc417
㊼ Brother-in-law/sister-in-law →Go to Xc417
㊽ Son-in-law/daughter-in-law →Go to Xc417
㊾ Grandchild →Go to Xc417
㊿ Other relatives →Go to Xc417
(52) Voluntary worker →Go to Xc417
(53) Publicly provided helper →Go to Xc417
(54) Personally hired helper →Go to Xc417
(55) Others

Xc416. If others, please specify.
____

Xc417. Did he/she need help with personal grooming (including brushing hair, clipping nails/toenails, putting on make-up and shaving) due to health problems, etc., during the three months before his/her death?
① Yes, needed help
③ No, didn't need help
⑤ He/she didn't do it before

Xc418. Did he/she need help with doing housework (cleaning, tidying up and washing dishes) due to health problems, etc., during the three months before his/her death?
① Yes, needed help

③ No, didn’t need help
⑤ He/she didn’t do it before

Xc419. Did he/she need help with preparing meals (preparing ingredients, cooking and setting the table) due to health problems, etc., during the three months before his/her death?
① Yes, needed help
③ No, didn’t need help
⑤ He/she didn’t do it before

Xc420. Did he/she need help with doing laundry due to health problems, etc., during the three months before his/her death?
① Yes, needed help
③ No, didn’t need help
⑤ He/she didn’t do it before

Xc421. Did he/she need help with going out a short distance without using transportation due to health problems, etc., during the three months before his/her death?
① Yes, needed help
③ No, didn’t need help
⑤ He/she didn’t do it before

Xc422. Did he/she need help with going out using transportation, such as buses, subways, taxies, and cars, due to health problems, etc., during the three months before his/her death?
① Yes, needed help
③ No, didn’t need help
⑤ He/she didn’t do it before

Xc423. Did he/she need help with shopping (paying money and getting change) due to health problems, etc., during the three months before his/her death?
① Yes, needed help
③ No, didn’t need help
⑤ He/she didn’t do it before

Xc424. Did he/she need help with managing his/her money (managing pocket money, bank accounts and assets) due to health problems, etc., during the three months before his/her death?
① Yes, needed help
③ No, didn’t need help

⑤ He/she didn’t do it before

Xc425. Did he/she need help with making phone calls due to health problems, etc., during the three months before his/her death?

① Yes, needed help

③ No, didn’t need help

⑤ He/she didn’t do it before

Xc426. Did he/she need help with taking medications due to health problems, etc., during the three months before his/her death?

① Yes, needed help

③ No, didn’t need help

⑤ He/she didn’t do it before

Logic

■ If R answered yes to at least one of Xc417~Xc426, then go to Xc427~Xc429.

Xc427. If he/she needed help with above activities of daily living, who helped him/her the most? Please state the help giver’s relationship to him/her.

② Spouse →Go to Xc429

③ Mother →Go to Xc429

④ Father →Go to Xc429

⑤ Mother-in-law →Go to Xc429

⑥ Father-in-law →Go to Xc429

⑦~㉖. Children →Go to Xc429

㉗~㊻ Sibling →Go to Xc429

㊼ Brother-in-law/sister-in-law →Go to Xc429

㊽ Son-in-law/daughter-in-law →Go to Xc429

㊾ Grandchild →Go to Xc429

㊿ Other relatives →Go to Xc429

(52) Voluntary worker →Go to Xc429

(53) Publicly provided helper →Go to Xc429

(54) Personally hired helper →Go to Xc429

(55) Others

(56) No one helped →Go to Xc501

Xc428. If others, please specify.

Xc429. Did [Xc427] receive money for helping him/her?
① Yes →Go to Xc501
⑤ No →Go to Xc501

Xc501. The next questions are about health insurance, What type of health insurance was he/she covered by during the one month before his/her death?
① National Health Insurance System
③ Medical Aid System →Go to Xc506
⑤ Not eligible for health insurance benefits due to long-term premium arrearage
Don’t know →Go to Xc507
Refuse to answer →Go to Xc507

Xc502. Did he/she enroll in the National Health Insurance System through (his/her or his/her family member’s) employer or the community?
① Through an employer
⑤ Through the community →Go to Xc504

Logic
■ If Xc502=①, then go to Xc503.

Xc503. If he/she was insured through an employer, was he/she the insured person or a dependent family member?
① Insured person
⑤ Dependent family member

Logic
■ If Xc502=⑤, then go to Xc504.

Xc504. If he/she was insured through the community, was he/she the household head?
① Yes
⑤ No

Xc505. Who paid his/her health insurance premiums during the one month before his/her death?
① He/she
③ Spouse

⑤ Son/daughter

⑦ Son-in-law/daughter-in-law

⑨ Other relatives

Logic

■ If Xc501=③, then go to Xc506.

Xc506. Was he/she covered by Type 1 Medical Aid or Type 2 Medical Aid during the one month before his/her death?

① Type 1

⑤ Type 2

Xc507. Was he/she covered by any private health insurance to compensate for hospital expenses? Private health insurance means every health insurance, regardless of its type, even including those whose term expired. Please exclude health-related special policy conditions included in pension insurance or whole life insurance.

① Yes

⑤ No

Xc508. Now I have some questions about the medical institutions he/she used. Was he/she ever hospitalized after the previous interview?

① Yes

⑤ No →Go to Xc522

Logic

■ If Xc508=①, then go to Xc509~Xc521.

Xc509. How many times was he/she hospitalized after the previous interview? (unit: a time)

________time [range:1~52]

Xc510. How many days did he/she stay in hospital during his/her last hospitalization? (unit: a day)

_________days [range:1~730]

Xc511. Did he/she ever go to the intensive care unit during his/her stay in hospital?

① Yes

⑤ No

Xc512. Did he/she ever use life-prolonging treatment, such as an oxygen respirator, during his/her stay in hospital?

① Yes

⑤ No

Xc513. Did he/she ever receive kidney dialysis during his/her stay in hospital?

① Yes

⑤ No

Xc514. Did he/she take prescribed antibiotics during his/her stay in hospital?

① Yes

⑤ No

Xc515. What was the total amount of medical expenses paid for his/her hospitalization since the previous interview? (unit: 10,000 Korean won)

_________MW [range:1~9997]

Don't know →Go to Xc517

Refuse to answer →Go to Xc517

Xc516. How much did he/she pay out-of-pocket? Also, how much did his/her relatives or private health insurances pay on behalf of him/her? Please include in the private health insurance category any payments that he/she made and were reimbursed by private health insurances. (unit: 10,000 Korean won) Categories are ① out-of-pocket payments (Xc516a), ② payments by relatives including children and parents (Xc516b), ③ payments reimbursed by private health insurances (Xc516c).

ITEMS		AMOUNT
Xc516a	Out-of-pocket payments	
Xc516b	Payments by relatives including children and parents	
Xc516c	Payments reimbursed by private health insurances	
Total		[Xc515] MW

→ Go to Xc518 or Xc522

Logic

■ If Xc515='Don't know' or 'Refuse to answer', then go to Xc517.

Xc517. Approximately, how much did it cost? Please choose among the following amounts.

① Less than 50MW

② 50MW~500MW

③ 500MW~1000MW

④ 1,000MW~2,000MW

⑤ 2,000MW~5,000MW

⑥ More than 5,000MW

Logic

■ IfXc502=⑤, then go to Xc518~Xc521.

Xc518. If he/she died in hospital, how long did he/she stay in hospital until his/her death? (unit: a day)

______days [range:1~730]

Logic

■ If Xc518=①, then go to Xc519 .

Xc519. You have said that he/she stayed in hospital just one day. Then how many hours did he/she stay in hospital? (unit: an hour)

_______hours [range:1~24]

Xc520. Why was he/she hospitalized at that time?

① Surgery →Go to Xc522

③ Treatment except surgery →Go to Xc522

⑤ To control his/her symptoms →Go to Xc522

⑦ Others

Xc521. If others, please specify.

____

Logic

■ If Xc522≧1, then go to Xc523~Xc530. If Xc522=0, then go to Xc531.

Xc522. How many times did he/she enter a nursing home, sanatorium, silver town, etc., after the previous interview? (unit: a time)

______time [range:0~52] *0(zero) →Go to Xc531

Xc523. How many days did he/she stay there during his/her last stay? (unit: a day)

_______days [range:1~730]

Xc524. What was the total amount paid to the elderly care facility since the previous interview? (unit: 10,000 Korean won))

__________MW [range:1~99997]

Don’t know →Go to Xc526

Refuse to answer →Go to Xc526

Xc525. How much did he/she pay out-of-pocket? Also, how much did his/her relatives or private health insurances pay on behalf of him/her? Please include in the private health insurance category any payments that he/she made and were reimbursed by private health insurances. (unit: 10,000 Korean won) Categories are ① out-of-pocket payments (Xc525a), ② payments by relatives including children and parents (Xc525b), ③ payments reimbursed by private health insurances (Xc525c).

ITEMS		AMOUNT
Xc525a	Out-of-pocket payments	
Xc525b	Payments by relatives including children and parents	
Xc525c	Payments reimbursed by private health insurances	
Total		[Xc524] MW

→Go to Xc527 or Go to Xc529

Logic

■ If Xc524='Don’t know’ or ‘Refuse to answer’, then go to Xc526.

Xc526. Approximately, how much did it cost? Please select after listening to the following amounts.

① Less than 50MW

② 50MW~Less than 500MW

③ 500MW~Less than 1000MW

④ 1,000MW~Less than 2,000MW

⑤ 2,000MW~Less than 5,000MW

⑥ More than 5,000MW

Logic

■ If Xa011=⑦, then go to Xc527~Xc528. If not, then go to Xc529.

Xc527. If he/she died at an elderly care facility, how long did he/she stay there at that time? (unit: a day)

______days [range:1~730]

Xc528. When did he/she enter that elderly care facility? (unit: year and month combined into 6 digits) [IWER: Enter the year and month using 6 digits. For example, mark 200701 for January 2007. If the month is not clear, enter 200700.]

__________ [range: 200600~200812] →Go to Xc531

Xc529. If he/she stayed but did not die at the elderly care facility, where did he/she stay until his/her death after leaving the facility?

① Lived alone →Go to Xc531

② Lived with his/her spouse → Go to Xc531

③ Lived with his/her child or child's family → Go to Xc531

④ Lived with other relatives → Go to Xc531

⑤ He/she was hospitalized → Go to Xc531

⑥ Others

Xc530. If others, please specify.

____

Logic

■ If Xc531≧1, then go to Xc532. If Xc531=0, then go to Xc541.

Xc531. How many times was he/she hospitalized in a hospice facility after the previous interview? (unit: a time)

______time [range: 0~52] * 0 (zero) →Go to Xc541

Xc532. How many days did he/she stay there during his/her last stay? (unit: a day)

______days [range:1 ~730]

Xc533. What was the total amount paid to the hospice facility since the previous interview? (unit: 10,000 Korean won)

__________MW [range:1~99997]

Don’t know →Go to Xc535

Refuse to answer →Go to Xc535

Xc534. How much did he/she pay out-of-pocket? Also, how much did his/her relatives or private health insurances pay on behalf of him/her? Please include in the private health insurance category any payments that he/she made and were reimbursed by private health insurances. (unit: 10,000 Korean won) Categories are ① out-of-pocket payments (Xc534a), ② payments by relatives including children and parents (Xc534b), ③ payments reimbursed by private health insurances (Xc534c).

ITEMS		AMOUNT
Xc534a	Out-of-pocket payments	
Xc534b	Payments by relatives including children and parents	
Xc534c	Payments reimbursed by private health insurances	
Total		[Xc533] MW

→Go to Xc536

Logic

■ If Xc533=’Don’t know’ or ’Refuse to answer’, then go to Xc535.

Xc535. Approximately, how much did it cost? Please select after listening to the following amounts.

① Less than 50MW

② 50MW~500MW

③ 500MW~1000MW

④ 1,000MW~2,000MW

⑤ 2,000MW~5,000MW

⑥ More than 5,000MW

Xc536. Did he/she die at a hospice unit in a hospital or a specialized hospice facility?

① Yes

⑤ No →Go to Xc539

Xc537. If he/she died at a hospice unit or specialized hospice facility, how long did he/she stay there at that time? (unit: a day)

______days [range:1~730]

Xc538. When did he/she enter that facility? (unit: year and month combined into 6 digits)
[IWER: Enter the year and month using 6 digits. For example, mark 200701 for January 2007. If the month is not clear, enter 200700.]
_________ [range: 200600~200812] →Go to Xc541

Logic
■ If Xc536=⑤, then go to Xc539.

Xc539. If he/she did not die at the hospice unit or hospice facility, where did he/she stay after leaving that facility?
① Lived alone →Go to Xc541
② Lived with his/her spouse →Go to Xc541
③ Lived with his/her child or child's family →Go to Xc541
④ Lived with other relatives →Go to Xc541
⑤ He/she was hospitalized →Go to Xc541
⑥ Others

Xc540. If others, please specify.

____

Xc541. Now I have some questions about the outpatient services he/she used. Did he/she ever see a doctor in a clinic or hospital for outpatient care after the previous interview?
① Yes
⑤ No →Go to Xc543

Xc542. How many times did he/she receive outpatient care after the previous interview? (unit: a time)
_____time [range: 1~730]

Xc543. Did he/she ever go to a hospital emergency room after the previous interview?
① Yes
⑤ No →Go to Xc545

Xc544. How many times did he/she go to an emergency room after the previous interview? (unit: a time)
_____time [range:1~730]

Logic

■ If Xc541=① or Xc543=①, then go to Xc545~Xc547.

Xc545. What was the total amount of medical expenses paid for using the clinic or hospital outpatient services and emergency room since the previous interview? (unit: 10,000 Korean won)

__________MW [range:1~99997]

Don't know →Go to Xc547

Refuse to answer →Go to Xc547

Xc546. How much did he/she pay out-of-pocket? Also, how much did his/her relatives or private health insurances pay on behalf of him/her? Please include in the private health insurance category any payments that he/she made and were reimbursed by private health insurances. (unit: 10,000 Korean won) Categories are ① out-of-pocket payments (Xc546a), ② payments by relatives including children and parents (Xc546b), ③ payments reimbursed by private health insurances (Xc546c).

ITEMS		AMOUNT
Xc546a	Out-of-pocket payments	
Xc546b	Payments by relatives including children and parents	
Xc546c	Payments reimbursed by private health insurances	
Total		[Xc545] MW

→Go to Xc548

Logic

■ If Xc545='Don't know' or 'Refuse to answer', then go to Xc547.

Xc547. Approximately, how much did it cost? Please select after listening to the following amounts.

① Less than 50MW

② 50MW~500MW

③ 500MW~1000MW

④ 1,000MW~2,000MW

⑤ 2,000MW~5,000MW

⑥ More than 5,000MW

Xc548. Did he/she take any medication on a regular basis before his/her death?

① Yes
⑤ No →Go to Xc552

Xc549. What was the total amount paid for such medications since the previous interview? (unit: 10,000 Korean won)
__________MW [range:1~9997]
Don't know →Go to Xc551
Refuse to answer →Go to Xc551

Xc550. How much did he/she pay out-of-pocket? Also, how much did his/her relatives or private health insurances pay on behalf of him/her? Please include in the private health insurance category any payments that he/she made and were reimbursed by private health insurances. (unit: 10,000 Korean won) Categories are ① out-of-pocket payments (Xc550a), ② payments by relatives including children and parents (Xc550b), ③ payments reimbursed by private health insurances (Xc550c).

ITEMS		AMOUNT
Xc550a	Out-of-pocket payments	
Xc550b	Payments by relatives including children and parents	
Xc550c	Payments reimbursed by private health insurances	
Total		[Xc549] MW

→Go to Xc552

Logic
■ If Xc549='Don't know' or 'Refuse to answer', then go to Xc551.

Xc551. Approximately, how much did it cost? Please choose among the following amounts.
① Less more 5MW
② 5MW~10MW
③ 10MW~50MW
④ 50 MW~100MW
⑤ 100MW~200MW
⑥ More than 200MW

Xc552. Did he/she ever receive a house call or visiting nursing services provided directly by a doctor

or nurse (health care provider) after the previous interview? Health care provider refers to doctors, nurses, visiting nurse personnel, physical therapists, chemical therapists, oxygen therapists, etc. Caregiver services for the purpose of taking care of patients not of treating them are excluded.

① Yes

⑤ No →Go to Xc557

Xc553. How many times did he/she receive such visiting health care after the previous interview? (unit: a time)

______time [range: 1~730]

Xc554. What was the total amount paid for the house calls or visiting nursing services since the previous interview? (unit: 10,000 Korean won)? (unit: 10,000 Korean won)

__________MW [range: 1~9997]

Don't know →Go to Xc556

Refuse to answer →Go to Xc556

Xc555. How much did he/she pay out-of-pocket? Also, how much did his/her relatives or private health insurances pay on behalf of him/her? Please include in the private health insurance category any payments that he/she made and were reimbursed by private health insurances. (unit: 10,000 Korean won) Categories are ① out-of-pocket payments (Xc555a), ② payments by relatives including children and parents (Xc555b), ③ payments reimbursed by private health insurances (Xc555c).

ITEMS		AMOUNT
Xc555a	Out-of-pocket payments	
Xc555b	Payments by relatives including children and parents	
Xc555c	Payments reimbursed by private health insurances	
Total		[Xc554] MW

→Go to Xc557

Logic

■ If Xc554 ='Don't know' or 'Refuse to answer', then go to Xc556.

Xc556. Approximately, how much did it cost? Please choose among the following amounts.

① Less than 50MW

② 50MW~ 500MW

③ 500MW~ 1000MW

④ 1,000MW~ 2,000MW

⑤ 2,000MW~ 5,000MW

⑥ More than 5,000MW

Xc557. Did he/she ever receive or use services provided by an elderly welfare facility or a local government or public health clinic after the previous interview?
For example, those services include side dish delivery services, elderly companionship services, counseling services by social workers, medical services, rehabilitation therapy, etc., provided at welfare centers.

① Yes

⑤ No

Xc558. Aside from the medical expenses mentioned earlier, did he/she purchase any uninsured items, such as medical instruments (patient beds or duvet, wheelchairs, sticks, etc.) or oriental medicine or nutritional supplements (medication not prescribed by a doctor), and patient food (supplementary health food), after the previous interview?

① Yes

⑤ No →Go to Xc562

Xc559. What was the total amount paid for purchasing such items since the previous interview? (unit: 10,000 Korean won)

__________MW [range:1~ 9997]

Don't know →Go to Xc561

Refuse to answer →Go to Xc561

Xc560. How much did he/she pay out-of-pocket? Also, how much did his/her relatives or private health insurances pay on behalf of him/her? Please include in the private health insurance category any payments that he/she made and were reimbursed by private health insurances. (unit: 10,000 Korean won) Categories are ① out-of-pocket payments (Xc560a), ② payments by relatives including children and parents (Xc560b), ③ payments reimbursed by private health insurances (Xc560c).

ITEMS		AMOUNT
Xc560a	Out-of-pocket payments	

Xc560b	Payments by relatives including children and parents	
Xc560c	Payments reimbursed by private health insurances	
Total		[Xc559] MW

→Go to Xc562

Logic

■ If Xc559='Don't know' or 'Refuse to answer', then go to Xc561.

Xc561. Approximately, how much did it cost? Please choose among the following amounts.

① Less than 50MW

② 50MW~500MW

③ 500MW~1000MW

④ 1,000MW~2,000MW

⑤ 2,000MW~5,000MW

⑥ More than 5,000MW

Xc562. Who mainly paid the medical expenses incurred before his/her death after the previous interview?

① He/she →Go to Xc564

③ Spouse →Go to Xc564

⑤ Child or child's family (including grandchild) →Go to Xc564

⑦ Other relatives →Go to Xc564

⑨ Others

Xc563. If others, please specify.

____

Xc564. Was there any person, except [Xc562], who provided financial support to cover the medical expenses?

① Yes

⑤ No, did →Go to Xc601

Xc565. Who was that person?

① He/she →Go to Xc601

③ Spouse →Go to Xc601

⑤ Child or child's family (including grandchild) →Go to Xc601
⑦ Other relatives →Go to Xc601
⑨ Others

Xc566. If others, please specify.

____

Xc601. Now I have some questions about his/her funeral and impacts on bereaved family members' health. Where was he/she buried?
① Cemetery →Go to Xc603
③ Family gravesite →Go to Xc603
⑤ Charnel house →Go to Xc603
⑦ Others

Xc602. If others, please specify.

____

Xc603. How much did his/her funeral cost? (unit: 10,000 Korean won)
______MW [range:0~99997]

Xc604. Was there any bereaved family member who received psychological treatment due to nervous disorder, depression, etc. after his/her death?
① Yes
⑤ No →Go to section Xd.

Xc605. Who received psychological treatment?
② Spouse →Go to section Xd.
③ Mother →Go to section Xd.
④ Father →Go to section Xd.
⑤ Mother-in-law →Go to section Xd.
⑥ Father-in-law →Go to section Xd.
⑦~㉖. Children →Go to section Xd.
㉗~㊻. Sibling →Go to section Xd.
㊼ Brother-in-law/sister-in-law→Go to section Xd.
㊽ Son-in-law/daughter-in-law →Go to section Xd.
㊾ Grandchild →Go to section Xd.
㊿ Other relatives →Go to section Xd.

(55) Others

Xc606. If others, please specify.

____

Xd. EMPLOYMENT

Logic

■ In previous interview, if a respondent had a job, then go to Xd001. If a respondent did not have a job, ten go to Xd002

Xd001. He/she responded in the previous interview that he/she was working as [job title: occupation] at [name of workplace: industry]. Was it his/her last job before his/her death?

① Yes →Go to Xd003

⑤ No →Go to Xd003

Don't know →Go to section Xf.

Refuse to answer →Go to section Xf.

Xd002. He/she responded in the previous interview that he/she was not working. Did he/she ever work before his/her death after that interview?

① Yes →Go to Xd005

⑤ No → Go to section Xf.

Don't know →Go to section Xf.

Refuse to answer →Go to section Xf.

Xd003. When did he/she stop working at that job? (unit: year and month combined into 6 digits)

[IWER: Enter the year and month using 6 digits. For example, mark 200701 for January 2007. If the month is not clear, enter 200700.]

____________________ [range:200600 ~ 200812]

Xd004. Why did he/she quit?

(in the case of wage workers)

① Bankruptcy, closure, temporary shutdown, etc., of business

② Layoff, dehiring, voluntary early retirement, etc.

③ Contract expired

④ Retirement (at the mandatory retirement age)

⑤ Too low wage

⑥ Work was temporary or did not have a good prospect

⑦ Aptitude, knowledge, and skills did not match well

⑧ Long working hours/unfavorable working conditions

⑨ Marriage, childbearing, parenting, nursing, etc.

⑩ Study or military service

⑪ Found a better job

⑫ To start up a new business

⑬ Poor health

⑭ Wanted to take a break

⑮ He/she died

⑯ Others

(in the case of non-wage workers)

① Business was slow

② Work did not have a good prospect

③ Aptitude, knowledge, and skills did not match well

④ Long working hours/unfavorable working conditions

⑤ Marriage, childbearing, parenting, nursing, etc.

⑥ Moved house

⑦ Study or military service

⑧ Found a paid job

⑨ To start up a new business

⑩ For unpaid assistance in family business

⑪ Poor health

⑫ Wanted to take a break

⑬ He/she died

⑭ Others

Logic

■ If Xd001=⑤ or Xd002=①, then go to Xd005~Xd027. Until Xd025=①, loop Xd005~Xd027.

Xd005. (As loop=1 & Xd002=①) When did he/she start the job? / (As loop=1 & Xd001=⑤ or loop>=2) I am going to ask you about his/her new job. When did he/she start the job? (unit: year and month combined into 6 digits)

[INT: Enter the year and month using 6 digits. For example, mark 200701 for January 2007. If the month is not clear, enter 200700.]

__________________[range:200600 ~ 200812]

Xd006. What was his/her new employment status?

① Regular worker

② Temporary worker

③ Casual worker

④ Employer

⑤ Self-employed

⑥ Unpaid family worker

Xd007. Which industry was the workplace engaged in?

① Agriculture/forestry →Go to Xd012

② Fishing →Go to Xd012

③ Mining and quarrying →Go to Xd012

④ Manufacturing

⑤ Electricity, gas, and water supply →Go to Xd012

⑥ Construction →Go to Xd012

⑦ Wholesale and retail trade →Go to Xd009

⑧ Hotels and restaurants →Go to Xd010

⑨ Transport →Go to Xd012

⑩ Post and telecommunications →Go to Xd012

⑪ Financial and insurance activities →Go to Xd012

⑫ Real estate and renting and leasing →Go to Xd012

⑬ Other activities →Go to Xd011

Xd008. Specifically speaking, which industrial classification best describes the workplace?

① Manufacture of food products and beverages

② Manufacture of textiles, wearing apparel, luggage, and footwear

③ Manufacture of metal and machinery materials and products

④ Manufacture of electronic and electric materials and products

⑤ Manufacture of motor vehicles and transport equipment products

⑥ Manufacture of other products

→Go to Xd012

Xd009. Specifically speaking, which industrial classification best describes the workplace?

① Sale of motor vehicles

② Wholesale

③ Retail trade

→Go to Xd012

Xd010. Specifically speaking, which industrial classification best describes the workplace?

① Hotels

② Restaurants

→Go to Xd012

Xd011. Specifically speaking, which industrial classification best describes the workplace?

① Business services

② Public administration, defense, and compulsory social security

③ Education

④ Health and social work

⑤ Recreational, culture, and sports related activities

⑥ Other community, repair, and personal services

⑦ Housekeeping services

⑧ International organizations

Xd012. What kind of work did he/she do in the workplace?

① Managers →(wage workers) Go to Xd017, (non-wage workers) Go to Xd019

② (Associate) Professionals →(wage workers) Go to Xd017, (non-wage workers) Go to Xd019

③ Clerks

④ Service workers →Go to Xd014

⑤ Sales workers →(wage workers) Go to Xd017, (non-wage workers) Go to Xd019

⑥ Skilled agricultural, forestry, and fishery workers →Go to Xd015

⑦ Craft and related trades workers →(wage workers) Go to Xd017, (non-wage workers) Go to Xd019

⑧ Equipment, machine operating and assembling workers →(wage workers) Go to Xd017, (non-wage workers) Go to Xd019

⑨ Elementary workers →Go to Xd016

⑩ Career soldiers →(wage workers) Go to Xd017, (non-wage workers) Go to Xd019

Xd013. Specifically speaking, which occupational classification best describes his/her job?

① General office clerks

② Customer services clerks

→(wage workers) Go to Xd017, (non-wage workers) Go to Xd019

Xd014. Specifically speaking, which occupational classification best describes his/her job?

① Personal services related workers

② Cooking and food services workers

③ Travel and transport related workers

④ Protective service workers

→(wage workers) Go to Xd017, (non-wage workers) Go to Xd019

Xd015. Specifically speaking, which occupational classification best describes his/her job?

① Skilled agricultural and livestock workers

② Skilled forestry workers

③ Skilled fishery workers

→(wage workers) Go to Xd017, (non-wage workers) Go to Xd019

Xd016. Specifically speaking, which occupational classification best describes his/her job?

① Housekeeping, cleaning, and laundry workers

② Building management and security related workers

③ Delivery, moving, and meter reading workers

④ Sanitation workers

⑤ Other services related elementary workers

⑥ Agriculture, forestry, and fishery related elementary workers

⑦ Manufacturing related elementary workers

⑧ Mining and construction related elementary workers

⑨ Transport related elementary workers

Logic

■ If Xd006=①, ②, or ③, then go to Xd017~Xd018.

Xd017. How many employees worked at all locations of the workplace including the head office, branch offices, branches, plants, and the field? (unit: number of people, workplace since [Xd005]: [Industry Xd007] [Occupation Xd012])

______________People [range:1~9997] →Go to Xd022

Don't know

Refuse to answer

Xd018. Then what is the closest approximation to the actual number of employees that worked at all locations of the workplace including the head office, branch offices, branches, plants, and the field? (unit: number of people, workplace since [Xd005]: [Industry Xd007] [Occupation Xd012])

① Fewer than 5 people

② 5 to 9 people

③ 10 to 29 people

④ 30 to 49 people

⑤ 50 to 99 people

⑥ 100 to 299 people

⑦ 300 to 499 people

⑧ 500 to 999 people

⑨ More than 1,000 people

Logic

■ If Xd006=④, ⑤, or ⑥, then go to Xd019~Xd021.

Xd019. How many officially paid employees did he/she have? Please exclude temporary workers employed in farming seasons or busy times (unit: number of people, workplace since [Xd005]: [Industry Xd007] [Occupation Xd012])

[IWER: Mark 0 if there was no employee.]

_________people [range:0~9997] →Go to Xd021

Don't know

Refuse to answer

Xd020. Then what is the closest approximation to the actual number of employees in his/her business? (workplace since [Xd005]: [Industry Xd007] [Occupation Xd012])

① Fewer than 5 people

② 5 to 9 people

③ 10 to 29 people

④ 30 to 49 people

⑤ 50 to 99 people

⑥ 100 to 299 people

⑦ 300 to 499 people

⑧ 500 to 999 people

⑨ More than 1,000 people

Xd021. Did he/she have unpaid family workers other than officially paid employees? (unit: number of people, workplace since [Xd005]: [Industry Xd007] [Occupation Xd012])

[IWER: Mark 0 if there was no employee.]

_______people [range:0~20]

Xd022. How many days per week did he/she work? (unit: days, workplace since [Xd005]: [Industry Xd007] [Occupation Xd012])

_____days [range:1~7]

Xd023. How many hours per week did he/she work excluding lunch time, break, etc.? (unit: hours, workplace since [Xd005]: [Industry Xd007] [Occupation Xd012])

_____hours [range:1~160]

Logic

■ If Xd006=①, ②, ③, ④, or ⑤, then go to Xd024.

Xd024. How much profit per month did he/she earn from this business? Please state a net profit excluding wages and expenses. (Unit: 10,000 Korean won, workplace since [Xd005]: [Industry Xd007] [Occupation Xd012])

[IWER: Mark 0 if there was no net profit, and mark 999997 if he/she ran a deficit.]

_____MW [range:0~999997]

Xd025. Was it his/her last job before his/her death? (workplace since [Xd005]: [Industry Xd007] [Occupation Xd012])

① Yes

⑤ No

Xd026. When did he/she stop working? (unit: year and month combined into 6 digits, workplace since [Xd005]: [Industry Xd007] [Occupation Xd012])

[IWER: Enter the year and month using 6 digits. For example, mark 200701 for January 2007. If the month is not clear, enter 200700.]

________________________[range:200600 ~ 200812]

Xd027. Why did he/she quit?

(workplace since [Xd005]: [Industry Xd007] [Occupation Xd012])

(in the case of wage workers)

① Bankruptcy, closure, temporary shutdown, etc. of business

② Layoff, dehiring, voluntary early retirement, etc.

③ Contract expired

④ Retirement (at the mandatory retirement age)

⑤ Too low wage

⑥ Work was temporary or did not have a good prospect

⑦ Aptitude, knowledge, and skills did not match well

⑧ Long working hours/unfavorable working conditions

⑨ Marriage, childbearing, parenting, nursing, etc.

⑩ Study or military service

⑪ Found a better job

⑫ To start up a new business

⑬ Poor health

⑭ Wanted to take a break

⑮ He/she died

⑯ Others

(in the case of non-wage workers)

① Business was slow

② Work did not have a good prospect

③ Aptitude, knowledge, and skills did not match well

④ Long working hours/unfavorable working conditions

⑤ Marriage, childbearing, parenting, nursing, etc.

⑥ Moved house

⑦ Study or military service

⑧ Found a paid job

⑨ To start up a new business

⑩ For unpaid assistance in family business

⑪ Poor health

⑫ Wanted to take a break

⑬ He/she died

⑭ Others

Xf. ASSETS AND DEBTS

Xf001. Did he/she leave any inheritance?

① Yes

⑤ No →Go to Xf037

Xf002. Did he/she leave his/her house as an inheritance?

① Yes

⑤ No →Go to Xf009

Xf003. What was the value? (unit: 10,000 Korean won)

_____MW [range:0~9999997] →Go to Xf009

Don’t know →Go to Xf004~Xf008 unfolding

Refuse to answer →Go to Xf004~Xf008 unfolding

< Xf004~Xf008. Unfolding bracket questions >

Xf004. Did it amount to less than, about equal to or more than 1,000 MW (10,000 Korean won)?

① Less than 1,000 MW

③ About 1,000 MW

⑤ More than 1,000 MW

Xf005. Did it amount to less than, about equal to, or more than 5,000 MW (10,000 Korean won)?

① Less than 5,000 MW

③ About 5,000 MW

⑤ More than 5,000 MW

Xf006. Did it amount to less than, about equal to or more than 10,000 MW (10,000 Korean won)?

① Less than 10,000 MW

③ About 10,000 MW

⑤ More than 10,000 MW

Xf007. Did it amount to less than, about equal to or more than 50,000 MW (10,000 Korean won)?

① Less than 50,000 MW

③ About 50,000 MW

⑤ More than 50,000 MW

Xf008. Did it amount to less than, about equal to or more than 100,000 MW (10,000 Korean won)?
① Less than 100,000 MW
③ About 100,000 MW
⑤ More than 100,000 MW

Xf009. Did he/she leave any other property except his/her house?
[IWER: Other property includes houses or buildings, studio apartments (Officetels), second houses, forest land, land, apartment or Officetel ownership, condominium membership, etc.]
① Yes
⑤ No →Go to Xf016

Xf010. What was the value? (unit: 10,000 Korean won)
_____MW [range:1~9999997] →Go to Xf016
Don't know →Go to Xf011~Xf015 unfolding
Refuse to answer →Go to Xf011~Xf015 unfolding

< Xf011~Xf015. Unfolding bracket questions >
Xf011. Did it amount to less than, about equal to or more than 1,000 MW (10,000 Korean won)?
① Less than 1,000 MW
③ About 1,000 MW
⑤ More than 1,000 MW

Xf012. Did it amount to less than, about equal to, or more than 5,000 MW (10,000 Korean won)?
① Less than 5,000 MW
③ About 5,000 MW
⑤ More than 5,000 MW

Xf013. Did it amount to less than, about equal to or more than 10,000 MW (10,000 Korean won)?
① Less than 10,000 MW
③ About 10,000 MW
⑤ More than 10,000 MW

Xf014. Did it amount to less than, about equal to or more than 50,000 MW (10,000 Korean won)?
① Less than 50,000 MW
③ About 50,000 MW
⑤ More than 50,000 MW

Xf015. Did it amount to less than, about equal to or more than 100,000 MW (10,000 Korean won)?
① Less than 100,000 MW
③ About 100,000 MW
⑤ More than 100,000 MW

Xf016. Did he/she leave any financial assets?
[IWER: Financial assets include cash over 50 MW (10,000-Korean won), bank savings, stocks /investment trust funds / mutual funds, bonds, insurance, private money lending, private savings club, etc.]
① Yes
⑤ No →Go to Xf023

Xf017. What was the value? (unit: 10,000 Korean won)
_____MW [range:1~999997] →Go to Xf023
Don't know →Go to Xf018~Xf022 unfolding
Refuse to answer →Go to Xf018~Xf022 unfolding

< Xf018~Xf022. Unfolding bracket questions >
Xf018. Did it amount to less than, about equal to or more than 100 MW (10,000 Korean won)?
① Less than 100 MW
③ About 100 MW
⑤ More than 100 MW

Xf019. Did it amount to less than, about equal to, or more than 500 MW (10,000 Korean won)?
① Less than 500 MW
③ About 500 MW
⑤ More than 500 MW

Xf020. Did it amount to less than, about equal to or more than 1,000 MW (10,000 Korean won)?
① Less than 1,000 MW
③ About 1,000 MW
⑤ More than 1,000 MW

Xf021. Did it amount to less than, about equal to or more than 5,000 MW (10,000 Korean won)?
① Less than 5,000 MW
③ About 5,000 MW
⑤ More than 5,000 MW

Xf022. Did it amount to less than, about equal to or more than 10,000 MW (10,000 Korean won)?
① Less than 10,000 MW
③ About 10,000 MW
⑤ More than 10,000 MW

Xf023. Did he/she have any insurance that pays compensation upon death?
[IWER:
- Term Life Insurance: A type of life insurance which covers the subscriber for a limited period of time.
- Whole Life Insurance: A type of life insurance which covers the subscriber until his/her death rather than for a limited period of time.
- Annuity Insurance: A type of life insurance in which the subscriber is paid a certain amount each year until his/her death or for a certain period of time.]
① Yes
⑤ No →Go to Xf030

Xf024. How much did his/her descendants receive in insurance benefits? (unit: 10,000 Korean won)
_____MW [range:1~9999997] →Go to Xf030
Don't know →Go to Xf025~Xf029 unfolding
Refuse to answer →Go to Xf025~Xf029 unfolding

< Xf025~Xf029. Unfolding bracket questions >
Xf025. Did it amount to less than, about equal to or more than 500 MW (10,000 Korean won)?
① Less than 500 MW
③ About 500 MW
⑤ More than 500 MW

Xf026. Did it amount to less than, about equal to or more than 1,000 MW (10,000 Korean won)?
① Less than 1,000 MW
③ About 1,000 MW
⑤ More than 1,000 MW

Xf027. Did it amount to less than, about equal to or more than 3,000 MW (10,000 Korean won)?
① Less than 3,000 MW
③ About 3,000 MW
⑤ More than 3,000 MW

Xf028. Did it amount to less than, about equal to or more than 5,000 MW (10,000 Korean won)?

① Less than 5,000 MW

③ About 5,000 MW

⑤ More than 5,000 MW

Xf029. Did it amount to less than, about equal to or more than 10,000 MW (10,000 Korean won)?

① Less than 10,000 MW

③ About 10,000 MW

⑤ More than 10,000 MW

Xf030. Apart from the inheritances you have mentioned so far, did he/she leave any other inheritances? For example, vehicles, antiques, paintings or membership etc.

① Yes

⑤ No →Go to Xf037

Xf031. What was the value? (unit: 10,000 Korean won)

_____MW [range:1~9999997] →Go to Xf037

Don't know →Go to Xf032~Xf036 unfolding

Refuse to answer →Go to Xf032~Xf036 unfolding

< Xf032~Xf036. Unfolding bracket questions >

Xf032. Did it amount to less than, about equal to or more than 500 MW (10,000 Korean won)?

① Less than 500 MW

③ About 500 MW

⑤ More than 500 MW

Xf033. Did it amount to less than, about equal to or more than 1,000 MW (10,000 Korean won)?

① Less than 1,000 MW

③ About 1,000 MW

⑤ More than 1,000 MW

Xf034. Did it amount to less than, about equal to or more than 3,000 MW (10,000 Korean won)?

① Less than 3,000 MW

③ About 3,000 MW

⑤ More than 3,000 MW

Xf035. Did it amount to less than, about equal to or more than 5,000 MW (10,000 Korean won)?

① Less than 5,000 MW

③ About 5,000 MW

⑤ More than 5,000 MW

Xf036. Did it amount to less than, about equal to or more than 10,000 MW (10,000 Korean won)?

① Less than 10,000 MW

③ About 10,000 MW

⑤ More than 10,000 MW

Xf037. Next are questions about debts. Did he/she leave any debt?

① Yes

⑤ No →Go to Xf044

Xf038. What was his/her total debt? (unit: 10,000 Korean won)

_____MW [range:1~9999997] →Go to Xf044

Don't know →Go to Xf039~Xf043 unfolding

Refuse to answer →Go to Xf039~Xf043 unfolding

< Xf039~Xf043. Unfolding bracket questions >

Xf039. Did it amount to less than, about equal to or more than 1,000 MW (10,000 Korean won)?

① Less than 1,000 MW

③ About 1,000 MW

⑤ More than 1,000 MW

Xf040. Did it amount to less than, about equal to or more than 3,000 MW (10,000 Korean won)?

① Less than 3,000 MW

③ About 3,000 MW

⑤ More than 3,000 MW

Xf041. Did it amount to less than, about equal to or more than 5,000 MW (10,000 Korean won)?

① Less than 5,000 MW

③ About 5,000 MW

⑤ More than 5,000 MW

Xf042. Did it amount to less than, about equal to or more than 10,000 MW (10,000 Korean won)?

① Less than 10,000 MW

③ About 10,000 MW

⑤ More than 10,000 MW

Xf043. Did it amount to less than, about equal to or more than 30,000 MW (10,000 Korean won)?

① Less than 30,000 MW

③ About 30,000 MW

⑤ More than 30,000 MW

Xf044. Did he/she leave any will regarding inheritance?

① Yes

⑤ No

Xf045. How much did he/she leave for her/his spouse/ child/ parents/ grandchild and so on? (unit: %)

[IWER: The sum of all parts must be equal to 100.]

Heir	Percentage(%)
Spouse	
Child ([Child' name])	
Child ([Child' name])	
⋮	
Child ([Child' name])	
Mother/ Father	
Grandchild	
Sibling	
Other relatives	
Donation to society	
Total	100%
